# Allylic Amination of Alkenyl Alcohols: Simultaneous Control of Chemoselectivity and Enantioselectivity in Nitrene Transfer Using Ion-Paired Catalysts

**DOI:** 10.1021/acscatal.5c04140

**Published:** 2025-08-08

**Authors:** Hannah K. Adams, Alexander Fanourakis, Amit Dahiya, Ioana Băltăreţu, Robert J. Phipps

**Affiliations:** Yusuf Hamied Department of Chemistry, 150385 University of Cambridge, Lensfield Road, Cambridge CB2 1EW, U.K.

**Keywords:** rhodium, amination, nitrenoid, chemoselectivity, enantioselectivity

## Abstract

When alkene-containing substrates are functionalized using metal nitrenoid complexes, aziridination is typically the favored reaction outcome. In certain cases, careful catalyst control may permit allylic C–H amination, but enantioselective protocols are very rare. This work describes the use of ion-paired Rh paddlewheel complexes to perform enantioselective allylic amination, where the typically preferred aziridination outcome can be overcome by the directing effect operating between the catalyst and the alcohol functional group in the substrate. A survey of different functional groups reveals that in this case, alcohols provide the optimum directing effect, and we carry out a systematic study to elucidate the important features of the chiral Cinchona alkaloid-derived cation associated with the anionic rhodium dimer. This reveals that the key inherent structural features of the natural alkaloids are necessary to obtain high enantio- and chemoselectivity, including the basic quinoline nitrogen and the free alcohol with natural stereochemistry. It also reveals that chemoselectivity and enantioselectivity are intrinsically linked, fully in line with our hypothesis of the reaction being guided by attractive noncovalent interactions.

## Graphical abstract

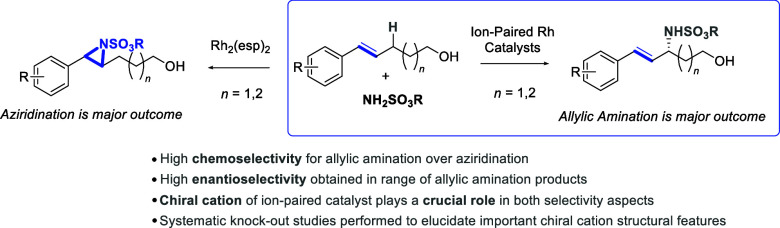

## 1 Introduction

Allylic amines are an important functional grouping and in some substitution patterns contain an amine stereocenter. The most widespread method to access this motif is via the displacement of an existing allylic leaving group.^1^ Alternatively, it can be obtained through direct functionalization of an allylic C–H bond, which is potentially more efficient but presents selectivity challenges.^2^ Various allylic C–H amination processes have been devised, and these can be approximately divided into methods that proceed via (1) π-allyl complex formation with hydride as the formal leaving group,^3^ (2) direct insertion of a metal nitrenoid into the allylic C–H bond,^4^ (3) sigmatropic rearrangements,^5^ and (4) radical mechanisms.^6^ Despite these advances, rendering allylic amination enantioselective is challenging, and there have been only a small number of successful strategies.^5b,7^

Catalysis using metal nitrenoids has become one of the key methodologies for direct amination of “activated” C–H bonds, typically benzylic, allylic, or tertiary.^4^ A major challenge when targeting allylic amination is that of chemoselectivity between aziridination and allylic C–H amination (Figure 1a).^8^ There have been important advances that achieve this in intramolecular settings, where ring closure factors have an influence.^9^ Intermolecular chemoselectivity poses greater challenges still and is rarer. In selected examples, chemoselective allylic amination using rhodium nitrenoids was reported by Dauban and co-workers using enantioenriched aminating agents in combination with chiral rhodium complexes, in some cases giving high diastereomeric excesses (Figure 1b, upper).^10^ Katsuki’s 2013 report using Ru-salen complexes still represents a landmark study, in which highly chemo-and enantioselective nitrene transfer was achieved (Figure 1b, upper middle).^11^ Ligand-switchable aziridination/allylic amination has been reported using silver nitrenoids by Schomaker, Berry, and co-workers, but this protocol was not extended to enantioselective methods (Figure 1b, lower middle).^8,12^ White and co-workers developed a bulky Mn-based catalyst for metallonitrene formation, which is highly selective for allylic amination, postulated to proceed via a stepwise radical mechanism (Figure 1b, lower).^3i^

**Figure 1:**
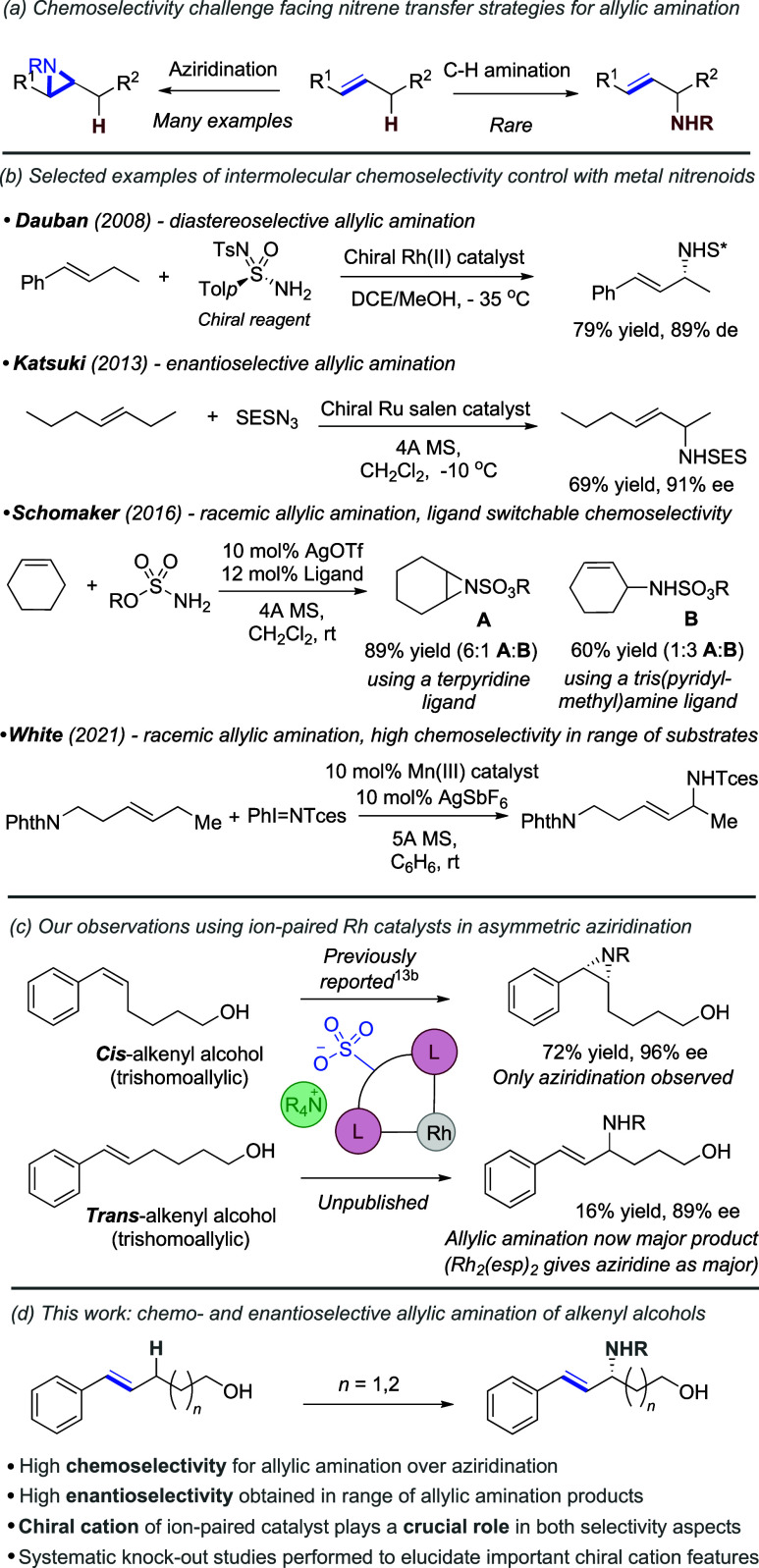
Background to chemoselectivity in allylic amination (a,b) and approach in this study (c,d).

We have developed a family of ion-paired Rh­(II,II) catalysts for enantioselective nitrene transfer in which the Rh complex is achiral but anionic and is paired with chiral cations derived from quaternized cinchona alkaloids.^13^ We propose that a network of attractive noncovalent interactions between ligand, cation, and substrate provides a high degree of organization within the chiral pocket provided by the cation.^14^ A functional group in the substrate is important to provide an interaction point with the catalyst, and we have had success using primary alcohols as directing groups for enantioselective amination of benzylic C–H bonds^13a,13c^ and aziridination of alkenes.^13b,13c^ During our studies on aziridination, it was apparent that alkenes with *cis*-geometry were excellent substrates, giving high yields and enantioselectivities across a variety of chain lengths between the alkene and the primary alcohol, including for trishomoallylic alcohols (Figure 1c, upper).^13b^ The corresponding *trans* isomers still gave aziridination but with relatively poor yields and enantioselectivities in the cases of homoallylic and bishomoallylic alcohols. Interestingly, we subsequently discovered that when the chain became longer in trishomoallylic alcohols, we observed a chemoselectivity switch to give allylic amination as the major product (Figure 1c, lower). Although the yield was low in part due to a challenging isolation, enantioselectivity was very high at 89% ee. In stark contrast, when amination was carried out on this substrate using Rh_2_(esp)_2_ as catalyst, aziridination was overwhelmingly preferred, suggesting that the ion-paired ligand was impacting chemoselectivity and enantioselectivity. Intrigued by the evidently powerful catalyst control in operation, we sought to fully explore the impact of the catalyst structure on the chemoselectivity and enantioselectivity and explore the scope of this process (Figure 1d).

## 2 Results and Discussion

We commenced our studies on phenyl-substituted trishomoallylic alcohol **1a** using **NH**_**2**_Pfps as the aminating agent (Table 1). As anticipated, amination of **1a** using Rh_2_(esp)_2_ as the catalyst resulted predominantly in aziridination to give **3a** (1:9 ratio **2a**/**3a**, entry 1). Switching to the ion-paired catalyst that was optimal in our previous aziridination, Rh_2_(**A**)_2_·(**4a**)_2_·(Pyr)_2_, produced a sizable swing in chemoselectivity toward allylic amination (2.7:1 ratio, entry 2) with major product **2a** being obtained in 85% ee. We next varied the geminal dialkyl groups on the achiral Rh­(II) dimers away from the methyl group (**B**–**D**) but saw little improvement (entries 3–5). We also evaluated catalyst **E** containing a phenyl linker between the central aryl ring of the ligand and the sulfonate group. In this case, the ee was high but selectivity between aziridination and allylic amination was poor (1.7:1, entry 6). Suspecting that electronic variation of the substrate aromatic ring could impact the innate preference between aziridination and allylic amination, we evaluated another substrate at this stage, **1b**, which contains an acetyl group at the *meta* position. In this case, Rh_2_(esp)_2_ gave a 1:1.5 ratio of **2b**/**3b** (entry 7). Extrapolating from the trends previously observed on **1a**, one might expect a large swing toward allylic amination using Rh_2_(**C**)_2_·(**4a**)_2_·(Pyr)_2_, and this was indeed borne out with a 23:1 ratio of **2b**:**3b** observed (entry 8). Here, **2b** was obtained in a slightly lower ee compared with **2a** using the same catalyst. We next sought to determine the effect of reducing the size of the quaternizing benzyl group. Rh_2_(**C**)_2_·(**4b**)_2_·(Pyr)_2_, where the triethylsilyl groups of **4a** are replaced with *tert*-butyl groups, gave a reduced ratio of 9:1 of **2b**:**3b** as well as lower ee (entry 9). Reducing further still to **4c**, which dispenses with the outer aryl rings but retains *tert*-butyl substituents at the *meta* positions of the benzylating group, gave further decreases in both chemoselectivity (6.5:1) and enantioselectivity, and cations **4d** and **4e**, which are closely related sterically, gave similar outcomes (entries 10–12). We sought to deconvolute potential contributions to chemoselectivity from the ligand sulfonate group and the chiral cation by evaluating Rh_2_(**C**)_2_·(**Bu**_**4**_ **N**)_2_ where the optimal sulfonated ligand scaffold is unchanged but is paired with a tetrabutylammonium cation instead of the cinchona alkaloid-derived cation. This provided a very clear answer as it gave a 1:1.2 ratio of **2b**:**3b**, almost identical to that of Rh_2_(esp)_2_ (entry 7 vs 13), indicating that the chiral cation is exclusively responsible for the chemoselectivity and enantioselectivity obtained using Rh_2_(**C**)_2_·(**4a**)_2_·(Pyr)_2_ on this substrate. At this stage, we evaluated **TcesNH**_**2**_ as an aminating agent, rather than the perfluorinated **PfpsNH**_**2**_, and were pleased to see that chemoselectivity was further improved (39:1) with only a slight ee decrease (73%, entry 14). Hopeful that this chemoselectivity improvement using **TcesNH**_**2**_ may translate back to the phenyl substrate **1a**, we were pleased to find that this was the case and now an 8:1 ratio of **2a**:**3a** could be obtained, compared with 3:1 previously (entry 15 vs 4). Furthermore, ee was improved from 88% to 93%. Retaining substrate **1a**, we again slightly reduced the size of the cation quaternizing group from **4a** to **4b** and again saw a small but significant decrease in both chemoselectivity (5:1) and enantioselectivity (86% ee, entry 16), fully consistent with prior observations on **1b**. Finally, evaluating the loading of the C_6_F_5_I­(OTFA)_2_ additive, it was found that 20 mol % gave a slightly higher yield without impacting selectivity (entry 17). In these reactions, we found that the yields were often moderate even though consumption of the starting material was usually high, and we speculate that some decomposition pathways may be in operation although we were unable to identify these. We also evaluated the reaction in the absence of the C_6_F_5_I­(OTFA)_2_ additive, which was detrimental to both metrics (entry 18). In previous work, we have consistently observed that the inclusion of this additive, which serves to release trifluoroacetic acid, has a beneficial impact on enantioselectivity and speculate that protonation of the basic nitrogen of the chiral cation may modify its conformation in a favorable manner.^13b^ For a comparison of several other hypervalent-iodine-based additives and a study on the loading of C_6_F_5_I­(OTFA)_2_, see the Supporting Information.

**Table 1:**
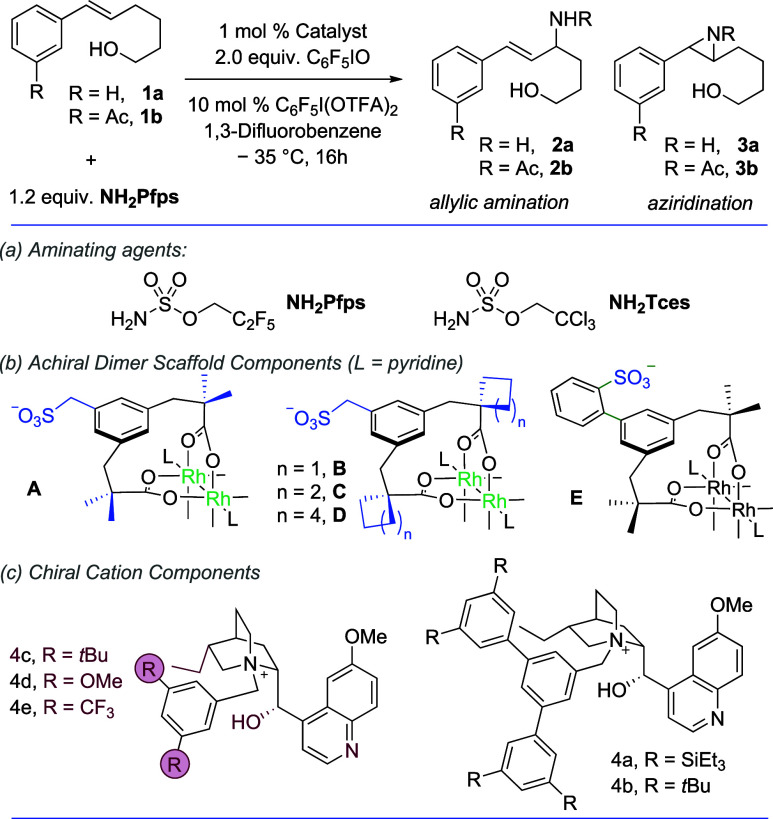
Optimization on Trishomoallylic Alcohol Substrates **1a** and **1b**

entry	substrate	catalyst	yield **2** ^a^/%	yield **3** ^a^/%	ratio **2**:**3**	ee **2**/%^b^
1	**1a**	Rh_2_(esp)_2_	5	48	1:9	
2	**1a**	Rh_2_(**A**)_2_·(**4a**)_2_·(Pyr)_2_	32	12	2.7:1	85
3	**1a**	Rh_2_(**B**)_2_·(**4a**)_2_·(Pyr)_2_	20	16	1.3:1	87
4	**1a**	Rh_2_(**C**)_2_·(**4a**)_2_·(Pyr)_2_	42	14	2.9:1	88
5	**1a**	Rh_2_(**D**)_2_·(**4a**)_2_·(Pyr)_2_	44	12	3.8:1	85
6	**1a**	Rh_2_(**E**)_2_·(**4a**)_2_·(Pyr)_2_	38	22	1.7:1	84
7	**1b**	Rh_2_(esp)_2_	17	27	1:1.5	
8	**1b**	Rh_2_(**C**)_2_·(**4a**)_2_·(Pyr)_2_	55	2	23:1	77
9	**1b**	Rh_2_(**C**)_2_·(**4b**)_2_·(Pyr)_2_	47	5	9:1	69
10	**1b**	Rh_2_(**C**)_2_·(**4c**)_2_·(Pyr)_2_	30	5	6.5:1	58
11	**1b**	Rh_2_(**C**)_2_·(**4d**)_2_·(Pyr)_2_	35	6	5.8:1	54
12	**1b**	Rh_2_(**C**)_2_·(**4e**)_2_·(Pyr)_2_	26	4	6.3:1	56
13	**1b**	Rh_2_(**C**)_2_·(**Bu**_**4**_ **N**)_2_	11	12	1:1.2	
14^c^	**1b**	Rh_2_(**C**)_2_·(**4a**)_2_·(Pyr)_2_	47	1	39:1	73
15^c^	**1a**	Rh_2_(**C**)_2_·(**4a**)_2_·(Pyr)_2_	36	4	8:1	93
16^c^	**1a**	Rh_2_(**C**)_2_·(**4b**)_2_·(Pyr)_2_	43	9	5:1	86
17^c^ ^,^ ^d^ ^,^ ^e^	**1a**	Rh_2_(**C**)_2_·(**4a**)_2_·(Pyr)_2_	49	5	9:1	93
18^c^ ^,^ ^e^	**1a**	Rh_2_(**C**)_2_·(**4a**)_2_·(Pyr)_2_	31	9	3:1	74

a Yield determined by NMR with internal standard.

b ee determined by chiral SFC analysis.

c Reaction using NH2Tces aminating agent.

d Using 20 mol % C_6_F_5_I­(OTFA)_2_.

e No C_6_F_5_I­(OTFA)_2_ additive used.

We evaluated further substrates under the optimized conditions using Rh_2_(**C**)_2_·(**4a**)_2_·(Pyr)_2_ as the catalyst and **TcesNH**_**2**_ as the aminating agent (Scheme 1). The conditions translated well to variations of **1a** bearing diverse arene substitution, and in all cases, allylic amination was the major outcome, in preference to aziridination. Yields were sometimes modest, but this is not attributable to poor selectivity but rather suspected decomposition pathways (vide supra). For *para* substitution, phenyl (**2c**), fluoro (**2d**), and *tert*-butyl (**2e**) could be accommodated, and in most cases, the enantioselectivity was very high (94–97% ee). For substituents at the *meta* position, acetyl-functionalized **1b**, explored during optimization, was a moderate ee example, while other substituents, acetoxy (**2f**), *tert*-butyl (**2g**), methoxy (**2h**), an ester (**2i**), and a Boc-protected amine (**2j**), were superior. At the *ortho* position, very high ee could be maintained with methoxy (**2k**), methyl (**2l**), chloro (**2m**), and bromo (**2n**). Ethyl-substituted **2o** was notable in that no amination was observed on the ethyl group and a highly hindered mesityl group was tolerated (**2p**). Geminal dimethyl substitution on the alcohol could be incorporated with only a small drop in ee (**2q**). We evaluated two substrates displaying extremes of electronics: trimethoxy-substituted **2r** and nitro-substituted **2s** but unfortunately no allylic amination product was obtained for either. We also evaluated a substrate containing an *N*-Ts indole, but this resulted in a complex mixture with no evidence of the desired allylic amination product formed. A challenge that we often encountered was obtaining racemic samples—for some substrates, even when 4 mol % Rh_2_(esp)_2_ was used, yields could be very low (<10%), leading to a nonselective mixture of aziridination and allylic amination or sometimes no allylic amination at all. For example, when Rh_2_(esp)_2_ was used in an attempt to access the racemate of **2o**, the only identifiable amination product resulted from insertion at the benzylic position of the ethyl substituent, highlighting the precise chemoselectivity and site selectivity imposed by our catalysts. We evaluated a nonstyrenyl trans alkene substrate, which gave the allylic amination product **2t** in a low yield of 18% but an encouraging ee of 75%. It is notable that in the crude NMR of this reaction, there was no evidence of an isomeric allylic amination product arising from amination at the allylic position distal to the alcohol group, neither was there evidence of aziridine. The low mass balance in the reaction again suggested unidentified decomposition pathways.

**Scheme 1:**
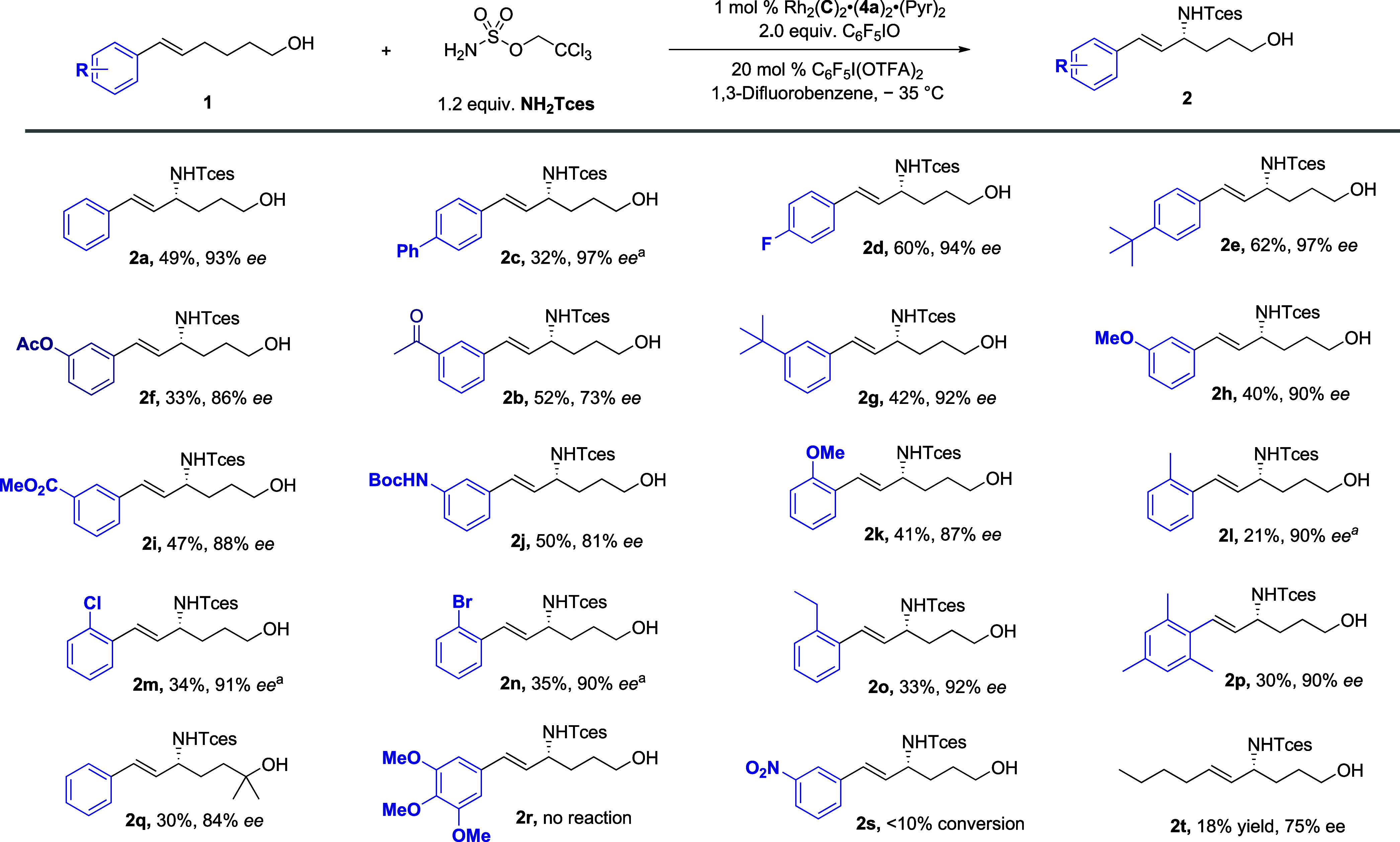
Scope of the Enantioselective Allylic Amination on Trishomoallylic Alcohols

a Reaction performed at −25 °C

We next questioned whether allylic amination may still be achievable with a reduced chain length between the alkene and the alcohol. Previously, *trans*-bishomoallylic alcohol **5a** had given rise to aziridination and then ring opening, albeit in moderate yield and low ee.^13b^ Our earlier optimization suggested that use of ****Tces**NH**_**2**_ as the aminating agent may bias selectivity toward allylic amination when used with our ion-paired catalysts; therefore, we evaluated **5a** under these modified conditions (Table 2). Use of Rh_2_(esp)_2_, even at high catalyst loading, gave primarily aziridination (1:3.5 **6a**:**7a**, entry 1). Use of Rh_2_(**C**)_2_·(**4a**)_2_·(Pyr)_2_, the optimal ion-paired catalyst for the longer chain substrates, gave an improved, but still moderate, ratio of 1.7:1 **6a**:**7a** but encouragingly, **6a** was obtained in 81% ee (entry 2). We have recently observed that the ion-paired catalyst based on biaryl scaffold **E** is effective for enantioselective nitrene transfer to shorter chain lengths for benzylic C–H amination and aziridination and hoped that this might favor allylic amination over aziridination in **5a**.^13c^ Gratifyingly, Rh_2_(**E**)_2_·(**4a**)_2_·(Pyr)_2_ delivered a 7:1 ratio of **6a**/**7a**, and furthermore, **6a** was obtained with excellent 93% ee, demonstrating that this catalyst is indeed an excellent match with the substrate (entry 3).

**Table 2:**
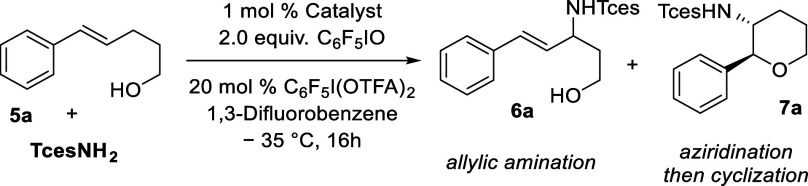
Catalyst Evaluation on Bishomoallylic Alcohol **5a**

entry	catalyst	yield **6a** ^a^/%	yield **7a** ^a^/%	ratio **6**:**7**	ee **6a** ^b^
1	Rh_2_(esp)_2_	11	39	1:3.5	
2	Rh_2_(**C**)_2_·(**4a**)_2_·(Pyr)_2_	32 (47)	19	1.7:1	81
3	Rh_2_(**E**)_2_·(**4a**)_2_·(Pyr)_2_	36 (48)	5	7:1	93

a Yield determined by NMR with internal standard, isolated yields in parentheses.

b ee determined by chiral SFC analysis.

We evaluated a selection of bishomoallylic alcohols (Scheme 2). An *ortho*-isopropyl group was very well tolerated (**6b**), and a methoxy group could be incorporated at a single *meta* position (**6c**) and *tert*-butyl groups at both (**6d**). *Ortho*-substituted ester **6e** was obtained in an acceptable yield and high ee using the original catalyst Rh_2_(**C**)_2_·(**4a**)_2_·(Pyr)_2_, demonstrating how arene substitution can have a strong influence in these systems. Finally, geminal dimethyl substitution on the alcohol could be incorporated (**6f**).

**Scheme 2:**
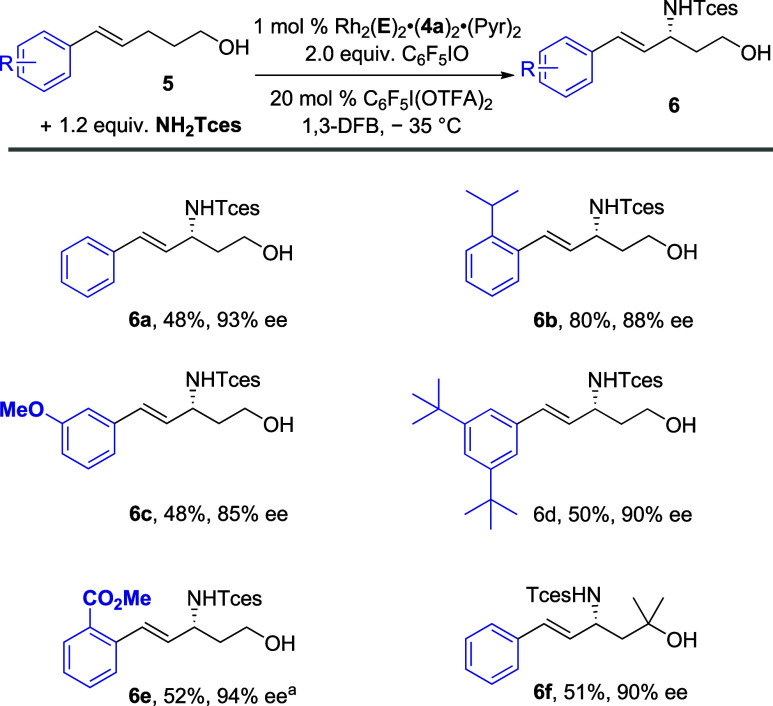
Scope of the Enantioselective Allylic Amination Bishomoallylic Alcohols

a Rh_2_(**C**)_2_·(**4a**)_2_·(Pyr)_2_ used as catalyst.

We next demonstrated the application of our allylic amine reaction products to the synthesis of an enantioenriched pyrrolidine (Scheme 3a, upper) and could determine the absolute stereochemistry of the allylic amine products by exchanging the Tces protecting group for Boc and comparing with the literature (Scheme 3a, lower).^15^ Additionally, we sought to gain more insight into the importance of the alcohol group by evaluating other functional groups in its place, examining an ester, methyl ether, and secondary and tertiary amides (Scheme 3b). Although an ester-containing substrate gave only traces of allylic amination (**9**), a better outcome was seen with a methyl ether, which gave a 28% NMR yield of **10** in a high 81% ee, with an accompanying 12% NMR yield of aziridine formed. However, the reaction profile was not as clean as with the alcohol directing group, and many other byproducts were visible in the crude reaction mixture, which rendered the purification very challenging, resulting in an isolated yield of only 6%. We have seen previously for benzylic C–H amination that in some cases, an ether seems to be an effective directing group, possibly acting as a hydrogen bond acceptor, which could account for the high ee observed here.^13d^ A secondary amide gave a similar NMR yield (28%) of allylic amination in **11** but the ee was lower at 44%. An analogous tertiary amide gave a complex outcome, which possibly included aziridination, cyclization, and allylic amination; these unfortunately could not be deconvoluted. These findings identify that the alcohol is the optimal directing group for allylic amination with these catalysts, in terms of both ee and yield of the allylic amine product. While the ether was also effective for high ee, the yield was lower, with multiple byproducts formed. It is possible that the substrate alcohol could act as both a hydrogen bond donor and acceptor in a complex network of interactions to provide the optimal outcome, while the corresponding methyl ether can function only as an acceptor, making the overall directing effect weaker.

**Scheme 3:**
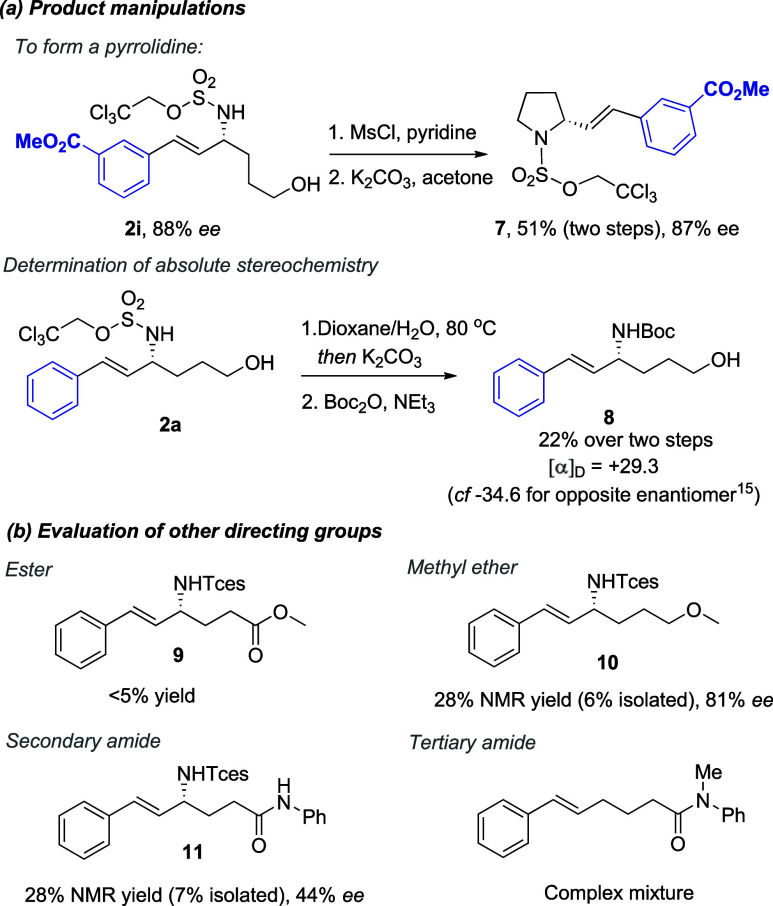
Product Manipulations (a) and Evaluation of Other Directing Groups (b)

To build on these insights, we sought to determine which structural features of the chiral cation were crucial. For previous C–H amination^13c^ and aziridination,^13c,16^ reactions we have carried out “knockout” studies relating to the Cinchona-derived cation, in which a series of ion-paired Rh catalysts where natural features of the cation are removed or modified are evaluated in a systematic manner. The series of directly comparable complexes available to use in this study diverged slightly from the optimal catalyst identified for allylic amination, in that they possessed gem-dimethyl substituents on the Rh complex (Table 1, **A**) as well as the benzylating group of cation **4b** featuring peripheral *tert*-butyl groups, rather than triethylsilyl groups (as in the optimal cation **4a**). Based on the earlier optimization trends, it was expected that this base complex Rh_2_(**A**)_2_·(**4b**)_2_·(Pyr)_2_ would give a slightly lower allylic amination:aziridination ratio and ee of the allylic product. This was the case, with a 3.3:1 crude ratio, and **2a** was formed in 23% yield with 82% ee. This can be directly compared with Table 1, entry 15, in which the optimal catalyst Rh_2_(**C**)_2_·(**4a**)_2_·(Pyr)_2_ gives 36% yield and 93% ee, with an 8:1 ratio. Nevertheless, we envisaged that the “knockout” study, even with a slightly suboptimal complex, should still provide valuable insights, and the results are shown in Figure 2. First, effective removal of the quinoline nitrogen and methoxy group by replacing the 5-methoxyquinoline of the alkaloid with a 1-naphthyl group (cation **4h**) was extremely detrimental to reactivity, giving <5% yield of **2a**. Return of the quinoline nitrogen in cation **4g** improved reactivity although in favor of aziridination over amination, with a moderate 59% ee obtained for **2a**. Surprisingly, returning the quinoline methoxy group in cation **4f** improved the outcome significantly, with 81% ee of **2a** and a 5.6:1 crude ratio, now favoring allylic amination. Addition of an *n*-butyl group at the quinoline 2-position in cation **4i** affected neither of the metrics, suggesting that the favorable impact of the quinoline nitrogen is not related to binding to the Rh metal center (on which quinoline 2-substitution would be expected to impact). Methylation of the alcohol of the alkaloid (cation **4j**) was highly detrimental to all aspects, as was inversion of the alcohol stereochemistry (cation **4k**). For both of these complexes in which the catalyst hydroxyl group was modified, aziridination took over as the major outcome, and the ee of the small amount of **2a** that was formed was poor. These findings were particularly interesting because in previous “knockout” evaluations, we only had enantioselectivity as a selectivity metric, but here, we can also observe the impact on chemoselectivity, adding an extra and intriguing dimension. These results clearly show that catalyst features which favor the formation of the allylic amination product also significantly boost its enantioselectivity. This outcome is consistent with the catalyst being directed to the allylic position, and when the directing interaction is impaired, the catalyst functions far less effectively, leading to lower ee. Acceleration of the formation of one product in a selective manner is a hallmark of noncovalent catalysis, and this is in full agreement with our hypothesis, in which the alcohol of the substrate is the optimal directing group in this transformation.^17^

**Figure 2:**
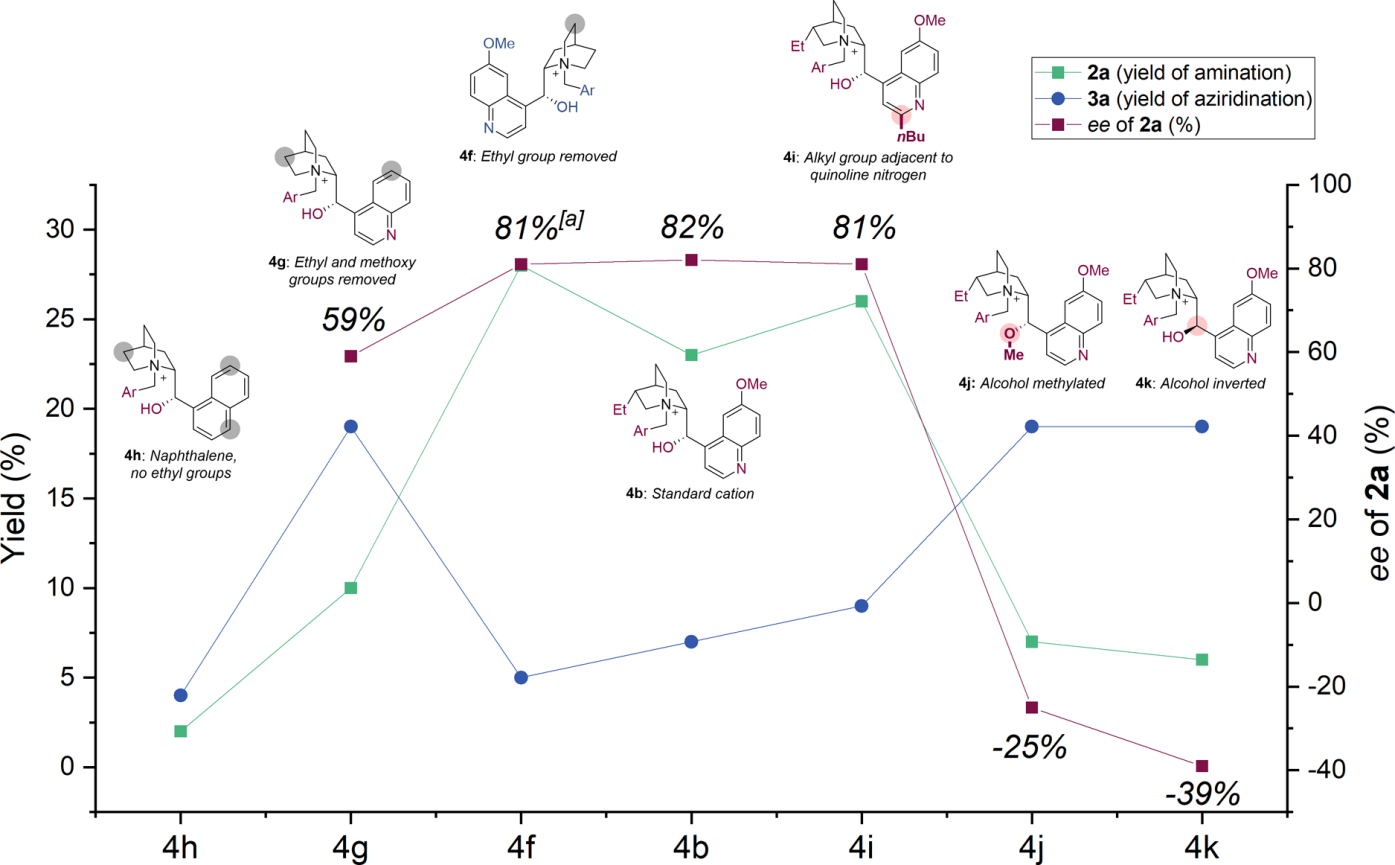
Investigation of the impact of the cation structure on enantioselectivity and chemoselectivity. ^a^Antipode of **2a** obtained due to cation **4f** having the opposite aminoalcohol configuration compared with the others.

## 3 Conclusions

In this study, we have demonstrated that ion-paired Rh catalysts can accomplish highly enantioselective and chemoselective allylic amination of *trans*-configured trishomoallylic and bishomoallylic alcohols. In contrast to Rh_2_(esp)_2_, which typically delivers either mixtures or predominantly aziridination on these substrates, our catalysts exert very high levels of control, and this can be attributed to the unique chiral pocket provided by the associated chiral cations. Chemo- and enantioselective allylic amination is still highly challenging to perform in an intermolecular manner, and we envisage that our system will be applicable to further substrate types in due course.

## 4 Experimental Section

### 4.1 General Procedure for the Enantioselective Rh-Catalyzed Allylic Amination Reaction

To a 4.0 mL crimp-top vial was added substrate (0.1 mmol, 1.0 equiv), the required **[Rh]** catalyst (1.0 mol %), TcesNH_2_ (27.1 mg, 0.12 mmol, 1.2 equiv), and 1,3-DFB (0.5 mL). The vial was cooled to −35 °C over 15 min. Following this, PFIOB (62.1 mg, 0.2 mmol, 2.0 equiv) and C_6_F_5_I­(OTFA)_2_ (10.4 mg, 20 mol %) were added to the vial at −35 °C in one portion. The vial was sealed and stirred at −35 °C overnight. Following this, thiourea (saturated solution, 1.0 mL) was added to quench the reaction, followed by CHCl_3_ (1.0 mL). The layers were separated, and the aqueous layer was extracted further with CHCl_3_ (3 × 1.0 mL). The combined organic layers were dried with MgSO_4_, filtered, and concentrated in vacuo. Purification by FCC (SiO_2_, 0–30% v/v acetone in CHCl_3_) afforded the allylic amination product. For full details, see the Supporting Information.

## Supplementary Material



## Notes

Additional optimization, full experimental details, characterization data for compounds (NMR spectra, SFC traces), evaluation of 6-C *cis* alkenyl alcohol substrate under optimized conditions, summary of yields and ee values for 2a using various catalyst loadings, summary of yields and ee values for 2a using various additive loadings, summary of yields and ee values for 2a using various additives, and summary of yields and ee values for 2a using various modified chiral cations (PDF)

## References

[1] Johannsen M., Jørgensen K. A. (1998). Allylic Amination. Chem. Rev..

[2] Ramirez T. A., Zhao B., Shi Y. (2012). Recent advances in
transition metal-catalyzed
sp3 C–H amination adjacent to double bonds and carbonyl groups. Chem. Soc. Rev..

[3] Reed S. A., White M. C. (2008). Catalytic Intermolecular
Linear Allylic
C–H Amination via Heterobimetallic Catalysis. J. Am. Chem. Soc..

[4] Collet F., Lescot C., Dauban P. (2011). Catalytic
C–H
amination: the stereoselectivity issue. Chem.
Soc. Rev..

[5] Bruncko M., Khuong T.-A. V., Sharpless K. B. (1996). Allylic
Amination and 1,2-Diamination with a Modified Diimidoselenium Reagent. Angew. Chem., Int. Ed..

[6] Cheng Q., Chen J., Lin S., Ritter T. (2020). Allylic Amination
of Alkenes with Iminothianthrenes to Afford Alkyl Allylamines. J. Am. Chem. Soc..

[7] Li H.-H., Chen X., Kramer S. (2023). Recent developments
for intermolecular enantioselective amination of non-acidic C­(sp3)–H
bonds. Chem. Sci..

[8] Liu W., Choi I., Zerull E. E., Schomaker J. M. (2022). Tunable
Silver-Catalyzed Nitrene Transfer: From Chemoselectivity to Enantioselective
C–H Amination. ACS Catal..

[9] Milczek E., Boudet N., Blakey S. (2008). Enantioselective
C-H
Amination Using Cationic Ruthenium­(II)–pybox Catalysts. Angew. Chem., Int. Ed..

[10] Liang C., Collet F., Robert-Peillard F., Müller P., Dodd R. H., Dauban P. (2008). Toward a Synthetically
Useful Stereoselective C–H Amination of Hydrocarbons. J. Am. Chem. Soc..

[11] Nishioka Y., Uchida T., Katsuki T. (2013). Enantio- and
Regioselective Intermolecular
Benzylic and Allylic C-H Bond Amination. Angew.
Chem., Int. Ed..

[12] Dolan N. S., Scamp R. J., Yang T., Berry J. F., Schomaker J. M. (2016). Catalyst-Controlled
and Tunable, Chemoselective Silver-Catalyzed Intermolecular Nitrene
Transfer: Experimental and Computational Studies. J. Am. Chem. Soc..

[13] Fanourakis A., Williams B. D., Paterson K. J., Phipps R. J. (2021). Enantioselective Intermolecular C–H
Amination
Directed by a Chiral Cation. J. Am. Chem. Soc..

[14] Ermanis K., Gibson D. C., Genov G. R., Phipps R. J. (2023). Interrogating the
Crucial Interactions at Play in the Chiral Cation-Directed Enantioselective
Borylation of Arenes. ACS Catal..

[15] Wu Y., Hu L., Li Z., Deng L. (2015). Catalytic asymmetric
umpolung reactions
of imines. Nature.

[16] Lit A. R., Takano S., Zachau C., Băltăreţu I., Phipps R. J. (2025). Asymmetric Aziridination of Allylic Carbamates Using
Ion-Paired Rhodium Complexes and Extrapolation to C–H Amination
of Phenethyl Carbamates. Angew. Chem., Int.
Ed..

[17] Sak M. H., Jacobsen E. N. (2025). Selective Noncovalent Catalysis with
Small Molecules. Chem. Rev..

