# Supplementary material for: Allylic Amination of Alkenyl Alcohols: Simultaneous Control of Chemoselectivity and Enantioselectivity in Nitrene Transfer Using Ion-Paired Catalysts
Source: ACS Catal. 2025 Aug 8;15(17):14639–46. doi: 10.1021/acscatal.5c04140 (PMC12418313; doi:10.1021/acscatal.5c04140)
Supplement: Supplementary file 1 [file cs5c04140_si_001.pdf]

Allylic Amination of Alkenyl Alcohols: Simultaneous Control of Chemoselectivity and Enantioselectivity in Nitrene Transfer Using Ion-Paired Catalysts

Hannah K. Adams, Alexander Fanourakis, Amit Dahiya, Ioana Băltărețu and Robert J. Phipps*

Yusuf Hamied Department of Chemistry, University of Cambridge, Lensfield Road, Cambridge, CB2 1EW, United Kingdom.

*Corresponding Author Email: rjp71@cam.ac.uk

Table of Contents

1. General Information ___ ***3***

2. Synthesis of Starting Materials ___ ***5***

2.1 General Procedures ___ **5**

2.2 Trans alkenyl alcohol substrate synthesis (6-C Chain length) ___ **7**

2.3 Trans alkenyl alcohol substrate synthesis (5-C Chain length) ___ **23**

3. Synthesis of Chiral Rh-Catalysts ___ ***27***

4. Sulfamate ester, Additive and Oxidant Synthesis ___ ***32***

5. Enantioselective Intermolecular Allylic Amination Products ___ ***33***

5.1 Substrate scope (6-C Chain length) ___ **34**

5.2 Substrate scope (5-C Chain length) ___ **47**

6. Catalyst and Additive Optimisation ___ ***51***

6.1 For 6-C Trans alkenyl alcohol substrate ___ **51**

6.2 Evaluation of 6-C Cis alkenyl alcohol substrate ___ **55**

7. Post Functionalisation of Allylic Amines ___ ***58***

8. Determination of Absolute Stereochemistry ___ ***59***

9. "Knock-out" studies ___ ***60***

10. Chiral SFC Traces ___ ***61***

11. NMR Spectra ___ ***90***

12. References ___ ***231***

1. General Information

Reaction Setup, Solvents and Reagents: All reactions were carried out under an inert argon or nitrogen atmosphere using standard Schlenk-septa techniques in heat gun-dried glassware unless otherwise stated. Reactions performed in 4.0 mL crimp-top vials that required cooling were placed in a Polar Bear Cub (by Cambridge Reactor Design) featuring a deep-welled heating block (IKA DB 5.2). All reagents were used as supplied from commercial sources without further purification unless otherwise stated. NaOH was finely ground. Tetrahydrofuran, Et_2O, MeOH, MeCN, methylene chloride, and *n*-hexane were purified by distillation on site under inert atmosphere *via* the following processes: tetrahydrofuran and Et_2O were pre-dried over sodium wire then distilled from calcium hydride and lithium aluminium hydride. MeOH, MeCN, CH_2Cl_2, and *n*-hexane were distilled from calcium hydride.

Chiral SFC Analysis: Performed on a Waters ACQUITY UPC2 System with a DAICEL CHIRALPAK IA, IC, IE, IG, IJ, or IK columns (4.6 x 250 mm, 3.0 μm) in a mixed solvent system of supercritical CO_2 and MeOH. A system backpressure of 138 bar was used in all cases.

Chiral HPLC Analysis: Performed on a Waters ARC system with CHIRALPAK YMC IG or IH columns (4.6 x 250 mm, 3 μm) in a mixed solvent system of *n*-hexane and *i*PrOH.

Chromatography: Analytical thin-layer chromatography was performed using precoated Merck glass backed silica gel plates (Silica gel 60 F254). Visualisation was by ultraviolet fluorescence (λ = 254 and 365 nm) and/or staining with potassium permanganate ($KMnO_4$) or Ceric Ammonium Molybdate (CAM). Flash column chromatography was performed using silica gel 60 (pore size: 60 Å, mesh: 40-63 μm) from Material Harvest®. All ratios of eluents are quoted as v/v.

High Resolution Mass Spectrometry (HRMS): Recorded on a Waters Micromass LCT Premier, a Waters Xevo G2-S or a Waters Vion IMS QTOF at the Department of Chemistry at the University of Cambridge. The ionisation method is noted – positive/negative electrospray ionisation (+/– ESI) or positive atmospheric solids analysis probe (ASAP+). Measured values are reported to 4 decimal places and are within ± 5 ppm of the calculated value. The calculated values are based on the most abundant isotope unless otherwise stated in the chemical formula. For ions bearing more than a single unit of charge, the masses reported as 'found' and 'required' are the mass/charge ratios.

NMR Spectroscopy: ^{1}H NMR spectra were recorded on 700 MHz TXO Cryoprobe, 600 MHz Bruker Avance DRX-600, 500 MHz Bruker DCH Cryoprobe, 400 MHz Bruker DPX-400 Dual, 400 MHz Avance III HD or 400 MHz Avance III HD Smart Probe spectrometers. Chemical shifts are reported in parts per million (ppm) and the spectra are calibrated to the resonance resulting from incomplete deuteration of the solvent ($CDCl_3$: 7.26 ppm; $(CD_3)_2CO$: 2.05 ppm, p; D_2O: 4.79 ppm; $(CD_3)_2SO$: 2.50 ppm, p; $C\mathit{D}_3OD$: 3.31 ppm, p; C_5D_5N (H–C–N): 8.74 ppm).[1] ^{13}C NMR spectra were recorded on the same spectrometers with complete proton decoupling. ^{13}C NMR experiments referenced as such were performed using a UDEFT sequence to increase the signal:noise ratio for ^{13}C signals of carbon nuclei along poly-fluorinated chains.[2] Chemical shifts are reported in ppm with the solvent resonance as the internal standard ($^{13}CDCl_3$: 77.16 ppm, t; $(^{13}\mathit{C}D_3)_2CO$: 29.84 ppm, sept; $(^{13}CD_3)_2SO$: 39.52 ppm, sept; $^{13}CD_3OD$: 49.00 ppm, sept; $^{13}C_5D_5N$ (C–N): 150.35 ppm, t).[1] ^{19}F NMR spectra were recorded on 400 MHz Avance III HD or 400 MHz Avance III HD Smart Probe spectrometers. Chemical shifts are reported in ppm with $CFCl_3$ as the external standard ($CFCl_3$: 0.00 ppm). Data are reported as follows: chemical shift δ, multiplicity (s = singlet, d = doublet, t = triplet, q = quartet, p = pentet, br = broad, m = multiplet or combinations thereof (^{13}C and all other nuclides except ^{1}H are singlets unless otherwise stated)), coupling constants J, number of nuclides (signals for all other nuclides except ^{1}H refer to one nuclide unless otherwise stated), assignment. ^{1}H NMR spectra are assigned as fully as possible, using ^{1}H-COSY, ^{1}H-NOESY, DEPT-135, HSQC and HMBC where appropriate to facilitate structural determination. Assignments either follow the numbering system shown on the structures or are described unambiguously. ^{1}H NMR signals are reported in ppm to 2 decimal places and all other nuclide signals to 1 decimal place. Coupling constants are reported in Hz to a maximum of 3 significant figures. For cinchona alkaloid-derived compounds the appearance and chemical shifts of the peaks in the NMR spectra can vary significantly depending on sample concentration and other factors. For spectra acquired in $CDCl_3$ the residual water peak is often visible at approximately 1.6 ppm in the ^{1}H NMR spectrum. For spectra acquired in C_5D_5N the residual water peak is often visible at approximately 4.9 ppm in the ^{1}H NMR spectrum.

Optical Rotations: Measured in spectrophotometric grade $CHCl_3$ on a Perkin Elmer 343 Polarimeter using a sodium lamp (λ = 589 nm, D-line). $[\alpha]_D$ values are reported at the stated temperature, with concentration in g /100mL.

Naming and Numbering of compounds: Systematic names were generated by the computer program ChemDraw according to the guidelines specified by the IUPAC. However, the numbering on the structures does not correspond to the systematic name.

2. Synthesis of Starting Materials

2.1 General Procedures

General Procedure 1 (GP1) [Formation of silyl alkynyl alcohol]

To an RBF containing alcohol alkyne (1.0 eq.) and TBSCl (1.2 eq.) in methylene chloride (1.0 M) was added TEA (1.2 eq.) dropwise at 0 °C. After 5 min, the ice bath was removed, and the reaction mixture was left to stir at rt overnight. Following this, the reaction was quenched with water (100 mL) and the organic layer separated. The aqueous layer was further extracted with methylene chloride (2 × 100 mL). The combined organic layers were washed with brine, dried with $MgSO_4$, filtered and concentrated *in vacuo*. The crude product was purified with FCC (SiO_2, 0-5% v/v EtOAc in petroleum ether) to give the title compound as a colourless oil.

General Procedure 2 (GP2) [Formation of trans vinyl boronate silyl-protected alcohols]

Alkynyl silyl alcohol (1.0 eq.) and 4,4,5,5-tetramethyl-[1,3,2]-dioxaborolane (1.2 eq.) were added to bis(cyclopentadienyl)zirconium chloride hydride (0.1 eq.) and TEA (0.1 eq.) under a N_2 atmosphere. The resulting mixture was heated at 60 °C overnight. The crude was diluted with hexanes and any precipitate removed by filtering over a short pad of silica gel and subsequently washed with hexanes. The filtrate was concentrated and dried under reduced pressure to give the title compound without further purification.

***NOTE**: The vinyl boronates are prone to degradation under ambient temperatures. To ensure the compound doesn't degrade, it is recommended to store the vinyl boronates in a freezer at -20 °C.*

General Procedure 3 (GP3) [Formation trans alkenyl alcohol substrates]

To an oven-dried microwave value was added the corresponding vinyl boronate (1.0 eq.), aryl halide (1.2 eq.), $Pd(dppf)Cl_2$ (5.0 mol%) and Cs_2CO_3 (3.0 eq.). The vial was subjected to three N_2 and vacuum cycles. THF:water (10:1, 0.5 M) was added and the vial was degassed with N_2 for 2 mins. The vial was heated to reflux overnight. Upon completion of the reaction, water (10 mL) and petroleum ether (10 mL) were added to the vial. The layers were separated, and the organic layer removed. The aqueous layer was further extracted twice with petroleum ether (2 × 10 mL). The combined organic layers were washed with brine, dried with $MgSO_4$, filtered

and concentrated *in vacuo* to give the crude product which was used without further purification in the next step.

The crude was dissolved in THF (0.5 M) and cooled to 0 °C. TBAF (1.0 M in THF, 1.5 eq.) was added dropwise to the reaction mixture. The reaction mixture was warmed to rt and stirred for 6 h. Upon completion, water (10 mL) and methylene chloride were added to the reaction mixture. The layers were separated, and the organic layer collected. The aqueous layer was further extracted twice with methylene chloride (2 × 10 mL). The combined organic layers were washed with brine, dried with $MgSO_4$, filtered and concentrated. The crude product was purified by FCC (SiO_2, 0-10% v/v acetone in $CHCl_3$) to give the title compound.

2.2 Trans alkenyl alcohol substrate synthesis (6-C Chain length)

tert-butyl(hex-5-yn-1-yloxy)dimethylsilane

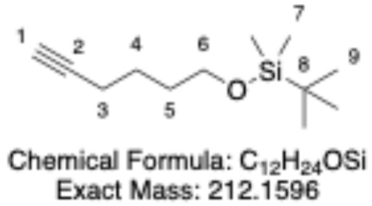

According to **GP1**, 5-hexyn-1-ol (10.0 g, 102 mmol, 1.0 eq.) and TBSCl (18.1 g, 120 mmol, 1.2 eq.) were dissolved in methylene chloride (150 mL) and TEA (15.5 mL, 120 mmol, 1.2 eq.) was added to the flask. FCC of the crude mixture revealed the title compound as a colourless oil (21.1 g, 99.4 mmol, 97%). ^{1}**H NMR** (700 MHz, $CDCl_3$): δ (ppm) 3.62 (t, *J* = 6.1 Hz, 2H, H-6), 2.21 (td, *J* = 7.0, 2.6 Hz, 2H, H-3), 1.93 (t, *J* = 2.7 Hz, 1H, H-1), 1.66 - 1.55 (m, 4H, H-4, H-5), 0.88 (s, 9H, H-9), 0.04 (s, 6H, H-7). ^{13}**C NMR** (176 MHz, $CDCl_3$): δ (ppm) 84.5, 68.3, 62.6, 31.8, 25.9, 25.0, 18.3, 18.2, -5.3. The spectroscopic data matches with that reported in the literature.[3]

(E)-tert-butyldimethyl((6-(4,4,5,5-tetramethyl-1,3,2-dioxaborolan-2-yl)hex-5-en-1-yl)oxy)silane

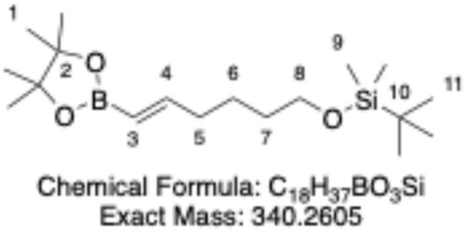

Prepared according to **GP2**, on a 14.3 mmol scale with respect to the silyl protected alcohol. The title compound was obtained as a colourless oil (1.00 g, 3.53 mmol, 25%). ^{1}**H NMR** (400 MHz, $CDCl_3$): δ (ppm) 6.65 (d, *J* = 17.6 Hz, 1H, H-3), 5.47-5.39 (m, 1H, H-4), 3.60 (t, *J* = 5.7 Hz, 2H, H-8), 2.21-2.13 (m, 2H, H-7), 1.55-1.41 (m, 4H, H-5, H-6), 1.27 (s, 12H, H-1), 0.89 (s, 9H, H-11), 0.04 (s, 6H, H-9). ^{13}**C NMR** (176 MHz, $CDCl_3$): δ (ppm) 154.5, 114.8, 112.3, 83.0, 63.0, 35.6, 32.3, 26.0, 24.8, 18.4, -5.3. The spectroscopic data matches with that reported in the literature.[4]

(E)-6-phenylhex-5-en-1-ol (1a)

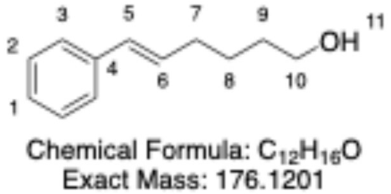

Prepared according to **GP3**, on a 2.0 mmol scale with respect to the silyl protected alcohol vinyl boronate and the corresponding aryl iodide (1.1 eq.). The title compound was obtained as a colourless oil (194 mg, 1.10 mmol, 55%). **^{1}H NMR** (700 MHz, $CDCl_3$): δ (ppm) 7.36 - 7.33 (m, 2H, H-3), 7.31 - 7.28 (m, 2H, H-2), 7.21 - 7.18 (m, 1H, H-1), 6.40 (d, J = 15.8 Hz, 1H, H-5), 6.23 (td, J = 15.8 Hz, 6.9 Hz, 1H, H-6), 3.68 (t, J = 6.5 Hz, 2H, H-10), 2.27-2.24 (m, 2H, H-7), 1.66 - 1.61 (m, 2H, H-9), 1.59 - 1.54 (m, 2H, H-8), 1.39 (br. s, 1H, H-11). **^{13}C NMR** (176 MHz, $CDCl_3$): δ (ppm) 137.8, 130.6, 130.2, 128.5, 126.9, 125.9, 62.8, 32.7, 32.3, 25.5. The spectroscopic data matches with that reported in the literature.[5]

(E)-(6-methoxyhex-1-en-1-yl)benzene

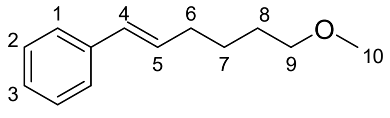

To a solution of ***(E)-6-phenylhex-5-en-1-ol*** (204 mg, 1.16 mmol, 1 eq.) in THF (11 mL) at 0 °C, NaH (60% mineral oil, 92 mg, 2.0 eq.) was added and the mixture was left to stir for15 min, followed by the dropwise addition of methyl iodide (0.7 mL, 10 eq.). The reaction mixture was stirred at room temperature for a further 16 hours, then quenched with water and extracted with ethyl acetate (x3). The combined organic phases were dried over magnesium sulfate and concentrated *in vacuo*. Purification by flash column chromatography (SiO_2, 0-5% v/v ethyl acetate in hexanes) afforded the title compound as a colourless oil (147 mg, 0.77 mmol, 67%). **^{1}H NMR** (400 MHz, $CDCl_3$): δ 7.38 – 7.26 (m, 4H, H-1, H-2), 7.24 – 7.16 (m, 1H, H-3), 6.40 (d, J = 15.6 Hz, 1H, H-4), 6.31 – 6.16 (m, 1H, H-5), 3.48 – 3.30 (m, 5H, H-9, H-10), 2.34 – 2.17 (m, 2H, H-6), 1.71 – 1.48 (m, 4H, H-7, H-8). **^{13}C NMR** (176 MHz, $CDCl_3$): δ 138.0, 130.8, 130.2, 128.6, 127.0, 126.1, 77.3, 77.2, 77.0, 72.8, 58.7, 32.9, 29.3, 26.0. **HRMS** (ESI) m/z Calc'd for $C_{13}H_{19}O+$ [M+H]+: 191.1430, Found: 191.1438 (δ = + 4.2 ppm).

(E)-1-(3-(6-hydroxyhex-1-en-1-yl)phenyl)ethan-1-one (1b)

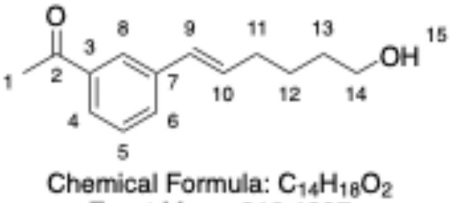

Prepared according to **GP3**, on a 1.5 mmol scale with respect to the silyl protected alcohol vinyl boronate and the corresponding aryl iodide (1.1 eq.). The title compound was obtained as a colourless oil (183 mg, 0.84 mmol, 56%). **^{1}H NMR** (700 MHz, $CDCl_3$): δ (ppm) 7.92 (s, 1H, H-8), 7.78 (d, *J* = 7.7 Hz, 1H, H-4), 7.53 (d, *J* = 7.7 Hz, 1H, H-6), 7.39 (t, *J* = 7.7 Hz, 1H, H-5), 6.44 (d, *J* = 15.8 Hz, 1H, H-9), 6.31 (td, *J* = 15.8, 6.8 Hz, 1H, H-10), 3.69 (app. q, *J* = 5.3 Hz, 1H, H-14), 2.61 (s, 3H, H-1), 2.28 (app. q, *J* = 7.1 Hz, H-11), 1.67-1.62 (m, 2H, H-13), 1.61-1.56 (m, 2H, H-12), 1.26-1.24 (m, 1H, H-15). **^{13}C NMR** (176 MHz, $CDCl_3$): δ (ppm) 198.3, 138.4, 137.4, 132.1, 130.4, 129.3, 128.7, 126.8, 125.7, 62.8, 37.7, 32.2, 26.7, 25.4. **HRMS** (ESI) *m/z* Calc'd for $C_{14}H_{19}O_2^+$ $[M+H]^+$: 219.1380, Found: 219.1374 (δ = - 2.7 ppm).

(E)-6-([1,1'-biphenyl]-4-yl)hex-5-en-1-ol (1c)

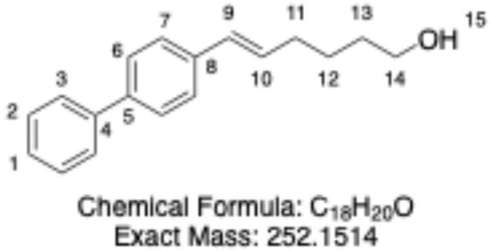

Prepared according to **GP3**, on a 1.2 mmol scale with respect to the silyl protected alcohol vinyl boronate and corresponding aryl bromide (1.1 eq.). The title compound was obtained as a pale-white solid (69.3 mg, 0.28 mmol, 23%). **^{1}H NMR** (400 MHz, $CDCl_3$): δ (ppm) 7.62-7.58 (m, 2H, H-3), 7.56-7.53 (m, 2H, H-6), 7.47-7.40 (m, 4H, H-2, H-7), 7.37-7.31 (m, 1H, H-1), 6.45 (d, *J* = 15.8 Hz, 1H, H-9), 6.26 (dt, *J* = 15.8, 6.8 Hz, 1H, H-10), 3.69 (t, *J* = 6.3 Hz, 2H, H-14), 2.32-2.25 (m, 2H, H-11), 1.71-1.53 (m, 4H, H-12, H-13), 1.35 (br. s, 1H, H-15). **^{13}C NMR** (101 MHz, $CDCl_3$): δ (ppm) 140.9, 139.7, 136.8, 130.8, 129.7, 128.8, 127.2, 127.1, 126.9, 126.3, 62.9, 32.8, 32.3, 25.1. **HRMS** (ESI) *m/z* Calc'd for $C_{18}H_{19}O^-$ $[M-H]^-$: 251.1436, Found: 251.1433 (δ = - 1.2 ppm).

(E)-6-(4-fluorophenyl)hex-5-en-1-ol (1d)

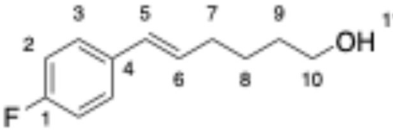

Chemical Formula: $C_{12}H_{15}FO$
Exact Mass: 194.1107

Prepared according to **GP3**, on a 0.60 mmol scale with respect to the silyl protected alcohol vinyl boronate and the corresponding aryl iodide (1.1 eq.). The title compound was obtained as a yellow oil (82.1 mg, 0.42 mmol, 73%). **^{1}H NMR** (400 MHz, $CDCl_3$): δ (ppm) 7.30-7.26 (m, 2H, H-2), 7.01-6.94 (m, 2H, H-3), 6.35 (d, J = 15.8 Hz, 1H, H-5), 6.12 (dt, J = 15.6, 7.0 Hz, 1H, H-6), 3.67 (app. q, J = 6.0 Hz, 2H, H-10), 2.23 (app. q, J = 7.0 Hz, 2H, H-7), 1.68-1.48 (m, 5H, H-8, H-9, H-11). **^{13}C NMR** (176 MHz, $CDCl_3$): δ (ppm) 166.7 (d, $J_{C\text{-}F}$ = 256.8 Hz), 133.9 (d, $J_{C\text{-}F}$ = 3.3 Hz), 133.4 (d, $J_{C\text{-}F}$ = 3.0 Hz), 130.3 (d, $J_{C\text{-}F}$ = 2.2 Hz), 127.3 (d, $J_{C\text{-}F}$ = 7.8 Hz), 115.4 (t, $J_{C\text{-}F}$ = 14.0 Hz), 62.6, 32.6, 32.3, 25.5. **^{19}F NMR** (376 MHz, $CDCl_3$): δ (ppm) -116.4. The spectroscopic data matches with that reported in the literature.[6]

(E)-6-(4-(tert-butyl)phenyl)hex-5-en-1-ol (1e)

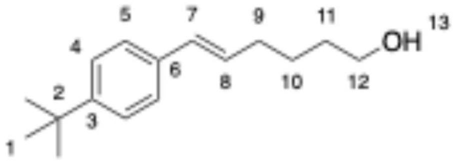

Chemical Formula: $C_{16}H_{24}O$
Exact Mass: 232.1827

Prepared according to **GP3**, on a 0.6 mmol scale with respect to the silyl protected alcohol vinyl boronate and the corresponding aryl bromide (1.1 eq.). The title compound was obtained as a colourless oil (54.6 mg, 0.24 mmol, 39%). **^{1}H NMR** (700 MHz, $CDCl_3$): δ (ppm) 7.33-7.31 (m, 2H, H-5), 7.29-7.26 (m, 2H, H-4), 6.37 (d, J = 15.8 Hz, 1H, H-7), 6.18 (td, J = 15.8, 6.9 Hz, 1H, H-8), 3.67 (app. q, J = 6.0 Hz, 2H, H-12), 2.25 (app. dq, J = 7.3 Hz, 1.4 Hz, 1H, H-9), 1.66-1.60 (m, 2H, H-11), 1.57-1.52 (m, 2H, H-10), 1.31 (s, 9H, H-1), 1.22 (t, J = 5.3 Hz, 1H, H-13). **^{13}C NMR** (176 MHz, $CDCl_3$): δ (ppm) 149.9, 135.0, 129.9, 129.8, 125.6, 125.4, 62.9, 34.5, 32.7, 32.3, 31.3, 25.6. **HRMS** (ESI) *m/z* Calc'd for $C_{16}H_{23}O^{-}$ [M-H]$^{-}$: 231.1749, Found: 231.1751 (δ = + 0.9 ppm).

3-iodophenyl acetate

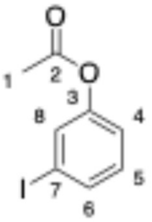

Ac_2O (0.51 g, 5.0 mmol, 1.0 eq.) and 3-iodophenol (1.1 g, 5.0 mmol, 1.0 eq.) were dissolved in DCM (20 mL). TEA (1.29 mL, 10 mmol, 2.0 eq.) was added dropwise and the reaction was stirred overnight at rt. After 16 h, the reaction was quenched with water. The organic layer was separated, dried with $MgSO_4$, filtered and concentrated *in vacuo*. The crude product was purified by FCC (SiO_2, DCM) to give the title compound as a colourless oil (1.31 g, 5.0 mmol, quant.). **^{1}H NMR** (700 MHz, $CDCl_3$): δ (ppm) 7.57 (d, J = 7.6 Hz, 1H, H-4), 7.48 (s, 1H, H-8), 7.12-7.07 (m, 2H, H-5, H-6), 2.30 (s, 3H, H-1). **^{13}C NMR** (176 MHz, $CDCl_3$): δ (ppm) 169.0, 150.9, 135.0, 130.8, 130.7, 121.2, 93.6, 21.0. The spectroscopic data matches with that reported in the literature.[7]

(E)-3-(6-hydroxyhex-1-en-1-yl)phenyl acetate (1f)

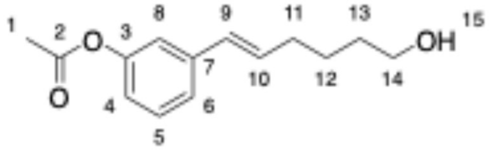

Prepared according to **GP3**, on a 1.2 mmol scale with respect to the silyl protected alcohol vinyl boronate **283** and corresponding aryl iodide **S368** (1.1 eq.). The title compound was obtained as a pale-brown solid (60.0 mg, 0.26 mmol, 21%). **^{1}H NMR** (700 MHz, $CDCl_3$): δ (ppm) 7.40 (d, J = 7.7 Hz, 1H, H-6), 7.26-7.25 (m, 1H, H-5), 6.96 (br. s, 1H, H-8), 6.83 (d, J = 7.8 Hz, 1H, H-4), 6.29 (d, J = 15.8 Hz, 1H, H-9), 6.08 (dt, J = 15.7, 6.7 Hz, 1H, H-10), 3.67 (t, J = 6.4 Hz, 2H, H-14), 2.46 (s, 3H, H-1), 2.22 (app. q, J = 7.2 Hz, 2H, H-11), 1.64-1.58 (m, 2H, H-13), 1.56-1.50 (m, 2H, H-12), 1.41 (br. s, 1H, H-15). **^{13}C NMR** (176 MHz, $CDCl_3$): δ (ppm) 145.0, 139.2, 136.7, 134.7, 132.3, 129.6, 128.8, 128.7, 127.6, 62.7, 32.7, 32.2, 25.4, 21.7. **HRMS**: (ESI) *m/z* Calc'd for $C_{14}H_{19}O_3^+$ $[M+H]^+$: 235.1334, Found: 235.1334 (δ = 0.0 ppm).

(E)-6-(3-(tert-butyl)phenyl)hex-5-en-1-ol (1g)

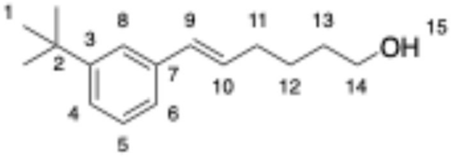

Prepared according to **GP3**, on a 0.6 mmol scale with respect to the silyl protected alcohol vinyl boronate and the corresponding aryl bromide (1.1 eq.). The title compound was obtained as a colourless oil (90.6 mg, 0.39 mmol, 65%). **^{1}H NMR** (700 MHz, $CDCl_3$): δ (ppm) 7.36-7.35 (m, 1H, H-8), 7.25-7.24 (m, 2H, H-4, H-6), 7.20-7.18 (m, 1H, H-5), 6.42 (d, *J* = 15.8 Hz, 1H, H-9), 6.23 (dt, *J* = 15.8, 6.9 Hz, 1H, H-10), 3.68 (t, *J* = 6.3 Hz, 2H, H-14), 2.27 (app. dq, *J* = 7.3, 1.4 Hz, 2H, H-11), 1.68-1.62 (m, 2H, H-13), 1.60-1.54 (m, 2H, H-12), 1.37 (br. s, 1H, H-15), 1.33 (s, 9H, H-1). **^{13}C NMR** (176 MHz, $CDCl_3$): δ (ppm) 151.3, 137.4, 130.6, 130.2, 128.2, 124.0, 123.1, 123.0, 62.9, 34.6, 32.8, 32.3, 31.4, 25.5. **HRMS** (ESI) *m/z* Calc'd for $C_{16}H_{23}O^-$ $[M-H]^-$: 231.1749, Found: 231.1751 (δ = + 0.9 ppm).

(E)-6-(3-methoxyphenyl)hex-5-en-1-ol (1h)

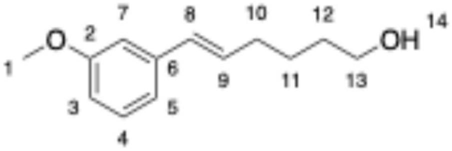

Prepared according to **GP3**, on a 1.5 mmol scale with respect to the silyl protected alcohol vinyl boronate and the corresponding aryl iodide (1.1 eq.). The title compound was obtained as a colourless oil (235 mg, 1.14 mmol, 76%). **^{1}H NMR** (700 MHz, $CDCl_3$): δ (ppm) 7.21 (t, *J* = 7.9 Hz, 1H, H-4), 6.94 (d, *J* = 7.5 Hz, 1H, H-5), 6.88 (s, 1H, H-7), 6.76-6.74 (m, 1H, H-3), 6.36 (d, *J* = 15.8 Hz, 1H, H-8), 6.23 (td, *J* = 15.7, 6.9 Hz, 1H, H-9), 3.81 (s, 3H, H-1), 3.68 (t, *J* = 6.5 Hz, 2H, H-13), 2.48 (app. q, *J* = 7.2 Hz, 2H, H-10), 1.66-1.61 (m, 2H, H-12), 1.59 (br. s, 1H, H-14), 1.58-1.53 (m, 2H, H-11). **^{13}C NMR** (176 MHz, $CDCl_3$): δ (ppm) 159.8, 139.2, 130.9, 130.0, 129.4, 118.6, 112.5, 111.3, 62.9, 55.1, 32.8, 32.3, 25.5. The spectroscopic data matches with that reported in the literature.[8]

Methyl-(E)-3-(6-hydroxyhex-1-en-1-yl)benzoate (1i)

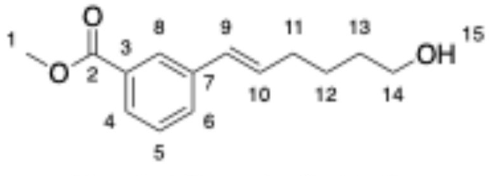

Prepared according to **GP3**, on a 1.1 mmol scale with respect to the silyl protected alcohol vinyl boronate and corresponding aryl iodide (1.1 eq.). The title compound was obtained as a brown oil (220 mg, 0.94 mmol, 85%). **^{1}H NMR** (400 MHz, $CDCl_3$): δ (ppm) 8.01 (t, *J* = 1.7 Hz, 1H, H-8), 7.86 (dt, *J* = 7.7, 1.4 Hz, 1H, H-4), 7.51 (td, *J* = 7.7, 1.3 Hz, 1H, H-6), 7.36 (t, *J* = 7.7 Hz, 1H, H-5), 6.42 (d, *J* = 15.9 Hz, 1H, H-9), 6.33 (dt, *J* = 15.8, 6.7 Hz, 1H, H-10), 3.92 (s, 3H, H-1), 3.68 (app. q, *J* = 5.6 Hz, 2H, H-14), 2.30-2.23 (m, 2H, H-11), 1.69-1.52 (m, 4H, H-12, H-13), 1.31-1.27 (m, 1H, H-15). **^{13}C NMR** (101 MHz, $CDCl_3$): δ (ppm) 167.2, 138.1, 132.0, 130.4, 130.3, 129.2, 128.5, 127.9, 127.0, 62.8, 52.1, 32.7, 32.2, 24.8. **HRMS** (ESI) *m/z* Calc'd for $C_{14}H_{19}O_3^+$ $[M+H]^+$: 235.1334, Found: 235.1334 (δ = 0.0 ppm).

tert-butyl (3-iodophenyl)carbamate

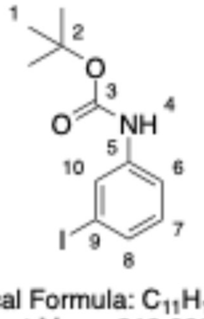

To a solution of *m*-iodoaniline (1.1 g, 5.0 mmol, 1.0 eq.) in MeOH (10 mL) was added TEA (1.29 mL, 10 mmol, 2.0 eq.) and Boc_2O (2.18 g, 10 mmol, 2.0 eq.), and the resulting mixture was heated to 50 °C and stirred for 6 h. Once completed, the reaction mixture was cooled to rt and concentrated *in vacuo*. EtOAc (15 mL) was added to the resulting residue, and the mixture was washed with H_2O (30 mL). The organic phase was washed with brine (30 mL), dried over $MgSO_4$, filtered and concentrated *in vacuo*. The crude was purified by FCC (SiO_2, 25% v/v EtOAc in hexanes) to afford the title compound as an orange solid (1.41 g, 4.39 mmol, 88%). **^{1}H NMR** (700 MHz, $CDCl_3$): δ (ppm) 7.83 (br. s, 1H, H-10), 7.31 (app. dq, *J* = 7.8, 0.8 Hz, 1H, H-8), 7,25 (d, *J* = 7.3 Hz, 1H, H-6), 6.94 (t, *J* = 8.00 Hz, 1H, H-7), 6.86 (br. s, 1H, H-4),

1.50 (s, 9H, H-1). **^{13}C NMR** (376 MHz, $CDCl_3$): δ (ppm) 152.7, 139.7, 131.9, 130.4, 127.3, 117.8, 94.4, 80.9, 28.4. The spectroscopic data matches with that reported in the literature.[9]

tert-butyl (E)-(3-(6-hydroxyhex-1-en-1-yl)phenyl)carbamate (1j)

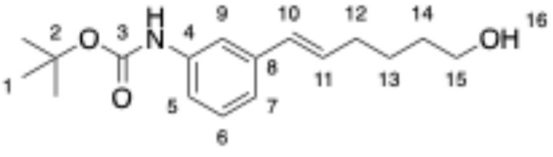

Prepared according to **GP3**, on a 1.2 mmol scale with respect to the silyl protected alcohol vinyl boronate and *tert-butyl (3-iodophenyl)carbamate* (1.1 eq.). The title compound was obtained as a brown amorphous solid (176 mg, 0.61 mmol, 51%). **^{1}H NMR** (700 MHz, $CDCl_3$): δ (ppm) 7.42 (br. s, 1H, H-9), 7.19 (t, *J* = 7.8 Hz, 1H, H-6), 7.13 (d, *J* = 7.8 Hz, 1H, H-5), 7.00 (d, *J* = 7.6 Hz, 1H, H-7), 6.63 (br. s, 1H, NH), 6.33 (d, *J* = 15.8 Hz, 1H, H-10), 6.21 (dt, *J* = 15.7, 6.9 Hz, 1H, H-11), 3.65 (t, *J* = 6.5 Hz, 2H, H-15), 2.21 (app. q, *J* = 7.2 Hz, 2H, H-12), 1.64-1.57 (m, 2H, H-14), 1.55-1.52 (m, 2H, H-13), 1.51 (s, 9H, H-1). **^{13}C NMR** (176 MHz, $CDCl_3$): δ (ppm) 152.9, 138.7, 138.6, 131.0, 129.9, 129.0, 120.8, 117.0, 115.9, 80.5, 62.7, 32.7, 32.2, 28.4, 25.4. **HRMS** (ESI) *m/z* Calc'd for $C_{13}H_{17}NO^+$ $[M\text{-}BOC+H]^+$: 192.1388, Found: 192.1396 (δ = + 4.2 ppm).

(E)-6-(2-methoxyphenyl)hex-5-en-1-ol (1k)

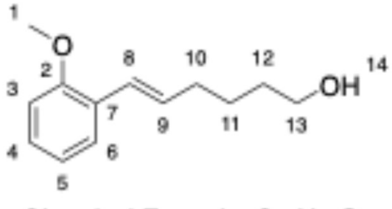

Prepared according to **GP3**, on a 1.2 mmol scale with respect to the silyl protected alcohol vinyl boronate and corresponding aryl iodide (1.1 eq.). The title compound was obtained as a colourless oil (104 mg, 0.50 mmol, 42%). **^{1}H NMR** (400 MHz, $CDCl_3$): δ (ppm) 7.41 (dd, *J* = 7.6, 1.6 Hz, 1H, H-6), 7.21-7.16 (m, 1H, H-4), 6.94-6.88 (m, 1H, H-5), 6.87-6.83 (m, 1H, H-3), 6.72 (d, *J* = 16.0 Hz, 1H, H-8), 6.23 (dt, *J* = 15.9, 6.9 Hz, 1H, H-9), 3.84 (s, 3H, H-1), 3.72-3.64 (m, 2H, H-13), 2.27 (app. dq, *J* = 7.2, 1.4 Hz, 2H, H-10), 1.70-1.51 (m, 4H, H-11, H-12),

1.28-1.24 (m, 1H, H-14). **^{13}C NMR** (101 MHz, $CDCl_3$): δ (ppm) 156.3, 131.3, 127.8, 126.8, 126.4, 124.7, 120.6, 110.8, 62.9, 55.5, 33.1, 32.4, 25.6. **HRMS** (ESI) *m/z* Calc'd for $C_{13}H_{19}O_2^+$ $[M+H]^+$: 207.1380, Found: 207.1381 (δ = + 0.7 ppm).

(E)-6-(o-tolyl)hex-5-en-1-ol (1l)

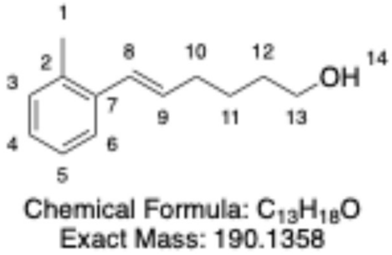

Prepared according to **GP3**, on a 0.6 mmol scale with respect to the silyl protected alcohol vinyl boronate and corresponding aryl iodide (1.1 eq.). The title compound was obtained as a colourless oil (34.2 mg, 0.18 mmol, 30%). **^{1}H NMR** (700 MHz, $CDCl_3$): δ (ppm) 7.40 (d, *J* = 7.4 Hz, 1H, H-6), 7.16-7.11 (m, 3H, H-3, H-4, H-5), 6.58 (d, *J* = 15.7 Hz, 1H, H-8), 6.10 (dt, *J* = 15.6, 6.9 Hz, 1H, H-9), 3.69 (app. q, *J* = 5.8 Hz, 2H, H-13), 2.33 (s, 3H, H-1), 2.28 (app. dq, *J* = 7.4, 1.6 Hz, 2H, H-10), 1.68-1.62 (m, 2H, H-12), 1.60-1.54 (m, 2H, H-11), 1.28-1.26 (m, 1H, H-14). **^{13}C NMR** (176 MHz, $CDCl_3$): δ (ppm) 136.9, 134.9, 131.9, 130.2, 128.0, 126.8, 126.0, 125.5, 62.9, 33.0, 32.3, 25.6, 19.9. **HRMS** (ESI) *m/z* Calc'd for $C_{13}H_{19}O^+$ $[M+H]^+$: 191.1431, Found: 191.1425 (δ = - 3.1 ppm).

(E)-6-(2-chlorophenyl)hex-5-en-1-ol (1m)

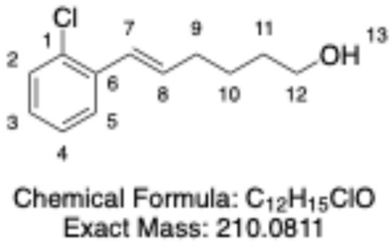

Prepared according to **GP3**, on a 1.2 mmol scale with respect to the silyl protected alcohol vinyl boronate and corresponding aryl bromide (1.1 eq.). The title compound was obtained as a pale-brown solid (146 mg, 0.70 mmol, 58%). **^{1}H NMR** (400 MHz, $CDCl_3$): δ (ppm) 7.52 (dd, *J* = 8.0, 1.21 Hz, 1H, H-6), 7.48 (dd, *J* = 7.8 Hz, 1H, H-2), 7.25-7.83 (m, 1H, H-3), 7.06 (dt, *J* = 7.9 Hz, 1.7 Hz, 1H, H-4), 6.72 (dt, *J* = 15.7, 1.4 Hz, 1H, H-7), 6.16 (dt, *J* = 15.7, 6.9 Hz, 1H, H-8), 3.72-3.65 (m, 2H, H-12), 2.30 (dt, *J* = 7.2, 1.5 Hz, 2H, H-9), 1.70-1.54 (m, 4H,

H-10, H-11), 1.28-1.25 (m, 1H, H-13). **^{13}C NMR** (101 MHz, $CDCl_3$): δ (ppm) 137.6, 133.7, 132.8, 129.1, 128.2, 127.4, 126.8, 123.2, 62.8, 32.8, 32.4, 25.3. **HRMS** (ESI) *m/z* Calc'd for $C_{12}H_{16}ClO^+$ $[M+H]^+$: 211.0884, Found: 211.0874 (δ = - 4.8 ppm).

(E)-6-(2-bromophenyl)hex-5-en-1-ol (1n)

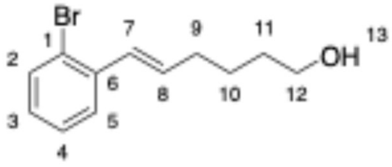

Prepared according to **GP3**, on a 1.2 mmol scale with respect to the silyl protected alcohol vinyl boronate and corresponding aryl iodide (1.1 eq.). The title compound was obtained as a colourless oil (94.5 mg, 0.37 mmol, 31%). **^{1}H NMR** (400 MHz, $CDCl_3$): δ (ppm) 7.52 (dd, *J* = 8.0, 1.1 Hz, 1H, H-2), 7.48 (dd, *J* = 7.8, 1.5 Hz, 1H, H-5), 7.26-7.20 (m, 1H, H-4), 7.07 (dt, *J* = 7.9, 1.6 Hz, 1H, H-3), 6.72 (d, *J* = 15.7 Hz, 1H, H-7), 6.18 (dt, *J* = 15.6, 6.9 Hz, 1H, H-8), 3.69 (app. q, *J* = 5.9 Hz, 2H, H-12), 2.32 (app. dq, *J* = 7.1, 1.3 Hz, 2H, H-9), 1.70-1.56 (m, 4H, H-10, H-11), 1.24 (app. t, *J* = 5.3 Hz, 1H, H-13). **^{13}C NMR** (101 MHz, $CDCl_3$): δ (ppm) 137.6, 133.7, 132.8, 129.1, 128.2, 127.4, 126.9, 123.2, 62.8, 32.8, 32.3, 25.3. **HRMS** The compound did not ionise.

(E)-6-(2-ethylphenyl)hex-5-en-1-ol (1o)

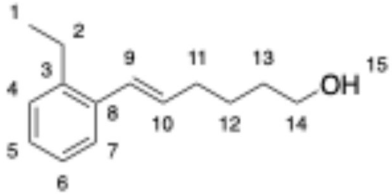

Prepared according to **GP3**, on a 1.1 mmol scale with respect to the silyl protected alcohol vinyl boronate and corresponding aryl bromide (1.1 eq.). The title compound was obtained as a colourless oil (130 mg, 0.64 mmol, 58%). **^{1}H NMR** (700 MHz, $CDCl_3$): δ (ppm) 7.42-7.39 (m, 1H, H-7), 7.18-7.12 (m, 3H, H-4, H-5, H-6), 6.64 (app. d, *J* = 15.6 Hz, 1H, H-9), 6.10 (dt, *J* = 15.6, 6.9 Hz, 1H, H-10), 3.69 (t, *J* = 6.4 Hz, 2H, H-14), 2.69 (q, *J* = 7.6 Hz, 2H, H-2), 2.28 (app. dq, *J* = 7.3, 1.5 Hz, 2H, H-11), 1.67-1.63 (m, 2H, H-13), 1.60-1.55 (m, 2H, H-12), 1.27

(br. s, 1H, H-15), 1.20 (t, *J* = 7.6 Hz, 3H, H-1). **^{13}C NMR** (176 MHz, $CDCl_3$): δ (ppm) 141.0, 136.3, 132.1, 128.6, 127.7, 127.1, 126.0, 125.8, 62.9, 33.0, 32.3, 26.4, 25.6, 15.2. **HRMS** (ESI) *m/z* Calc'd for $C_{14}H_{19}O^-$ $[M-H]^-$: 203.1436, Found: 203.1430 (δ = - 3.0 ppm).

(E)-6-mesitylhex-5-en-1-ol (1p)

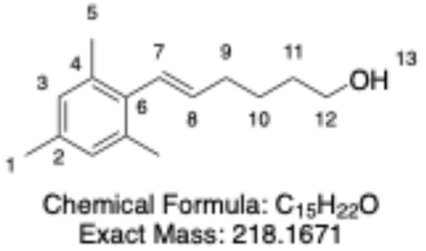

Prepared according to **GP3**, on a 0.6 mmol scale with respect to the silyl protected alcohol vinyl boronate and the corresponding aryl iodide (1.1 eq.). The title compound was obtained as a colourless oil (98.0 mg, 0.45 mmol, 75%). **^{1}H NMR** (700 MHz, $CDCl_3$): δ (ppm) 6.85 (s, 2H, H-3), 6.30 (d, *J* = 16.1 Hz, 1H, H-7), 5.64 (td, *J* = 16.1, 6.9 Hz, 1H, H-8), 3.69 (app. q, *J* = 5.3 Hz, 2H, H-12), 2.29-2.26 (m, 5H, H-1, H-9), 2.25 (s, 6H, H-5), 1.69-1.64 (m, 2H, H-11), 1.59-1.54 (m, 2H, H-10), 1.28-1.25 (m, 1H, H-13). **^{13}C NMR** (176 MHz, $CDCl_3$): δ (ppm) 135.8, 135.7, 135.1, 134.6, 128.4, 127.6, 62.9, 33.2, 32.3, 25.7, 21.0, 20.9. **HRMS** (ESI) *m/z* Calc'd for $C_{15}H_{23}O^+$ $[M-H]^-$: 217.1592, Found: 217.1598 (δ = + 2.8 ppm).

Methyl 5-bromovalerate

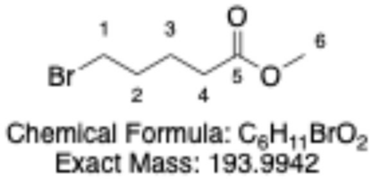

To an RBF was added 5-bromovaleric acid (5.43 g, 30 mmol, 1.0 eq.) and MeOH (50 mL), AcCl (2.14 mL, 30 mmol, 1.0 eq.) was added dropwise at 0 °C. The ice-bath was removed, and the solution was refluxed for 5 h. Once completed, the solvent was removed under reduced pressure to give the crude compound as a yellowish oil at sufficient purity for the next step (5.82 g, 30.0 mmol, quant.) **^{1}H NMR** (700 MHz, $CDCl_3$): δ (ppm) 3.67 (s, 3H, H-6), 3.41 (t, *J* = 6.7 Hz, 2H, H-4), 2.35 (t, *J* = 7.4 Hz, 2H, H-1), 1.92-1.87 (m, 2H, H-3), 1.81-1.76 (m, 2H, H-2). **^{13}C NMR** (101 MHz, $CDCl_3$): δ (ppm) 173.6, 51.6, 33.1, 33.0, 32.0, 23.5. The spectroscopic data matches with that reported in the literature.[10]

(E)-2-methyl-7-phenylhept-6-en-2-ol (1q)

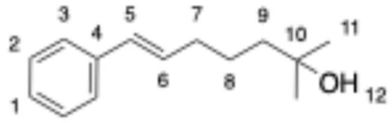

To an oven-dried RBF was added *Methyl (E)-6-phenylhex-5-enoate* (0.10 g, 0.5 mmol, 1.0 eq.) and THF (10 mL). The flask was cooled to 0 °C and MeMgBr (3.0 M in Et_2O, 0.5 mL, 1.5 mmol, 3.0 eq.) was added dropwise. The ice-bath was removed, and the reaction was stirred at rt overnight. Following this, NH_4Cl (sat. soln., 10 mL) was added slowly and the resulting mixture was diluted with EtOAc (10 mL). The layers were separated, and the aqueous layer was further extracted with EtOAc (2 × 20 mL). The combined organic layers were dried over $MgSO_4$, filtered, and concentrated *in vacuo*. The crude mixture was purified by FCC (SiO_2, 0-10% v/v acetone in $CHCl_3$) to give the title compound as a white solid (0.11 g, 0.50 mmol, quant.). **^{1}H NMR** (700 MHz, $CDCl_3$): δ (ppm) 7.35-7.33 (m, 2H, H-3), 7.30-7.27 (m, 2H, H-2), 7.21-7.17 (m, 1H, H-1), 6.39 (d, *J* = 15.8 Hz, 1H, H-5), 6.24 (dt, *J* = 15.8, 6.9 Hz, 1H, H-6), 2.23 (app. dq, *J* = 7.0, 1.3 Hz, 2H, H-7), 1.57-1.50 (m, 4H, H-8, H-9), 1.23 (s, 6H, H-11), 1.20 (br. s, 1H, H-12). **^{13}C NMR** (176 MHz, $CDCl_3$): δ (ppm) 137.8, 130.7, 130.1, 128.5, 126.9, 125.9, 71.0, 43.4, 33.4, 29.3, 24.2. **HRMS**: The compound did not ionise.

(E)-6-(3,4,5-trimethoxyphenyl)hex-5-en-1-ol (1r)

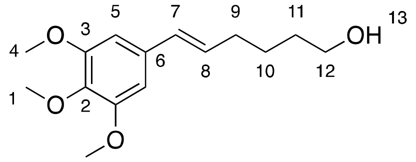

Prepared according to **GP3**, on a 1.1 mmol scale with respect to the silyl protected alcohol vinyl boronate and corresponding aryl bromide (1.1 eq.). The title compound was obtained as a pale-yellow solid (282 mg, 1.06 mmol, 96%). **^{1}H NMR** (400 MHz, $CDCl_3$): δ (ppm) 6.56 (s, 2H, H-5), 6.32 (d, *J* = 15.7 Hz, 1H, H-7), 6.13 (app. t, *J* = 15.7 Hz, 1H, H-8), 3.88 (s, 6H, H-4), 3.83 (s, 3H, H-1), 3.71-3.65 (m, 2H, H-12), 2.24 (app. dq, *J* = 7.3, 1.3 Hz, 2H, H-9), 1.68-1.51 (m, 4H, H-10, H-11), 1.29-1.26 (m, 1H, H-13). **^{13}C NMR** (101 MHz, $CDCl_3$): δ

(ppm) 153.3, 137.3, 133.6, 130.1, 130.0, 103.0, 62.8, 60.9, 50.1, 32.6, 32.3, 25.5. The spectroscopic data matches with that reported in the literature.[11]

(E)-dec-5-en-1-ol (1t)

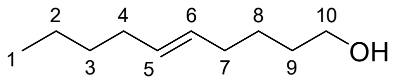

Chemical Formula: $C_{10}H_{20}O$
Exact Mass: 156.1514

To a solution of dec-5-yn-1-ol (0.4 mL, 2.0 mmol, 1.0 eq.) in diglyme (14 mL), $LiAlH_4$ (2.4 M in THF, 5 mL, 12.0 mmol, 6.0 eq.) was added and the mixture was heated at reflux for 18 hours. Due to incomplete conversion of the starting material, another portion of $LiAlH_4$ (2.4 M in THF, 5 mL, 12.0 mmol, 6.0 eq.) was added and the mixture was refluxed for another 18 hours before being cooled to 0 °C and diluted with Et_2O. The reaction mixture was quenched slowly with water, followed by aqueous NaOH solution (2.5 M). The reaction mixture was stirred at room temperature for 20 minutes, then dried over $MgSO_4$, filtered and concentrated *in vacuo*. The crude mixture was dissolved in hexane and washed with water (x5). The organic phase was dried over $MgSO_4$ and concentrated *in vacuo* to afford the title compound (84 mg, 27%) as a colourless oil. **^{1}H NMR** (400 MHz, $CDCl_3$): δ 5.49 – 5.29 (m, 2H, H-5, H-6), 3.64 (t, *J* = 6.5 Hz, 2H, H-10), 2.06 – 1.91 (m, 4H, H-4, H-7), 1.62 – 1.51 (m, 2H, H-9), 1.47 – 1.38 (m, 2H, H-8), 1.35 – 1.28 (m, 4H, H-2, H-3), 0.88 (t, *J* = 6.7 Hz, 3H, H-1). **^{13}C NMR** (176 MHz, $CDCl_3$): δ 131.0, 129.9, 63.1, 32.4, 32.4, 32.4, 31.9, 25.9, 22.3, 14.1. The spectroscopic data matches with that reported in the literature.[12]

Methyl 5-((1-phenyl-1H-tetrazol-5-yl)thio)pentanoate

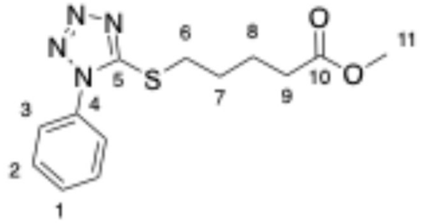

Chemical Formula: $C_{13}H_{16}N_4O_2S$
Exact Mass: 292.0994

To an RBF was added methyl 5-bromovalerate (2.4 mL, 16.5 mmol, 1.1 eq.), 1-phenyl-1H-tetrazole-5-thiol (2.67 g, 15.0 mmol, 1.0 eq.), K_2CO_3 (4.15 g, 30 mmol, 2.0 eq.), NaI (0.22 g, 3.0 mmol, 0.1 eq.) and DMF (15 mL). After being stirred at room temperature for 16 hours, the

reaction was quenched with water and extracted with methylene chloride (x2). The combined organic layers were washed with brine, dried over $MgSO_4$, filtered, and concentrated *in vacuo*. The crude mixture was purified by FCC (SiO_2, 0-50% v/v EtOAc in hexanes) to afford the title compound as a yellow oil (3.01 g, 10.3 mmol, 69%). **^{1}H NMR** (400 MHz, $CDCl_3$) δ 7.60 – 7.49 (m, 5H, H-1, H-2, H-3), 3.66 (s, 3H, H-11), 3.40 (t, *J* = 7.1 Hz, 2H, H-6), 2.37 (t, *J* = 7.2 Hz, 2H, H-9), 1.94 – 1.73 (m, 4H, H-7, H-8). **^{13}C NMR** (176 MHz, $CDCl_3$) δ 173.7, 154.4, 133.8, 130.3, 124.0, 51.8, 33.4, 33.0, 28.7, 23.9. The spectroscopic data matches with that reported in the literature.[13]

Methyl 5-((1-phenyl-1H-tetrazol-5-yl)sulfonyl)pentanoate

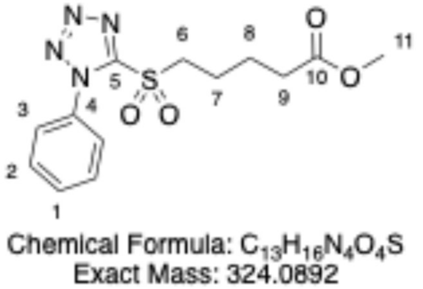

To a stirred solution of ***Methyl 5-((1-phenyl-1H-tetrazol-5-yl)thio)pentanoate*** (2.92 g, 10 mmol, 1.0 eq.) in EtOH (50 mL) at 0 °C was added $(NH_4)_6Mo_7O_{24}\cdot H_2O$ (1.98 g, 1.6 mmol, 0.16 eq.) and H_2O_2 (30% aq. soln., 5.70 mL, 73.4 mmol, 7.34 eq.). The resulting mixture was stirred at rt for 6 h. Upon completion, $Na_2S_2O_5$ (sat. soln., 50 mL) was added to quench the reaction, and the aqueous layer was extracted with ethyl acetate (x2). The combined organic extracts were washed with brine, dried over $MgSO_4$, filtered and concentrated *in vacuo*. The crude mixture was purified by FCC (SiO_2, 0-40% v/v ethyl acetate in hexanes) to afford the title compound as a yellow oil (2.25 g, 6.95 mmol, 70%). **^{1}H NMR** (700 MHz, $CDCl_3$) δ 7.69 (d, *J* = 7.5 Hz, 2H, H-3), 7.66 – 7.58 (m, 3H, H-2, H-1), 3.78 – 3.74 (m, 2H, H-6), 3.68 (s, 3H, H-11), 2.40 (t, *J* = 7.2 Hz, 2H, H-9), 2.02 (p, *J* = 7.6 Hz, 2H, H-7), 1.85 (p, *J* = 7.4 Hz, 2H, H-8). **^{13}C NMR** (176 MHz, $CDCl_3$) δ 173.1, 153.5, 133.1, 131.6, 129.9, 125.2, 55.8, 51.9, 33.3, 23.5, 21.8. The spectroscopic data matches with that reported in the literature.[13]

Methyl (E)-6-phenylhex-5-enoate

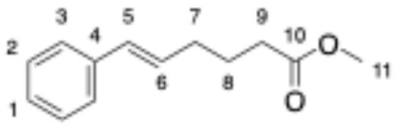

To a solution of ***Methyl 5-((1-phenyl-1H-tetrazol-5-yl)sulfonyl)pentanoate*** (2.10 g, 6.5 mmol, 1.0 eq.) in THF (28 mL) was added NaHMDS (1.0 M in THF, 7.15 mmol, 1.1 eq.) at -78 °C. The resulting mixture was stirred at the same temperature for 15 min, then benzaldehyde (1.0 mL, 9.75 mmol, 1.5 eq.) was added dropwise. The resultant mixture was slowly warmed to room temperature and left to stir for 18 hours. Following this, the reaction was quenched by the addition of water (10 mL) and extracted with EtOAc (3 × 10 mL). The combined organic layers were concentrated *in vacuo*. The crude mixture was dissolved in a minimum amount of methanol and washed with a saturated aqueous solution of sodium bisulfite to remove the aldehyde impurities, extracted with EtOAc, dried with $MgSO_4$, filtered, concentrated *in vacuo*, and then further purified by FCC (SiO_2, 0-5% v/v EtOAc in hexanes) to afford the title compound as a colourless oil (164 mg, 0.82 mmol, 12%). **^{1}H NMR** (700 MHz, $CDCl_3$) δ 7.36 – 7.32 (m, 2H, H-3), 7.29 (t, J = 7.7 Hz, 2H, H-2), 7.23 – 7.17 (m, 1H, H-1), 6.40 (dt, J = 15.9, 1.6 Hz, 1H, H-5), 6.18 (dt, J = 15.8, 7.0 Hz, 1H, H-6), 3.67 (s, 3H, H-11), 2.37 (t, J = 7.5 Hz, 2H, H-9), 2.26 (app. qd, J = 7.2, 1.5 Hz, 2H, H-7), 1.83 (p, J = 7.4 Hz, 2H, H-8). **^{13}C NMR** (176 MHz, $CDCl_3$) δ 174.2, 137.7, 130.9, 129.7, 128.6, 127.1, 126.1, 51.7, 33.5, 32.5, 24.6. The spectroscopic data matches with that reported in the literature.[5]

(E)-N-methyl-N,6-diphenylhex-5-enamide

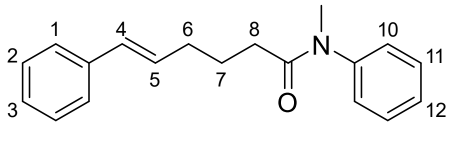

To a solution of *N*-methylaniline (0.1 mL, 1.0 mmol, 2.0 eq.) in Et_2O (20 mL) at 0 °C, MeMgBr (0.8 mL, 1.4 M in THF/toluene, 2.1 eq.) was added dropwise. ***Methyl 5-((1-phenyl-1H-tetrazol-5-yl)sulfonyl)pentanoate*** (102 mg, 0.5 mmol, 1.0 eq.) in Et_2O (10 mL) was added and the reaction was stirred for 3 h at room temperature. The reaction was quenched with water (20 mL) and extracted with EtOAc (x3). The combined organic layers were washed with HCl (1

N, 10 mL) and brine (10 mL), dried with $MgSO_4$, filtered and concentrated *in vacuo*. The crude was purified by FCC (SiO_2 v/v 0-20% EtOAc in hexanes) to afford. the title compound as a colourless oil (104 mg, 0.37 mmol, 75%). **^{1}H NMR** (700 MHz, $CDCl_3$): δ 7.38 (t, *J* = 7.7 Hz, 2H), 7.33 – 7.26 (m, 5H), 7.20 – 7.14 (m, 3H), 6.29 (d, *J* = 15.7 Hz, 1H, H-4), 6.06 (dt, *J* = 15.2, 6.9 Hz, 1H, H-5), 3.26 (s, 3H, H-9), 2.13 (dt, *J* = 13.9, 7.0 Hz, 4H, H-6, H-8), 1.79 – 1.76 (m, 2H, H-7). **^{13}C NMR** (176 MHz, $CDCl_3$): δ 173.1, 144.3, 137.8, 130.5, 130.2, 129.9, 128.6, 127.8, 127.5, 127.0, 126.1, 37.4, 33.5, 32.5, 25.2. HRMS (ESI) m/z Calc'd for $C_{19}H_{22}NO^+$ $[M+H]^+$: 280.1696, Found: 280.1709 (δ = + 4.5 ppm).

2.3 Trans alkenyl alcohol substrate synthesis (5-C Chain length)

tert-butyldimethyl(pent-4-yn-1-yloxy)silane

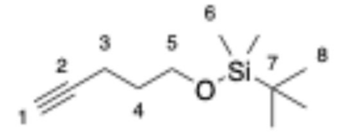

Chemical Formula: $C_{11}H_{22}OSi$
Exact Mass: 198.1440

According to **GP1**, 5-pentyn-1-ol (4.29 g, 51.0 mmol, 1.0 eq.) and TBSCl (9.04 g, 60.0 mmol, 1.2 eq.) were dissolved in methylene chloride (100 ml) and TEA (7.76 ml, 60.0 mmol, 1.2 eq.) was added to the flask. FCC of the crude mixture revealed the title compound as a colourless oil (9.74 g, 49.0 mmol, 96%). **^{1}H NMR** (400 MHz, $CDCl_3$): δ (ppm) 3.69 (t, J = 6.0 Hz, 2H, H-5), 2.27 (td, J = 7.1, 2.6 Hz, 2H, H-3), 1.88 (t, J = 2.6 Hz, 1H, H-1), 1.75-1.71 (m, 2H, H-4), 0.88 (s, 9H, H-8), 0.05 (s, 6H, H-6). **^{13}C NMR** (101 MHz, $CDCl_3$): δ (ppm) 84.3, 68.2, 61.4, 31.5, 25.9, 18.3, 14.8, -5.4. The spectroscopic data matches with that reported in the literature.[14]

(E)-tert-butyldimethyl((5-(4,4,5,5-tetramethyl-1,3,2-dioxaborolan-2-yl)pent-4-en-1-yl)oxy)silane

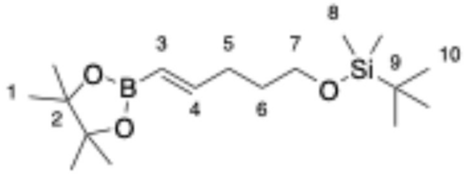

Chemical Formula: $C_{17}H_{35}BO_3Si$
Exact Mass: 326.2449

Prepared according to **GP2**, on a 14.3 mmol scale with respect to the silyl protected alcohol. The title compound was obtained as a colourless oil (2.00 g, 6.13 mmol, 43%). **^{1}H NMR** (700 MHz, $CDCl_3$): δ (ppm) 6.66 (d, J = 17.9 Hz, 1H, H-3), 5.44 (dt, J = 17.8, 1.4 Hz, 1H, H-4), 3.61 (t, J = 6.4 Hz, 2H, H-7), 2.24-2.16 (m, 2H, H-5), 1.67-1.60 (m, 2H, H-6), 1.26 (s, 12H, H-1), 0.88 (s, 9H, H-10), 0.03 (s, 6H, H-8). **^{13}C NMR** (176 MHz, $CDCl_3$): δ (ppm) 154.2, 83.0, 62.6, 53.4, 32.1, 31.4, 25.9, 24.8, 18.3, -5.3. The spectroscopic data matches with that reported in the literature.[14]

(E)-5-phenylpent-4-en-1-ol (5a)

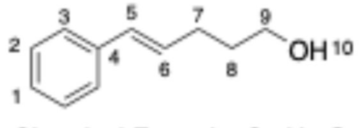

Prepared according to **GP3**, on a 2.0 mmol scale with respect to the silyl protected alcohol vinyl boronate and corresponding aryl iodide (1.1 eq.). The title compound was obtained as a colourless oil (130 mg, 0.80 mmol, 40%). **^{1}H NMR** (700 MHz, $CDCl_3$): δ (ppm) 7.37-7.33 (m, 2H, H-3), 7.31-7.28 (m, 2H, H-2), 7.22-7.18 (m, 1H, H-1), 6.43 (d, *J* = 15.8 Hz, 1H, H-5), 6.24 (dt, *J* = 15.8, 7.0 Hz, 1H, H-6), 3.71 (t, *J* = 6.5 Hz, 2H, H-9), 2.32 (app. Dq, *J* = 7.6, 1.4 Hz, 2H, H-7), 1.76 (app. P, *J* = 7.3 Hz, 2H, H-8), 1.41 (br. S, 1H, H-10). **^{13}C NMR** (176 MHz, $CDCl_3$): δ (ppm) 137.6, 130.4, 130.1, 128.5, 127.0, 126.0, 62.4, 32.3, 29.3. The spectroscopic data matches with that reported in the literature.[15]

(E)-5-(2-isopropylphenyl)pent-4-en-1-ol (5b)

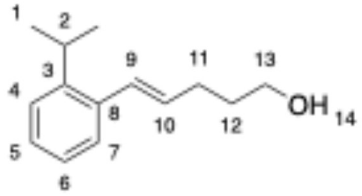

Prepared according to **GP3**, on a 1.07 mmol scale with respect to the silyl protected alcohol vinyl boronate and corresponding aryl bromide (1.1 eq.). The title compound was obtained as a colourless oil (174 mg, 0.86 mmol, 80%). **^{1}H NMR** (700 MHz, $CDCl_3$): δ (ppm) 7.38 (d, *J* = 7.7 Hz, 1H, H-7), 7.25 (d, *J* = 7.1 Hz, 1H, H-4), 7.22 (t, *J* = 7.4 Hz, 1H, H-5), 7.14 (t, *J* = 7.5 Hz, 1H, H-6), 6.76 (d, *J* = 15.5 Hz, 1H, H-9), 6.08-6.03 (m, 1H, H-10), 3.73 (t, *J* = 6.3 Hz, 2H, H-13), 3.24 (s, *J* = 6.7 Hz, 1H, H-2), 2.34 (app. Q, *J* = 7.2 Hz, 2H, H-11), 1.78 (app. P, *J* = 7.1 Hz, 2H, H-12), 1.41 (br. S, 1H, H-14), 1.24 (d, *J* = 6.8 Hz, 6H, H-1). **^{13}C NMR** (176 MHz, $CDCl_3$): δ (ppm) 145.3, 136.0, 132.0, 128.3, 127.3, 126.4, 125.8, 124.8, 62.5, 32.4, 29.6, 29.1, 23.4. **HRMS** The compound did not ionise.

(E)-5-(3-methoxyphenyl)pent-4-en-1-ol (5c)

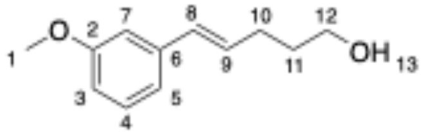

Chemical Formula: $C_{12}H_{16}O_2$
Exact Mass: 192.1150

Prepared according to **GP3**, on a 1.07 mmol scale with respect to the silyl protected alcohol vinyl boronate and the corresponding aryl iodide (1.1 eq.). The title compound was obtained as a colourless oil (205 mg, 0.93 mmol, 89%). **^{1}H NMR** (700 MHz, $CDCl_3$): δ (ppm) 7.21 (t, *J* = 7.9 Hz, 1H, H-4), 6.94 (d, *J* = 7.6 Hz, 1H, H-5), 6.88 (t, *J* = 2.0 Hz, 1H, H-7), 6.77-6.75 (m, 1H, H-3), 6.39 (d, *J* = 15.8 Hz, 1H, H-8), 6.23 (dt, *J* = 15.7, 7.0 Hz, 1H, H-9), 3.81 (s, 3H, H-1), 3.74-3.69 (m, 2H, H-12), 2.33-2.30 (m, 2H, H-10), 1.76 (app. P, *J* = 7.3 Hz, 2H, H-11), 1.30 (br. S, 1H, H-13). **^{13}C NMR** (176 MHz, $CDCl_3$): δ (ppm) 159.8, 139.1, 130.4, 130.3, 129.5, 118.7, 112.6, 111.3, 62.4, 55.2, 32.2, 29.3. The spectroscopic data matches with that reported in the literature.[16]

(E)-5-(3,5-di-tert-butylphenyl)pent-4-en-1-ol (5d)

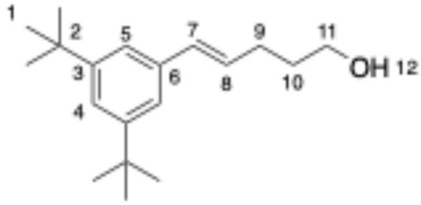

Chemical Formula: $C_{19}H_{30}O$
Exact Mass: 274.2297

Prepared according to **GP3**, on a 1.2 mmol scale with respect to the silyl protected alcohol vinyl boronate and corresponding aryl bromide (1.1 eq.). The title compound was obtained as a colourless oil (197 mg, 0.72 mmol, 60%). **^{1}H NMR** (700 MHz, $CDCl_3$): δ (ppm) 7.30-7.29 (m, 1H, H-4), 7.20 (d, *J* = 1.7 Hz, 2H, H-5), 6.45 (d, *J* = 15.4 Hz, 1H, H-7), 6.22 (dt, *J* = 15.7, 7.0 Hz, 1H, H-8), 3.72 (app. Q, *J* = 5.6 Hz, 2H, H-11), 2.36-2.29 (m, 2H, H-9), 1.77 (app. P, *J* = 6.9 Hz, 2H, H-10), 1.33 (s, 18H, H-1). **^{13}C NMR** (176 MHz, $CDCl_3$): δ (ppm) 150.8, 136.8, 131.3, 129.3, 121.3, 120.3, 62.4, 34.8, 32.3, 31.4, 29.3. **HRMS** The compound did not ionise.

Methyl (E)-2-(5-hydroxypent-1-en-1-yl)benzoate (5e)

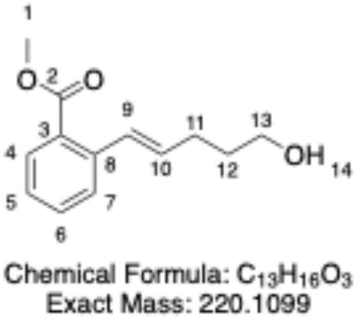

Prepared according to **GP3**, on a 1.07 mmol scale with respect to the silyl protected alcohol vinyl boronate and corresponding aryl iodide (1.1 eq.). The title compound was obtained as a colourless oil (121 mg, 0.55 mmol, 51%). **^{1}H NMR** (700 MHz, $CDCl_3$): δ (ppm) 7.85 (d, J = 7.8 Hz, 1H, H-4), 7.52 (d, J = 7.8 Hz, 1H, H-7), 7.45 (t, J = 7.6 Hz, 1H, H-6), 7.27 (t, J = 7.7 Hz, 1H, H-5), 7.15 (d, J = 15.7 Hz, 1H, H-9), 6.12 (dt, J = 16.1, 7.0 Hz, 1H, H-10), 3.90 (s, 3H, H-1), 3.75 – 3.71 (m, 3H, H-13), 2.37 (app. Q, J = 7.2 Hz, 2H, H-11), 1.78 (app. P, J = 6.8 Hz, 2H, H-12), 1.56 (br. S, 1H, H-14). **^{13}C NMR** (176 MHz, $CDCl_3$): δ (ppm) 168.0, 139.7, 132.9, 132.1, 130.0, 129.4, 128.1, 127.4, 126.7, 75.0, 62.3, 52.1, 31.9, 29.6. **HRMS** (ESI) *m/z* Calc'd for $C_{13}H_{17}O_3^+$ $[M+H]^+$: 221.1178, Found: 221.1179 (δ = + 0.5 ppm).

(E)-2-methyl-6-phenylhex-5-en-2-ol (5f)

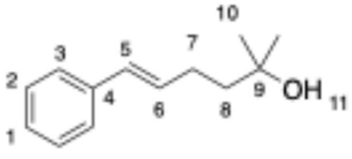

To an oven-dried RBF was added *Ethyl (E)-5-phenylpent-4-enoate* (0.10 g, 0.50 mmol, 1.0 eq.) and THF (10 mL). The flask was cooled to 0 °C and MeMgBr (3.0 M in Et_2O, 0.5 mL, 1.5 mmol, 3.0 eq.) was added dropwise. The ice-bath was removed, and the reaction was stirred at rt overnight. Following this, NH_4Cl (sat. soln., 10 mL) was added slowly and the resulting mixture was then diluted with EtOAc (10 mL). The layers were separated, and the aqueous layer was further extracted with EtOAc (2 × 20 mL). The combined organic layers were dried over $MgSO_4$, filtered, and concentrated *in vacuo*. The crude mixture was purified by FCC (SiO_2, 0-10% v/v acetone in $CHCl_3$) to give the title compound as a white solid (0.11 g, 0.50 mmol, quant.). **^{1}H NMR** (700 MHz, $CDCl_3$): δ (ppm) 7.36 (d, J = 7.5 Hz, 2H, H-3), 7.31 (t, J = 7.6 Hz, 2H, H-2), 7.21 (t, J = 7.3 Hz, 1H, H-1), 6.44 (d, J = 15.8 Hz, 1H, H-5), 6.27 (dt, J =

15.9, 6.9 Hz, 1H, H-6), 2.35-2.31 (m, 2H, H-7), 1.69-1.65 (m, 2H, H-8), 1.28 (s, 6H, H-10). **^{13}C NMR** (176 MHz, $CDCl_3$): δ (ppm) 137.8, 130.9, 129.9, 128.5, 126.9, 126.0, 70.9, 43.3, 29.3, 28.1. The spectroscopic data matches with that reported in the literature.[17]

3. Synthesis of Chiral Rh-Catalysts

The syntheses of catalysts Rh_2(**A**)$_2$•(**4a**)$_2$•(Pyr)$_2$, Rh_2(**B**)$_2$•(**4a**)$_2$•(Pyr)$_2$, Rh_2(**C**)$_2$•(**4a**)$_2$•(Pyr)$_2$, Rh_2(**D**)$_2$•(**4a**)$_2$•(Pyr)$_2$, Rh_2(**E**)$_2$•(**4a**)$_2$•(Pyr)$_2$, Rh_2(**C**)$_2$•(**4b**)$_2$•(Pyr)$_2$, Rh_2(**C**)$_2$•(**4c**)$_2$•(Pyr)$_2$, Rh_2(**C**)$_2$•(**4d**)$_2$•(Pyr)$_2$, Rh_2(**C**)$_2$•(**4e**)$_2$•(Pyr)$_2$, Rh_2(**A**)$_2$•(**4b**)$_2$•(Pyr)$_2$, Rh_2(**A**)$_2$•(**4f**)$_2$•(Pyr)$_2$,

$Rh_2(\mathbf{A})_2$•$(\mathbf{4g})_2$•$(Pyr)_2$, $Rh_2(\mathbf{A})_2$•$(\mathbf{4h})_2$•$(Pyr)_2$, $Rh_2(\mathbf{A})_2$•$(\mathbf{4i})_2$•$(Pyr)_2$, $Rh_2(\mathbf{A})_2$•$(\mathbf{4j})_2$•$(Pyr)_2$, and $Rh_2(\mathbf{A})_2$•$(\mathbf{4k})_2$•$(Pyr)_2$ have all been previously reported.[18,19,20] All catalysts were used in their pyridine-ligated form.

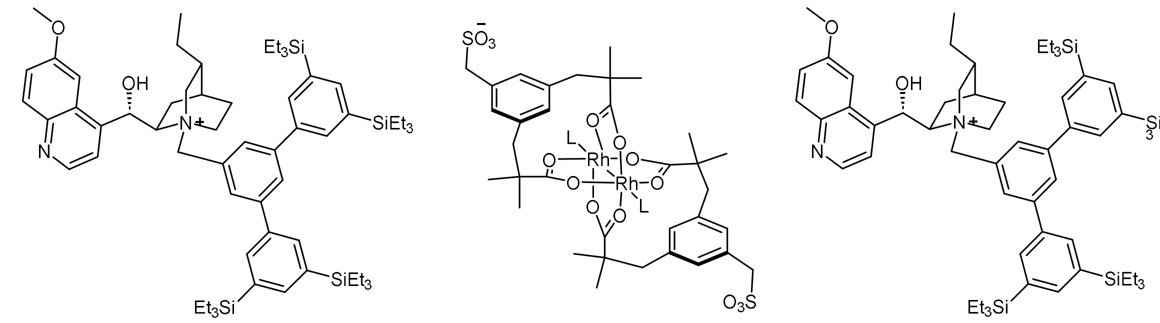

$Rh_2(\mathbf{A})_2$•$(\mathbf{4a})_2$•$(Pyr)_2$ L = pyridine

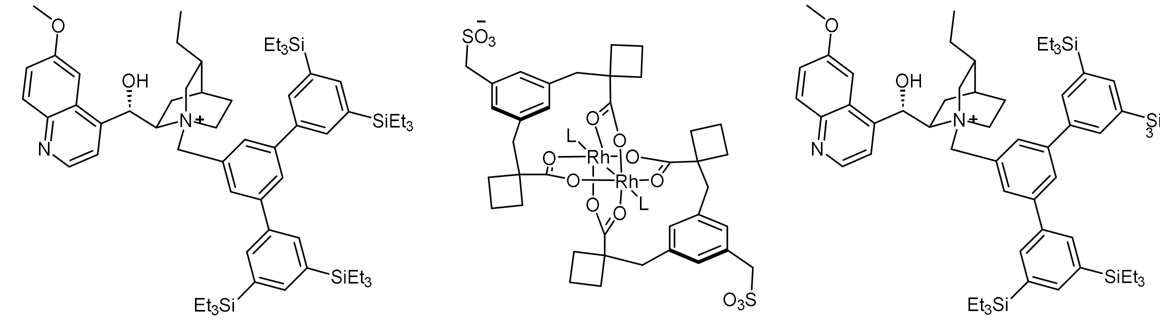

$Rh_2(\mathbf{B})_2$•$(\mathbf{4a})_2$•$(Pyr)_2$ L = pyridine

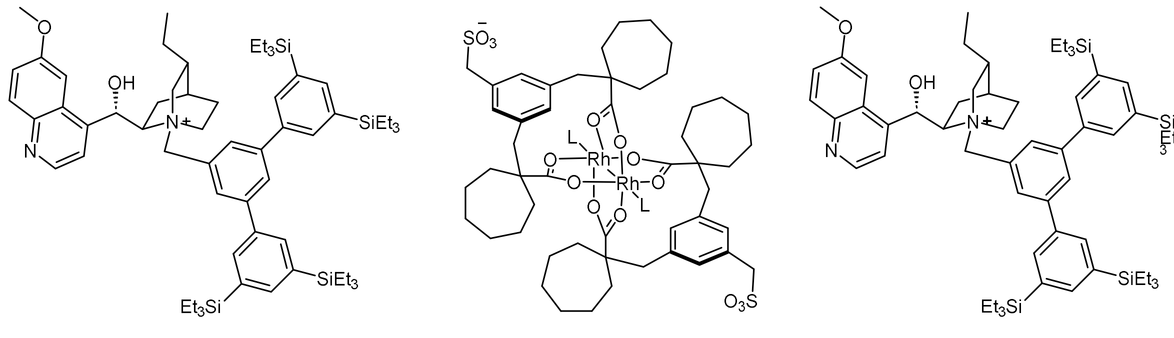

$Rh_2(\mathbf{D})_2$•$(\mathbf{4a})_2$•$(Pyr)_2$ L = pyridine

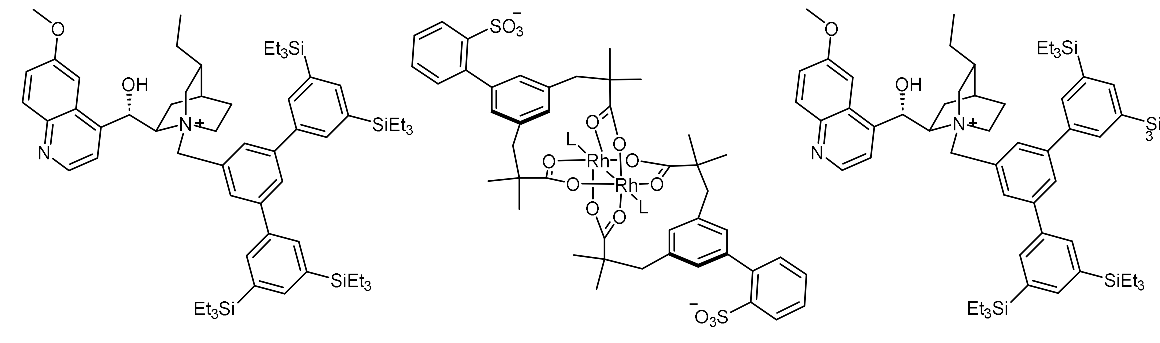

$Rh_2(\mathbf{E})_2$•$(\mathbf{4a})_2$•$(Pyr)_2$ L = pyridine

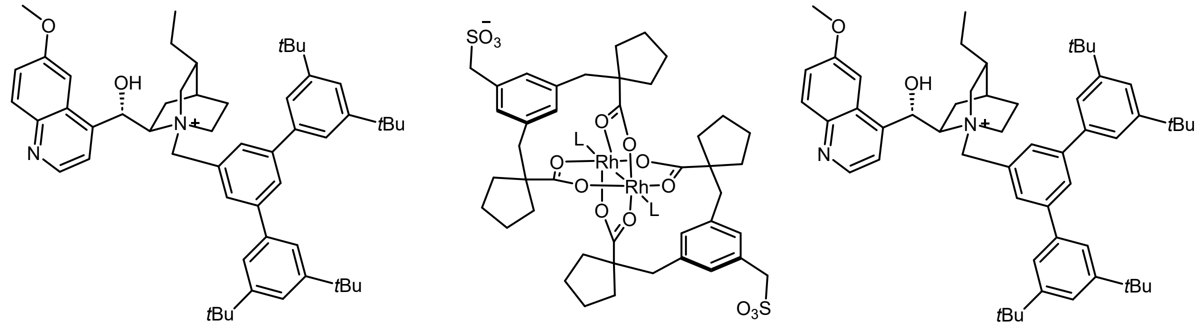

$Rh_2(\mathbf{C})_2$•$(\mathbf{4b})_2$•$(Pyr)_2$

L = pyridine

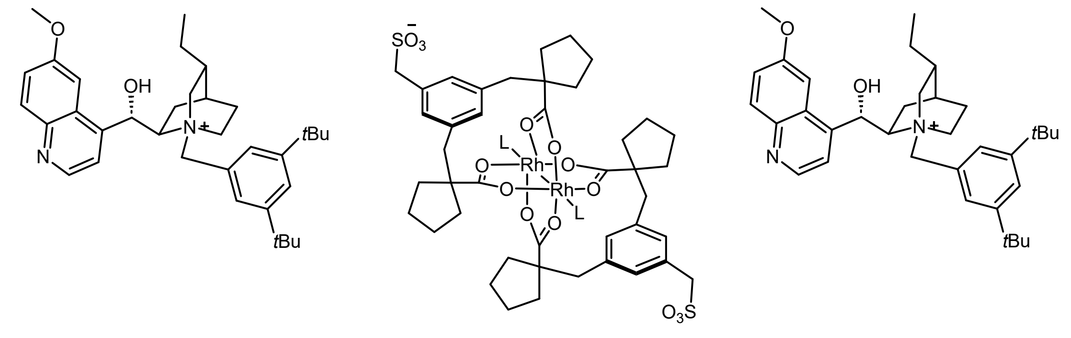

$Rh_2(\mathbf{C})_2$•$(\mathbf{4c})_2$•$(Pyr)_2$

L = pyridine

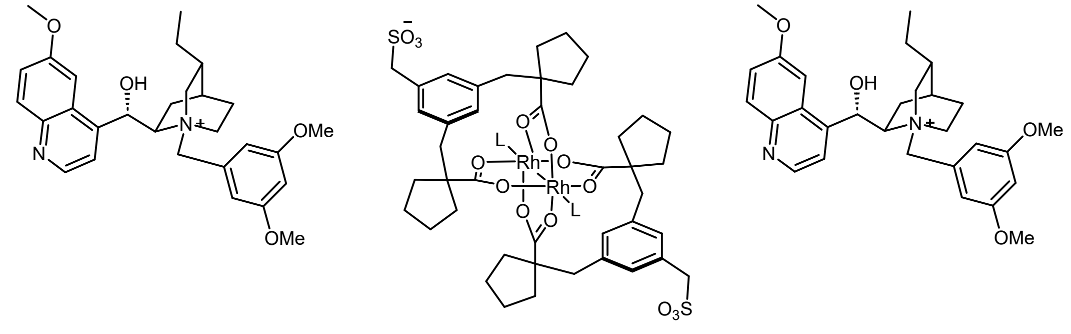

$Rh_2(\mathbf{C})_2$•$(\mathbf{4d})_2$•$(Pyr)_2$

L = pyridine

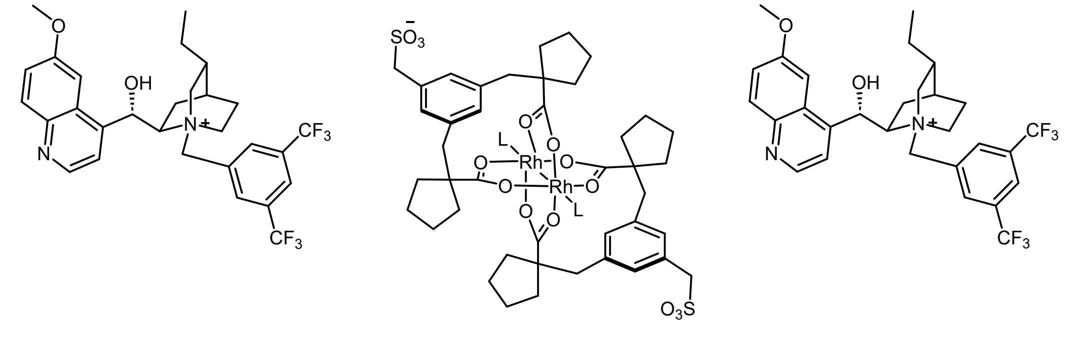

$Rh_2(\mathbf{C})_2$•$(\mathbf{4e})_2$•$(Pyr)_2$

L = pyridine

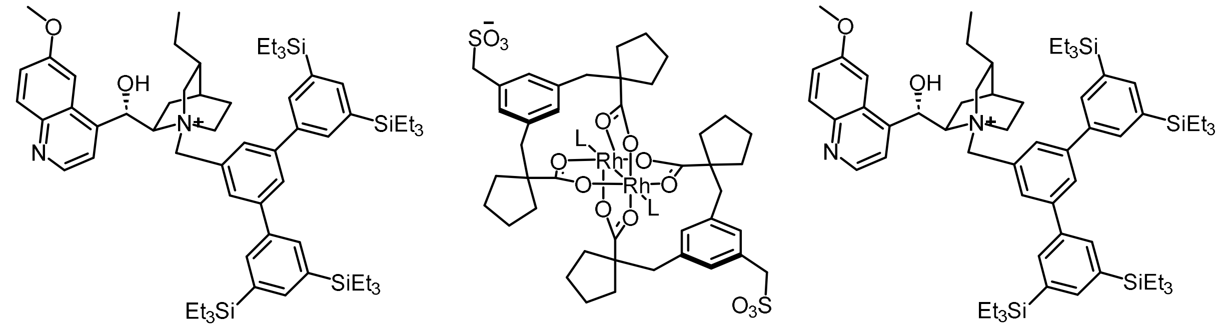

$Rh_2(\mathbf{C})_2{\cdot}(\mathbf{4a})_2{\cdot}(Pyr)_2$

L = pyridine

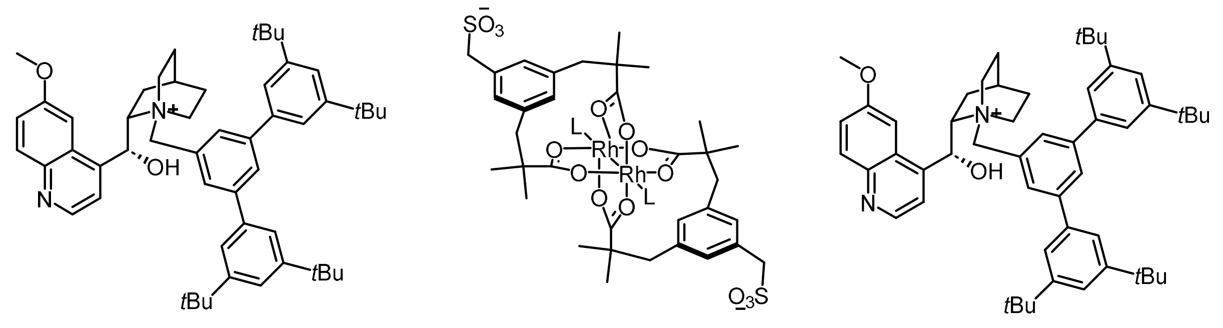

$Rh_2(\mathbf{A})_2{\cdot}(\mathbf{4f})_2{\cdot}(Pyr)_2$

L = pyridine

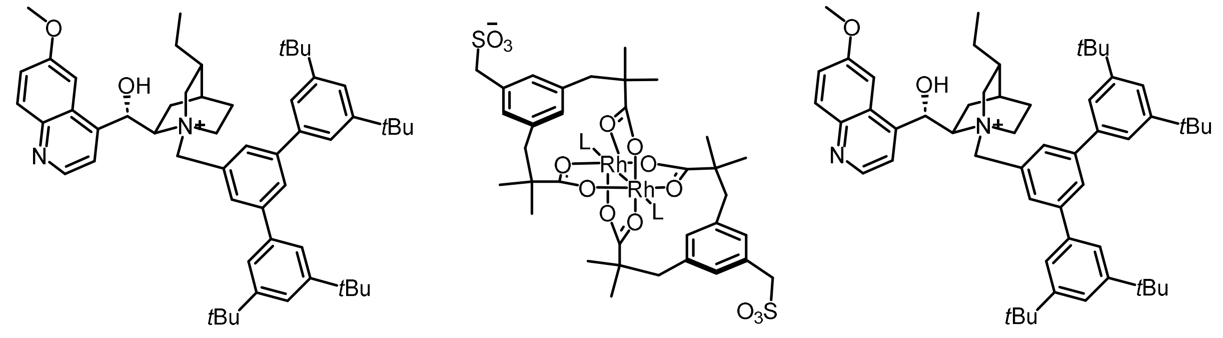

$Rh_2(\mathbf{A})_2{\cdot}(\mathbf{4b})_2{\cdot}(Pyr)_2$

L = pyridine

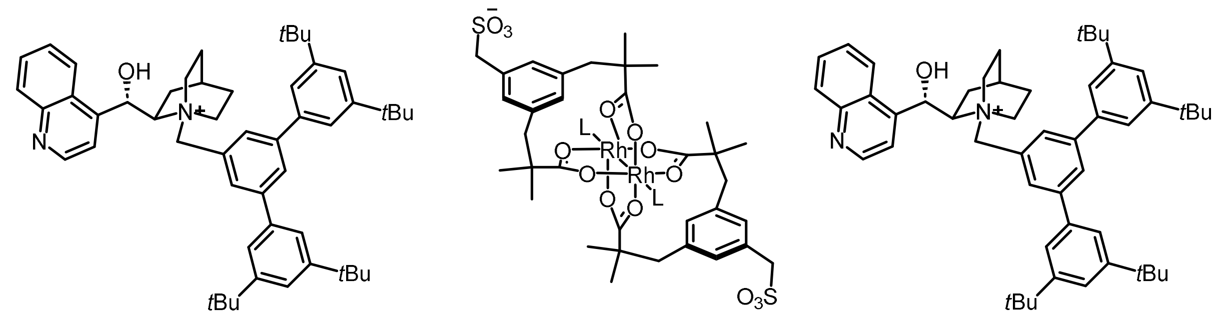

$Rh_2(\mathbf{A})_2{\cdot}(\mathbf{4g})_2{\cdot}(Pyr)_2$

L = pyridine

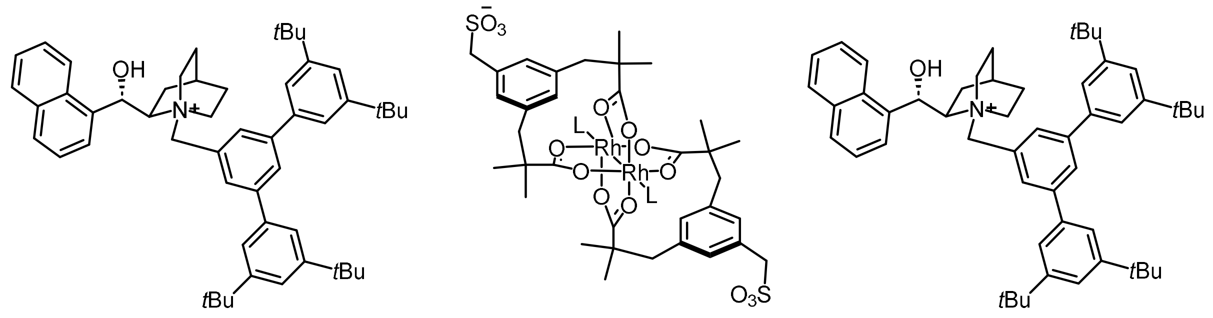

$Rh_2(\mathbf{A})_2$•$(\mathbf{4h})_2$•$(Pyr)_2$ L = pyridine

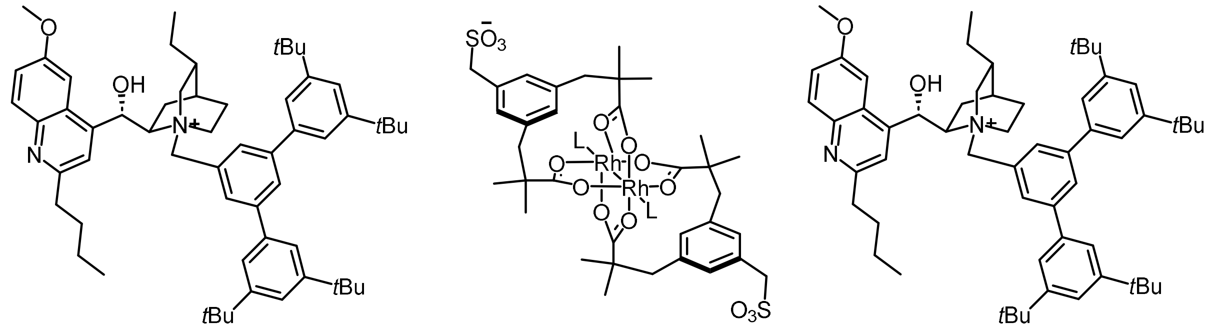

$Rh_2(\mathbf{A})_2$•$(\mathbf{4i})_2$•$(Pyr)_2$ L = pyridine

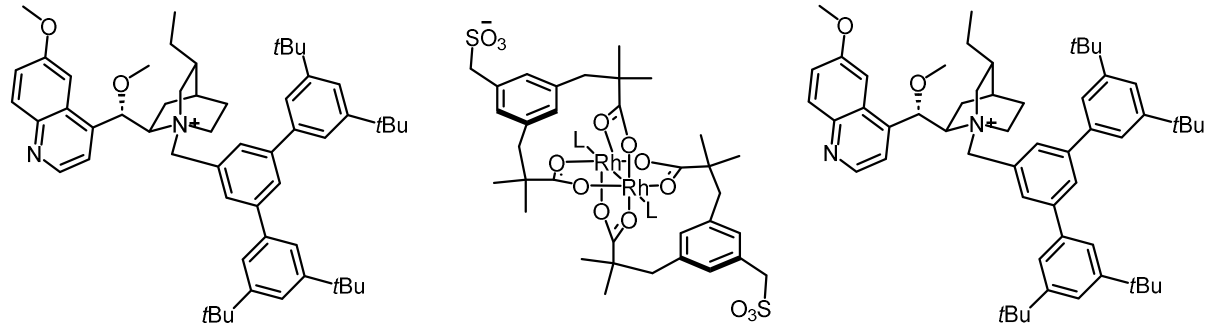

$Rh_2(\mathbf{A})_2$•$(\mathbf{4j})_2$•$(Pyr)_2$ L = pyridine

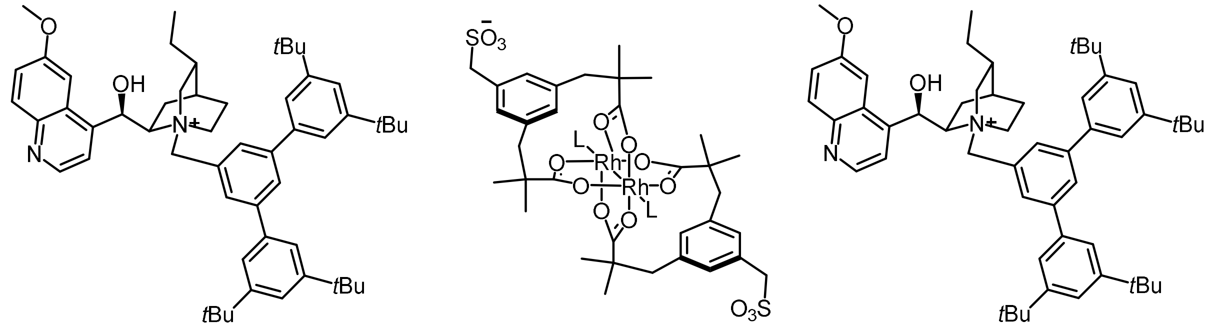

$Rh_2(\mathbf{A})_2$•$(\mathbf{4k})_2$•$(Pyr)_2$ L = pyridine

4. Sulfamate ester, Additive and Oxidant Synthesis

The syntheses of **NH_2Pfps, NH_2Tces, $C_6F_5I(OTFA)_2$** and **PFIOB** have all been previously reported.[18]

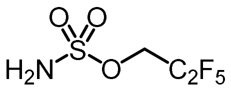

NH_2Pfps

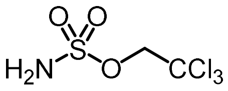

NH_2Tces

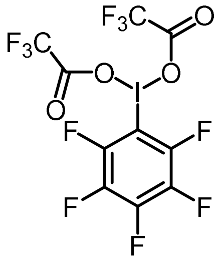

$C_6F_5I(OTFA)_2$

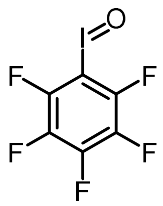

PFIOB

5. Enantioselective Intermolecular Allylic Amination Products

General Procedure 4 (GP4) [Enantioselective Rh-catalysed Allylic Amination Reaction]

To a 4.0 mL crimp-top vial was added substrate (0.1 mmol, 1.0 eq.), **[Rh]** (1.0 mol%), $TcesNH_2$ (27.4 mg, 0.12 mmol, 1.2 eq.), and 1,3-DFB (0.5 mL). The vial was cooled to - 35 °C over 15 min. Following this, PFIOB (62.0 mg, 0.2 mmol, 2.0 eq.) and $C_6F_5I(OTFA)_2$ (10.4 mg, 20 mol%) were added to the vial at - 35 °C in one portion. The vial was sealed and stirred at - 35 °C overnight. Following this, thiourea (sat. soln., 1.0 mL) was added to quench the reaction, followed by $CHCl_3$ (1.0 mL). The layers were separated, and the aqueous layer extracted further with $CHCl_3$ (3 × 1.0 mL). The combined organic layers were dried with $MgSO_4$, filtered and concentrated *under a gentle stream of nitrogen* . Purification by FCC (SiO_2, 0-30% v/v acetone in $CHCl_3$) afforded the title compounds.

General Procedure 5 (GP5) [Racemic Rh-catalysed Allylic Amination Reaction]

To a 4.0 mL crimp-top vial was added substrate (0.1 mmol, 1.0 eq.), $Rh_2(esp)_2$ (3.05 mg, 4.0 mol%), $TcesNH_2$ (0.12 mmol), and 1,3-DFB (0.5 mL). The vial was cooled to - 35 °C over 15 min. Following this, PFIOB (0.2 mmol, 2.0 eq.) was added to the vial at - 35 °C in one portion. The vial was sealed and stirred at -35 °C overnight. Following this, thiourea (sat. soln., 1.0 mL) was added to quench the reaction, followed by $CHCl_3$ (1.0 mL). The layers were separated, and the aqueous layer extracted further with $CHCl_3$ (3 × 1.0 mL). The combined organic layers were dried with $MgSO_4$, filtered and concentrated. Purification by FCC (SiO_2, 0-30% v/v acetone in $CHCl_3$) afforded the title compounds.

NOTE*: If $Rh_2(esp)_2$ or* ***$Rh_2(A)_2$·(Bu_4N_2)*** *gave no racemic allylic amination product following completion of the reaction,* ***$Rh_2(A)_2$·(4f)·Pyr*** *(2.6 mg, 1.0 mol%) was used to access the opposite enantiomers. In SFC traces provided at the end of the experimental section, some will show racemate while others will show the opposite enantiomer if the racemate could not be obtained.*

5.1 Substrate scope (6-C Chain length)

2,2,2-trichloroethyl (R,E)-(6-hydroxy-1-phenylhex-1-en-3-yl)sulfamate (2a)

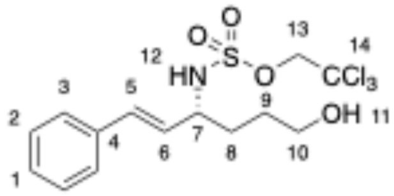

Chemical Formula: $C_{14}H_{18}Cl_3NO_4S$
Exact Mass: 401.0022

The following compound was prepared using **GP4** with **1a** as the starting material and **Rh2(C)2·(4a)2**·(Pyr)2 (3.26 mg, 1.0 mol%) as the [Rh] catalyst. Following purification, the title compound was isolated as a colourless oil (19.6 mg, 0.049 mmol, 49%, 93% *ee*). **^{1}H NMR** (700 MHz, $CDCl_3$): δ (ppm) 7.38-7.35 (m, 2H, H-3), 7.34-7.29 (m, 2H, H-2), 7.27-7.23, 1H, H-1), 6.65 (d, *J* = 15.8 Hz, 1H, H-5), 6.12 (dd, *J* = 15.9, 7.3 Hz, 1H, H-6), 5.82 – 5.66 (m, 1H, H-12), 4.61 (s, 2H, H-13), 4.23 (app. q, *J* = 6.7 Hz, 1H, H-7), 3.75 – 3.72 (m, 2H, H-10), 1.89 (app. q, *J* = 7.0 Hz, 2H, H-8), 1.78-1.71 (m, 2H, H-9), 1.62 (br. s, 1H, H-11). **^{13}C NMR** (176 MHz, $CDCl_3$): δ (ppm) 136.0, 132.6, 138.7, 128.1, 128.0, 126.6, 93.5, 78.1, 62.4, 57.2, 32.4, 27.9. $[\alpha]_D^{25.0}$ = +22.8 (c. 1.15, $CHCl_3$). **HRMS** (ESI) *m/z* Calc'd for $C_{14}H_{17}Cl_3NO_4S^-$ $[M-H]^-$: 399.9944, Found: 399.9941 (δ = - 0.8 ppm). **Chiral SFC analysis** DAICEL CHIRALPAK IG (CO_2:MeOH, 80:20, 2.5 mL min^{-1}, 40 °C) t_R = 3.65 (minor), 4.00 (major) minutes.

2,2,2-trichloroethyl (R,E)-(1-(3-acetylphenyl)-6-hydroxyhex-1-en-3-yl)sulfamate (2b)

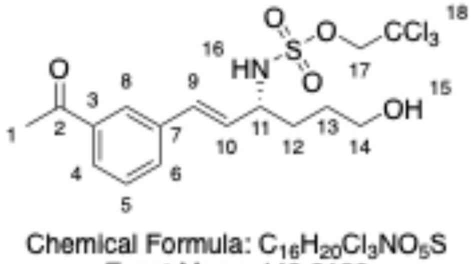

Chemical Formula: $C_{16}H_{20}Cl_3NO_5S$
Exact Mass: 443.0128

The following compound was prepared using **GP4** with **1b** as the starting material and **Rh2(C)2·(4a)2**·(Pyr)2 (3.26 mg, 1.0 mol%) as the [Rh] catalyst. Following purification, the title compound was isolated as a colourless oil (23.0 mg, 0.052 mmol, 52%, 73% *ee*). **^{1}H NMR** (700 MHz, $CDCl_3$): δ (ppm) 7.96 (s, 1H, H-8), 7.82 (app. dd, *J* = 7.7, 0.7 Hz, 1H, H-4), 7.55

(d, J = 7.6 Hz, 1H, H-6), 7.42 (t, J = 7.7 Hz, 1H, H-5), 6.69 (d, J = 15.9 Hz, 1H, H-9), 6.23 (dd, J = 15.9, 7.1 Hz, 1H, H-10), 6.00 (br. s, 1H, H-16), 4.63-4.59 (m, 2H, H-17), 4.26 (app. q, J = 6.7 Hz, 1H, H-11), 3.78-3.72 (m, 2H, H-14), 2.61 (s, 3H, H-1), 1.90 (app. q, J = 7.0 Hz, 2H, H-12), 1.75 (app. p, J = 6.7, 2H, H-13), 1.64 (s, 1H, H-15). **^{13}C NMR** (176 MHz, $CDCl_3$): δ (ppm) 198.1, 137.4, 136.7, 131.4, 131.2, 129.8, 128.9, 127.9, 126.1, 93.5, 78.1, 62.4, 57.0, 32.4, 27.8, 26.7. $[\alpha]_D^{25.0}$ = +13.4 (c. 2.39, $CHCl_3$). **HRMS** (ESI) *m/z* Calc'd for $C_{16}H_{19}Cl_3NO_5S^-$ $[M-H]^-$: 442.0049, Found: 442.0048 (δ = - 0.2 ppm). **Chiral SFC analysis** DAICEL CHIRALPAK IG (CO_2:MeOH, 80:20, 2.5 mL min^{-1}, 40 °C) t_R = 6.48 (minor), 7.27 (major) minutes.

2,2,2-trichloroethyl (R,E)-(1-([1,1'-biphenyl]-4-yl)-6-hydroxyhex-1-en-3-yl)sulfamate (2c)

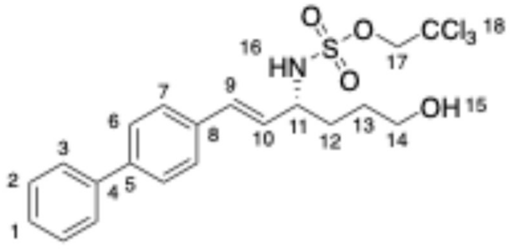

The following compound was prepared using **GP4** with **1c** as the starting material and **$Rh_2(C)_2 \cdot (4a)_2$**$\cdot(Pyr)_2$ (3.26 mg, 1.0 mol%) as the [Rh] catalyst. The reaction was performed at - 25°C. Following purification, the title compound was isolated as an off-white solid (15.3 mg, 0.032 mmol, 32%, 97% *ee*). **^{1}H NMR** (700 MHz, $CDCl_3$): δ (ppm) 7.61-7.58 (m, 2H, H-3), 7.57-7.54 (m, 2H, H-6), 7.46-7.42 (m, 4H, H-2, H-7), 7.35 (t, J = 7.4 Hz, 1H, H-1), 6.69 (d, J = 15.8 Hz, 1H, H-9), 6.17 (dd, J = 15.9, 7.2 Hz, 1H, H-10), 5.70 (d, J = 6.8 Hz, 1H, H-16), 4.63 (app. d, J = 0.8 Hz, 2H, H-17), 4.26 (app. p, J = 6.7 Hz, 1H, H-11), 3.79-3.72 (m, 2H, H-14), 1.91 (app. q, J = 7.0 Hz, 2H, H-12), 1.80-1.73 (m, 2H, H-13), 1.66 (br. s, 1H, H-15). **^{13}C NMR** (176 MHz, $CDCl_3$): δ (ppm) 140.9, 140.5, 135.0, 132.1, 128.8, 128.2, 127.5, 127.3, 127.0, 126.9, 93.5, 78.1, 62.4, 57.2, 32.5, 27.9. $[\alpha]_D^{25.0}$ = +13.0 (c. 1.09, $CHCl_3$). **HRMS** (ESI) *m/z* Calc'd for $C_{20}H_{21}Cl_3NO_4S^-$ $[M-H]^-$: 476.0257, Found: 476.0263 (δ = + 1.3 ppm). **Chiral SFC analysis** DAICEL CHIRALPAK IE (CO_2:MeOH, 85:15, 2.5 mL min^{-1}, 40 °C) t_R = 12.75 (major), 13.81 (minor) minutes.

2,2,2-trichloroethyl (R,E)-(1-(4-fluorophenyl)-6-hydroxyhex-1-en-3-yl)sulfamate (2d)

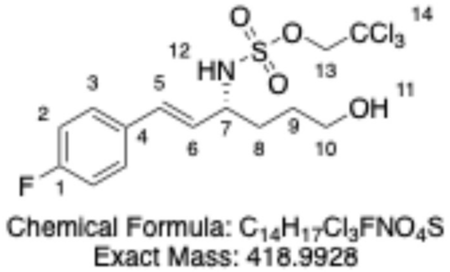

The following compound was prepared using **GP4** with **1d** as the starting material and **Rh2(C)2·(4a)2**·(Pyr)2 (3.26 mg, 1.0 mol%) as the [Rh] catalyst. Following purification, the title compound was isolated as a pale-brown oil (25.1 mg, 0.060 mmol, 60%, 94% *ee*). **^{1}H NMR** (700 MHz, $CDCl_3$): δ (ppm) 7.36-7.31 (m, 2H, H-3), 7.03-6.99 (m, 2H, H-2), 6.61 (d, J = 15.8 Hz, 1H, H-5), 6.04 (dd, J = 15.9, 7.2 Hz, 1H, H-6), 5.65 (d, J = 7.2 Hz, 1H, H-12), 4.61 (s, 2H, H-13), 4.23 (app. p, J = 7.0 Hz, 1H, H-7), 3.78-3.71 (m, 2H, H-10), 1.91-1.87 (m, 2H, H-8), 1.78-1.72 (m, 2H, H-9), 1.60-1.58 (m, 1H, H-11). **^{13}C NMR** (176 MHz, $CDCl_3$): δ (ppm) 162.6 ($^{1}J_{C-F}$, d, J = 247.6 Hz), 132.2 ($^{4}J_{C-F}$, d, J = 2.8 Hz), 131.4, 128.1 ($^{3}J_{C-F}$, d, J = 8.1 Hz), 127.9, 115.6 ($^{2}J_{C-F}$, d, J = 21.2 Hz), 93.5, 78.1, 62.4, 57.1, 32.5, 27.8. **^{19}F NMR** (376 MHz, $CDCl_3$): δ (ppm) -114.6. $[\alpha]_D^{25.0}$ = +18.2 (c. 0.55, $CHCl_3$). **HRMS** (ESI) *m/z* Calc'd for $C_4H_{16}Cl_3FNO_4S^-$ $[M-H]^-$: 417.9850, Found: 417.9859 (δ = + 2.2 ppm). **Chiral SFC analysis** DAICEL CHIRALPAK IG (CO_2:MeOH, 80:20, 2.5 mL min^{-1}, 40 °C) t_R = 3.01 (minor), 3.25 (major) minutes.

2,2,2-trichloroethyl (R,E)-(1-(4-(tert-butyl)phenyl)-6-hydroxyhex-1-en-3-yl)sulfamate (2e)

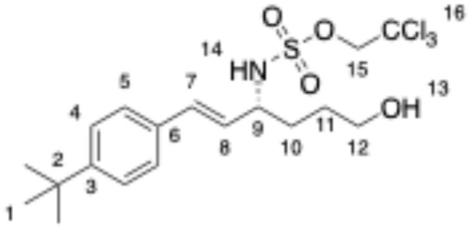

The following compound was prepared using **GP4** with **1e** as the starting material and **Rh2(C)2·(4a)2**·(Pyr)2 (3.26 mg, 1.0 mol%) as the [Rh] catalyst. Following purification, the title compound was isolated as a clear oil (28.3 mg, 0.062 mmol, 62%, 97% *ee*). **^{1}H NMR** (700 MHz, $CDCl_3$): δ (ppm) 7.35-7.32 (m, 2H, H-5), 7.31-7.29 (m, 2H, H-4), 6.62 (d, J = 15.8 Hz, 1H, H-7), 6.08 (dd, J = 15.7, 7.1 Hz, 1H, H-8), 5.68 (br. s, 1H, H-14), 4.61 (s, 2H, H-15), 4.25-

4.20 (m, 1H, H-9), 3.75-3.70 (m, 2H, H-12), 1.90-1.86 (m, 2H, H-10), 1.76-1.70 (m, 2H, H-11), 1.64 (br. s, 1H, H-13), 1.31 (s, 9H, H-1). **^{13}C NMR** (176 MHz, $CDCl_3$): δ (ppm) 151.3, 133.2, 132.2, 127.3, 126.3, 125.6, 93.5, 78.1, 62.4, 57.2, 34.6, 32.4, 31.3, 28.0. $[\alpha]_D^{25.0}$ = +14.2 (c. 0.71, $CHCl_3$). **HRMS** (ESI) *m/z* Calc'd for $C_{18}H_{25}Cl_3NO_4S^-$ $[M-H]^-$: 456.0570, Found: 456.0575 (δ = + 1.1 ppm). **Chiral SFC analysis** DAICEL CHIRALPAK IG (CO_2:MeOH, 80:20, 2.5 mL min^{-1}, 40 °C) t_R = 6.43 (minor), 8.73 (major) minutes.

(R,E)-3-(6-hydroxy-3-(((2,2,2-trichloroethoxy)sulfonyl)amino)hex-1-en-1-yl)phenyl acetate (2f)

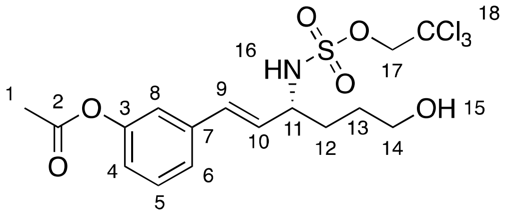

Chemical Formula: $C_{16}H_{20}Cl_3NO_6S$
Exact Mass: 459.0077

The following compound was prepared using **GP4** with **1f** as the starting material and **$Rh_2(C)_2$·(4a)$_2$**·$(Pyr)_2$ (3.26 mg, 1.0 mol%) as the [Rh] catalyst. Following purification, the title compound was isolated as a colourless oil (15.1 mg, 0.033 mmol, 33%, 86% *ee*). **^{1}H NMR** (700 MHz, $CDCl_3$): δ (ppm) 7.32 (app. t, *J* = 7.9 Hz, 1H, H-5), 7.21 (d, *J* = 7.7 Hz, 1H, H-4), 7.11 (s, 1H, H-8), 6.98 (d, *J* = 8.0 Hz, 1H, H-6), 6.62 (d, *J* = 15.9 Hz, 1H, H-9), 6.12 (dd, *J* = 15.9, 7.1 Hz, 1H, H-10), 5.88 (br. s, 1H, H-16), 4.61 (s, 2H, H-17), 4.24-4.19 (m, 1H, H-11), 3.76-3.70 (m, 2H, H-14), 2.31 (s, 3H, H-1), 1.86 (app. q, *J* = 7.0 Hz, 2H, H-13), 1.80 (br. s, 1H, H-15), 1.72 (app. p, *J* = 6.6 Hz, 2H, H-12). **^{13}C NMR** (176 MHz, $CDCl_3$): δ (ppm) 169.5, 151.0, 137.8, 131.5, 129.6, 129.4, 124.3, 121.1, 119.4, 93.5, 78.1, 62.3, 56.9, 32.3, 27.8, 21.2. $[\alpha]_D^{25.0}$ = +13.5 (c. 1.43, $CHCl_3$). **HRMS** (ESI) *m/z* Calc'd for $C_{16}H_{19}Cl_3NO_6S^-$ $[M-H]^-$: 458.0004, Found: 458.0016 (δ = + 2.6 ppm). **Chiral SFC analysis** DAICEL CHIRALPAK IG (CO_2:MeOH, 85:15, 2.5 mL min^{-1}, 40 °C) t_R = 6.52 (minor), 6.96 (major) minutes.

2,2,2-trichloroethyl (R,E)-(1-(3-(tert-butyl)phenyl)-6-hydroxyhex-1-en-3-yl)sulfamate (2g)

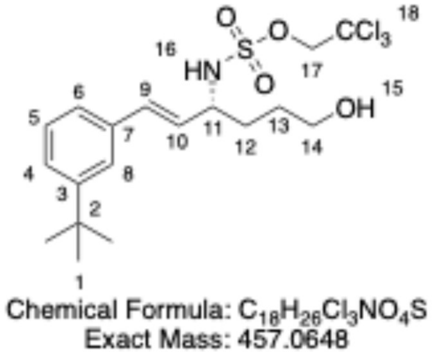

The following compound was prepared using **GP4** with **1g** as the starting material and **Rh2(C)2·(4a)2·**(Pyr)2 (3.26 mg, 1.0 mol%) as the [Rh] catalyst. Following purification, the title compound was isolated as a colourless oil (19.2 mg, 0.042 mmol, 42%, 92% *ee*). **^{1}H NMR** (700 MHz, $CDCl_3$): δ (ppm) 7.36 (s, 1H, H-8), 7.30 (d, *J* = 7.8 Hz, 1H, H-4), 7.27-7.24 (m, 1H, H-5), 7.20 (d, *J* = 7.5 Hz, 1H, H-6), 6.66 (d, *J* = 15.8 Hz, 1H, H-9), 6.12 (dd, *J* = 15.9, 7.2 Hz, 1H, H-10), 5.64 (d, *J* = 6.9 Hz, 1H, H-16), 4.62 (s, 2H, H-17), 4.24 (app. p, *J* = 6.8 Hz, 1H, H-11), 3.77-3.70 (m, 2H. H-14), 1.89 (app. q, *J* = 7.0 Hz, 2H, H-12), 1.79-1.71 (m, 2H, H-13), 1.65 (br. s, 1H, H-15), 1.32 (s, 9H, H-1). **^{13}C NMR** (176 MHz, $CDCl_3$): δ (ppm) 151.5, 135.7, 133.1, 128.4, 127.6, 125.3, 123.9, 123.5, 93.5, 78.1, 62.4, 57.2, 34.7, 32.4, 31.3, 28.0. $[\alpha]_D^{25.0}$ = +14.9 (c. 1.30, $CHCl_3$). **HRMS** (ESI) *m/z* Calc'd for $C_{18}H_{25}Cl_3NO_4S^-$ $[M-H]^-$: 456.0570, Found: 456.0575 (δ = + 1.1 ppm). **Chiral SFC analysis** DAICEL CHIRALPAK IC (CO_2:MeOH, 91:09, 2.5 mL min^{-1}, 40 °C) t_R = 16.87 (major), 17.62 (minor) minutes.

2,2,2-trichloroethyl (R,E)-(6-hydroxy-1-(3-methoxyphenyl)hex-1-en-3-yl)sulfamate (2h)

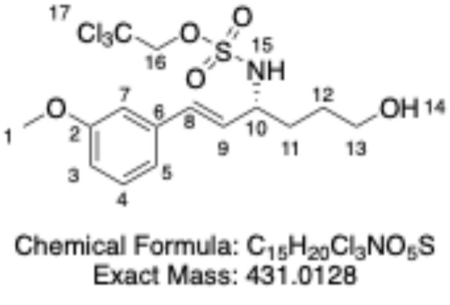

The following compound was prepared using **GP4** with **1h** as the starting material and **Rh2(C)2·(4a)2·**(Pyr)2 (3.26 mg, 1.0 mol%) as the [Rh] catalyst. Following purification, the title compound was isolated as a colourless oil (17.2 mg, 0.040 mmol, 40%, 90% *ee*). **^{1}H NMR** (700 MHz, $CDCl_3$): δ (ppm) 7.23 (app. t, *J* = 8.0 Hz, 1H, H-4), 6.96 (app. d, *J* = 7.6 Hz, 1H,

H-5), 6.90 (app. t, J = 2.0 Hz, 1H, H-7), 6.82-6.80 (m, 1H, H-3), 6.62 (d, J = 15.8 Hz, 1H, H-8), 6.12 (dd, J = 15.8, 7.2 Hz, 1H, H-9), 5.67 (d, J = 6.4 Hz, 1H, H-15), 4.61 (app. d, J = 1.0 Hz, 2H, H-16), 4.23 (app. p, J = 6.6 Hz, 1H, H-10), 3.81 (s, 3H, H-1), 3.76-3.72 (m, 2H, H-13), 1.91-1.87 (m, 2H, H-11), 1.77-1.71 (m, 2H, H-12), 1.64 (br. s, 1H, H-14). **^{13}C NMR** (176 MHz, $CDCl_3$): δ (ppm) 159.8, 137.4, 132.5, 129.6, 128.4, 119.2, 113.8, 111.9, 93.5, 78.1, 62.4, 57.1, 55.3, 32.4, 27.9. $[\alpha]_D^{25.0}$ = +16.6 (c. 1.35, $CHCl_3$). **HRMS** (ESI) *m/z* Calc'd for $C_{15}H_{19}Cl_3NO_5S^-$ [M-H]$^-$: 430.0050, Found: 430.0066 (δ = + 3.7 ppm). **Chiral SFC analysis** DAICEL CHIRALPAK IG (CO_2:MeOH, 80:20, 2.5 mL min^{-1}, 40 °C) t_R = 5.08 (minor), 6.07 (major) minutes.

Methyl (R,E)-3-(6-hydroxy-3-(((2,2,2-trichloroethoxy)sulfonyl)amino)hex-1-en-1-yl)benzoate (2i)

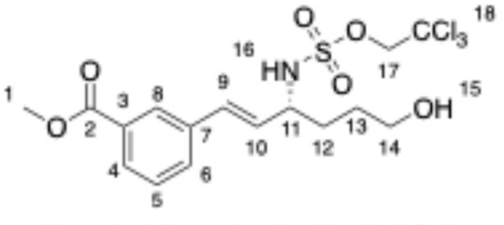

The following compound was prepared using **GP4** with **1i** as the starting material and **Rh$_2$(C)$_2$·(4a)$_2$**·(Pyr)$_2$ (3.26 mg, 1.0 mol%) as the [Rh] catalyst. Following purification, the title compound was isolated as a clear oil (21.6 mg, 0.047 mmol, 47%, 88% *ee*). **^{1}H NMR** (700 MHz, $CDCl_3$): δ (ppm) 8.05 (s, 1H, H-8), 7.91 (app. d, J = 7.7 Hz, 1H, H-4), 7.51 (d, J = 7.7 Hz, 1H, H-6), 7.38 (app. t, J = 7.7 Hz, 1H, H-5), 6.67 (d, J = 15.8 Hz, 1H, H-9), 6.22 (dd, J = 15.8, 7.2 Hz, 1H, H-10), 5.94 (d, J = 6.4 Hz, 1H, H-16), 4.62-4.59 (m, 2H, H-17), 4.25 (app. p, J = 6.8 Hz, 1H, H-11), 3.92 (s, 3H, H-1), 3.77-3.70 (m, 2H, H-14), 1.90 (app. q, J = 7.2 Hz, 2H, H-12), 1.83 (br. s, 1H, H-15), 1.76 (app. p, J = 6.5 Hz, 2H, H-13). **^{13}C NMR** (176 MHz, $CDCl_3$): δ (ppm) 166.9, 136.4, 131.4, 131.2, 130.5, 129.6, 129.0, 128.8, 127.4, 93.5, 78.1, 62.4, 57.0, 52.3, 32.4, 27.9. $[\alpha]_D^{25.0}$ = +15.8 (c. 1.80, $CHCl_3$). **HRMS** (ESI) *m/z* Calc'd for $C_{16}H_{19}Cl_3NO_6S^-$ [M-H]$^-$: 457.9999, Found: 457.9995 (δ = - 0.9 ppm). **Chiral SFC analysis** DAICEL CHIRALPAK IG (CO_2:MeOH, 80:20, 2.5 mL min^{-1}, 40 °C) t_R = 5.87 (minor), 6.68 (major) minutes.

2,2,2-trichloroethyl (R,E)-(1-(3-((tert-butoxycarbonyl)amino)phenyl)-6-hydroxyhex-1-en-3-yl)sulfamate (2j)

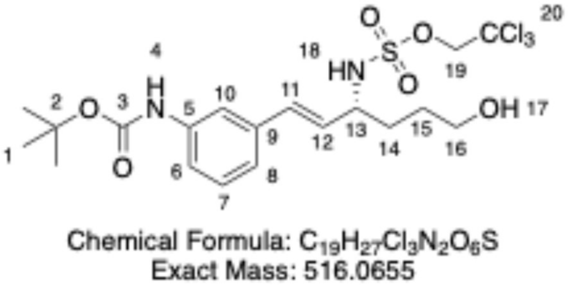

The following compound was prepared using **GP4** with **1j** as the starting material and **Rh2(C)2·(4a)2**·(Pyr)2 (3.26 mg, 1.0 mol%) as the [Rh] catalyst. Following purification, the title compound was isolated as a brown solid (25.8 mg, 0.050 mmol, 50%, 81% *ee*). **^{1}H NMR** (700 MHz, $CDCl_3$): δ (ppm) 7.54 (s, 1H, H-10), 7.22 (app. t, *J* = 7.8 Hz, 1H, H-7), 7.13 (app. dd, *J* = 8.0, 1.3 Hz, 1H, H-6), 7.02 (d, *J* = 7.7 Hz, 1H, H-8), 6.60 (d, *J* = 15.8 Hz, 1H, H-11), 6.54 (br. s, 1H, H-4), 6.12 (dd, *J* = 15.8, 7.2 Hz, 1H, H-12), 5.79 (d, *J* = 5.7 Hz, 1H, H-18), 4.61 (s, 2H, H-19), 4.20 (app. p, *J* = 6.3 Hz, 1H, H-13), 3.76-3.68 (m, 2H, H-16), 1.87-1.83 (m, 2H, H-14), 1.82-1.79 (m, 1H, H-17), 1.74-1.69 (m, 2H, H-15), 1.52 (s, 9H, H-1). **^{13}C NMR** (176 MHz, $CDCl_3$): δ (ppm) 152.8, 138.7, 137.0, 132.3, 129.2, 128.6, 121.6, 118.1, 116.3, 93.5, 78.1, 62.4, 57.1, 32.3, 28.4, 28.0, 25.6. $[\alpha]_D^{25.0}$ = +19.8 (c. 1.79, $CHCl_3$). **HRMS** (ESI) *m/z* Calc'd for $C_{19}H_{26}Cl_3N_2O_6S^-$ $[M-H]^-$: 515.0577, Found: 515.0579 (δ = + 0.4 ppm). **Chiral SFC analysis** DAICEL CHIRALPAK IG (CO_2:MeOH, 80:20, 2.5 mL min^{-1}, 40 °C) t_R = 5.57 (minor), 6.69 (major) minutes.

2,2,2-trichloroethyl (R,E)-(6-hydroxy-1-(2-methoxyphenyl)hex-1-en-3-yl)sulfamate (2k)

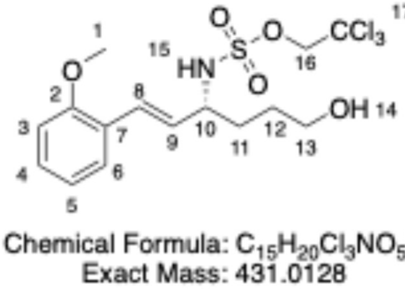

The following compound was prepared using **GP4** with **1k** as the starting material and **Rh2(C)2·(4a)2**·(Pyr)2 (3.26 mg, 1.0 mol%) as the [Rh] catalyst. Following purification, the title compound was isolated as a pale-brown oil (17.7 mg, 0.041 mmol, 41%, 87% *ee*). **^{1}H NMR** (700 MHz, $CDCl_3$): δ (ppm) 7.39 (d, *J* = 7.6 Hz, 1H, H-6), 7.25-7.22 (m, 1H, H-4), 6.96-6.90 (m, 2H, H-5, H-8), 6.86 (d, *J* = 8.3 Hz, H-3), 6.15 (dd, *J* = 16.0, 7.3 Hz, 1H, H-9), 5.59 (d, *J* =

7.1 Hz, 1H, H-15), 4.64-4.60 (m, 2H, H-16), 4.22 (app. p, *J* = 6.8 Hz, 1H, H-10), 3.84 (s, 3H, H-1), 3.75-3.71 (m, 2H, H-13), 1.88 (app. q, *J* = 7.1 Hz, 2H, H-11), 1.77-1.71(m, 2H, H-12), 1.67 (br. s, 1H, H-14). **^{13}C NMR** (176 MHz, $CDCl_3$): δ (ppm) 156.8, 129.2, 128.7, 127.6, 127.1, 125.0, 120.6, 110.9, 93.6, 78.2, 62.4, 57.6, 55.4, 32.4, 28.1. $[\alpha]_D^{25.0}$ = +11.5 (c. 1.03, $CHCl_3$). **HRMS** (ESI) *m/z* Calc'd for $C_{15}H_{19}Cl_3NO_5S^-$ [M-H]$^-$: 430.0050, Found: 430.0066 (δ = 3.7 ppm). **Chiral SFC analysis** DAICEL CHIRALPAK IG (CO_2:MeOH, 80:20, 2.5 mL min^{-1}, 40 °C) t_R = 3.45 (minor), 3.68 (major) minutes.

2,2,2-trichloroethyl (R,E)-(6-hydroxy-1-(o-tolyl)hex-1-en-3-yl)sulfamate (2l)

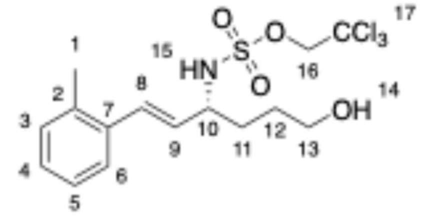

The following compound was prepared using **GP4** with **1l** as the starting material and **Rh$_2$(C)$_2$·(4a)$_2$**·(Pyr)$_2$ (3.26 mg, 1.0 mol%) as the [Rh] catalyst. The reaction was performed at - 25 °C. Following purification, the title compound was isolated as a colourless oil (8.72 mg, 0.021 mmol, 21%, 90% *ee*). **^{1}H NMR** (700 MHz, $CDCl_3$): δ (ppm) 7.26-7.24 (m, 2H, H-5, H-6), 7.13-7.11 (m, 2H, H-3, H-4), 6.61 (d, *J* = 15.8 Hz, 1H, H-8), 6.06 (dd, *J* = 15.8, 7.3 Hz, 1H, H-9), 5.56 (d, *J* = 7.3 Hz, 1H, H-15), 4.61 (s, 2H, H-16), 4.25-4.20 (m, 1H, H-10), 3.76-3.71 (m, 2H, H-13), 2.33 (s, 3H, H-1), 1.90-1.85 (m, 2H, H-11), 1.77-1.70 (m, 2H, H-12), 1.26 (s, 1H, H-14). **^{13}C NMR** (176 MHz, $CDCl_3$): δ (ppm) 138.0, 133.2, 132.6, 132.5, 129.3, 127.0, 126.6, 126.5, 93.5, 78.1, 62.4, 57.2, 32.4, 28.0, 21.2. $[\alpha]_D^{25.0}$ = +11.1 (c. 0.68, $CHCl_3$). **HRMS** (ESI) *m/z* Calc'd for $C_{15}H_{19}Cl_3NO_4S^-$ [M-H]$^-$: 414.0100, Found: 414.0108 (δ = + 1.9 ppm). **Chiral SFC analysis** DAICEL CHIRALPAK IG (CO_2:MeOH, 90:10, 2.5 mL min^{-1}, 40 °C) t_R = 8.64 (minor), 9.71 (major) minutes.

2,2,2-trichloroethyl (R,E)-(1-(2-chlorophenyl)-6-hydroxyhex-1-en-3-yl)sulfamate (2m)

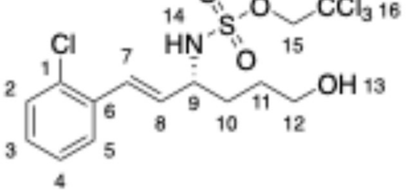

Chemical Formula: $C_{14}H_{17}Cl_4NO_4S$
Exact Mass: 434.9632

The following compound was prepared using **GP4** with **1m** as the starting material and **$Rh_2(C)_2$·(4a)$_2$**·(Pyr)$_2$ (3.26 mg, 1.0 mol%) as the [Rh] catalyst. The reaction was performed at - 25 °C. Following purification, the title compound was isolated as a colourless oil (14.8 mg, 0.034 mmol, 34%, 91% *ee*). **^{1}H NMR** (700 MHz, $CDCl_3$): δ (ppm) 7.54 – 7.50 (m, 1H, H-2), 7.40 – 7.36 (m, 1H, H-5), 7.27 – 7.19 (m, 2H, H-3, H-4), 7.04 (d, *J* = 15.9 Hz, 1H, H-7), 6.16 (dd, *J* = 15.9, 7.1 Hz, 1H, H-8), 5.80 (d, *J* = 7.4 Hz, 1H, H-14), 4.66 (d, *J* = 1.8 Hz, 2H, H-15), 4.31 (app. p, *J* = 6.8 Hz, 1H, H-9), 3.85 – 3.71 (m, 2H, H-12), 1.99 – 1.90 (m, 2H, H-10), 1.84 – 1.74 (m, 2H, H-11), 1.68 (s, 1H, H-13). **^{13}C NMR** (176 MHz, $CDCl_3$): δ (ppm) 134.4, 133.4, 131.3, 129.9, 129.2, 128.8, 127.1, 127.0, 93.6, 78.3, 62.5, 57.1, 32.5, 28.0. $[\alpha]_D^{25.0}$ = +15.7 (c. 0.43, $CHCl_3$). **HRMS** (ESI) *m/z* Calc'd for $C_{14}H_{16}Cl_4NO_4S^-$ [M-H]$^-$: 433.9554, Found: 433.9533 (δ = - 4.8 ppm). **Chiral SFC analysis** DAICEL CHIRALPAK IG (CO_2:MeOH, 80:20, 2.5 mL min^{-1}, 40 °C) t_R = 3.31 (minor), 3.64 (major) minutes.

2,2,2-trichloroethyl (R,E)-(1-(2-bromophenyl)-6-hydroxyhex-1-en-3-yl)sulfamate (2n)

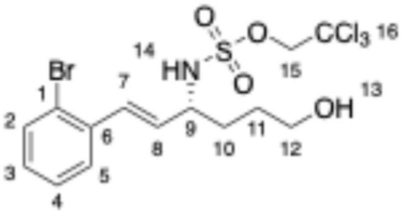

Chemical Formula: $C_{14}H_{17}BrCl_3NO_4S$
Exact Mass: 478.9127

The following compound was prepared using **GP4** with **1n** as the starting material and **$Rh_2(C)_2$·(4a)$_2$**·(Pyr)$_2$ (3.26 mg, 1.0 mol%) as the [Rh] catalyst. The reaction was performed at - 25 °C. Following purification, the title compound was isolated as a brown oil (16.8 mg, 0.035 mmol, 35%, 90% *ee*). **^{1}H NMR** (700 MHz, $CDCl_3$): δ (ppm) 7.55 (d, *J* = 8.0 Hz, 1H, H-2), 7.48 (d, *J* = 7.8 Hz, 1H, H-5), 7.28-7.25 (m, 1H, H-4), 7.12 (app. t, *J* = 7.7 Hz, H-3), 6.97 (d, *J* = 15.8 Hz, 1H, H-7), 6.09 (dd, *J* = 15.8, 7.1 Hz, 1H, H-8), 5.81 (d, *J* = 6.7 Hz, 1H, H-14), 4.67-

4.62 (m, 2H, H-15), 4.28 (app. p, *J* = 6.7 Hz, 1H, H-9), 3.80-3.73 (m, 2H, H-12), 1.92 (app. q, *J* = 7.1 Hz, 2H, H-10), 1.80-1.73 (m, 2H, H-11), 1.68 (br. s, 1H, H-13). ^{13}**C NMR** (176 MHz, $CDCl_3$): δ (ppm) 136.1, 133.0, 131.4, 131.2, 129.3, 127.6, 127.2, 123.7, 93.5, 78.2, 62.4, 56.8, 32.3, 27.8. $[\alpha]_D^{25.0}$ = +13.0 (c. 0.98, $CHCl_3$). **HRMS** (ESI) *m/z* Calc'd for $C_{14}H_{16}BrCl_3NO_4S^-$ $[M-H]^-$: 477.9049, Found: 477.9069 (δ = 2.1 ppm). **Chiral SFC analysis** DAICEL CHIRALPAK IG (CO_2:MeOH, 80:20, 2.5 mL min^{-1}, 40 °C) t_R = 3.17 (minor), 3.47 (major) minutes.

2,2,2-trichloroethyl (R,E)-(1-(2-ethylphenyl)-6-hydroxyhex-1-en-3-yl)sulfamate (2o)

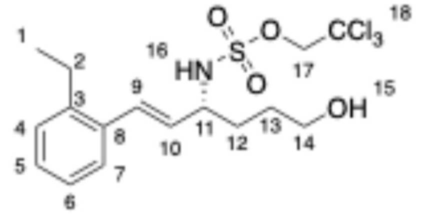

The following compound was prepared using **GP4** with **1o** as the starting material and **$Rh_2(C)_2\cdot(4a)_2$**$\cdot(Pyr)_2$ (3.26 mg, 1.0 mol%) as the [Rh] catalyst. Following purification, the title compound was isolated as a yellow oil (14.2 mg, 0.033 mmol, 33%, 92% *ee*). ^{1}**H NMR** (700 MHz, $CDCl_3$): δ (ppm) 7.41 (d, *J* = 7.7 Hz, 1H, H-7), 7.23-7.20 (m, 1H, H-6), 7.18-7.14 (m, 2H, H-4, H-5), 6.91 (d, *J* = 15.7 Hz, 1H, H-9), 6.00 (dd, *J* = 15.6, 7.4 Hz, 1H, H-10), 5.64 (d, *J* = 6.8 Hz, 1H, H-16), 4.63-4.60 (m, 2H, H-17), 4.28-4.23 (m, 1H, H-11), 3.78-3.72 (m, 2H, H-14), 2.70 (q, *J* = 7.6 Hz, 1H, H-2), 1.93-1.88 (m, 2H, H-12), 1.80-1.71 (m, 2H, H-13), 1.60 (br. s, 1H, H-15). 1.20 (t, *J* = 7.6 Hz, 3H, H-1). ^{13}**C NMR** (176 MHz, $CDCl_3$): δ (ppm) 141.8, 134.6, 130.4, 129.6, 128.8, 128.2, 126.1, 126.0, 93.5, 78.1, 62.4, 57.4, 32.5, 27.9, 26.3, 15.3. $[\alpha]_D^{25.0}$ = +14.7 (c. 0.57, $CHCl_3$). **HRMS** (ESI) *m/z* Calc'd for $C_{16}H_{21}Cl_3NO_4S^-$ $[M-H]^-$: 428.0256, Found: 428.0235 (δ = - 4.9 ppm). **Chiral SFC analysis** DAICEL CHIRALPAK IJ (CO_2:MeOH, 92:08, 2.5 mL min^{-1}, 40 °C) t_R = 7.34 (major), 7.70 (minor) minutes.

2,2,2-trichloroethyl (R,E)-(6-hydroxy-1-mesitylhex-1-en-3-yl)sulfamate (2p)

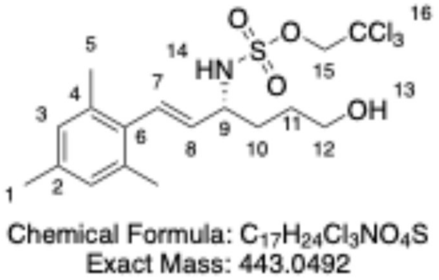

The following compound was prepared using **GP4** with **1p** as the starting material and **$Rh_2(C)_2\cdot(4a)_2$**$\cdot(Pyr)_2$ (3.26 mg, 1.0 mol%) as the [Rh] catalyst. Following purification, the title compound was isolated as a brown oil (13.3 mg, 0.030 mmol, 30%, 90% *ee*). **^{1}H NMR** (700 MHz, $CDCl_3$): δ (ppm) 6.85 (s, 2H, H-3), 6.61 (d, *J* = 16.1 Hz, 1H, H-7), 5.61 (dd, *J* = 16.1, 7.1 Hz, 1H, H-8), 4.63 (s, 2H, H-15), 4.28-4.23 (m, 1H, H-9), 3.79-3.72 (m, 2H, H-12), 2.27-2.25 (m, 9H, H-1, H-5), 1.92-1.87 (m, 2H, H-10), 1.82-1.72 (m, 2H, H-11). **^{13}C NMR** (176 MHz, $CDCl_3$): δ (ppm) 136.6, 135.8, 133.0, 132.8, 130.4, 128.6, 93.6, 78.1, 62.4, 57.5, 32.6, 30.9, 27.9, 20.9. $[\alpha]_D^{25.0}$ = + 24.0 (c. 1.21, $CHCl_3$). **HRMS** (ESI) *m/z* Calc'd for $C_{17}H_{23}Cl_3NO_4S^-$ $[M-H]^-$: 442.0413, Found: 442.0434 (δ = + 4.8 ppm). **Chiral SFC analysis** DAICEL CHIRALPAK IG (CO_2:MeOH, 80:20, 2.5 mL min^{-1}, 40 °C) t_R = 3.00 (minor), 3.56 (major) minutes.

2,2,2-trichloroethyl (R,E)-(6-hydroxy-6-methyl-1-phenylhept-1-en-3-yl)sulfamate (2q)

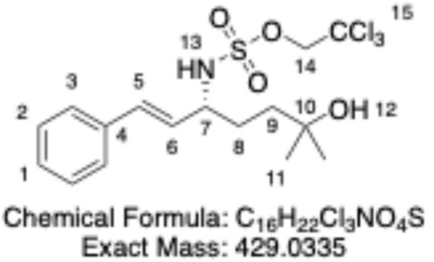

The following compound was prepared using **GP4** with **1q** as the starting material and **$Rh_2(C)_2\cdot(4a)_2$**$\cdot(Pyr)_2$ (3.26 mg, 1.0 mol%) as the [Rh] catalyst. Following purification, the title compound was isolated as a white solid (12.9 mg, 0.030 mmol, 30%, 84% *ee*). **^{1}H NMR** (700 MHz, $CDCl_3$): δ (ppm) 7.37-7.36 (m, 2H, H-3), 7.33-7.30 (m, 2H, H-2), 7.27-7.24 (m, 1H, H-1), 6.64 (d, *J* = 15.8 Hz, 1H, H-5), 6.13 (dd, *J* = 15.9, 7.2 Hz, 1H, H-6), 5.75 (d, *J* = 7.1 Hz, 1H, H-13), 4.61 (s, 2H, H-14), 4.21-4.16 (m, 1H, H-7), 1.94-1.82 (m, 2H, H-8), 1.65 (app. t, *J* = 7.7 Hz, 2H, H-9), 1.60 (br. s, 1H, H-12), 1.28 (s, 3H, H-11a), 1.26 (s, 3H, H-11b). **^{13}C NMR**

(176 MHz, $CDCl_3$): δ (ppm) 136.1, 132.5, 128.6, 128.3, 128.1, 126.6, 93.5, 78.1, 70.9, 57.7, 38.6, 30.0, 29.8. $[\alpha]_D^{25.0}$ = +20.2 (c. 1.18, $CHCl_3$). **HRMS** (ESI) *m/z* Calc'd for $C_{16}H_{21}Cl_3NO_4S^-$ $[M-H]^-$: 428.0257, Found: 428.0257 (δ = 0.0 ppm). **Chiral SFC analysis** DAICEL CHIRALPAK IC (CO_2:MeOH, 90:10, 2.5 mL min^{-1}, 40 °C) t_R = 4.88 (major), 5.17 (minor) minutes.

2,2,2-trichloroethyl (R,E)-(1-hydroxydec-5-en-4-yl)sulfamate (2t)

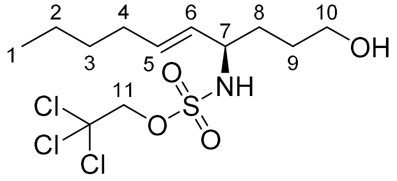

Chemical Formula: $C_{12}H_{22}Cl_3NO_4S$
Exact Mass: 381.0335

The following compound was prepared using **GP4** with **1t** as the starting material and **$Rh_2(C)_2$·(4a)$_2$**·$(Pyr)_2$ (3.26 mg, 1.0 mol%) as the [Rh] catalyst. Following purification, the title compound was isolated as a colourless oil (6.8 mg, 0.018 mmol, 18%, 75% *ee*). **^{1}H NMR** (400 MHz, $CDCl_3$): δ 5.73 (dt, *J* = 14.3, 6.7 Hz, 1H, H-5), 5.46 – 5.24 (m, 2H, H-6, NH), 4.60 (s, 2H, H-11), 4.00 (t, *J* = 6.8 Hz, 1H, H-7), 3.70 (t, *J* = 5.9 Hz, 2H, H-10), 2.04 (q, *J* = 7.1 Hz, 2H, H-4), 1.79 – 1.63 (m, 4H, H-8, H-9), 1.40 – 1.24 (m, 4H, H-2, H-3), 0.89 (t, *J* = 7.0 Hz, 3H, H-1). **^{13}C NMR** (101 MHz, $CDCl_3$) δ 134.5, 128.8, 93.7, 78.2, 62.5, 57.3, 32.5, 32.0, 31.3, 28.2, 22.4, 14.0. $[\alpha]_D^{25.0}$ = +2.33 (c. 0.34, $CHCl_3$). HRMS (ESI) m/z Calc'd for $C_{12}H_{21}Cl_3NO_4S^-$ $[M-H]^-$: 380.0262, Found: 380.0258 (δ = -1.2 ppm). Chiral HPLC analysis CHIRALPAK IH (Hexane: ^{i}PrOH, 92:08, 1.25 mL min^{-1}, 40 °C) t_R = 12.79 (major), 15.23 (minor) minutes. ***Note: 4 mol% $Rh_2(A)_2$·(Bu_4N_2) was required to access racemate.***

2,2,2-trichloroethyl (R,E)-(6-methoxy-1-phenylhex-1-en-3-yl)sulfamate (10)

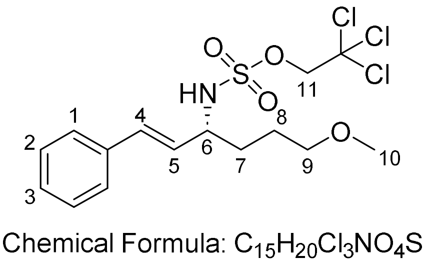

Chemical Formula: $C_{15}H_{20}Cl_3NO_4S$
Exact Mass: 415.0179

The following compound was prepared using **GP4** with ***(E)-(6-methoxyhex-1-en-1-yl)benzene*** as the starting material and **$Rh_2(C)_2$·(4a)$_2$**·$(Pyr)_2$ (3.26 mg, 1.0 mol%) as the [Rh] catalyst. Following purification, the title compound was isolated as a colourless oil (2.4 mg, 0.006 mmol, 6%, 81% *ee*). **^{1}H NMR** (700 MHz, $CDCl_3$): δ 7.39 (d, *J* = 7.6 Hz, 2H, H-1), 7.34 (t, *J* = 7.6 Hz, 2H, H-2), 7.28 – 7.25 (m, 1H, H-3), 6.66 (d, *J* = 15.9 Hz, 1H, H-4), 6.14 (dd, *J* = 15.9, 7.1 Hz, 1H, H-5), 6.03 (d, *J* = 7.1 Hz, 1H, NH), 4.65 – 4.61 (m, 2H, H-11), 4.24 (t, *J* = 6.7 Hz, 1H, H-6), 3.47 (t, *J* = 5.6 Hz, 2H, H-9), 3.41 (s, 3H, H-10), 1.96 – 1.85 (m, 2H, H-7), 1.83 – 1.72 (m, 2H, H-8). **^{13}C NMR** (176 MHz, $CDCl_3$): δ 136.3, 132.5, 128.8, 128.4, 128.1, 126.7, 93.7, 78.2, 72.5, 58.9, 57.0, 33.1, 25.4. $[\boldsymbol{\alpha}]_D^{25.0}$ = + 25.8 (c. 0.18, $CHCl_3$). HRMS (ESI) m/z Calc'd for $C_{15}H_{19}Cl_3NO_4S^-$ $[M-H]^-$: 414.0106, Found: 414.0108 (δ = 0.6 ppm). Chiral HPLC analysis CHIRALPAK IH (Hexane: ^{i}PrOH, 90:10, 1.25 mL min^{-1}, 40 °C) t_R = 12.12 (major), 13.23 (minor) minutes. ***Note: 4 mol% $Rh_2(A)_2$·(4f) was required to access opposite enantiomer.***

2,2,2-trichloroethyl (R,E)-(6-oxo-1-phenyl-6-(phenylamino)hex-1-en-3-yl)sulfamate (11)

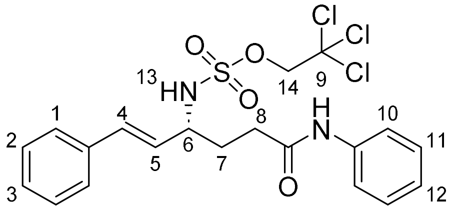

Chemical Formula: $C_{20}H_{21}Cl_3N_2O_4S$
Exact Mass: 490.0288

The following compound was prepared using **GP4** with ***(E)-N,6-diphenylhex-5-enamide*** as the starting material and **$Rh_2(C)_2$·(4a)$_2$**·$(Pyr)_2$ (3.26 mg, 1.0 mol%) as the [Rh] catalyst. Following purification, the title compound was isolated as a colourless oil (3.4 mg, 0.007 mmol, 7%, 44% *ee*). **^{1}H NMR** (700 MHz, $CDCl_3$): δ 7.48 (d, *J* = 8.0 Hz, 2H, H-2), 7.39 – 7.28 (m, 8H, H-1, H-10, H-11, H-9), 7.27 – 7.23 (m, 1H, H-12), 7.13 (t, *J* = 7.4 Hz, 1H, H-3), 6.68 (d, *J* = 15.9 Hz, 1H, H-4), 6.32 (t, *J* = 8.6 Hz, 1H, H-13), 6.15 (dd, *J* = 15.9, 7.0 Hz, 1H, H-5), 4.61 (s, 2H, H-14), 4.30 – 4.23 (m, 1H, H-6), 2.69 – 2.54 (m, 2H, H-8), 2.23 – 2.06 (m, 2H, H-7). **^{13}C NMR** (176 MHz, $CDCl_3$): δ 171.3, 137.4, 136.0, 132.9, 129.2, 128.8, 128.3, 127.8, 126.8, 124.9, 120.4, 93.6, 78.2, 57.3, 33.6, 30.4. $[\boldsymbol{\alpha}]_D^{25.0}$ = +15.9 (c. 0.26, $CHCl_3$). HRMS (ESI) m/z Calc'd for $C_{20}H_{20}Cl_3N_2O_4S^-$ $[M-H]^-$: 489.0215, Found: 489.0204 (δ = -2.2 ppm). Chiral SFC analysis DAICEL CHIRALPAK IG (CO_2:MeOH, 75:25, 2.50 mL min^{-1}, 40 °C) t_R = 7.67 (major), 11.65 (minor) minutes.

5.2 Substrate scope (5-C Chain length)

2,2,2-trichloroethyl (R,E)-(5-hydroxy-1-phenylpent-1-en-3-yl)sulfamate (6a)

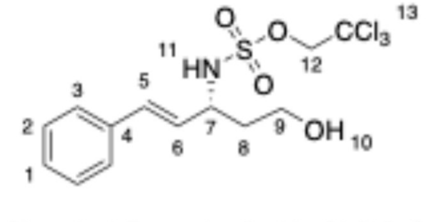

The following compound was prepared using **GP4** with **5a** as the starting material and **Rh$_2$(E)$_2$·(4a)$_2$·**(Pyr)$_2$ (3.36 mg, 1.0 mol%) as the [Rh] catalyst. Following purification, the title compound was isolated as a colourless oil (18.6 mg, 0.048 mmol, 48%, 93% *ee*). **^{1}H NMR** (700 MHz, $CDCl_3$): δ (ppm) 7.41-7.36 (m, 2H, H-3), 7.36-7.30 (m, 2H, H-2), 7.28-7.24 (m, 1H, H-1), 6.69 (d, *J* = 15.8 Hz, 1H, H-5), 6.19 (dd, *J* = 15.9, 7.0 Hz, 1H, H-6), 5.92 (d, *J* = 6.9 Hz, 1H, H-11), 4.63 (s, 2H, H-12), 4.48-4.43 (m, 1H, H-7), 4.00-3.95 (m, 1H, H-9a), 3.91-3.86 (m, 1H, H-9b), 2.13-2.09 (m, 1H, H-8a), 1.93-1.83 (m, 2H, H-8b, H-10). **^{13}C NMR** (176 MHz, $CDCl_3$): δ (ppm) 136.0, 132.5, 128.7, 128.1, 127.5, 126.6, 93.5, 78.2, 59.8, 56.0, 36.5. $[\boldsymbol{\alpha}]_{\mathrm{D}}^{\mathbf{25.0}}$ = +22.0 (c. 1.21, $CHCl_3$). **HRMS** (ESI) *m/z* Calc'd for $C_{13}H_{15}Cl_3NO_4S^-$ $[M-H]^-$: 385.9787, Found: 385.9775 (δ = - 3.1 ppm). **Chiral SFC analysis** DAICEL CHIRALPAK IG (CO_2:MeOH, 80:20, 2.5 mL min^{-1}, 40 °C) $\boldsymbol{t_R}$ = 4.17 (minor), 6.54 (major) minutes.

2,2,2-trichloroethyl (R,E)-(5-hydroxy-1-(2-isopropylphenyl)pent-1-en-3-yl)sulfamate (6b)

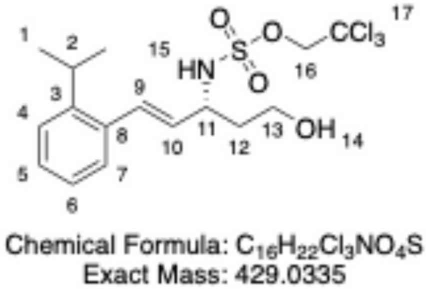

The following compound was prepared using **GP4** with **5b** as the starting material. **Rh$_2$(E)$_2$·(4a)$_2$·**(Pyr)$_2$ (3.36 mg, 1.0 mol%) was used as the ion-paired rhodium complex in the transformation following Condition B. Following purification, the title compound was isolated as a colourless oil (34.3 mg, 0.080 mmol, 80%, 88% *ee*). **^{1}H NMR** (700 MHz, $CDCl_3$): δ (ppm)

7.38 (d, J = 7.8 Hz, 1H, H-7), 7.27-7.25 (m, 2H, H-4, H-5), 7.17-7.13 (m, 1H, H-6), 7.05 (d, J = 15.5 Hz, 1H, H-9), 6.02 (dd, J = 15.6, 7.1 Hz, 1H, H-10), 5.90 (br. s, 1H, H-15), 4.64 (s, 2H, H-16), 4.50-4.45 (m, 1H, H-11), 4.02-3.97 (m, 1H, H-13a), 3.93-3.87 (m, 1H, H-13b), 3.22 (hep, J = 6.9 Hz, 1H, H-2), 2.15-2.08 (m, 1H, H-12a), 1.94-1.87 (m, 1H, H-12b), 1.81 (br. s, 1H, H-14), 1.25-1.22 (m, 6H, H-1). **^{13}C NMR** (176 MHz, $CDCl_3$): δ (ppm) 146.1, 134.4, 130.5, 129.6, 128.3, 126.5, 125.9, 125.1, 93.5, 78.2, 59.9, 56.3, 36.6, 29.2, 23.42, 23.39. $[\alpha]_D^{25.0}$ = +12.0 (c. 1.35, $CHCl_3$). **HRMS** (ESI) *m/z* Calc'd for $C_{16}H_{21}Cl_3NO_4S^-$ $[M-H]^-$: 428.0257, Found: 428.0236 (δ = - 4.9 ppm). **Chiral SFC analysis** DAICEL CHIRALPAK IJ (CO_2:MeOH, 92:08, 2.5 mL min^{-1}, 40 °C) t_R = 5.32 (major), 5.55 (minor) minutes.

2,2,2-trichloroethyl (R,E)-(5-hydroxy-1-(3-methoxyphenyl)pent-1-en-3-yl)sulfamate (6c)

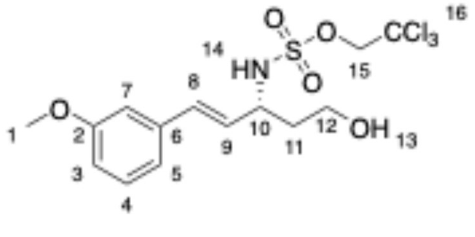

The following compound was prepared using **GP4** with **5c** as the starting material. **$Rh_2(E)_2·(4a)_2$**·$(Pyr)_2$ (3.36 mg, 1.0 mol%) was used as the ion-paired rhodium complex in the transformation following Condition B. Following purification, the title compound was isolated as a pale-brown oil (20.0 mg, 0.048 mmol, 48%, 85% *ee*). **^{1}H NMR** (700 MHz, $CDCl_3$): δ (ppm) 7.23 (app. t, J = 7.9 Hz, 1H, H-4), 6.97 (d, J = 7.6 Hz, 1H, H-5), 6.90 (app. t, J = 2.0 Hz, 1H, H-7), 6.83-6.80 (m, 1H, H-3), 6.67 (d, J = 15.9 Hz, 1H, H-8), 6.18 (dd, J = 15.8, 1.4 Hz, 1H, H-9), 5.87 (d, J = 7.1 Hz, 1H, H-14), 4.63 (s, 2H, H-15), 4.48-4.43 (m, 1H, H-10), 4.06-3.96 (m, 1H, H-12a), 3.91-3.87 (m, 1H, H-12b), 3.81 (s, 3H, H-1), 2.14-2.09 (m, 1H, H-11a), 1.92-1.86 (m, 1H, H-11b). **^{13}C NMR** (176 MHz, $CDCl_3$): δ (ppm) 159.8, 137.4, 132.4, 129.6, 127.8, 119.2, 113.8, 112.0, 93.5, 78.1, 59.8, 55.9, 55.3, 36.5. $[\alpha]_D^{25.0}$ = +18.0 (c. 1.51, $CHCl_3$). **HRMS** (ESI) *m/z* Calc'd for $C_{14}H_{17}Cl_3NO_5S^-$ $[M-H]^-$: 415.9865, Found: 415.9876 (δ = - 4.1 ppm). **Chiral SFC analysis** DAICEL CHIRALPAK IJ (CO_2:MeOH, 90:10, 2.5 mL min^{-1}, 40 °C) t_R = 8.07 (minor), 8.93 (major) minutes.

2,2,2-trichloroethyl (R,E)-(1-(3,5-di-tert-butylphenyl)-5-hydroxypent-1-en-3-yl)sulfamate (6d)

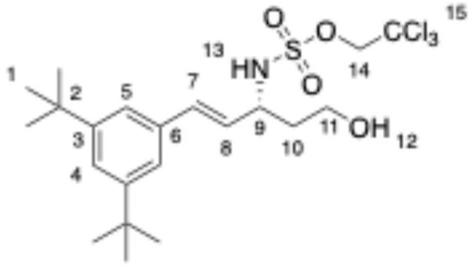

The following compound was prepared using **GP4** with **5d** as the starting material. **$Rh_2(E)_2$·$(4a)_2$**·$(Pyr)_2$ (3.36 mg, 1.0 mol%) was used as the ion-paired rhodium complex in the transformation following Condition B. Following purification, the title compound was isolated as a white fluffy solid (25.0 mg, 0.050 mmol, 50%, 90% *ee*). **^{1}H NMR** (700 MHz, $CDCl_3$): δ (ppm) 7.35-7.33 (m, 1H, H-4), 7.22-7.20 (m, 2H, H-5), 6.71 (d, *J* = 15.8 Hz, 1H, H-7), 6.17 (dd, *J* = 15.9, 6.9 Hz, 1H, H-8), 5.85 (d, *J* = 6.7 Hz, 1H, H-13), 4.66-4.62 (m, 2H, H-14), 4.50-4.43 (m, 1H, H-9), 4.01-3.95 (m, 1H, H-11a), 3.90-3.87 (m, 1H, H-11b), 2.16-2.08 (m, 1H, H-10a), 1.94-1.89 (m, 1H, H-10b), 1.86 (br. s, 1H, H-12), 1.32 (s, 18H, H-1). **^{13}C NMR** (176 MHz, $CDCl_3$): δ (ppm) 151.1, 135.2, 133.6, 126.6, 122.5, 120.9, 94.5, 78.2, 59.7, 55.9, 36.6, 34.8, 31.4. $[\alpha]_D^{25.0}$ = +45.2 (c. 2.11, $CHCl_3$). **HRMS** (ESI) *m/z* Calc'd for $C_{21}H_{31}Cl_3NO_4S^-$ [M-H]$^-$: 498.1039, Found: 498.1042 (δ = + 0.6 ppm). **Chiral SFC analysis** DAICEL CHIRALPAK IG (CO_2:MeOH, 90:10, 1.25 mL min^{-1}, 40 °C) $\boldsymbol{t_R}$ = 6.97 (major), 7.46 (minor) minutes.

Methyl (R,E)-2-(5-hydroxy-3-(((2,2,2-trichloroethoxy)sulfonyl)amino)pent-1-en-1-yl)benzoate (6e)

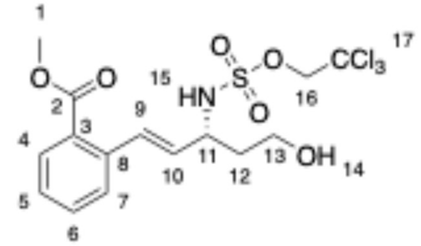

The following compound was prepared using **GP4** with **5e** as the starting material. **$Rh_2(C)_2$·$(4a)_2$**·$(Pyr)_2$ (3.26 mg, 1.0 mol%) was used as the ion-paired rhodium complex in the transformation. Following purification, the title compound was isolated as a colourless oil (23.1 mg, 0.052 mmol, 52%, 94% *ee*). **^{1}H NMR** (700 MHz, $CDCl_3$): δ (ppm) 7.92-7.90 (m, 1H, H-

4), 7.51-7.47 (m, 2H, H-6, H-7), 7.41-7.38 (m, 1H, H-9), 7.35-7.31 (m, 1H, H-5), 6.16 (br. s, 1H, H-15), 6.02 (dd, J = 15.8, 6.4 Hz, 1H, H-10), 4.68 (d, J = 10.9 Hz, 1H, H-16a), 4.65 (d, J = 10.9 Hz, 1H, H-16b), 4.49 (app. q, J = 5.8 Hz, 1H, H-11), 4.00-3.95 (m, 1H, H-13a), 3.90 (s, 3H, H-1), 3.88-3.84 (m, 1H, H-13b), 2.50 (br. s, 1H, H-14), 2.15-2.10 (m, 1H, H-12a), 1.95-1.89 (m, 1H, H-12b). **^{13}C NMR** (176 MHz, $CDCl_3$): δ (ppm) 167.7, 138.8, 132.5, 131.6, 130.6, 130.2, 128.2, 127.9, 127.6, 93.6, 78.2, 59.6, 55.6, 52.3, 36.1. $[\alpha]_D^{25.0}$ = +10.8 (c. 1.80, $CHCl_3$). **HRMS** (ESI) *m/z* Calc'd for $C_{15}H_{17}Cl_3NO_6S^-$ [M-H]$^-$: 443.9842, Found: 443.9833 (δ = - 2.0 ppm). **Chiral SFC analysis** DAICEL CHIRALPAK IG (CO_2:MeOH, 80:20, 2.5 mL min^{-1}, 40 °C) t_R = 4.19 (major), 5.51 (minor) minutes.

2,2,2-trichloroethyl (R,E)-(5-hydroxy-5-methyl-1-phenylhex-1-en-3-yl)sulfamate (6f)

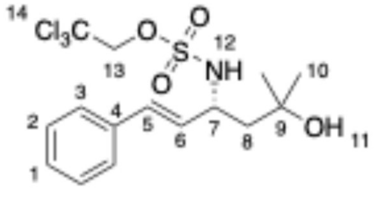

Chemical Formula: $C_{15}H_{20}Cl_3NO_4S$
Exact Mass: 415.0179

The following compound was prepared using **GP4** with **5f** as the starting material. **$Rh_2(E)_2$·(4a)$_2$**·(Pyr)$_2$ (3.36 mg, 1.0 mol%) was used as the ion-paired rhodium complex in the transformation . Following purification, the title compound was isolated as a white solid (21.2 mg, 0.051 mmol, 51%, 90% *ee*). **^{1}H NMR** (700 MHz, $CDCl_3$): δ (ppm) 7.36-7.35 (m, 2H, H-3), 7.31-7.28 (m, 2H, H-2), 7.25-7.22 (m, 1H, H-1), 6.94 (br. s, 1H, H-12), 6.63 (d, J = 15.8 Hz, 1H, H-5), 6.13 (dd, J = 15.8, 8.0 Hz, 1H, H-6), 4.59 (s, 2H, H-13), 4.45-4.41 (m, 1H, H-7), 1.95 (dd, J = 14.9, 10.8 Hz, 1H, H-8a), 1.72 (dd, J = 14.9, 3.5 Hz, 1H, H-8b), 1.66 (br. s, 1H, H-11), 1.44 (s, 3H, H-10a), 1.34 (s, 3H, H-10b). **^{13}C NMR** (176 MHz, $CDCl_3$): δ (ppm) 136.2, 132.2, 128.9, 128.6, 128.0, 126.6, 93.5, 78.0, 72.3, 55.6, 45.7, 32.6, 27.8. $[\alpha]_D^{25.0}$ = +11.3 (c. 0.38, $CHCl_3$). **HRMS** (ESI) *m/z* Calc'd for $C_{15}H_{19}Cl_3NO_4S^-$ [M-H]$^-$: 414.0100, Found: 414.0120 (δ = + 4.8 ppm). **Chiral SFC analysis** DAICEL CHIRALPAK IG (CO_2:MeOH, 80:20, 2.5 mL min^{-1}, 40 °C) t_R = 3.02 (minor), 3.43 (major) minutes.

6. Catalyst and Additive Optimisation

6.1 For 6-C Trans alkenyl alcohol substrate

*Table S1 - Summary of yields and ee values for **2a** using various catalyst loadings*

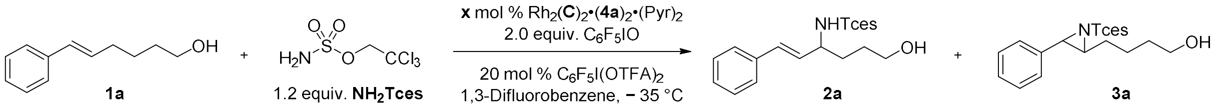

Entry	Catalyst loading / %	Yield of 2a / %[a]	2a:3a ratio[a]	*ee* of 2a / %[b]
**1**	1.0	49%	9 : 1	93%
**2**	2.0	43%	10 : 1	93%

[a]Reactions performed according to **GP4**, on a 0.1 mmol scale with respect to **1a**. Yields and ratios refer to NMR yields of **2a** and **3a**, respectively, using 1,3,5-Trimethoxybenzene as an internal standard. [b]*ee* determined by chiral SFC analysis.

*Table S2 - Summary of yields and ee values for **2a** using various additive loadings*

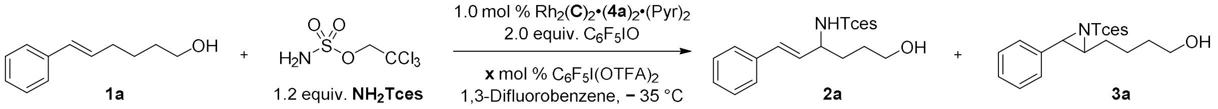

Entry	Additive loading / %	Yield of 2a / %[a]	2a:3a ratio[a]	*ee* of 2a / %[b]
**1**	0	20%	3 : 1	74%
**2**	5	25%	8 : 1	94%
**3**	10	36%	8 : 1	92%
**4**	15	40%	8.5 : 1	92%
**5**	20	49%	9 : 1	93%
**6**	30	15%	3.7 : 1	78%
**7**	40	8%	3 : 1	74%
**8**	50	9%	2.8 : 1	82%

[a]Reactions performed according to **GP4**, on a 0.1 mmol scale with respect to **1a**. Yields and ratios refer to NMR yields of **2a** and **3a**, respectively, using 1,3,5-Trimethoxybenzene as an internal standard. [b]*ee* determined by chiral SFC analysis.

*Table S3 - Summary of yields and ee values for **2a** using various additives*

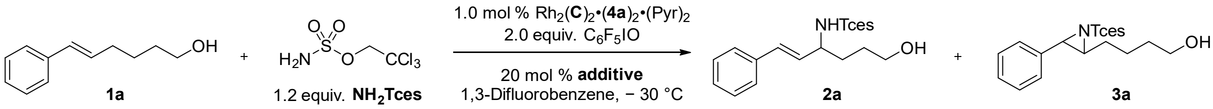

Entry	Additive	Yield of 2a / %[a]	2a:3a ratio[a]	*ee* of 2a / %[b]
**1**	$C_6H_5I(OAc)_2$	36%	7.2 : 1	91%
**2**	$C_6H_5I(OTFA)_2$	44%	8.8 : 1	92%
**3**	$C_6H_5I(OH)(OTs)$	22%	3.1 : 1	78%

[a]Reactions performed according to **GP4**, on a 0.1 mmol scale with respect to **1a**. Yields and ratios refer to NMR yields of **2a** and **3a**, respectively, using 1,3,5-Trimethoxybenzene as an internal standard. [b]*ee* determined by chiral SFC analysis.

2,2,3,3,3-pentafluoropropyl (R,E)-(6-hydroxy-1-phenylhex-1-en-3-yl)sulfamate (2a-pfps)

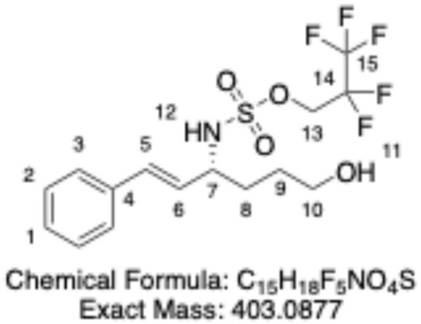

This example refers to entry 4 from Table 1 and the below values are isolated, rather than taken from crude NMR with internal standard (42%) and SFC analysis of crude reaction mixture (88% ee). The compound was prepared using **GP4** with **1a** as the starting material and $PfpsNH_2$ (0.12 mmol) as the sulfamate ester instead of $TcesNH_2$. **$Rh_2(C)_2·(4a)_2$**$·(Pyr)_2$ (3.26 mg, 1.0 mol%) was used as the [Rh] catalyst. The chemoselectivity ratio was determined to be **2.9**:**1** **allylic amine:aziridine** ratio from analysis of the reaction mixture with ^{1}H NMR. Following purification, the title compound was isolated as a colourless oil (12.9 mg, 0.032 mmol, 32%, 90% *ee*). **^{1}H NMR** (700 MHz, $CDCl_3$): δ (ppm) 7.36 (d, *J* = 7.9 Hz, 2H, H-3), 7.32 (t, *J* = 7.5 Hz, 2H, H-2), 7.28-7.26 (m, 1H, H-1), 6.62 (d, *J* = 15.9 Hz, 1H, H-5), 6.08 (dd, *J* = 15.9 Hz,

1H, H-6), 5.72 (br. s, 1H, H-12), 4.45 (t, J = 12.4 Hz, 2H, H-13), 4.21-4.14 (m, 1H, H-7), 3.77-3.70 (m, 2H, H-10), 1.87 (app. q, J = 6.9 Hz, 2H, H-8), 1.77-1.68 (m, 2H, H-9), 1.62 (br. s, 1H, H-11). **^{13}C NMR** (176 MHz, $CDCl_3$): δ (ppm) 135.9, 132.6, 128.7, 128.1, 127.8, 126.5, 118.3 (m),* 111.3 (m),* 63.9 (t, $J_{C\text{-}F}$ = 28.2 Hz), 62.4, 57.1, 32.4, 27.7. **^{19}F NMR** (376 MHz, $CDCl_3$): δ (ppm) -84.5, -124.4. $[\alpha]_D^{25.0}$ = +22.0 (c. 0.72, $CHCl_3$). **HRMS** (ESI) *m/z* Calc'd for $C_{15}H_{17}F_5NO_4S^-$ [M-H]$^-$: 402.0798, Found: 402.0816 (δ = + 4.5 ppm). **Chiral SFC analysis** DAICEL CHIRALPAK IG (CO_2:MeOH, 95:5, 2.5 mL min^{-1}, 40 °C) t_R = 5.91 (minor), 6.25 (major) minutes.

*Assigned with (^{1}H)-(^{13}C) HMBC.

2,2,3,3,3-pentafluoropropyl (R,E)-(1-(3-acetylphenyl)-6-hydroxyhex-1-en-3-yl)sulfamate (2b-pfps)

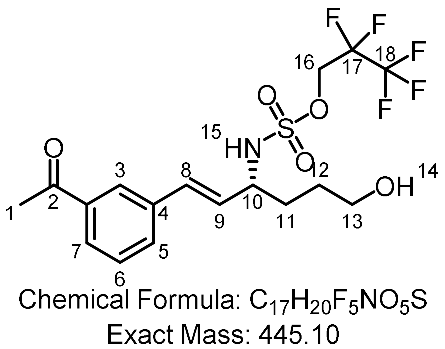

Chemical Formula: $C_{17}H_{20}F_5NO_5S$
Exact Mass: 445.10

This example refers to entry 8 from Table 1 and the below values are isolated, rather than taken from crude NMR with internal standard (55%) and SFC analysis of crude reaction mixture (77% ee). The compound was prepared using **GP4** with **1b** as the starting material and $PfpsNH_2$ (0.12 mmol) as the sulfamate ester instead of $TcesNH_2$. **$Rh_2(C)_2·(4a)_2$**·$(Pyr)_2$ (3.26 mg, 1.0 mol%) was used as the [Rh] catalyst. The chemoselectivity ratio was determined to be **23:1 allylic amine:aziridine** ratio from analysis of the reaction mixture with ^{1}H NMR. Following purification, the title compound was isolated as a colourless oil (23.6 mg, 0.053 mmol, 53%, 77% *ee*). **^{1}H NMR** (700 MHz, $CDCl_3$): δ (ppm) 7.95 (s, 1H, H-3), 7.83 (d, J = 7.9 Hz, 1H, H-7), 7.54 (d, J = 7.8 Hz, 1H, H-5), 7.42 (t, J = 7.7 Hz, 1H, H-6), 6.66 (d, J = 15.8 Hz, 1H, H-8), 6.27 (br. s, 1H, H-15), 6.18 (dd, J = 15.9, 7.2 Hz, 1H, H-9), 4.45 (t, J = 12.4 Hz, 3H, H-16), 4.17 (app. t, J = 6.3 Hz, 1H, H-10), 3.77 – 3.67 (m, 2H, H-13), 2.61 (s, 3H, H-1), 2.12 (br. s, 1H, H-14), 1.92 – 1.82 (m, 2H, H-11), 1.77 – 1.70 (m, 2H, H-12). **^{13}C NMR** (176 MHz, $CDCl_3$): δ (ppm) 198.5, 137.5, 136.7, 131.5, 131.3, 129.7, 129.1, 128.1, 126.1, 118.3 (qt, J = 286.1, 34.3 Hz), 111.6 (tq, J = 256.3, 38.3 Hz), 63.9 (t, J = 28.2 Hz), 62.4, 57.1, 32.5, 27.9, 26.8. **^{19}F NMR**

(376 MHz, $CDCl_3$): δ (ppm) -83.6, -123.4. $[\alpha]_D^{25.0}$ = +38.6 (c. 1.28, $CHCl_3$). **HRMS** (ESI) *m/z* Calc'd for $C_{17}H_{19}F_5NO_5S^-$ [M-H]$^-$: 444.0910, Found: 444.0916 (δ = + 1.4 ppm). **Chiral SFC analysis** DAICEL CHIRALPAK IG (CO_2:MeOH, 92:8, 2.5 mL min^{-1}, 40 °C) t_R = 6.25 (minor), 6.99 (major) minutes.

2,2,2-trichloroethyl (R,E)-(6-hydroxy-1-phenylhex-1-en-3-yl)sulfamate (2a)

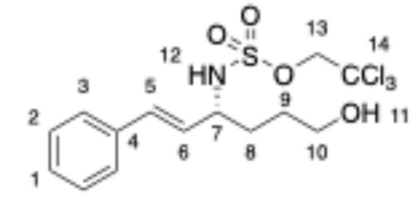

This example refers to entry 15 from Table 1 and the below values are isolated, rather than taken from crude NMR with internal standard (36%) and SFC analysis of crude reaction mixture (93% ee). The chemoselectivity ratio was determined to be **9**:**1 allylic amine:aziridine** ratio from analysis of the reaction mixture with ^{1}H NMR. The compound was prepared using **GP4** with **1a** as the starting material and **$Rh_2(C)_2\cdot(4a)_2$**$\cdot(Pyr)_2$ (3.26 mg, 1.0 mol%) as the [Rh] catalyst. Following purification, the title compound was isolated as a colourless oil (19.6 mg, 0.049 mmol, 49%, 93% *ee*). **^{1}H NMR** (700 MHz, $CDCl_3$): δ (ppm) 7.38-7.35 (m, 2H, H-3), 7.34-7.29 (m, 2H, H-2), 7.27-7.23, 1H, H-1), 6.65 (d, *J* = 15.8 Hz, 1H, H-5), 6.12 (dd, *J* = 15.9, 7.3 Hz, 1H, H-6), 5.82-5.66 (m, 1H, H-12), 4.61 (s, 2H, H-13), 4.23 (app. q, *J* = 6.7 Hz, 1H, H-7), 3.75-3.72 (m, 2H, H-10), 1.89 (app. q, *J* = 7.0 Hz, 2H, H-8), 1.78-1.71 (m, 2H, H-9), 1.62 (br. s, 1H, H-11). **^{13}C NMR** (176 MHz, $CDCl_3$): δ (ppm) 136.0, 132.6, 138.7, 128.1, 128.0, 126.6, 93.5, 78.1, 62.4, 57.2, 32.4, 27.9. $[\alpha]_D^{25.0}$ = +22.8 (c. 1.15, $CHCl_3$). **HRMS** (ESI) *m/z* Calc'd for $C_{14}H_{17}Cl_3NO_4S^-$ [M-H]$^-$: 399.9944, Found: 399.9941 (δ = - 0.8 ppm). **Chiral SFC analysis** DAICEL CHIRALPAK IG (CO_2:MeOH, 80:20, 2.5 mL min^{-1}, 40 °C) t_R = 3.65 (minor), 4.00 (major) minutes.

2,2,2-trichloroethyl 2-(4-hydroxybutyl)-(E)-3-phenylaziridine-1-sulfonate (3a)

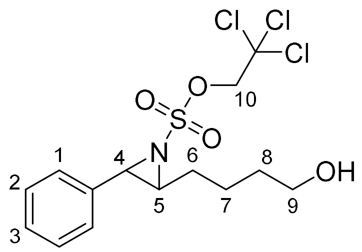

Chemical Formula: $C_{14}H_{18}Cl_3NO_4S$
Exact Mass: 401.0022

The following compound was prepared using **GP4** with **1a** as the starting material and **$Rh_2(esp)_2$** (3.03 mg, 4.0 mol%) as the [Rh] catalyst. Following purification, the title compound was isolated as a colourless oil (9.0 mg, 0.020 mmol, 20% yield, 87% purity). **^{1}H NMR** (700 MHz, $CDCl_3$) δ 7.37 – 7.32 (m, 3H, H-1, H-3), 7.28 (d, *J* = 6.4 Hz, 2H, H-2), 4.77 (app. q, *J* = 10.9 Hz, 2H, H-10), 3.80 (d, *J* = 4.6 Hz, 1H, H-4), 3.71 – 3.64 (m, 2H, H-9), 3.00 – 2.94 (m, 1H, H-5), 2.27 – 2.19 (m, 1H, H-6a), 2.01 – 1.94 (m, 1H, H-6b), 1.77 – 1.62 (m, 4H, H-7, H-8). **^{13}C NMR** (101 MHz, $CDCl_3$) δ 134.1, 128.8, 128.8, 126.6, 92.9, 79.6, 62.3, 52.7, 50.2, 32.0, 28.1, 23.7. **HRMS** (ESI) *m/z* Calc'd for $C_{14}H_{19}Cl_3NO_4S^+$ $[M+H]^+$: 402.0095, Found: 402.0097 (δ = + 0.5).

6.2 Evaluation of 6-C Cis alkenyl alcohol substrate

With the optimised conditions for allylic amination on the 6-C *(E)*-configured alkenyl alcohols in hand, we sought to assess what the chemoselectivity outcome would be on the *(Z)*-configured isomer of **2a**, which has previously been shown to preferentially undergo aziridination under similar reaction conditions.[19] The outcome was that aziridination was still very much preferred (45% yield), although a small amount of allylic amine product (12%) was formed.

Scheme S1. Evaluation of 6-C Cis alkenyl alcohol substrate under optimised conditions

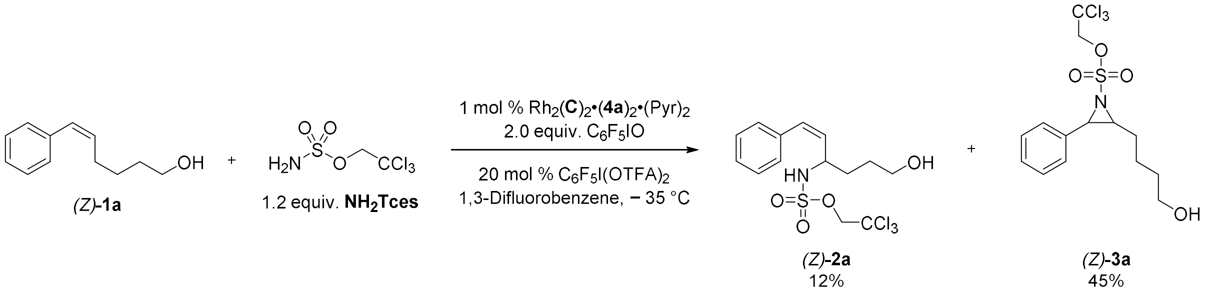

[a]Reactions performed according to **GP4**, on a 0.1 mmol scale with respect to **1a**. Yields refer to NMR yields, using 1,3,5-Trimethoxybenzene as an internal standard.

The synthesis of (Z)-6-phenylhex-5-en-1-ol has previously been reported.[19]

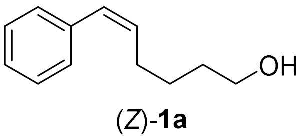

(*Z*)-**1a**

2,2,2-trichloroethyl (Z)-(6-hydroxy-1-phenylhex-1-en-3-yl)sulfamate ((Z)-2a)

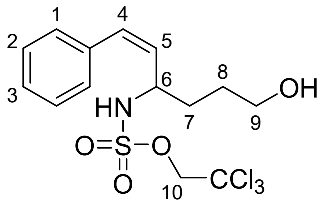

Chemical Formula: $C_{14}H_{18}Cl_3NO_4S$
Exact Mass: 401.0022

The following compound was prepared using **GP4** with ***(Z)*-1a** as the starting material and **$Rh_2(C)_2$·$(4a)_2$**·$(Pyr)_2$ (3.36 mg, 1.0 mol%) as the [Rh] catalyst. Following purification, the title compound was isolated as a colourless oil (3.1 mg, 0.008 mmol, 8%). **^{1}H NMR** (700 MHz, $CDCl_3$) δ 7.35 (t, *J* = 7.5 Hz, 2H, H-2), 7.31 – 7.27 (m, 3H, H-1, H-3), 6.63 (d, *J* = 11.7 Hz, 1H, H-4), 5.60 (dd, *J* = 11.6, 9.8 Hz, 1H, H-5), 5.51 (s, 1H, NH), 4.59 (dt, *J* = 9.6, 6.5 Hz, 1H, H-6), 4.52 (s, 2H, H-10), 3.66 (t, *J* = 5.9 Hz, 2H, H-9), 1.88 – 1.75 (m, 2H, H-8), 1.75 – 1.61 (m, 2H, H-7), 1.26 (s, 1H, OH). **^{13}C NMR** (176 MHz, $CDCl_3$) δ 136.1, 132.0, 131.0, 128.7, 128.6, 127.7, 93.6, 78.3, 62.4, 52.7, 32.8, 27.9. **HRMS** (ESI) *m/z* Calc'd for $C_{14}H_{17}Cl_3NO_4S^-$ $[M-H]^-$: 399.9949, Found: 399.9941 (δ = - 2.0 ppm).

2,2,2-trichloroethyl 2-(4-hydroxybutyl)-(Z)-3-phenylaziridine-1-sulfonate ((Z)-3a)

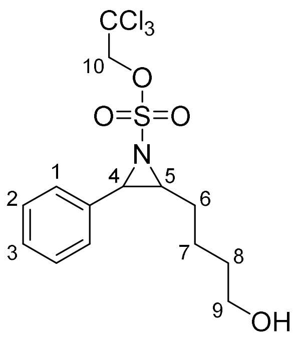

Chemical Formula: $C_{14}H_{18}Cl_3NO_4S$
Exact Mass: 401.0022

The following compound was prepared using **GP4** with ***(Z)*-1a** as the starting material and **$Rh_2(C)_2$·$(4a)_2$**·$(Pyr)_2$ (3.36 mg, 1.0 mol%) as the [Rh] catalyst. Following purification, the title compound was isolated as a colourless oil (12.6 mg, 0.032 mmol, 32%). **^{1}H NMR** (700 MHz, $CDCl_3$) δ 7.40 – 7.31 (m, 5H, H-1, H-2, H-3), 4.95 – 4.80 (m, 2H, H-10), 4.09 (dd, *J* = 7.3, 2.8 Hz, 1H, H-4), 3.60 – 3.49 (m, 2H, H-9), 3.23 – 3.13 (m, 1H, H-5), 1.57 – 1.44 (m, 4H, H-7, H-8), 1.43 – 1.37 (m, 2H, H-6), 1.28 – 1.19 (m, 1H, OH). **^{13}C NMR** (176 MHz, $CDCl_3$) δ 131.8, 128.7, 128.5, 127.5, 93.1, 79.7, 62.5, 48.7, 47.9, 32.0, 25.8, 23.1. **HRMS** (ESI) *m/z* Calc'd for $C_{14}H_{19}Cl_3NO_4S^+$ $[M+H]^+$: 402.0095, Found: 402.0078 (δ = - 4.2 ppm).

7. Post Functionalisation of Allylic Amines

Methyl (S,E)-3-(2-(1-((2,2,2-trichloroethoxy)sulfonyl)(Pyr)rolidin-2-yl)vinyl)benzoate

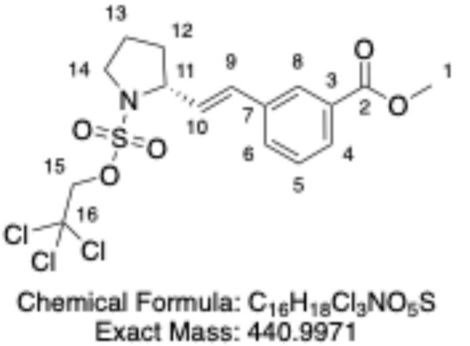

Chemical Formula: $C_{16}H_{18}Cl_3NO_5S$
Exact Mass: 440.9971

To a vial charged with amino alcohol (24.1 mg, 0.05 mmol, 1.0 eq.) under a nitrogen atmosphere was added Pyridine (anhyd., 0.025 M). The solution was cooled to 0 °C, methanesulfonyl chloride (7.74 µL, 0.1 mmol, 2.0 eq.) was added dropwise and the resulting mixture was stirred at room temperature for 3 hours. The reaction was diluted with methylene chloride (10 mL) and subsequently washed with HCl (3 N, 10 mL), $NaHCO_3$ (sat. soln., 10 mL) and brine (10 mL). Following this, the organics were collected, dried over $MgSO_4$, filtered and concentrated under reduced pressure to give the crude mesylated alcohol. The mesylate was transferred to a microwave vial, where K_2CO_3 (20.7 mg, 0.15 mmol, 3.0 eq.) and acetone (5.0 mL) were added. The vial was sealed under air and the resulting suspension was stirred at 100 °C for 3 h. After cooling to room temperature, the solvent was removed under a stream of nitrogen to give the crude residue, which was dissolved in a minimum amount of methylene chloride and purified by FCC (SiO_2, 0-5% v/v acetone in $CHCl_3$) to give the title compound as a pale-brown oil (11.1 mg, 0.025 mmol, 50% over 2 steps, 87% *ee*).

^{1}H NMR (700 MHz, $CDCl_3$): δ (ppm) 8.06 (app. t, *J* = 1.5 Hz, 1H, H-8), 7.90 (dt, *J* = 7.7, 1.3 Hz, 1H, H-4), 7.55-7.53 (m, 1H, H-6), 7.38 (app. t, *J* = 7.7 Hz, 1H, H-5), 6.64 (d, *J* = 15.7 Hz, 1H, H-9), 6.24 (dd, *J* = 15.8, 7.1 Hz, 1H, H-10), 4.74-4.47 (m, 3H, H-11, H-15), 3.92 (s, 3H, H-1), 3.68-3.63 (m, 1H, H-14a), 3.60-3.56 (m, 1H, H-14b), 2.31-2.25 (m, 1H, H-12a), 2.11-2.03 (m, 2H, H-13), 1.94-1.92 (m, 1H, H-12b). **^{13}C NMR** (176 MHz, $CDCl_3$): δ (ppm) 166.9, 136.6, 131.1, 130.8, 130.5, 129.7, 128.9, 128.7, 127.5, 93.8, 77.6, 63.1, 52.2, 49.8, 32.9, 24.4. $[\alpha]_D^{25.0}$ = +32.6 (c. 1.39, $CHCl_3$). **HRMS** (ESI) *m/z* Calc'd for $C_{16}H_{18}Cl_3NO_5SNa^+$ $[M+Na]^+$: 463.9863, Found: 463.9884 (δ = + 4.4 ppm). **Chiral SFC analysis** DAICEL CHIRALPAK IG (CO_2:MeOH, 80:20, 2.5 mL min^{-1}, 40 °C) t_R = 7.78 (minor), 16.28 (major) minutes.

8. Determination of Absolute Stereochemistry

tert-butyl (R,E)-(6-hydroxy-1-phenylhex-1-en-3-yl)carbamate

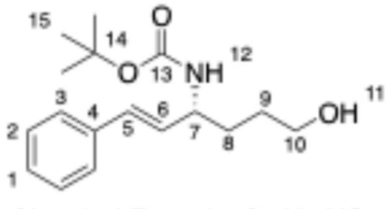

Chemical Formula: $C_{17}H_{25}NO_3$
Exact Mass: 291.1834

To a 4-dram vial was added **2a** (51.2 mg, 0.13 mmol, 1.0 eq.) and 1,4-dioxane/H_2O (2:1 v/v 2.25 mL). The vial was sealed and heated at 80 ˚C overnight. Following this, the vial was cooled to rt and K_2CO_3 (250 mg, 1.80 mmol, 14.0 equiv.) was added, and the reaction was stirred for 15 min at rt. Water (10 mL) was added to the mixture and the aqueous layer was extracted with methylene chloride (3 × 10 mL). The combined organic layers were dried over $MgSO_4$, filtered and concentrated *in vacuo*. The crude mixture was used without further purification for the next step. To an RBF was added the crude reaction mixture (21.5 mg), Boc_2O (34.0 mg, 0.15 mmol, 1.1 eq.) and methylene chloride (10 ml). The mixture was cooled to 0 °C and TEA (41 µL, 0.30 mmol, 2.0 eq.) was added dropwise. The reaction mixture was stirred at rt for 6 h. Following this, the reaction was diluted with water (10 mL) and the aqueous layer was extracted with methylene chloride (3 × 10 mL). The combined organic layers were dried over $MgSO_4$, filtered and concentrated *in vacuo*. The crude mixture was purified by FCC (SiO_2, v/v 0-30% EtOAc in hexanes) to give the title compound as a colourless oil (8.2 mg, 0.028 mmol, 22% over 2 steps).

^{1}H NMR (700 MHz, $CDCl_3$): δ (ppm) 7.37-7.34 (m, 2H, H-3), 7.33-7.28 (m, 2H, H-2), 7.25-7.21 (m, 1H, H-1), 6.52 (d, *J* = 15.9 Hz, 1H, H-5), 6.09 (dd, *J* = 15.8, 6.2 Hz, 1H, H-6), 4.60 (br. s, 1H, H-12), 4.31 (br. s, 1H, H-7), 3.72-3.67 (m, 2H, H-10), 1.77-1.64 (m, 4H, H-8, H-9), 1.45 (s, 9H, H-15), 1.30-1.20 (m, 1H, H-11). **^{13}C NMR** (176 MHz, $CDCl_3$): δ (ppm) 155.4, 136.7, 130.3, 128.6, 127.6, 126.4, 79.5, 62.6, 52.1, 32.1, 28.9, 28.4. $[\alpha]_D^{25.0}$ = +29.2 (c. 0.75, $CHCl_3$). **HRMS** (ESI) *m/z* Calc'd for $C_{17}H_{22}Cl_3NO_5SNa^+$ $[M+Na]^+$: 314.1732, Found: 314.1732 (δ = 0.0 ppm). The spectroscopic data matches with that reported in the literature.[21]

9. "Knock-out" studies

Table S4 - Summary of yields and ee values for 2a using various modified chiral cations

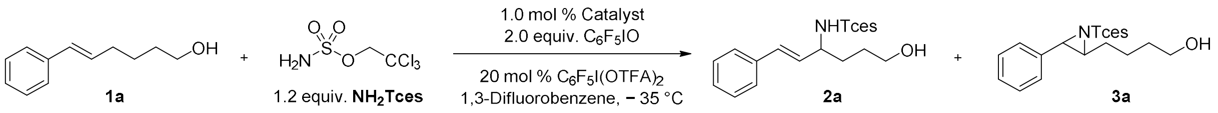

Entry	Catalyst	Yield of 2a / %[a]	Yield of 3a / %[a]	*ee* of 2a / %[b]
**1**	Rh_2(**A**)$_2$•(**4b**)$_2$•(Pyr)$_2$	23%	7%	82%
**2**	Rh_2(**A**)$_2$•(**4f**)$_2$•(Pyr)$_2$	28%	5%	81%[c]
**3**	Rh_2(**A**)$_2$•(**4g**)$_2$•(Pyr)$_2$	10%	19%	59%
**4**	Rh_2(**A**)$_2$•(**4h**)$_2$•(Pyr)$_2$	<5%	4%	N.D.
**5**	Rh_2(**A**)$_2$•(**4i**)$_2$•(Pyr)$_2$	26%	9%	81%
**6**	Rh_2(**A**)$_2$•(**4j**)$_2$•(Pyr)$_2$	7%	19%	-25%
**7**	Rh_2(**A**)$_2$•(**4k**)$_2$•(Pyr)$_2$	6%	19%	-39%

[a]Reactions performed according to **GP4**, on a 0.1 mmol scale with respect to **1a**. Yields refer to NMR yields of **2a** and **3a**, respectively, using 1,3,5-Trimethoxybenzene as an internal standard. [b]*ee* determined by chiral SFC analysis of purified **2a**. [c]The antipode 2a was obtained in this case, but the magnitude of *ee* is shown for clarity.

10. Chiral SFC Traces

2,2,3,3,3-pentafluoropropyl (R,E)-(6-hydroxy-1-phenylhex-1-en-3-yl)sulfamate (2a-pfps)

SFC CHIRALPAK IG (CO_2:MeOH = 95:05, 2.50 ml min^{-1}, 40 °C, 248 nm) t_R = 5.91 min (minor), 6.25 min (major) indicated 90% *ee.*

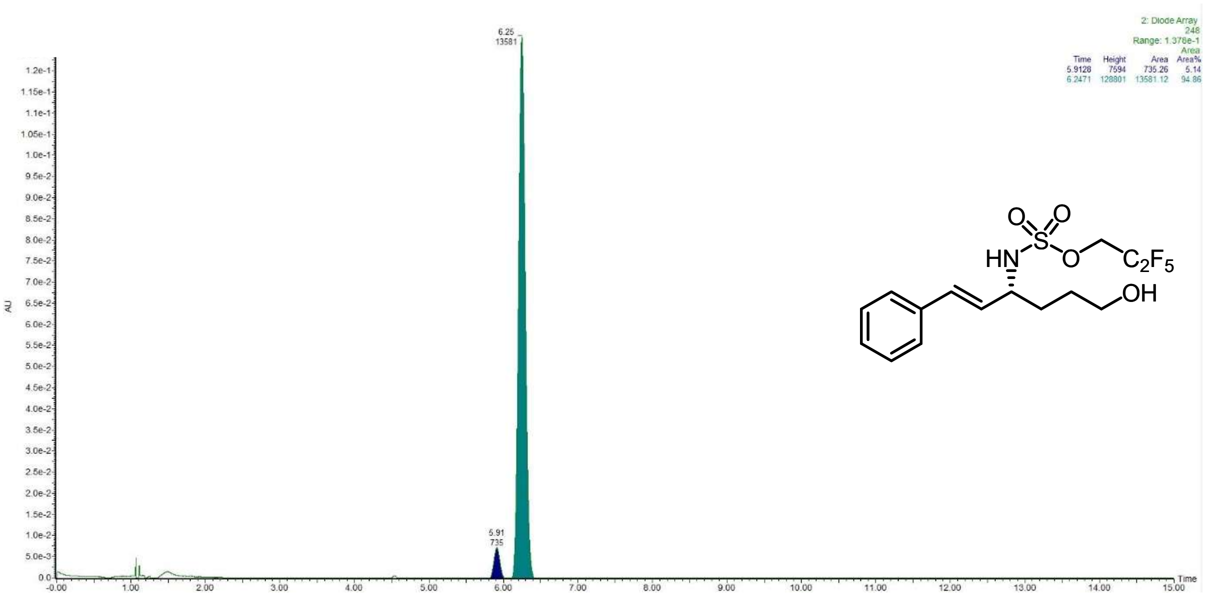

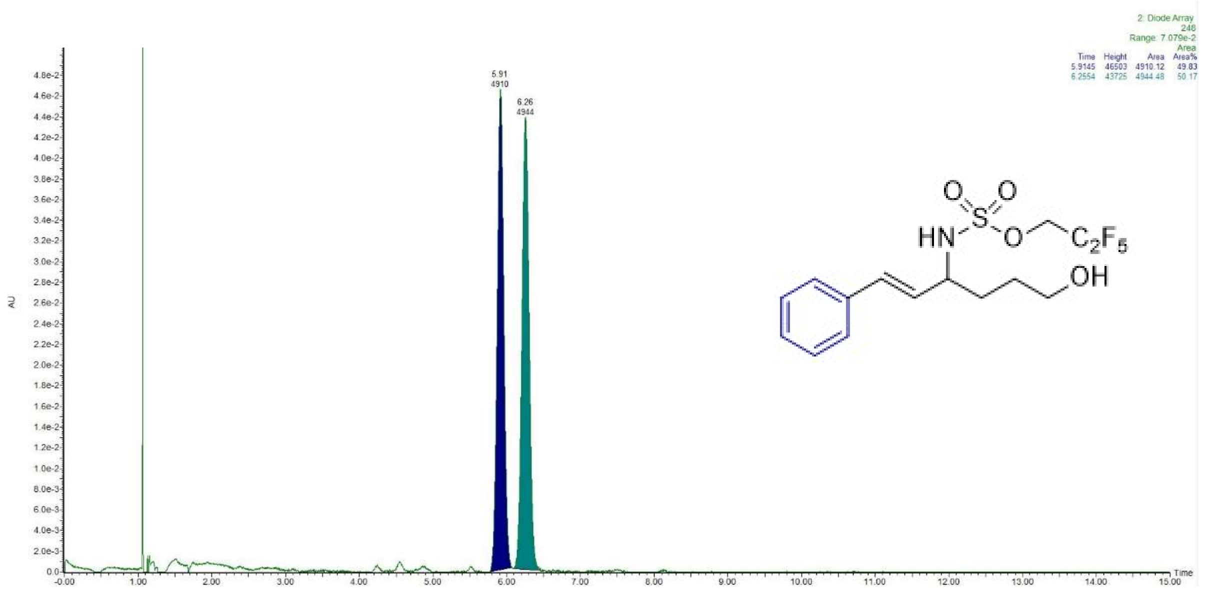

2,2,3,3,3-pentafluoropropyl (R,E)-(1-(3-acetylphenyl)-6-hydroxyhex-1-en-3-yl)sulfamate (2b-pfps)

SFC CHIRALPAK IG (CO_2:MeOH = 92:08, 2.50 ml min^{-1}, 40 °C, 232 nm) t_R = 6.25 min (minor), 6.99 min (major) indicated 77% *ee.*

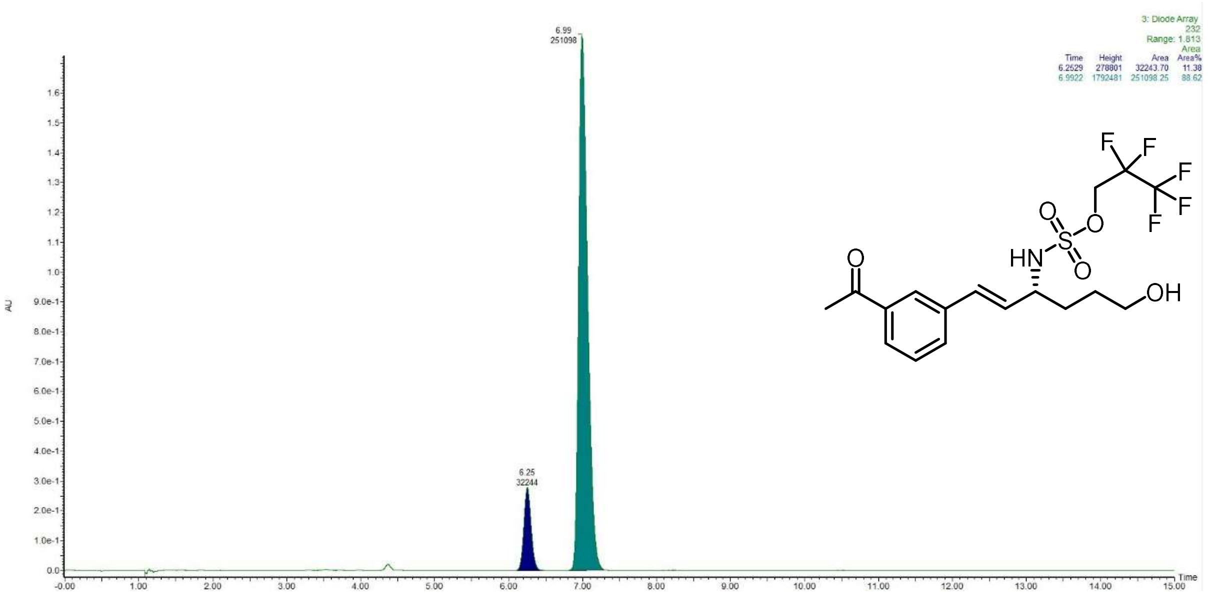

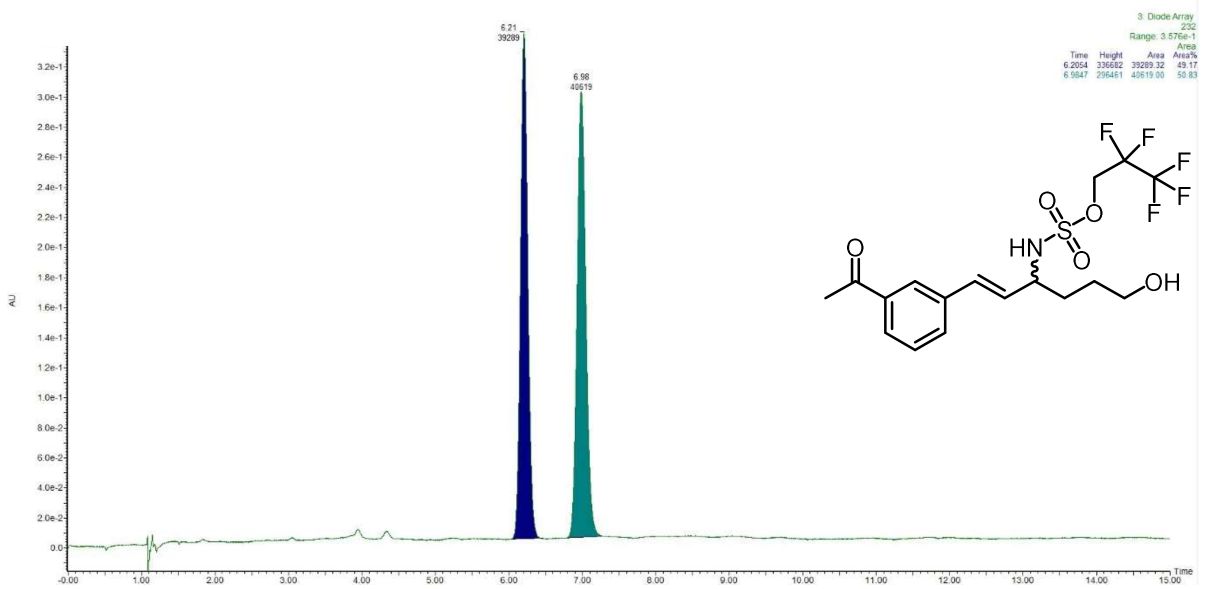

2,2,2-trichloroethyl (R,E)-(6-hydroxy-1-phenylhex-1-en-3-yl)sulfamate (2a)

SFC CHIRALPAK IG (CO_2:MeOH = 80:20, 2.50 ml min^{-1}, 40 °C, 248 nm) t_R = 3.65 min (minor), 4.00 min (major) indicated 93% *ee.*

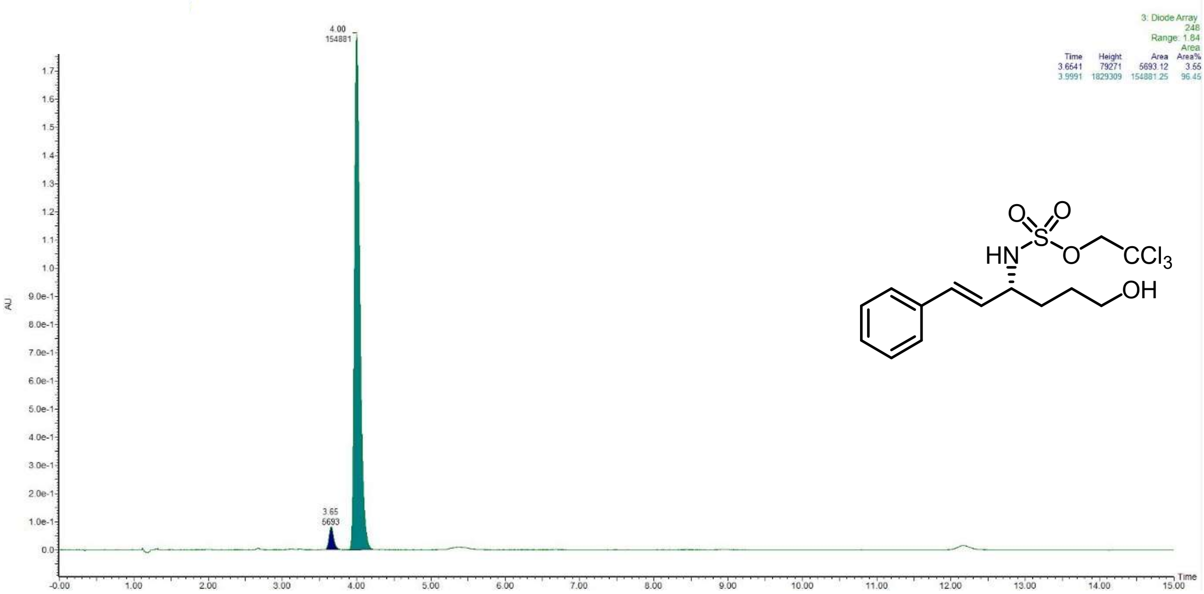

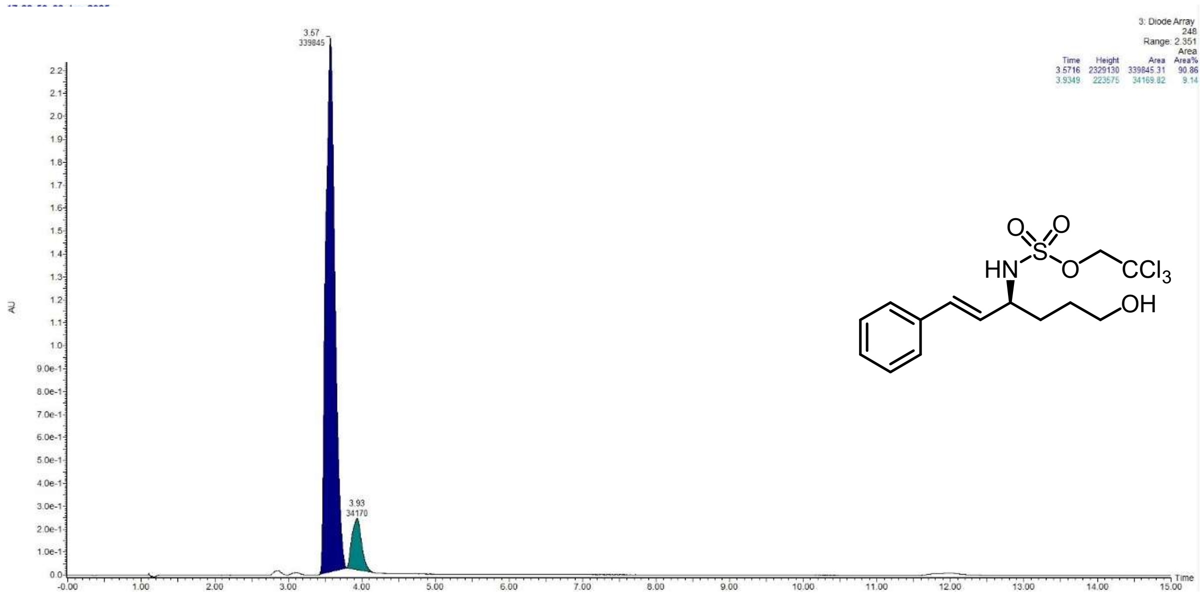

2,2,2-trichloroethyl (R,E)-(1-([1,1'-biphenyl]-4-yl)-6-hydroxyhex-1-en-3-yl)sulfamate (2c)

SFC CHIRALPAK IE (CO_2:MeOH = 85:15, 2.5 ml min^{-1}, 40 °C, 248 nm) t_R = 12.75 min (major), 13.81 min (minor) indicated 97% *ee.*

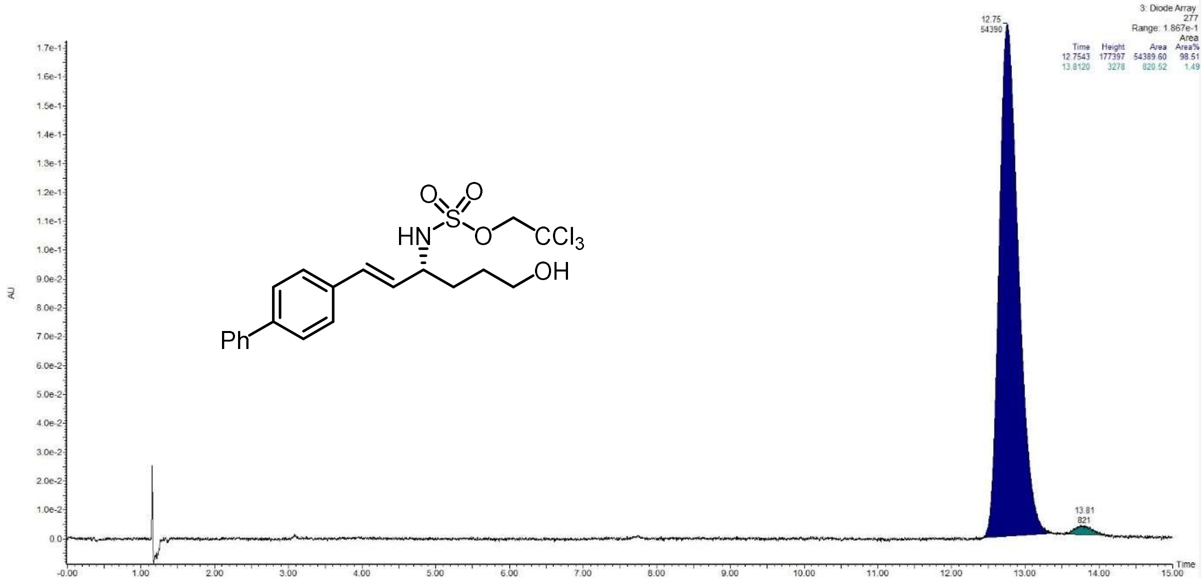

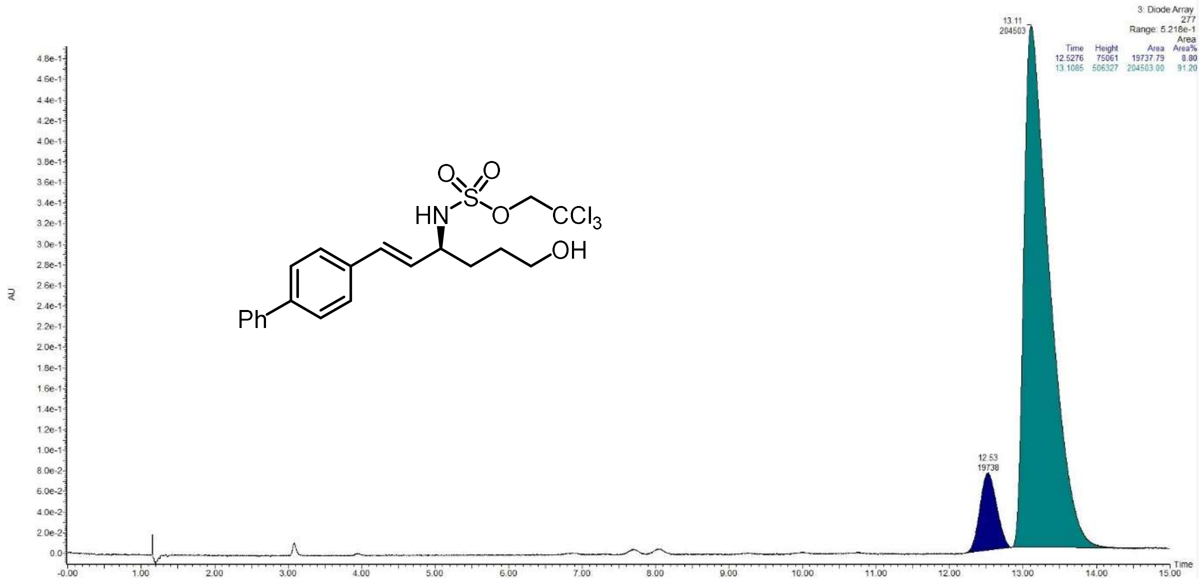

2,2,2-trichloroethyl (R,E)-(1-(4-fluorophenyl)-6-hydroxyhex-1-en-3-yl)sulfamate (2d)

SFC CHIRALPAK IG (CO_2:MeOH = 80:20, 2.5 ml min^{-1}, 40 °C, 248 nm) t_R = 3.01 min (minor), 3.25 min (major) indicated 94% *ee.*

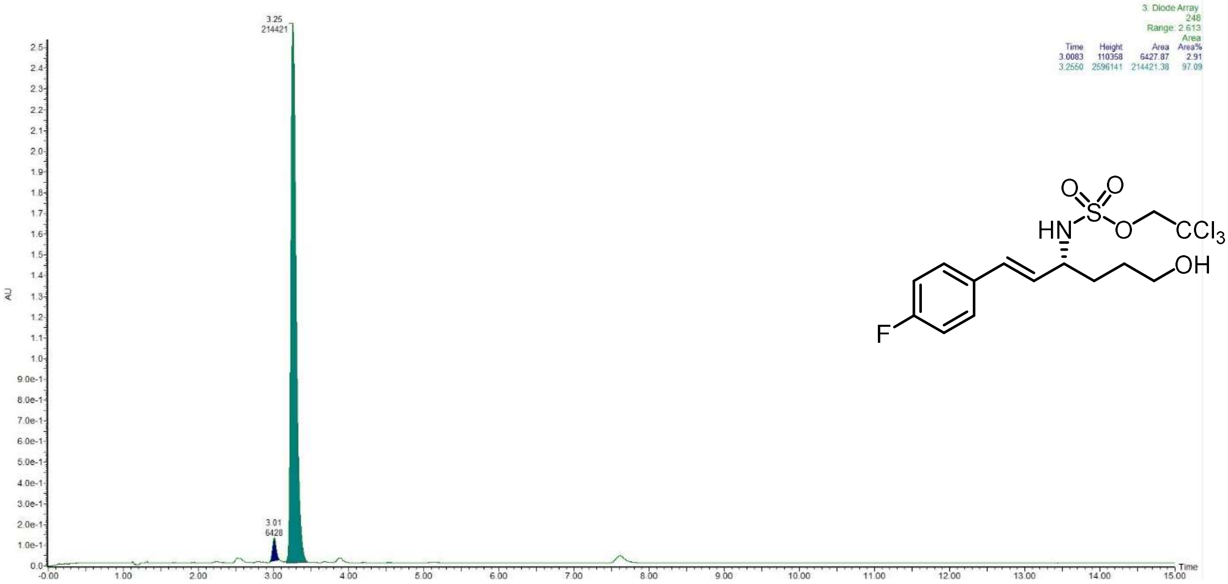

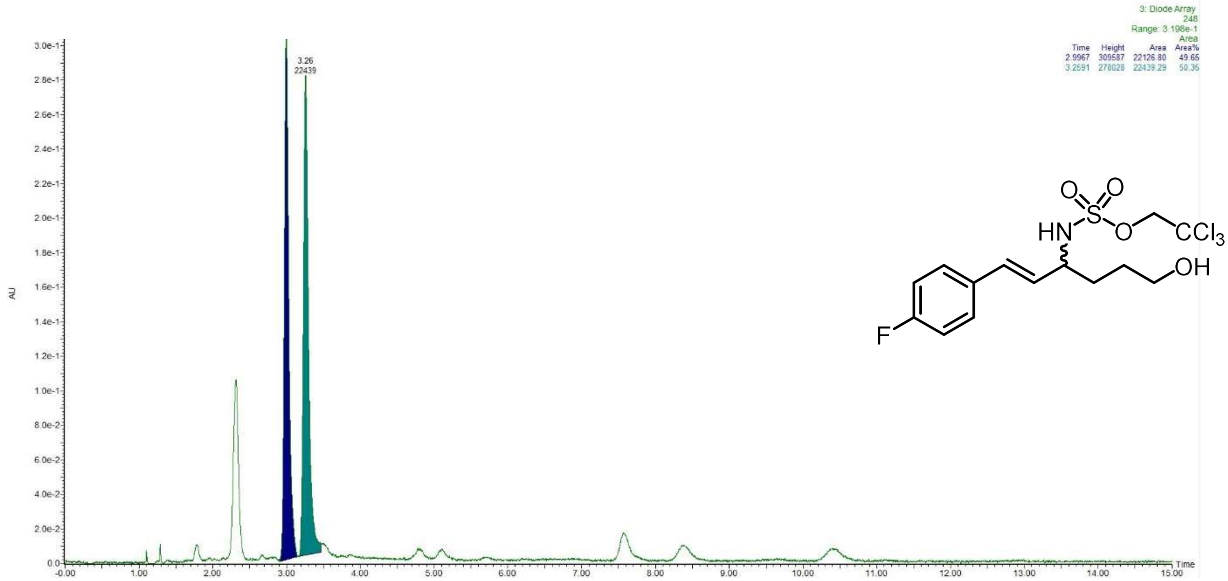

2,2,2-trichloroethyl (R,E)-(1-(4-(tert-butyl)phenyl)-6-hydroxyhex-1-en-3-yl)sulfamate (2e)

SFC CHIRALPAK IG (CO_2:MeOH = 80:20, 2.5 ml min^{-1}, 40 °C, 248 nm) t_R = 6.43 min (minor), 8.73 min (major) indicated 97% *ee.*

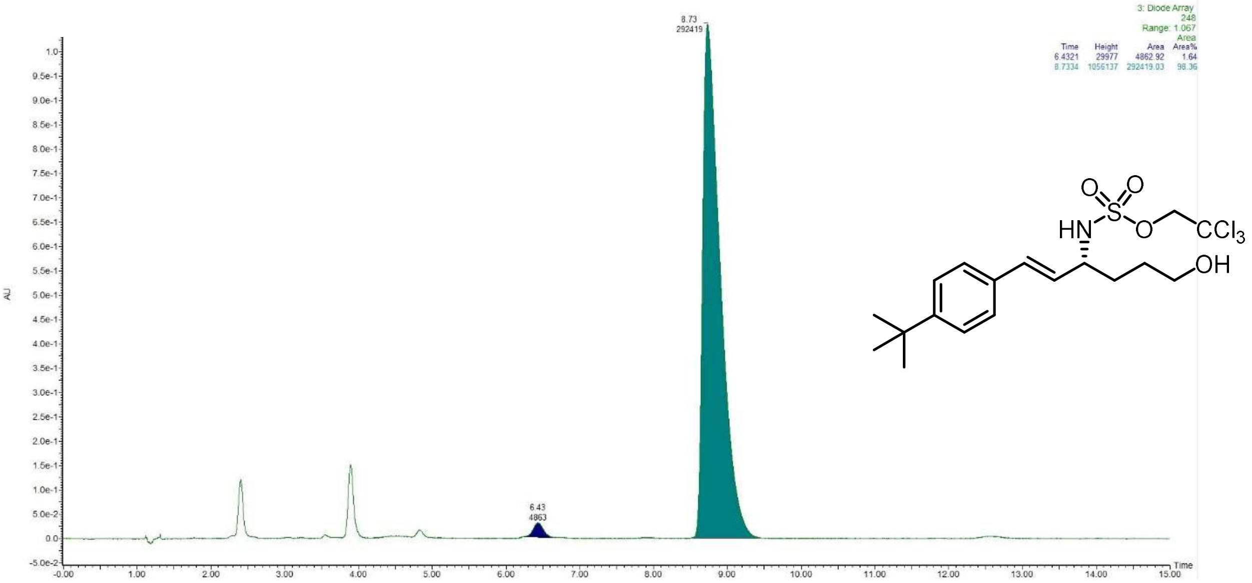

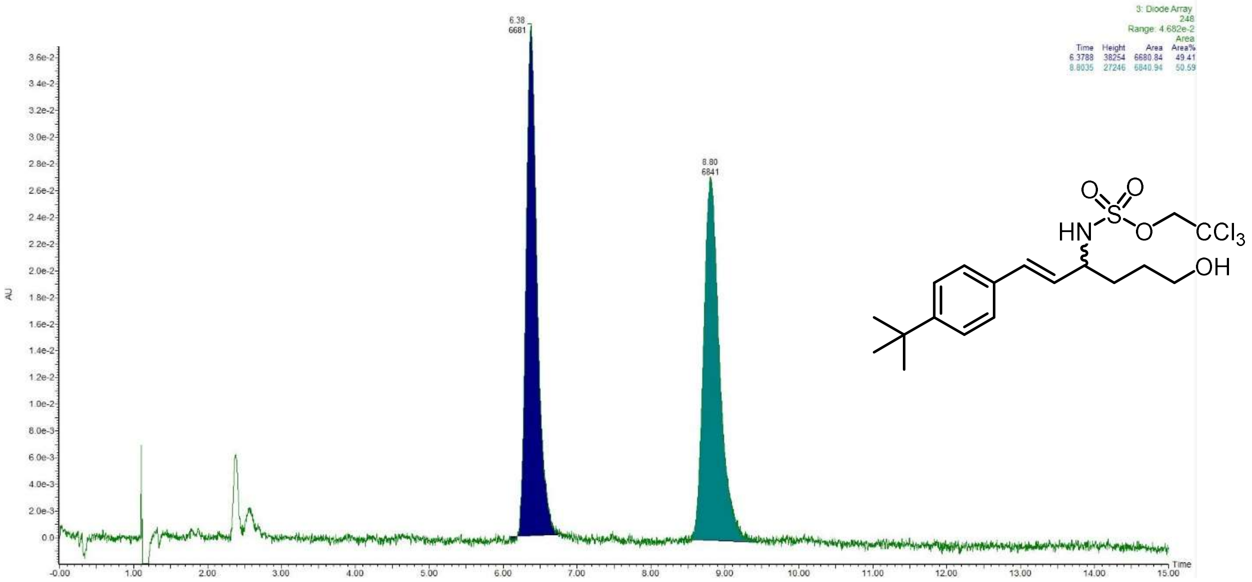

(R,E)-3-(6-hydroxy-3-(((2,2,2-trichloroethoxy)sulfonyl)amino)hex-1-en-1-yl)phenyl acetate (2f)

SFC CHIRALPAK IG (CO_2:MeOH = 85:15, 2.5 ml min^{-1}, 40 °C, 248 nm) t_R = 6.52 min (minor), 6.96 min (major) indicated 86% *ee.*

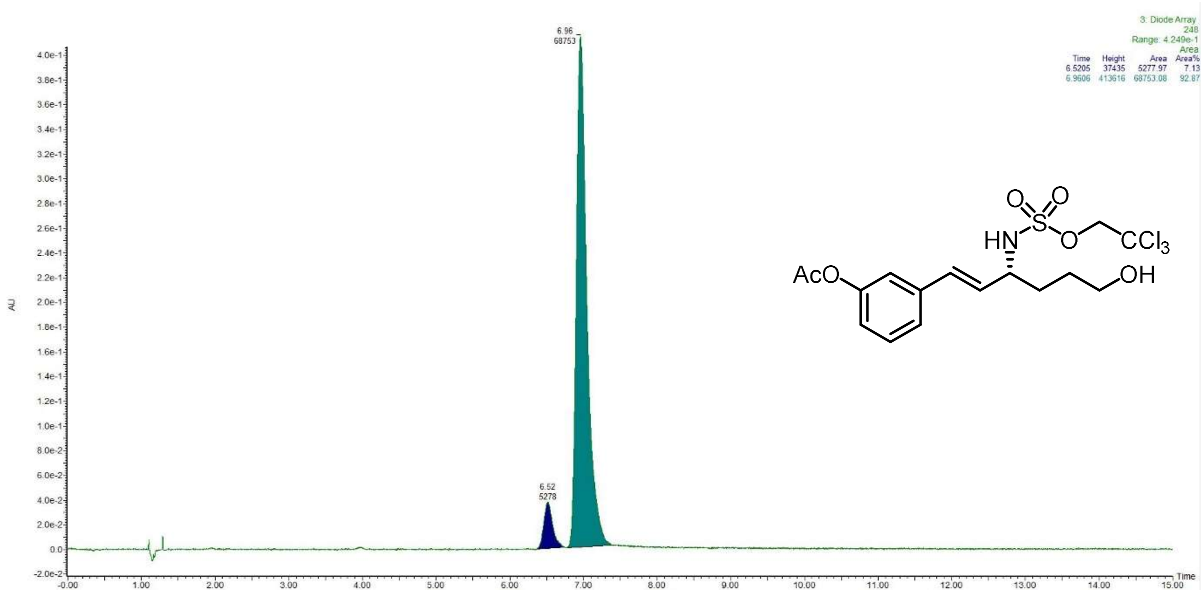

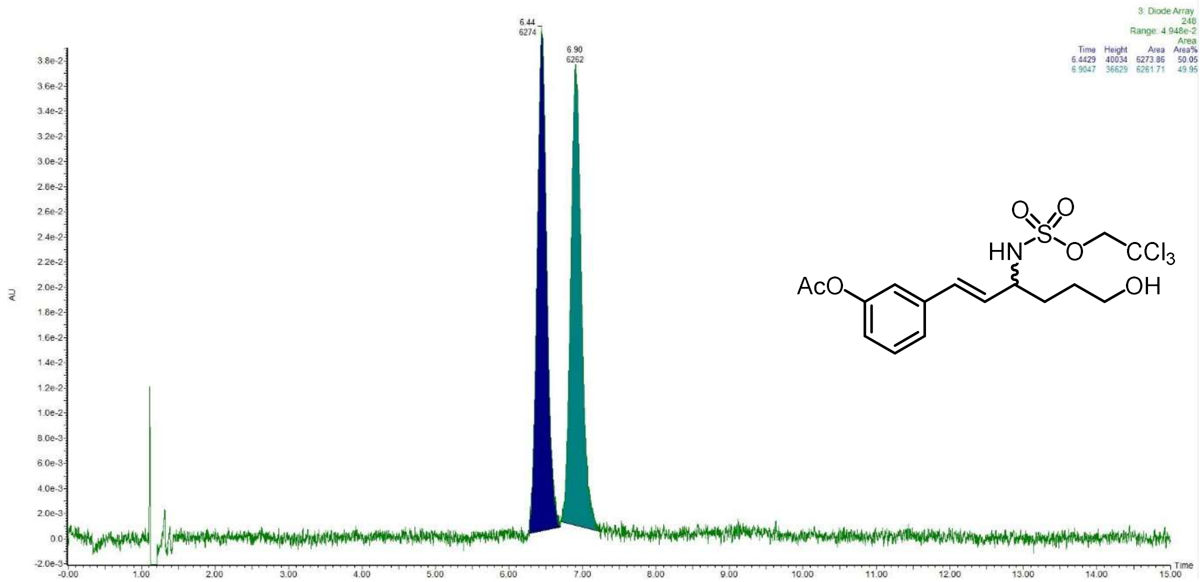

2,2,2-trichloroethyl (R,E)-(1-(3-acetylphenyl)-6-hydroxyhex-1-en-3-yl)sulfamate (2b)

SFC CHIRALPAK IG (CO_2:MeOH = 80:20, 2.5 ml min^{-1}, 40 °C, 232 nm) t_R = 6.48 min (minor), 7.27 min (major) indicated 73% *ee.*

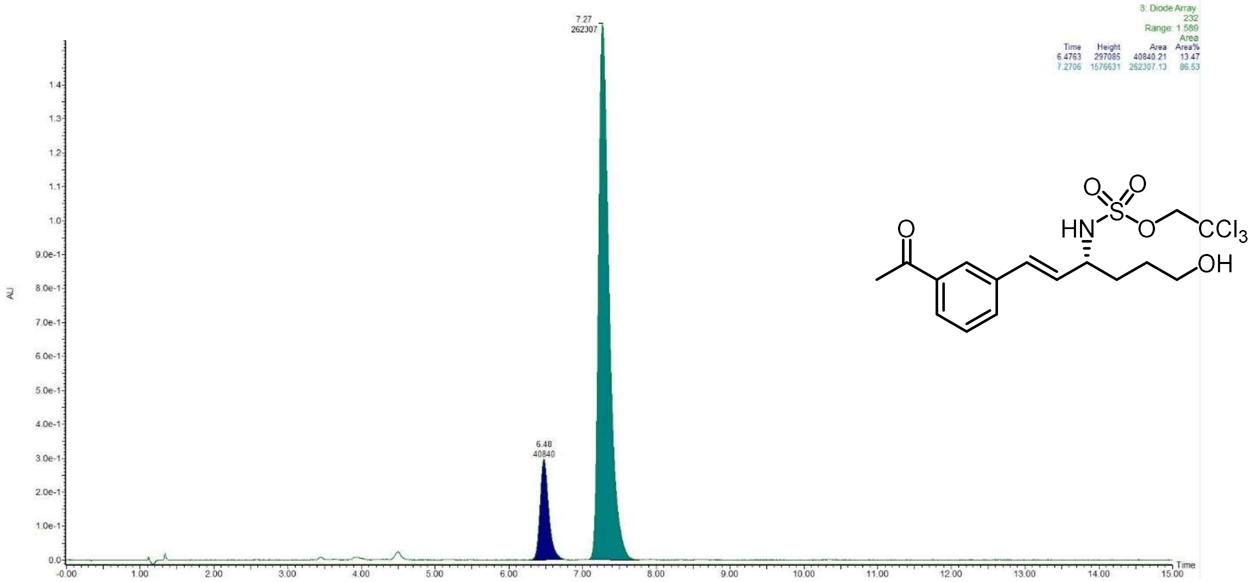

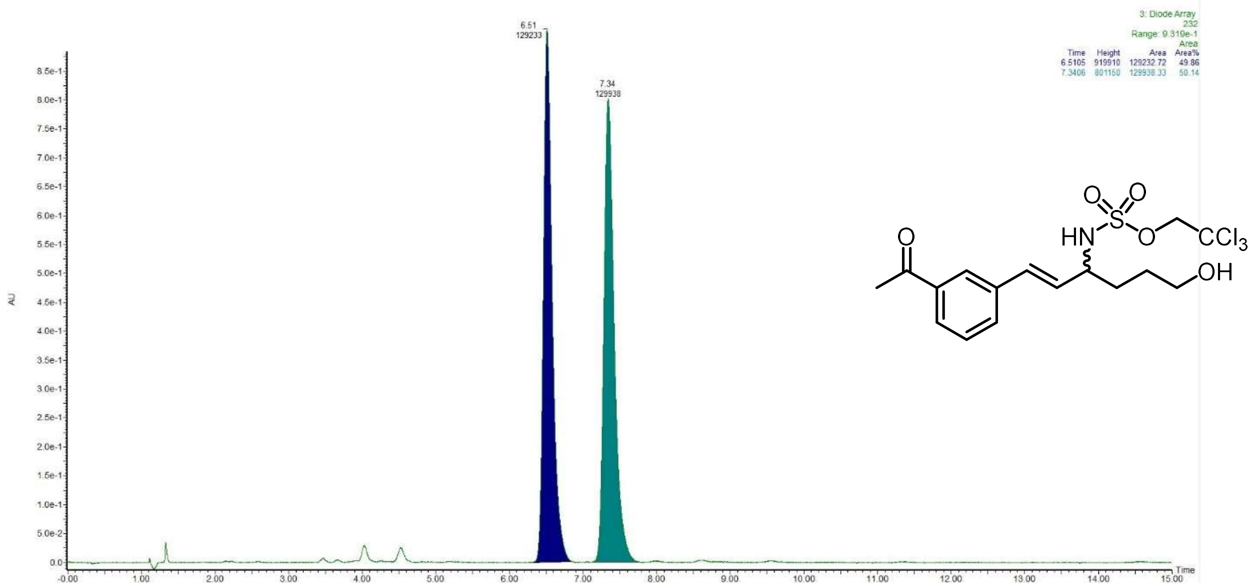

2,2,2-trichloroethyl (R,E)-(1-(3-(tert-butyl)phenyl)-6-hydroxyhex-1-en-3-yl)sulfamate (2g)

SFC CHIRALPAK IC (CO_2:MeOH = 91:09, 2.5 ml min^{-1}, 40 °C, 248 nm) t_R = 16.87 min (major), 17.62 min (minor) indicated 92% *ee.*

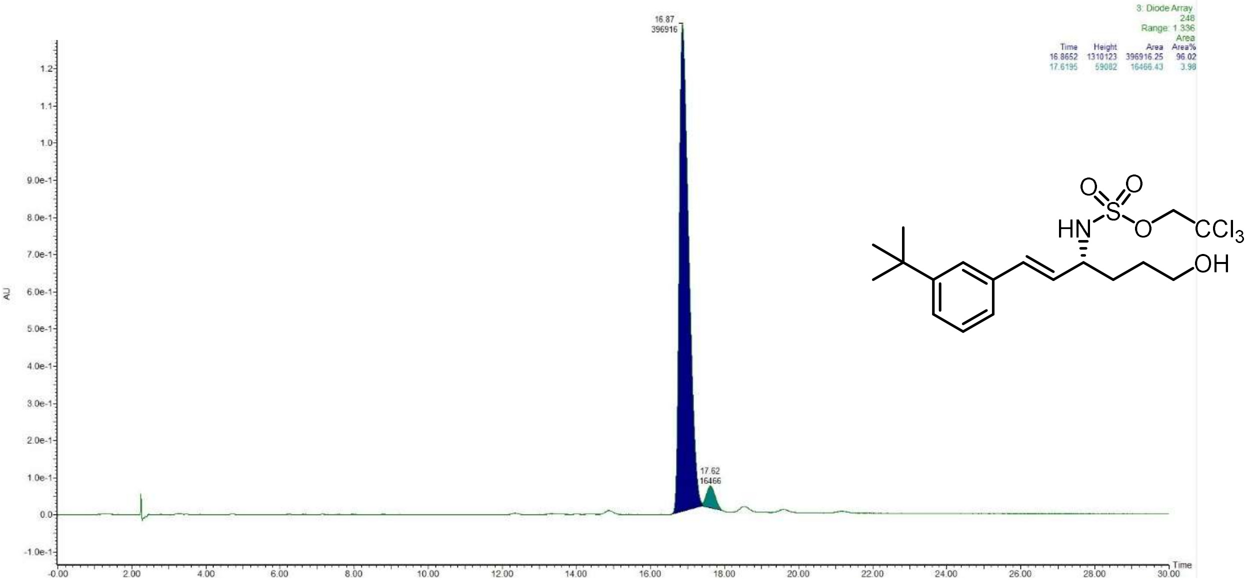

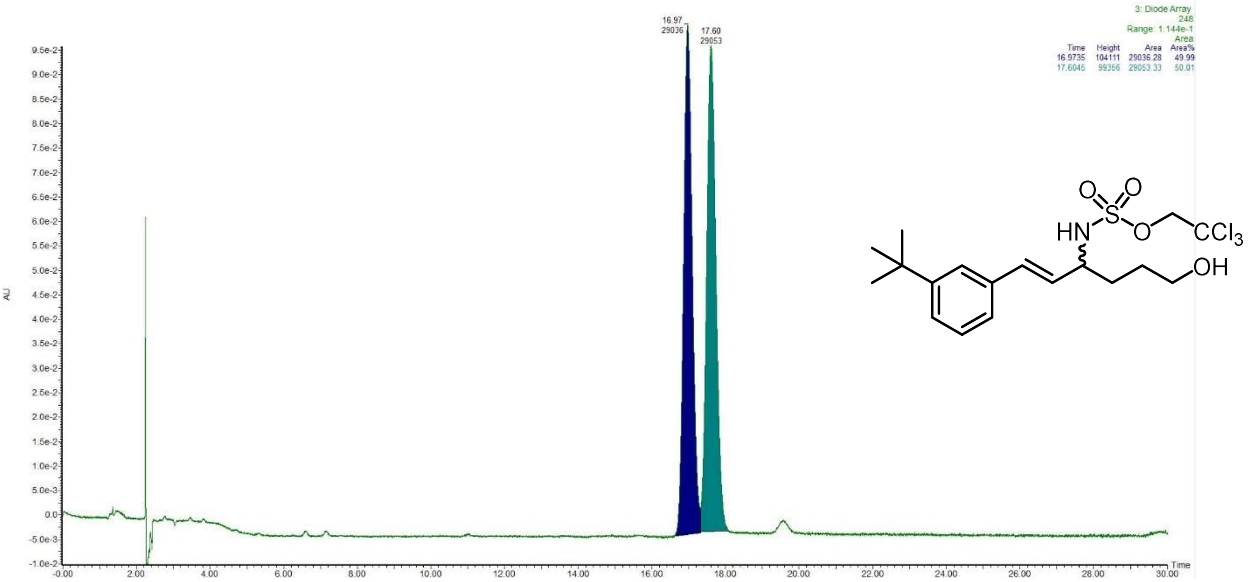

2,2,2-trichloroethyl (R,E)-(6-hydroxy-1-(3-methoxyphenyl)hex-1-en-3-yl)sulfamate (2h)

SFC CHIRALPAK IG (CO_2:MeOH = 80:20, 2.5 ml min^{-1}, 40 °C, 248 nm) t_R = 5.08 min (minor), 6.07 min (major) indicated 90% *ee.*

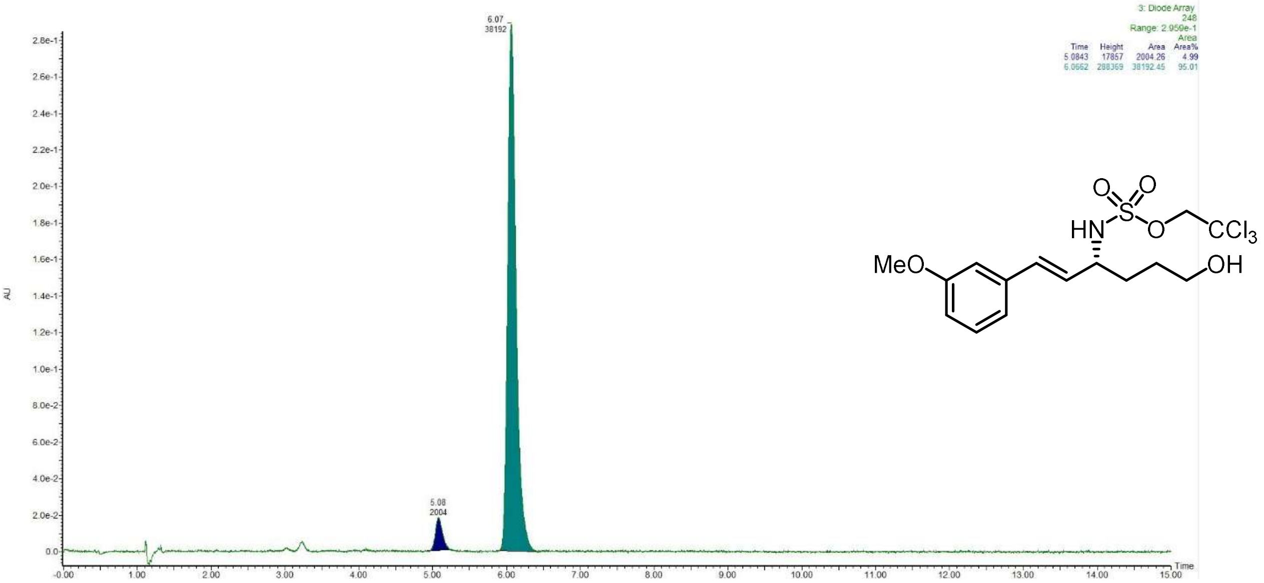

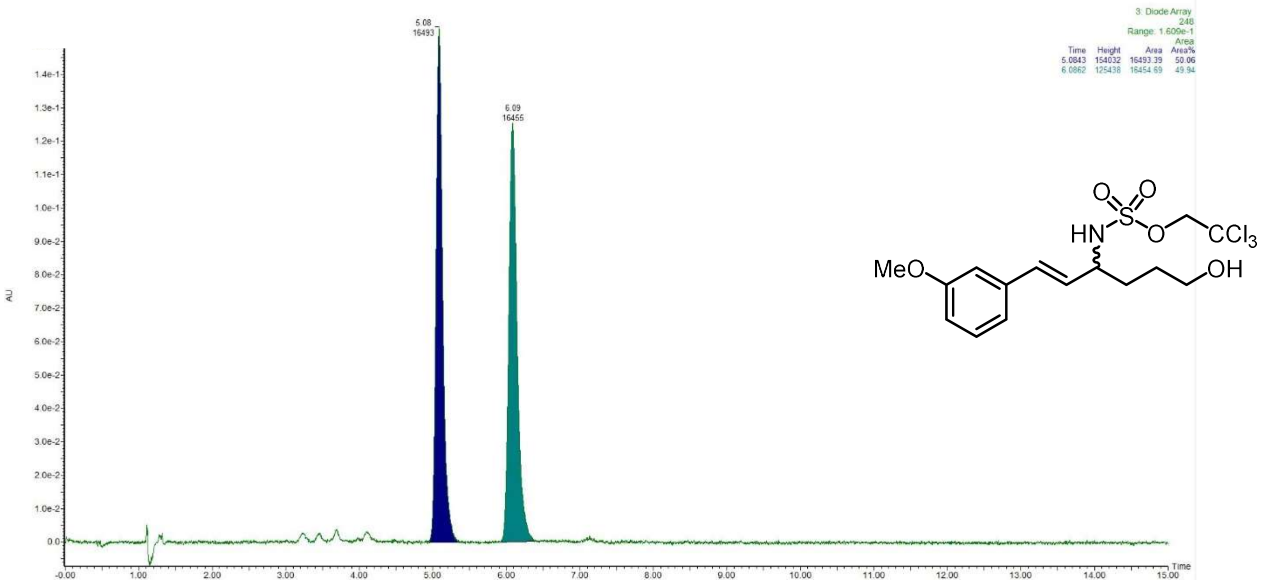

Methyl (R,E)-3-(6-hydroxy-3-(((2,2,2-trichloroethoxy)sulfonyl)amino)hex-1-en-1-yl) benzoate (2i)

SFC CHIRALPAK IG (CO_2:MeOH = 80:20, 2.5 ml min^{-1}, 40 °C, 248 nm) t_R = 5.87 min (minor), 6.68 min (major) indicated 88% *ee.*

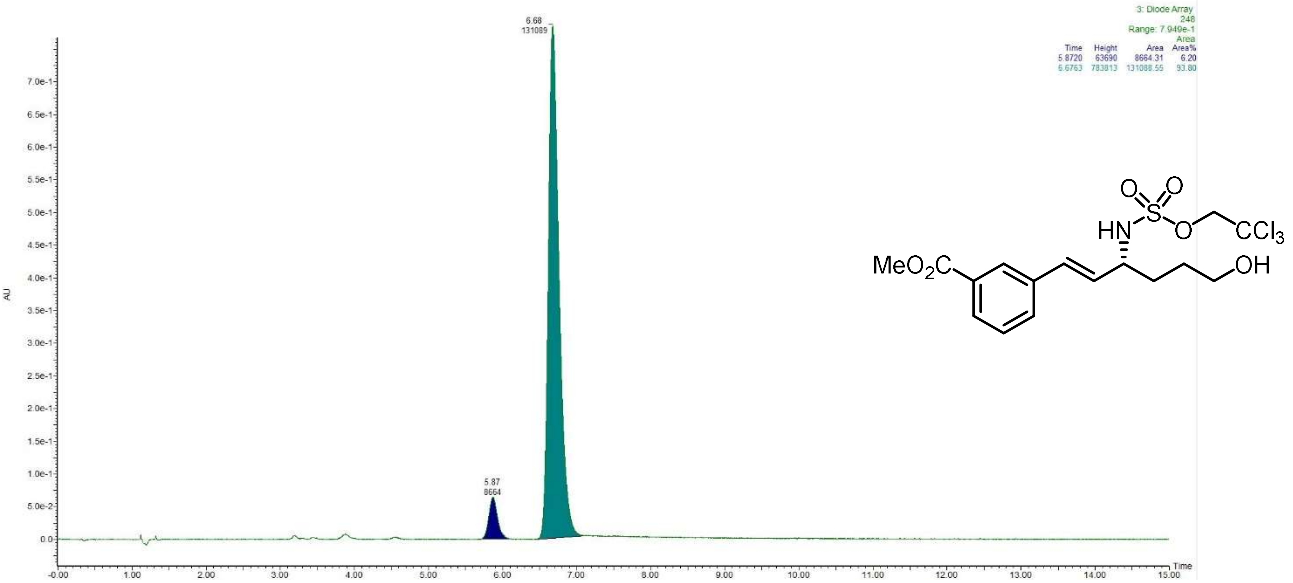

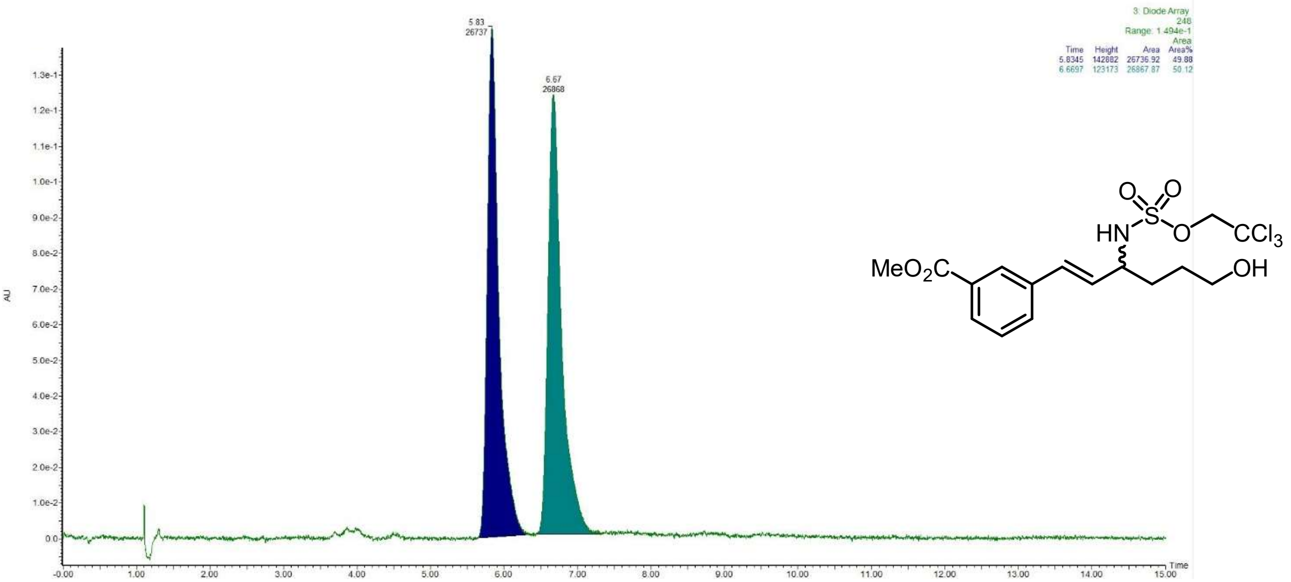

2,2,2-trichloroethyl (R,E)-(1-(3-((tert-butoxycarbonyl)amino)phenyl)-6-hydroxyhex-1-en-3-yl)sulfamate (2j)

SFC CHIRALPAK IG (CO_2:MeOH = 80:20, 2.5 ml min^{-1}, 40 °C, 248 nm) t_R = 5.57 min (minor), 6.69 min (major) indicated 81% *ee.*

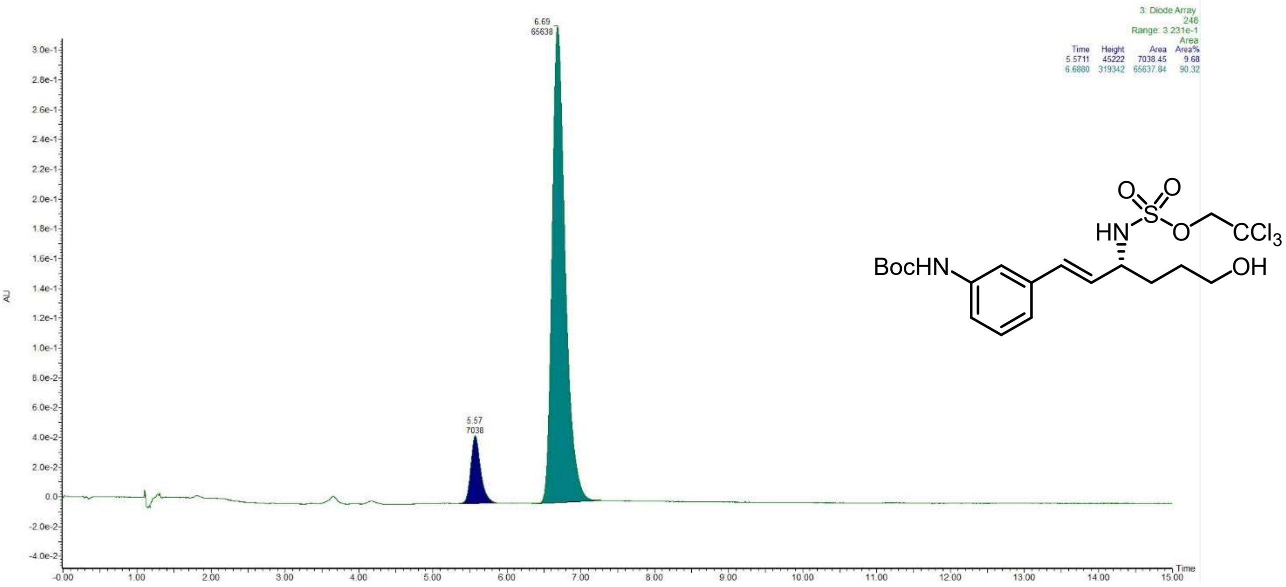

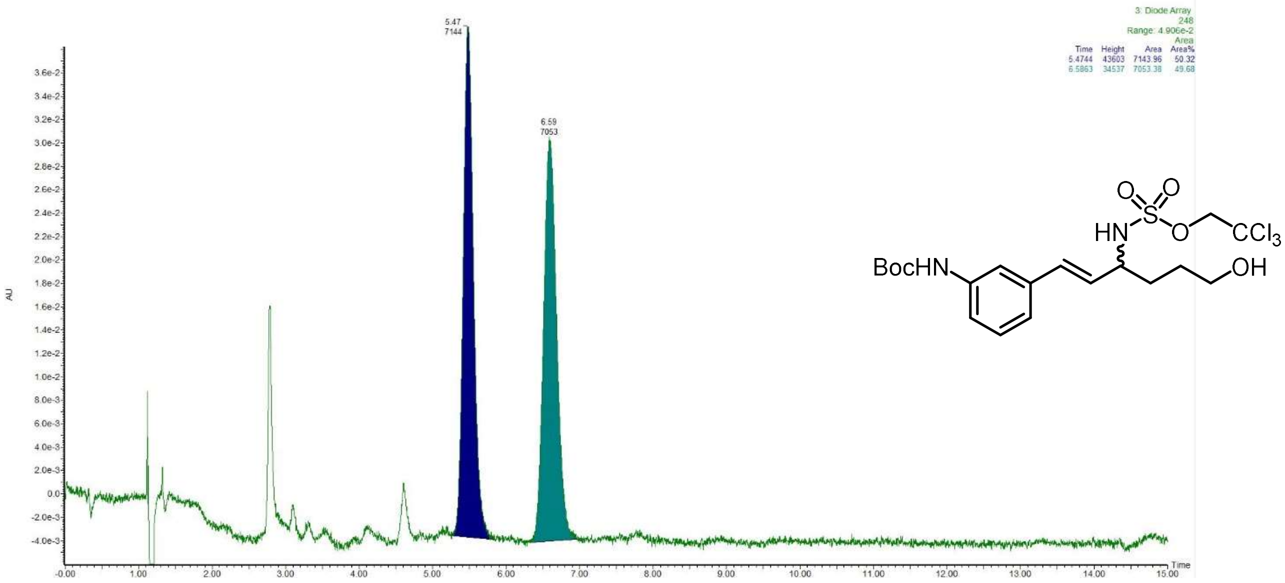

2,2,2-trichloroethyl (R,E)-(6-hydroxy-1-(2-methoxyphenyl)hex-1-en-3-yl)sulfamate (2k)

SFC CHIRALPAK IG (CO_2:MeOH = 80:20, 2.5 ml min^{-1}, 40 °C, 248 nm) t_R = 3.45 min (minor), 3.68 min (major) indicated 87% *ee.*

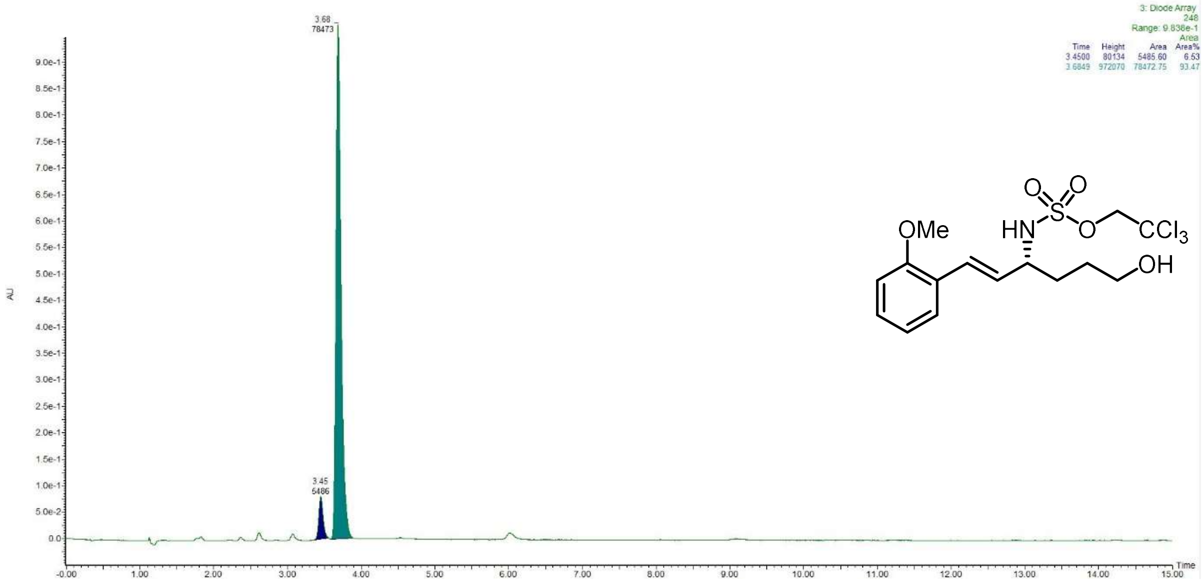

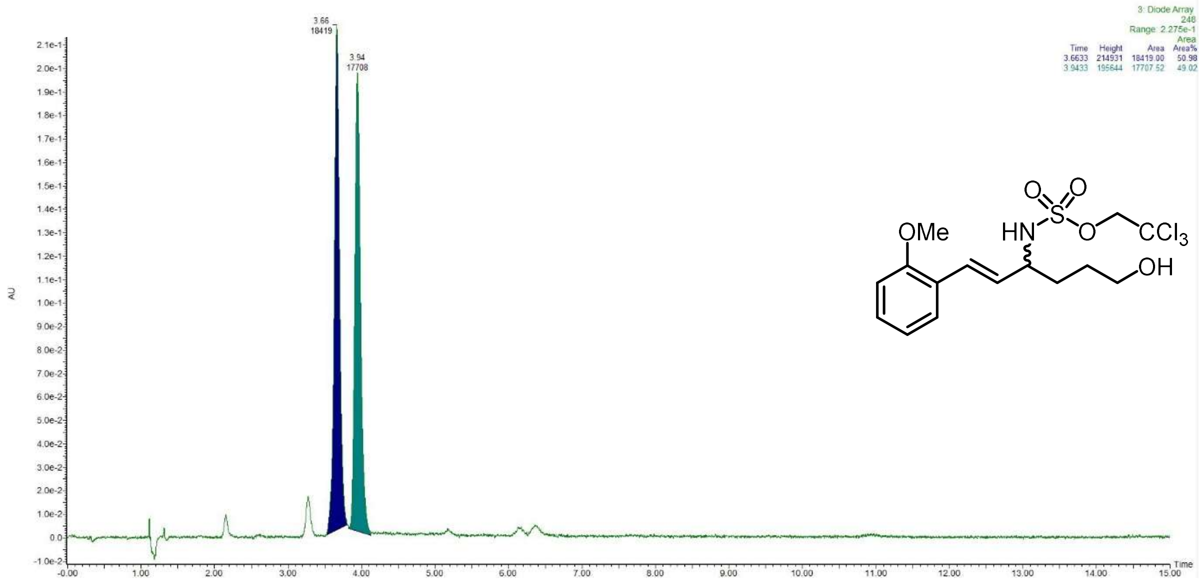

2,2,2-trichloroethyl (R,E)-(6-hydroxy-1-(o-tolyl)hex-1-en-3-yl)sulfamate (2l)

SFC CHIRALPAK IG (CO_2:MeOH = 90:10, 2.5 ml min^{-1}, 40 °C, 248 nm) t_R = 8.64 min (minor), 9.71 min (major) indicated 90% *ee.*

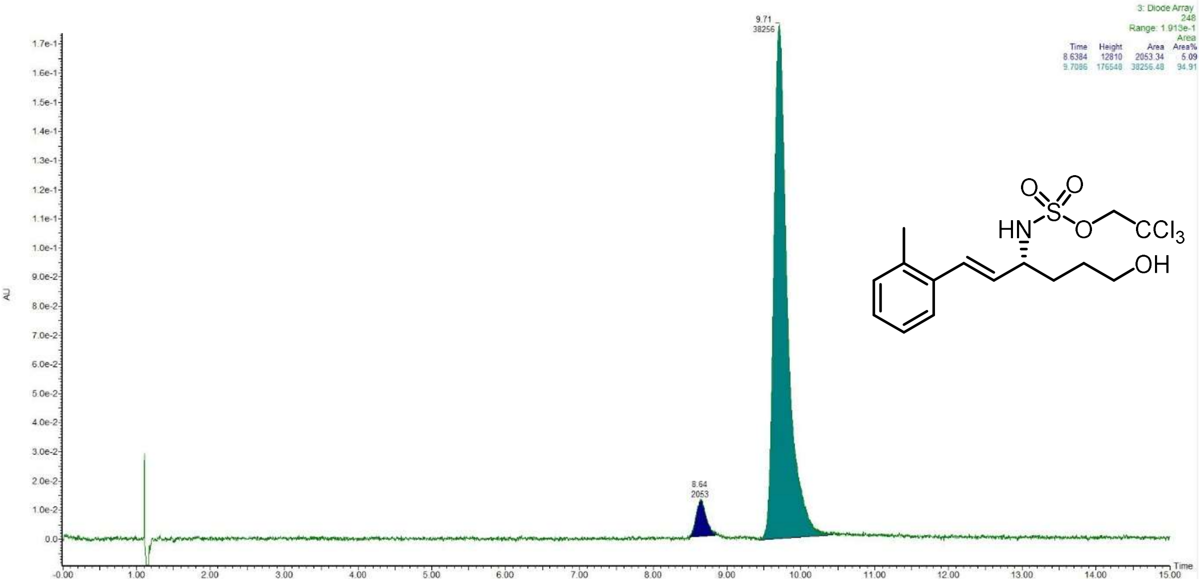

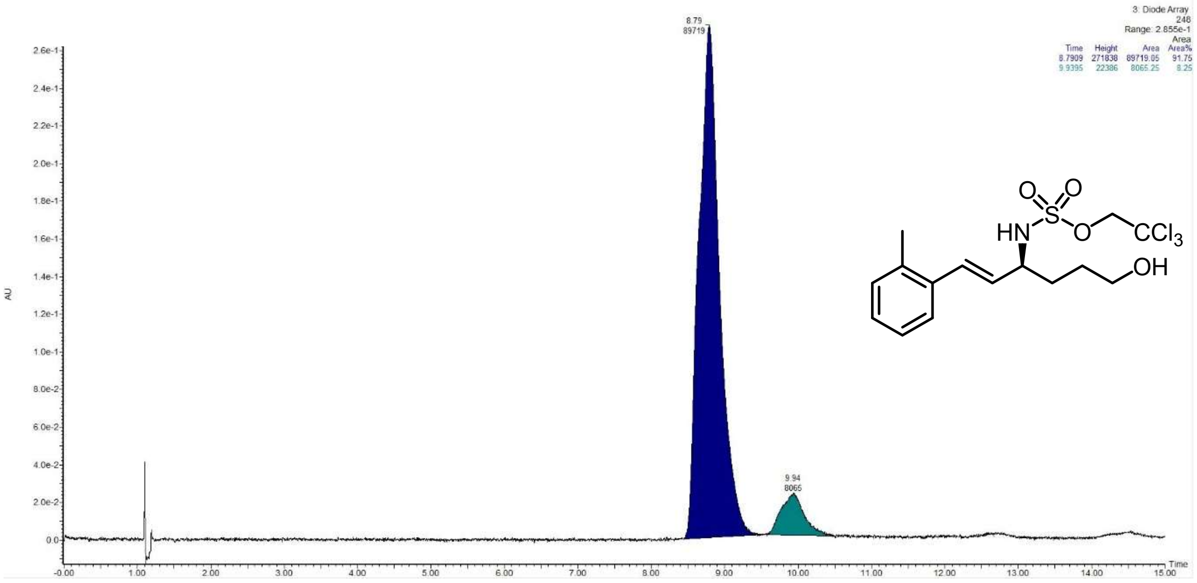

2,2,2-trichloroethyl (R,E)-(1-(2-chlorophenyl)-6-hydroxyhex-1-en-3-yl)sulfamate (2m)

SFC CHIRALPAK IG (CO_2:MeOH = 80:20, 2.5 ml min^{-1}, 40 °C, 248 nm) t_R = 3.53 min (minor), 3.83 min (major) indicated 91% *ee.*

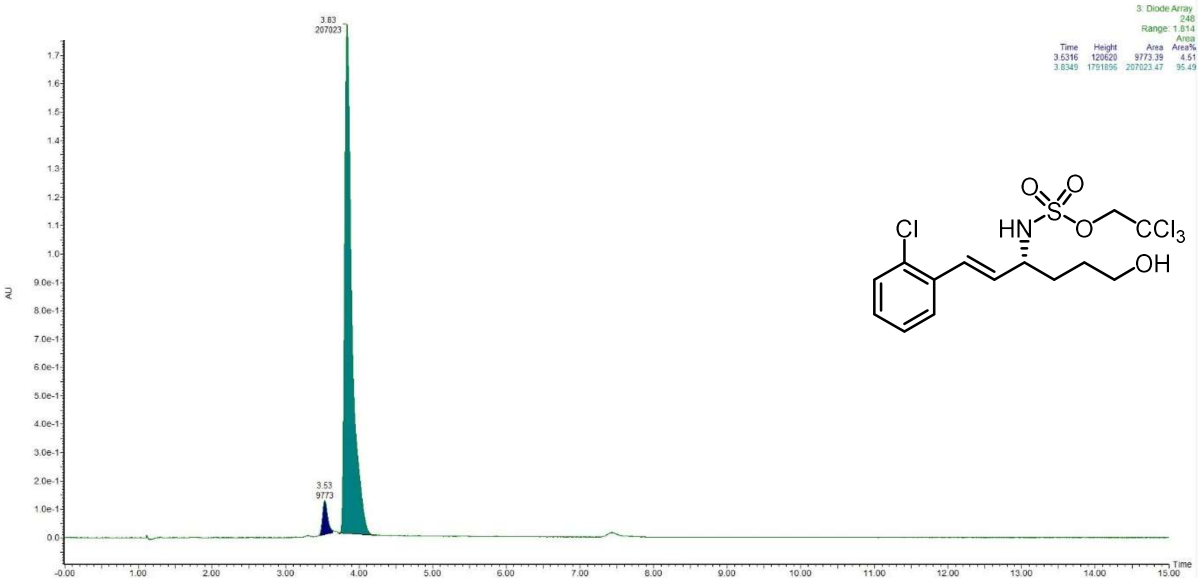

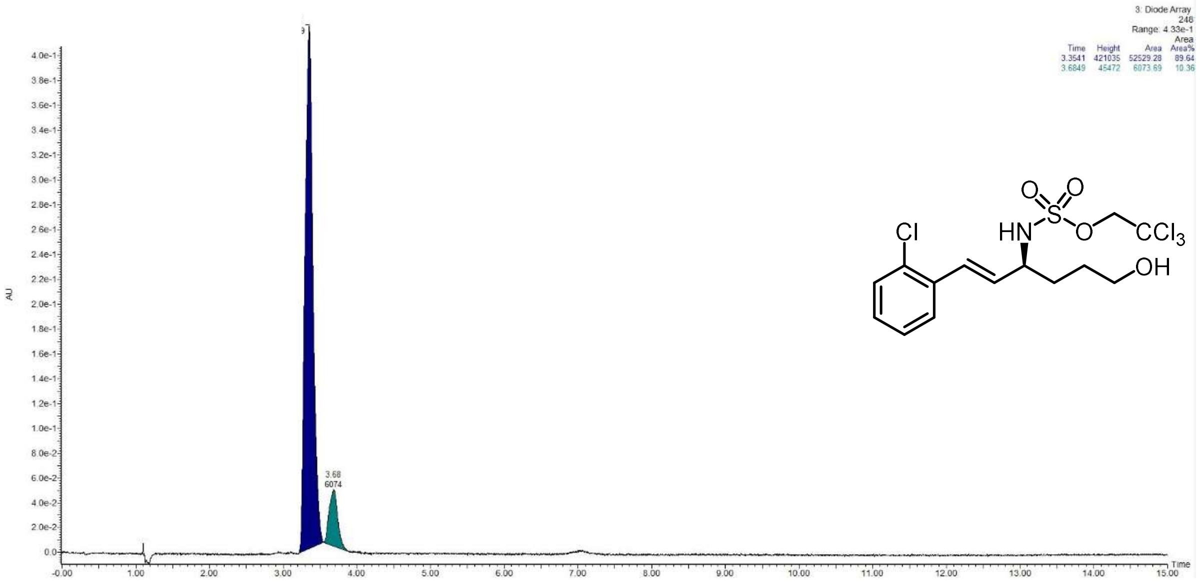

2,2,2-trichloroethyl (R,E)-(1-(2-bromophenyl)-6-hydroxyhex-1-en-3-yl)sulfamate (2n)

SFC CHIRALPAK IG (CO_2:MeOH = 80:20, 2.5 ml min^{-1}, 40 °C, 248 nm) t_R = 3.17 min (minor), 3.47 min (major) indicated 90% *ee.*

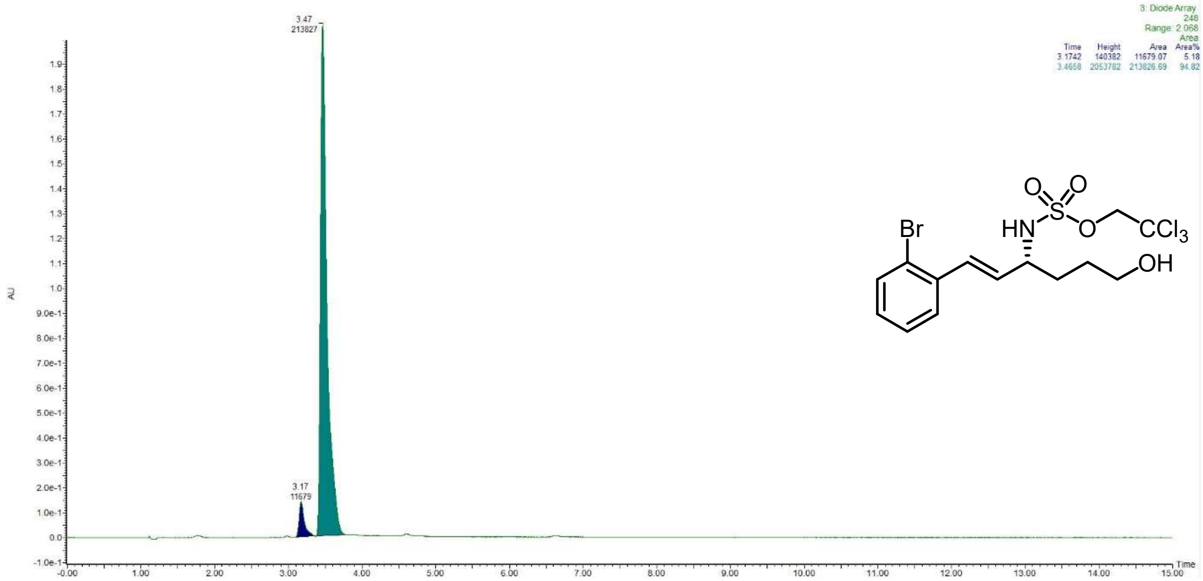

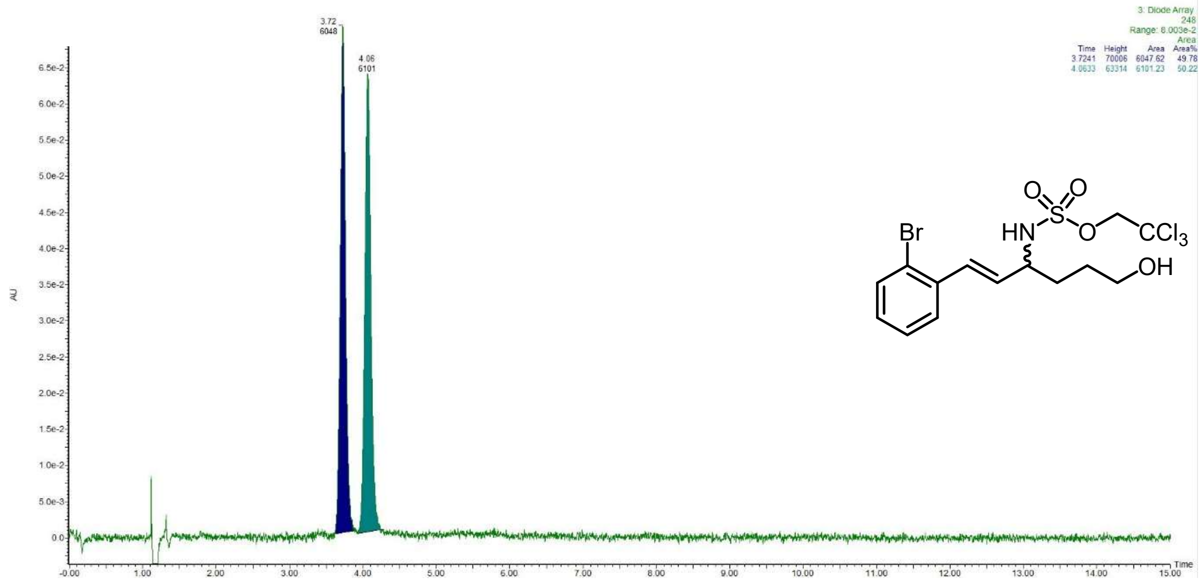

2,2,2-trichloroethyl (R,E)-(1-(2-ethylphenyl)-6-hydroxyhex-1-en-3-yl)sulfamate (2o)

SFC CHIRALPAK IJ (CO_2:MeOH = 92:08, 2.5 ml min^{-1}, 40 °C, 248 nm) t_R = 7.34 min (major), 7.70 min (minor) indicated 92% *ee.*

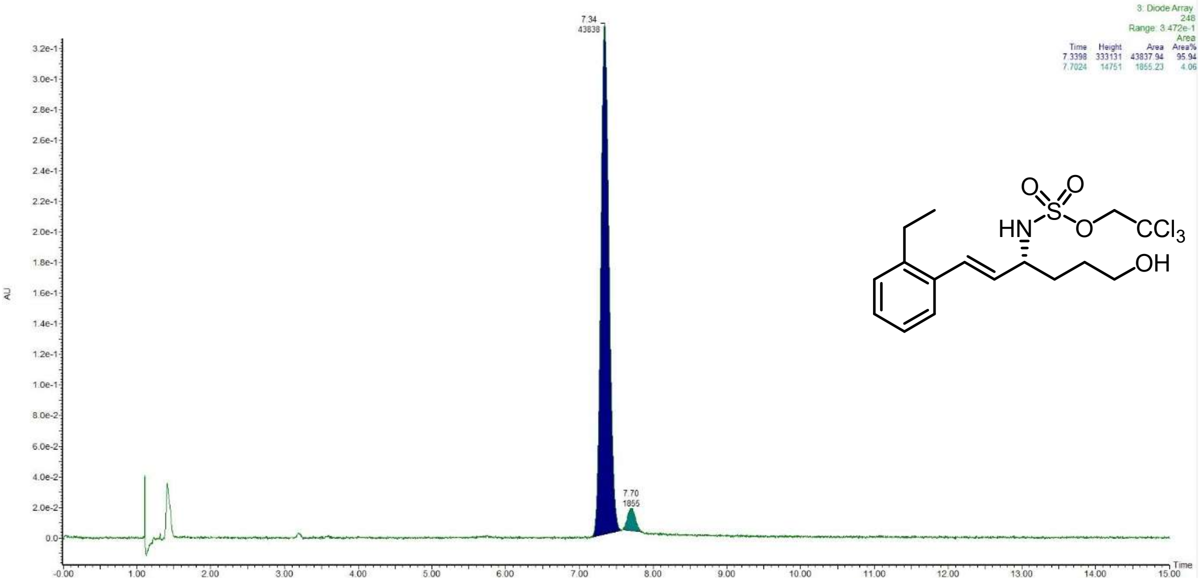

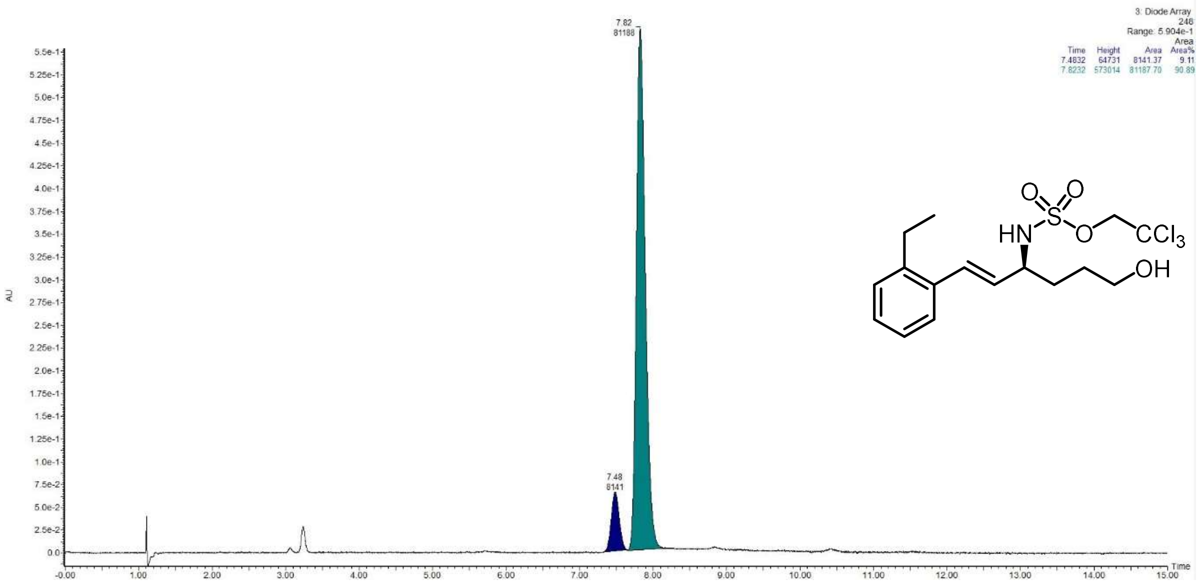

2,2,2-trichloroethyl (R,E)-(6-hydroxy-1-mesitylhex-1-en-3-yl)sulfamate (2p)

SFC CHIRALPAK IG (CO_2:MeOH = 80:20, 2.5 ml min^{-1}, 40 °C, 248 nm) t_R = 3.00 min (minor), 3.56 min (major) indicated 90% *ee.*

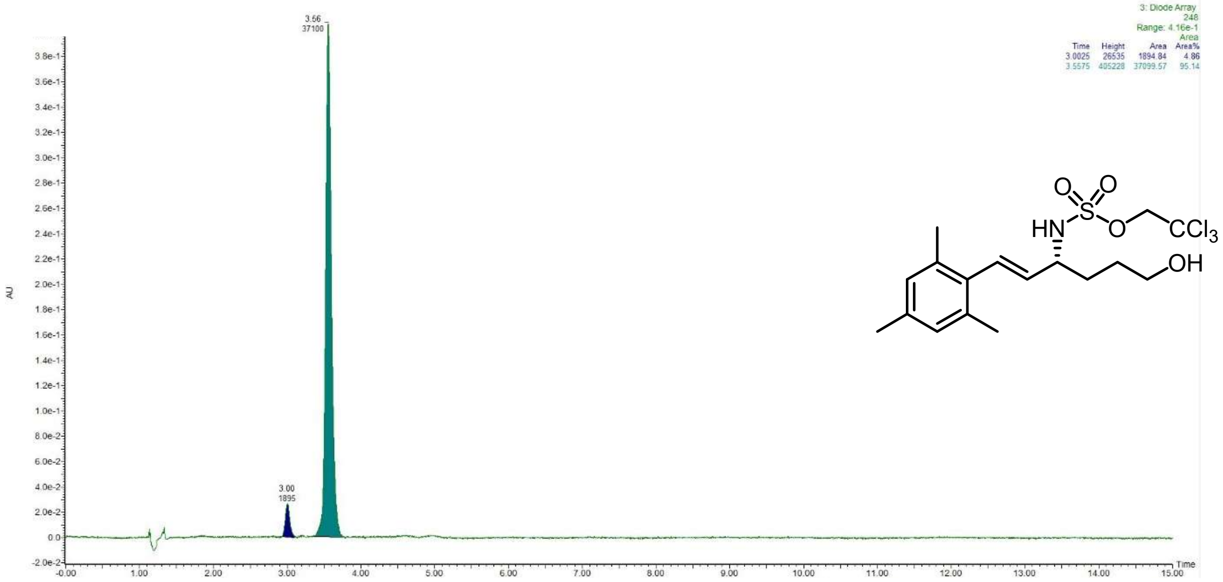

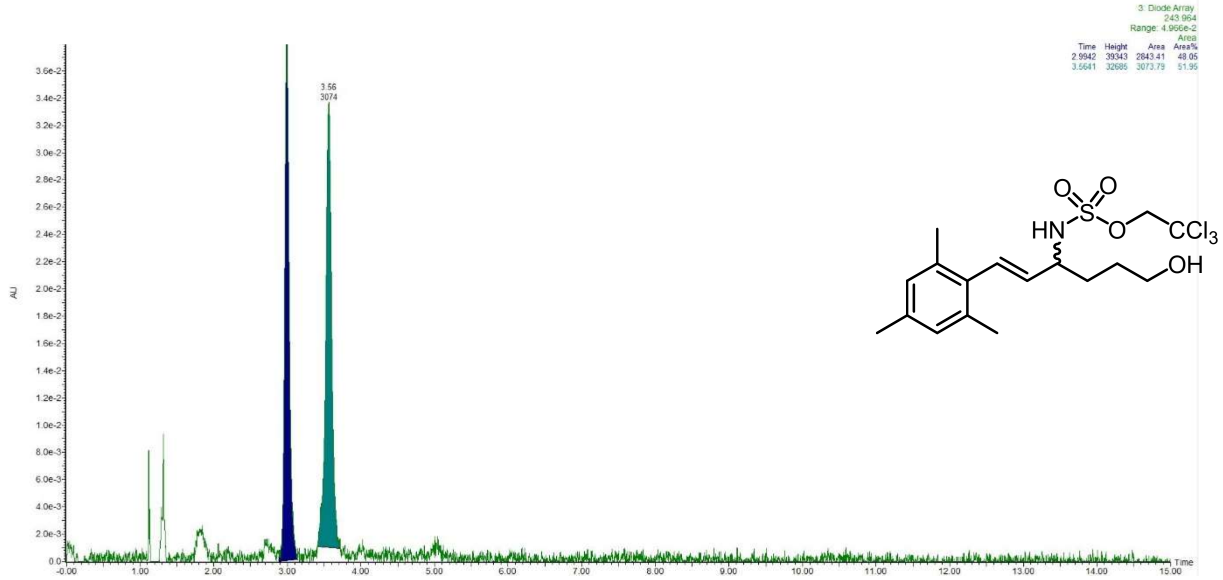

2,2,2-trichloroethyl (R,E)-(6-hydroxy-6-methyl-1-phenylhept-1-en-3-yl)sulfamate (2q)

SFC CHIRALPAK IC (CO_2:MeOH = 90:10, 2.5 ml min^{-1}, 40 °C, 248 nm) t_R = 4.88 min (minor), 5.17 min (major) indicated 84% *ee.*

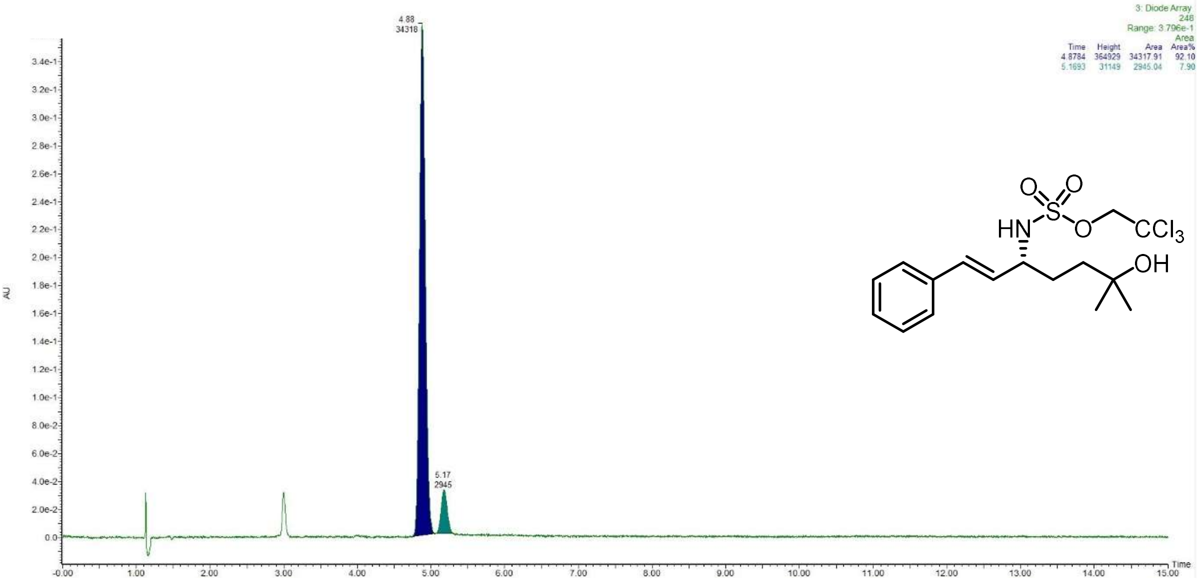

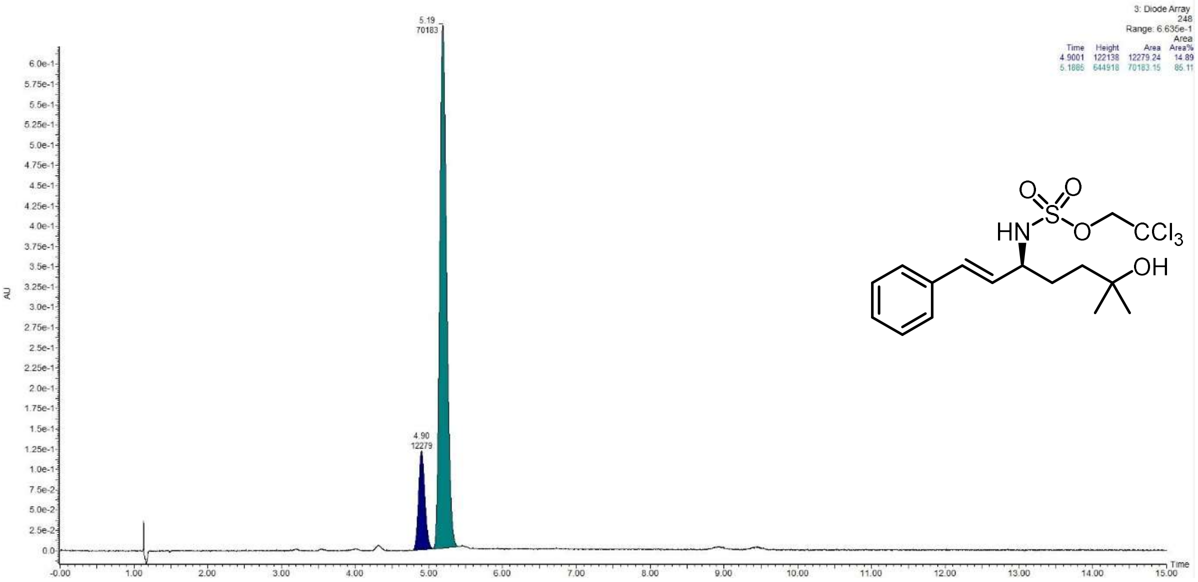

2,2,2-trichloroethyl (R,E)-(1-hydroxydec-5-en-4-yl)sulfamate (2t)

HPLC CHIRALPAK IH (Hexane: ^{I}PrOH, 92:08, 1.25 mL min^{-1}, 40 °C, 203 nm) t_R = 12.8 (major), 15.3 (minor) minutes indicated 75% *ee*.

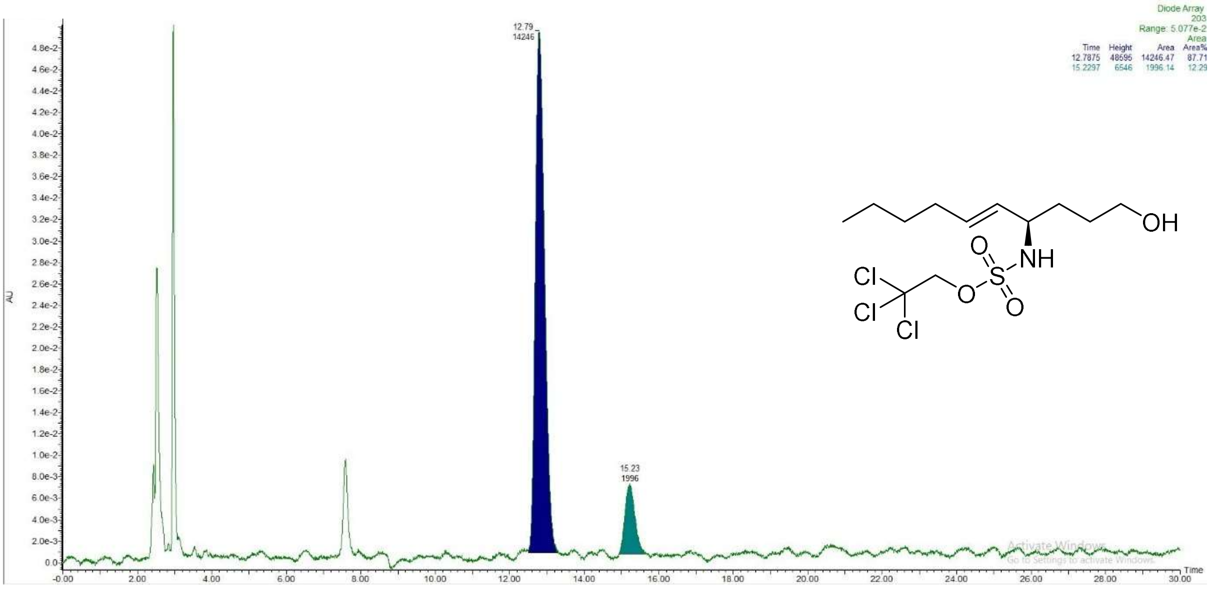

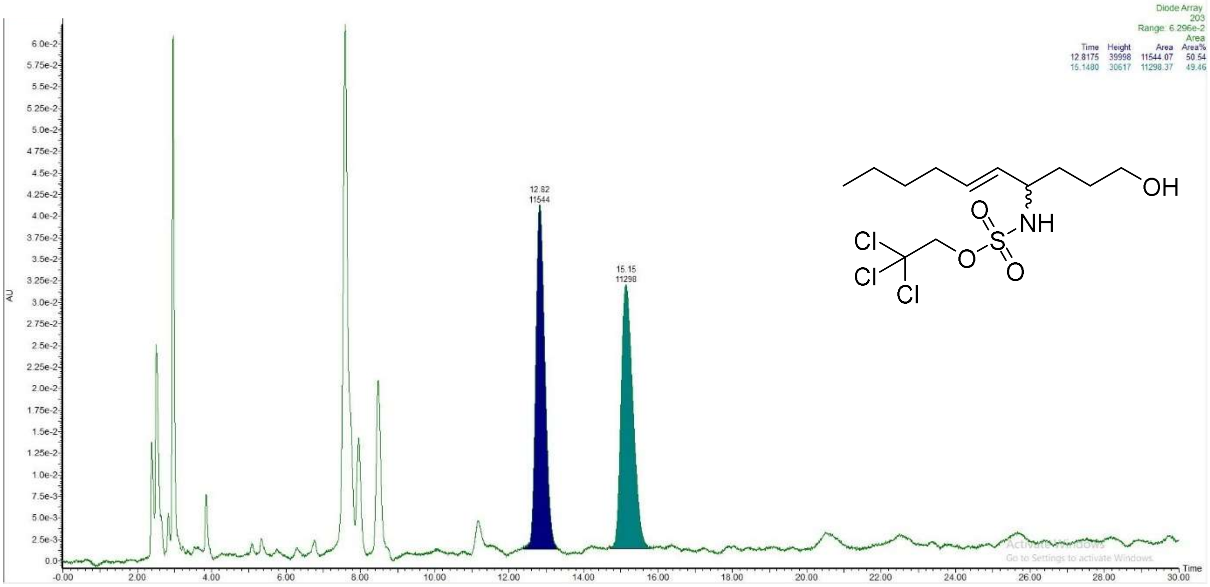

2,2,2-trichloroethyl (R,E)-(6-methoxy-1-phenylhex-1-en-3-yl)sulfamate (10)

HPLC CHIRALPAK IH (Hexane: ^{i}PrOH, 90:10, 1.25 mL min^{-1}, 40 °C, 250 nm) t_R = 12.1 (major), 13.2 (minor) minutes indicated 81% *ee*.

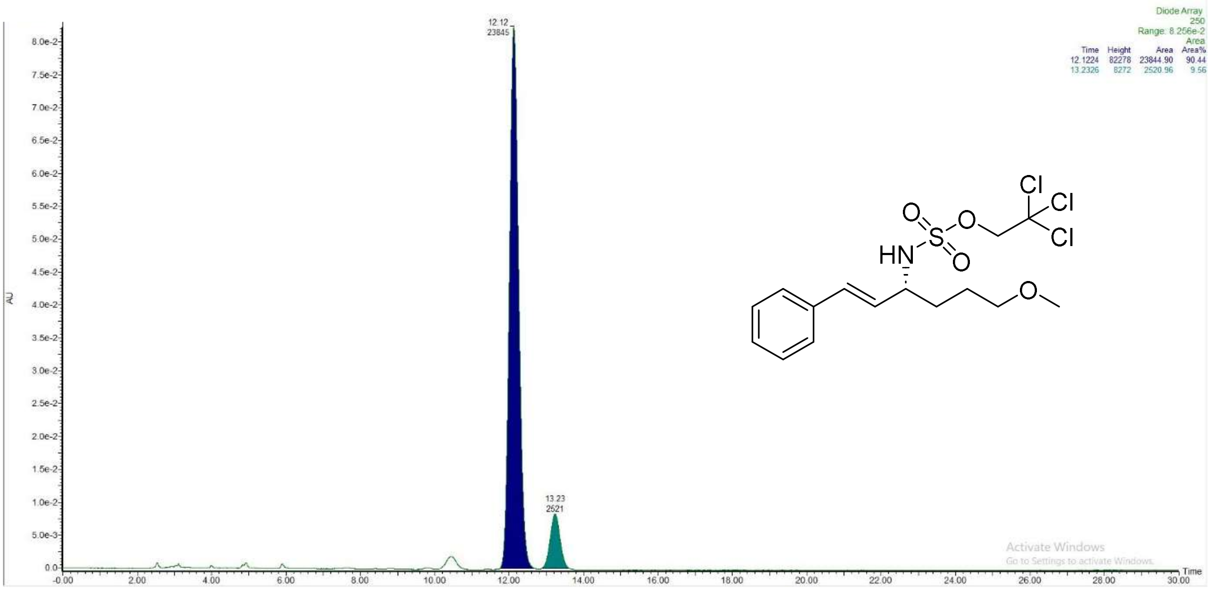

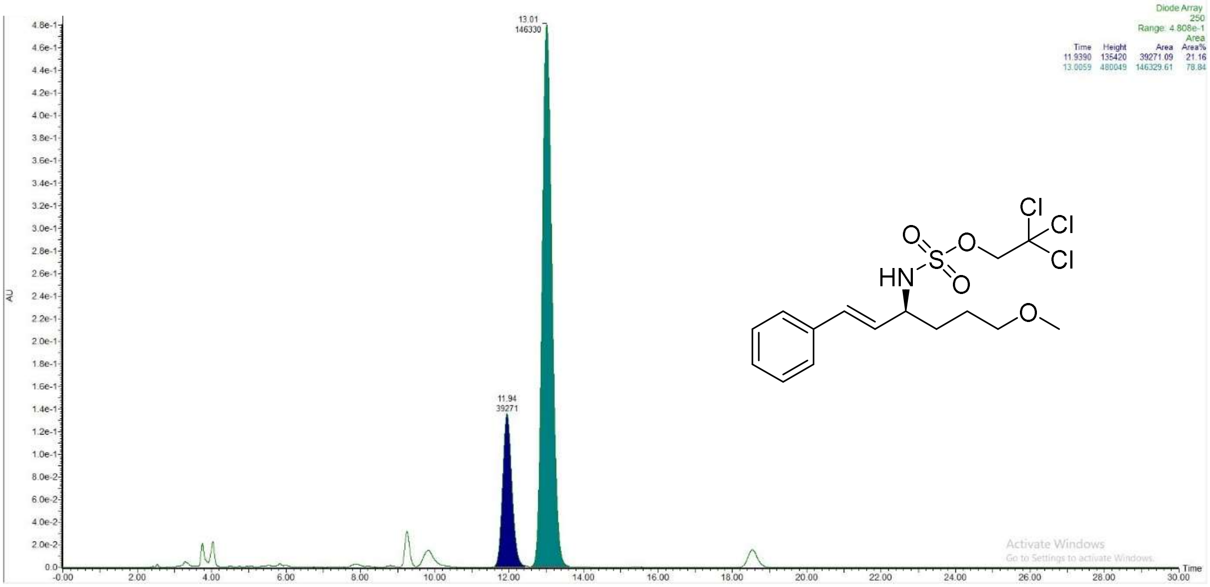

2,2,2-trichloroethyl (R,E)-(6-oxo-1-phenyl-6-(phenylamino)hex-1-en-3-yl)sulfamate (11)

SFC DCHIRALPAK IG (CO_2:MeOH, 75:25, 2.50 mL min^{-1}, 40 °C, 251) t_R = 7.67 (major), 11.65 (minor) minutes indicated 44% *ee*.

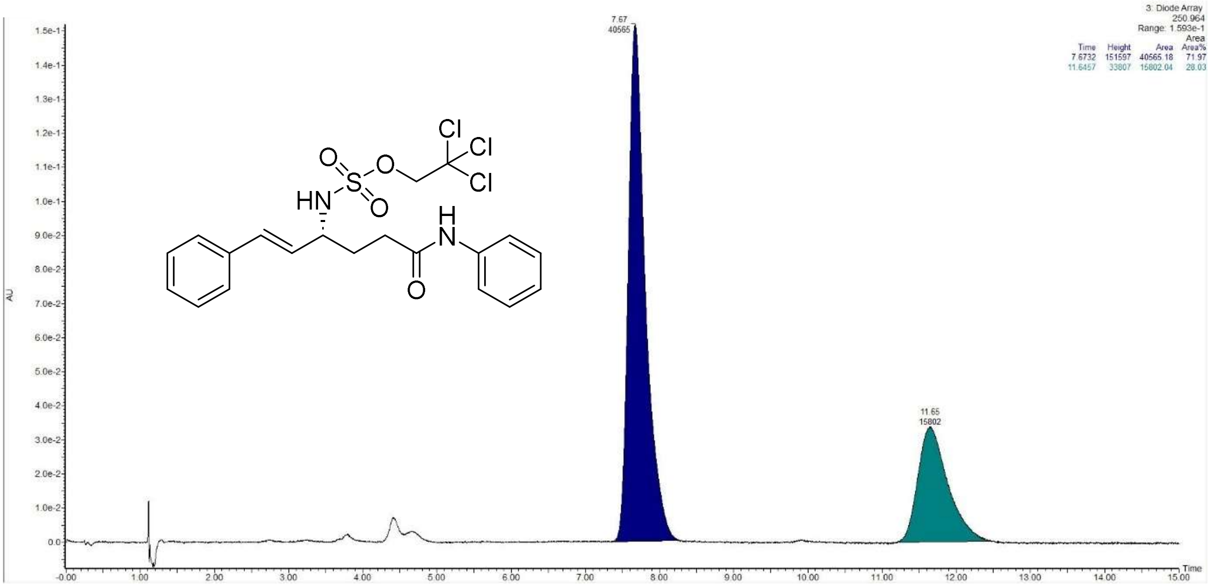

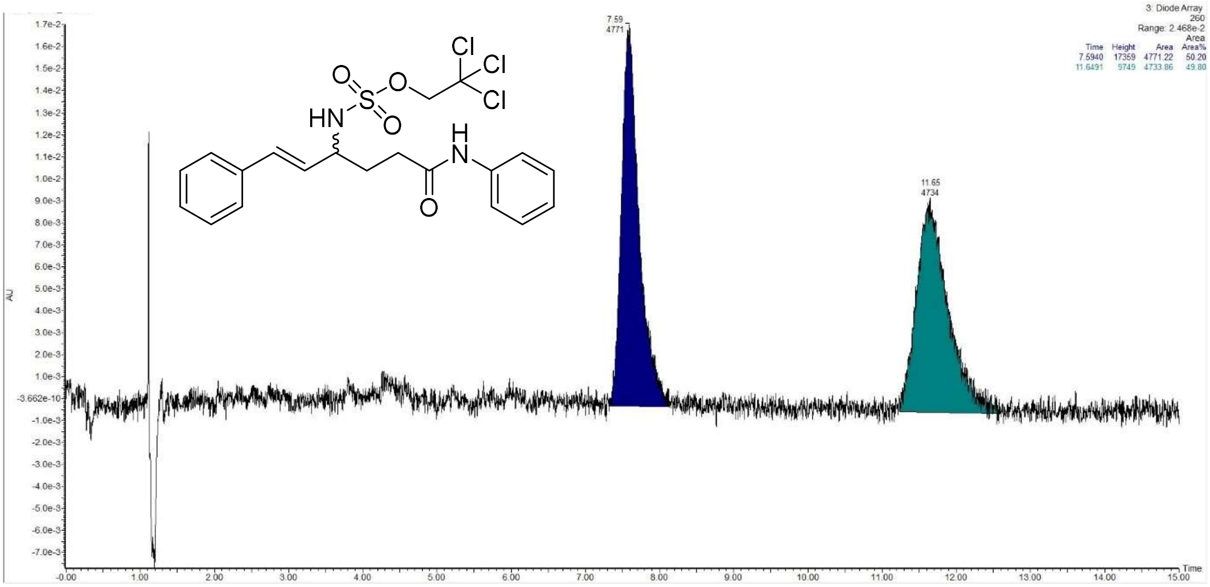

2,2,2-trichloroethyl (R,E)-(5-hydroxy-1-phenylpent-1-en-3-yl)sulfamate (6a)

SFC CHIRALPAK IG (CO_2:MeOH = 80:20, 2.5 ml min^{-1}, 40 °C, 248 nm) t_R = 4.17 min (minor), 6.54 min (major) indicated 93% *ee.*

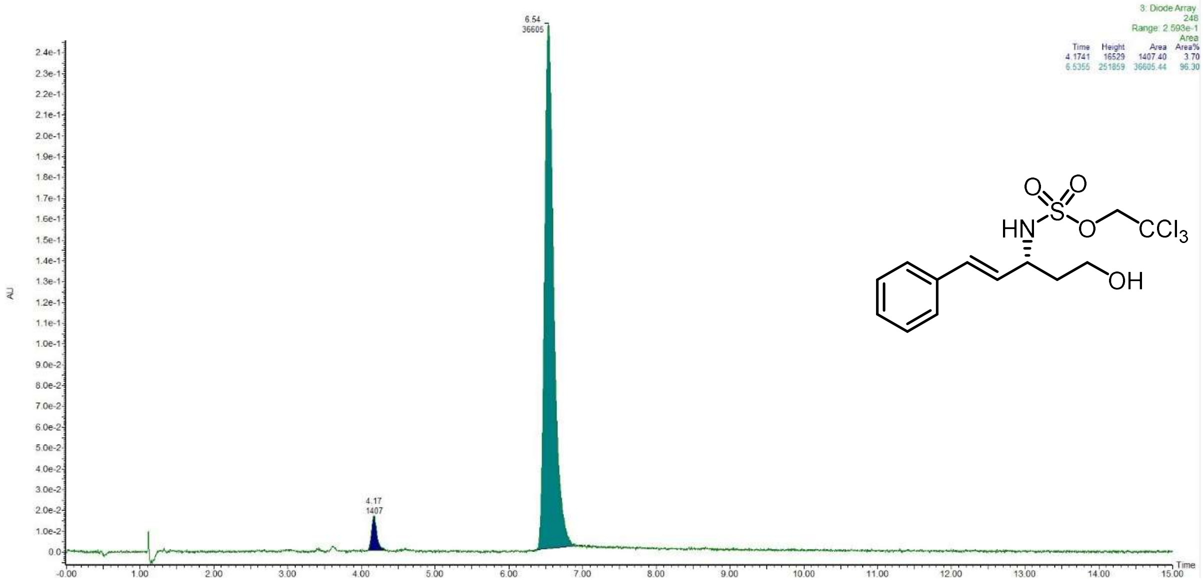

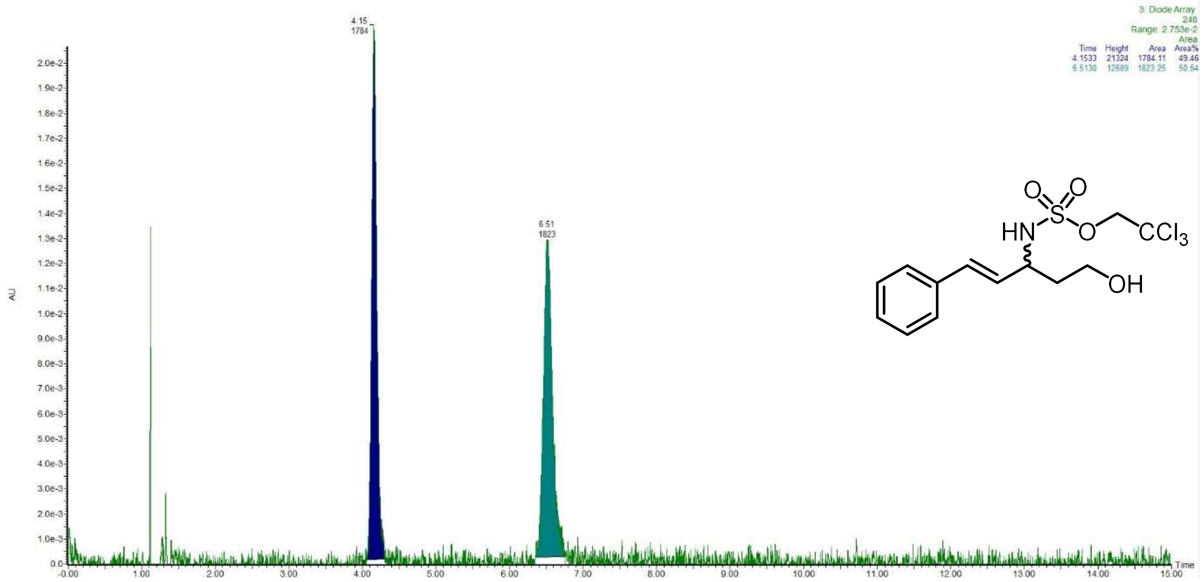

2,2,2-trichloroethyl (R,E)-(5-hydroxy-1-(2-isopropylphenyl)pent-1-en-3-yl)sulfamate (6b)

SFC CHIRALPAK IJ (CO_2:MeOH = 92:08, 2.5 ml min^{-1}, 40 °C, 248 nm) t_R = 5.32 min (major), 5.55 min (minor) indicated 88% *ee.*

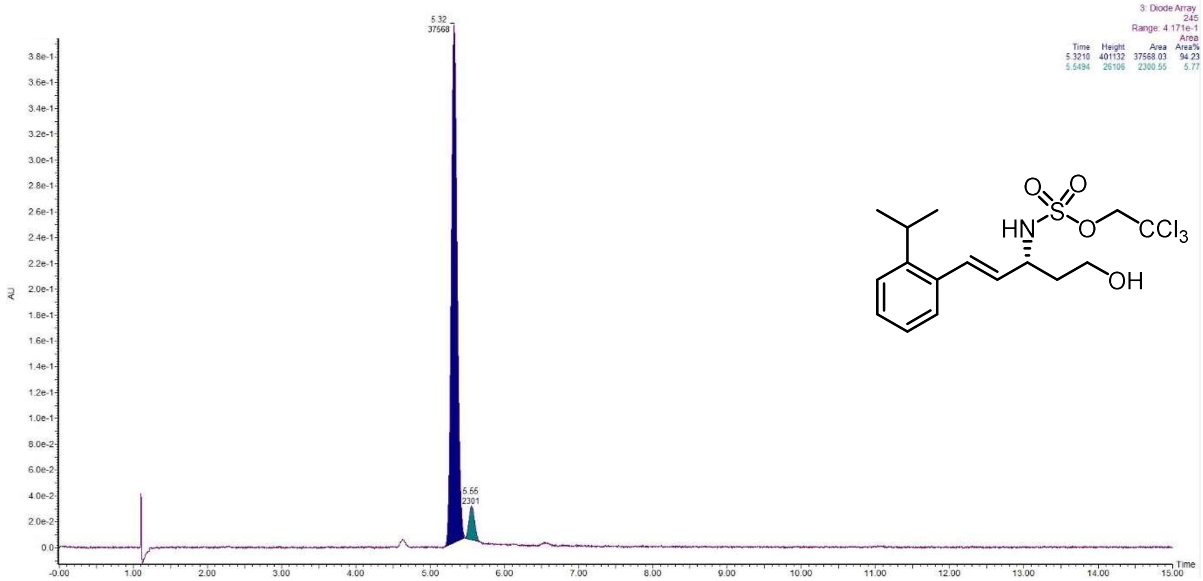

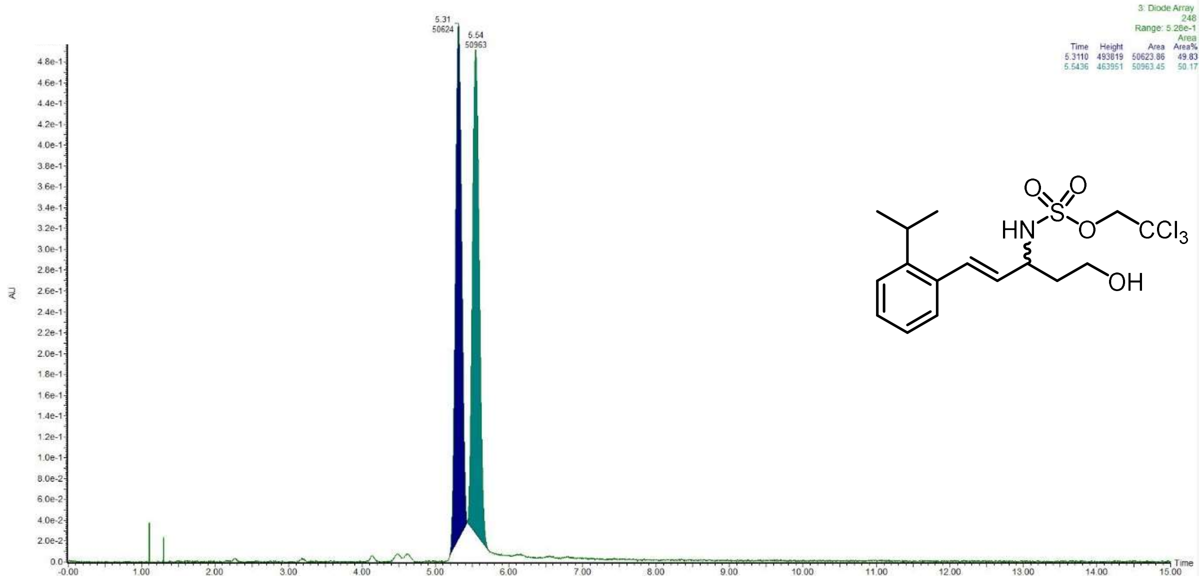

2,2,2-trichloroethyl (R,E)-(5-hydroxy-1-(3-methoxyphenyl)pent-1-en-3-yl)sulfamate (6c)

SFC CHIRALPAK IJ (CO_2:MeOH = 90:10, 2.5 ml min^{-1}, 40 °C, 248 nm) t_R = 8.07 min (minor), 8.93 min (major) indicated 85% *ee.*

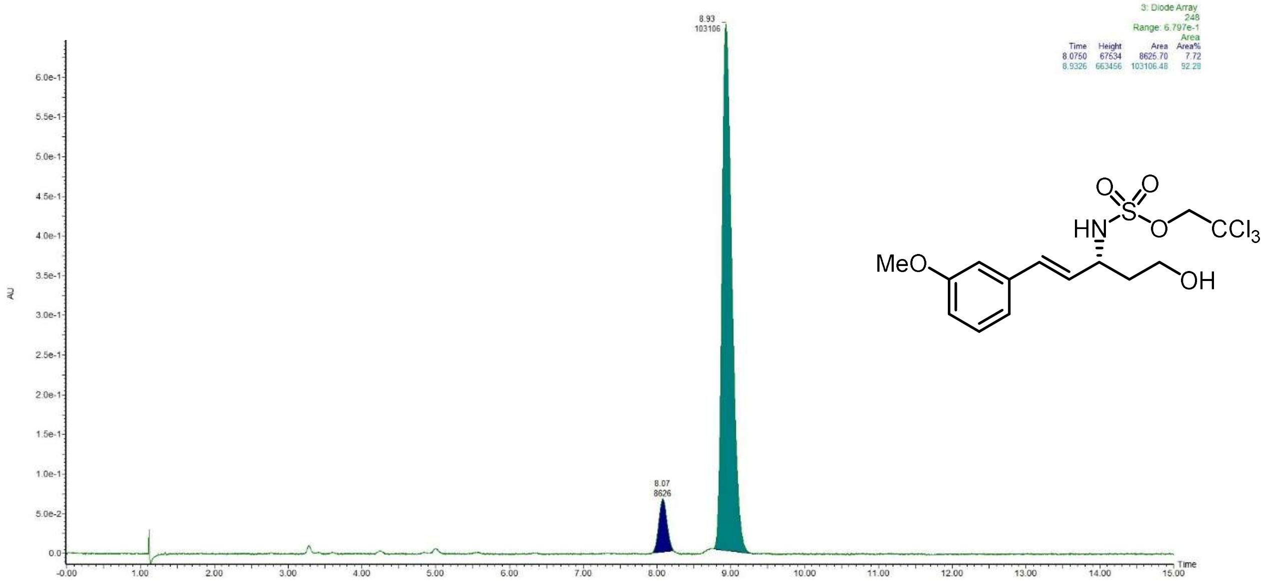

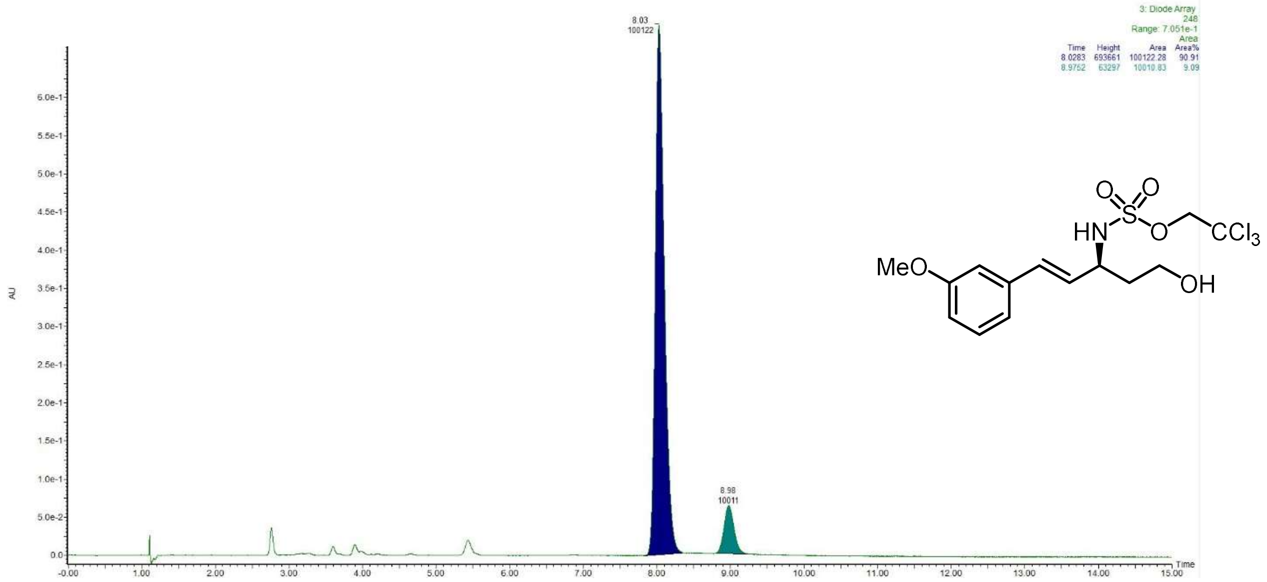

2,2,2-trichloroethyl (R,E)-(6-hydroxy-1-(p-tolyl)hex-1-en-3-yl)sulfamate (6d)

SFC CHIRALPAK IG (CO_2:MeOH = 90:10, 1.25 ml min^{-1}, 40 °C, 248 nm) t_R = 6.97 min (major), 7.46 min (minor) indicated 90% *ee.*

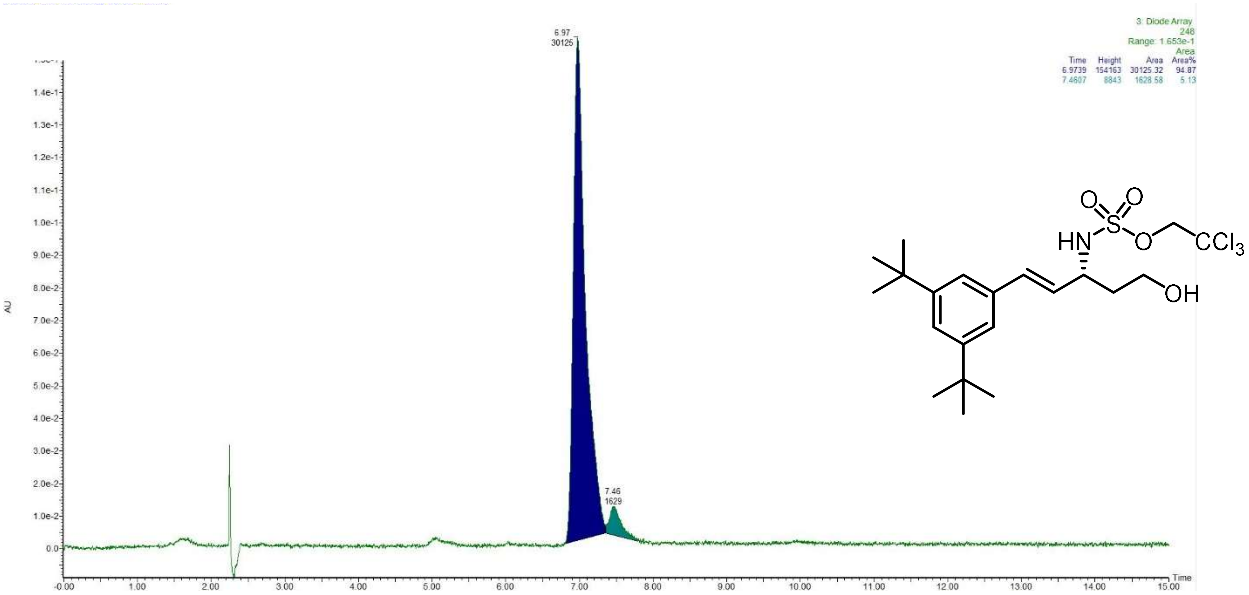

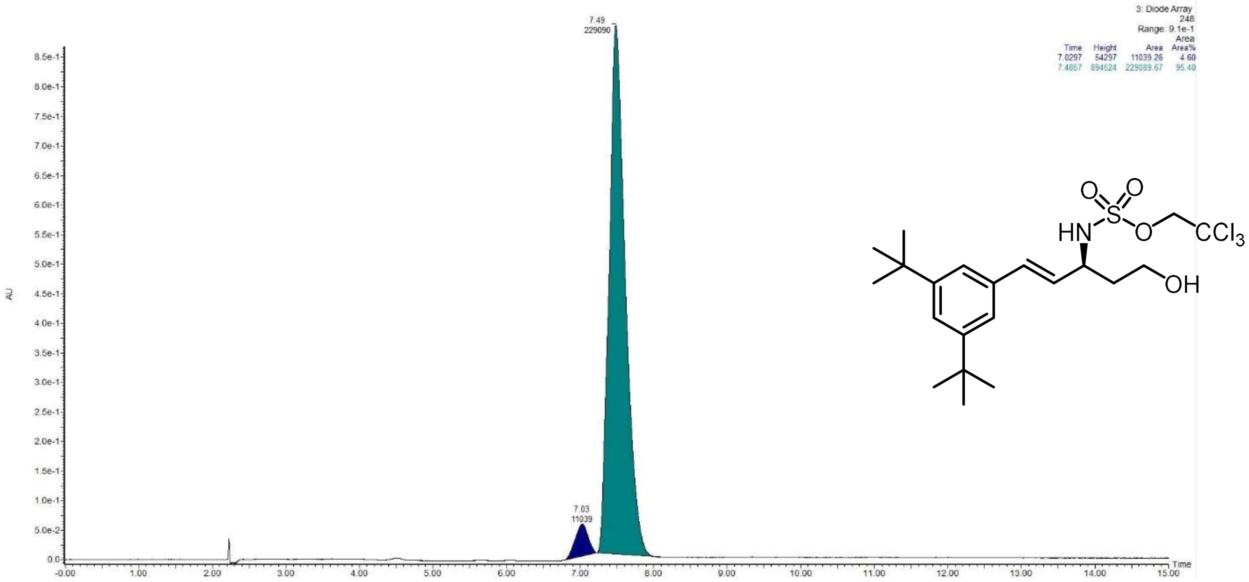

Methyl (R,E)-2-(5-hydroxy-3-(((2,2,2-trichloroethoxy)sulfonyl)amino)pent-1-en-1-yl)benzoate (6e)

SFC CHIRALPAK IG (CO_2:MeOH = 80:20, 2.5 ml min^{-1}, 40 °C, 248 nm) t_R = 4.19 min (major), 5.51 min (minor) indicated 94% *ee.*

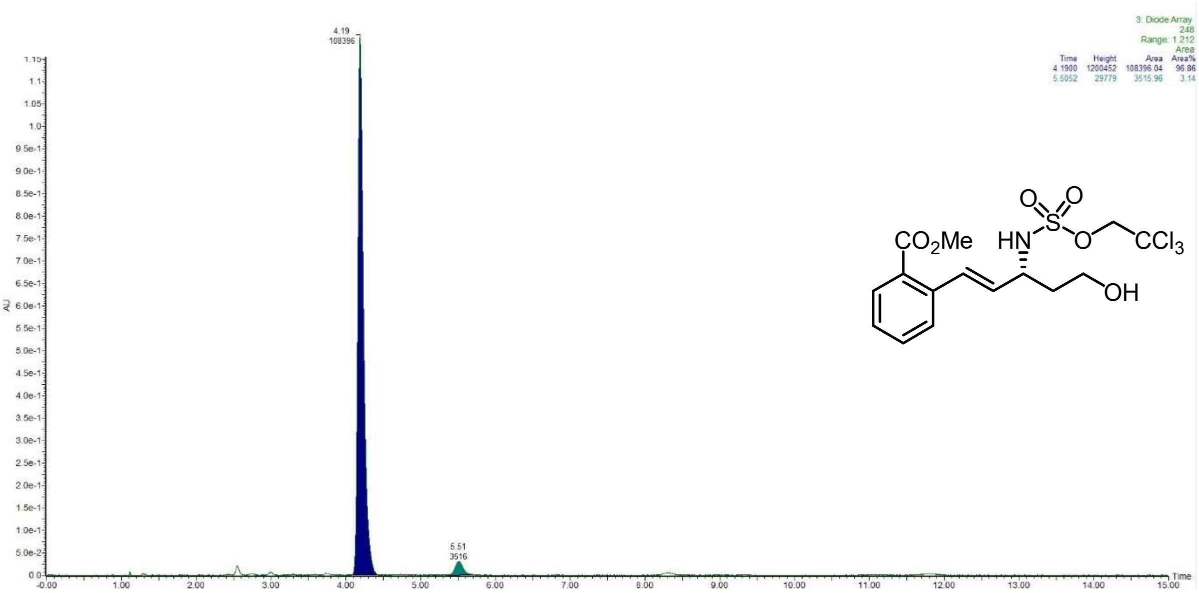

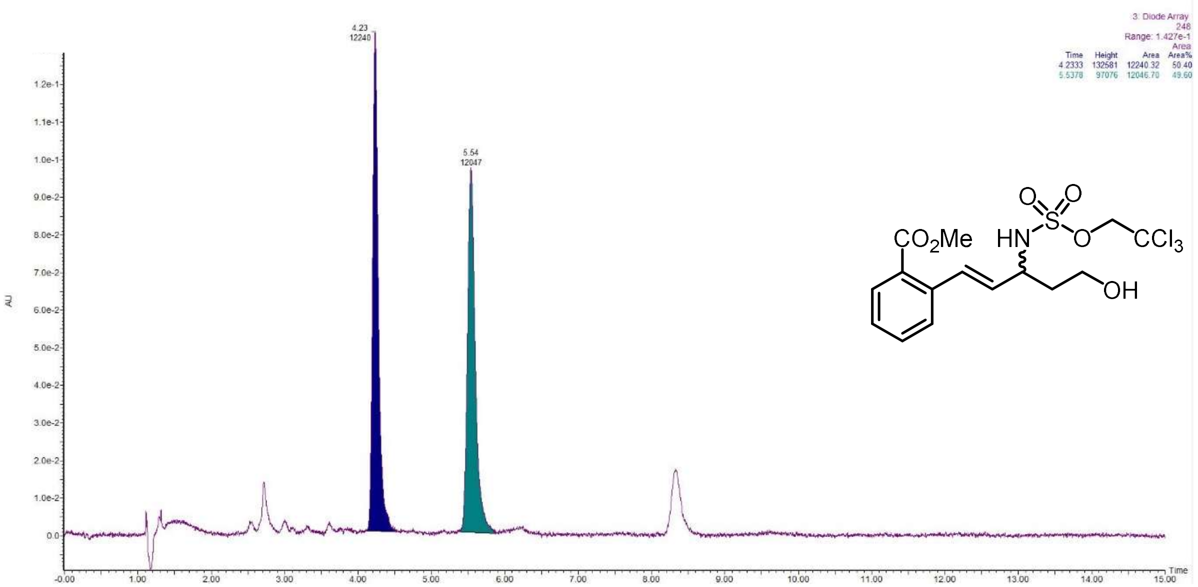

2,2,2-trichloroethyl (R,E)-(5-hydroxy-5-methyl-1-phenylhex-1-en-3-yl)sulfamate (6f)

SFC CHIRALPAK IG (CO_2:MeOH = 80:20, 2.5 ml min^{-1}, 40 °C, 248 nm) t_R = 3.02 min (minor), 3.43 min (major) indicated 90% *ee.*

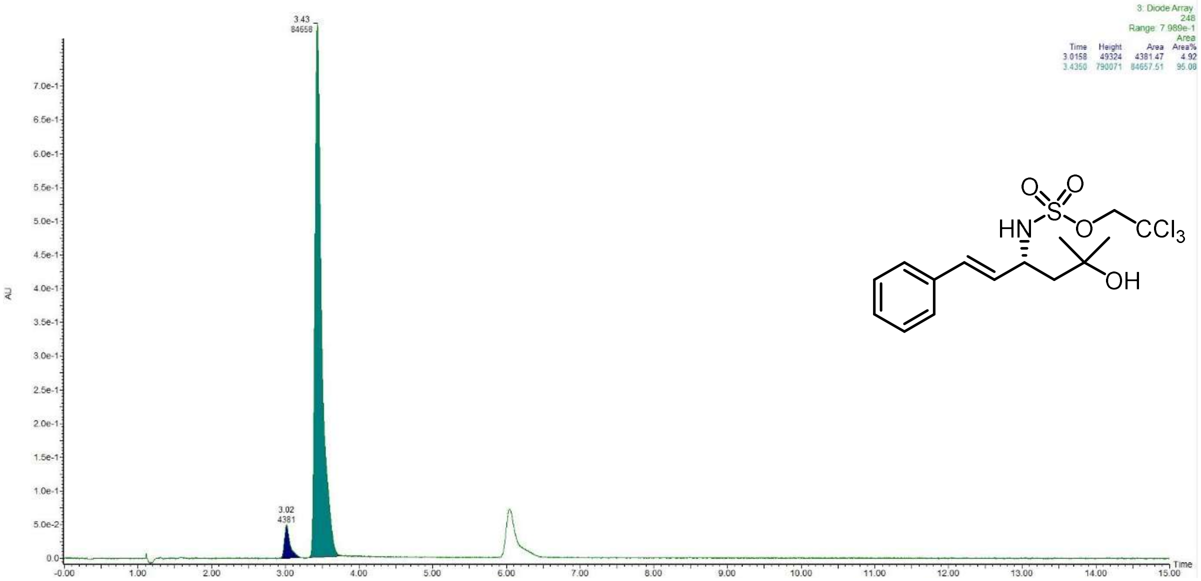

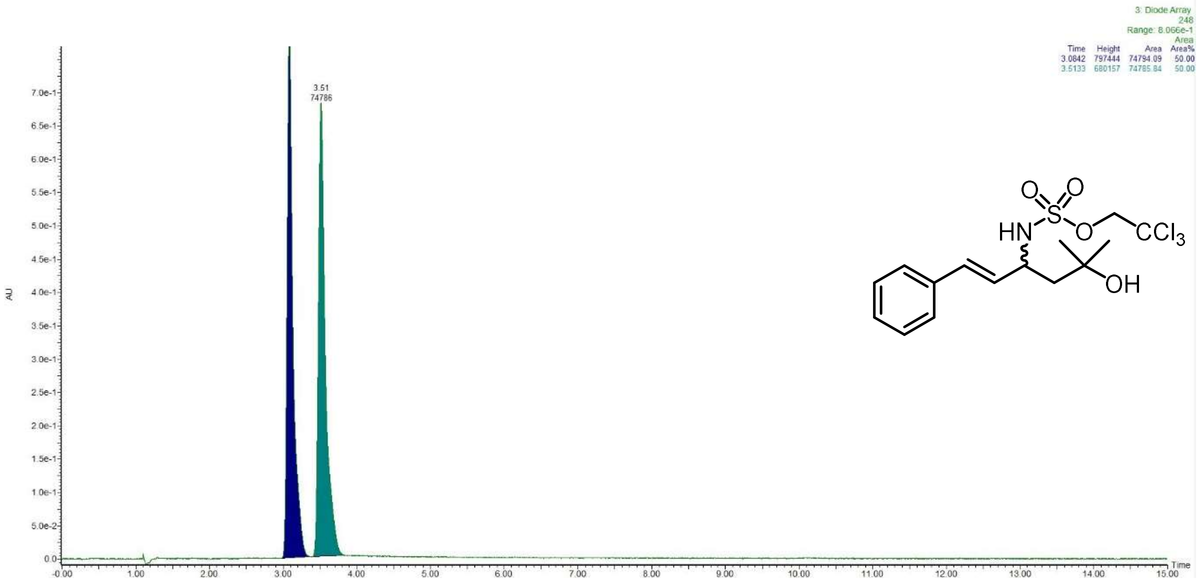

Methyl (S,E)-3-(2-(1-((2,2,2-trichloroethoxy)sulfonyl)pyrrolidin-2-yl)vinyl)benzoate

SFC CHIRALPAK IG (CO_2:MeOH = 80:20, 2.5 ml min^{-1}, 40 °C, 248 nm) t_R = 7.78 min (minor), 16.28 min (major) indicated 87% *ee.*

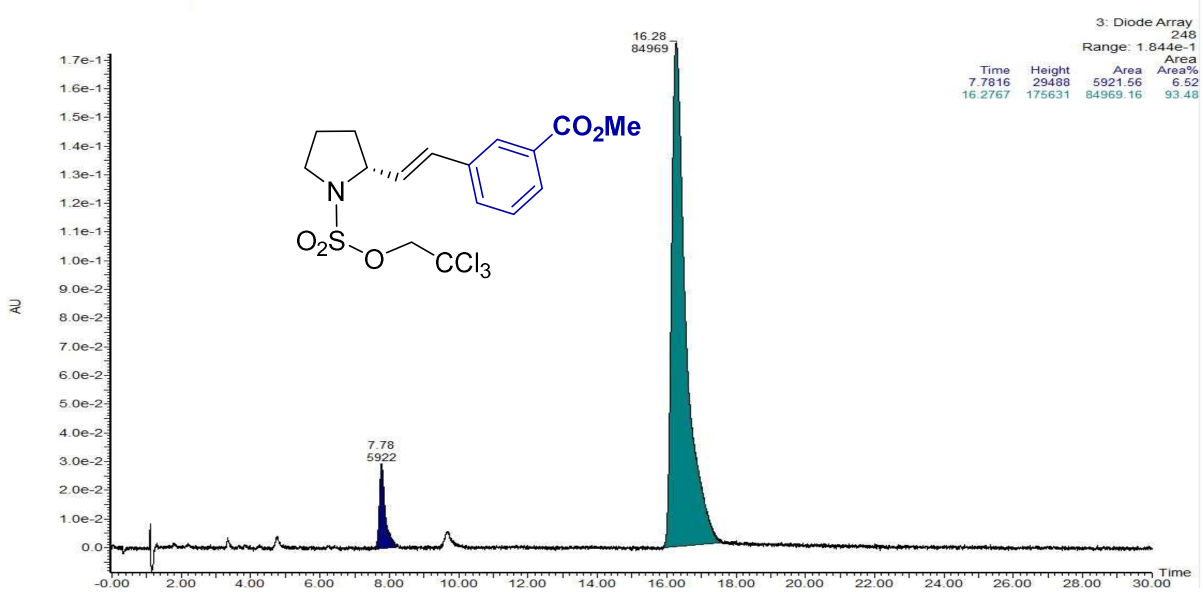

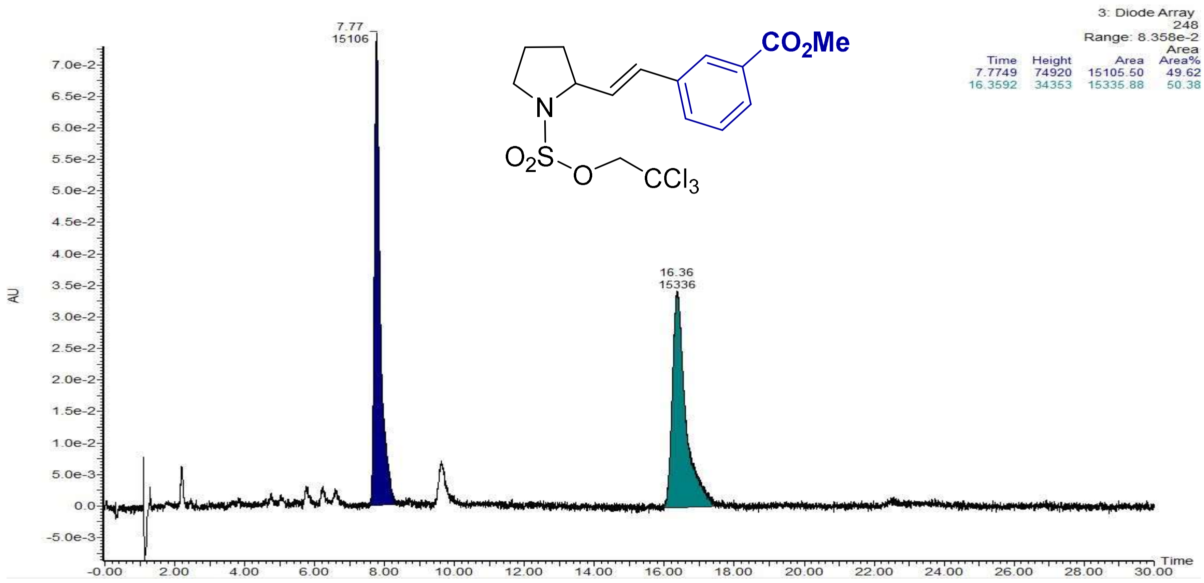

11. NMR Spectra

^{1}H NMR (500 MHz, $CDCl_3$) for ***tert-butyl(hex-5-yn-1-yloxy)dimethylsilane***

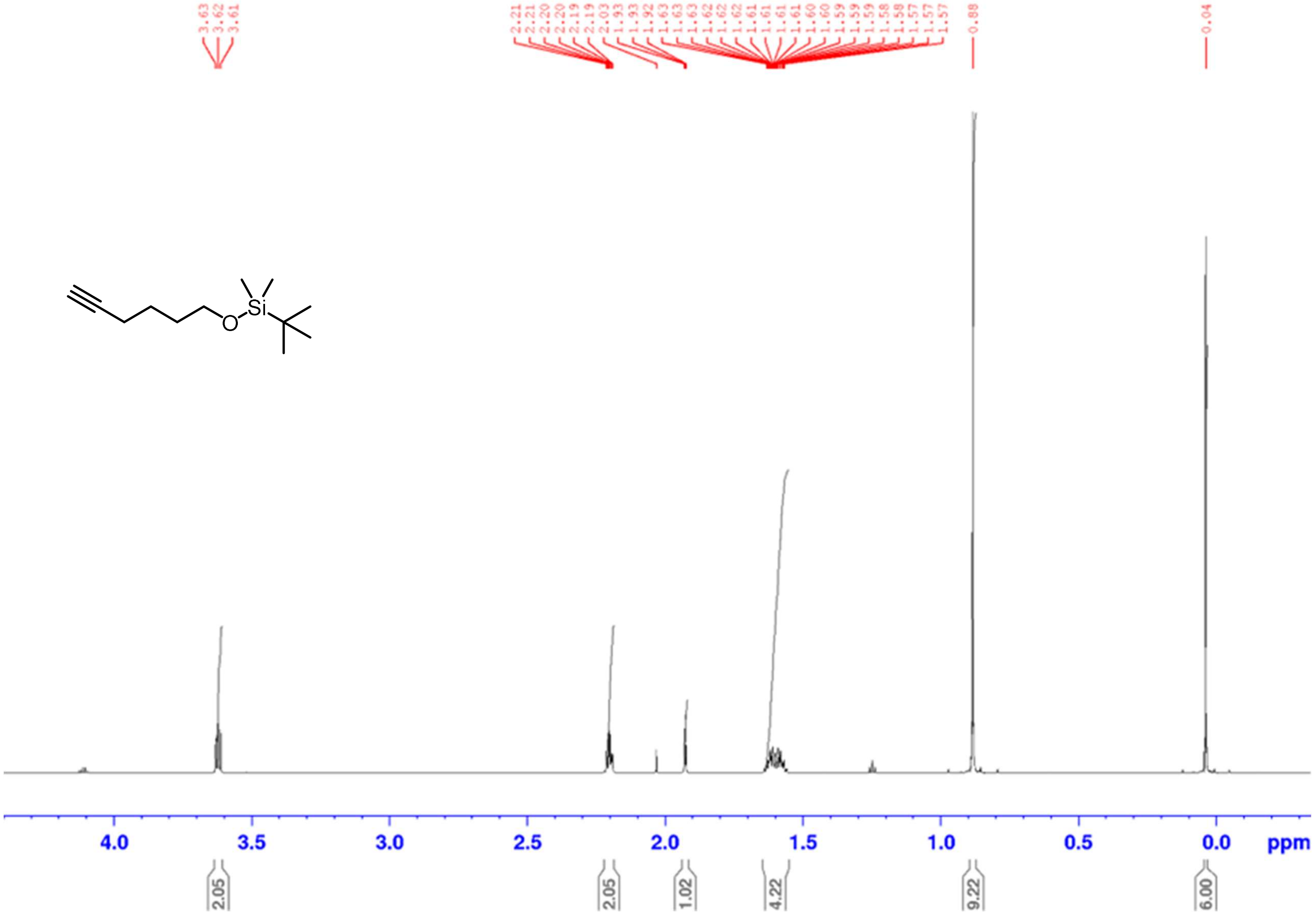

^{13}C NMR (126 MHz, $CDCl_3$) for ***tert-butyl(hex-5-yn-1-yloxy)dimethylsilane***

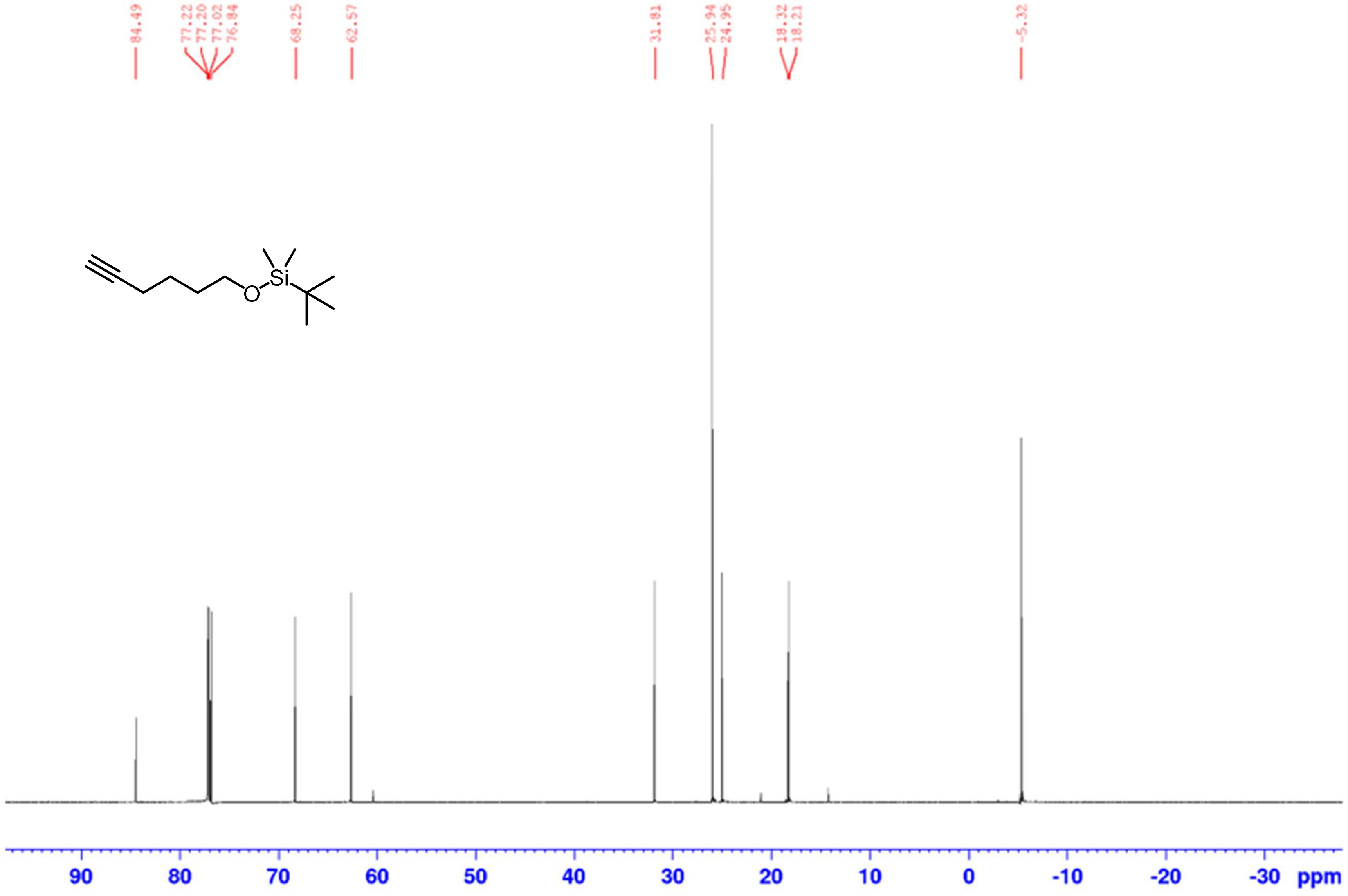

^{1}H NMR (700 MHz, $CDCl_3$) for ***(E)-tert-butyldimethyl((6-(4,4,5,5-tetramethyl-1,3,2-dioxaborolan-2-yl)hex-5-en-1-yl)oxy)silane***

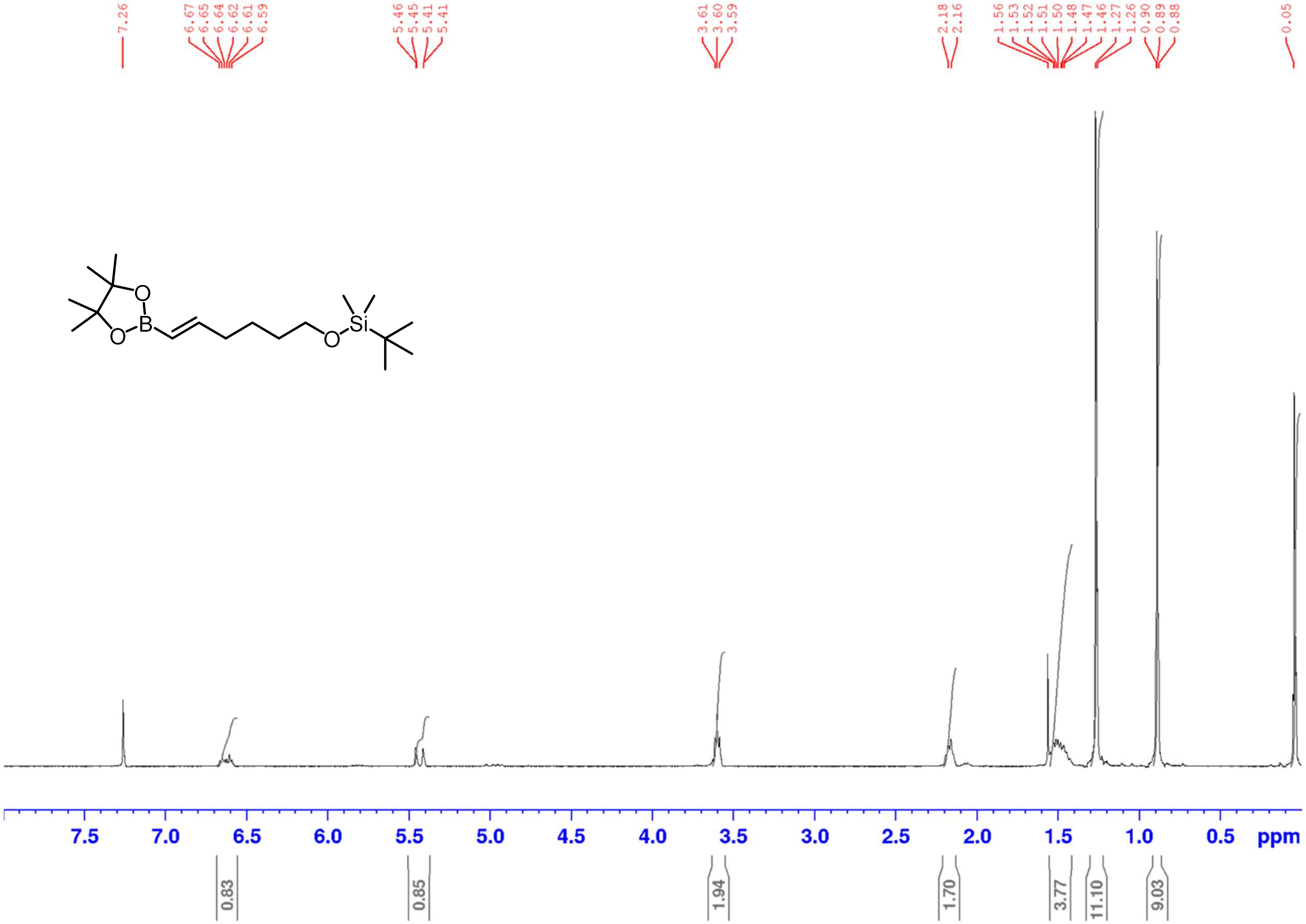

^{13}C NMR (176 MHz, $CDCl_3$) for ***(E)-tert-butyldimethyl((6-(4,4,5,5-tetramethyl-1,3,2-dioxaborolan-2-yl)hex-5-en-1-yl)oxy)silane***

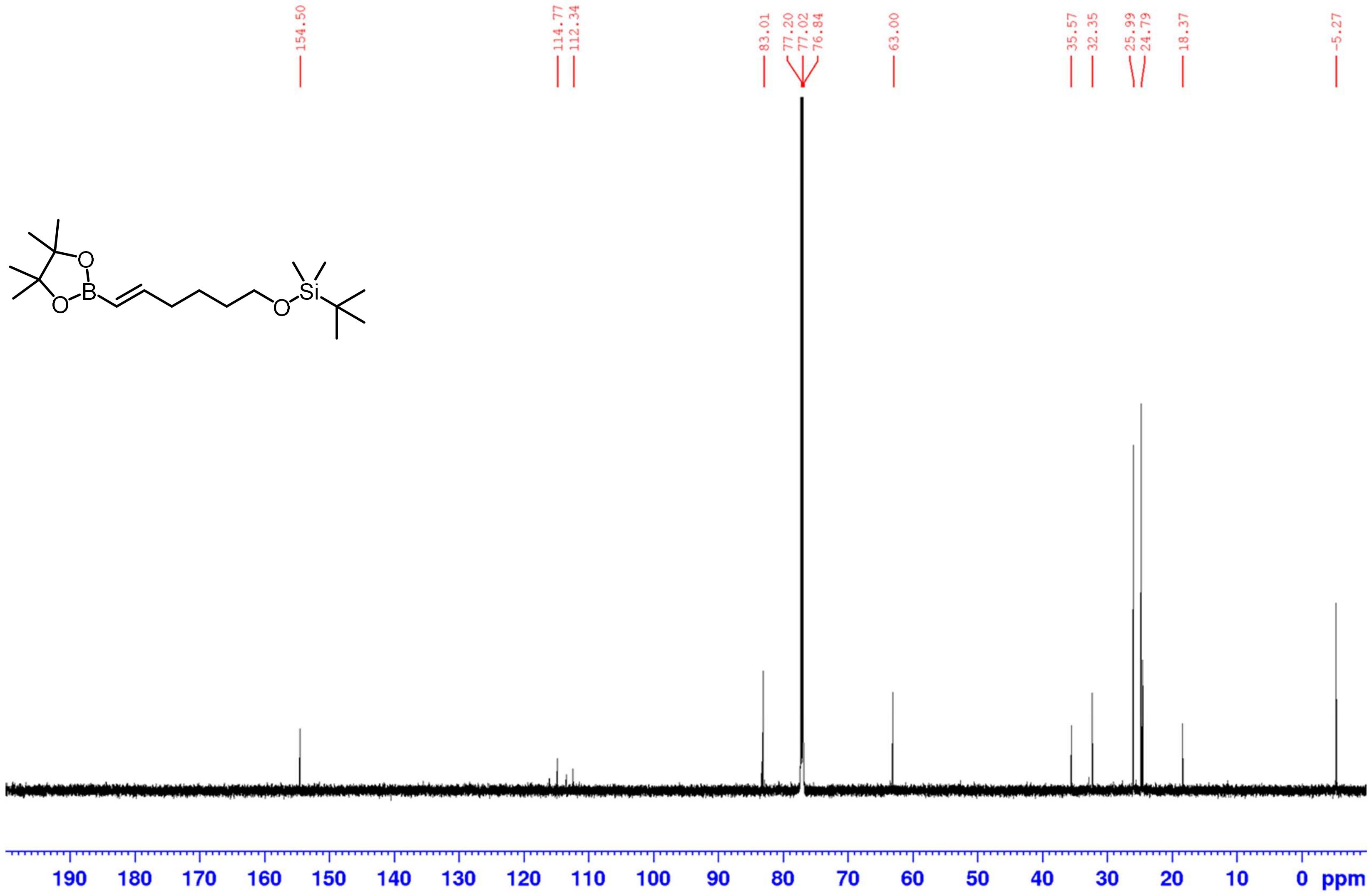

^{1}H NMR (700 MHz, $CDCl_3$) for ***(E)-6-phenylhex-5-en-1-ol (1a)***

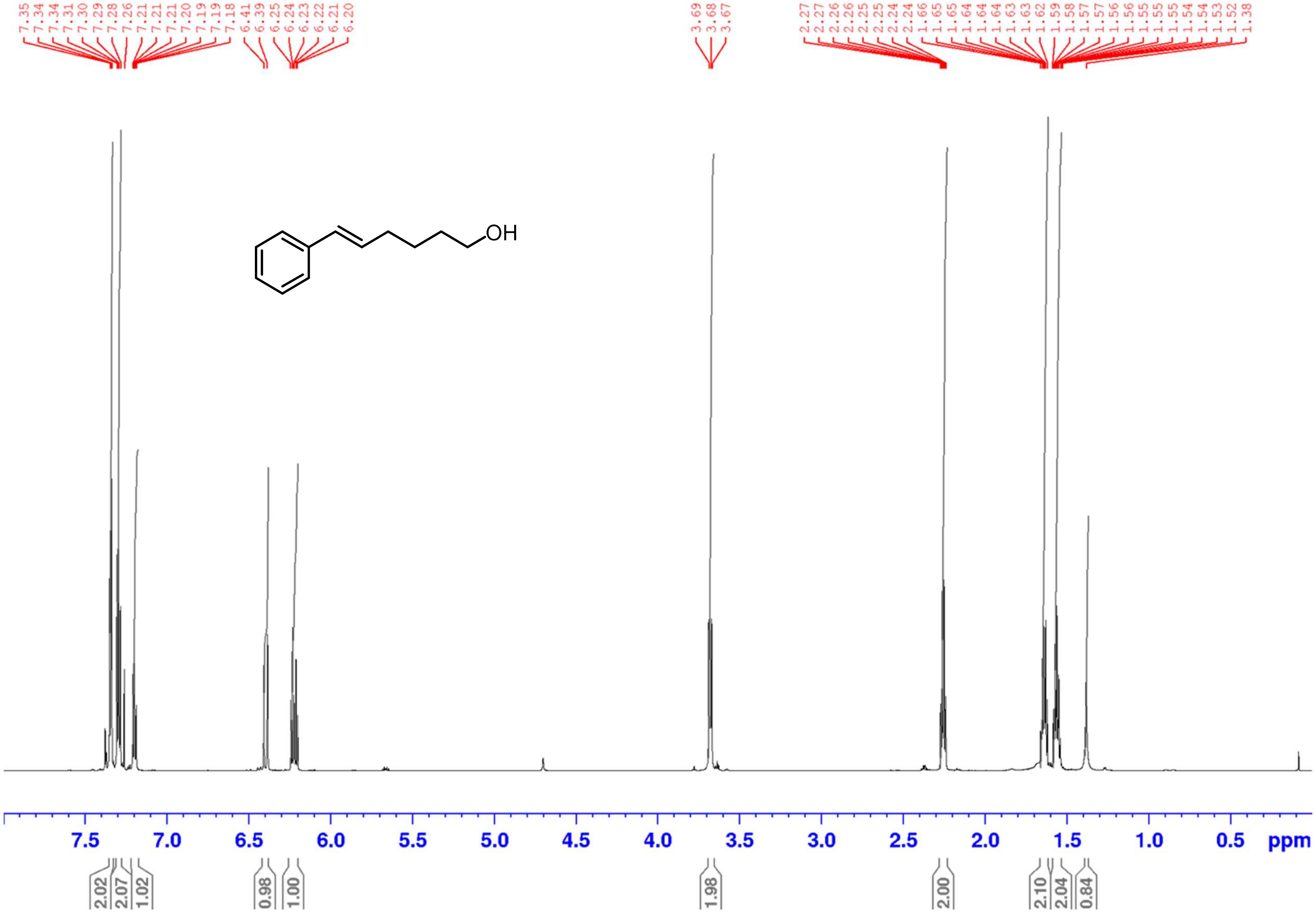

^{13}C NMR (176 MHz, $CDCl_3$) for ***(E)-6-phenylhex-5-en-1-ol (1a)***

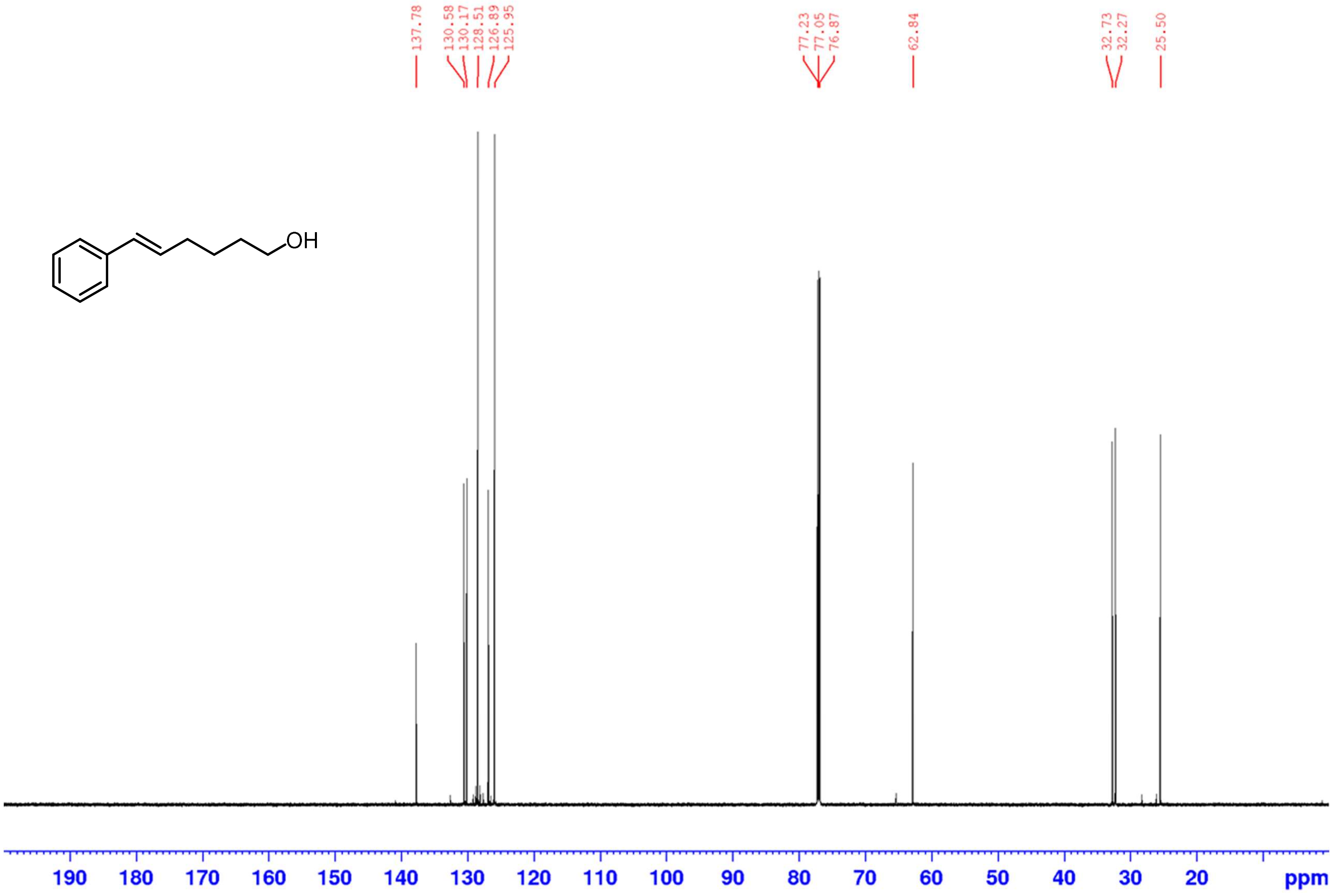

^{1}H NMR (400 MHz, $CDCl_3$) for ***(E)-(6-methoxyhex-1-en-1-yl)benzene***

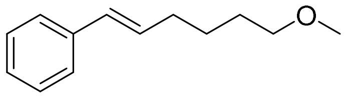

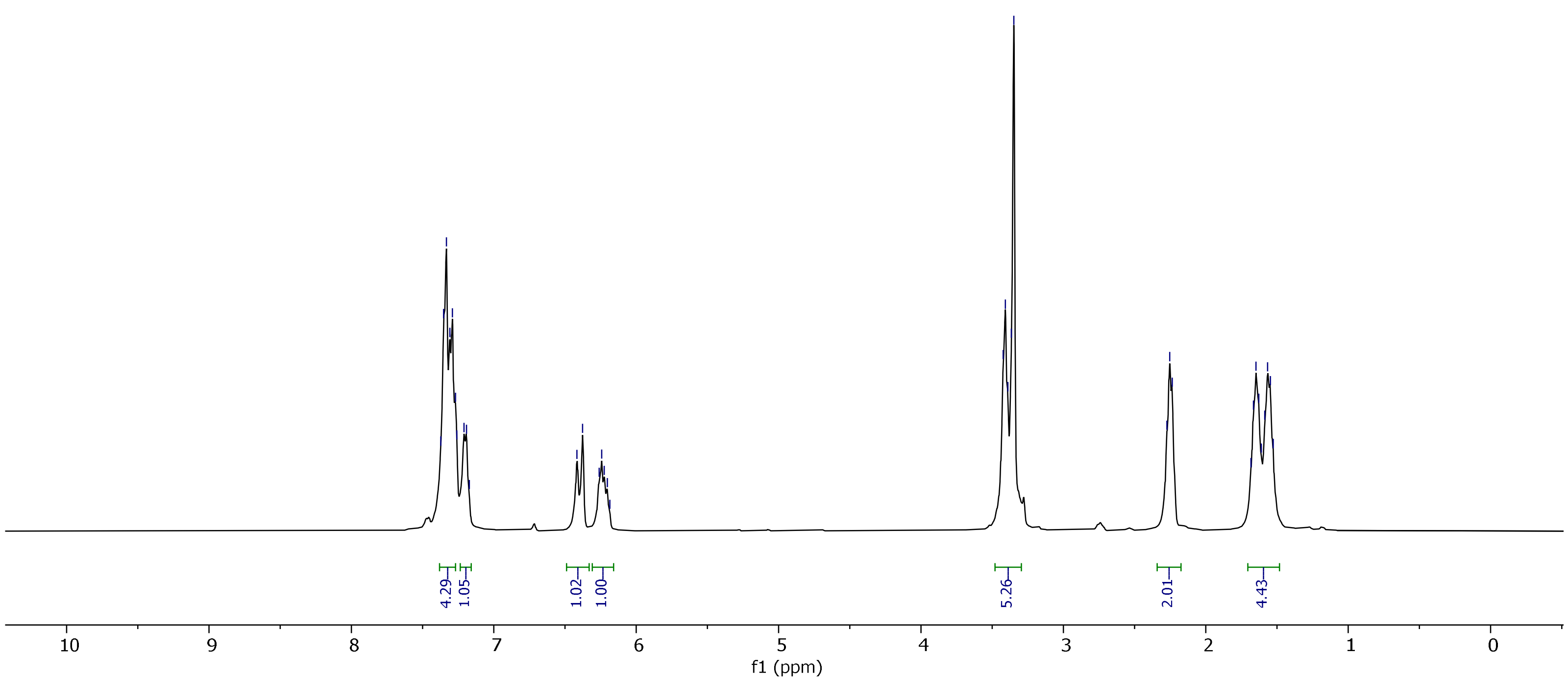

^{13}C NMR (176 MHz, $CDCl_3$) for ***(E)-(6-methoxyhex-1-en-1-yl)benzene***

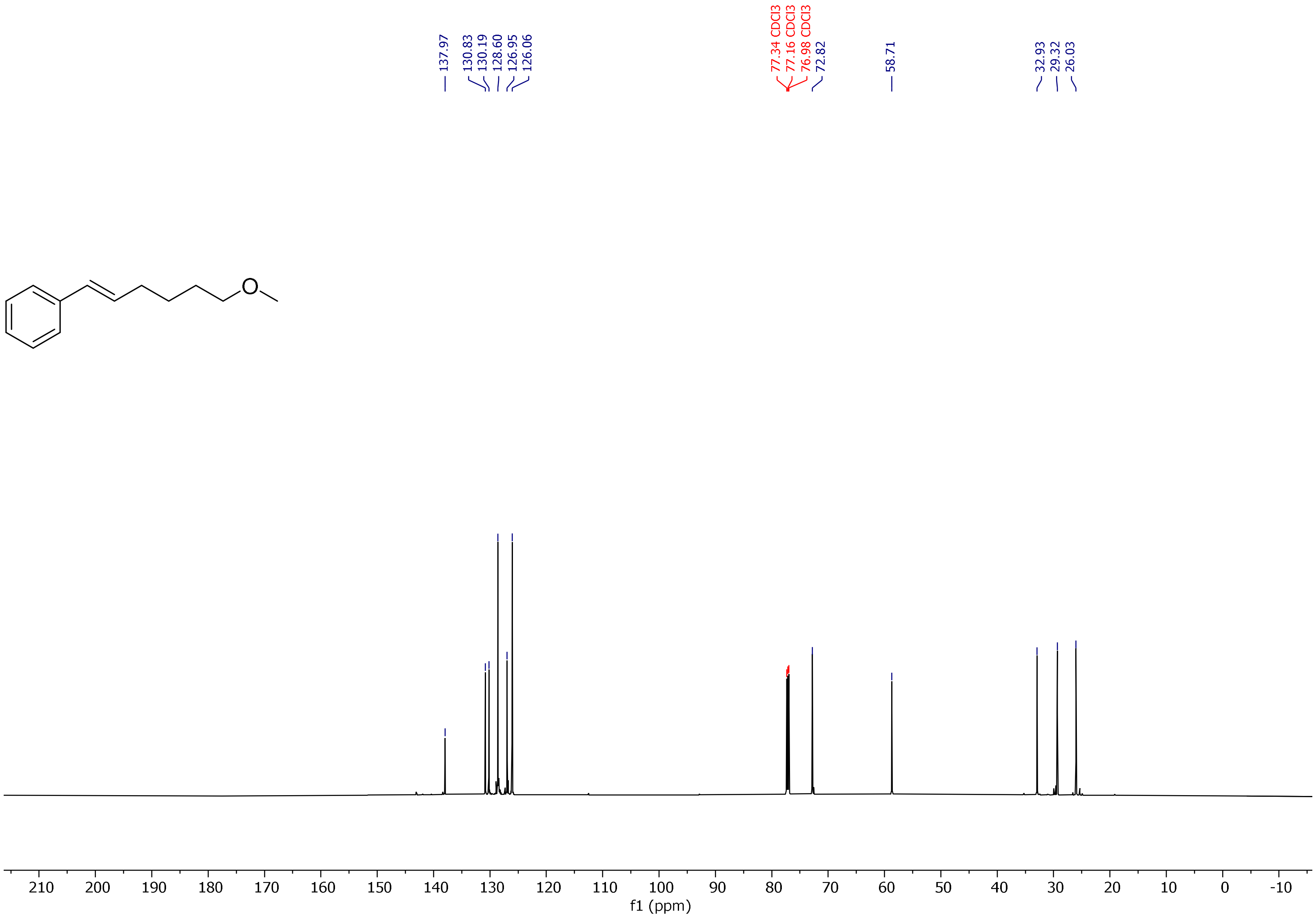

^{1}H NMR (700 MHz, $CDCl_3$) for ***(E)-6-([1,1'-biphenyl]-4-yl)hex-5-en-1-ol (1c)***

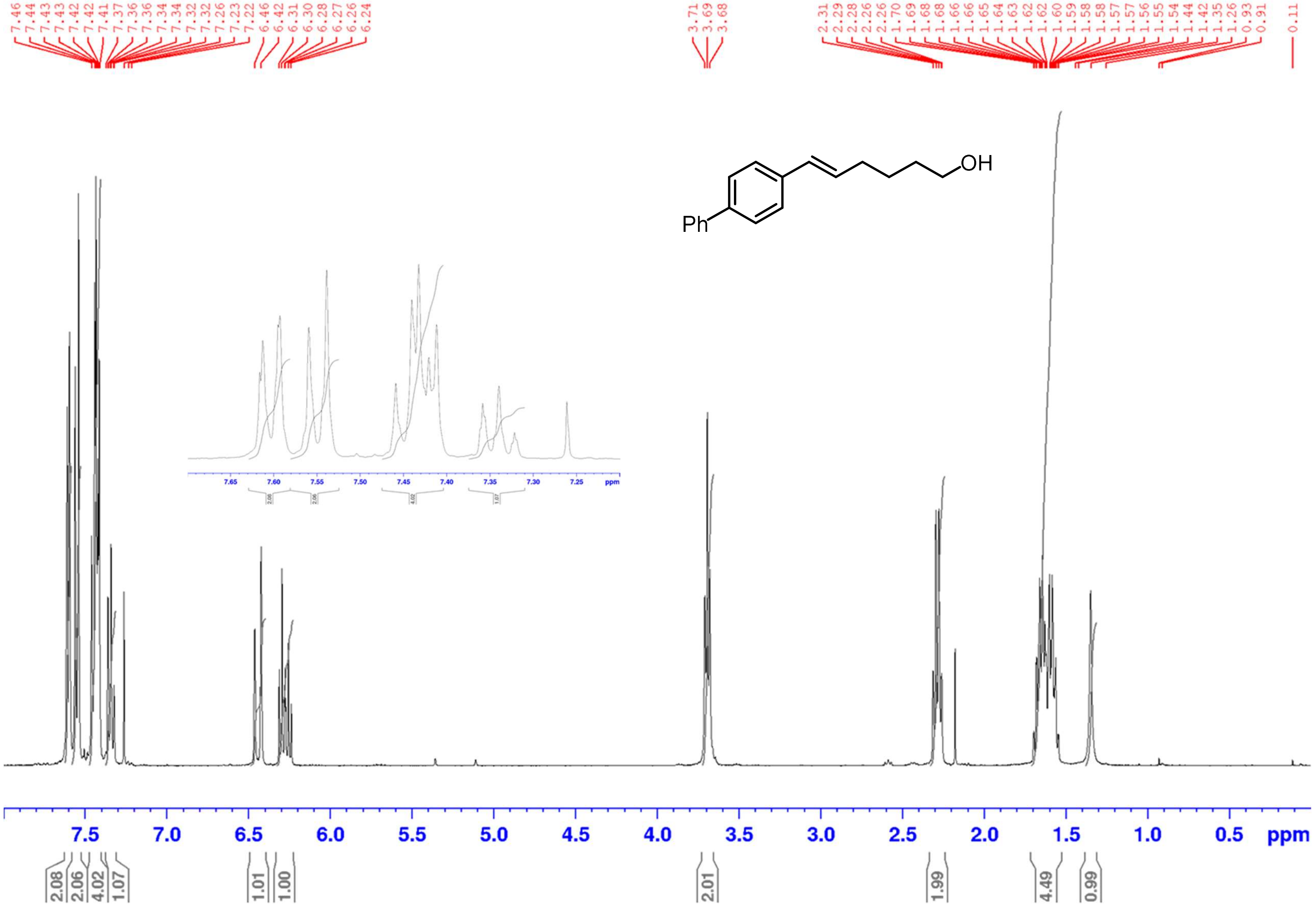

^{13}C NMR (176 MHz, $CDCl_3$) for ***(E)-6-([1,1'-biphenyl]-4-yl)hex-5-en-1-ol (1c)***

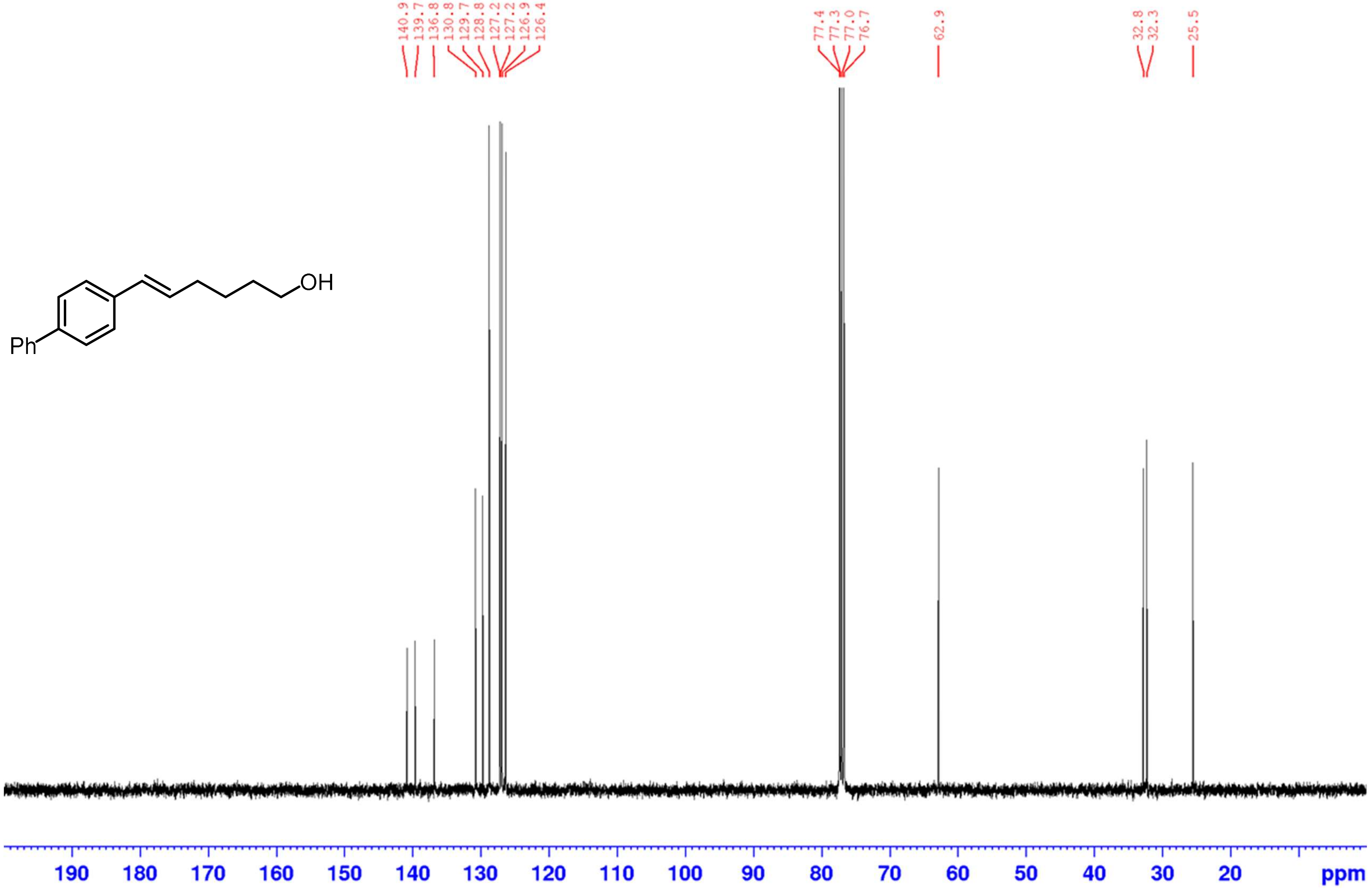

^{1}H NMR (700 MHz, $CDCl_3$) for ***(E)-6-(4-fluorophenyl)hex-5-en-1-ol (1d)***

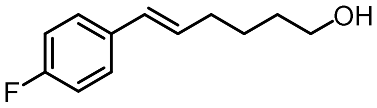

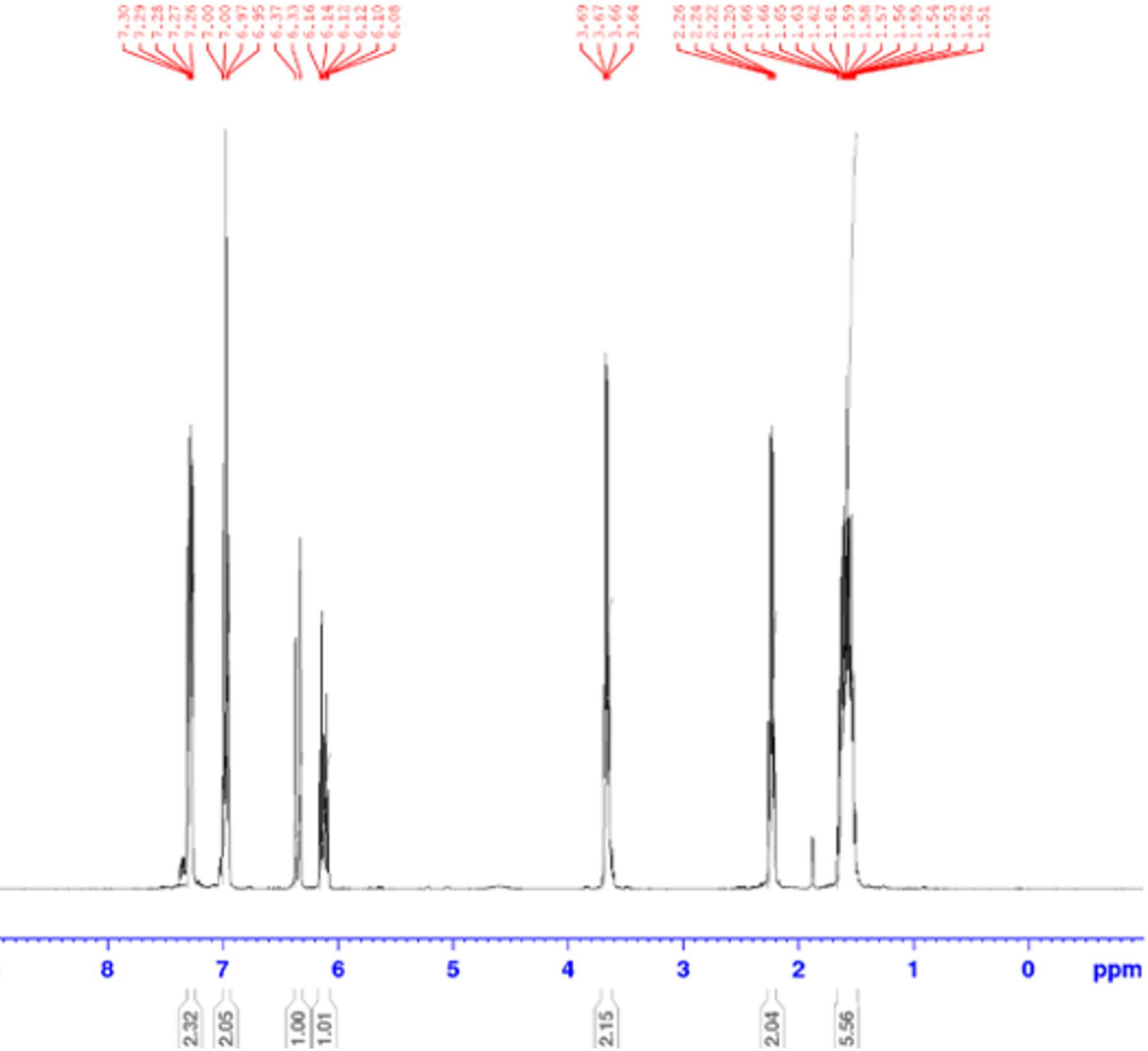

^{13}C NMR (176 MHz, $CDCl_3$) for ***(E)-6-(4-fluorophenyl)hex-5-en-1-ol (1d)***

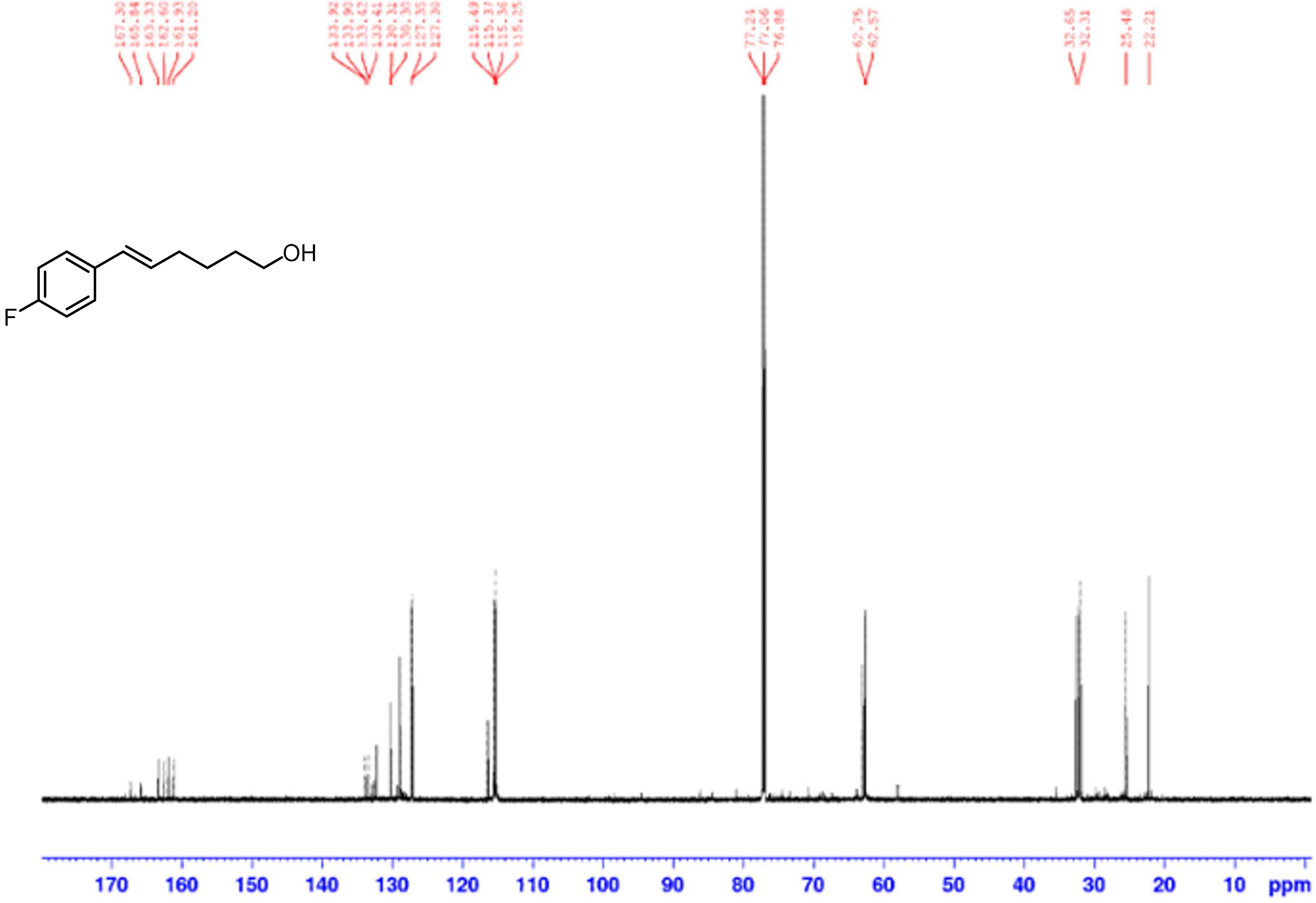

^{19}F NMR (376 MHz, $CDCl_3$) for ***(E)-6-(4-fluorophenyl)hex-5-en-1-ol (1d)***

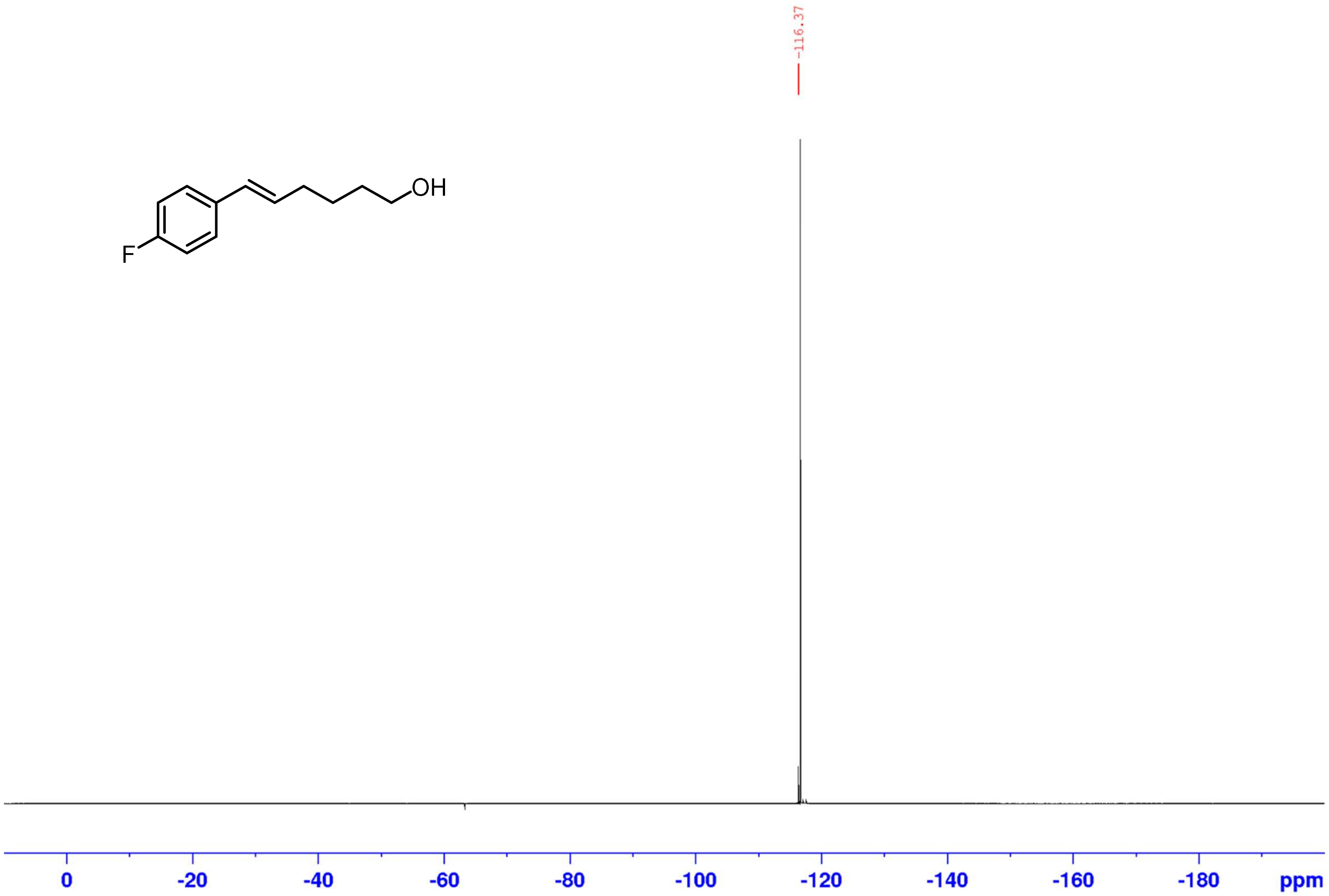

^{1}H NMR (700 MHz, $CDCl_3$) for ***(E)-6-(4-(tert-butyl)phenyl)hex-5-en-1-ol (1e)***

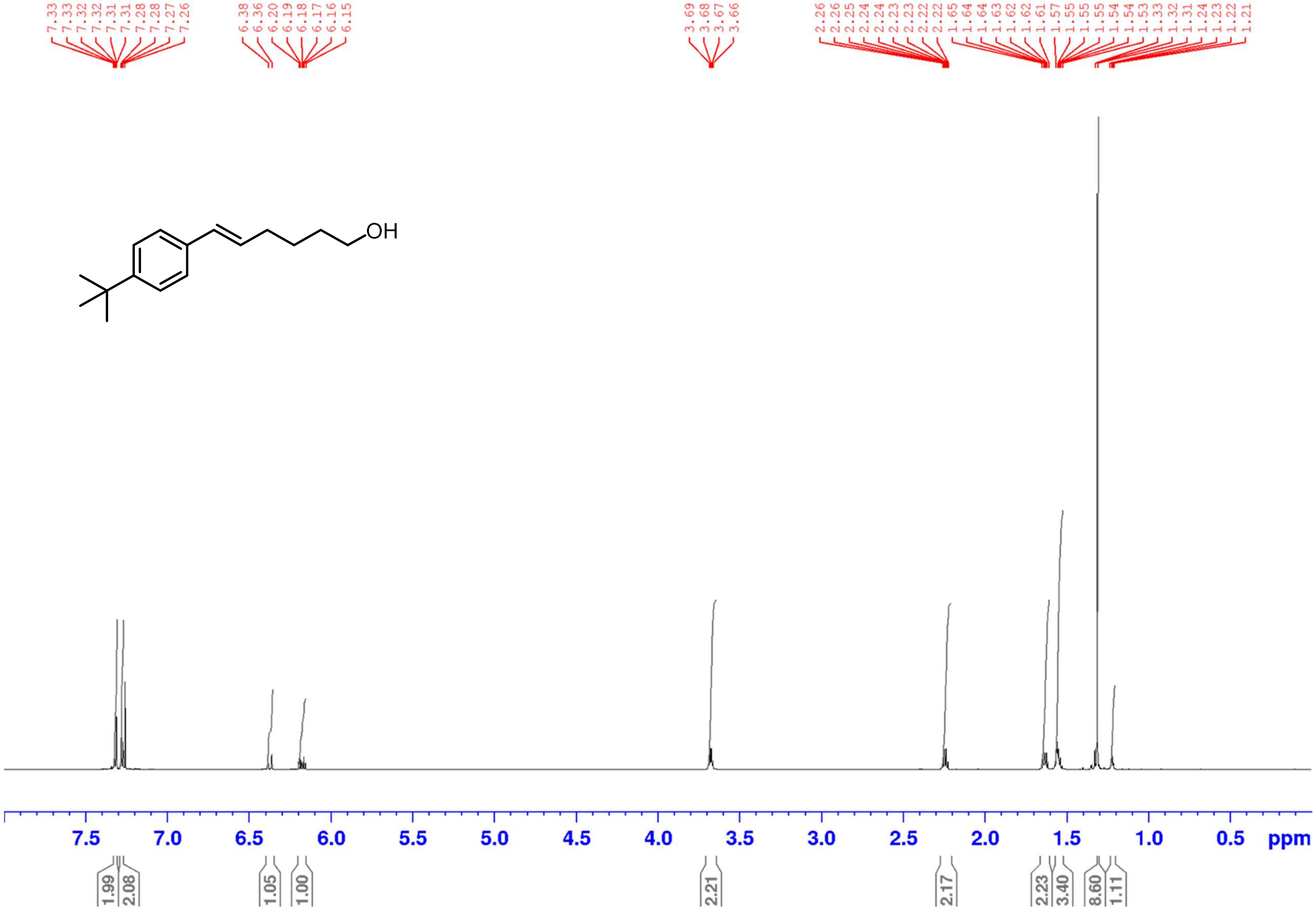

^{13}C NMR (176 MHz, $CDCl_3$) for ***(E)-6-(4-(tert-butyl)phenyl)hex-5-en-1-ol (1e)***

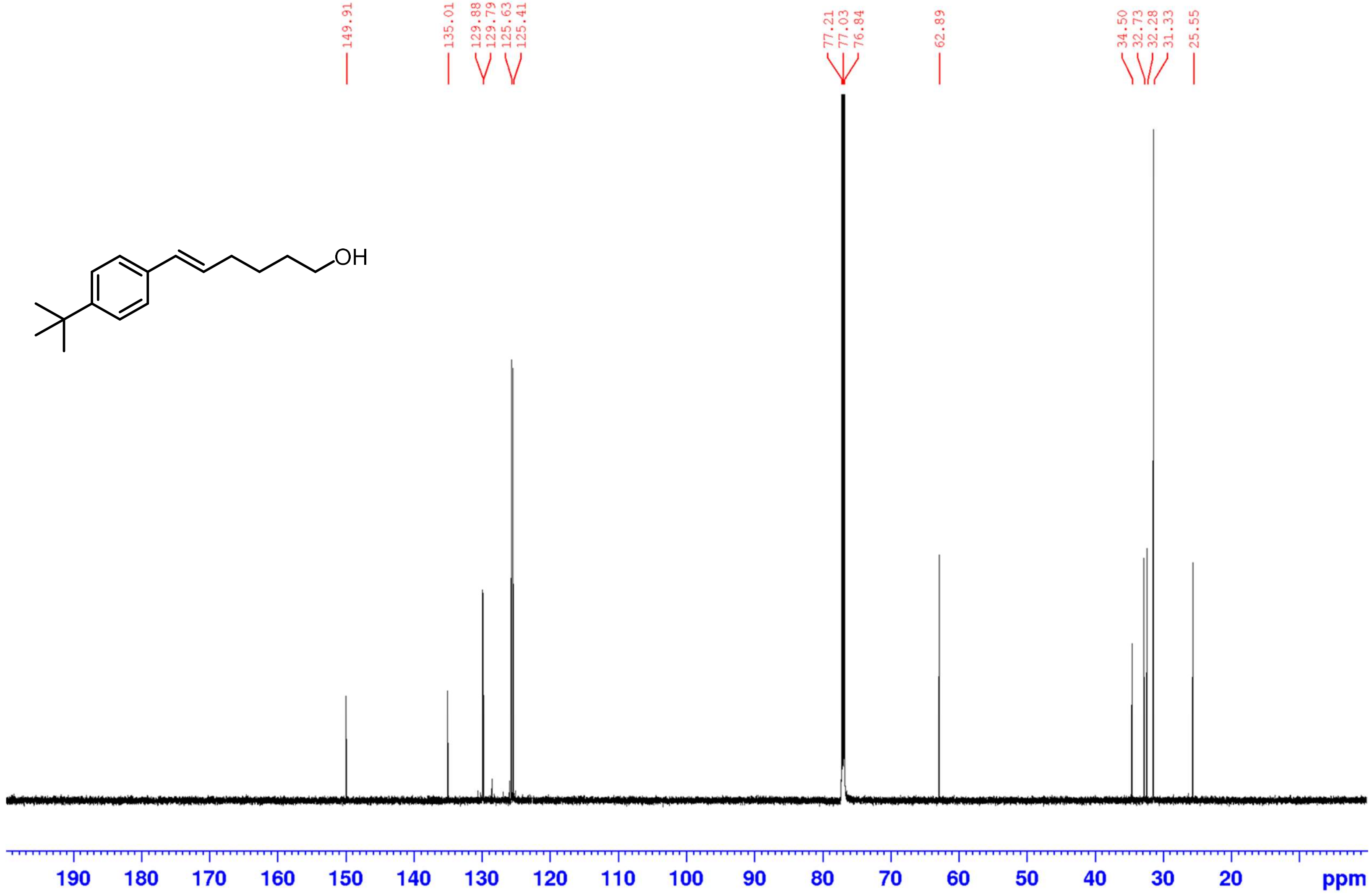

¹H NMR (700 MHz, $CDCl_3$) for ***(E)-3-(6-hydroxyhex-1-en-1-yl)phenyl acetate (1f)***

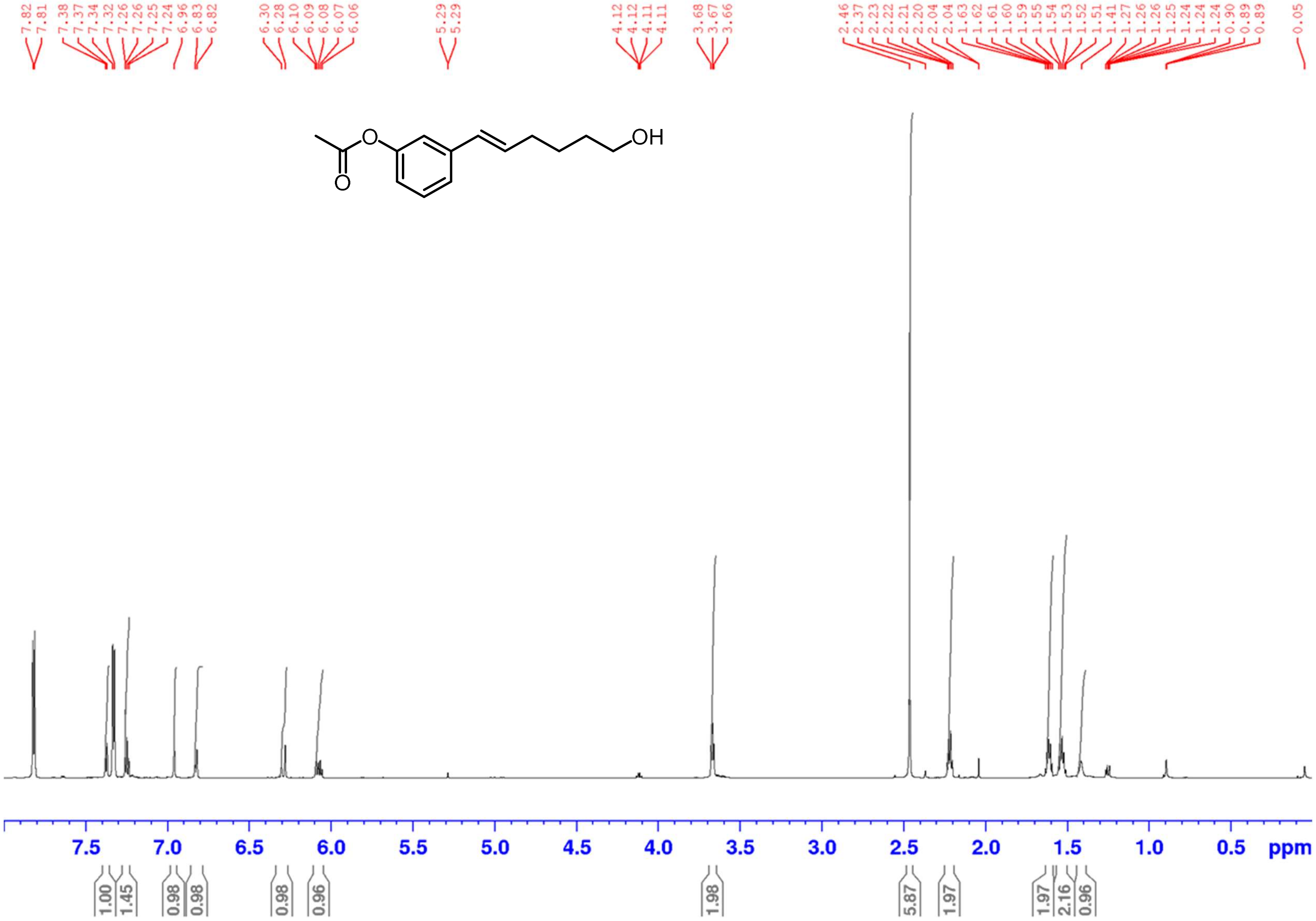

^{13}C **NMR** (176 MHz, $CDCl_3$) for ***(E)-3-(6-hydroxyhex-1-en-1-yl)phenyl acetate (1f)***

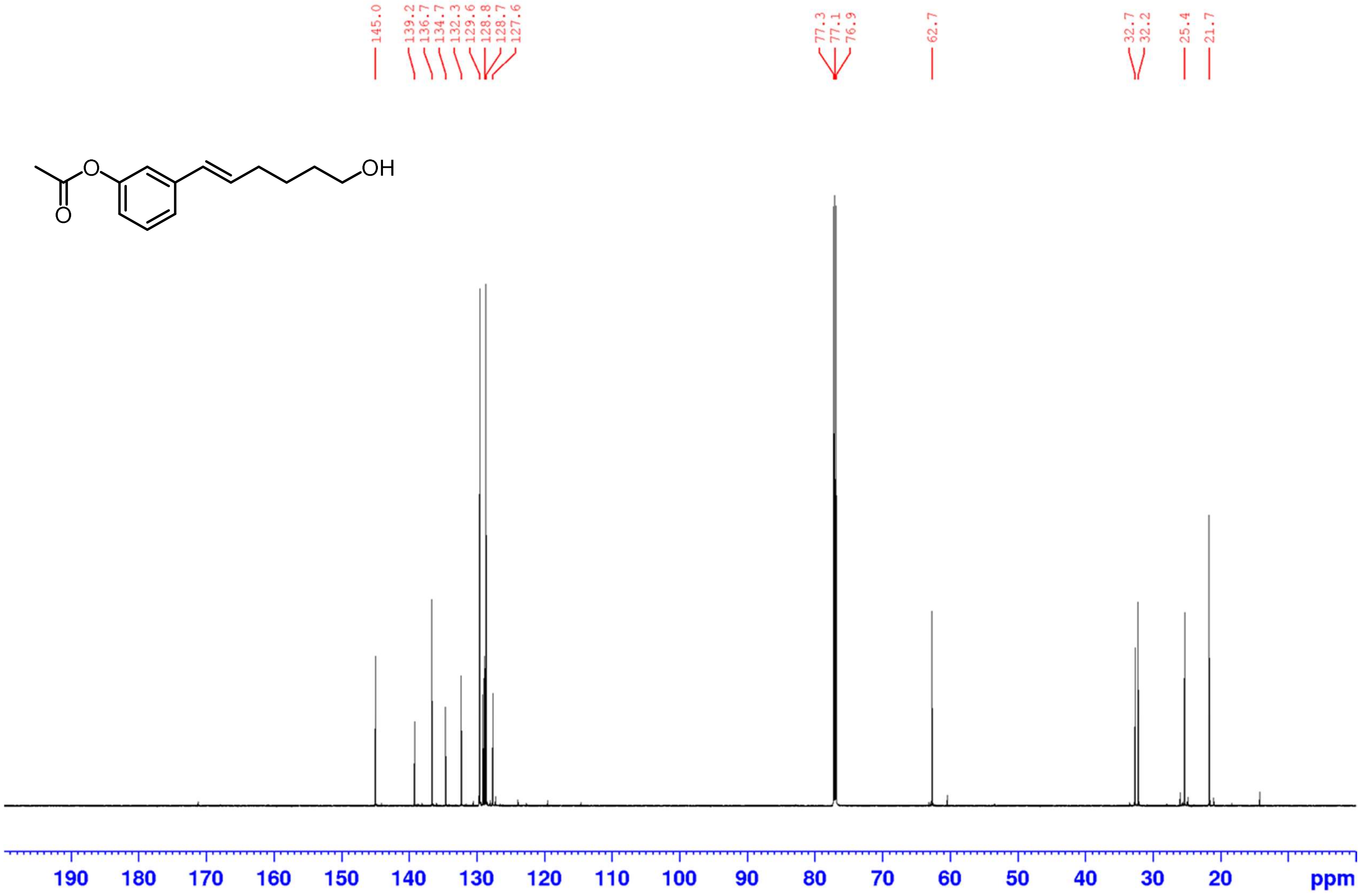

¹H NMR (400 MHz, $CDCl_3$) for ***(E)-1-(3-(6-hydroxyhex-1-en-1-yl)phenyl)ethan-1-one (1b)***

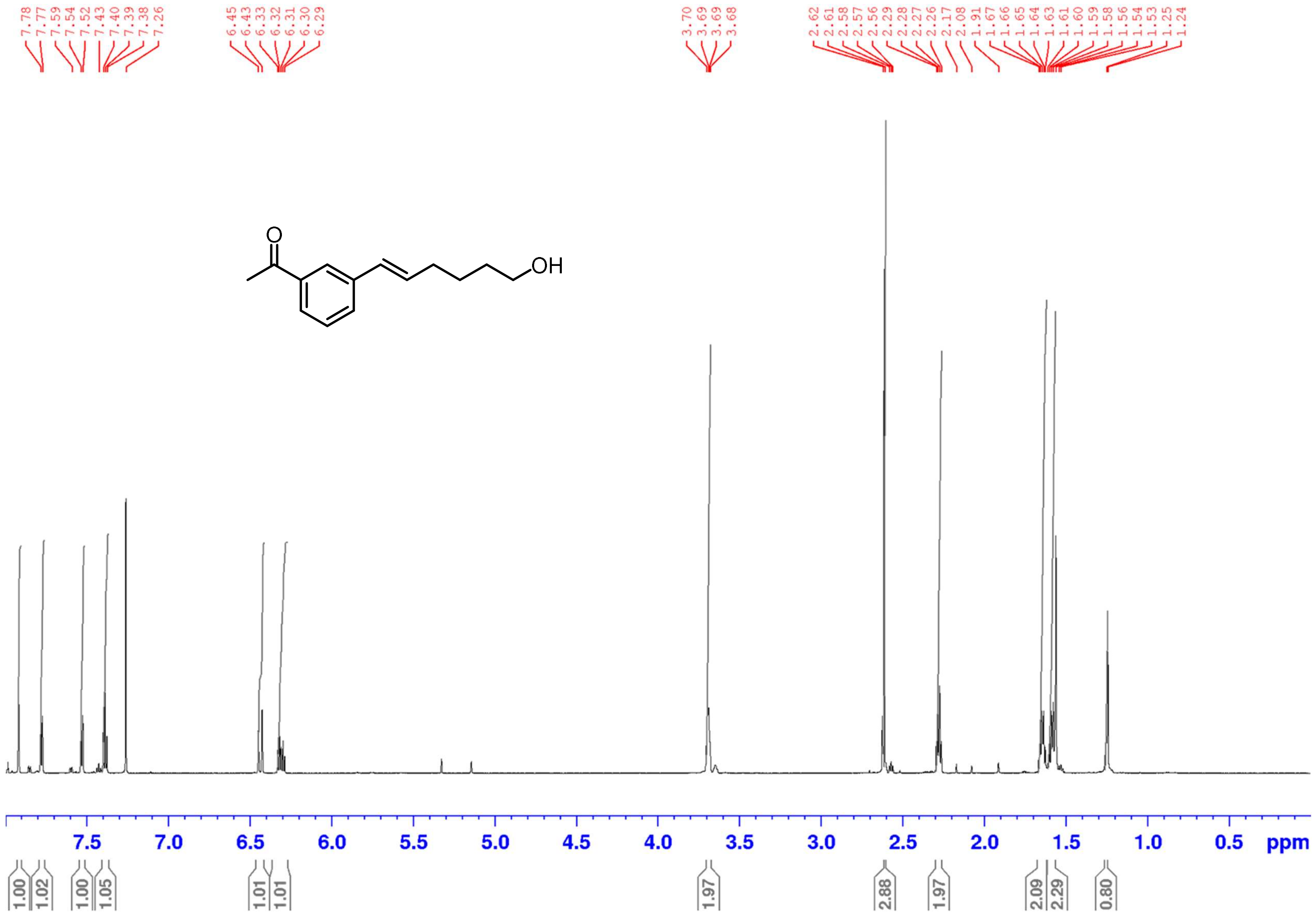

^{13}C NMR (101 MHz, $CDCl_3$) for ***(E)-1-(3-(6-hydroxyhex-1-en-1-yl)phenyl)ethan-1-one (1b)***

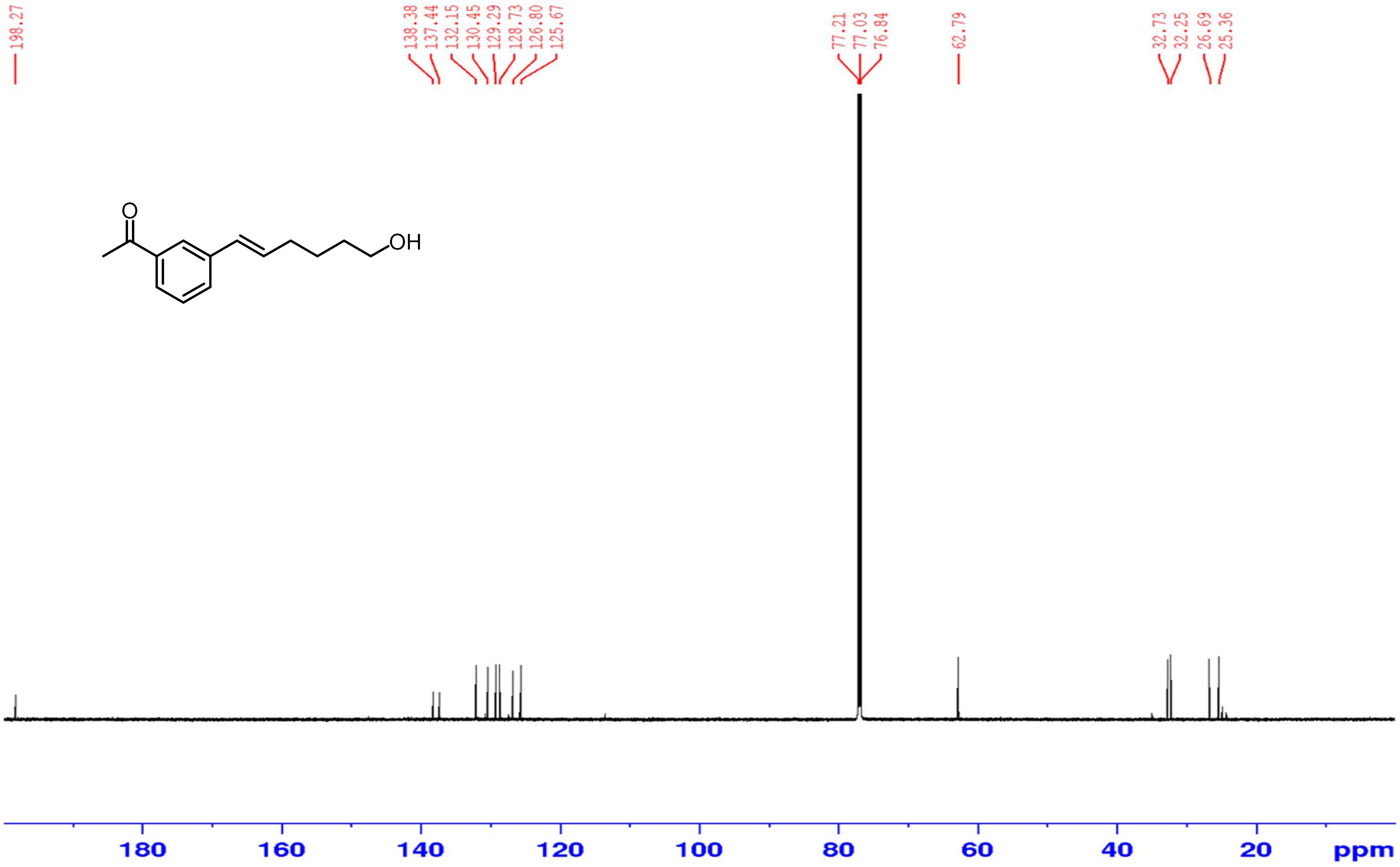

¹H NMR (700 MHz, $CDCl_3$) for ***(E)-6-(3-(tert-butyl)phenyl)hex-5-en-1-ol (1g)***

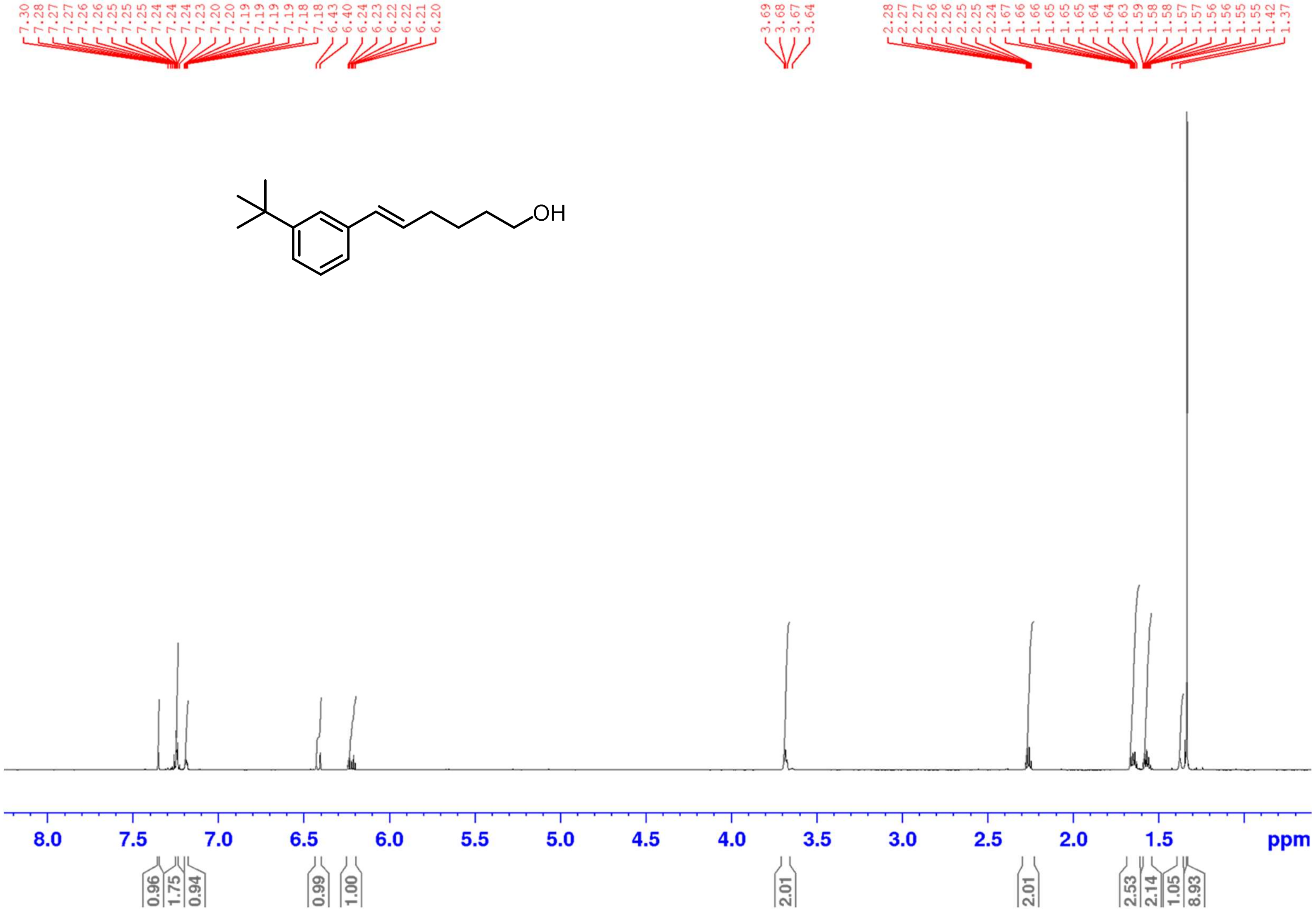

^{13}C NMR (176 MHz, $CDCl_3$) for ***(E)-6-(3-(tert-butyl)phenyl)hex-5-en-1-ol (1g)***

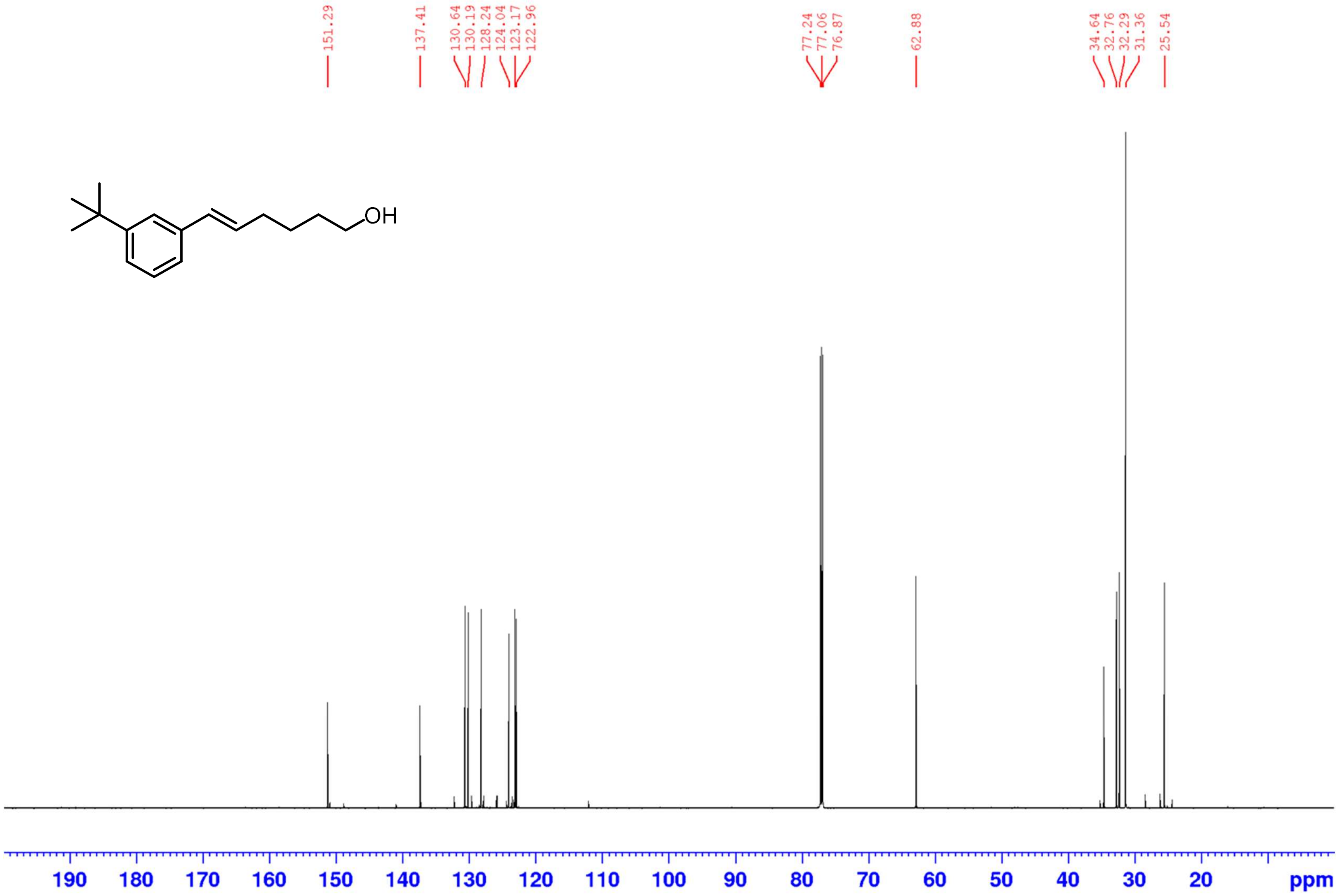

¹H NMR (700 MHz, $CDCl_3$) for ***(E)-6-(3-methoxyphenyl)hex-5-en-1-ol (1h)***

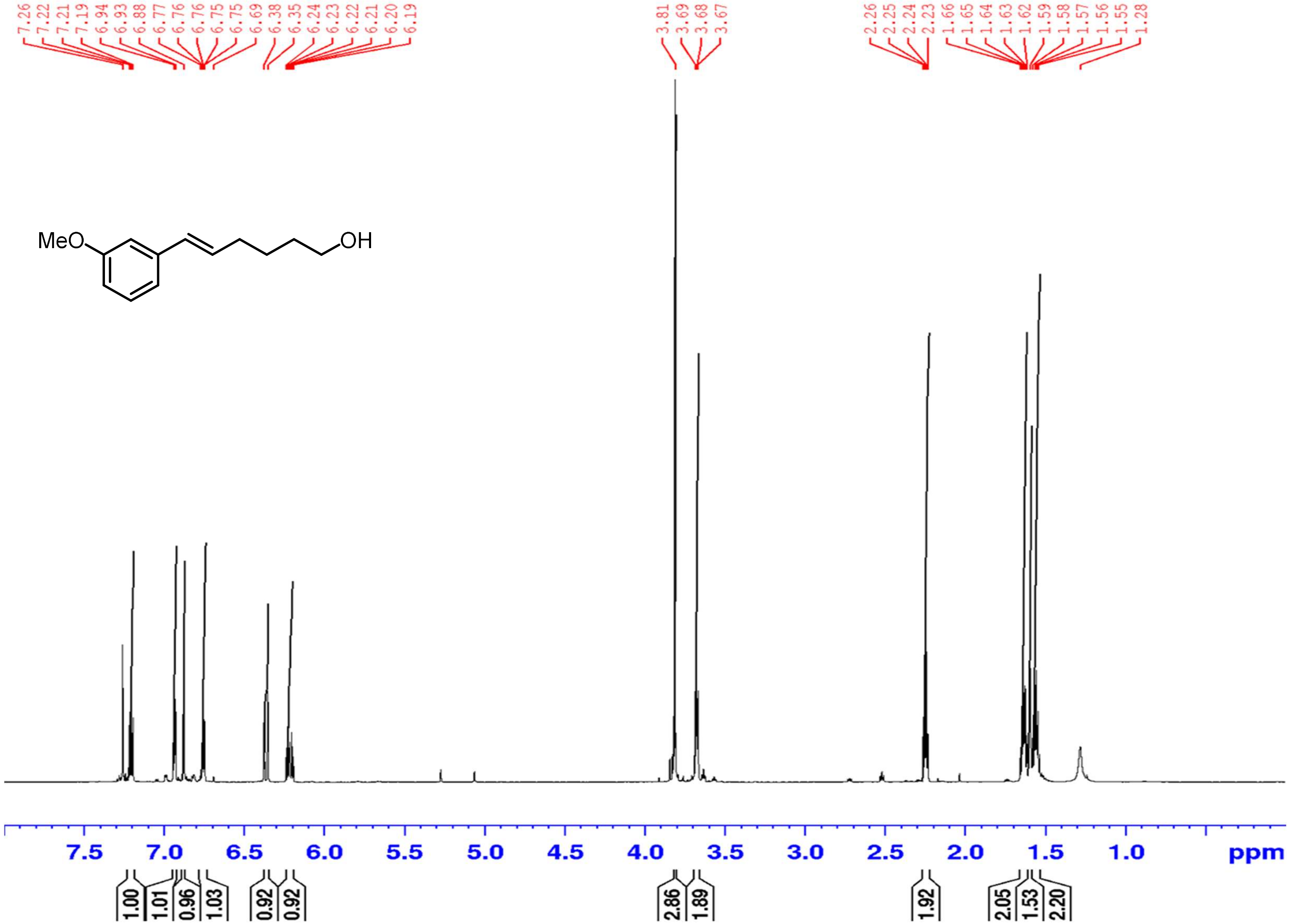

^{13}C NMR (176 MHz, $CDCl_3$) for ***(E)-6-(3-methoxyphenyl)hex-5-en-1-ol (1h)***

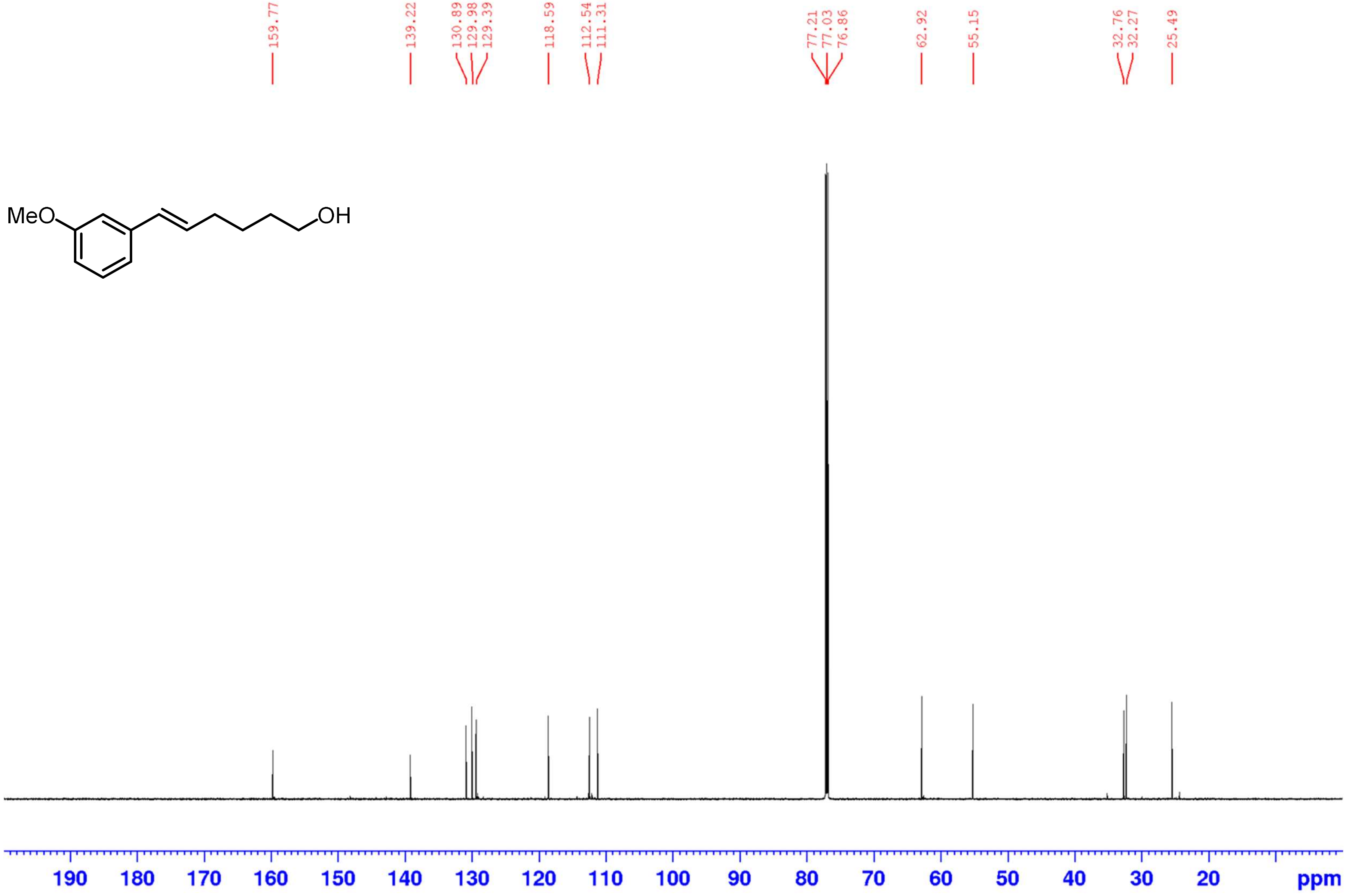

^{1}H NMR (700 MHz, $CDCl_3$) for ***Methyl-(E)-3-(6-hydroxyhex-1-en-1-yl)benzoate (1i)***

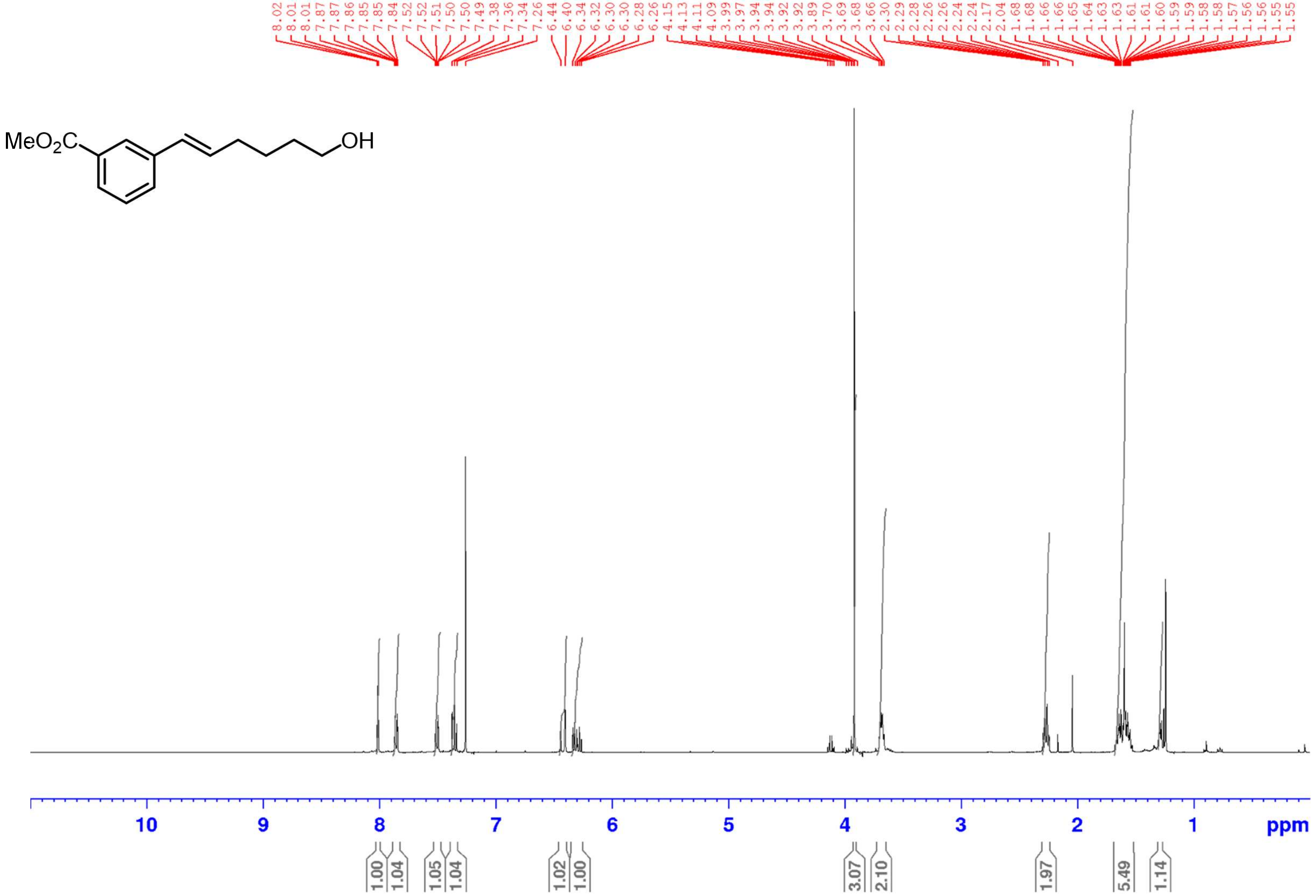

^{13}C NMR (176 MHz, $CDCl_3$) for ***Methyl-(E)-3-(6-hydroxyhex-1-en-1-yl)benzoate (1i)***

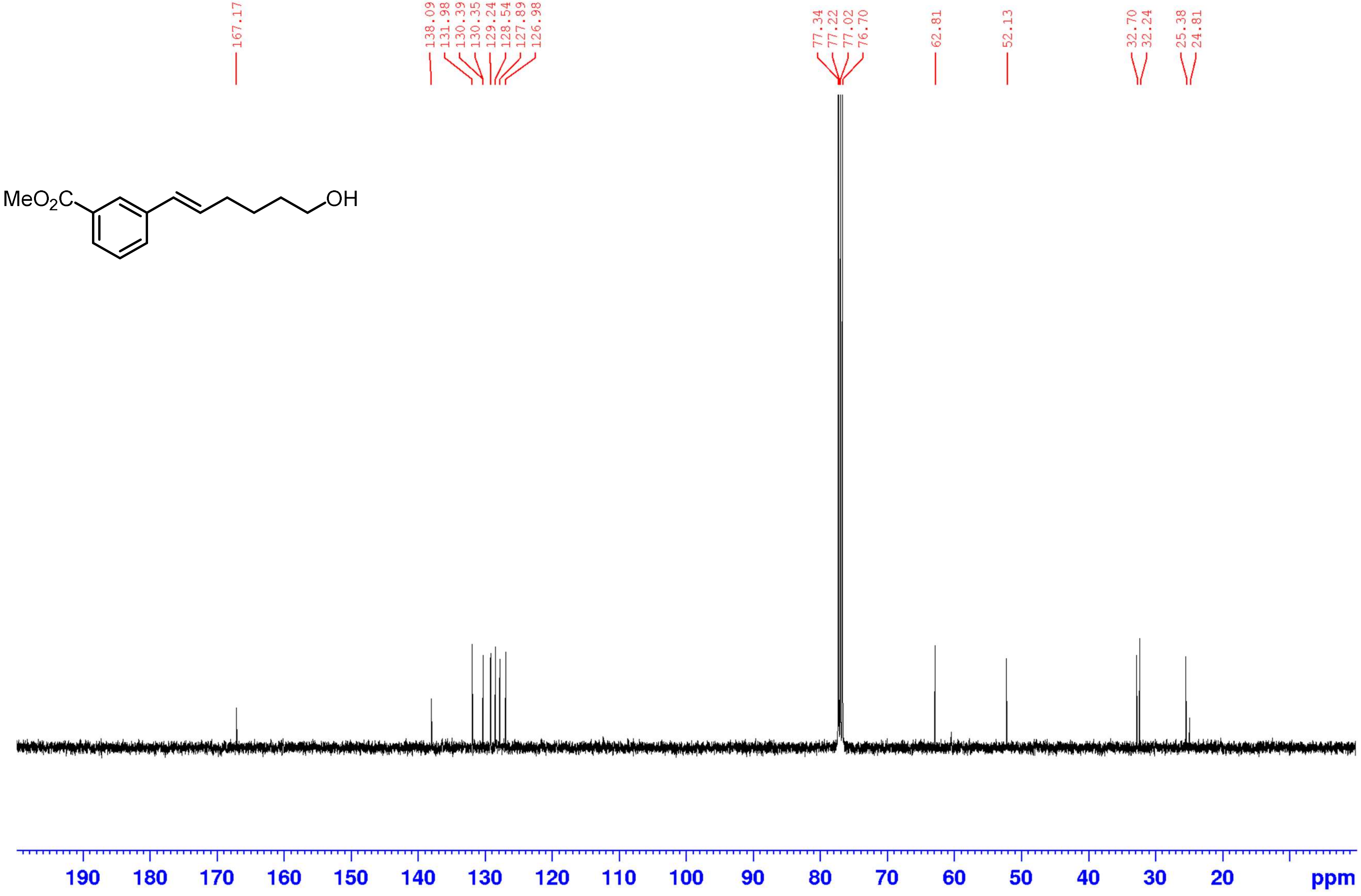

^{1}H NMR (700 MHz, $CDCl_3$) for ***tert-Butyl (E)-(3-(6-hydroxyhex-1-en-1-yl)phenyl)carbamate (1j)***

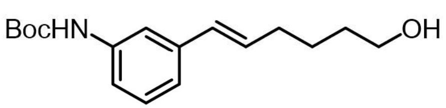

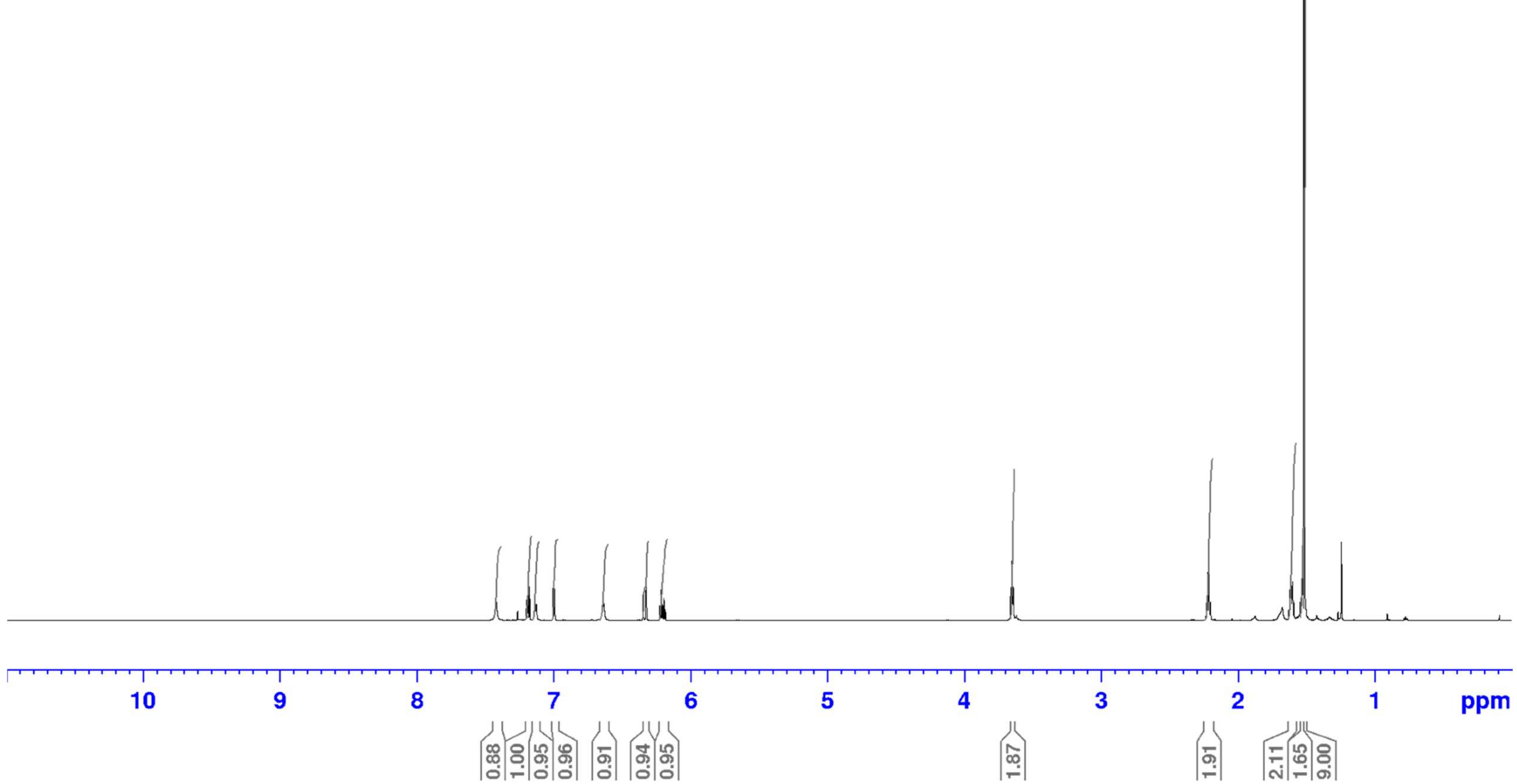

^{13}C NMR (176 MHz, $CDCl_3$) for ***tert*-Butyl (*E*)-(3-(6-hydroxyhex-1-en-1-yl)phenyl)carbamate (1j)**

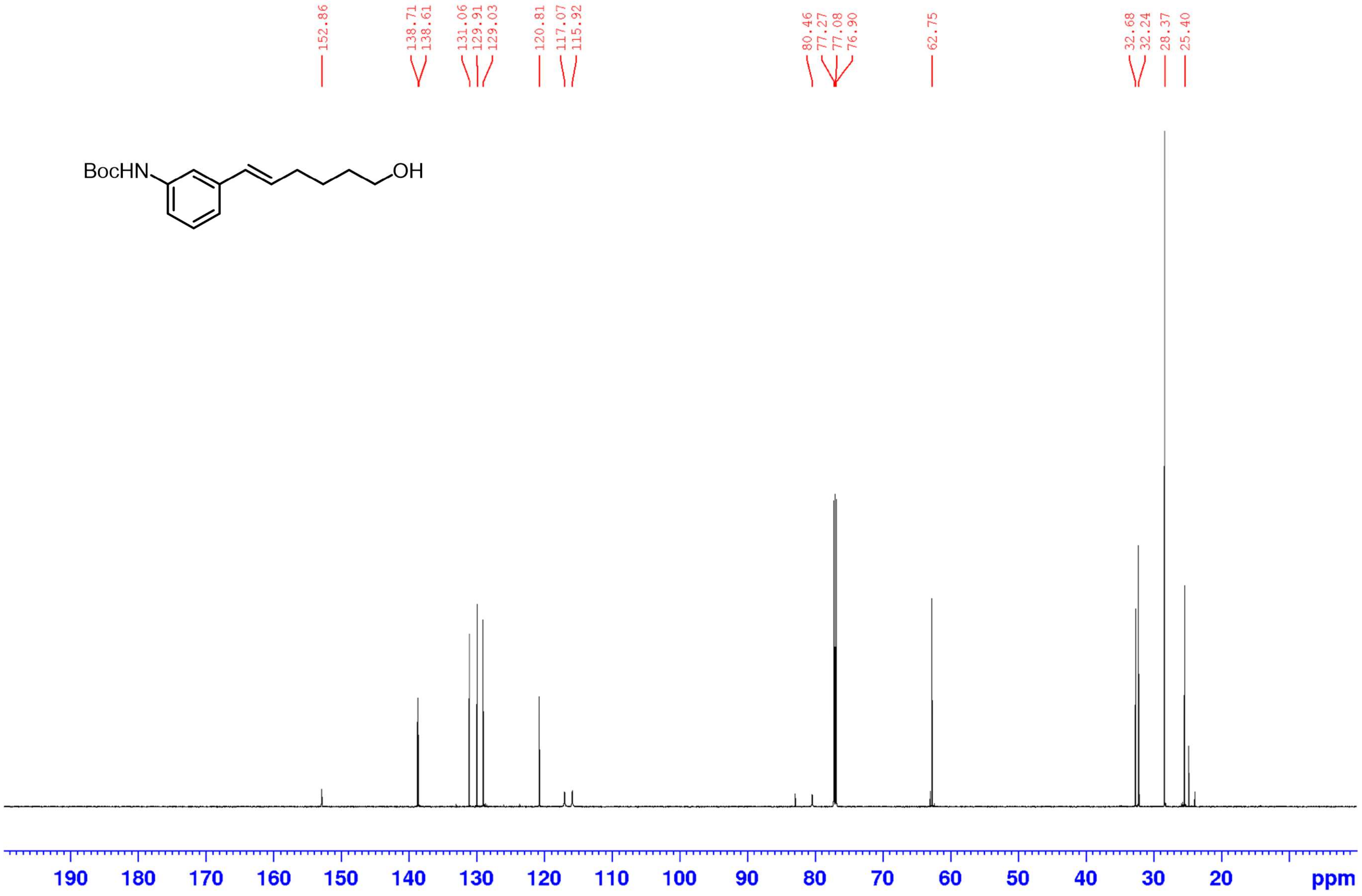

^{1}H NMR (400 MHz, CDCl3) for *(E)-6-(2-methoxyphenyl)hex-5-en-1-ol (1k)*

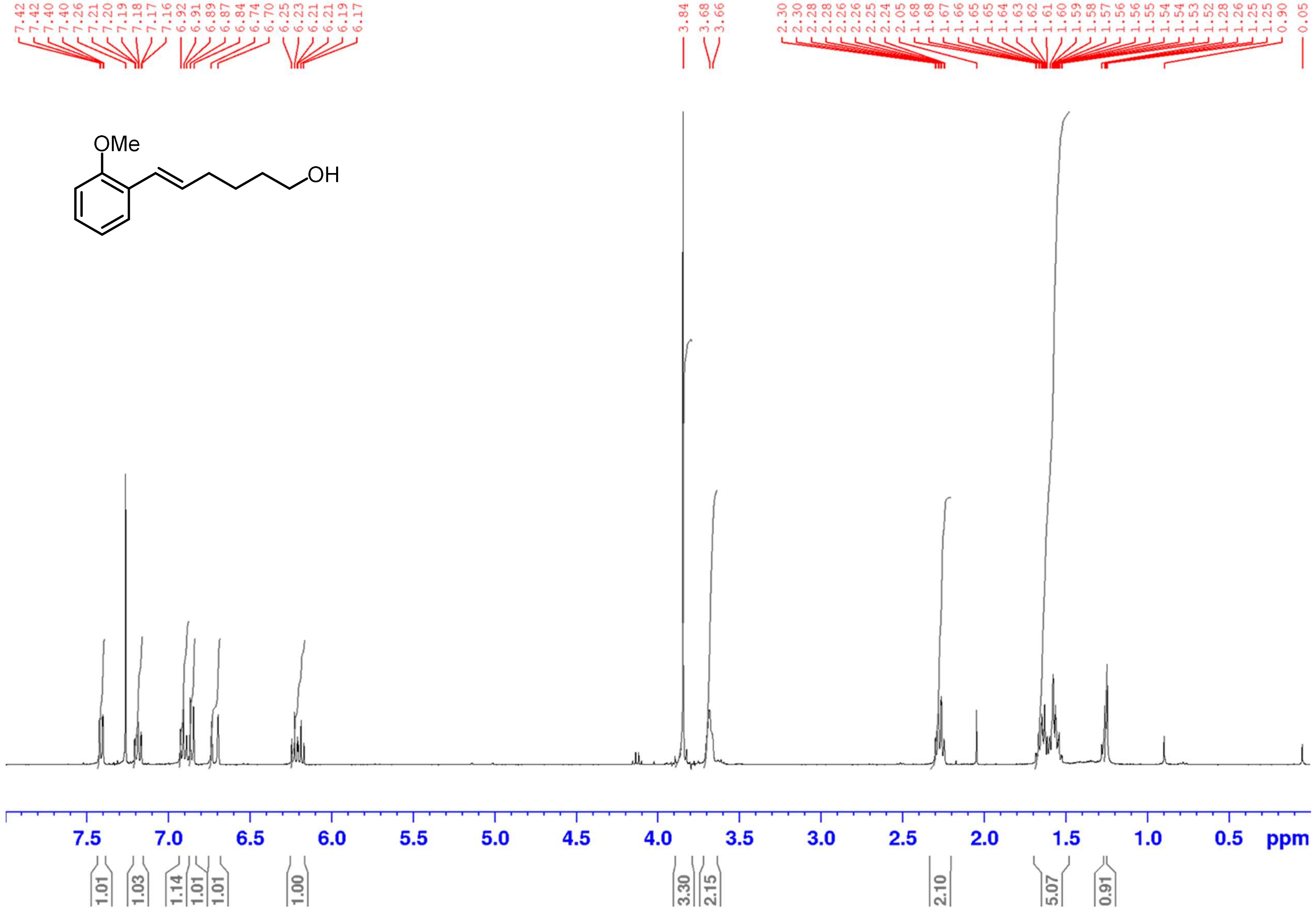

^{13}C NMR (101 MHz, $CDCl_3$) for ***(E)-6-(2-methoxyphenyl)hex-5-en-1-ol (1k)***

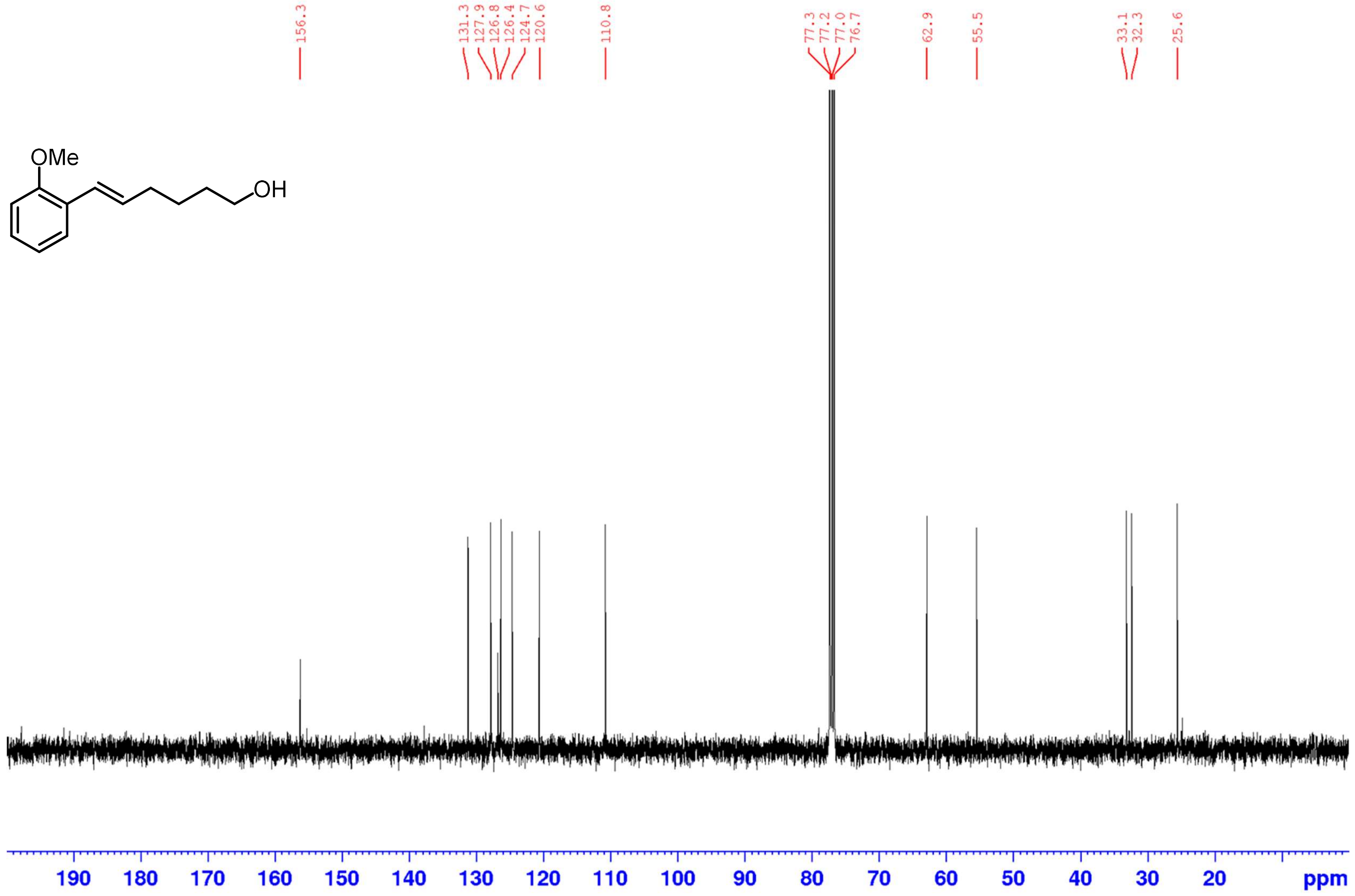

^{1}H NMR (700 MHz, $CDCl_3$) for ***(E)-6-(o-tolyl)hex-5-en-1-ol (1l)***

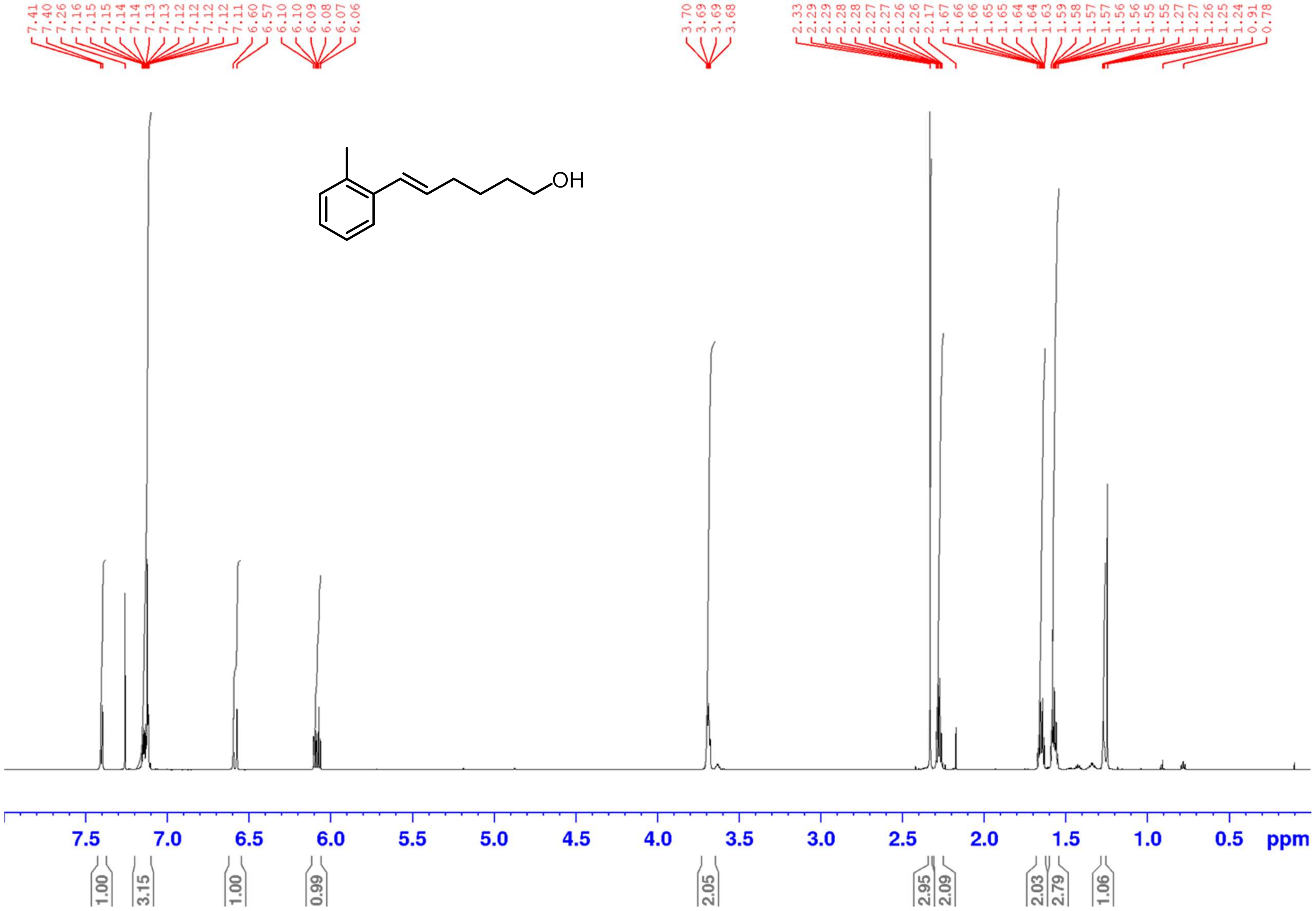

^{13}C NMR (176 MHz, $CDCl_3$) for ***(E)-6-(o-tolyl)hex-5-en-1-ol (1l)***

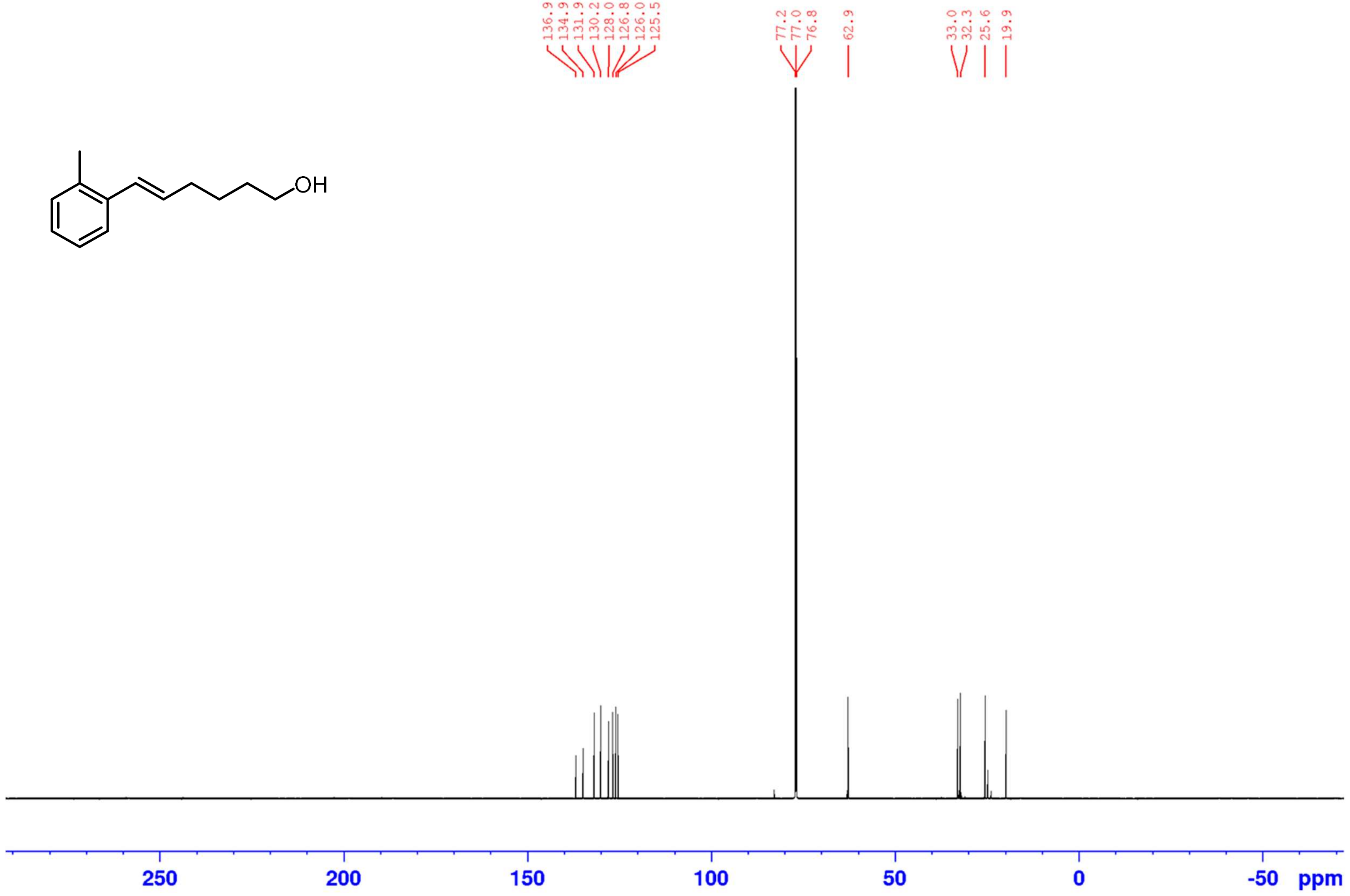

^{1}H NMR (400 MHz, $CDCl_3$) for ***(E)-6-(2-chlorophenyl)hex-5-en-1-ol (1m)***

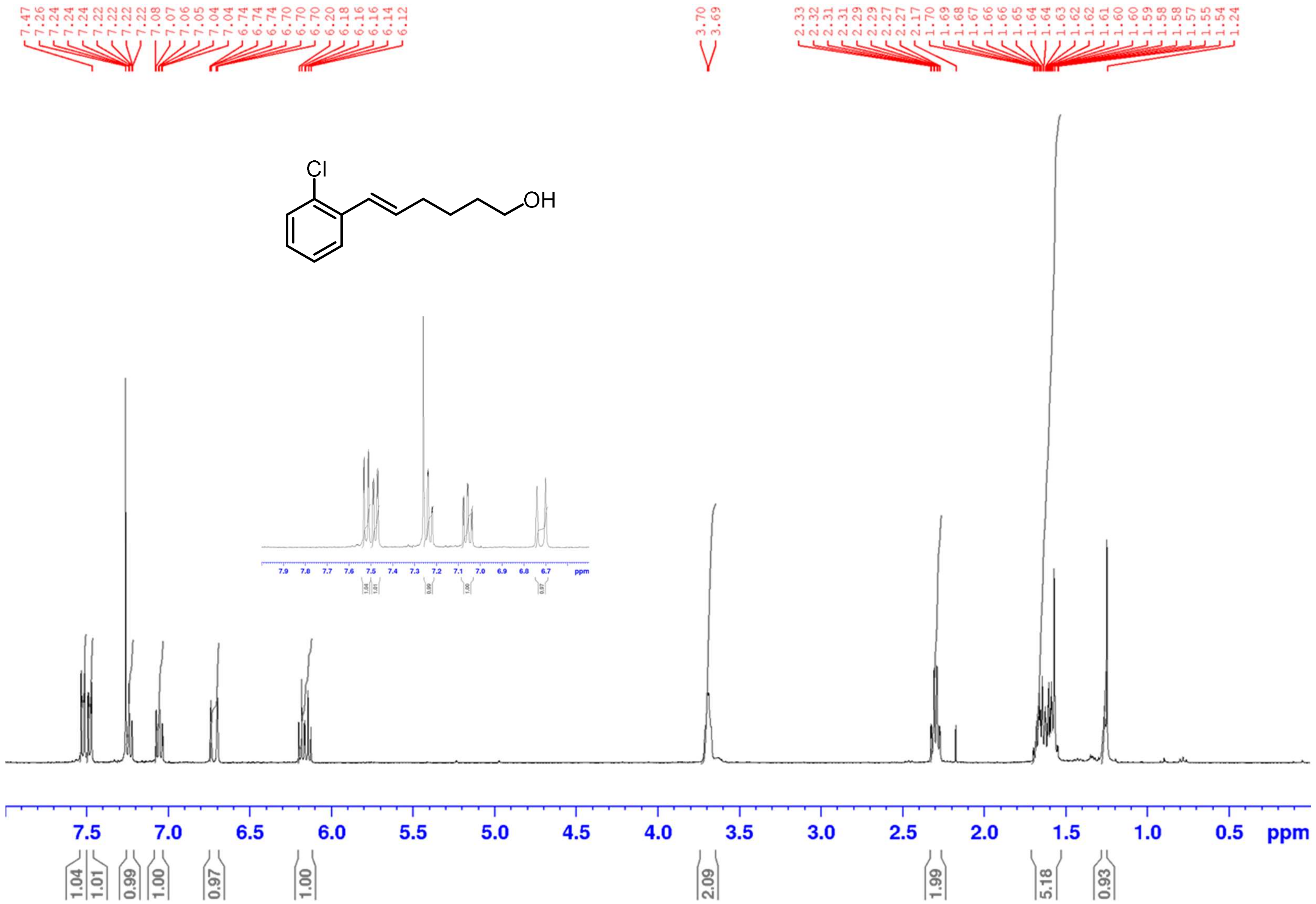

^{13}C NMR (101 MHz, $CDCl_3$) for ***(E)-6-(2-chlorophenyl)hex-5-en-1-ol (1m)***

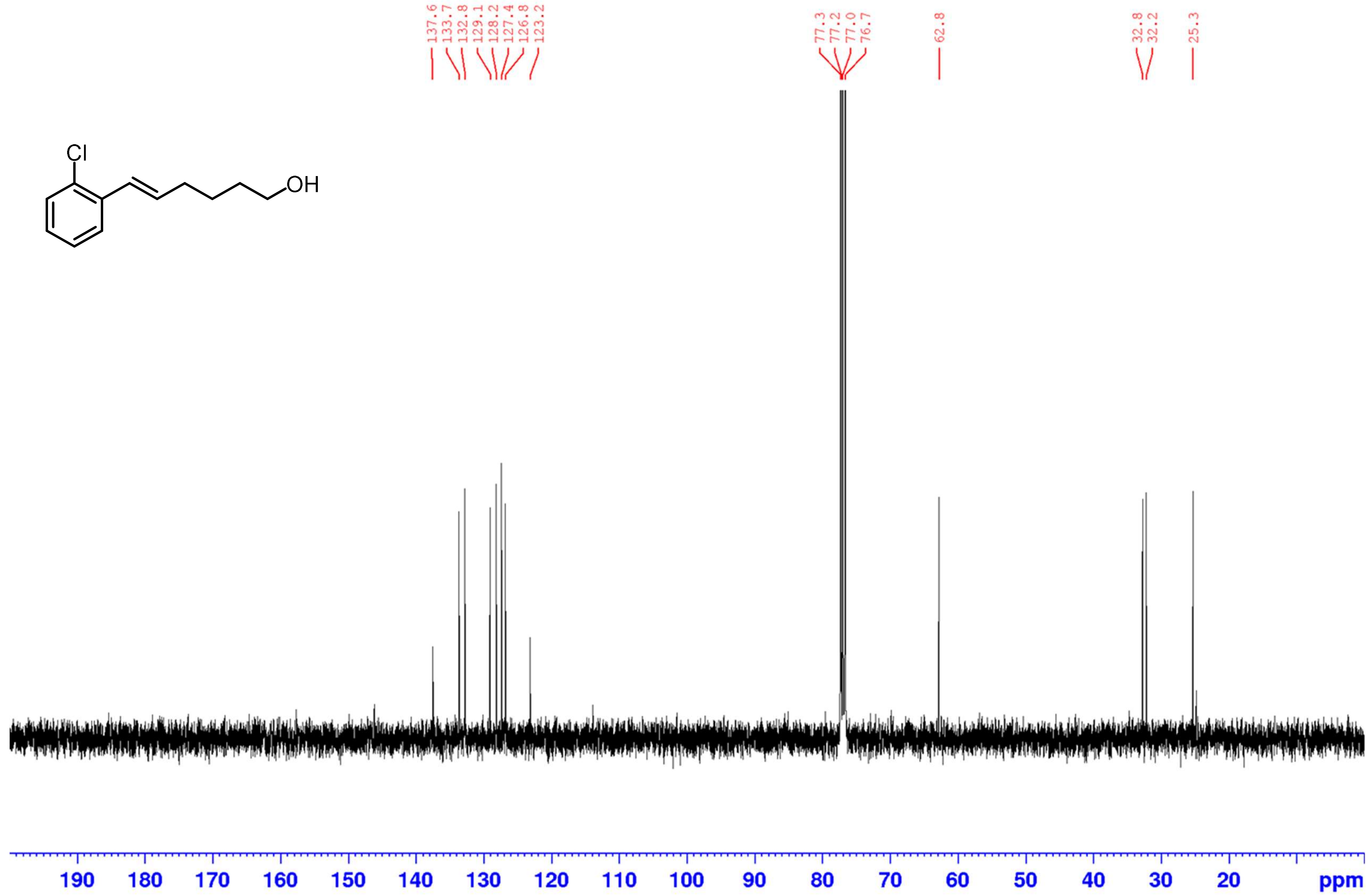

^{1}H NMR (400 MHz, $CDCl_3$) for ***(E)-6-(2-bromophenyl)hex-5-en-1-ol (1n)***

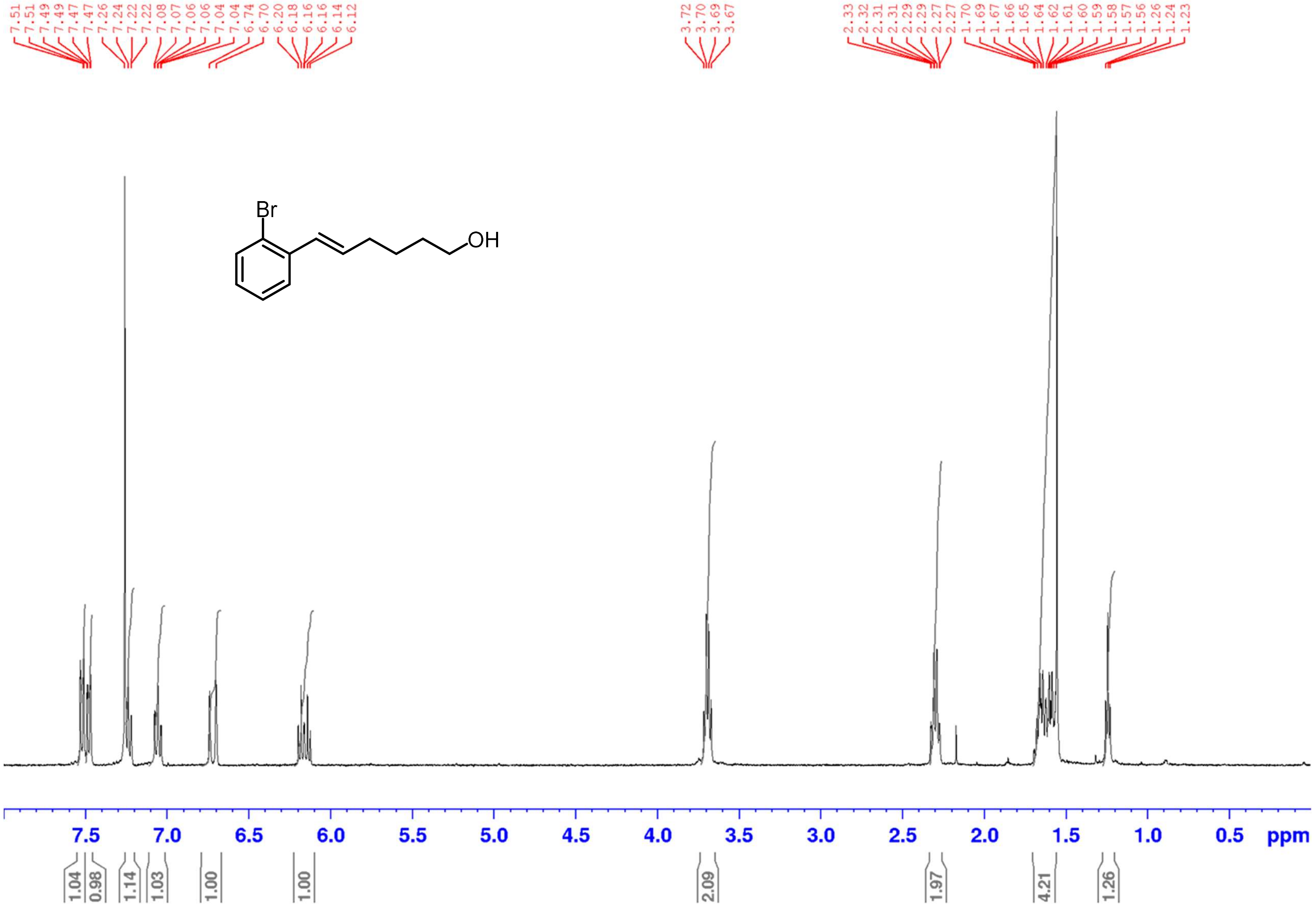

^{13}C NMR (101 MHz, $CDCl_3$) for ***(E)-6-(2-bromophenyl)hex-5-en-1-ol (1n)***

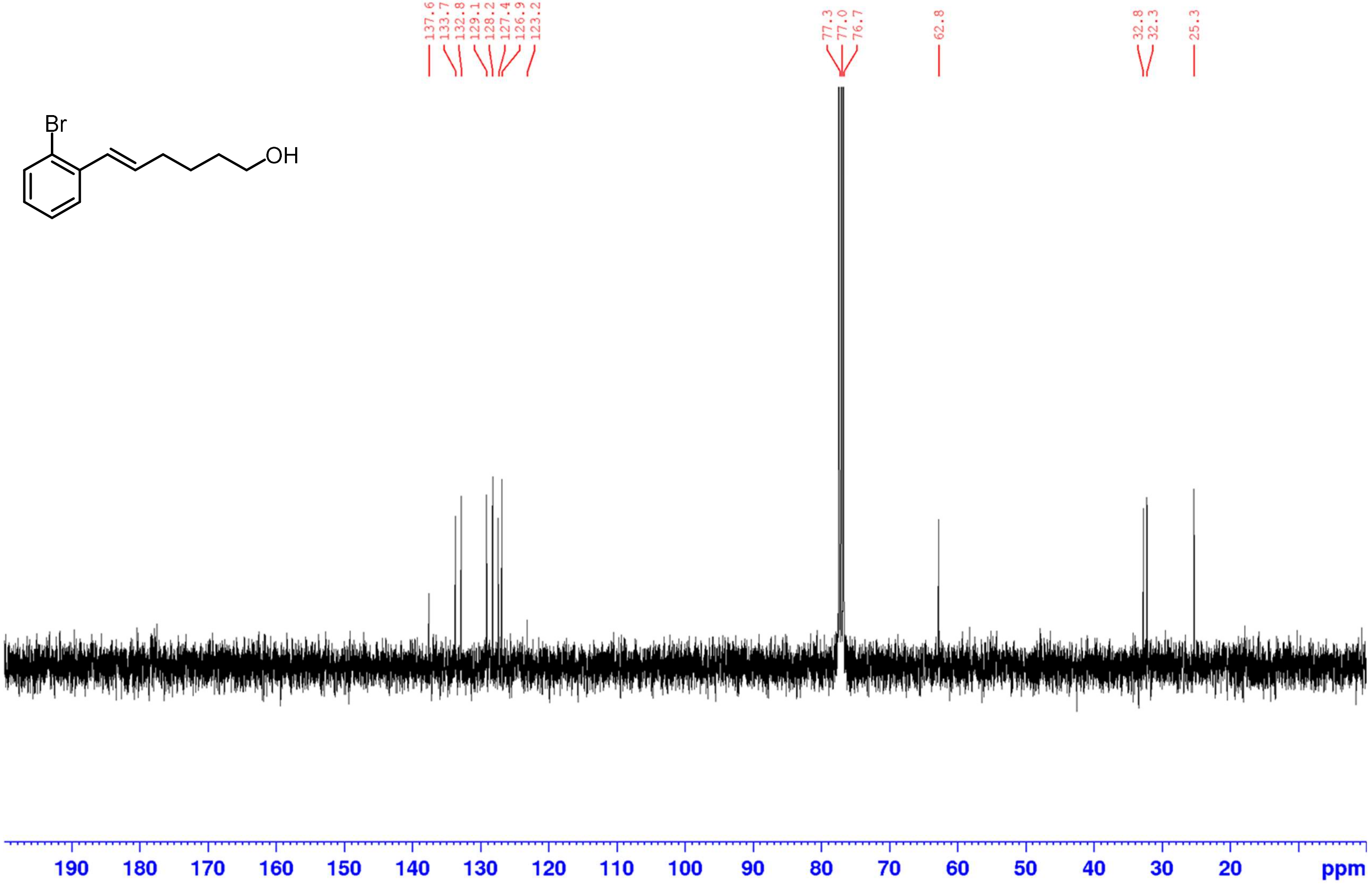

^{1}H NMR (700 MHz, $CDCl_3$) for ***(E)-6-(2-ethylphenyl)hex-5-en-1-ol (1o)***

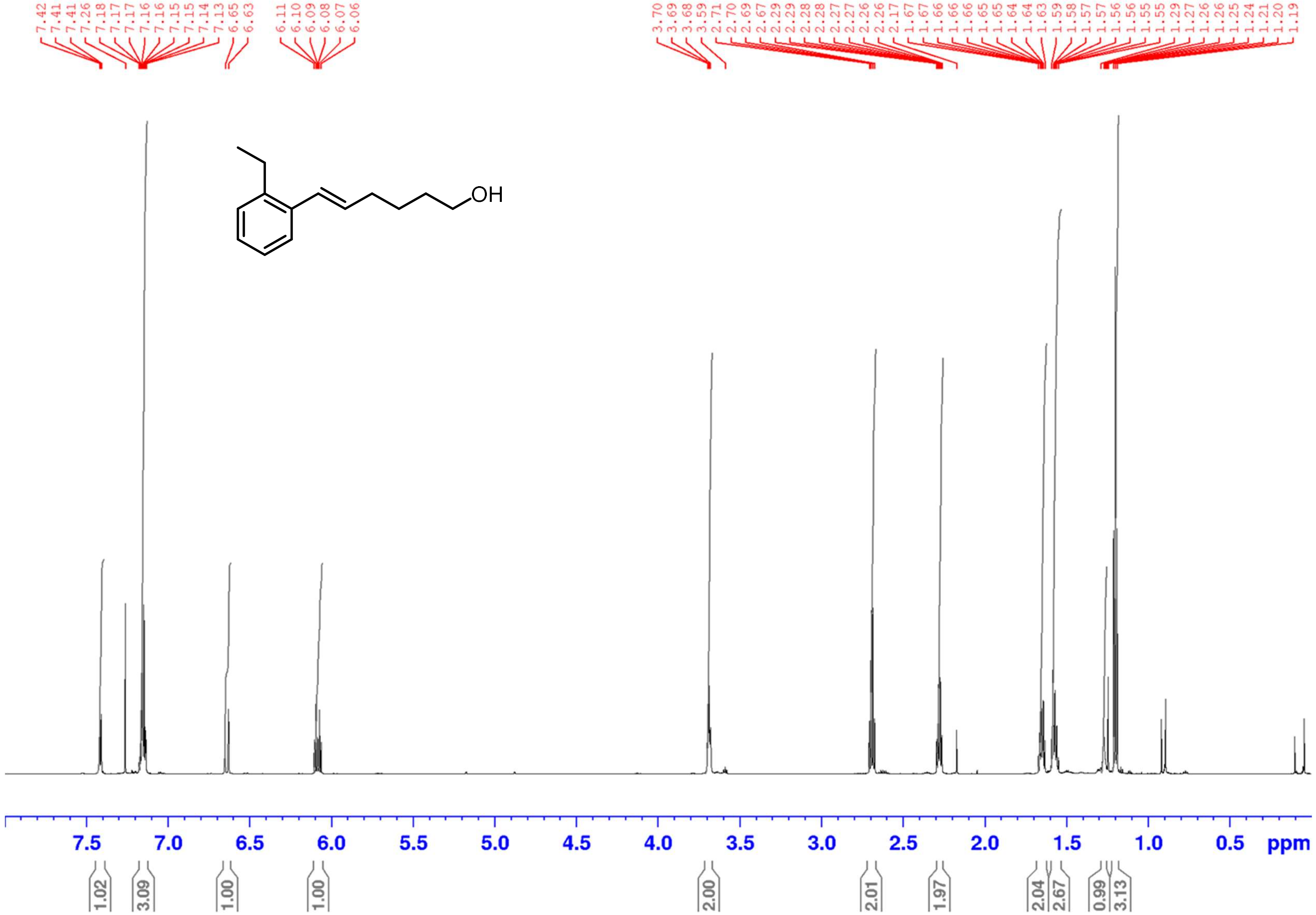

^{13}C NMR (176 MHz, $CDCl_3$) for ***(E)-6-(2-ethylphenyl)hex-5-en-1-ol (1o)***

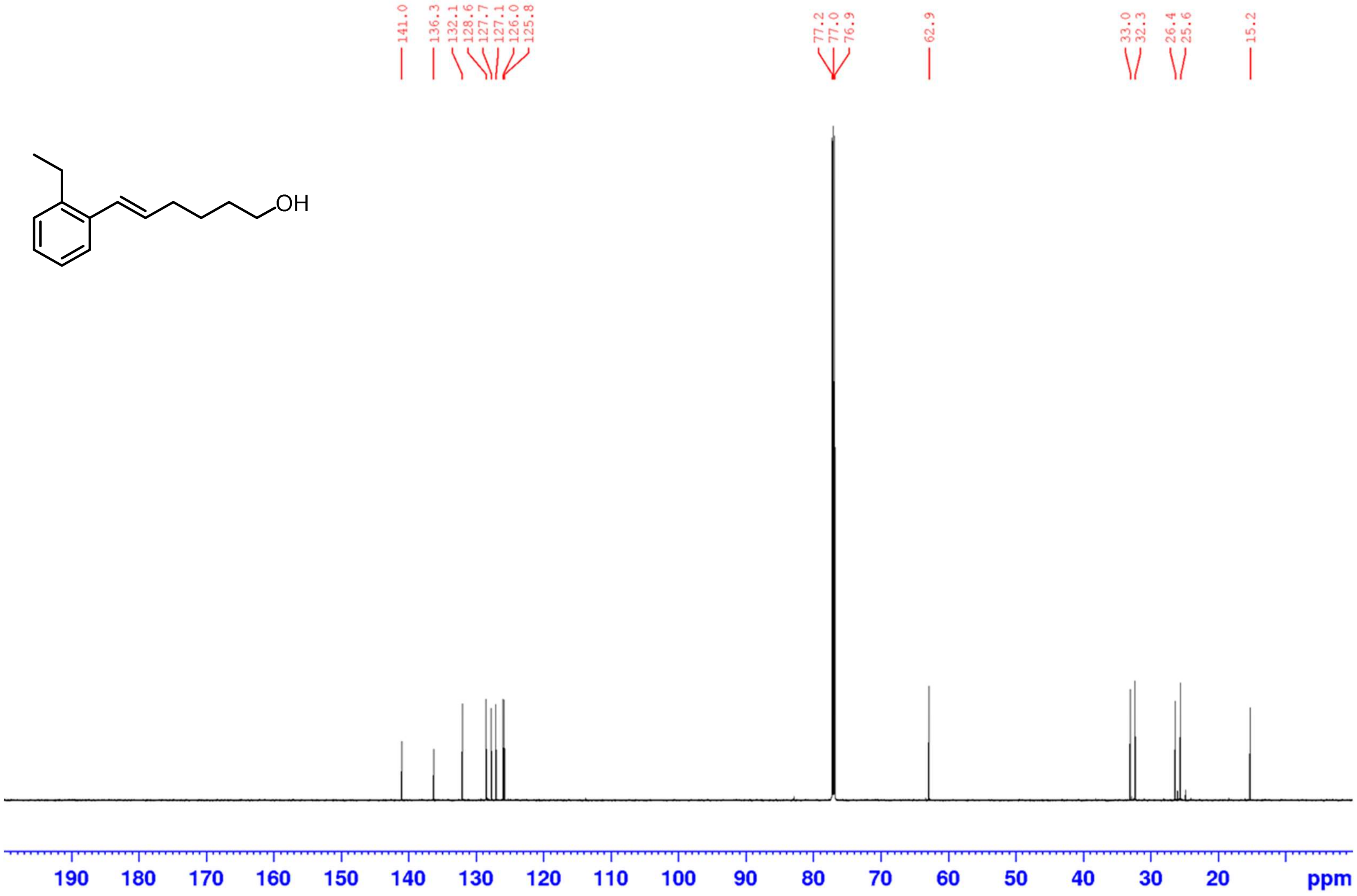

^{1}H NMR (700 MHz, $CDCl_3$) for ***(E)-6-mesitylhex-5-en-1-ol (1p)***

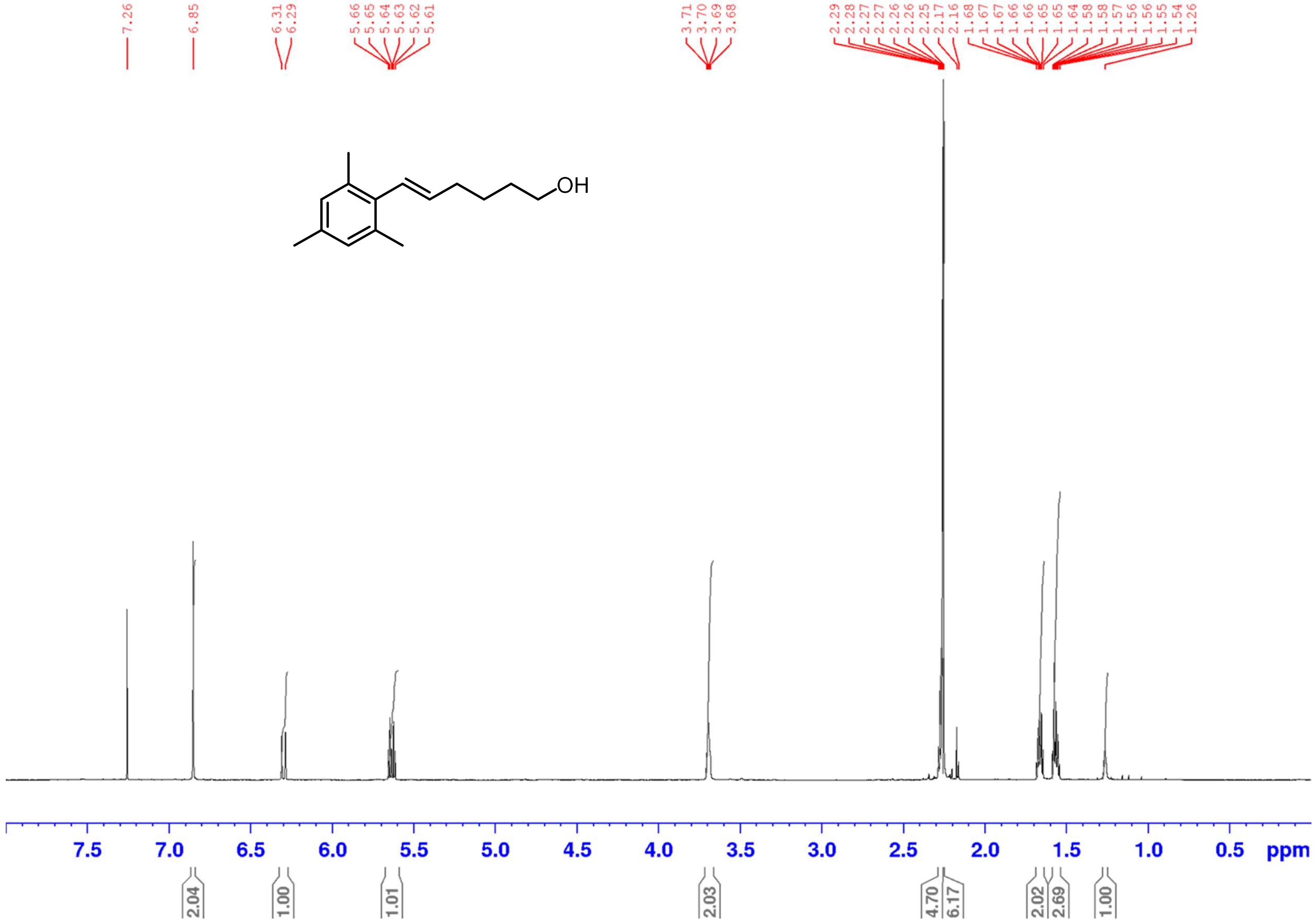

^{13}C NMR (176 MHz, $CDCl_3$) for ***(E)-6-mesitylhex-5-en-1-ol (1p)***

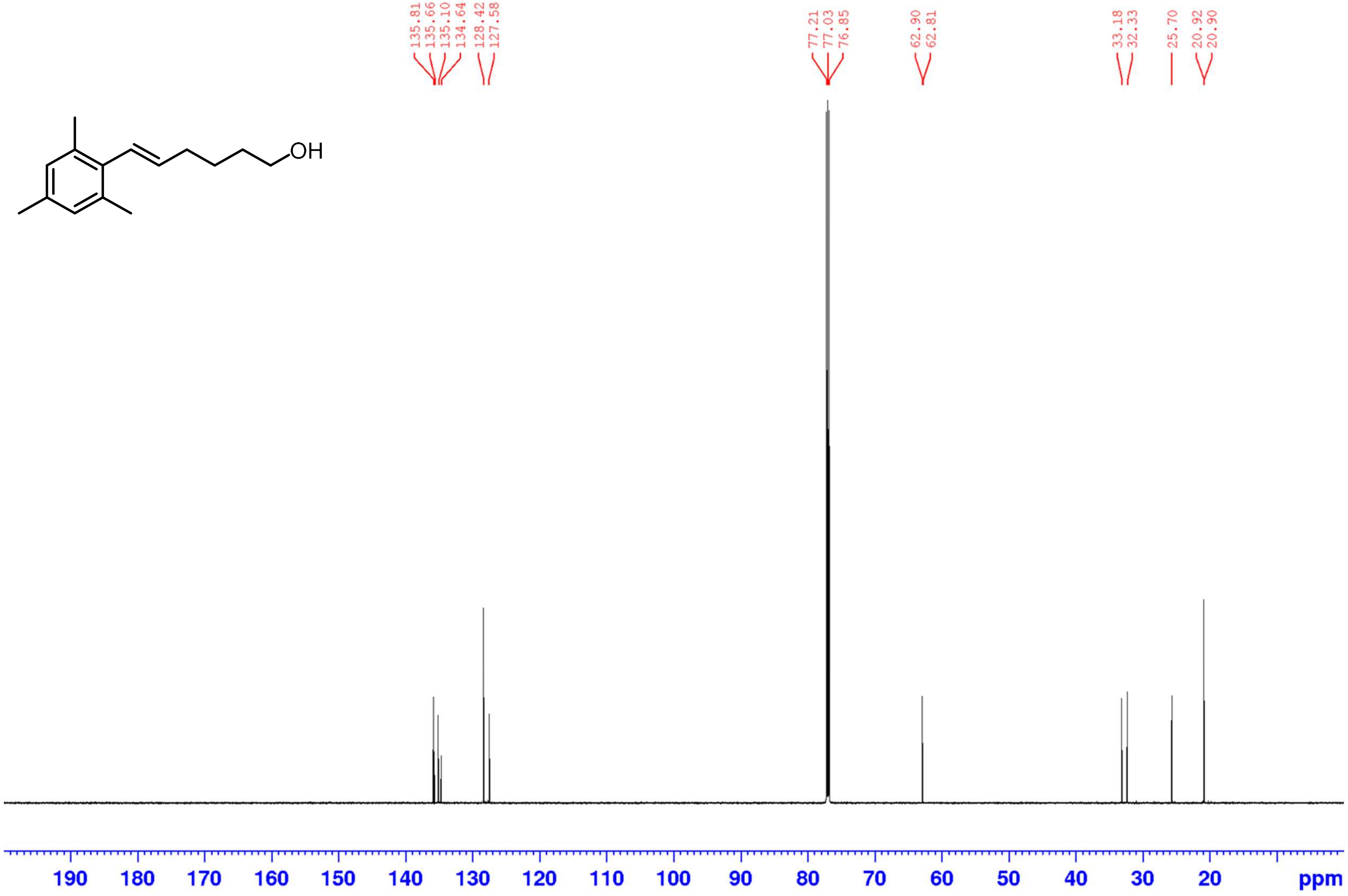

¹H NMR (700 MHz, $CDCl_3$) for ***(E)-2-methyl-7-phenylhept-6-en-2-ol (1q)***

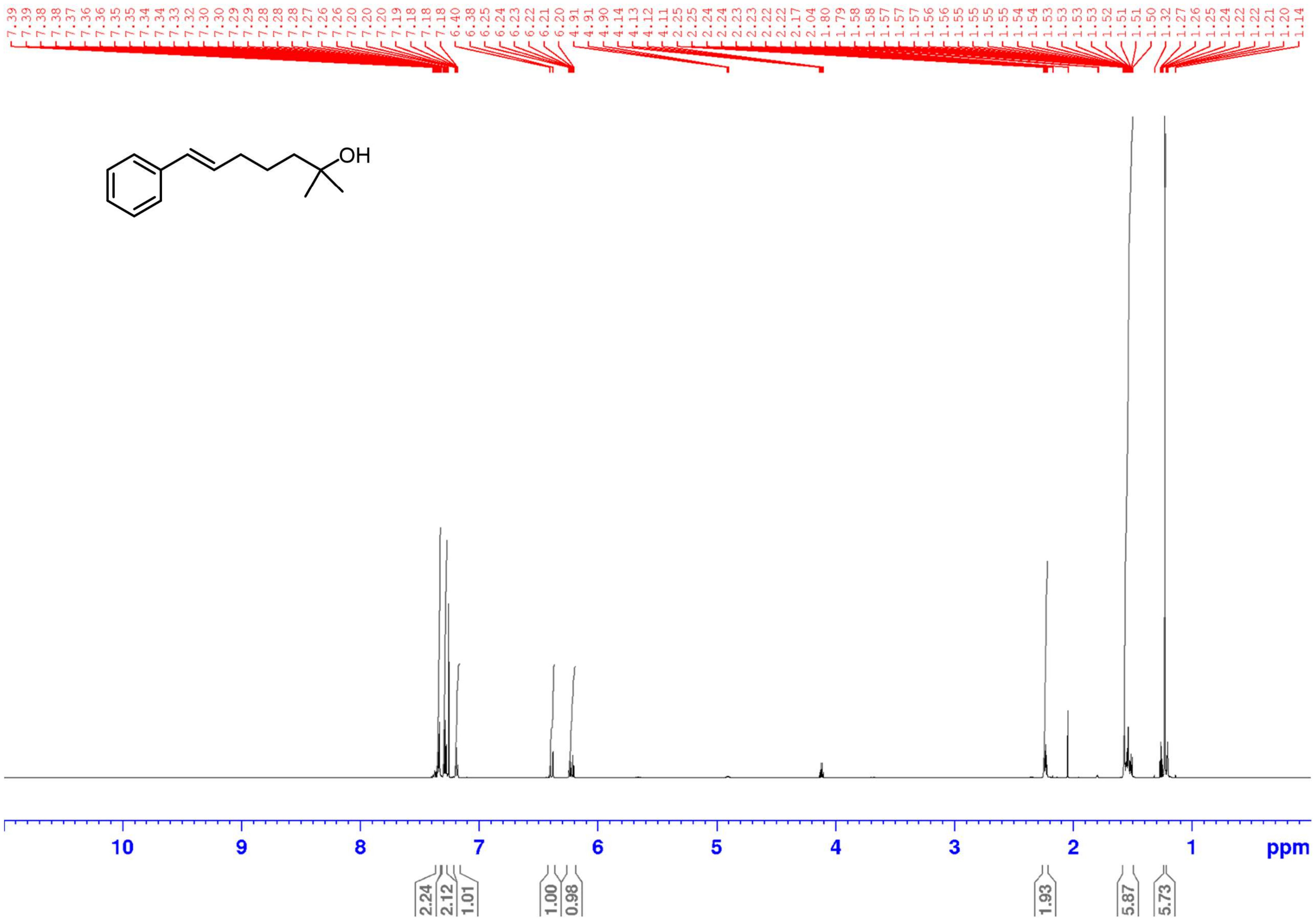

^{13}C NMR (176 MHz, $CDCl_3$) for ***(E)*-2-methyl-7-phenylhept-6-en-2-ol (1q)**

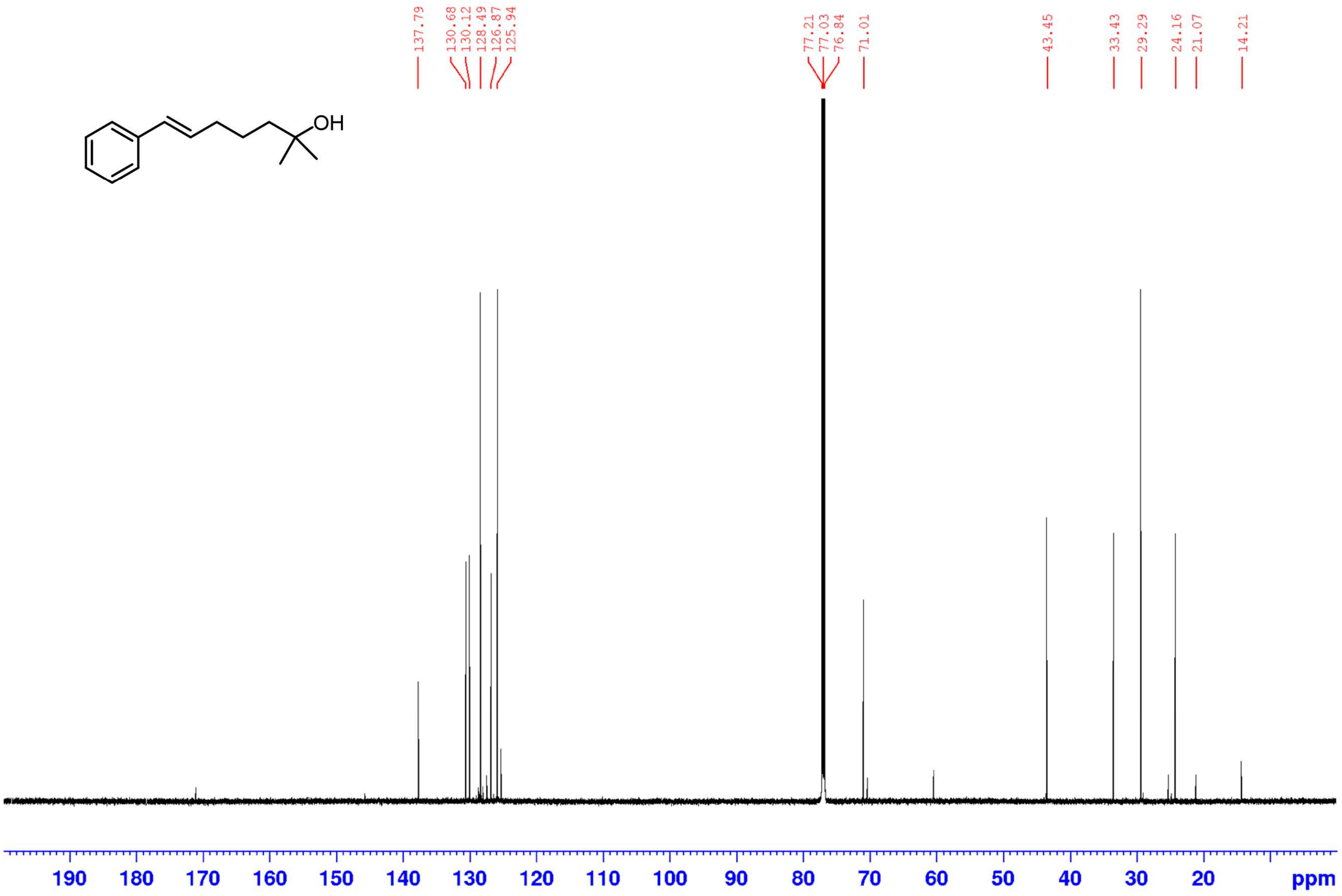

^{1}H NMR (700 MHz, $CDCl_3$) for ***(E)-6-(3,4,5-trimethoxyphenyl)hex-5-en-1-ol (1r)***

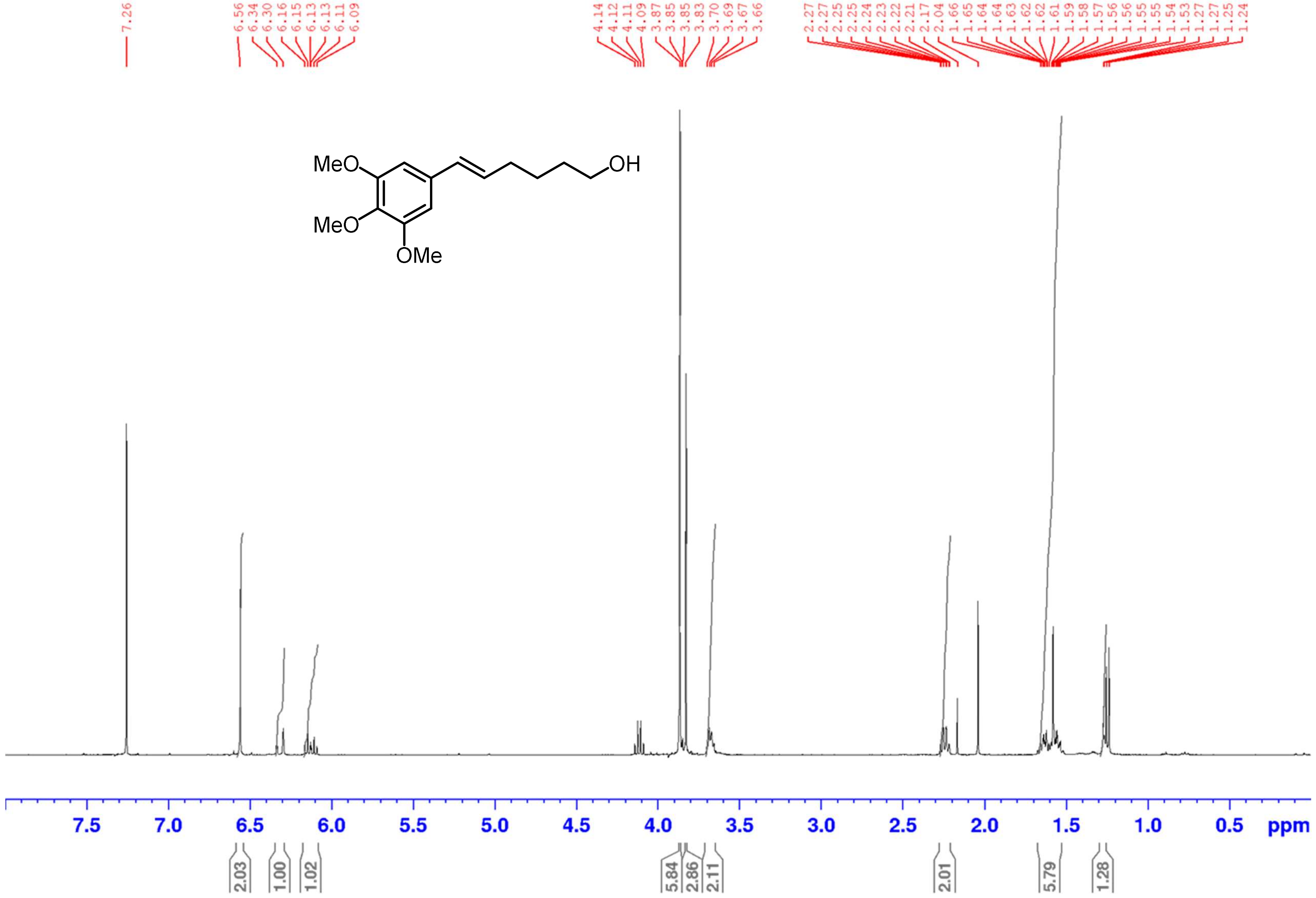

^{13}C NMR (176 MHz, $CDCl_3$) for ***(E)-6-(3,4,5-trimethoxyphenyl)hex-5-en-1-ol (1r)***

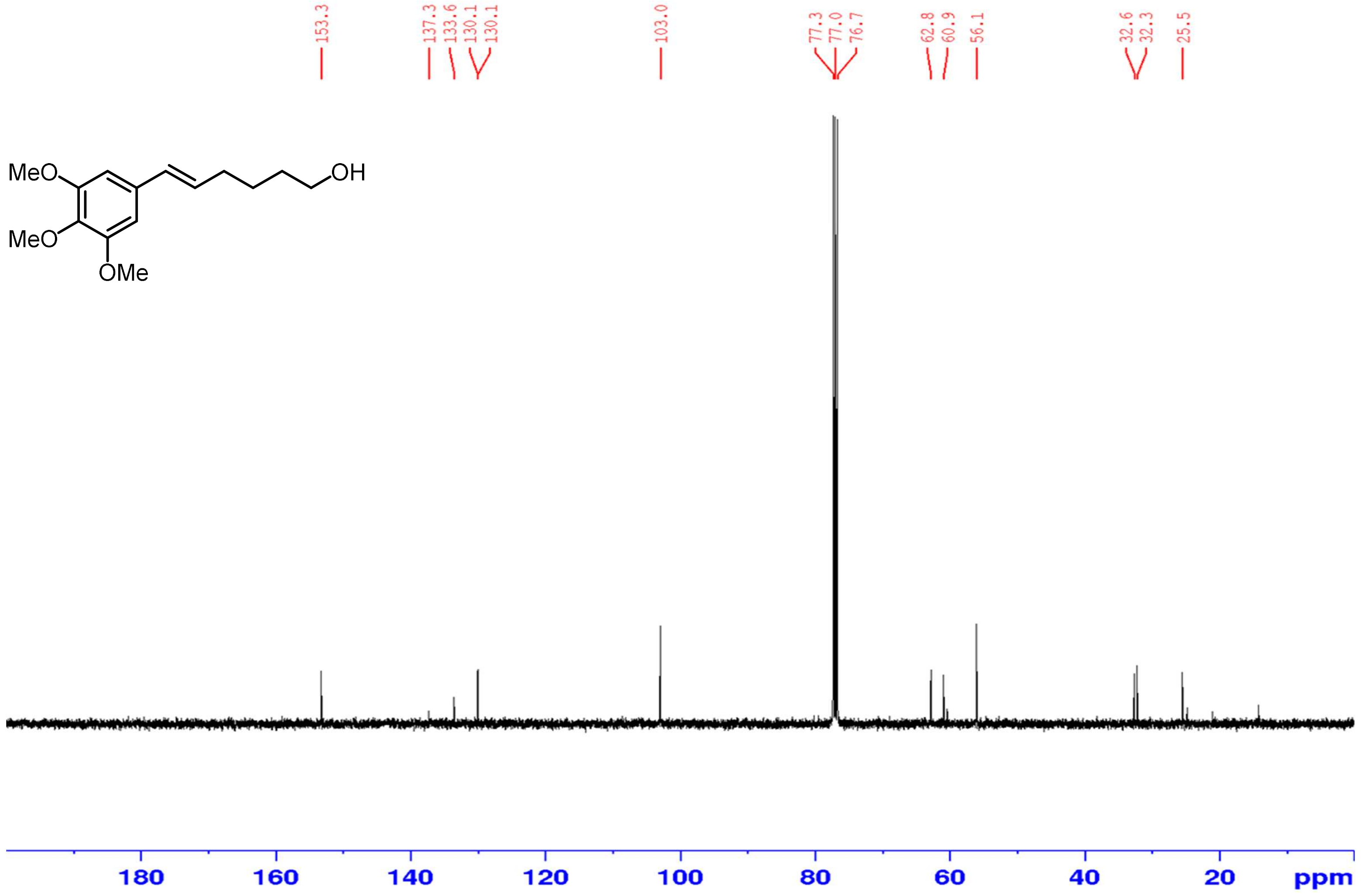

^{1}H NMR (400 MHz, $CDCl_3$) for ***(E)-dec-5-en-1-ol***

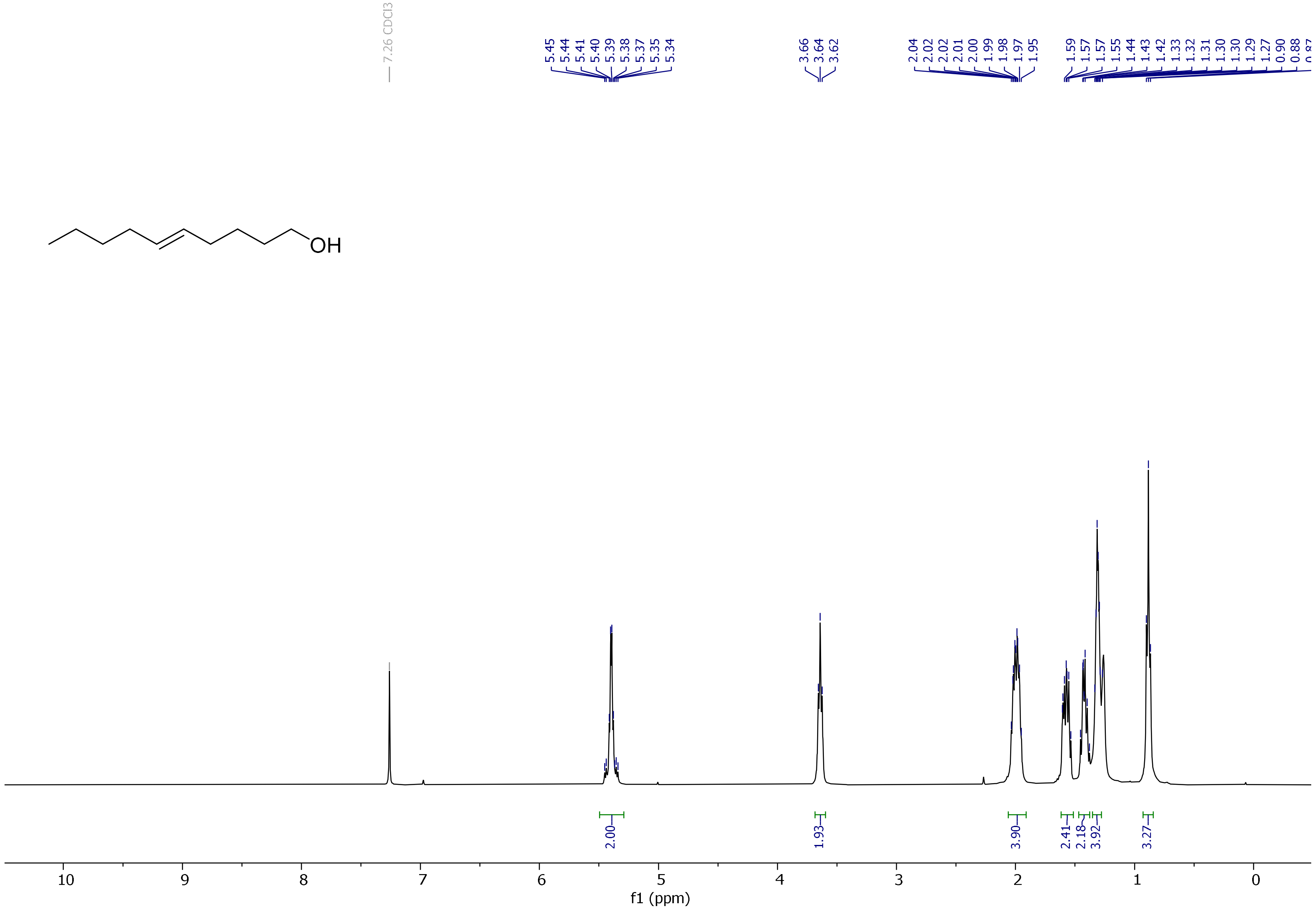

^{13}C NMR (176 MHz, $CDCl_3$) for ***(E)-dec-5-en-1-ol***

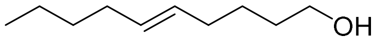

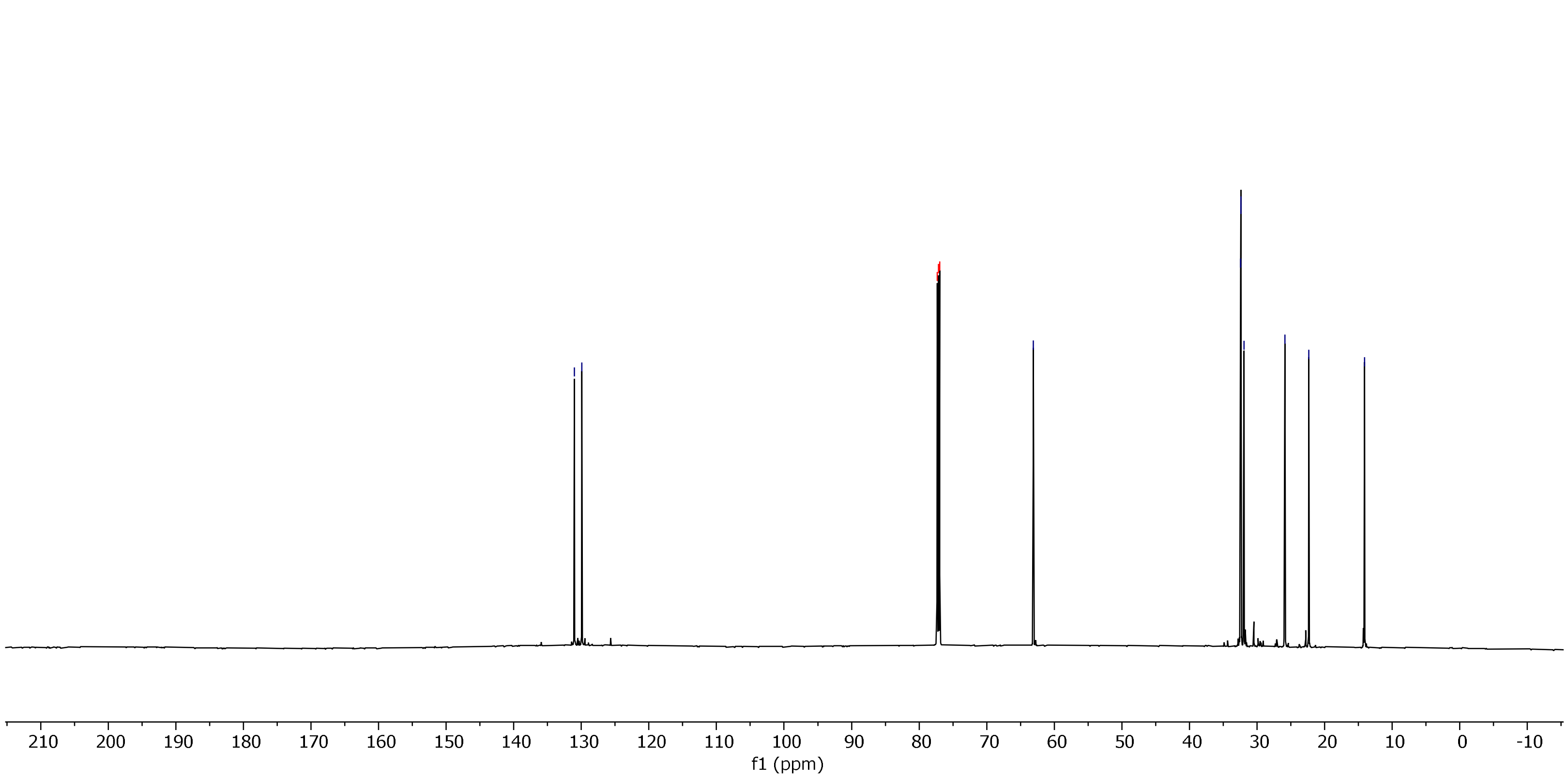

¹H NMR (700 MHz, $CDCl_3$) for ***Methyl 5-bromovalerate***

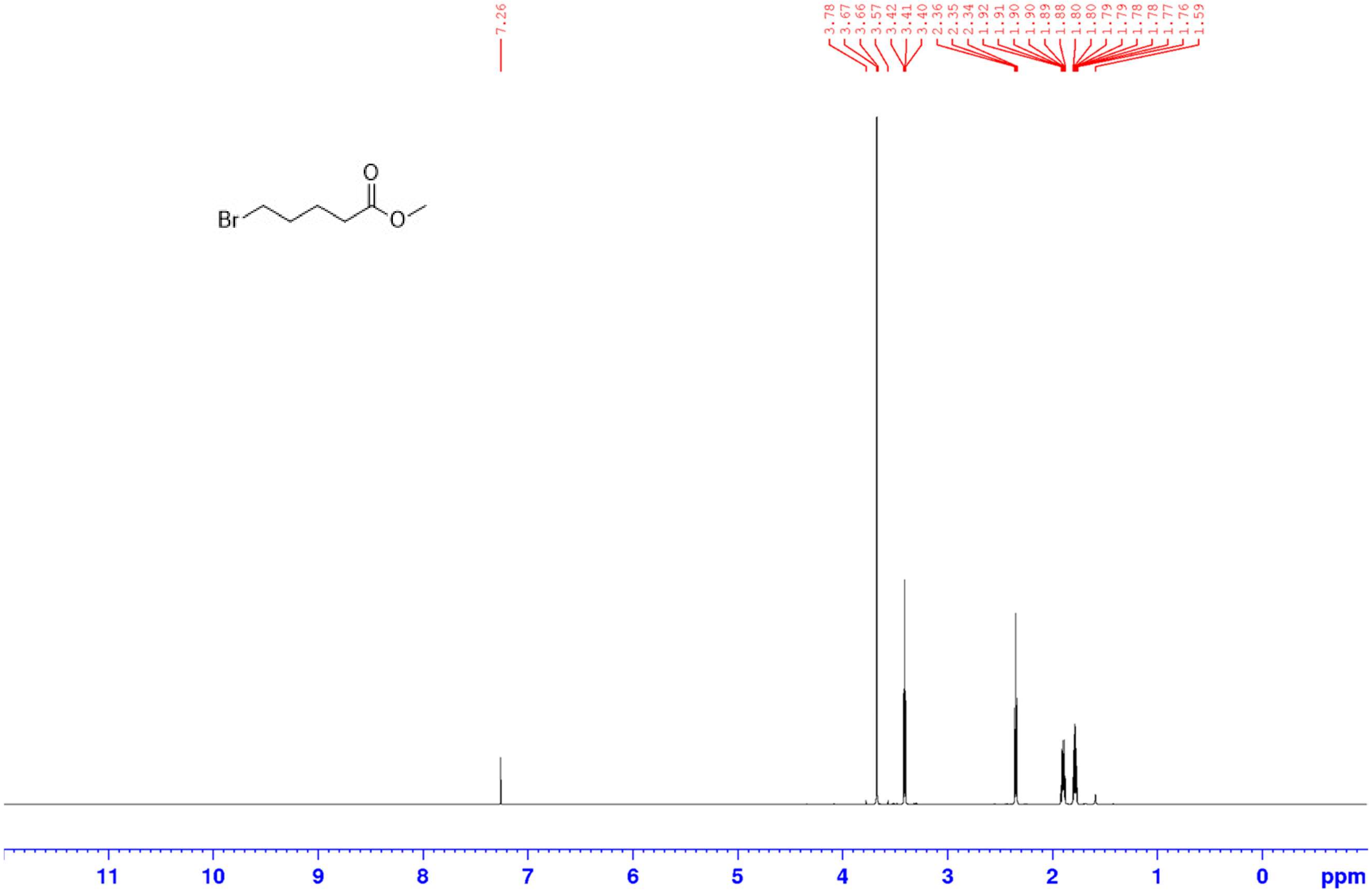

^{13}C NMR (101 MHz, $CDCl_3$) for ***Methyl 5-bromovalerate***

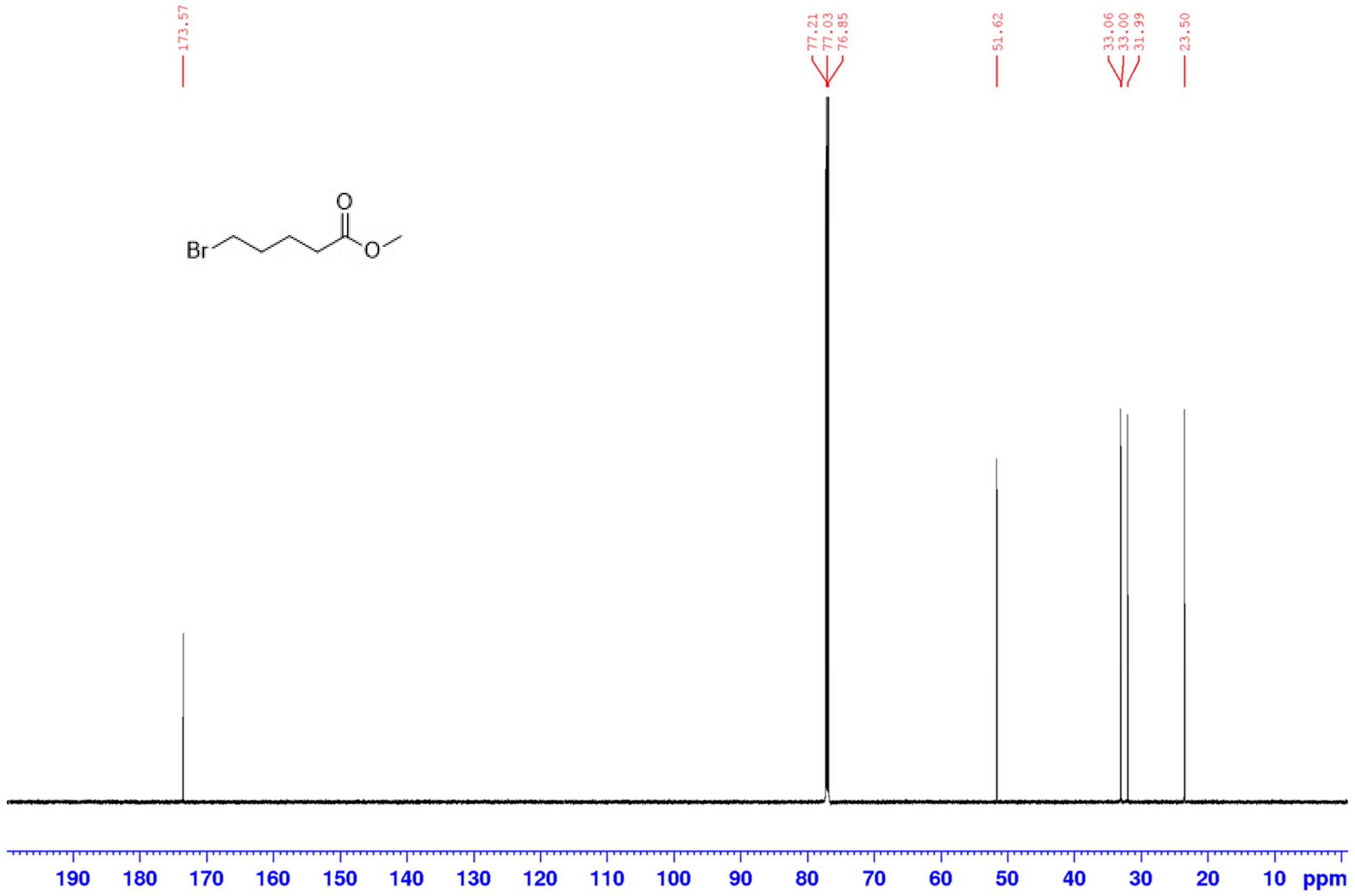

¹H NMR (400 MHz, $CDCl_3$) for ***Methyl 5-((1-phenyl-1H-tetrazol-5-yl)thio)pentanoate***

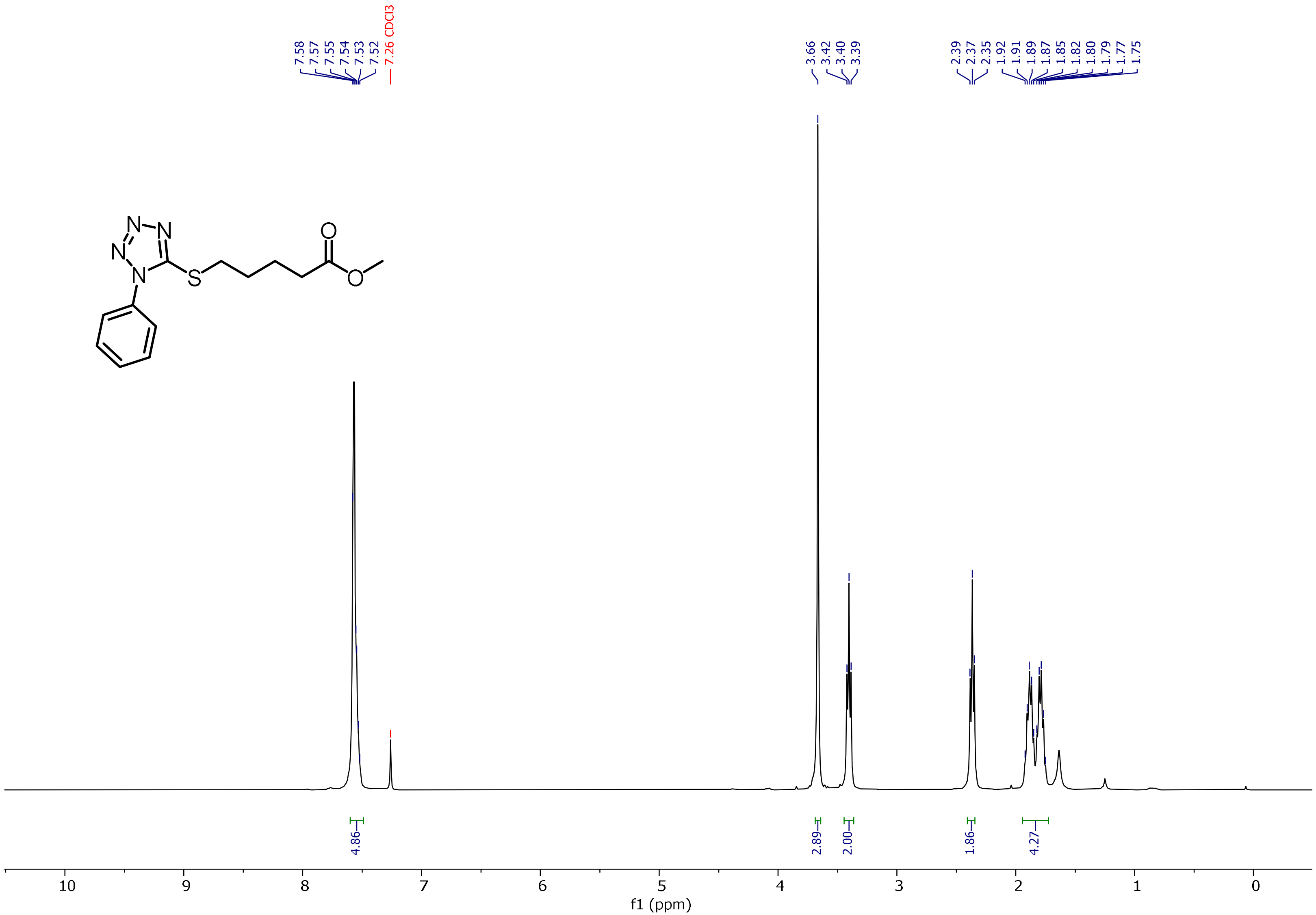

^{13}C NMR (176 MHz, $CDCl_3$) for ***Methyl 5-((1-phenyl-1H-tetrazol-5-yl)thio)pentanoate***

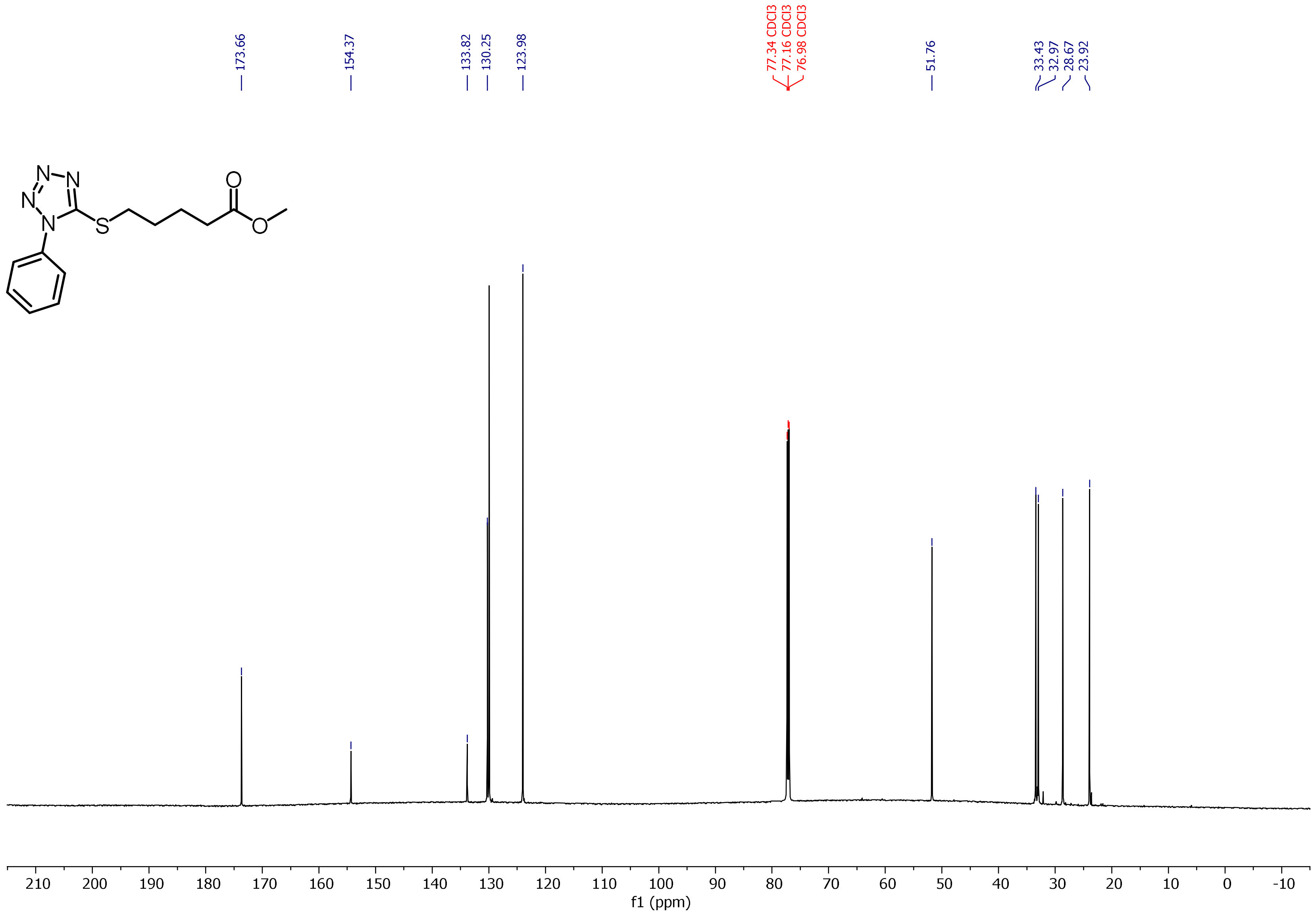

^{1}H NMR (700 MHz, $CDCl_3$) for ***Methyl 5-((1-phenyl-1H-tetrazol-5-yl)sulfonyl)pentanoate***

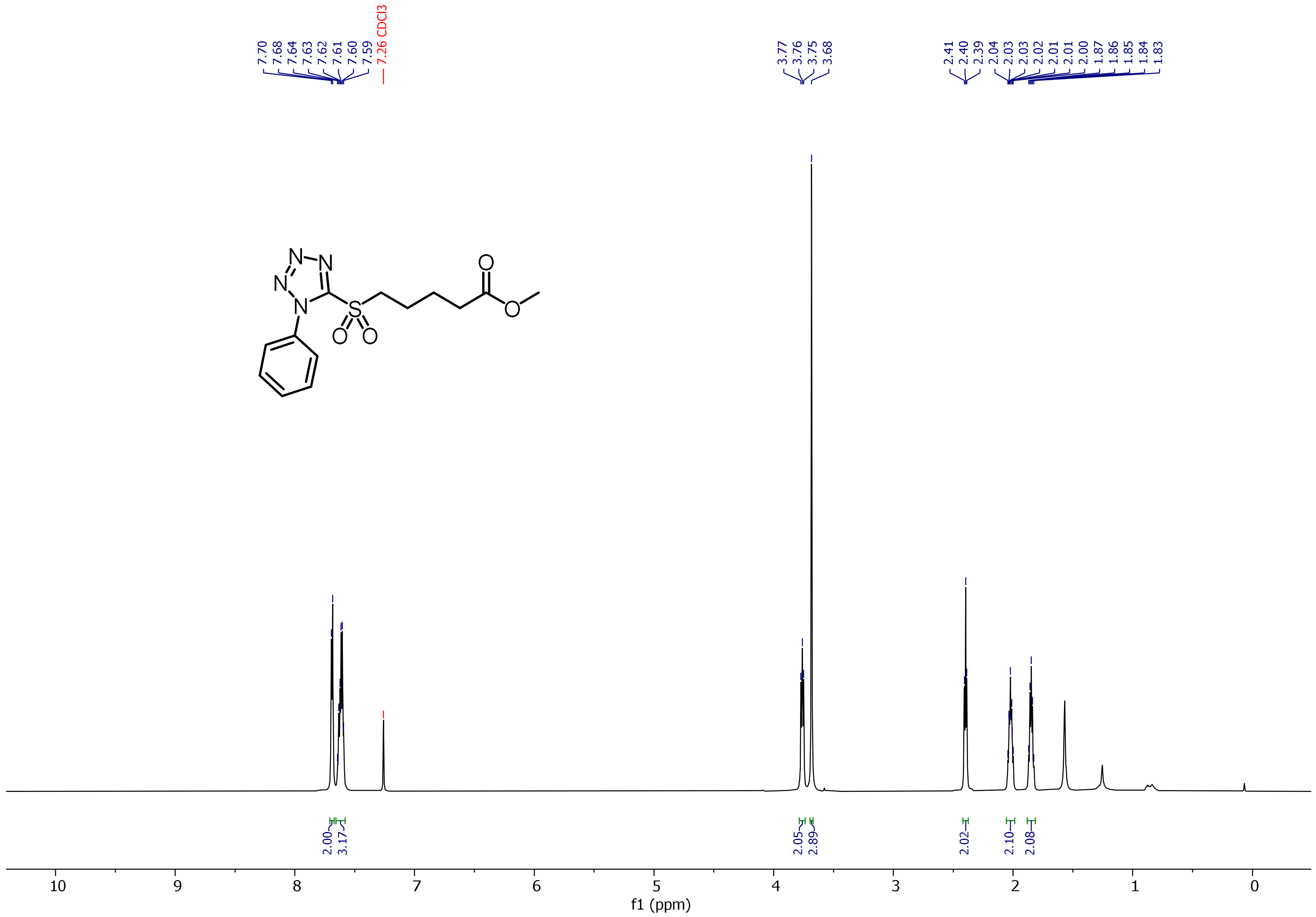

^{13}C NMR (176 MHz, $CDCl_3$) for ***Methyl 5-((1-phenyl-1H-tetrazol-5-yl)sulfonyl)pentanoate***

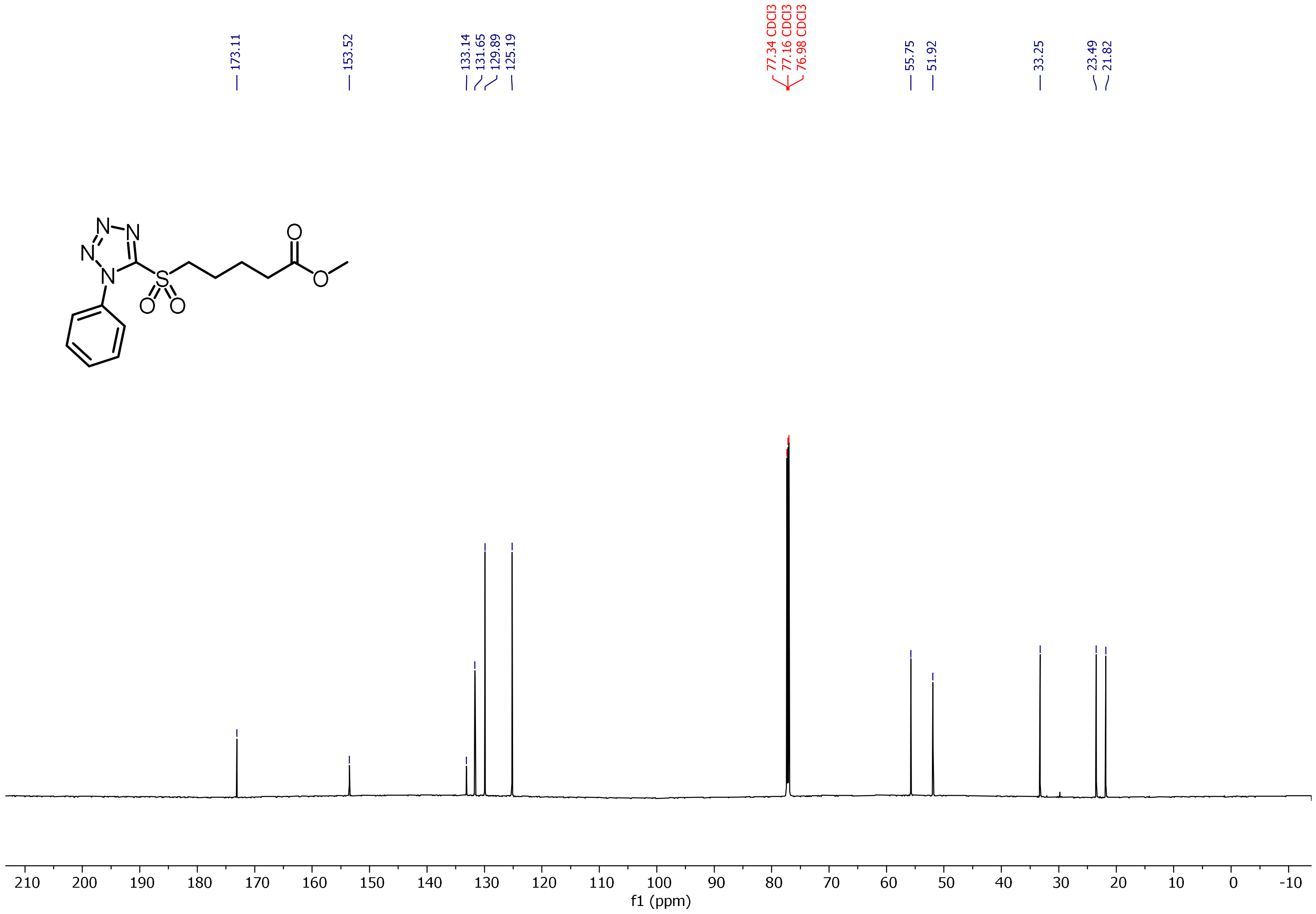

^{1}H NMR (700 MHz, $CDCl_3$) for ***Methyl (E)-6-phenylhex-5-enoate***

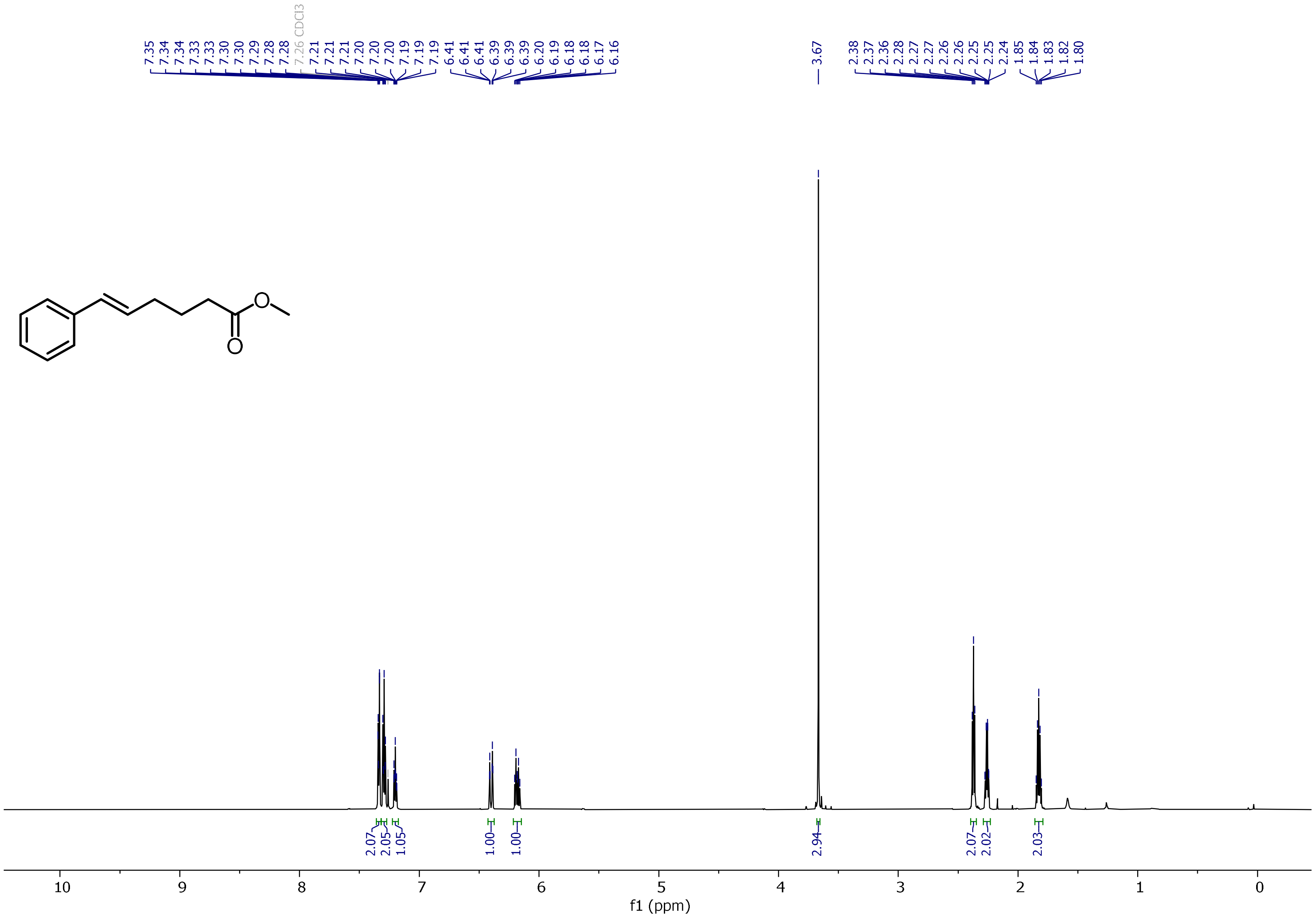

^{13}C NMR (176 MHz, $CDCl_3$) for ***Methyl (E)-6-phenylhex-5-enoate***

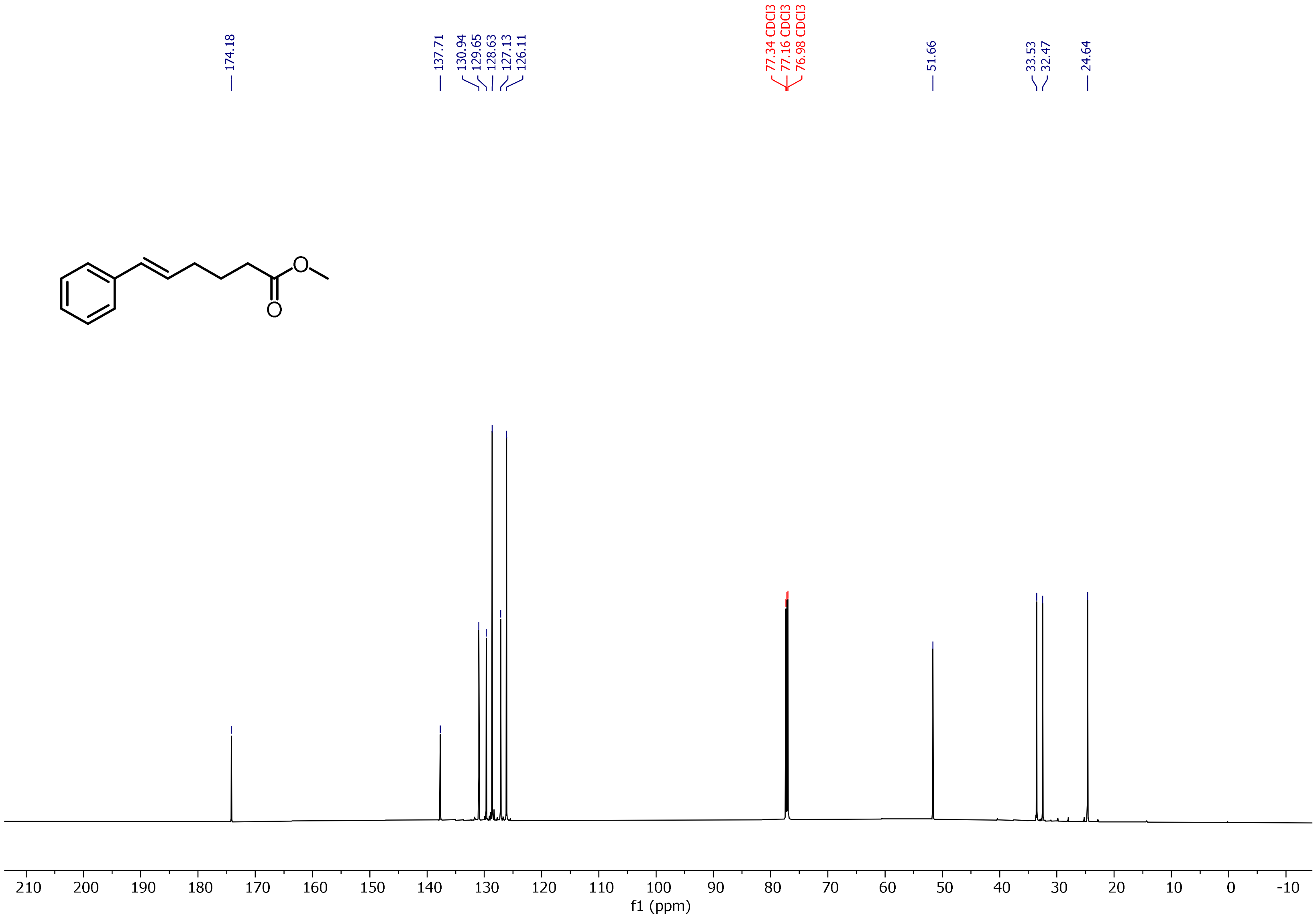

^{1}H NMR (700 MHz, $CDCl_3$) for ***(E)-N-methyl-N,6-diphenylhex-5-enamide***

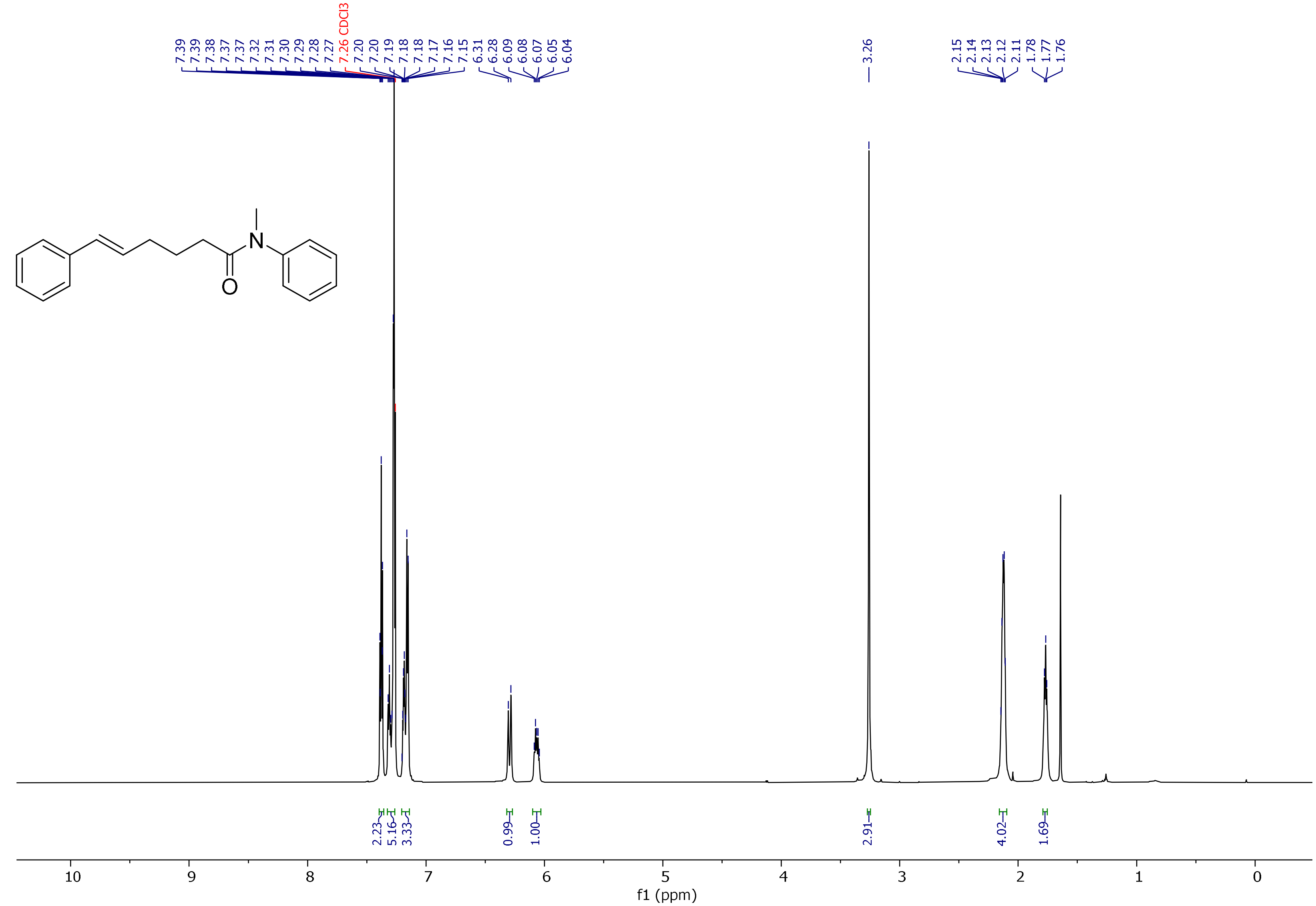

^{13}C NMR (176 MHz, $CDCl_3$) for ***(E)-N-methyl-N,6-diphenylhex-5-enamide***

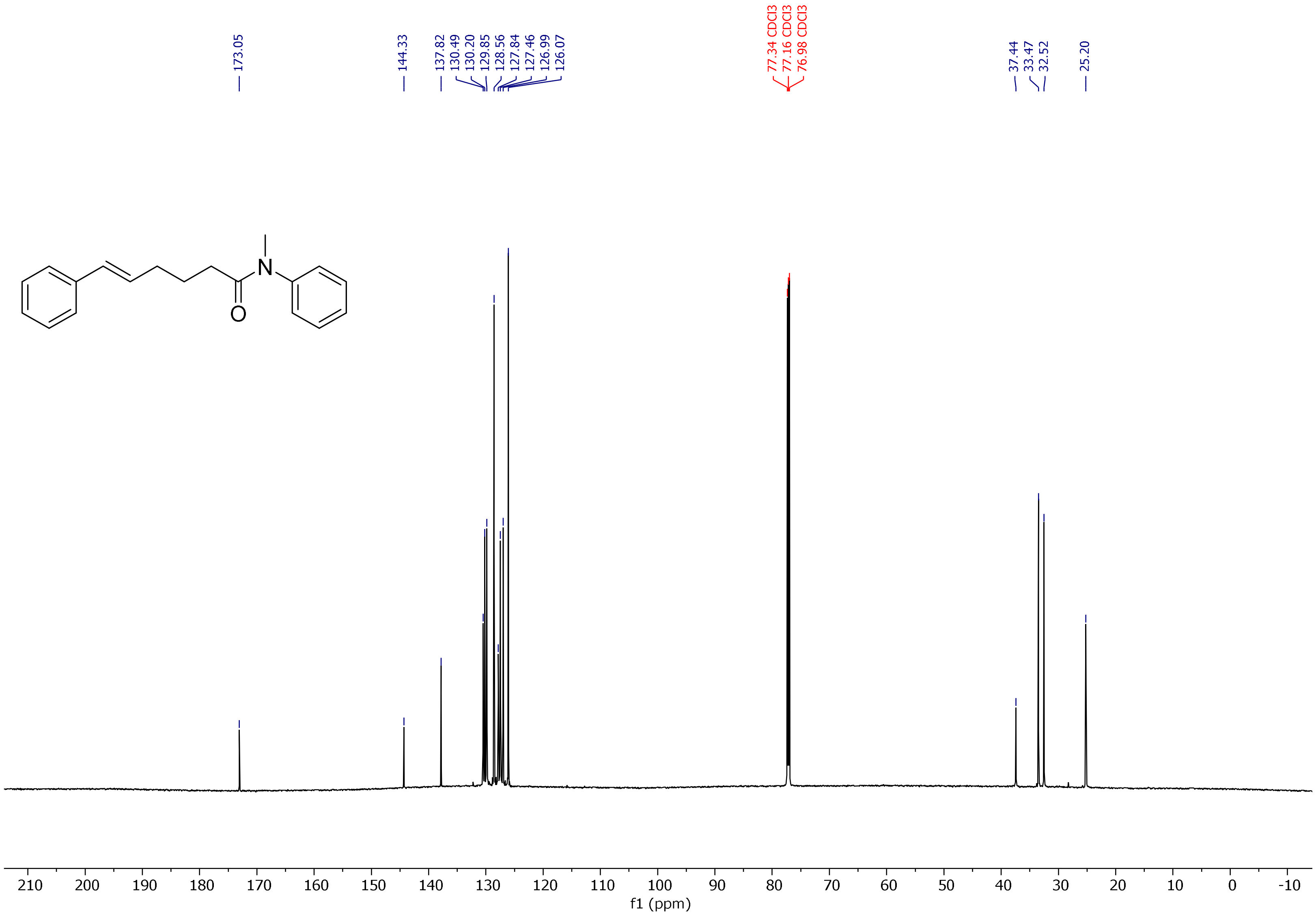
173.05
144.33
137.82
130.49
130.20
129.85
128.56
127.84
127.46
126.99
126.07
77.34 CDCl3
77.16 CDCl3
76.98 CDCl3
37.44
33.47
32.52
25.20
N
O
210 200 190 180 170 160 150 140 130 120 110 100 90 80 70 60 50 40 30 20 10 0 -10
f1 (ppm)

^{1}H NMR (400 MHz, $CDCl_3$) for ***tert-butyldimethyl(pent-4-yn-1-yloxy)silane***

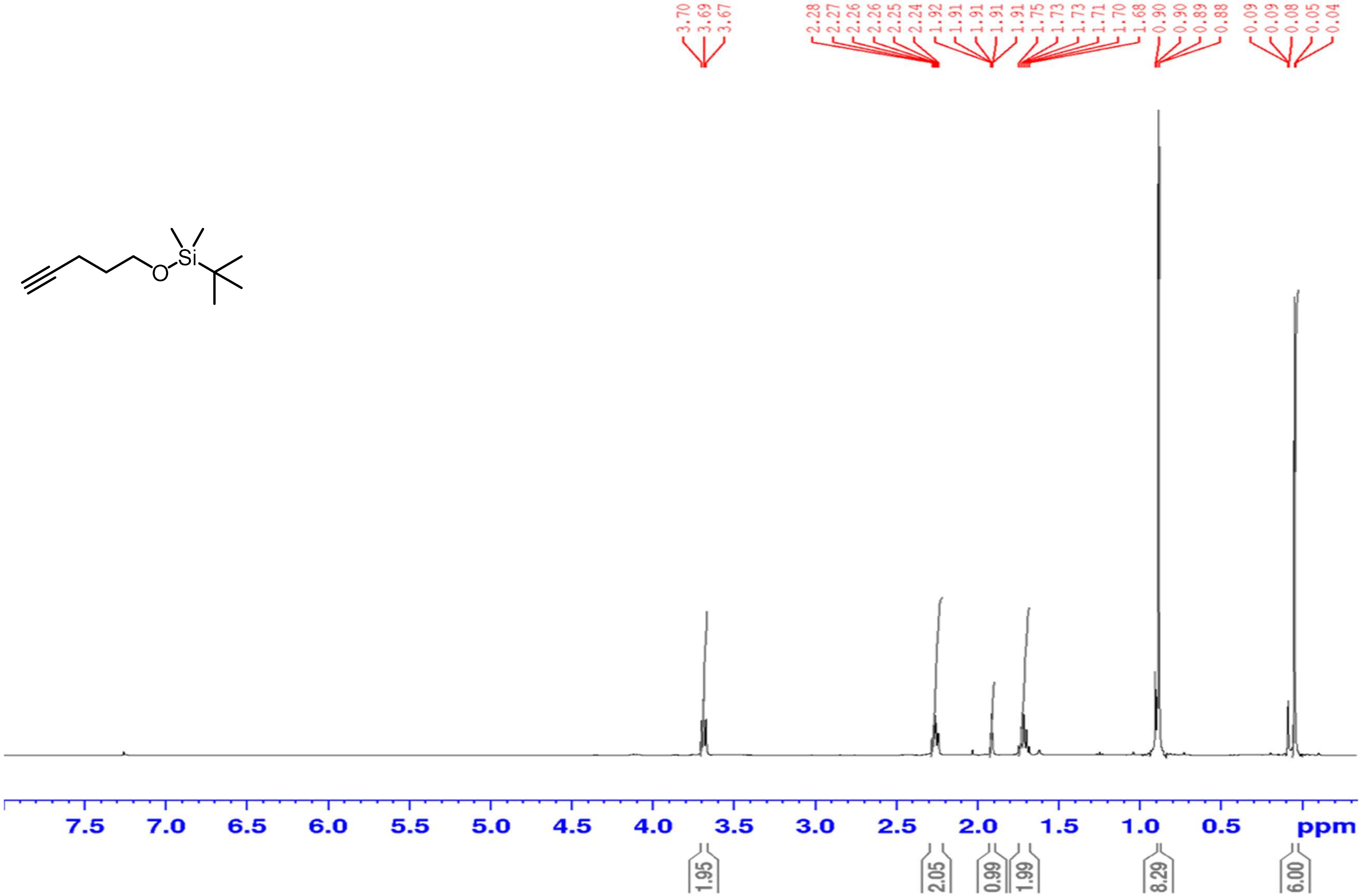

^{13}C NMR (101 MHz, $CDCl_3$) for ***tert-butyldimethyl(pent-4-yn-1-yloxy)silane***

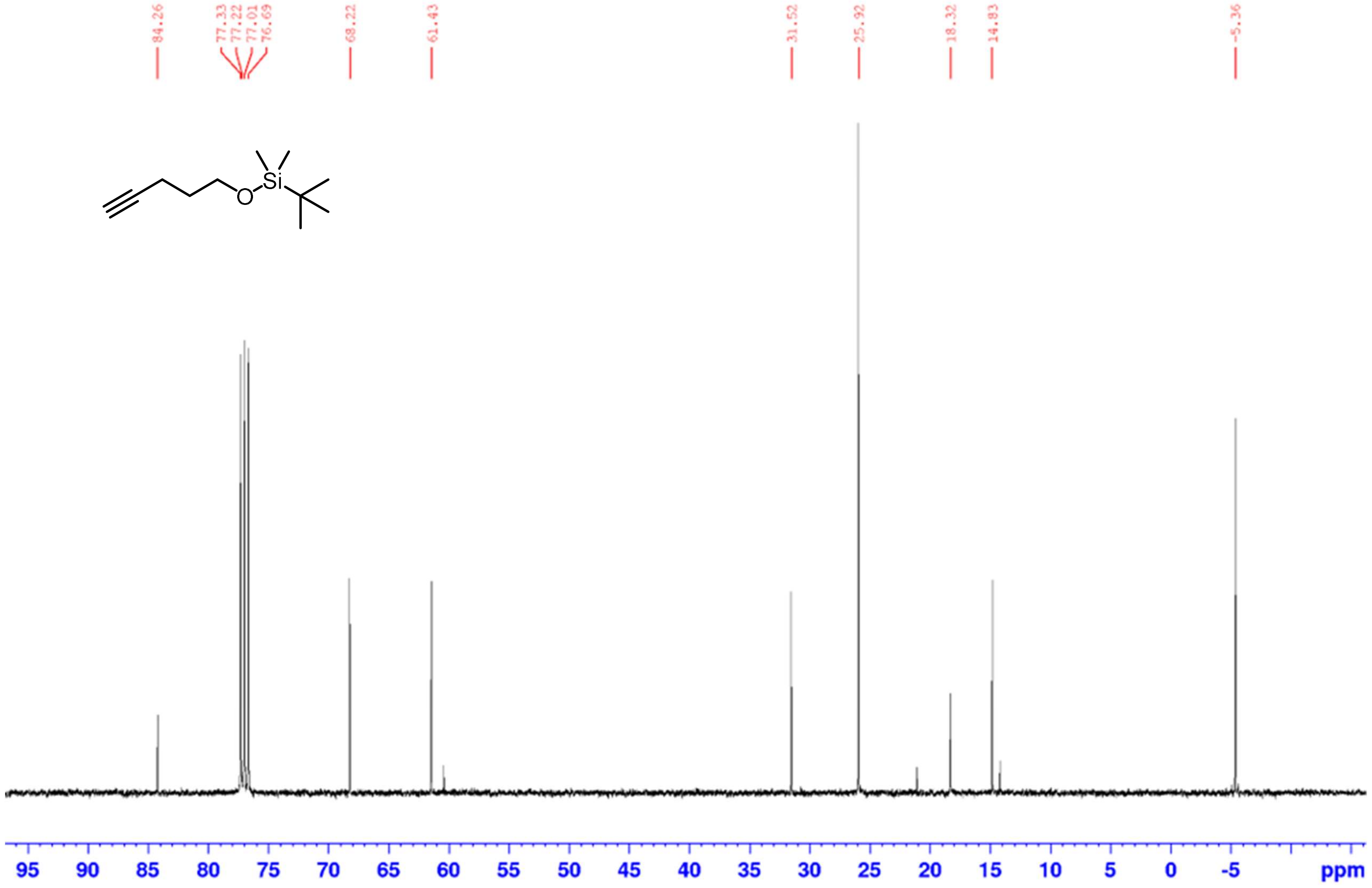

^{1}H NMR (700 MHz, $CDCl_3$) for ***(E)-tert-butyldimethyl((5-(4,4,5,5-tetramethyl-1,3,2-dioxaborolan-2-yl)pent-4-en-1-yl)oxy)silane***

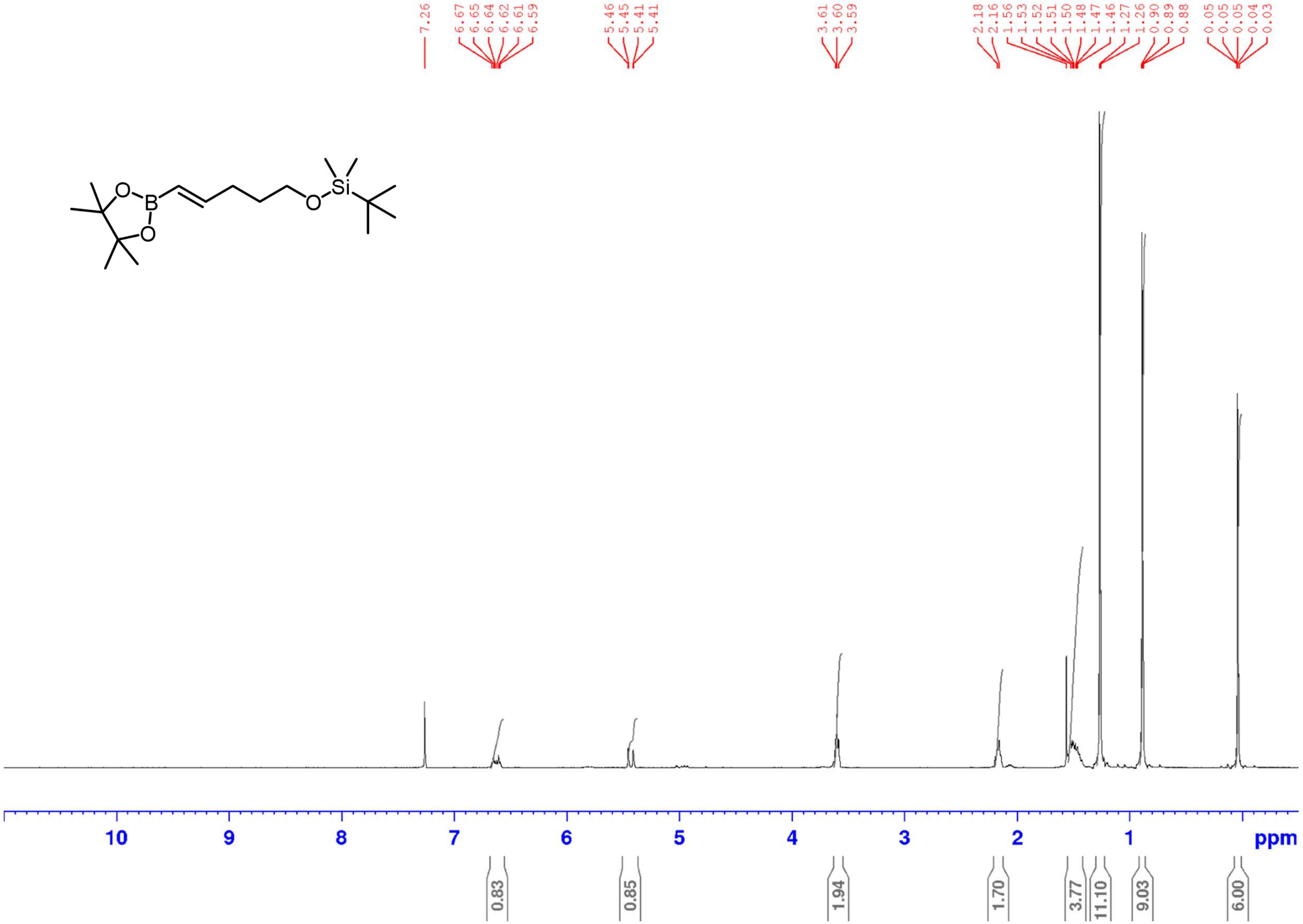

^{13}C NMR (176 MHz, $CDCl_3$) for ***(E)-tert-butyldimethyl((5-(4,4,5,5-tetramethyl-1,3,2-dioxaborolan-2-yl)pent-4-en-1-yl)oxy)silane***

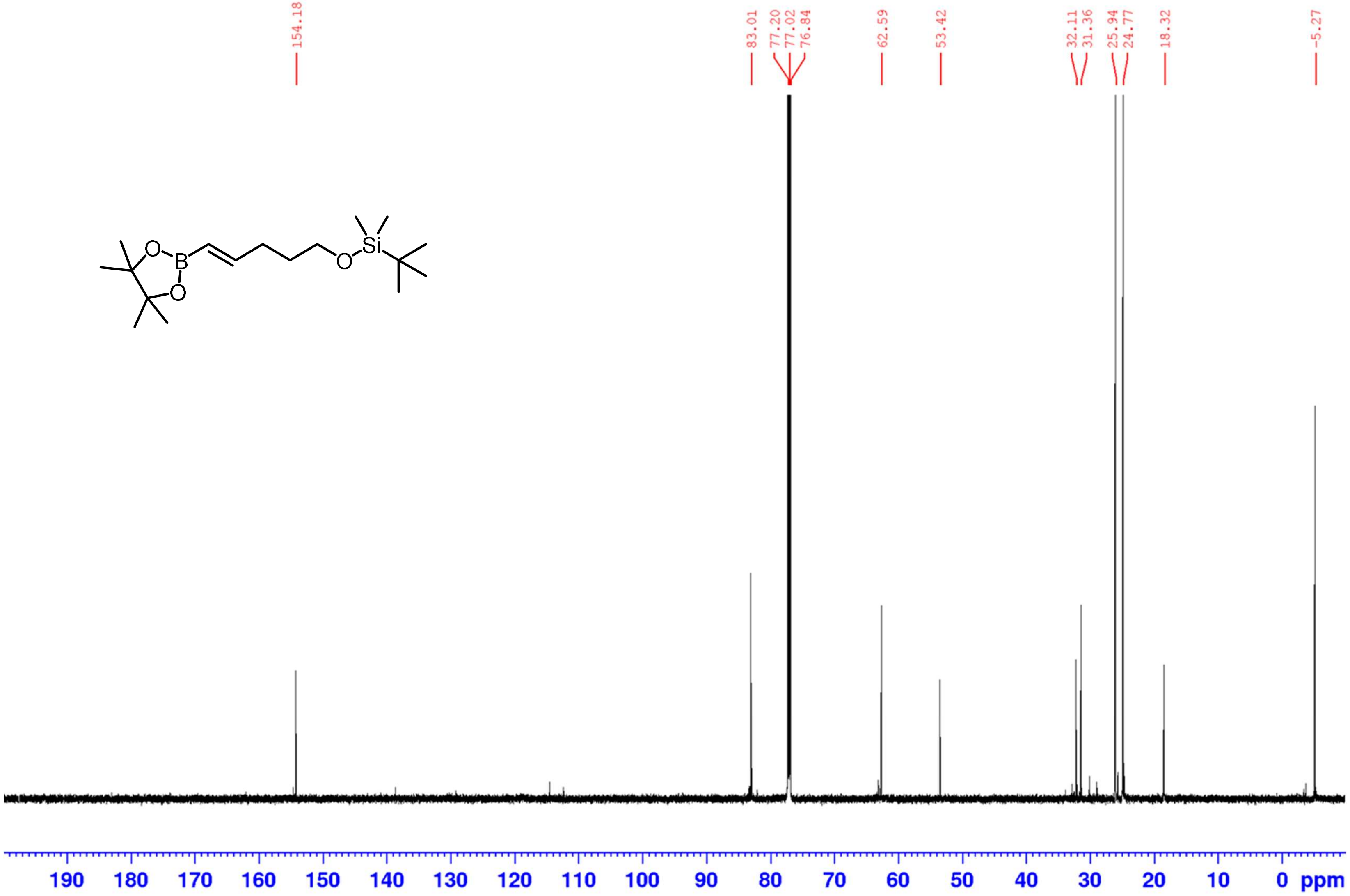

^{1}H NMR (700 MHz, $CDCl_3$) for ***(E)-5-phenylpent-4-en-1-ol (5a)***

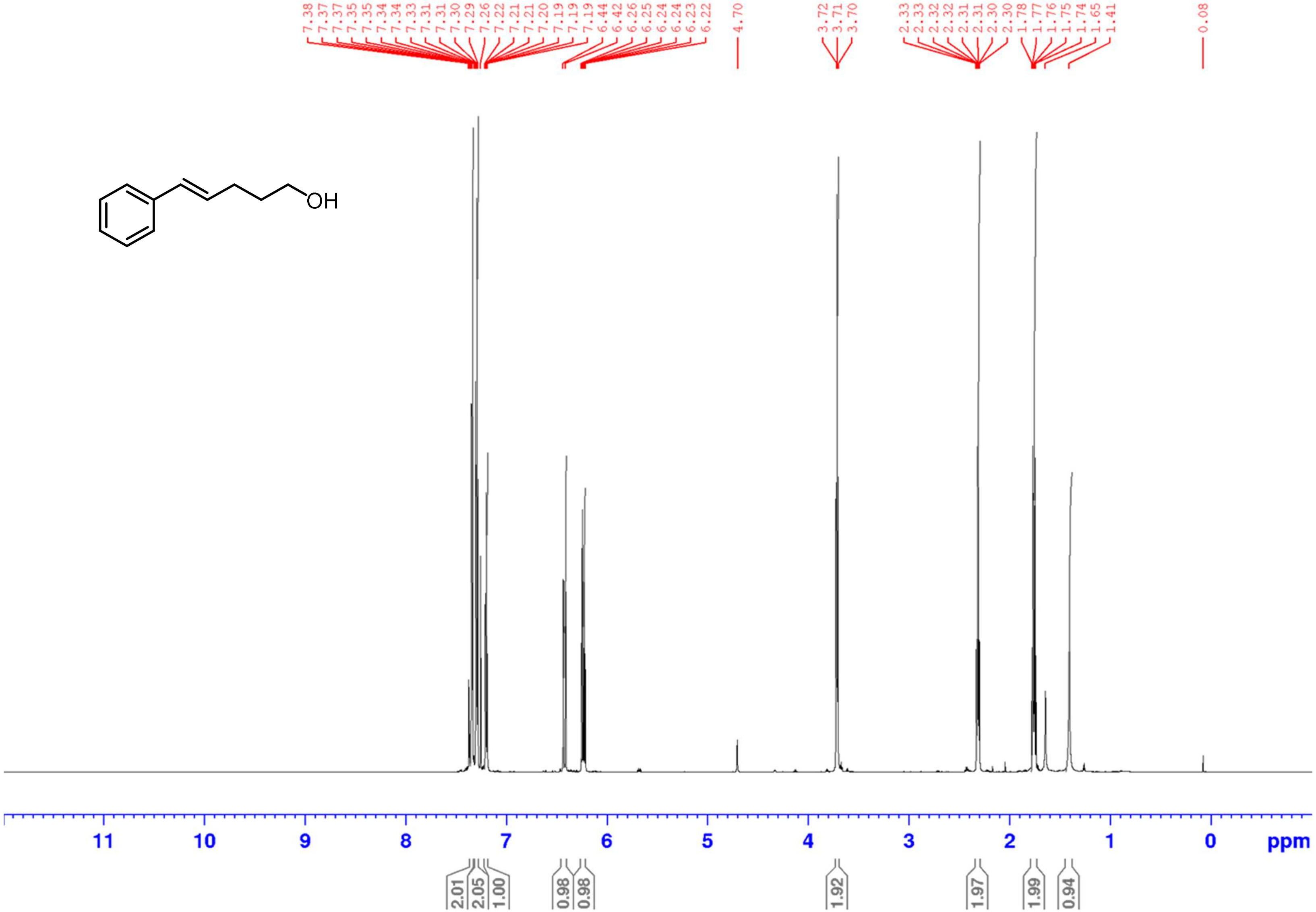

13C NMR (176 MHz, $CDCl_3$) for ***(E)-5-phenylpent-4-en-1-ol (5a)***

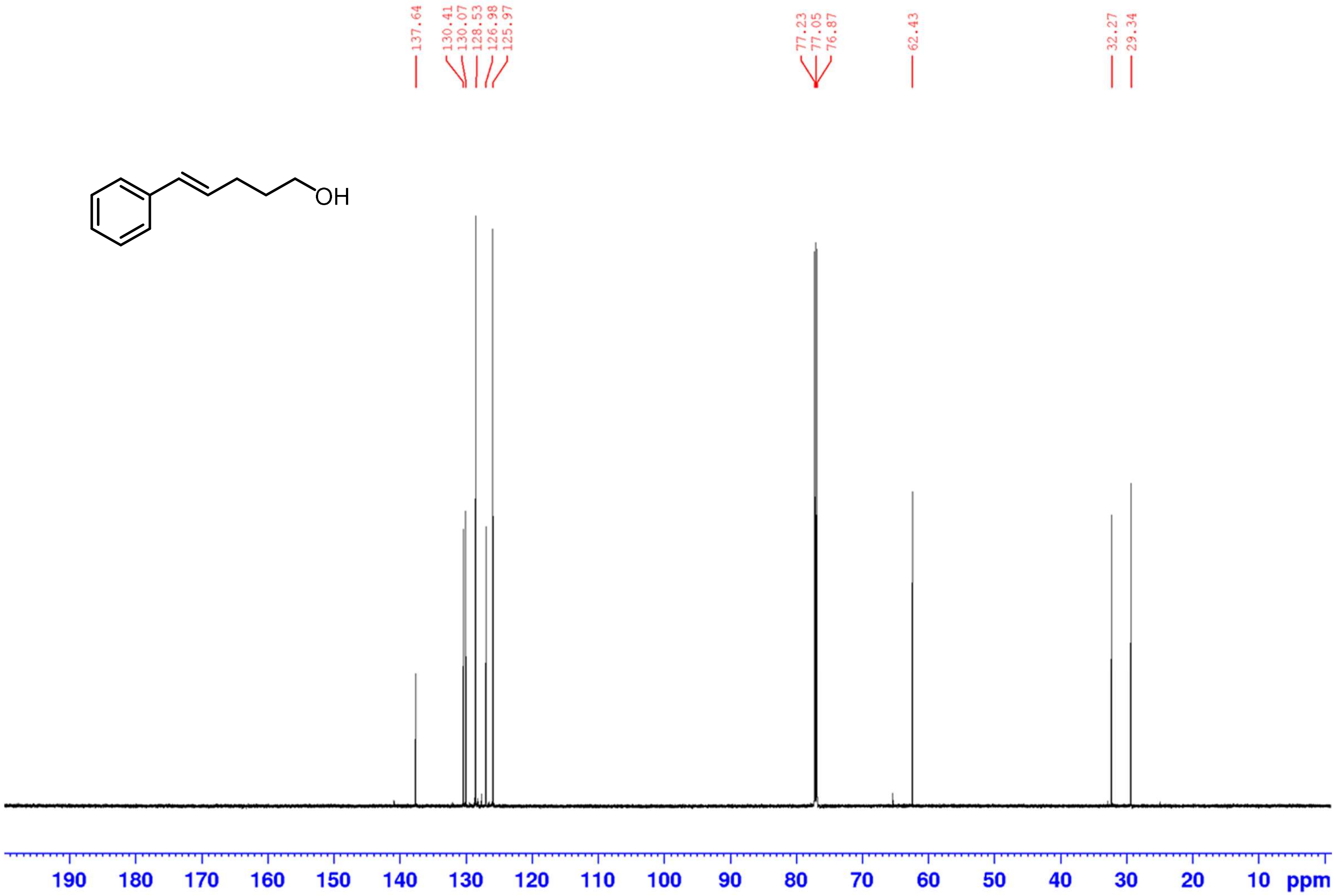

^{1}H NMR (700 MHz, $CDCl_3$) for ***(E)-5-(2-isopropylphenyl)pent-4-en-1-ol (5b)***

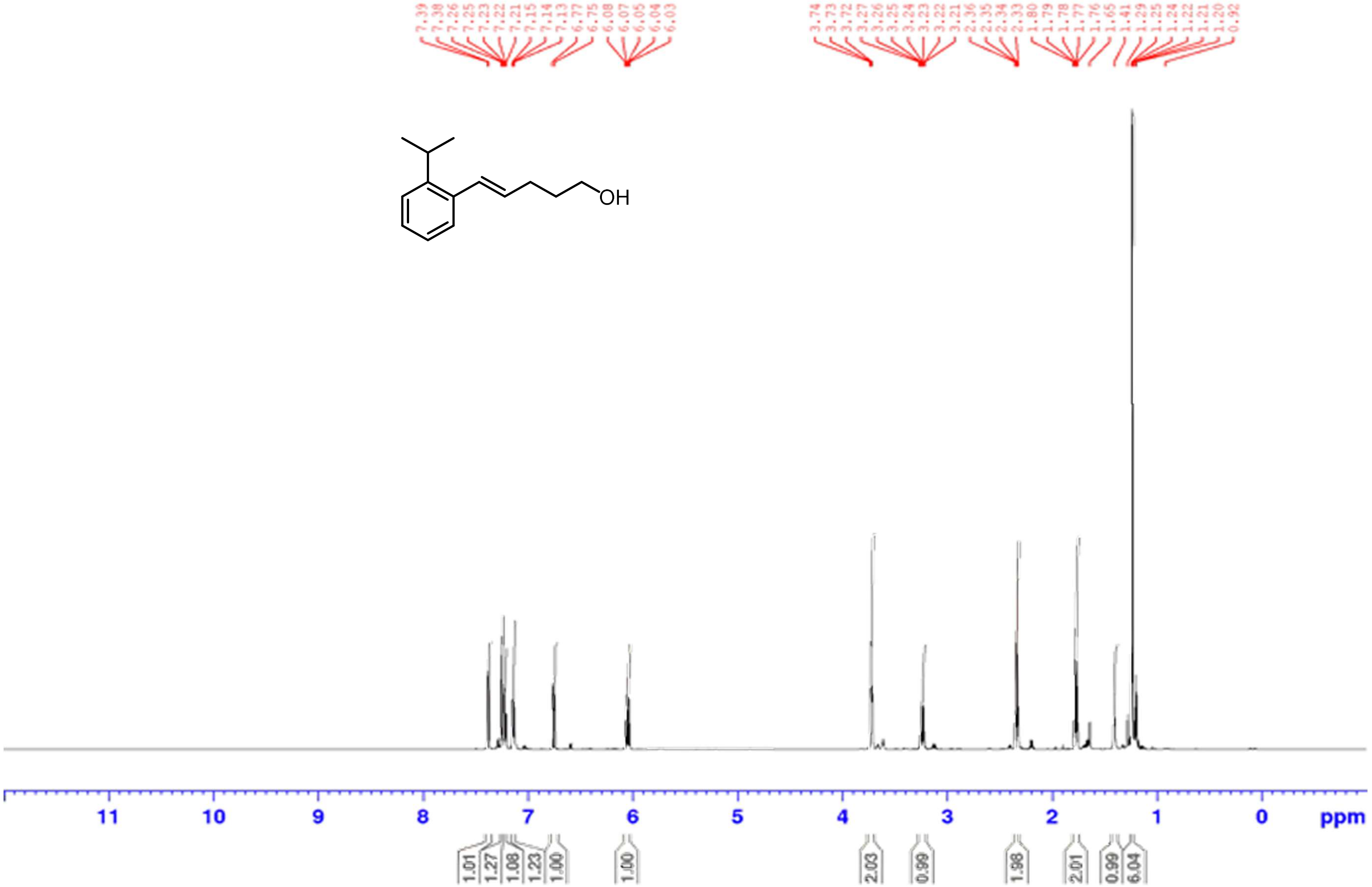

^{13}C NMR (176 MHz, $CDCl_3$) for ***(E)-5-(2-isopropylphenyl)pent-4-en-1-ol (5b)***

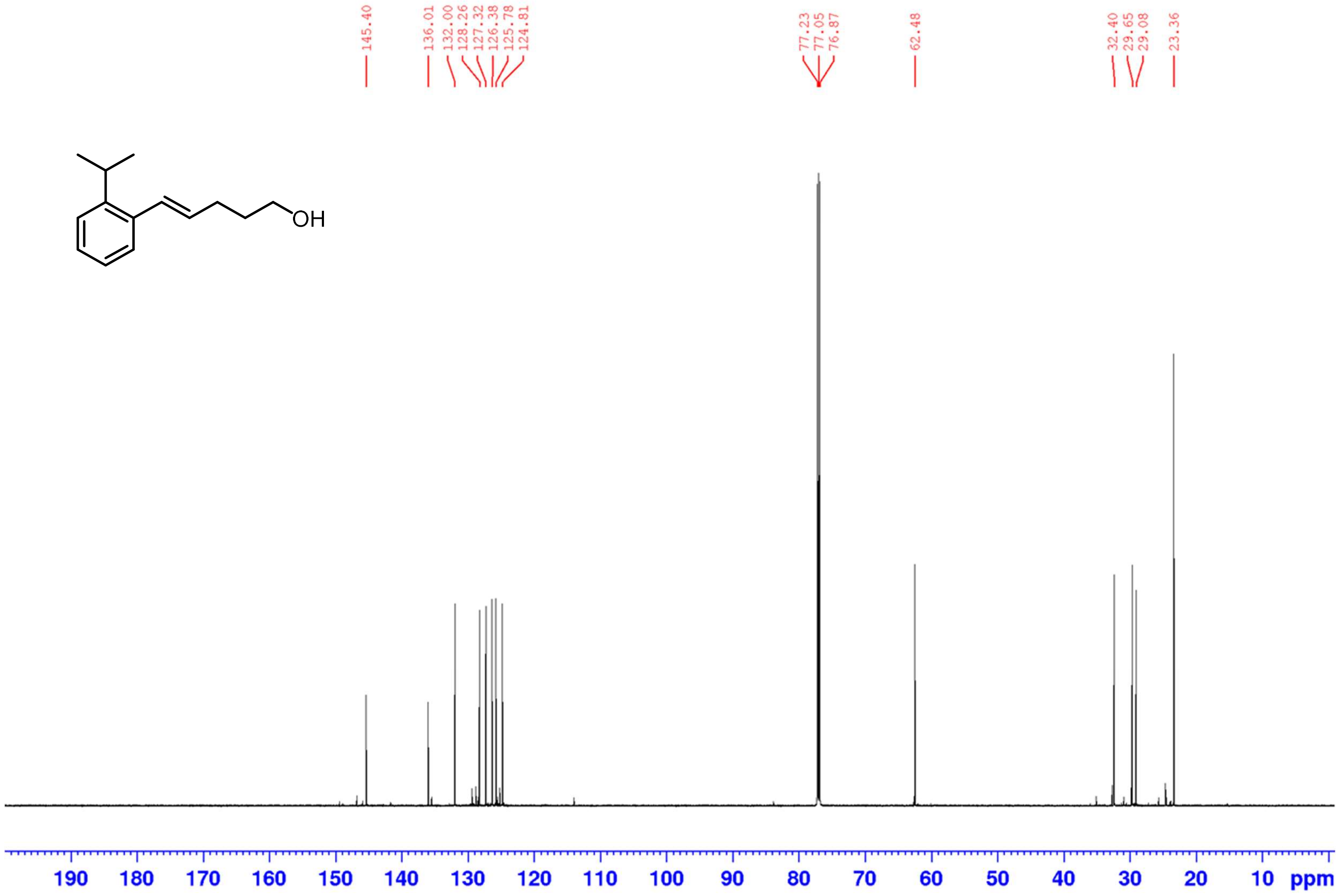

^{1}H NMR (700 MHz, $CDCl_3$) for ***(E)-5-(3-methoxyphenyl)pent-4-en-1-ol (5c)***

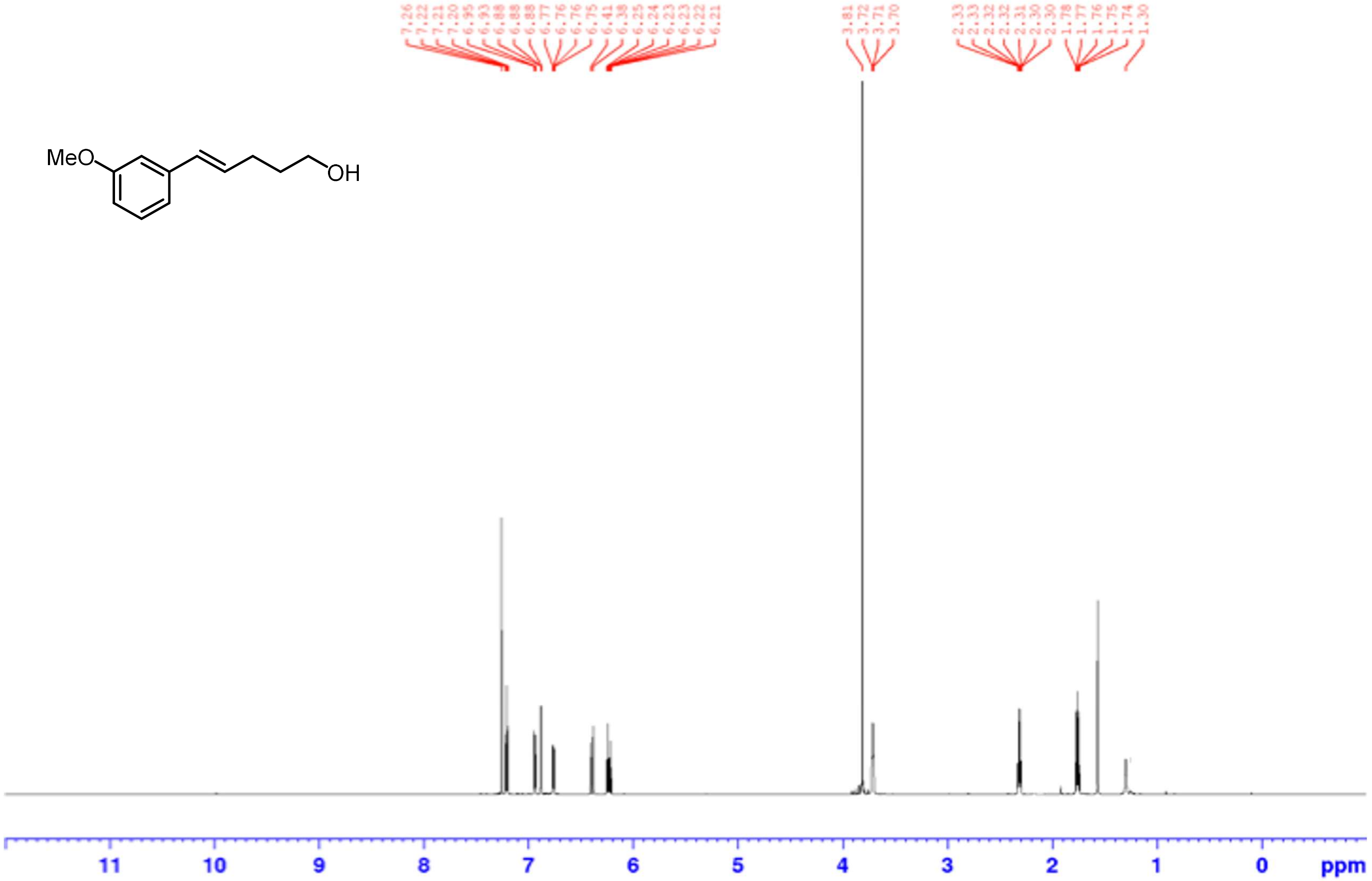

^{13}C NMR (176 MHz, $CDCl_3$) for ***(E)-5-(3-methoxyphenyl)pent-4-en-1-ol (5c)***

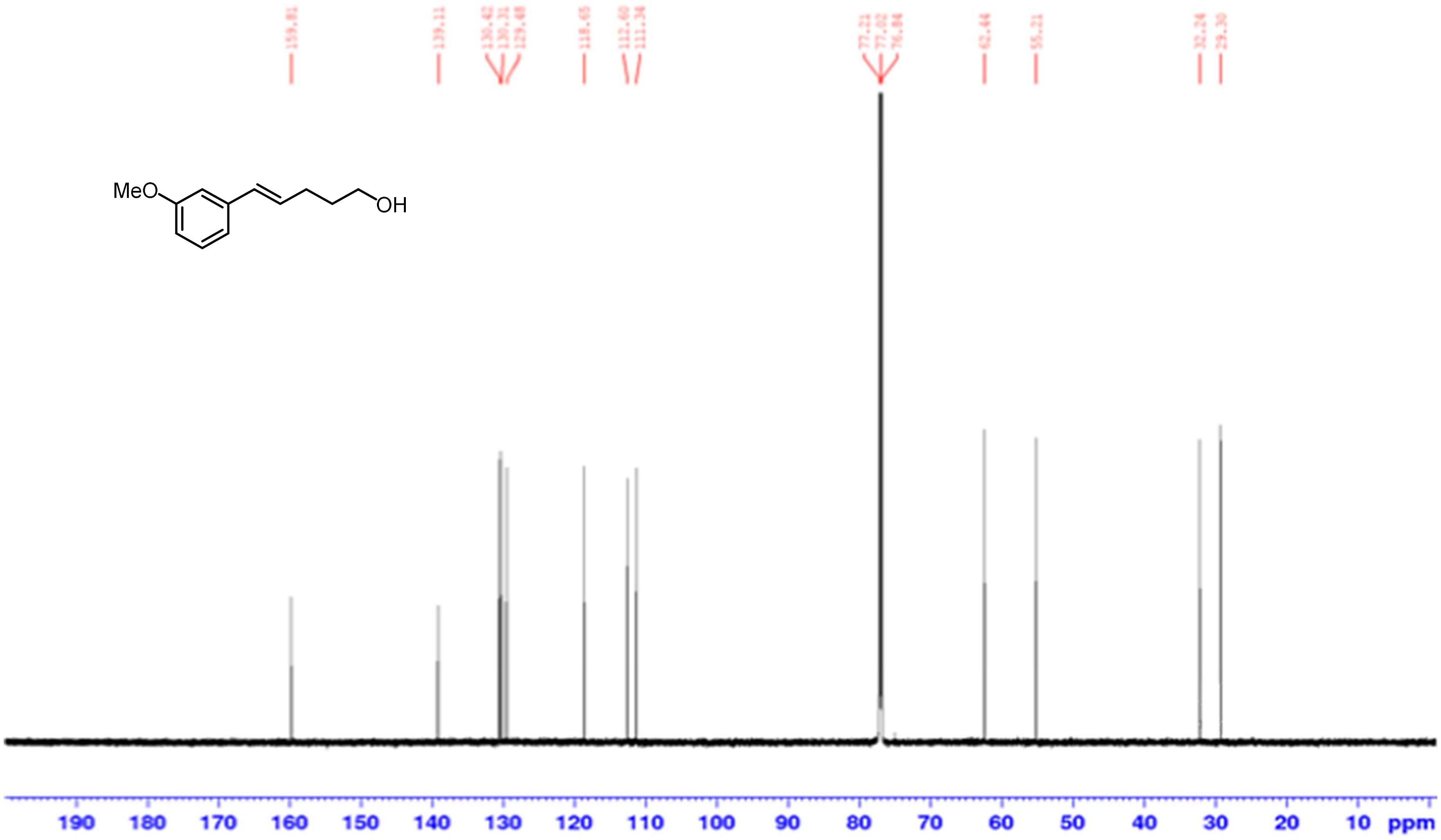

^{1}H NMR (700 MHz, $CDCl_3$) for ***(E)-5-(3,5-di-tert-butylphenyl)pent-4-en-1-ol (5d)***

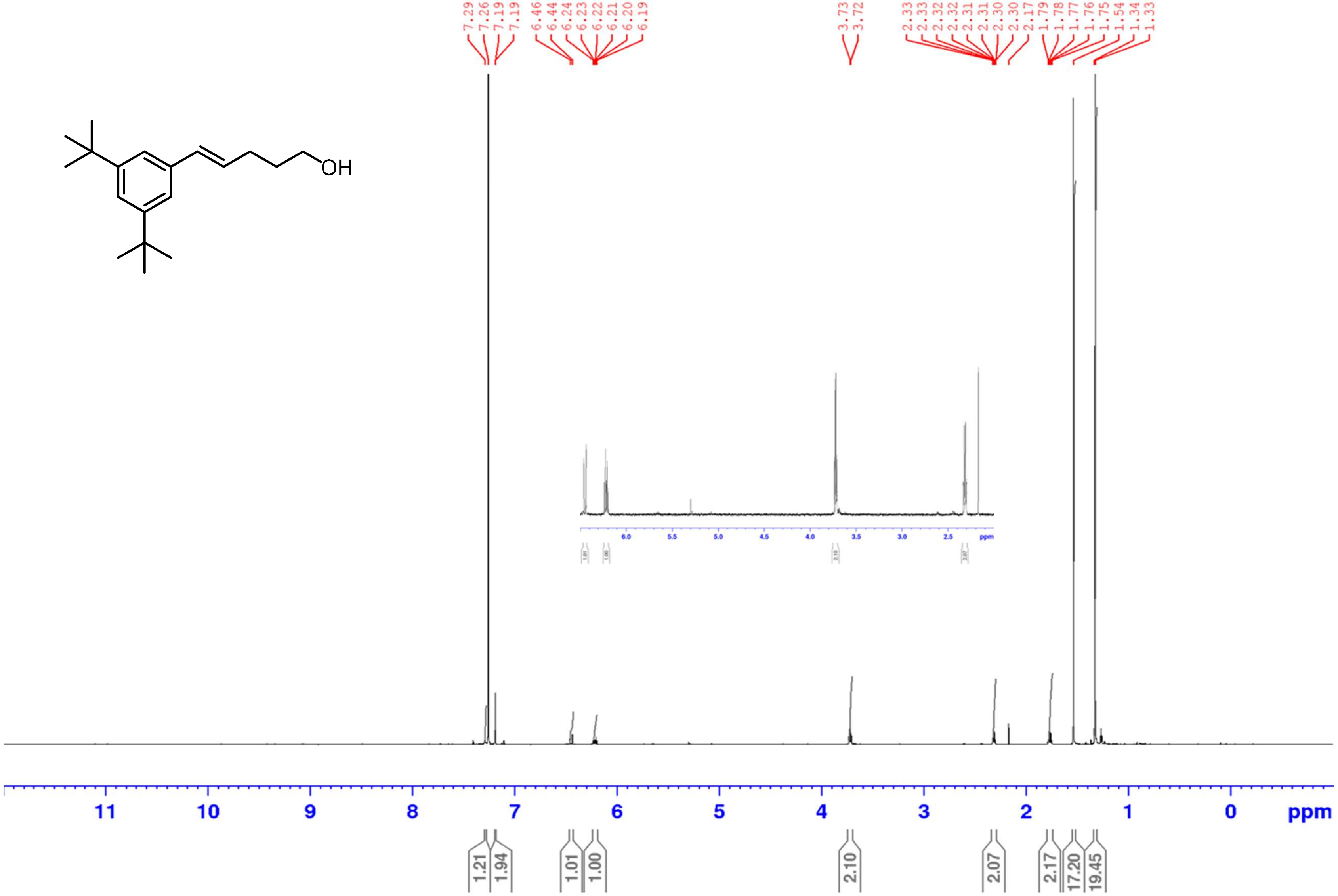

^{13}C NMR (176 MHz, $CDCl_3$) for ***(E)-5-(3,5-di-tert-butylphenyl)pent-4-en-1-ol (5d)***

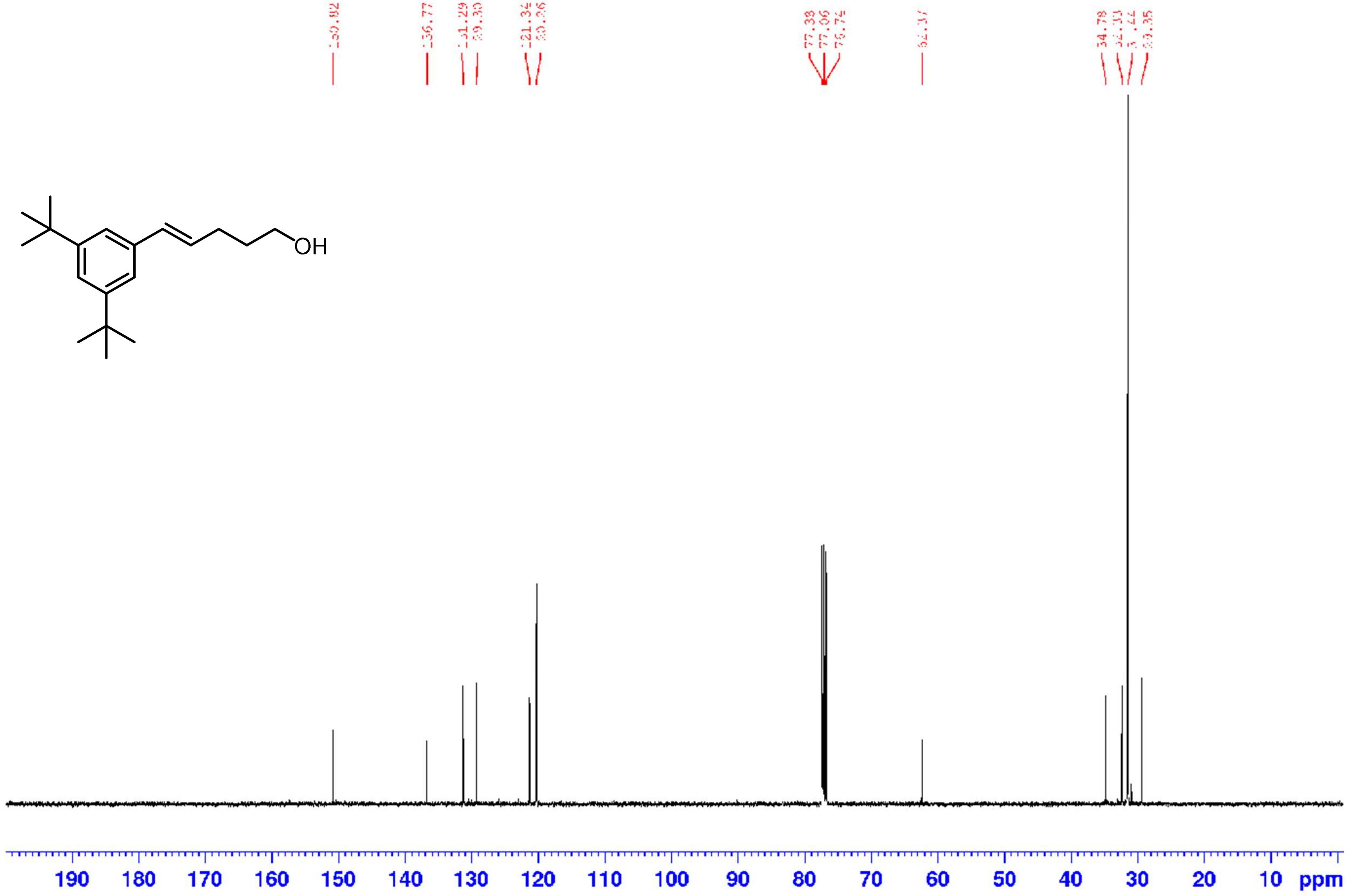

^{1}H NMR (700 MHz, $CDCl_3$) for ***Methyl (E)-2-(5-hydroxypent-1-en-1-yl)benzoate (5e)***

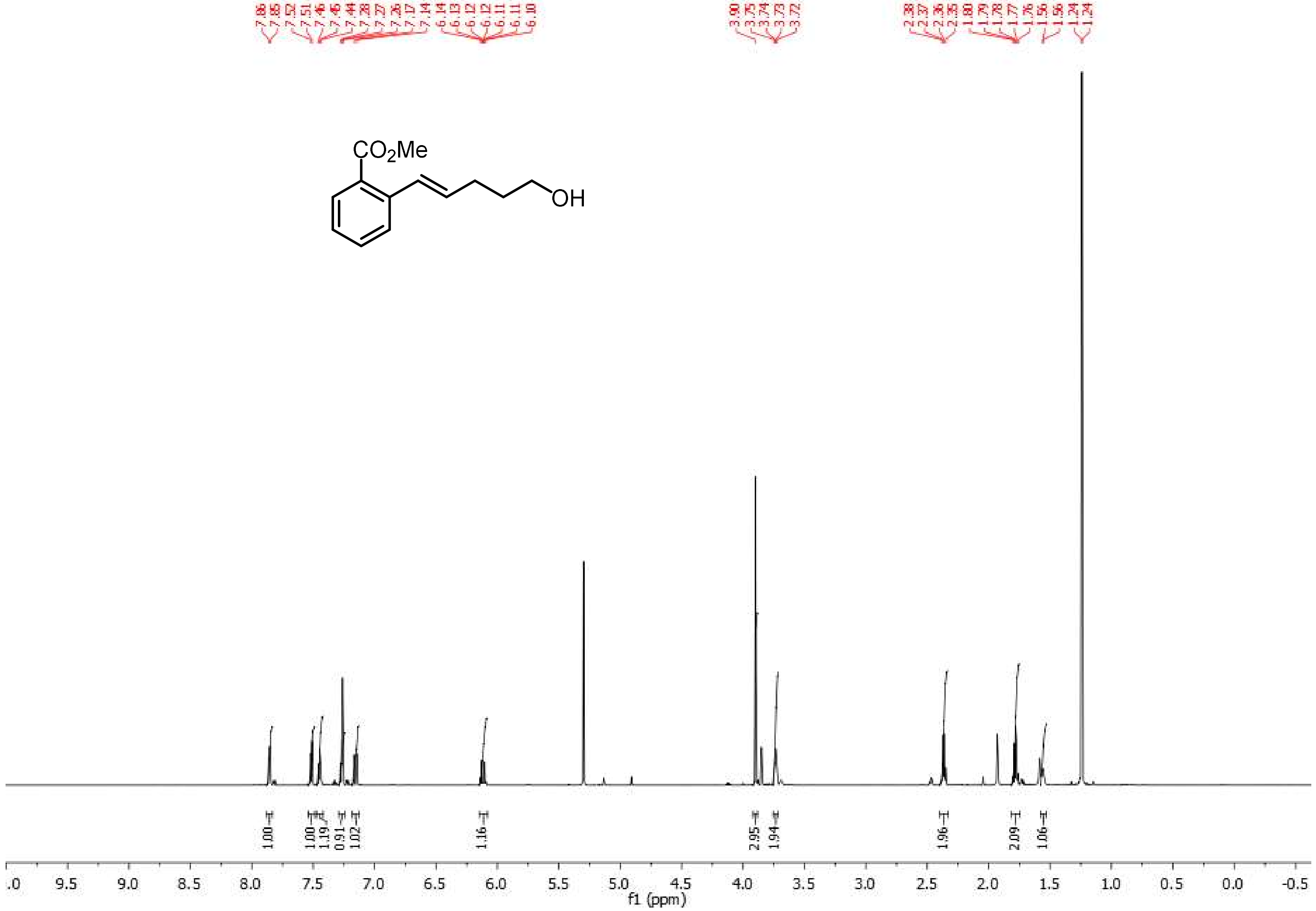

^{13}**C NMR** (176 MHz, $CDCl_3$) for ***methyl (E)-2-(5-hydroxypent-1-en-1-yl)benzoate (5e)***

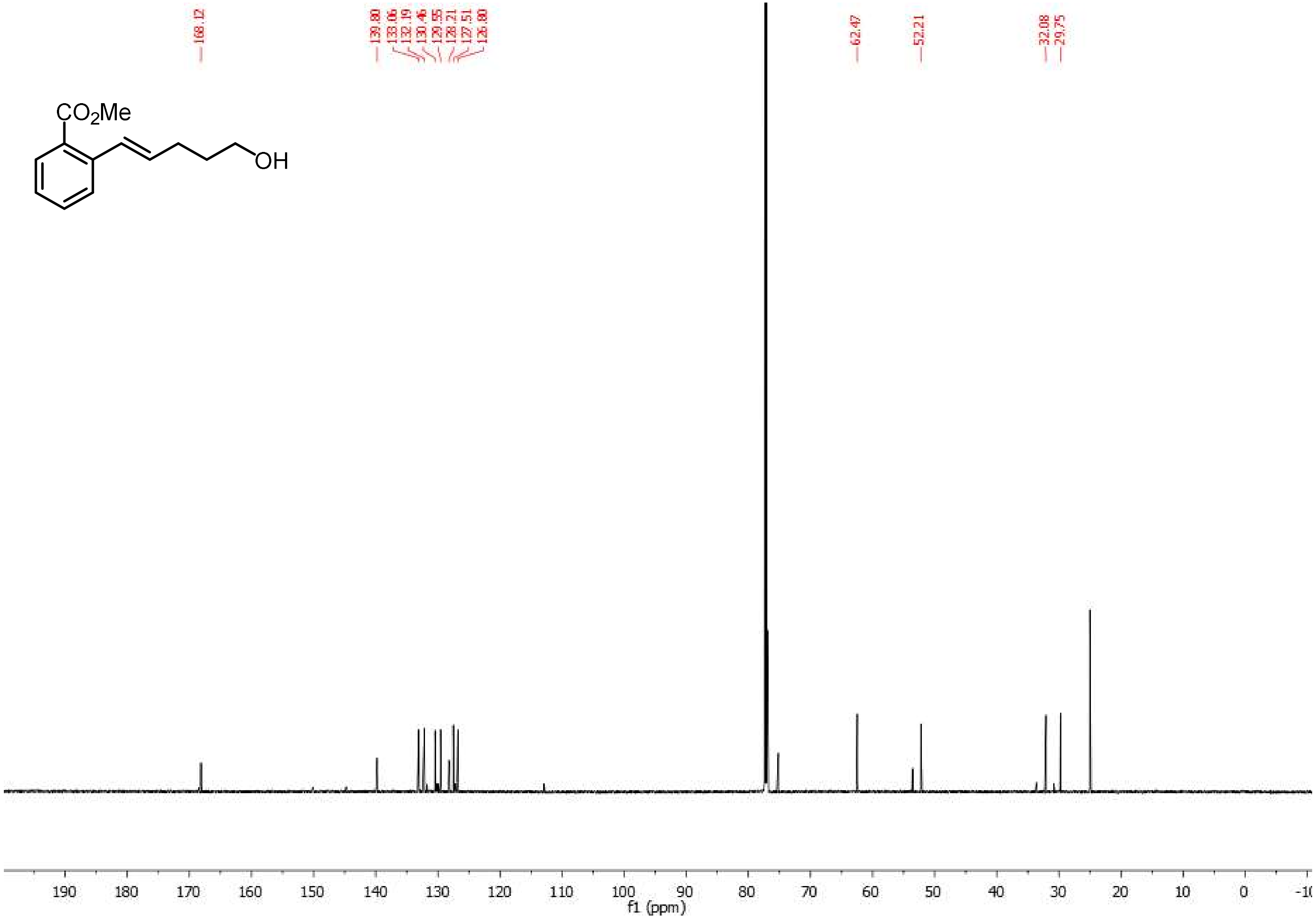

^{1}H NMR (700 MHz, $CDCl_3$) for ***(E)-2-methyl-6-phenylhex-5-en-2-ol (5f)***

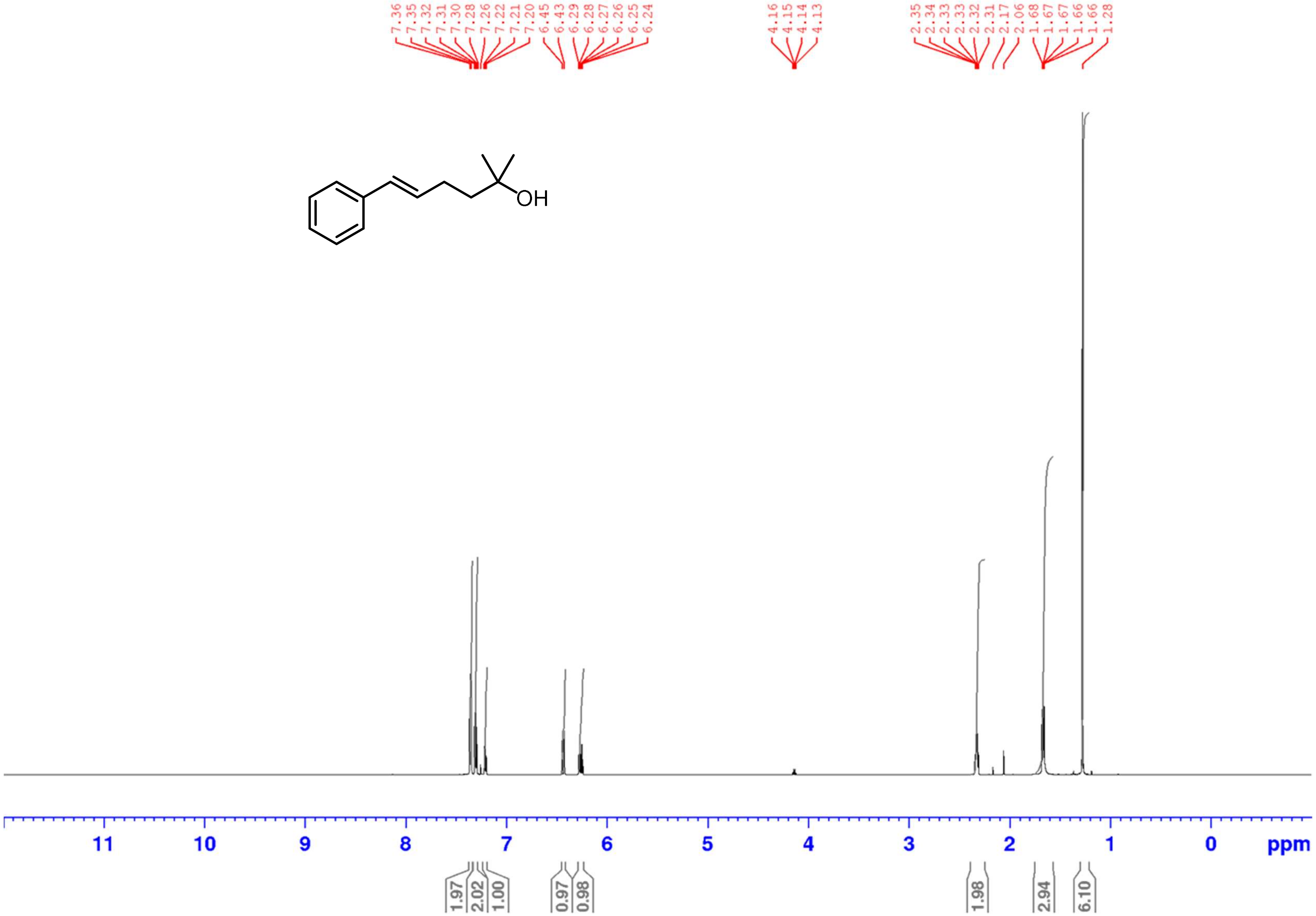

^{13}C NMR (176 MHz, $CDCl_3$) for ***(E)-2-methyl-6-phenylhex-5-en-2-ol (5f)***

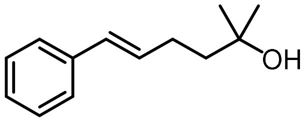

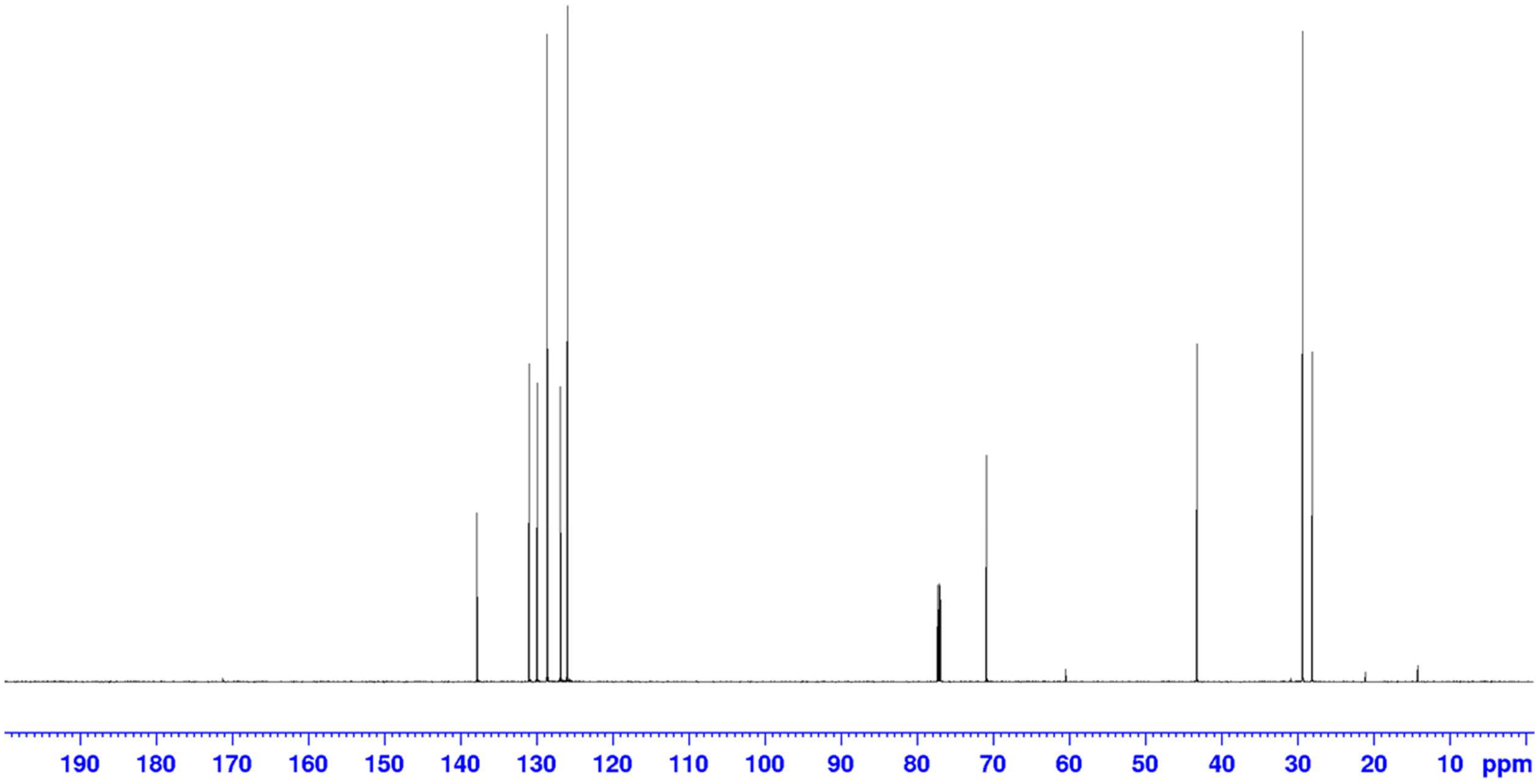

^{1}H NMR (700 MHz, $CDCl_3$) for ***2,2,2-trichloroethyl (R,E)-(6-hydroxy-1-phenylhex-1-en-3-yl)sulfamate (2a)***

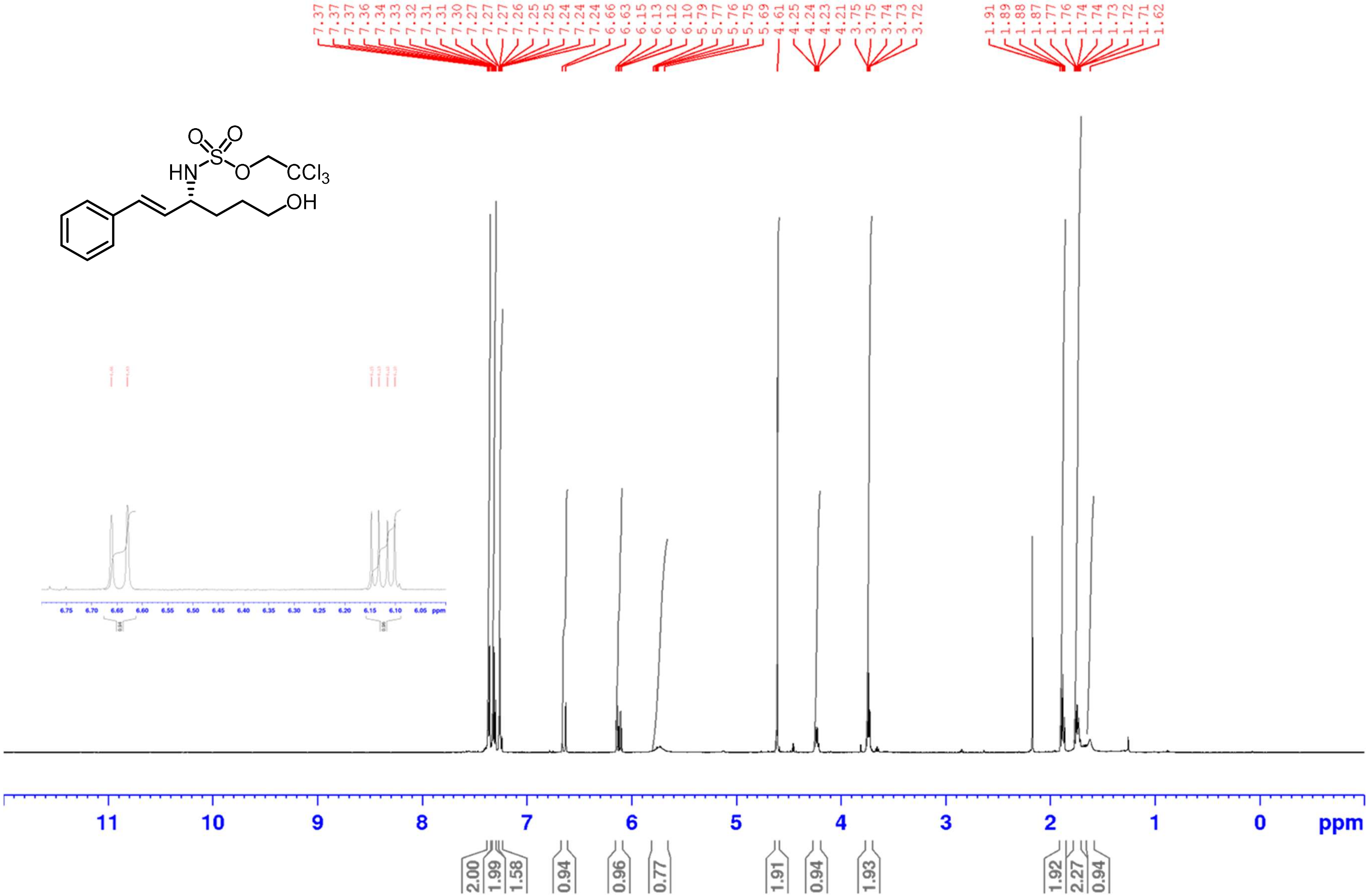

^{13}C NMR (176 MHz, $CDCl_3$) for ***2,2,2-trichloroethyl (R,E)-(6-hydroxy-1-phenylhex-1-en-3-yl)sulfamate (2a)***

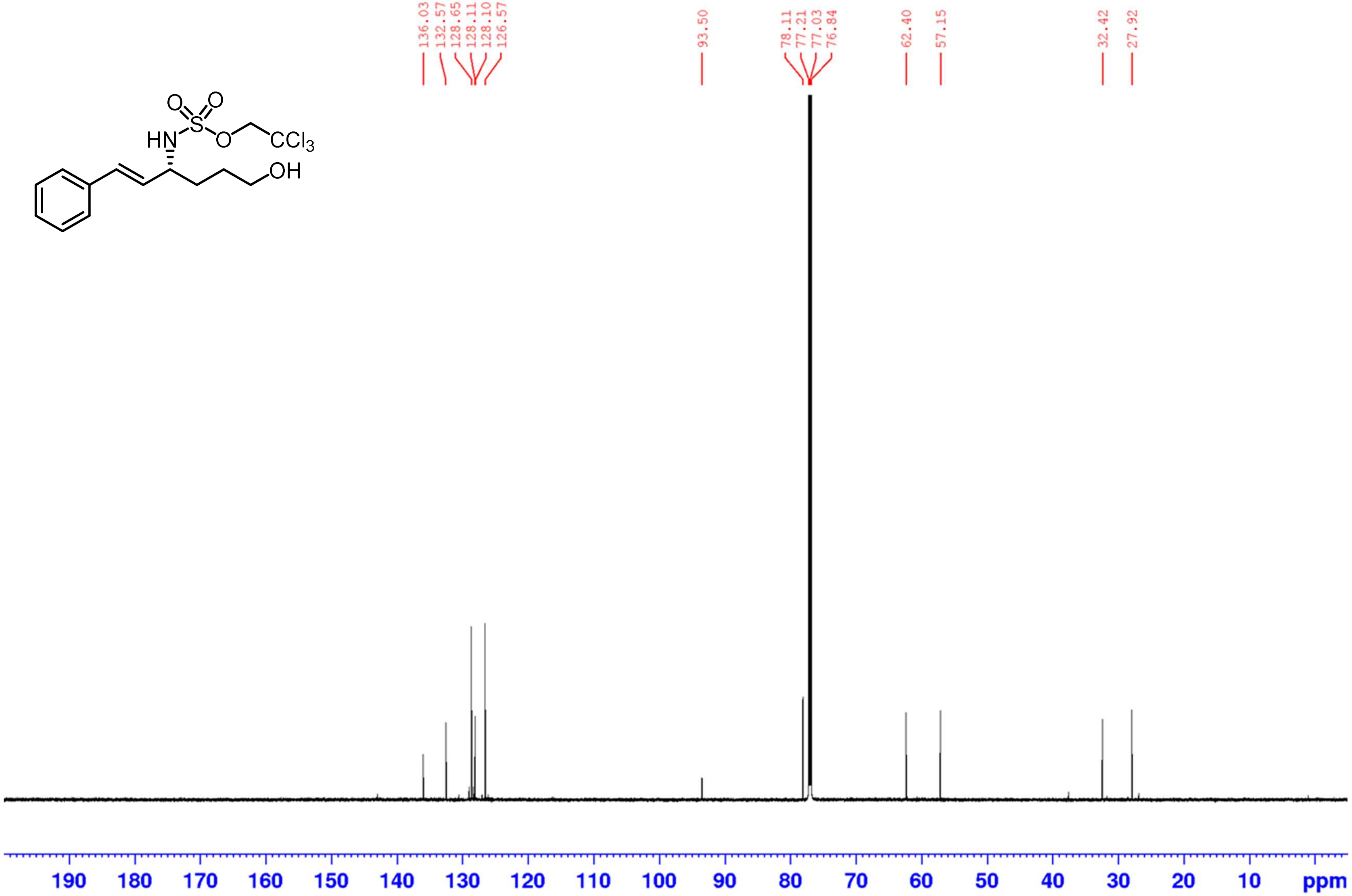

^{1}H NMR (700 MHz, $CDCl_3$) for ***2,2,3,3,3-pentafluoropropyl (R,E)-(6-hydroxy-1-phenylhex-1-en-3-yl)sulfamate (2a-pfps)***

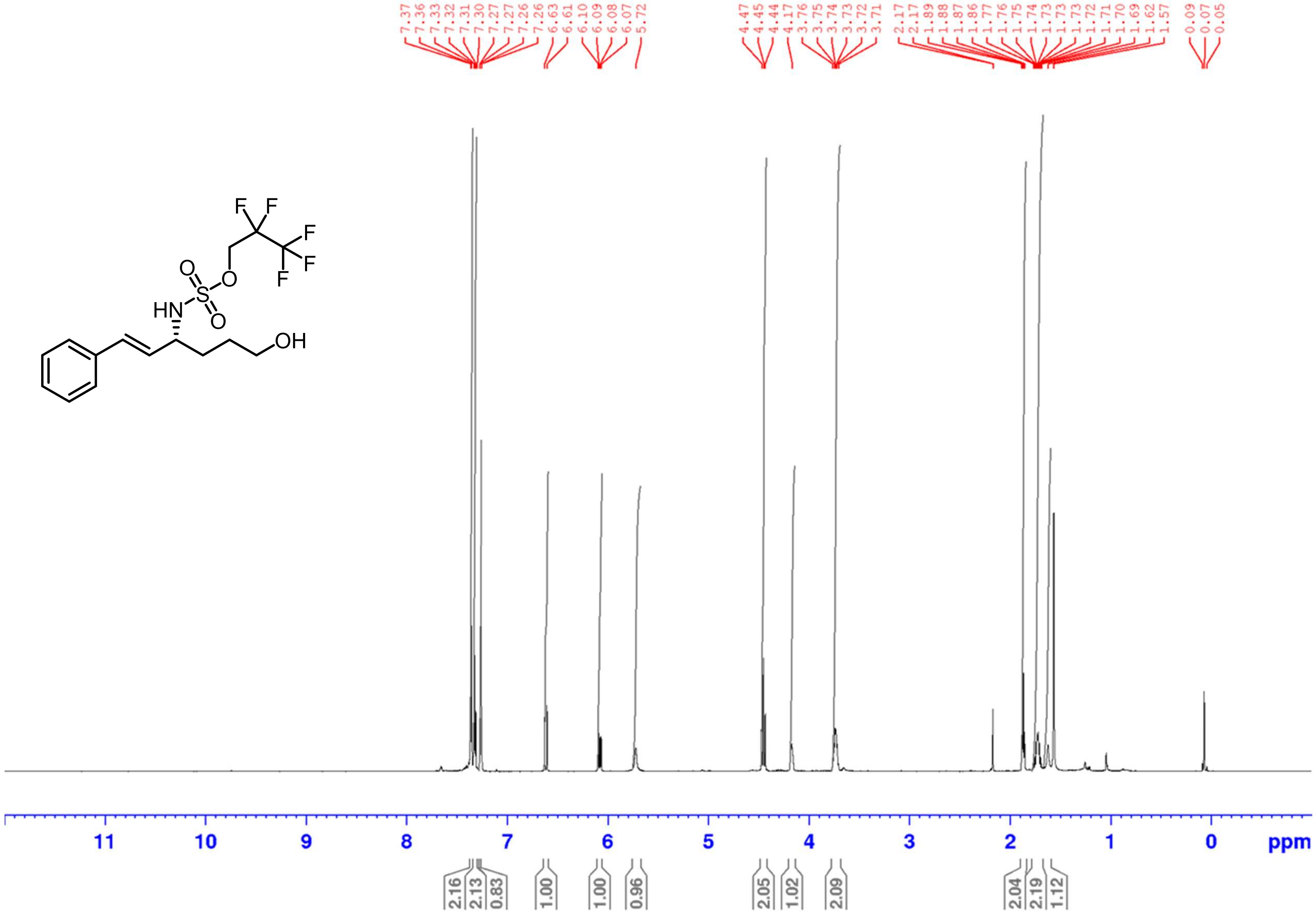

^{13}C NMR (176 MHz, $CDCl_3$) for ***2,2,3,3,3-pentafluoropropyl (R,E)-(6-hydroxy-1-phenylhex-1-en-3-yl)sulfamate(2a-pfps)***

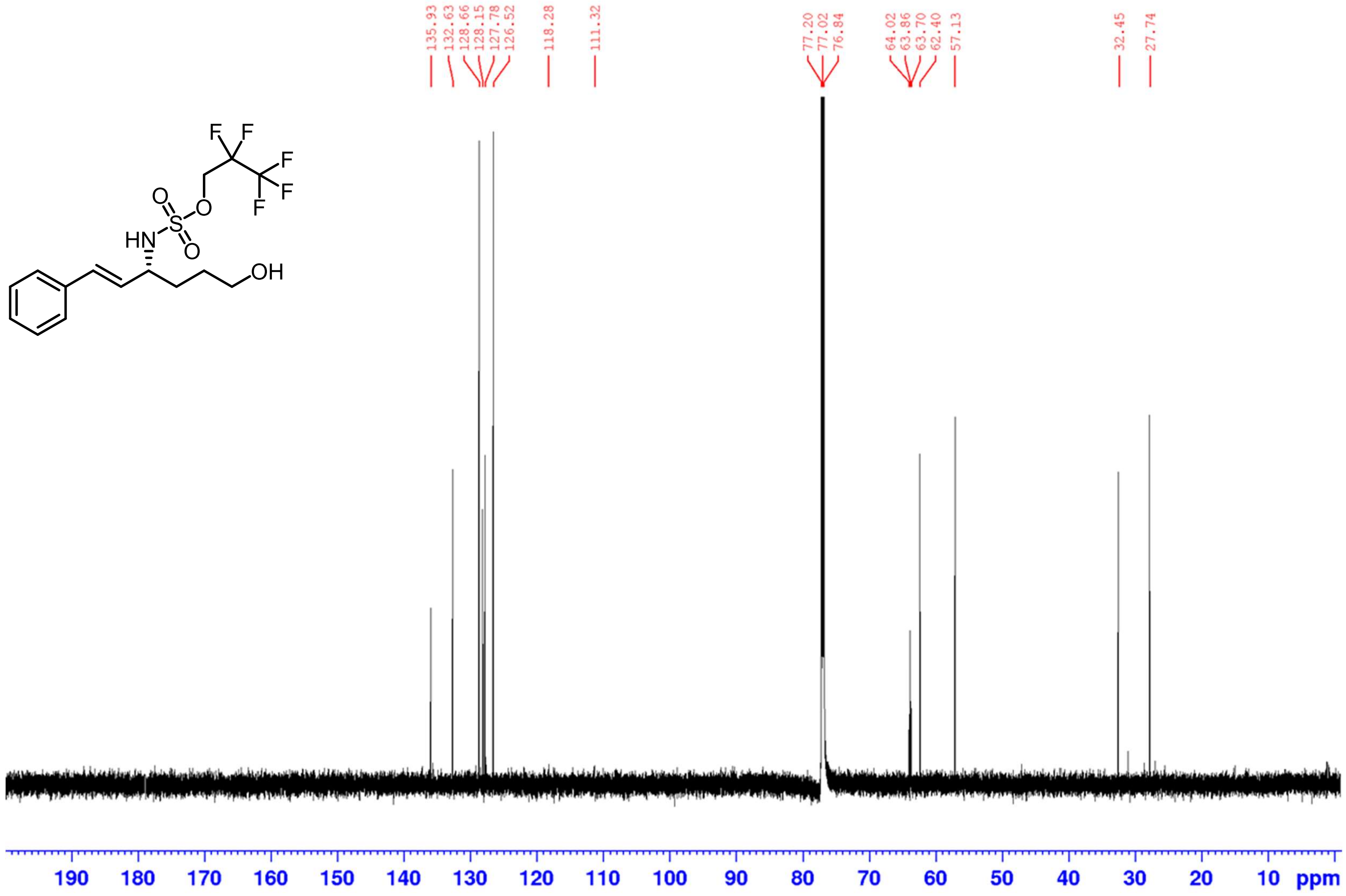

^{19}F NMR (376 MHz, $CDCl_3$) for ***2,2,3,3,3-pentafluoropropyl (R,E)-(6-hydroxy-1-phenylhex-1-en-3-yl)sulfamate (2a-pfps)***

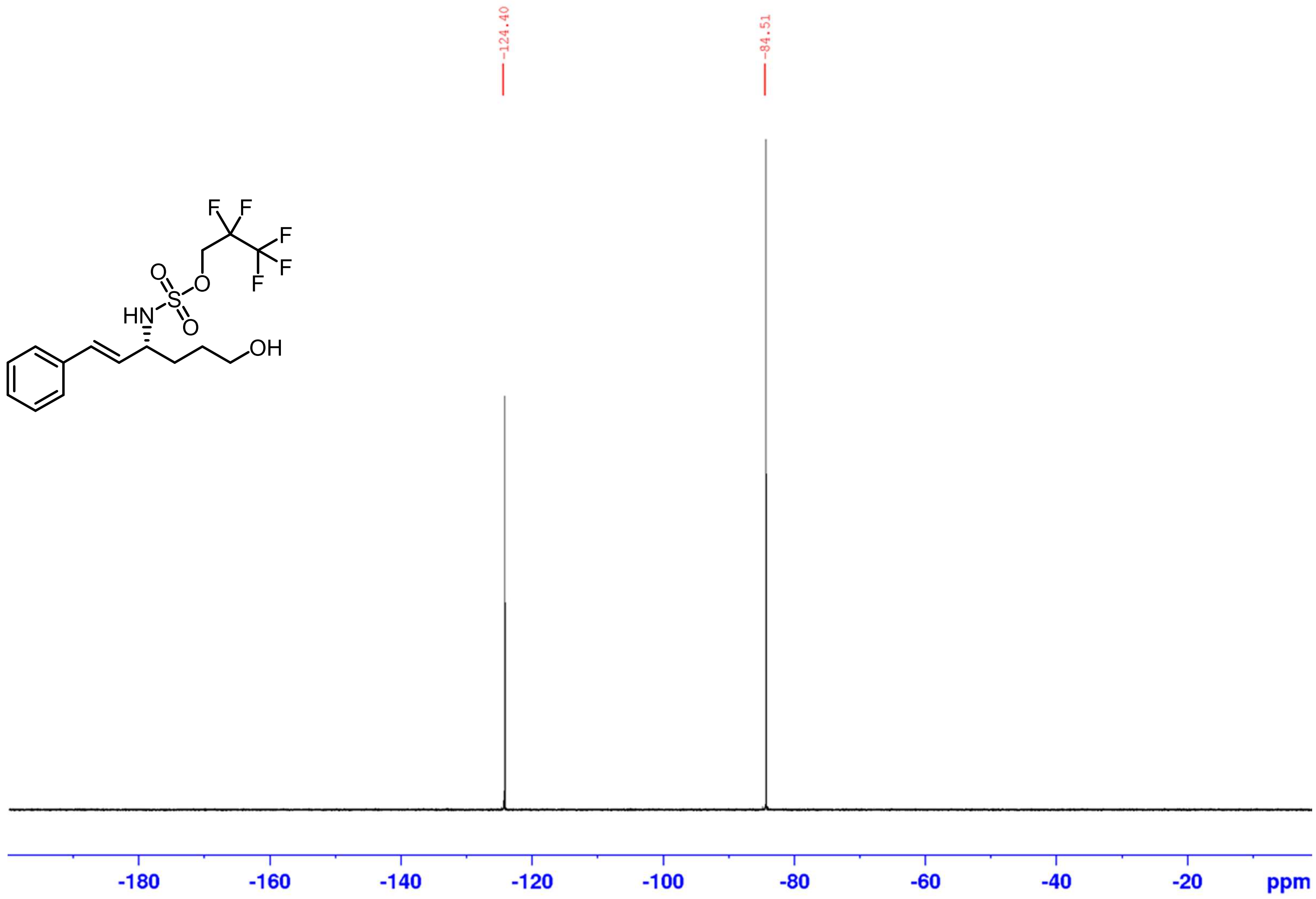

^{1}H NMR (700 MHz, $CDCl_3$) for ***2,2,2-trichloroethyl 2-(4-hydroxybutyl)-(E)-3-phenylaziridine-1-sulfonate (3a)***

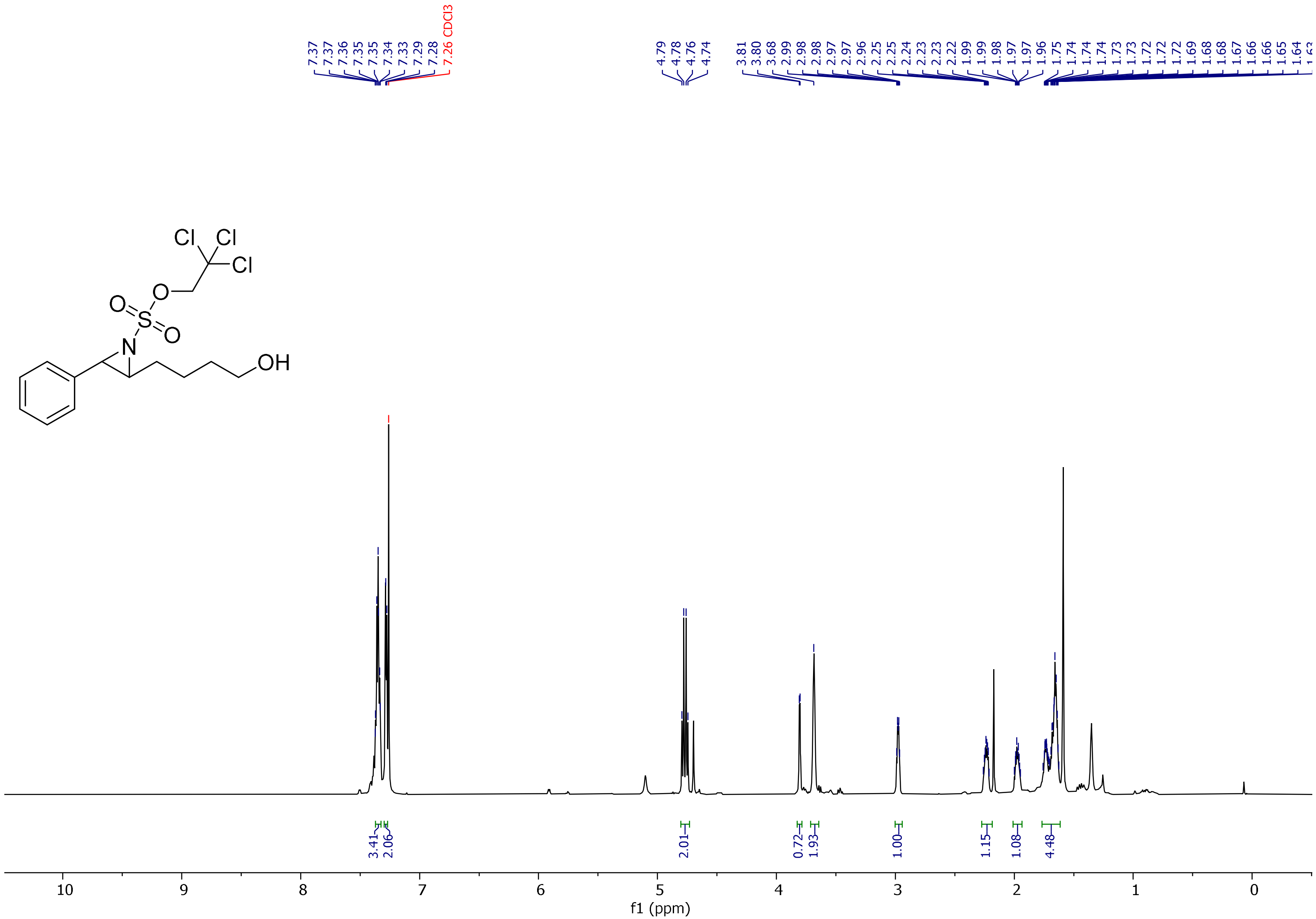

^{13}C NMR (101 MHz, $CDCl_3$) for ***2,2,2-trichloroethyl 2-(4-hydroxybutyl)-(E)-3-phenylaziridine-1-sulfonate (3a)***

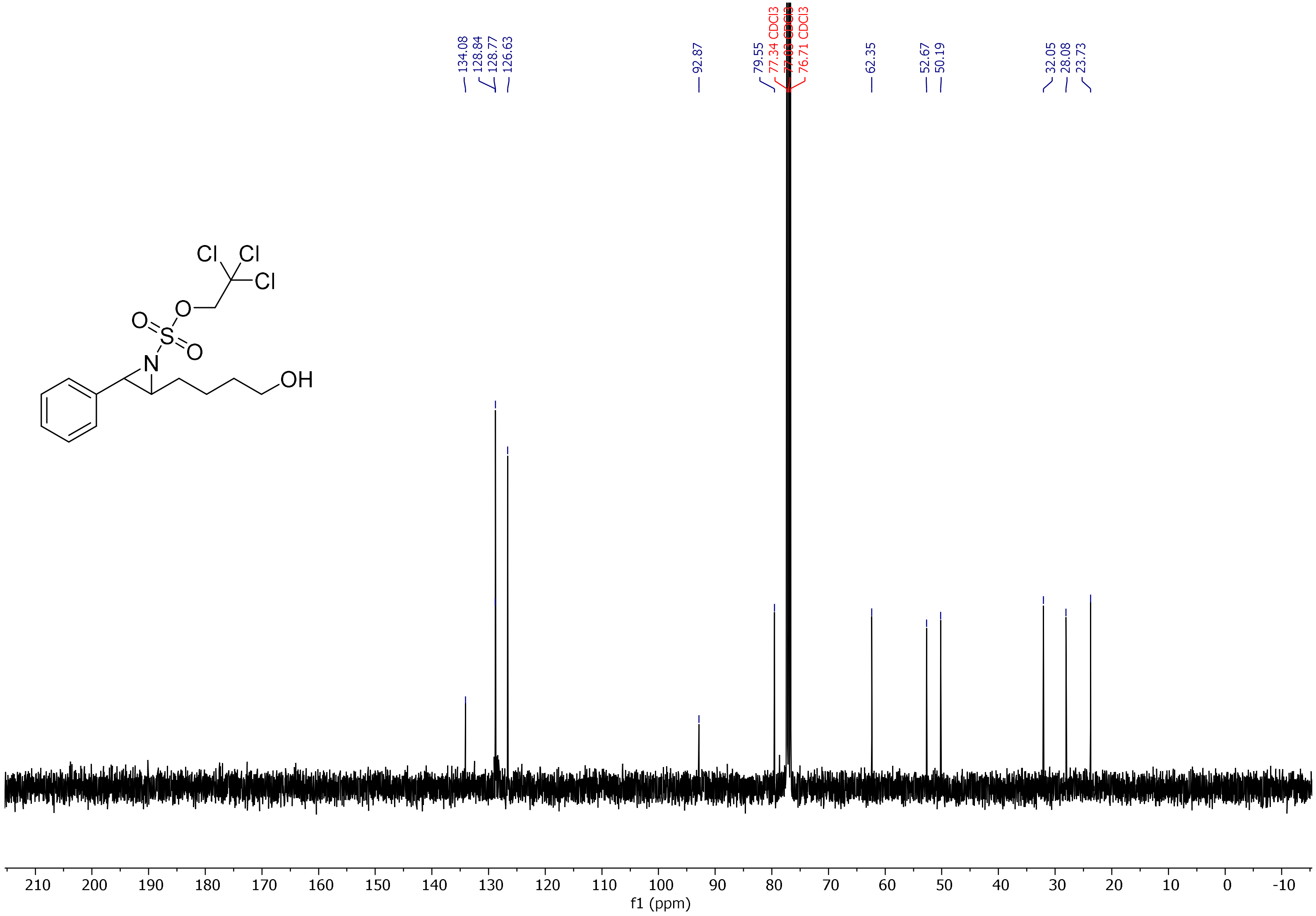

^1H NMR (700 MHz, $CDCl_3$) for ***2,2,2-trichloroethyl (Z)-(6-hydroxy-1-phenylhex-1-en-3-yl)sulfamate ((Z)-2a)***

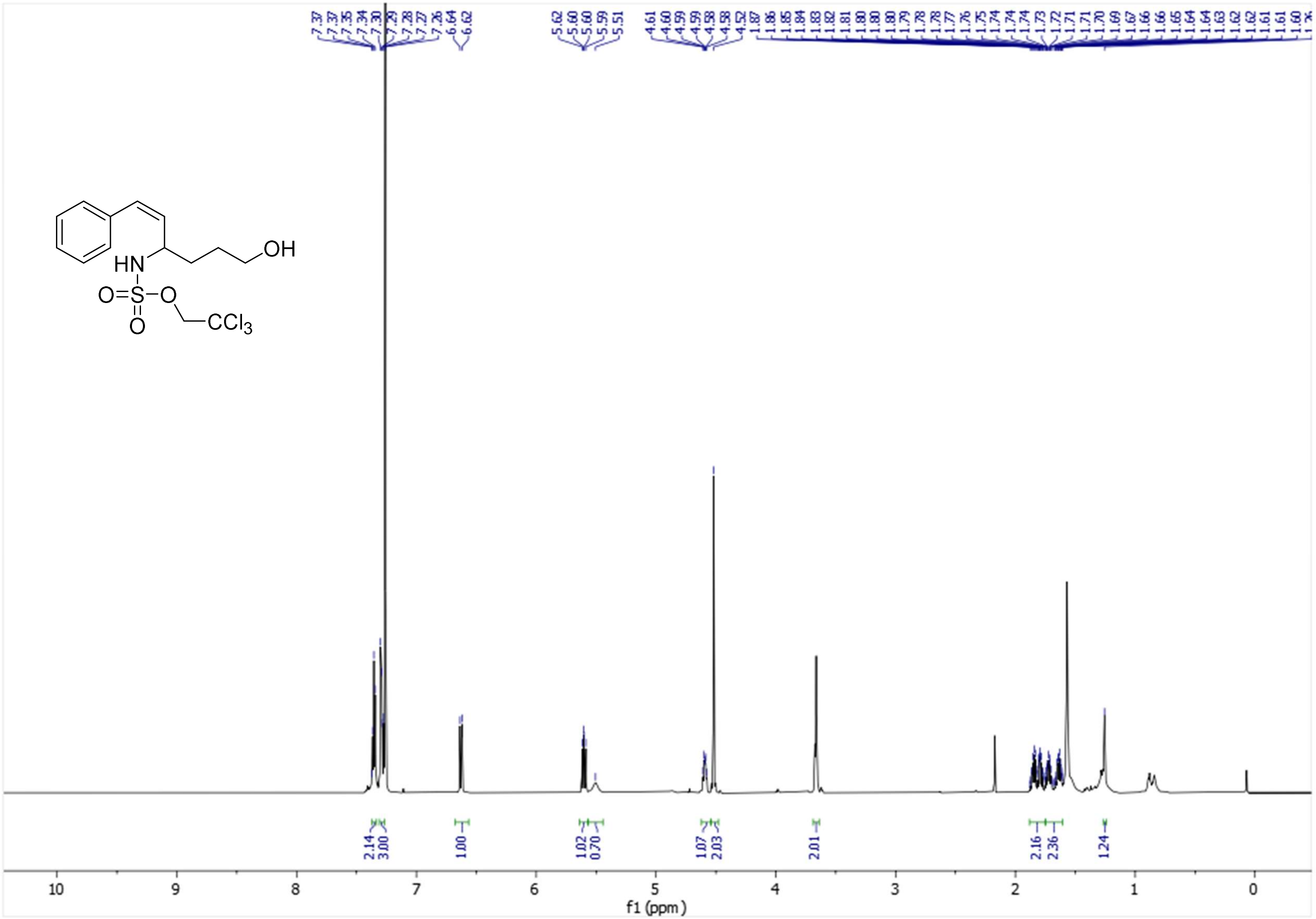

^{13}C NMR (176 MHz, $CDCl_3$) for ***2,2,2-trichloroethyl (Z)-(6-hydroxy-1-phenylhex-1-en-3-yl)sulfamate ((Z)-2a)***

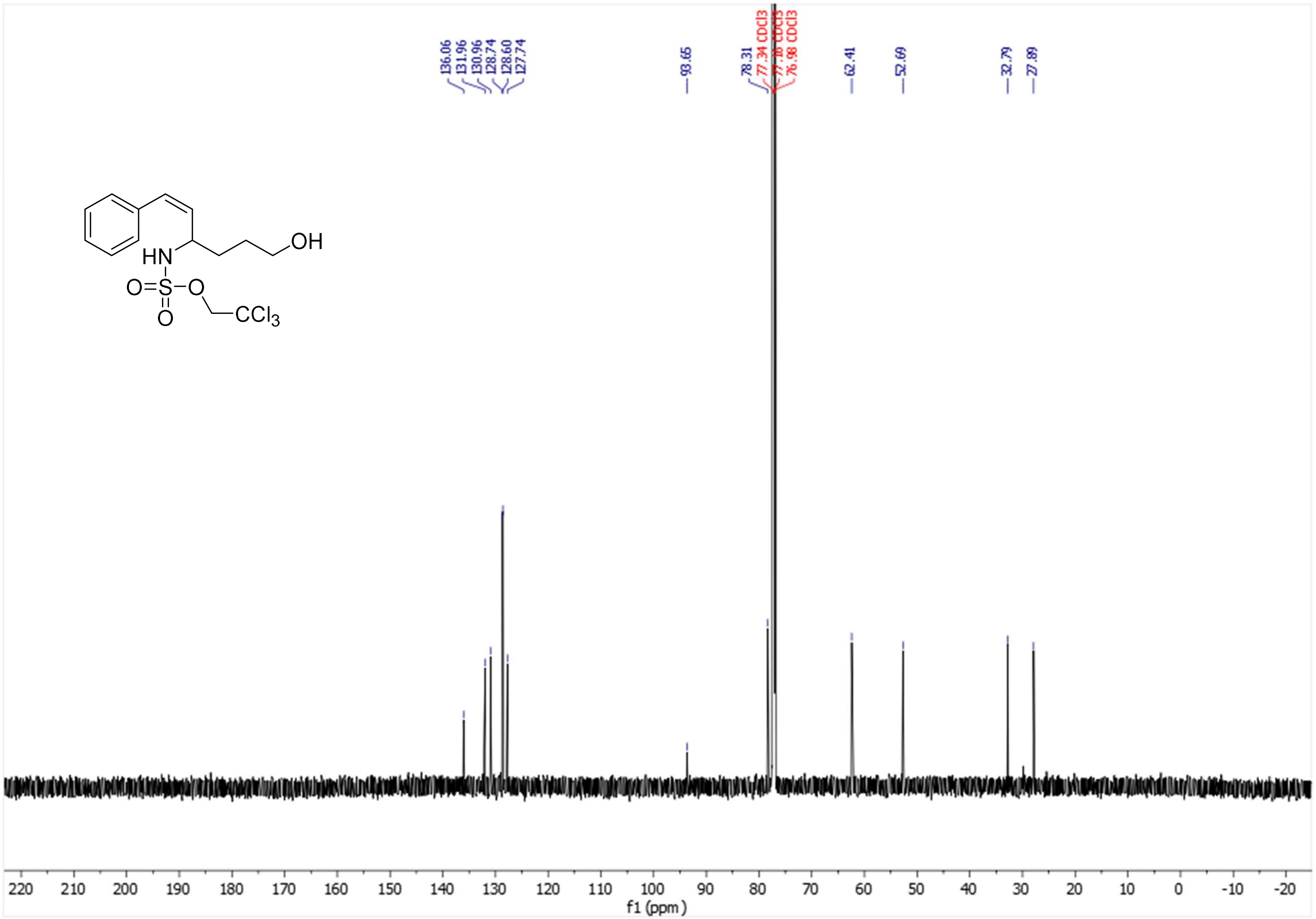

^{1}H NMR (700 MHz, $CDCl_3$) for ***2,2,2-trichloroethyl 2-(4-hydroxybutyl)-(Z)-3-phenylaziridine-1-sulfonate ((Z)-3a)***

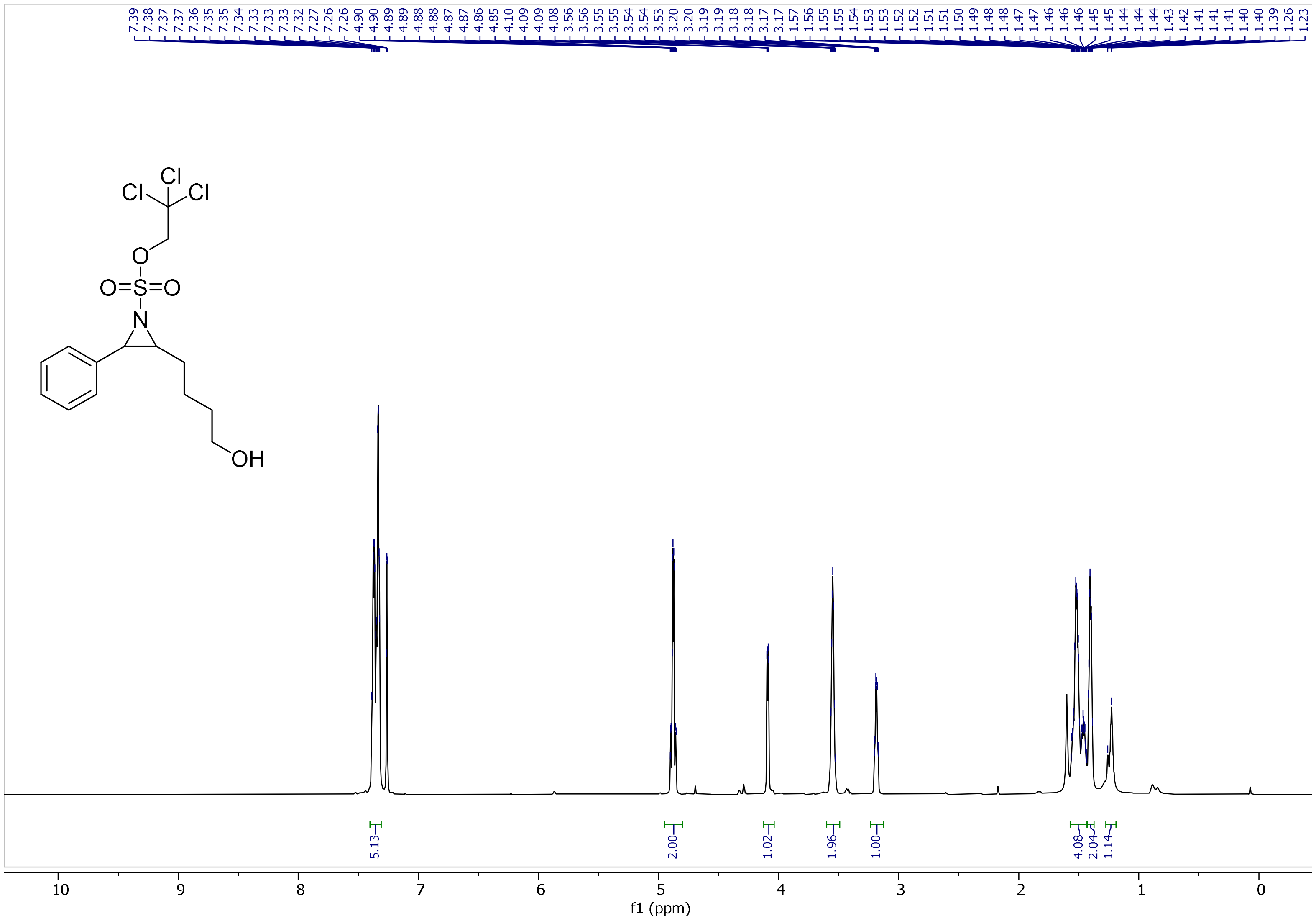

^{13}C NMR (176 MHz, $CDCl_3$) for ***2,2,2-trichloroethyl 2-(4-hydroxybutyl)-(Z)-3-phenylaziridine-1-sulfonate ((Z)-3a)***

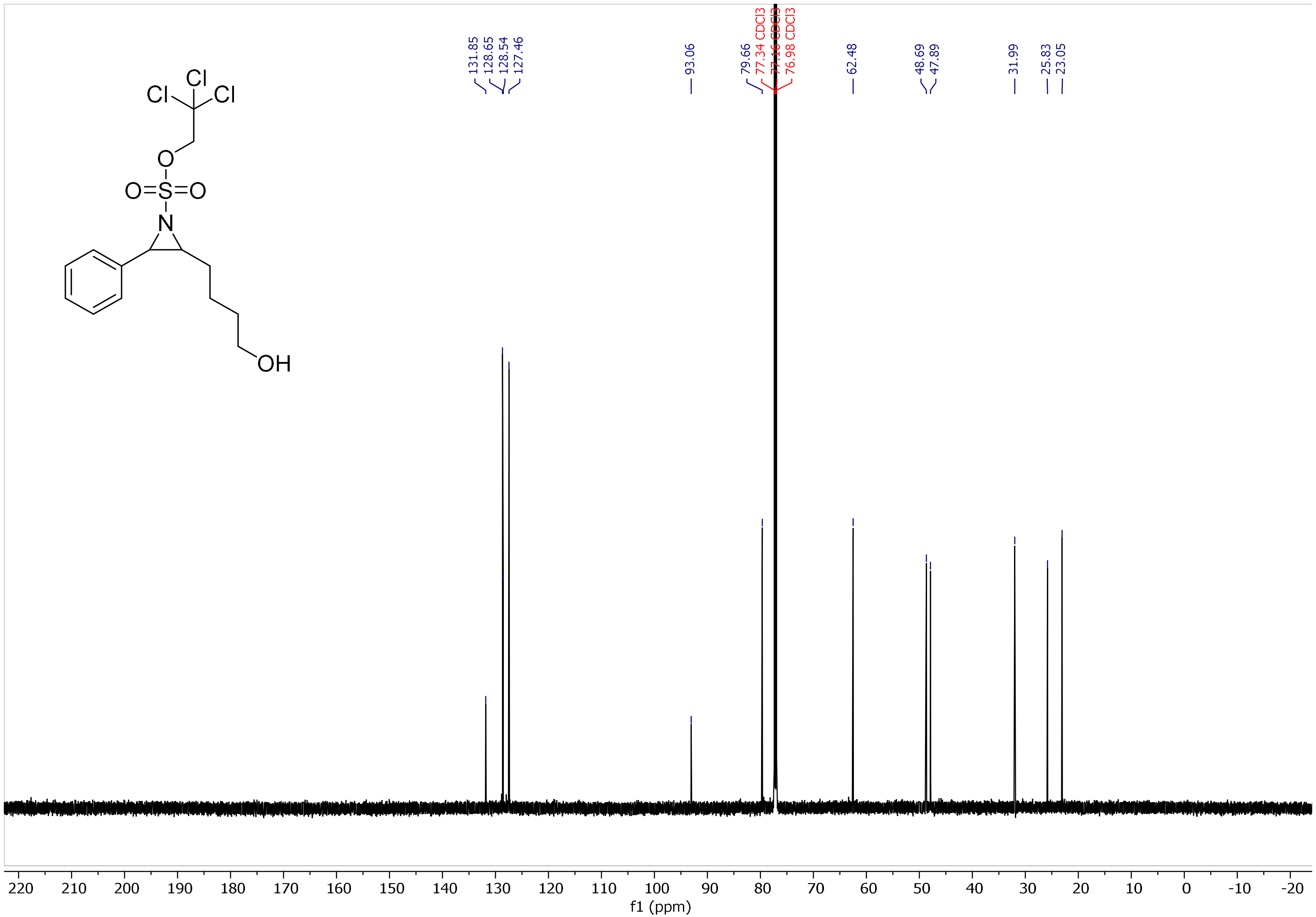

^{1}H NMR (700 MHz, $CDCl_3$) for ***2,2,3,3,3-pentafluoropropyl (R,E)-(1-(3-acetylphenyl)-6-hydroxyhex-1-en-3-yl)sulfamate (2b-pfps)***

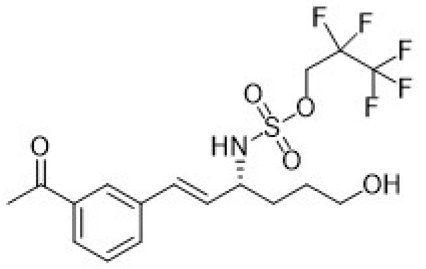

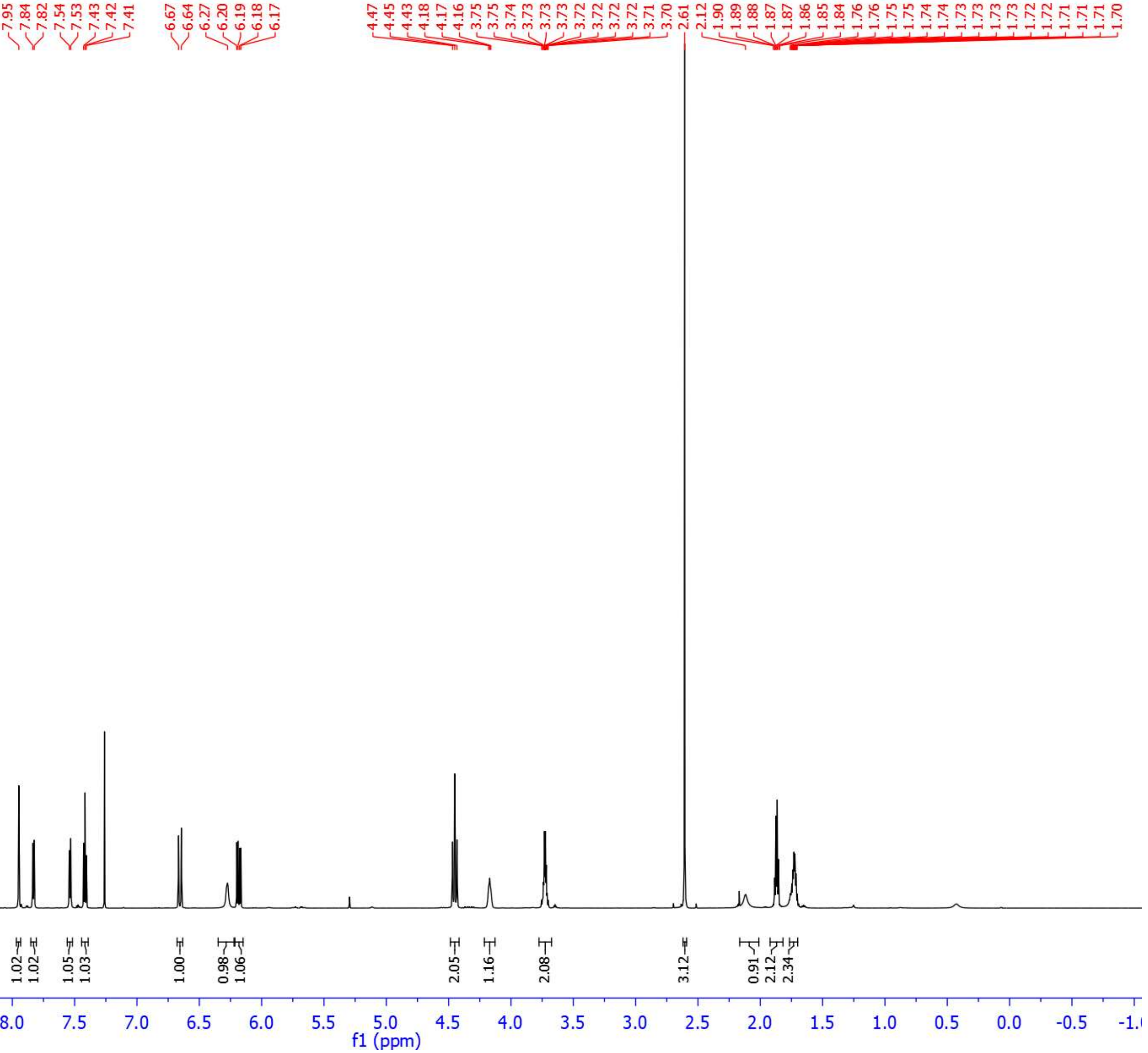

^{13}C NMR (176 MHz, CDCl$_3$) for *2,2,3,3,3-pentafluoropropyl (R,E)-(1-(3-acetylphenyl)-6-hydroxyhex-1-en-3-yl)sulfamate (2b-pfps)*

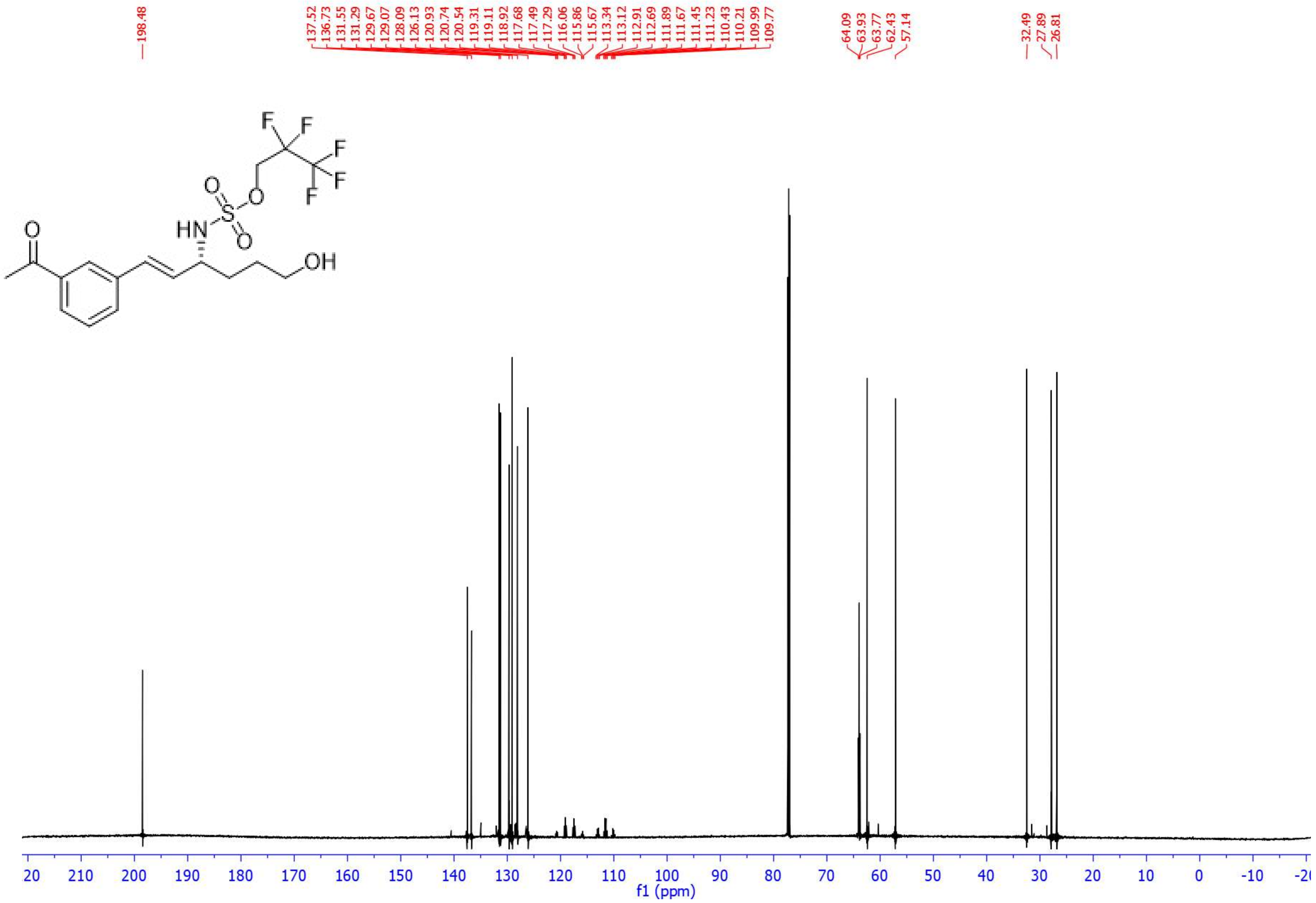

^{19}F NMR (376 MHz, $CDCl_3$) for ***2,2,3,3,3-pentafluoropropyl (R,E)-(1-(3-acetylphenyl)-6-hydroxyhex-1-en-3-yl)sulfamate (2b-pfps):***

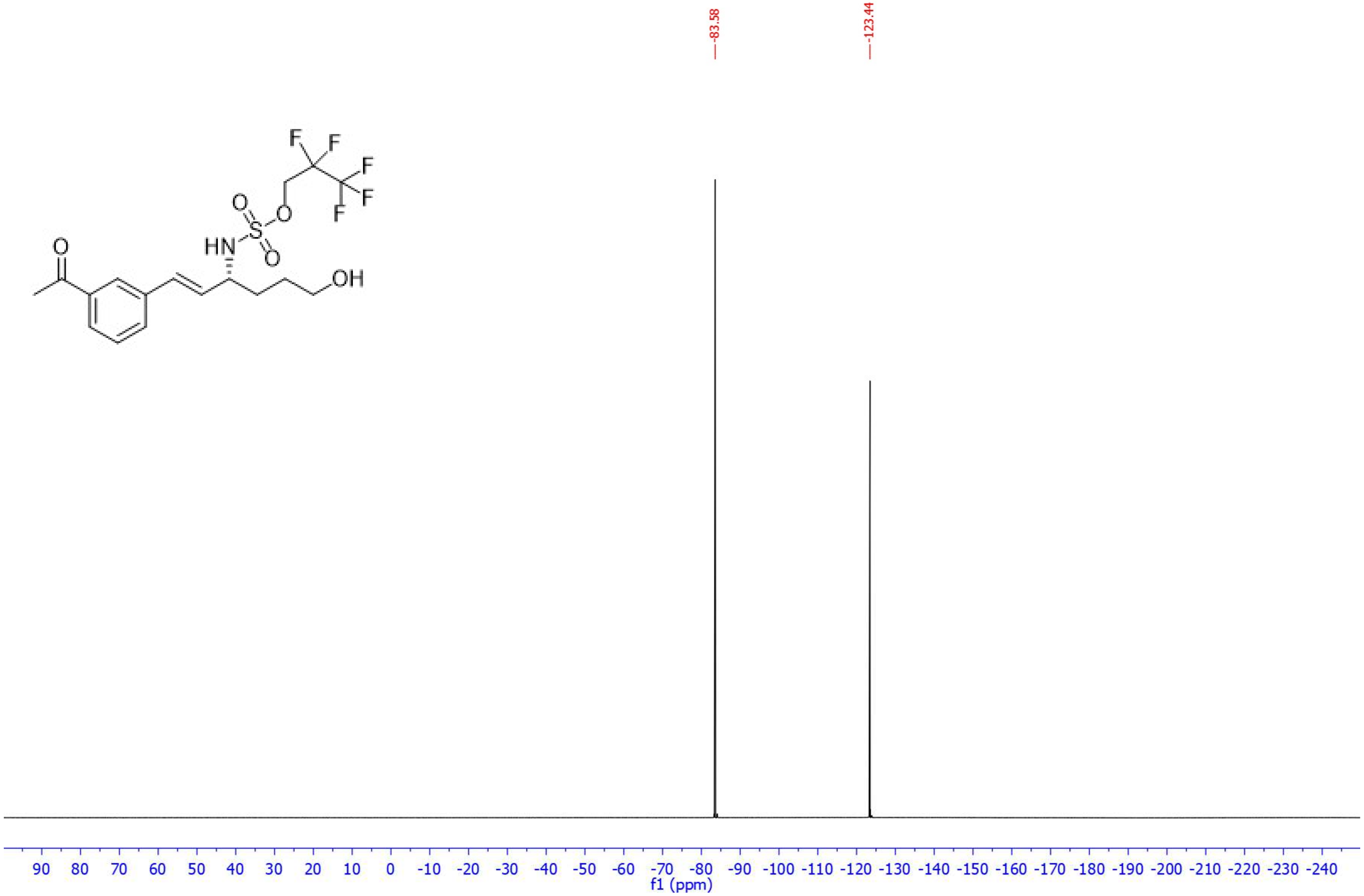

^{1}H NMR (700 MHz, $CDCl_3$) for ***2,2,2-trichloroethyl (R,E)-(1-([1,1'-biphenyl]-4-yl)-6-hydroxyhex-1-en-3-yl)sulfamate (2c)***

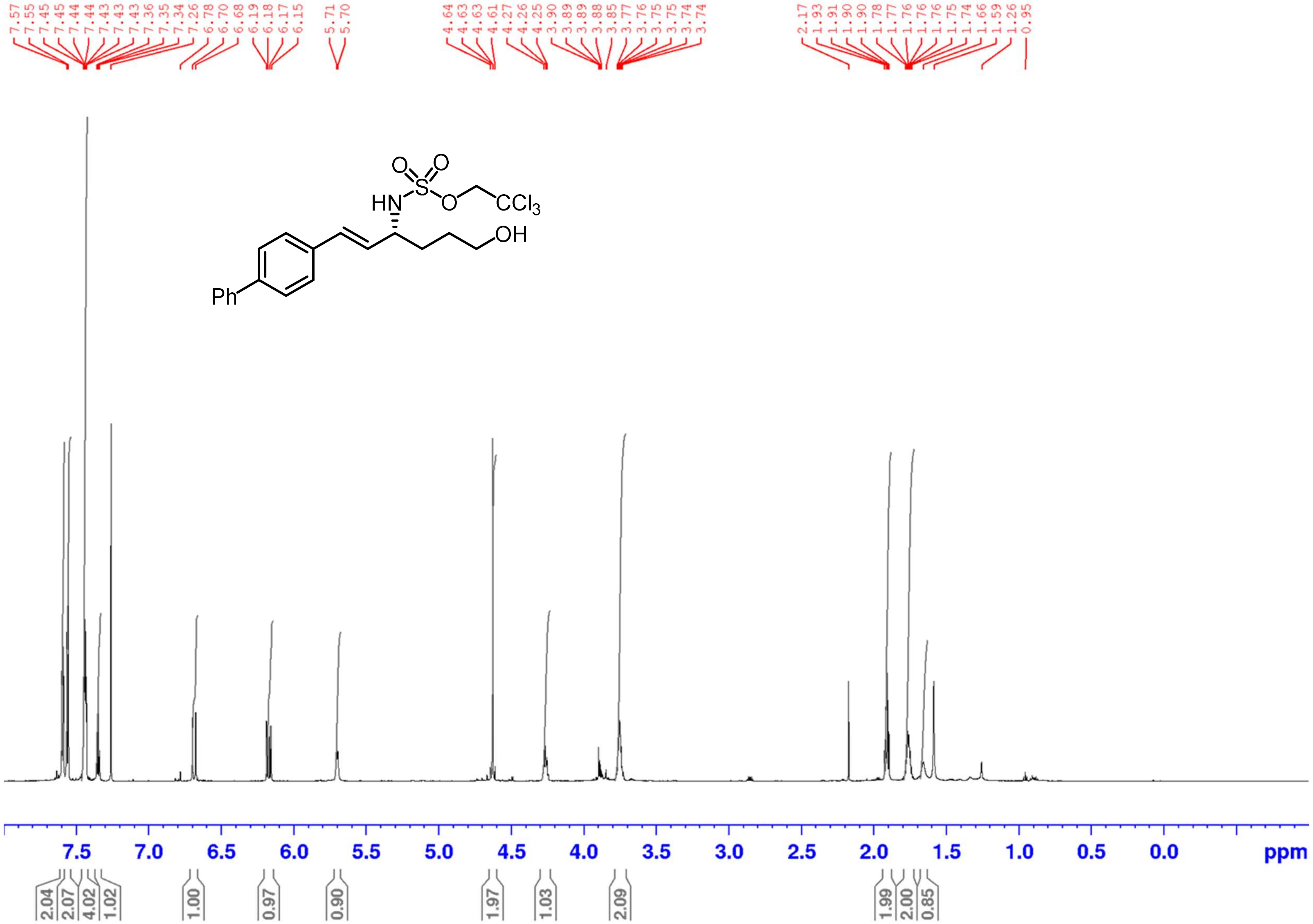

^{13}C NMR (176 MHz, $CDCl_3$) for ***2,2,2-trichloroethyl (R,E)-(1-([1,1'-biphenyl]-4-yl)-6-hydroxyhex-1-en-3-yl)sulfamate (2c)***

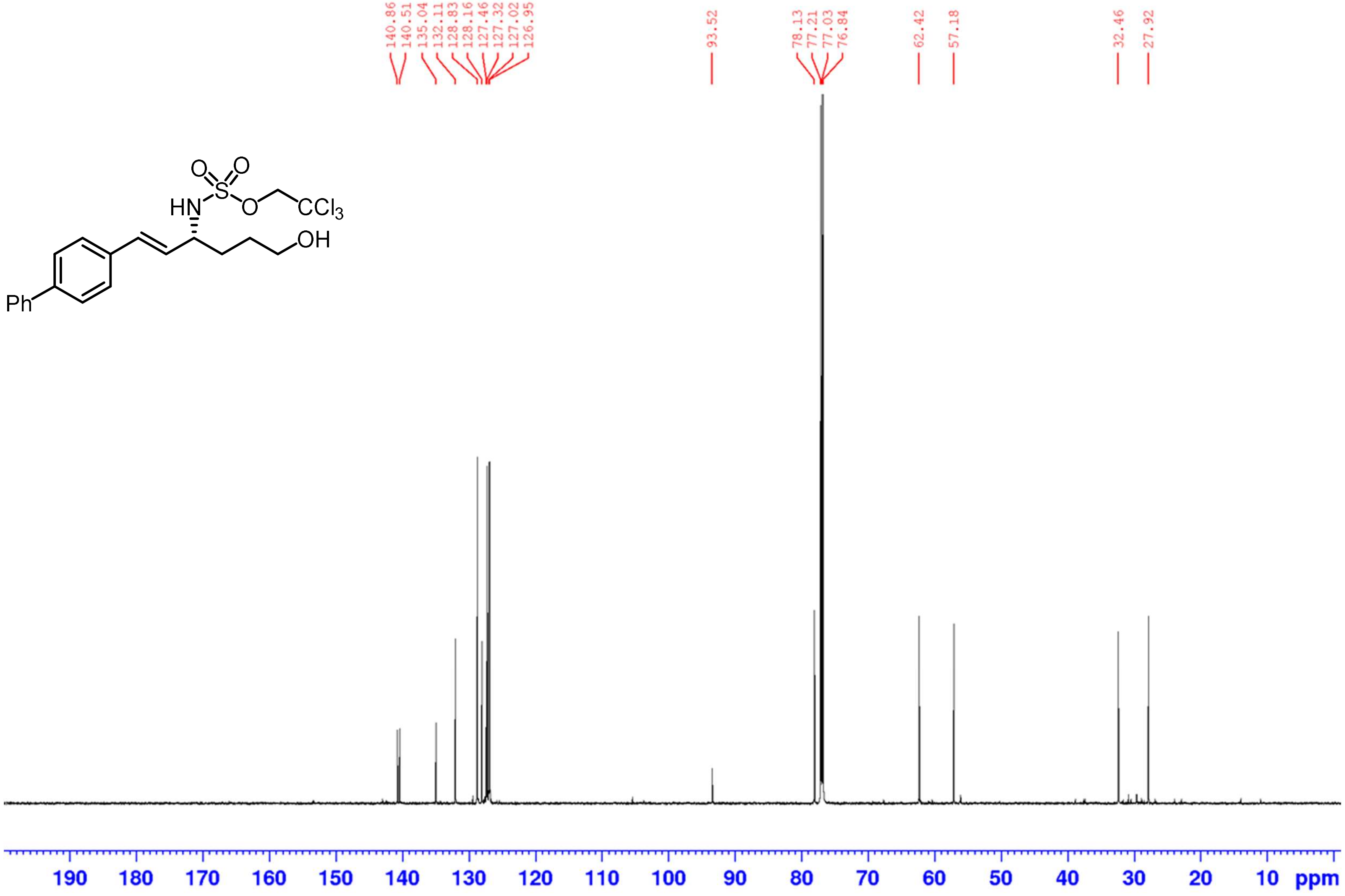

¹H NMR (700 MHz, $CDCl_3$) for ***2,2,2-trichloroethyl (R,E)-(1-(4-fluorophenyl)-6-hydroxyhex-1-en-3-yl)sulfamate (2d)***

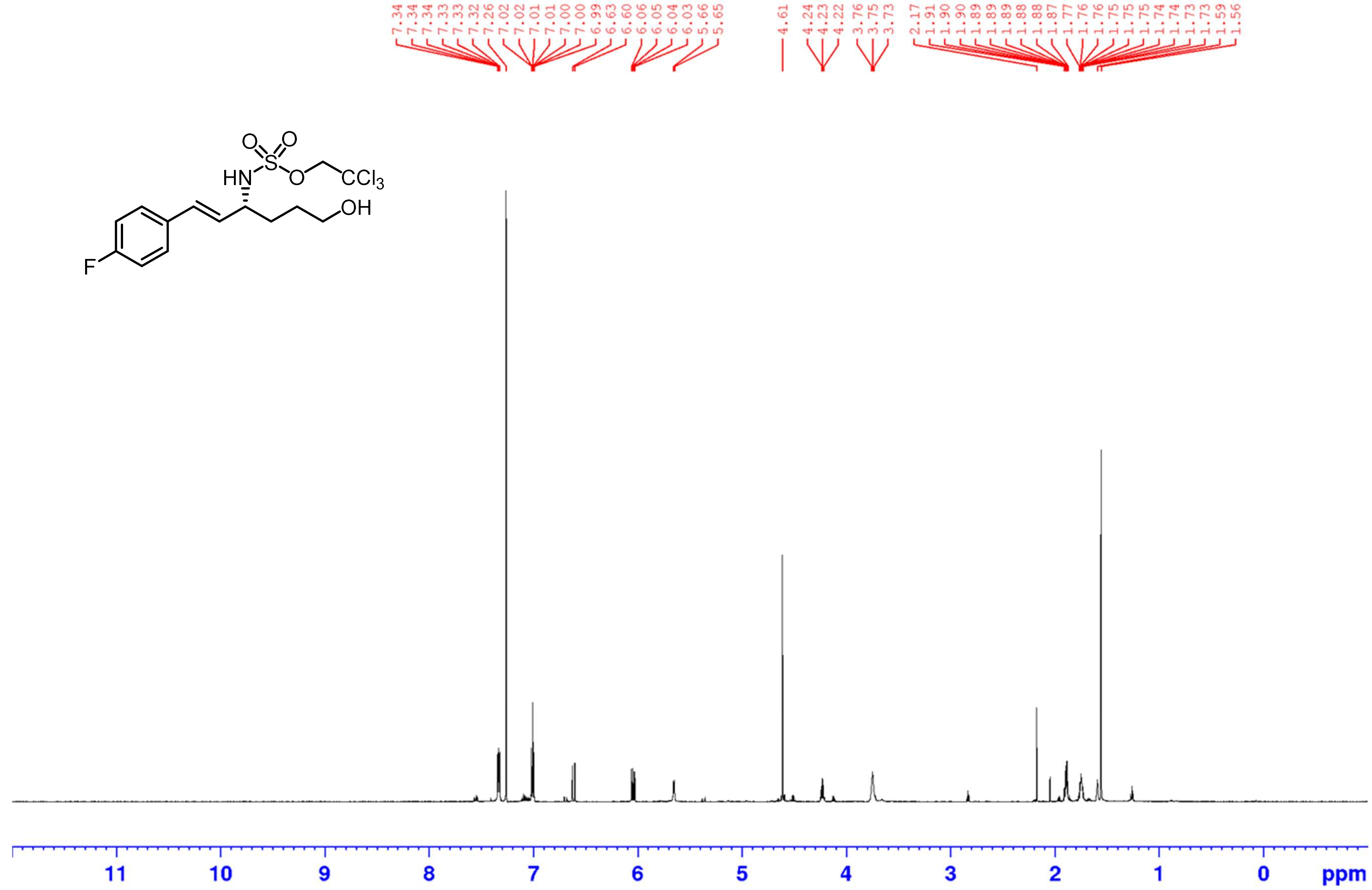

^{13}C **NMR** (176 MHz, $CDCl_3$) for ***2,2,2-trichloroethyl (R,E)-(1-(4-fluorophenyl)-6-hydroxyhex-1-en-3-yl)sulfamate (2d)***

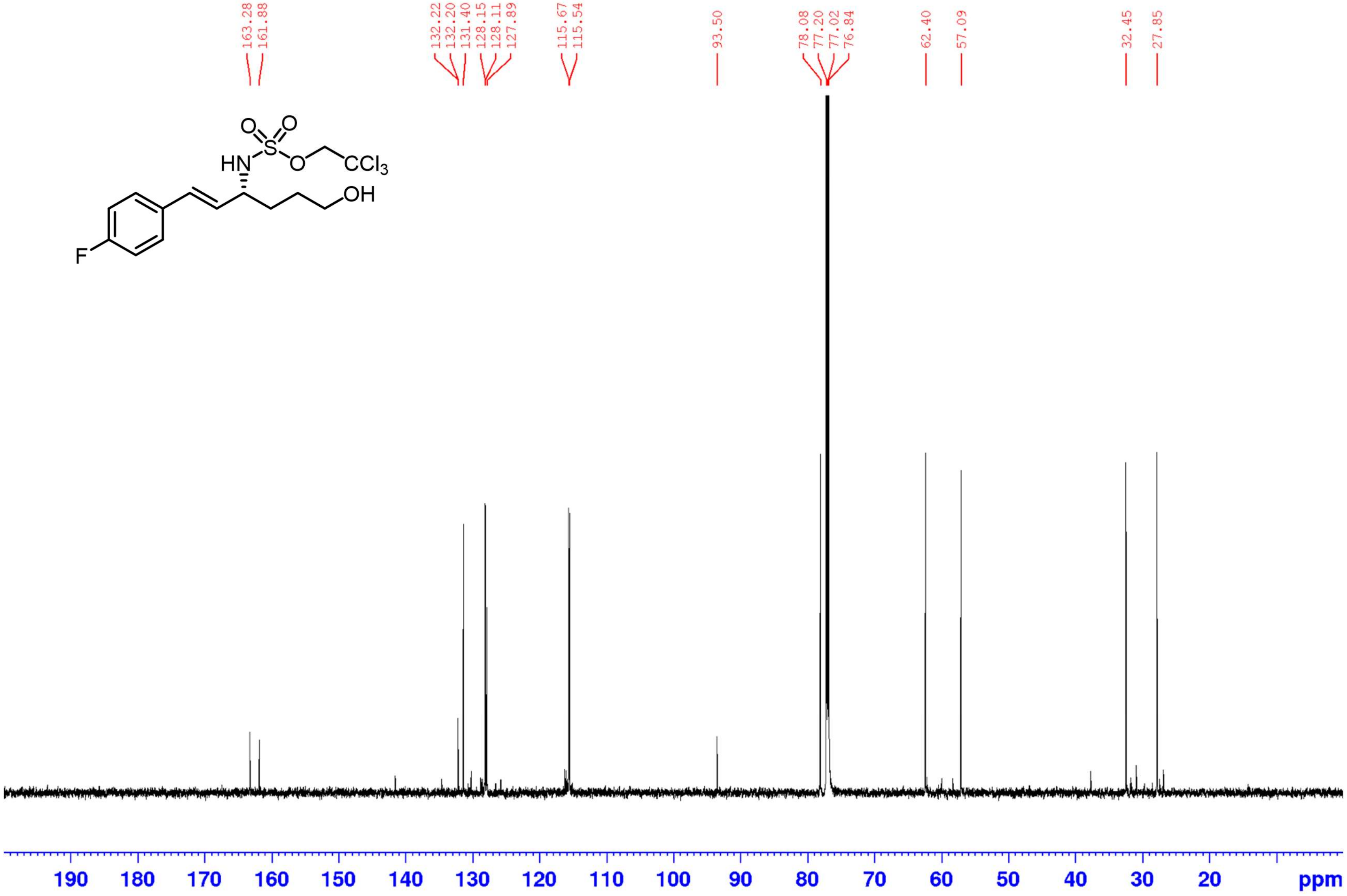

[19]**F NMR** (376 MHz, $CDCl_3$) for ***2,2,2-trichloroethyl (R,E)-(1-(4-fluorophenyl)-6-hydroxyhex-1-en-3-yl)sulfamate (2d)***

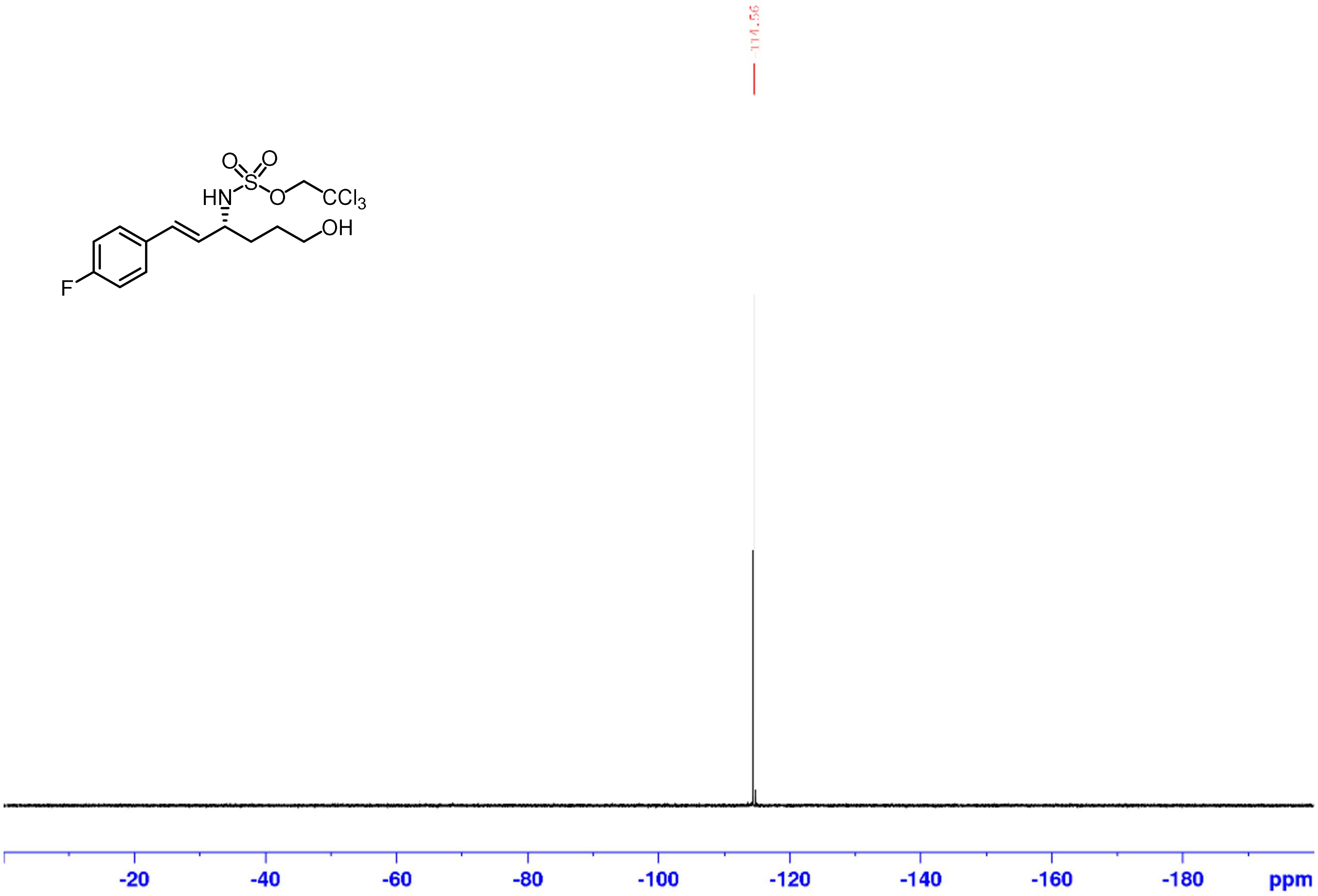

^{1}H NMR (700 MHz, $CDCl_3$) for ***2,2,2-trichloroethyl (R,E)-(1-(4-(tert-butyl)phenyl)-6-hydroxyhex-1-en-3-yl)sulfamate (2e)***

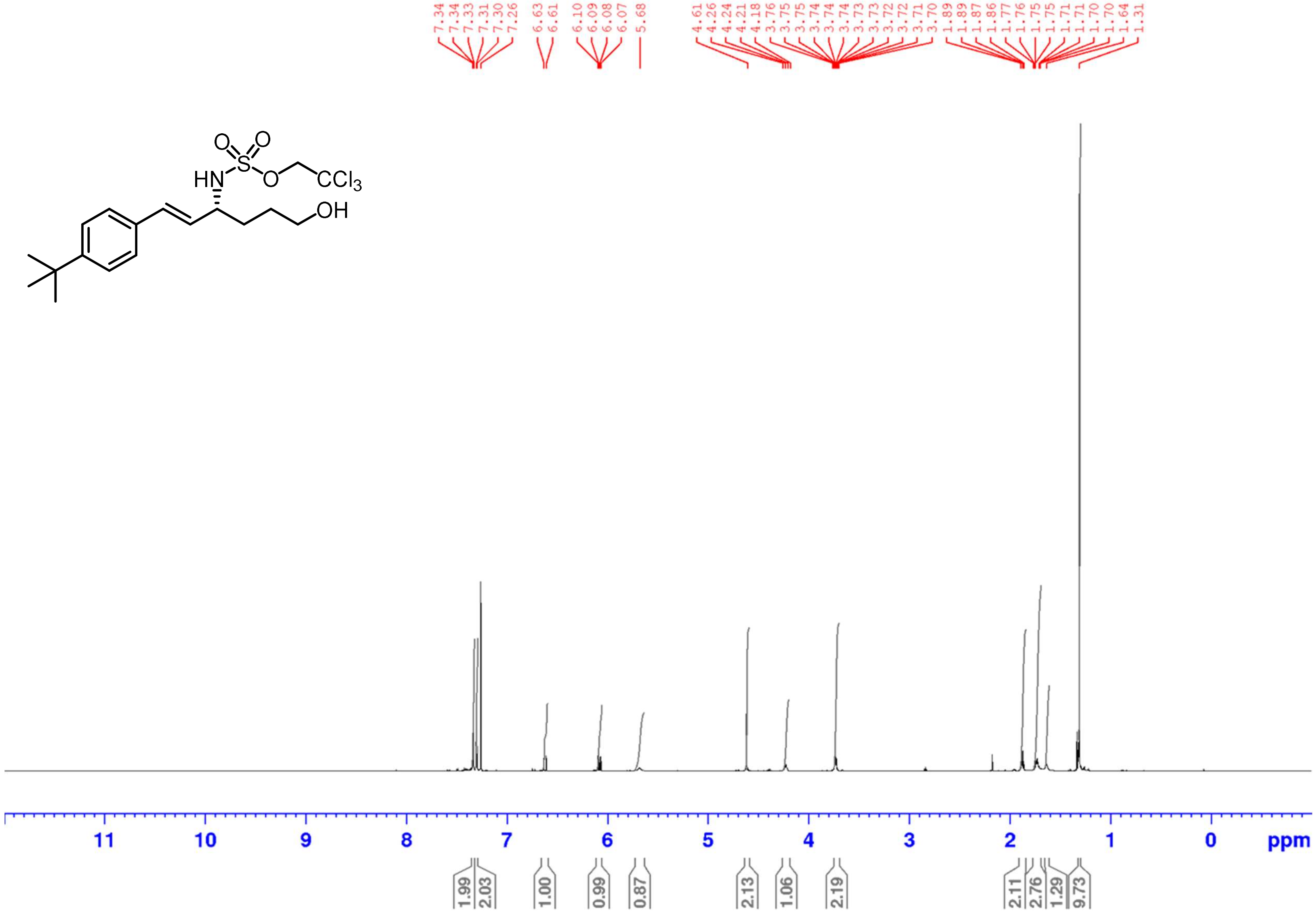

^{13}**C NMR** (176 MHz, $CDCl_3$) for ***2,2,2-trichloroethyl (R,E)-(1-(4-(tert-butyl)phenyl)-6-hydroxyhex-1-en-3-yl)sulfamate (2e)***

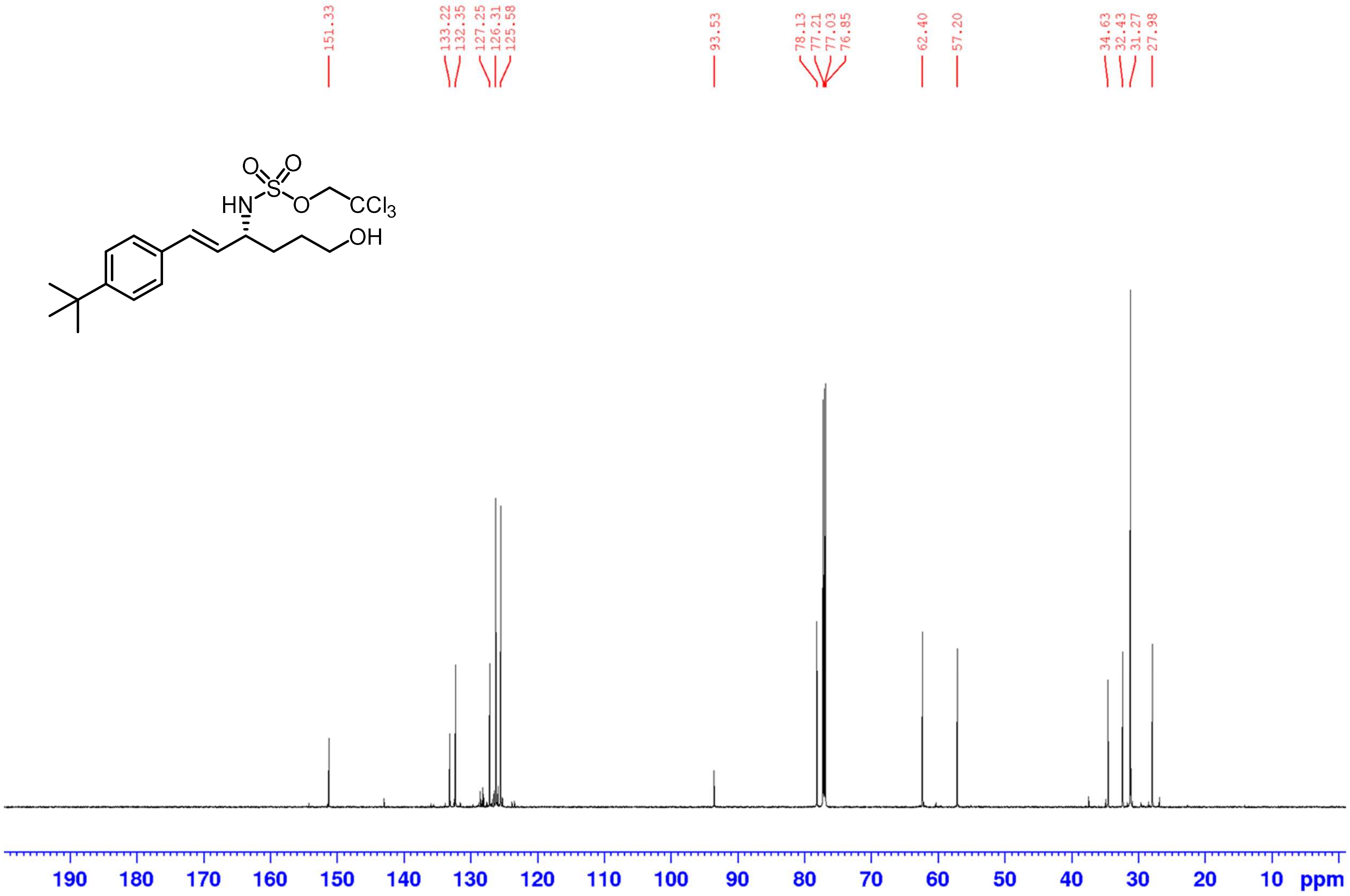

^{1}H NMR (700 MHz, $CDCl_3$) for ***(R,E)-3-(6-hydroxy-3-(((2,2,2-trichloroethoxy)sulfonyl)amino)hex-1-en-1-yl)phenyl acetate (2f)***

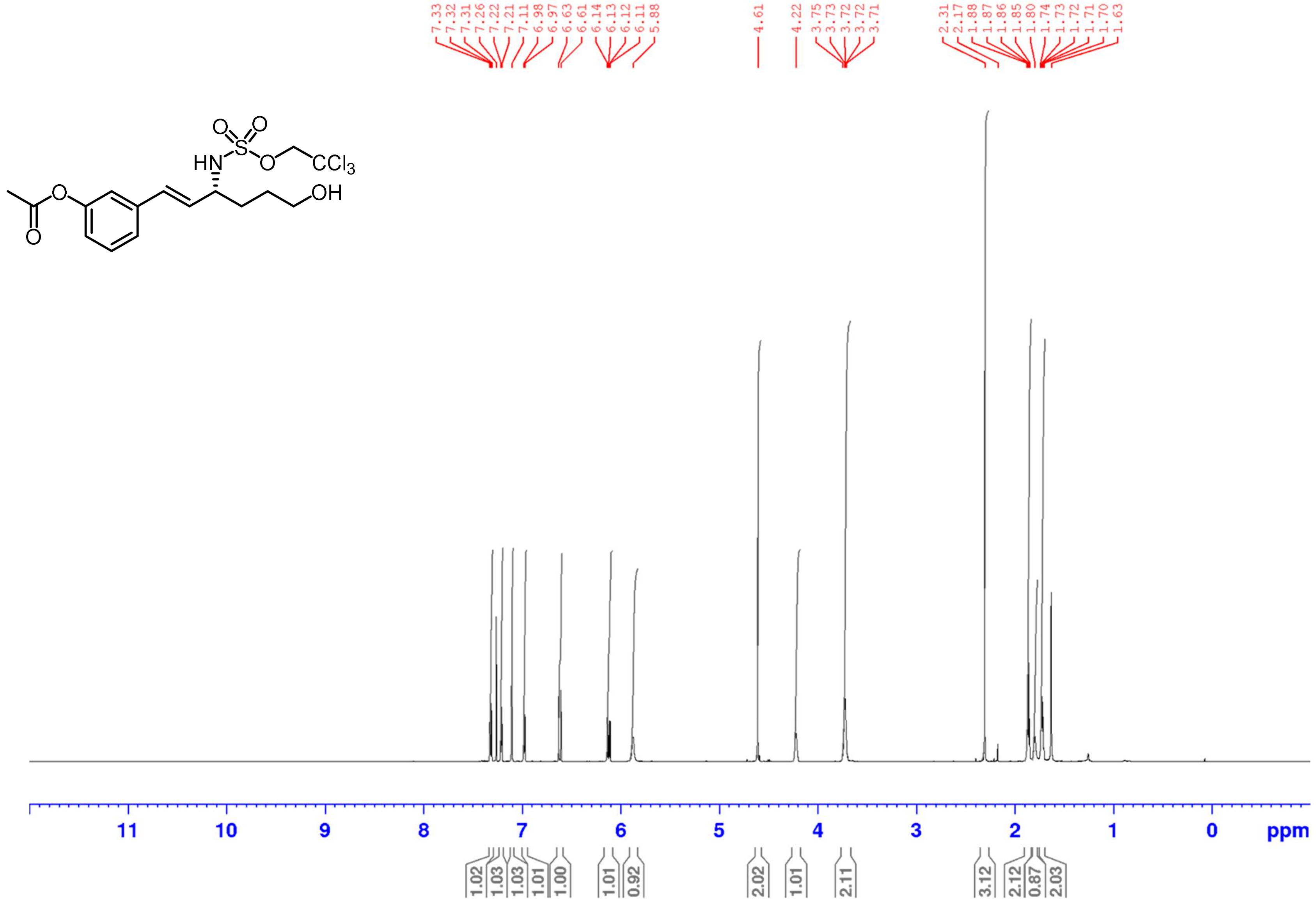

^{13}C NMR (176 MHz, $CDCl_3$) for ***(R,E)-3-(6-hydroxy-3-(((2,2,2-trichloroethoxy)sulfonyl)amino)hex-1-en-1-yl)phenyl acetate (2f)***

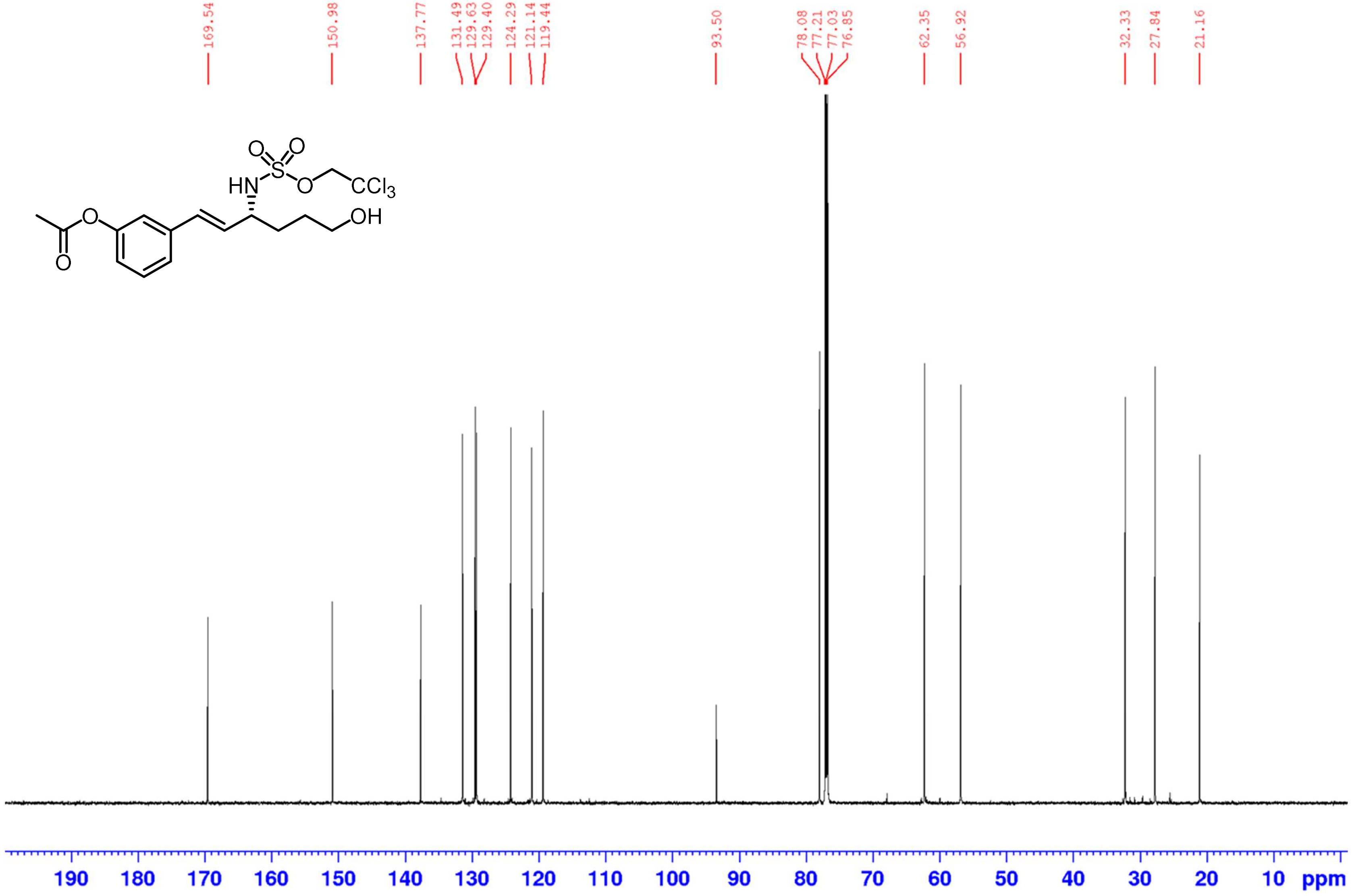

^{1}H NMR (700 MHz, $CDCl_3$) for ***2,2,2-trichloroethyl (R,E)-(1-(3-acetylphenyl)-6-hydroxyhex-1-en-3-yl)sulfamate (2b)***

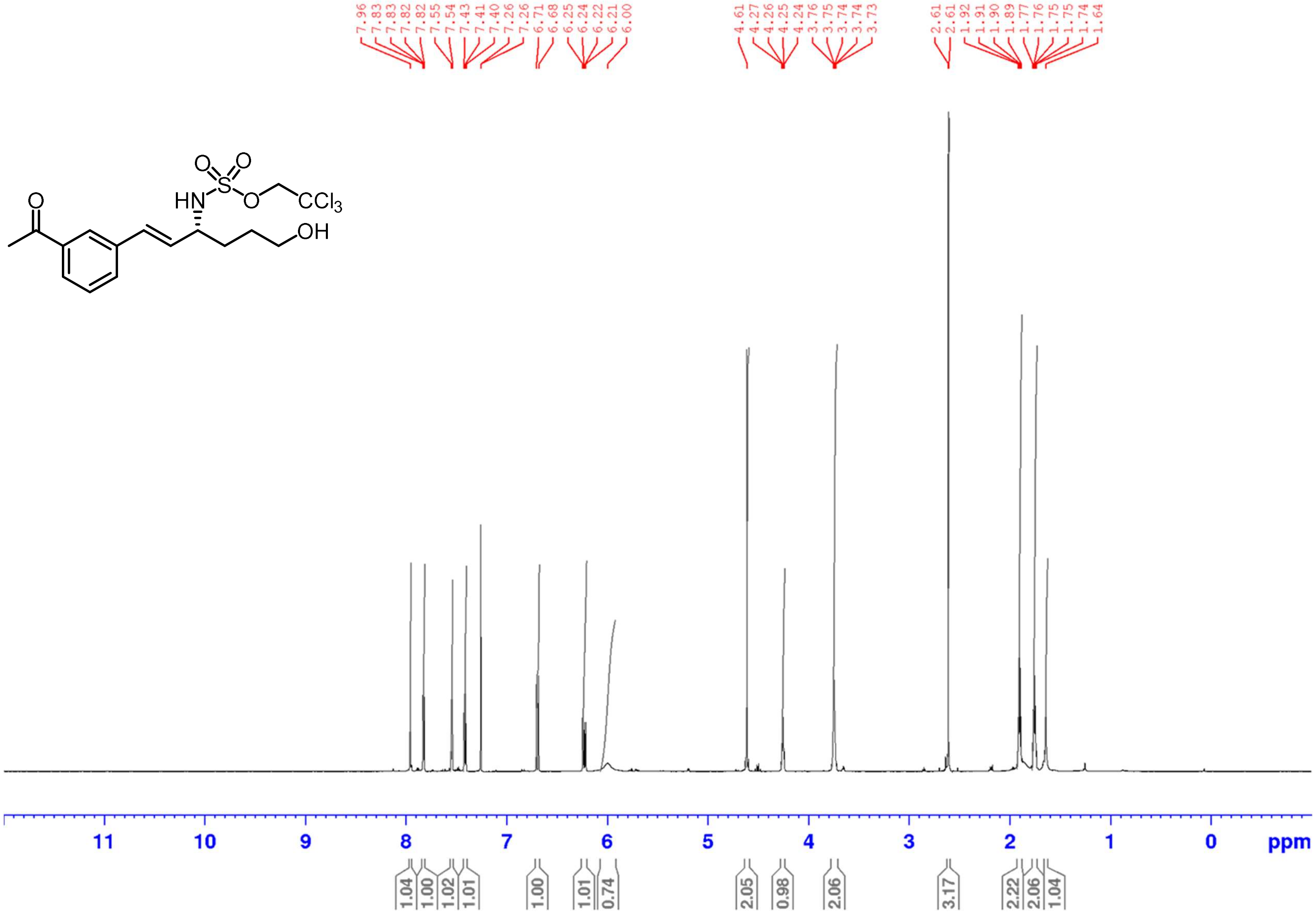

^{13}C NMR (176 MHz, $CDCl_3$) for ***2,2,2-trichloroethyl (R,E)-(1-(3-acetylphenyl)-6-hydroxyhex-1-en-3-yl)sulfamate (2b)***

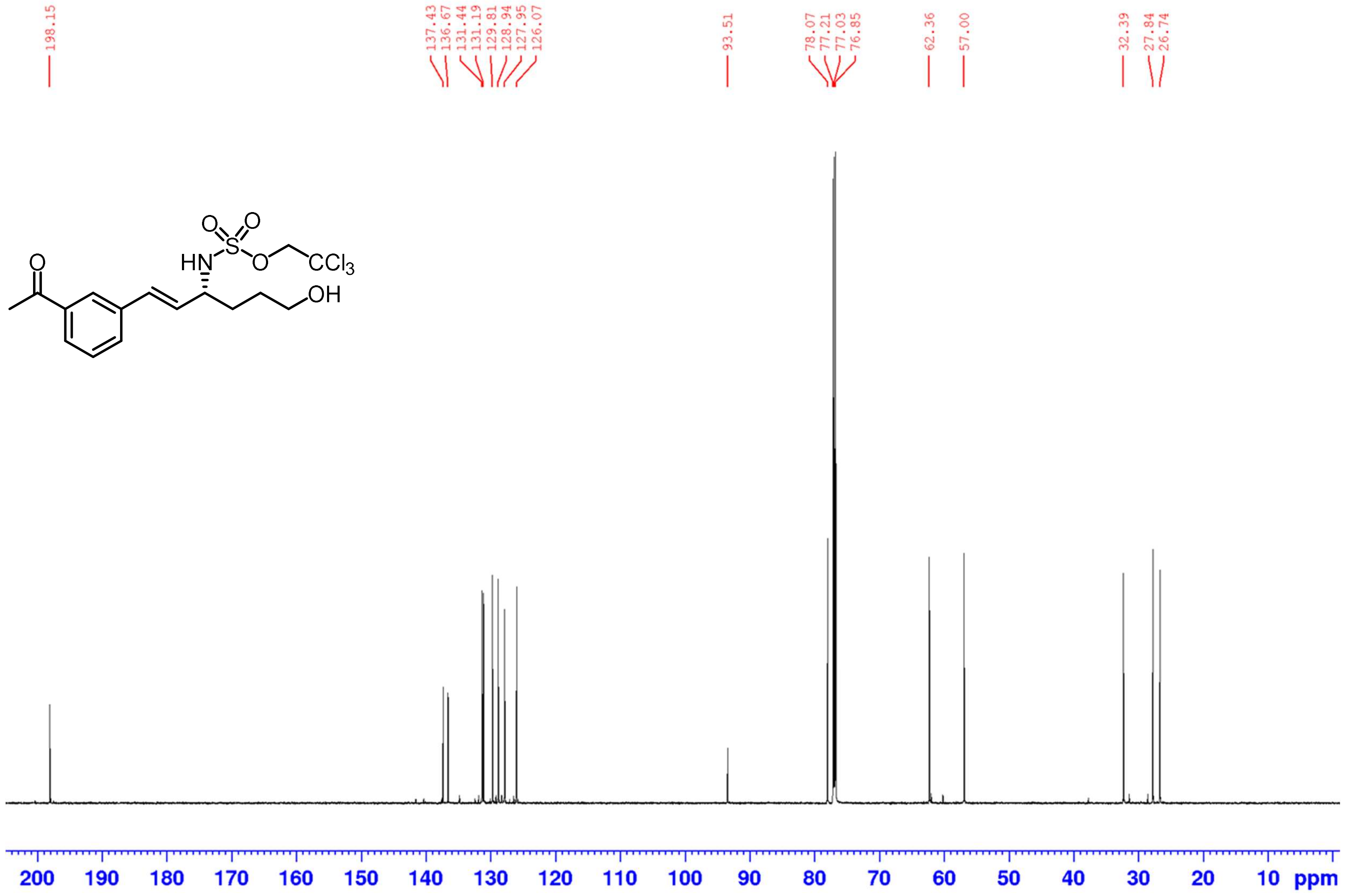

^{1}H NMR (700 MHz, $CDCl_3$) for ***2,2,2-trichloroethyl (R,E)-(1-(3-(tert-butyl)phenyl)-6-hydroxyhex-1-en-3-yl)sulfamate (2g)***

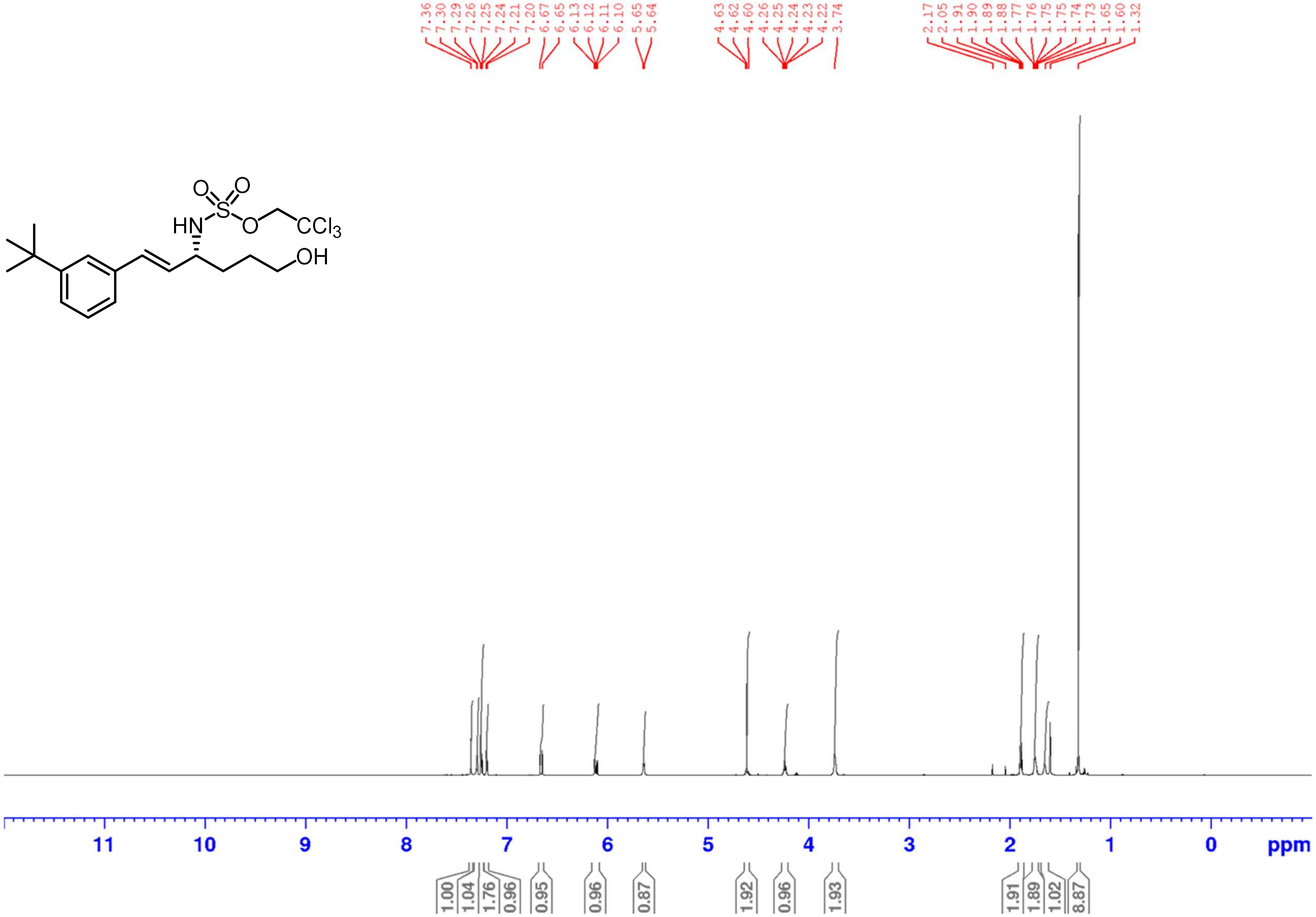

^{13}C NMR (176 MHz, $CDCl_3$) for ***2,2,2-trichloroethyl (R,E)-(1-(3-(tert-butyl)phenyl)-6-hydroxyhex-1-en-3-yl)sulfamate (2g)***

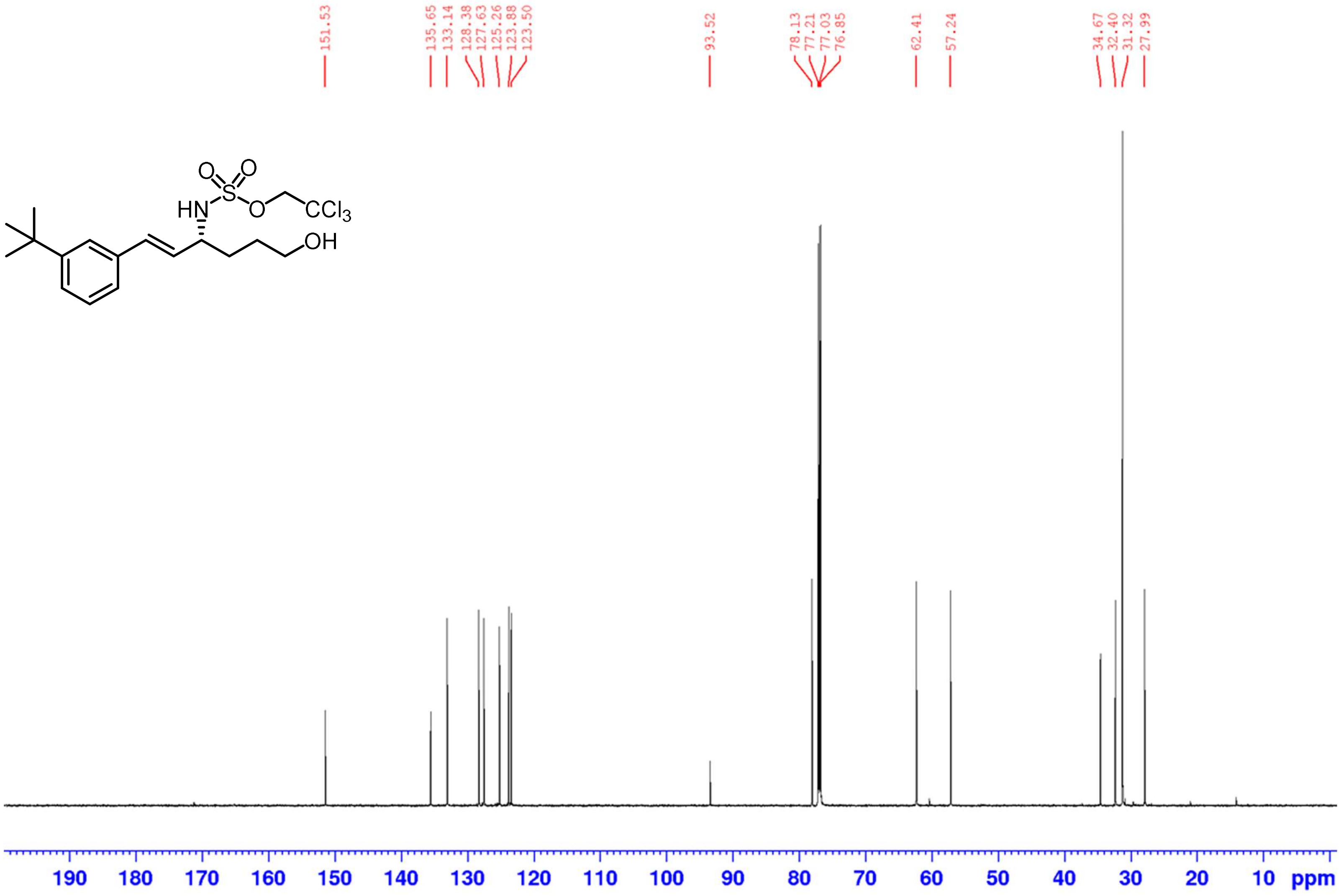

^{1}H NMR (700 MHz, $CDCl_3$) for ***2,2,2-trichloroethyl (R,E)-(6-hydroxy-1-(3-methoxyphenyl)hex-1-en-3-yl)sulfamate (2h)***

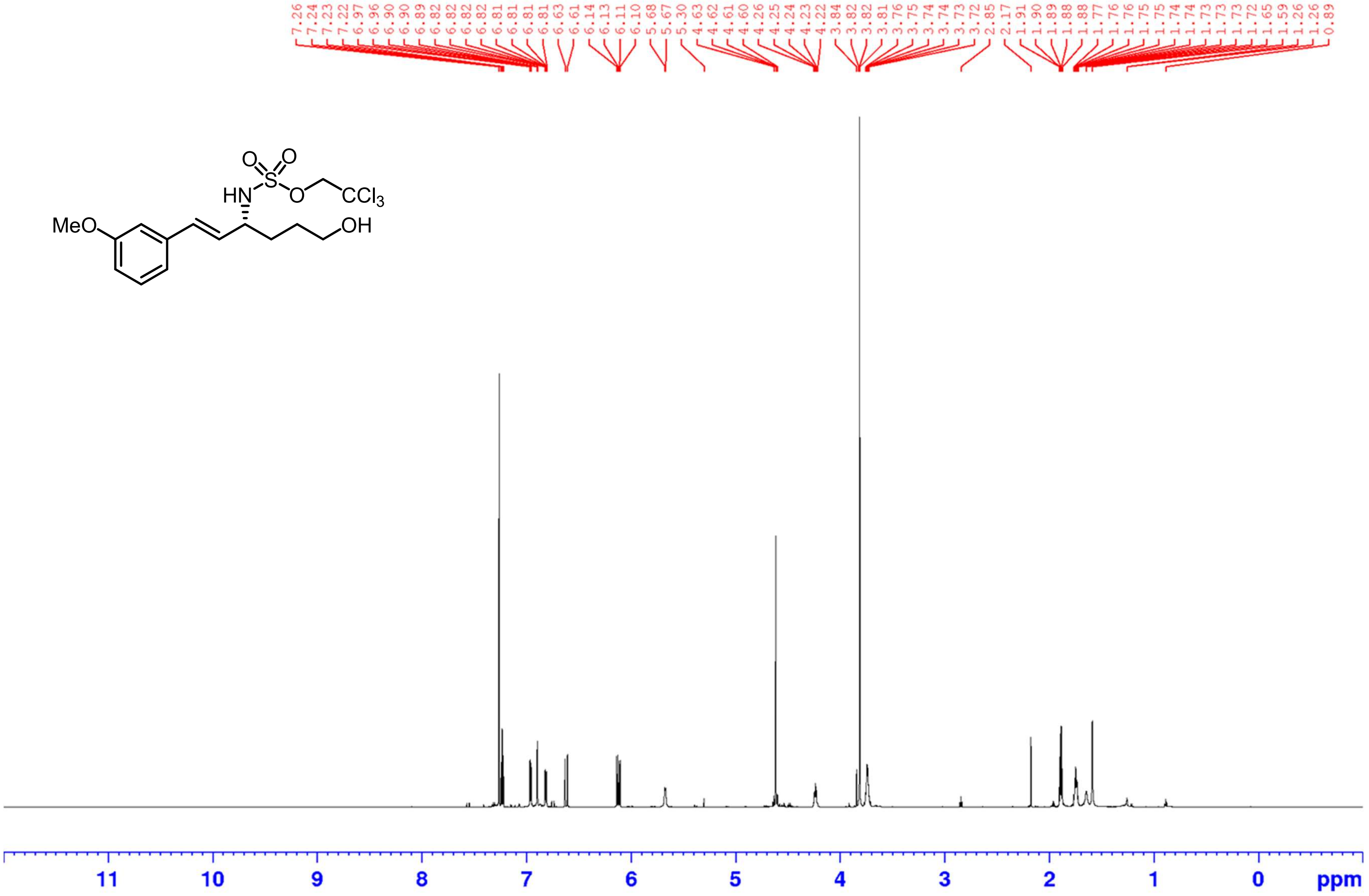

^{13}C NMR (176 MHz, $CDCl_3$) for ***2,2,2-trichloroethyl (R,E)-(6-hydroxy-1-(3-methoxyphenyl)hex-1-en-3-yl)sulfamate (2h)***

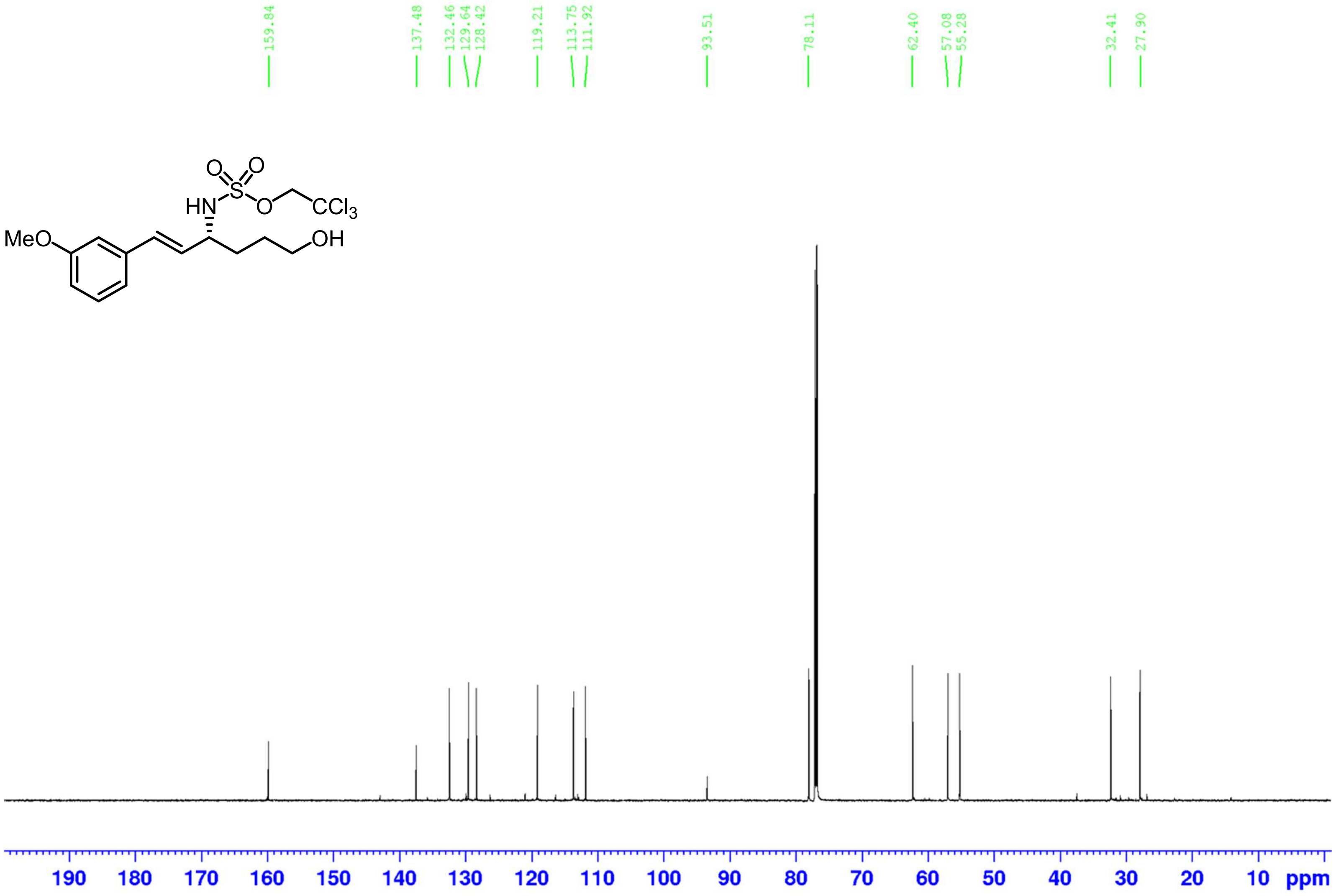

^{1}H NMR (700 MHz, $CDCl_3$) for ***Methyl (R,E)-3-(6-hydroxy-3-(((2,2,2-trichloroethoxy)sulfonyl)amino)hex-1-en-1-yl)benzoate (2i)***

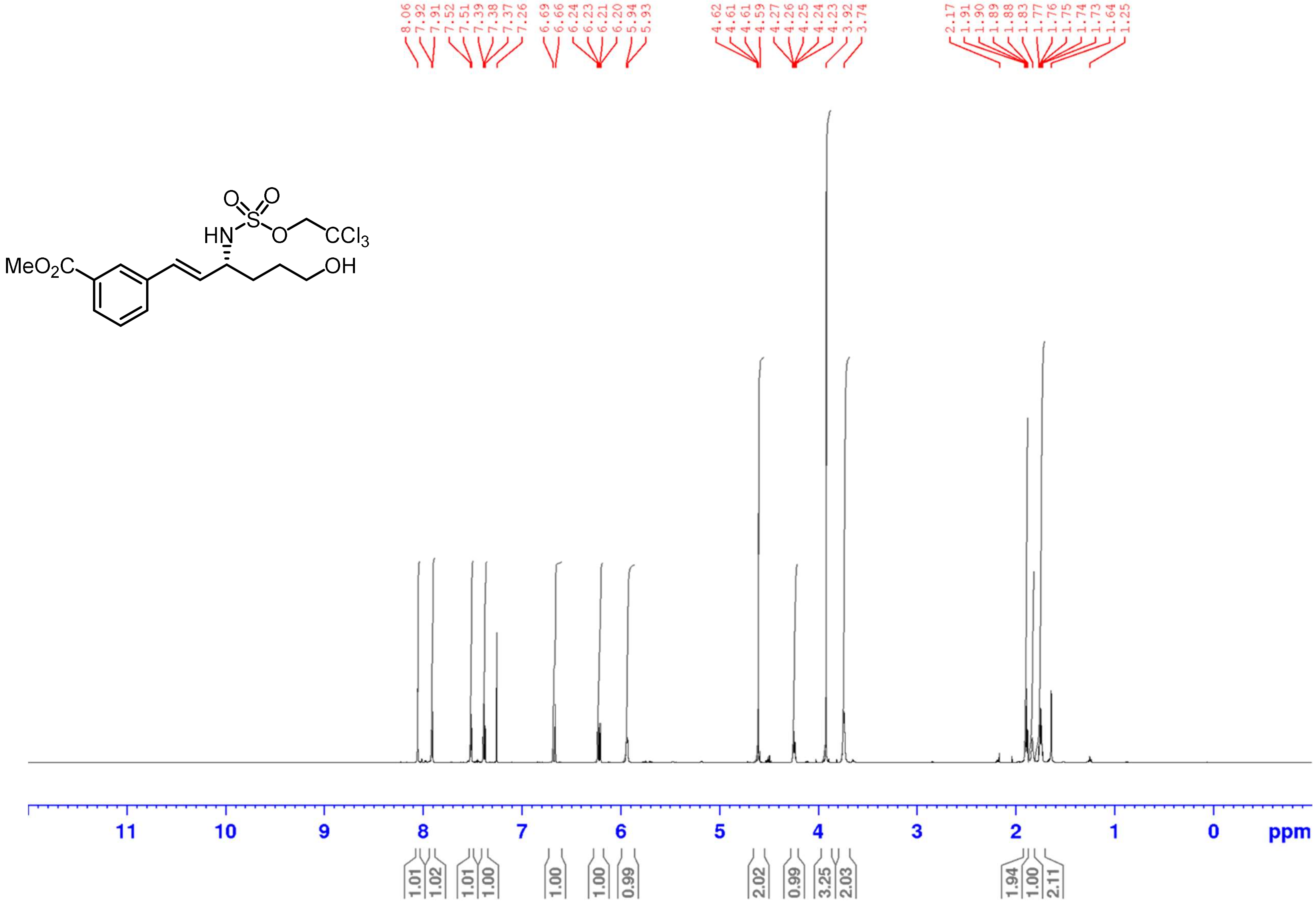

^{13}C NMR (176 MHz, $CDCl_3$) for ***Methyl (R,E)-3-(6-hydroxy-3-(((2,2,2-trichloroethoxy)sulfonyl)amino)hex-1-en-1-yl)benzoate (2i)***

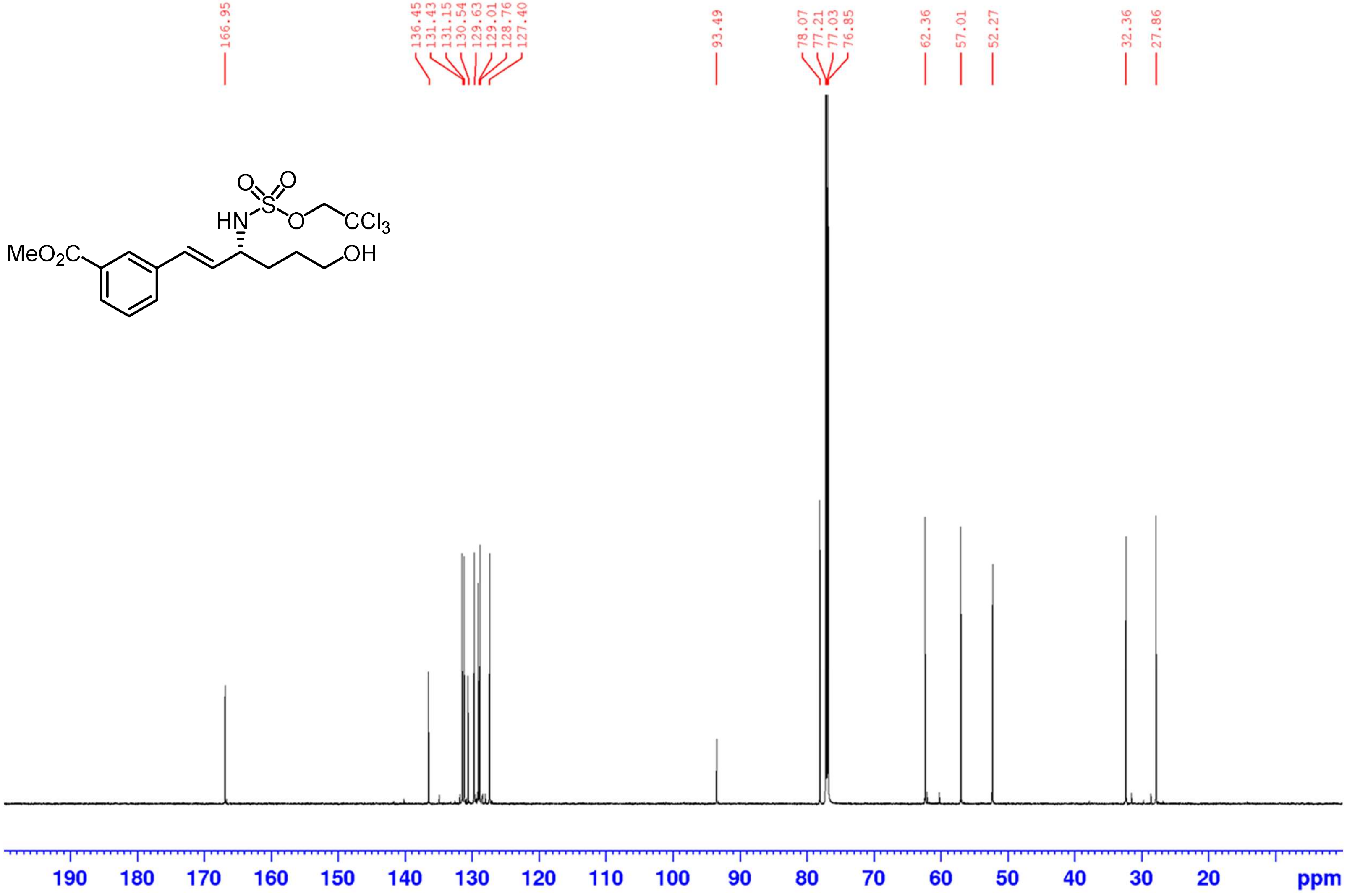

¹H NMR (700 MHz, $CDCl_3$) for ***2,2,2-trichloroethyl (R,E)-(1-(3-((tert-butoxycarbonyl)amino)phenyl)-6-hydroxyhex-1-en-3-yl)sulfamate (2j)***

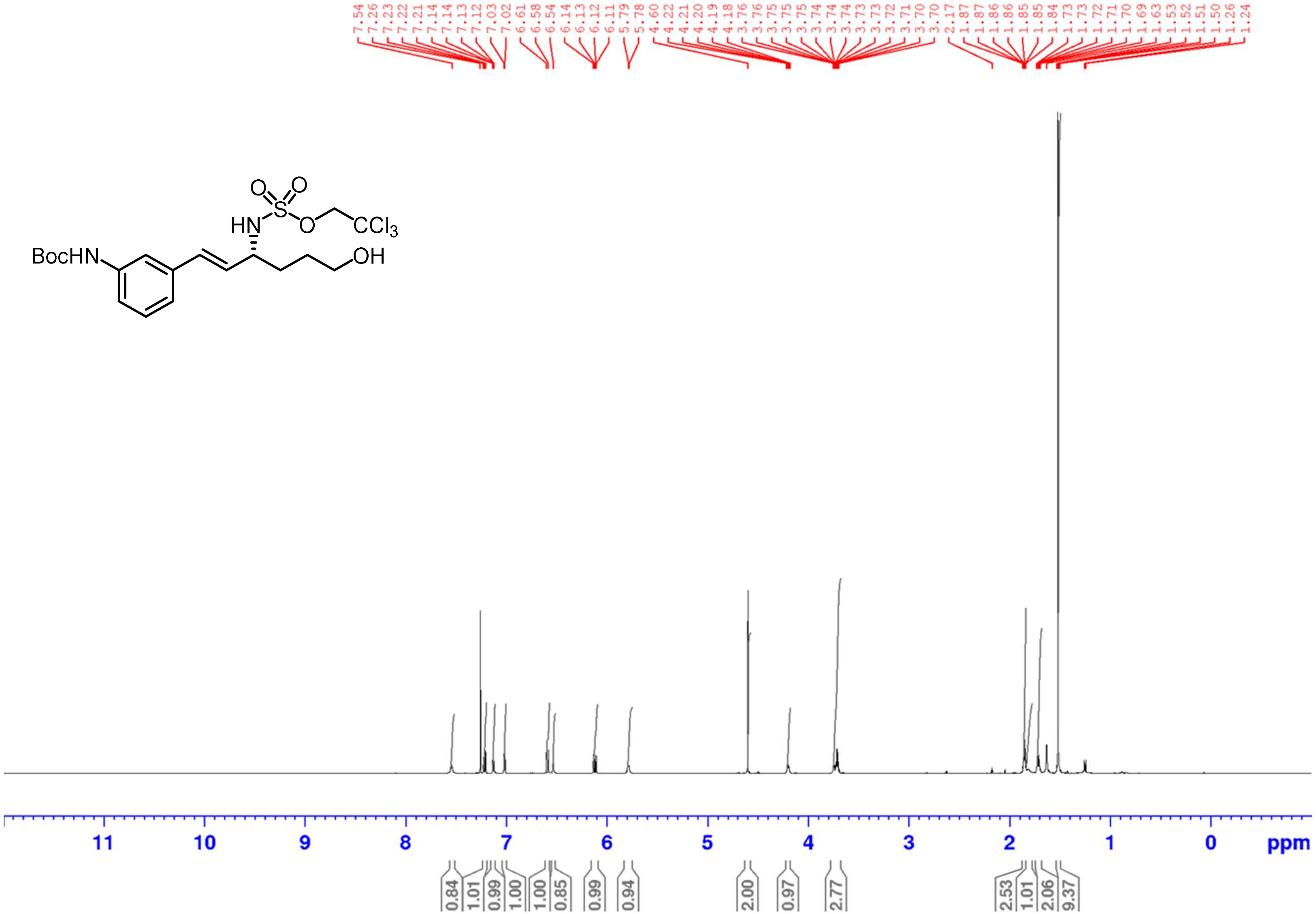

^{13}C **NMR** (176 MHz, $CDCl_3$) for ***2,2,2-trichloroethyl (R,E)-(1-(3-((tert-butoxycarbonyl)amino)phenyl)-6-hydroxyhex-1-en-3-yl)sulfamate (2j)***

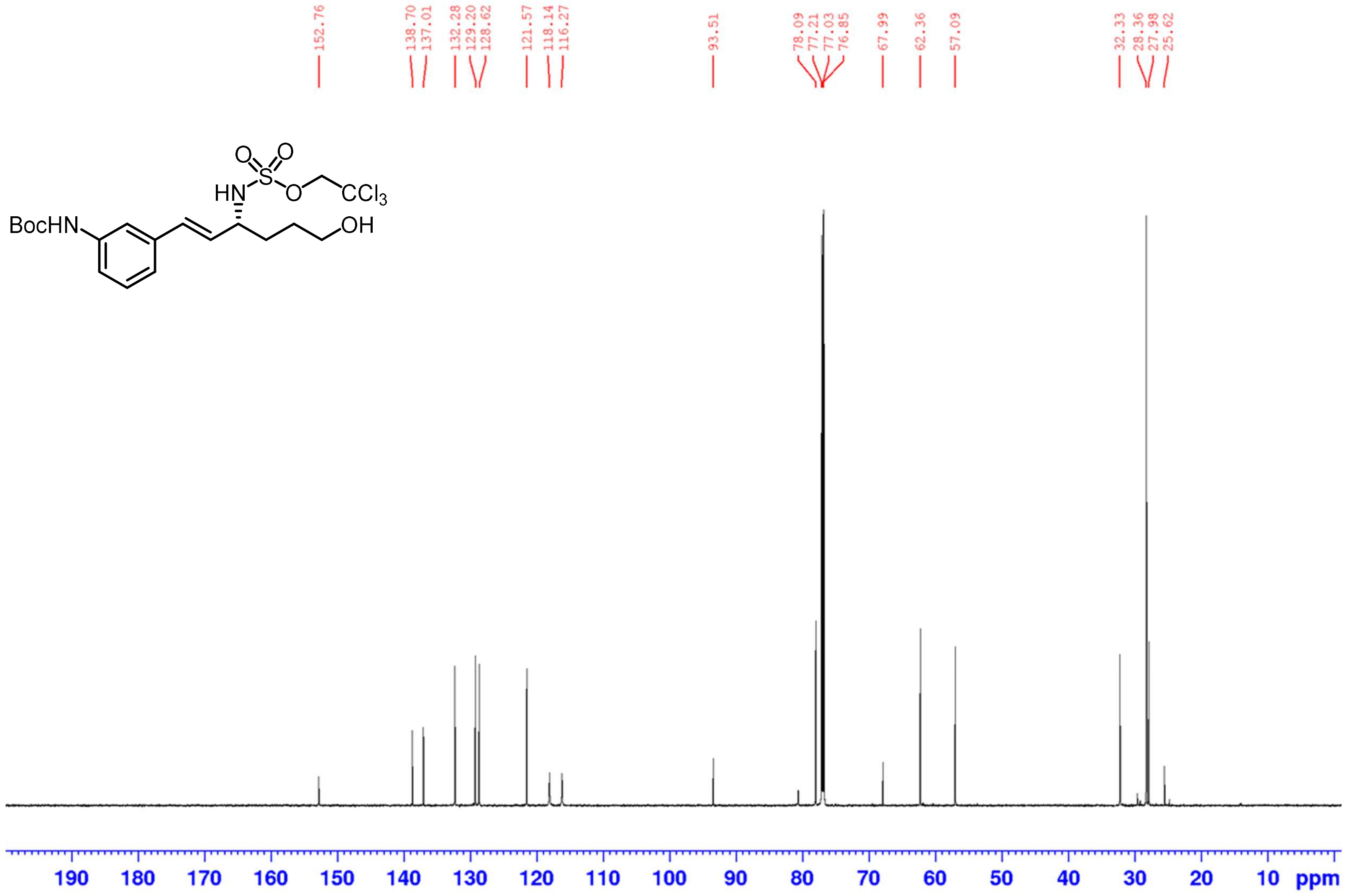

^{1}H NMR (700 MHz, $CDCl_3$) for ***2,2,2-trichloroethyl (R,E)-(6-hydroxy-1-(2-methoxyphenyl)hex-1-en-3-yl)sulfamate (2k)***

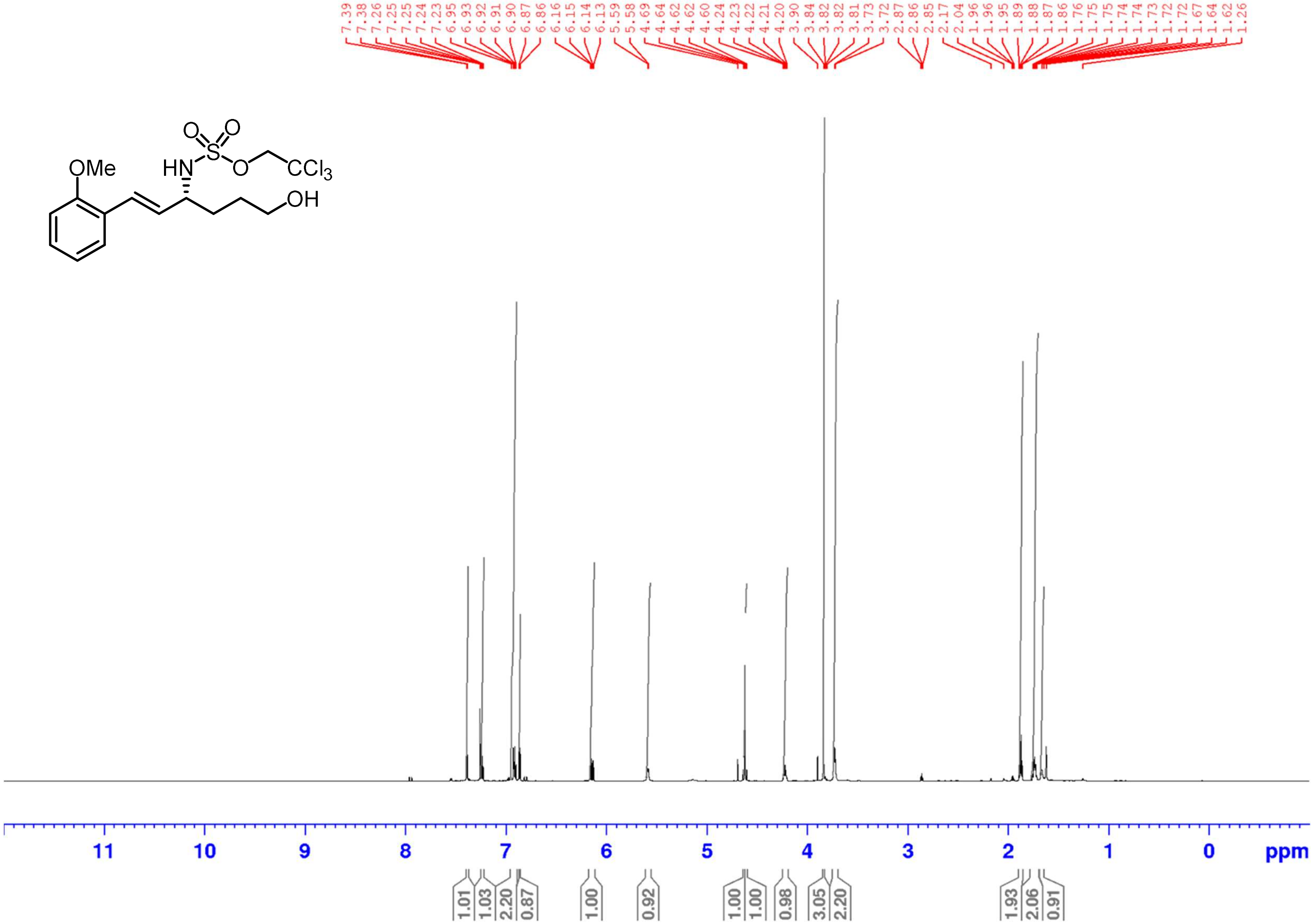

^{13}C NMR (176 MHz, $CDCl_3$) for ***2,2,2-trichloroethyl (R,E)-(6-hydroxy-1-(2-methoxyphenyl)hex-1-en-3-yl)sulfamate (2k)***

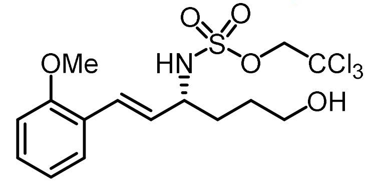

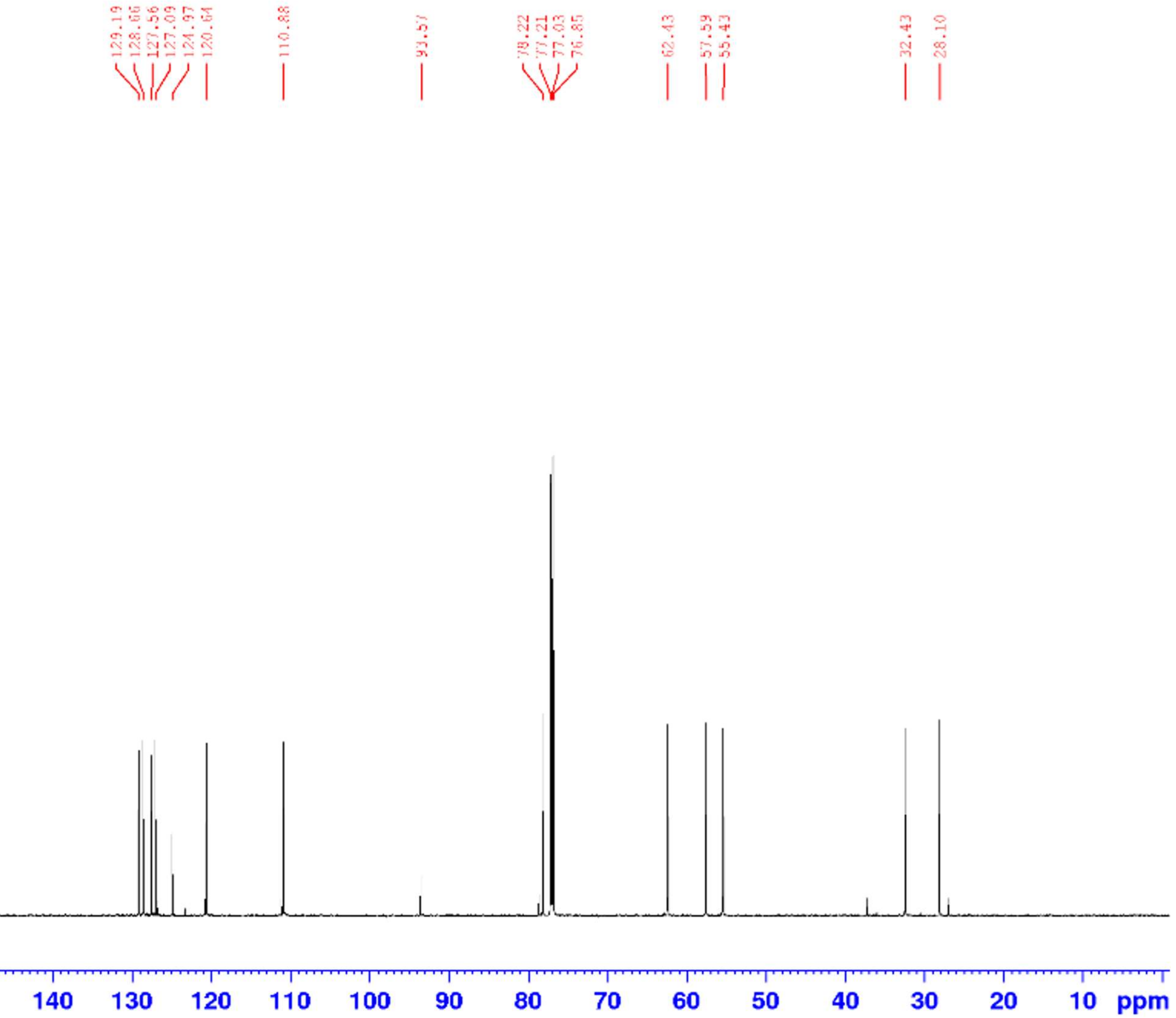

^{1}H NMR (700 MHz, $CDCl_3$) for *2,2,2-trichloroethyl (R,E)-(6-hydroxy-1-(o-tolyl)hex-1-en-3-yl)sulfamate (2l)*

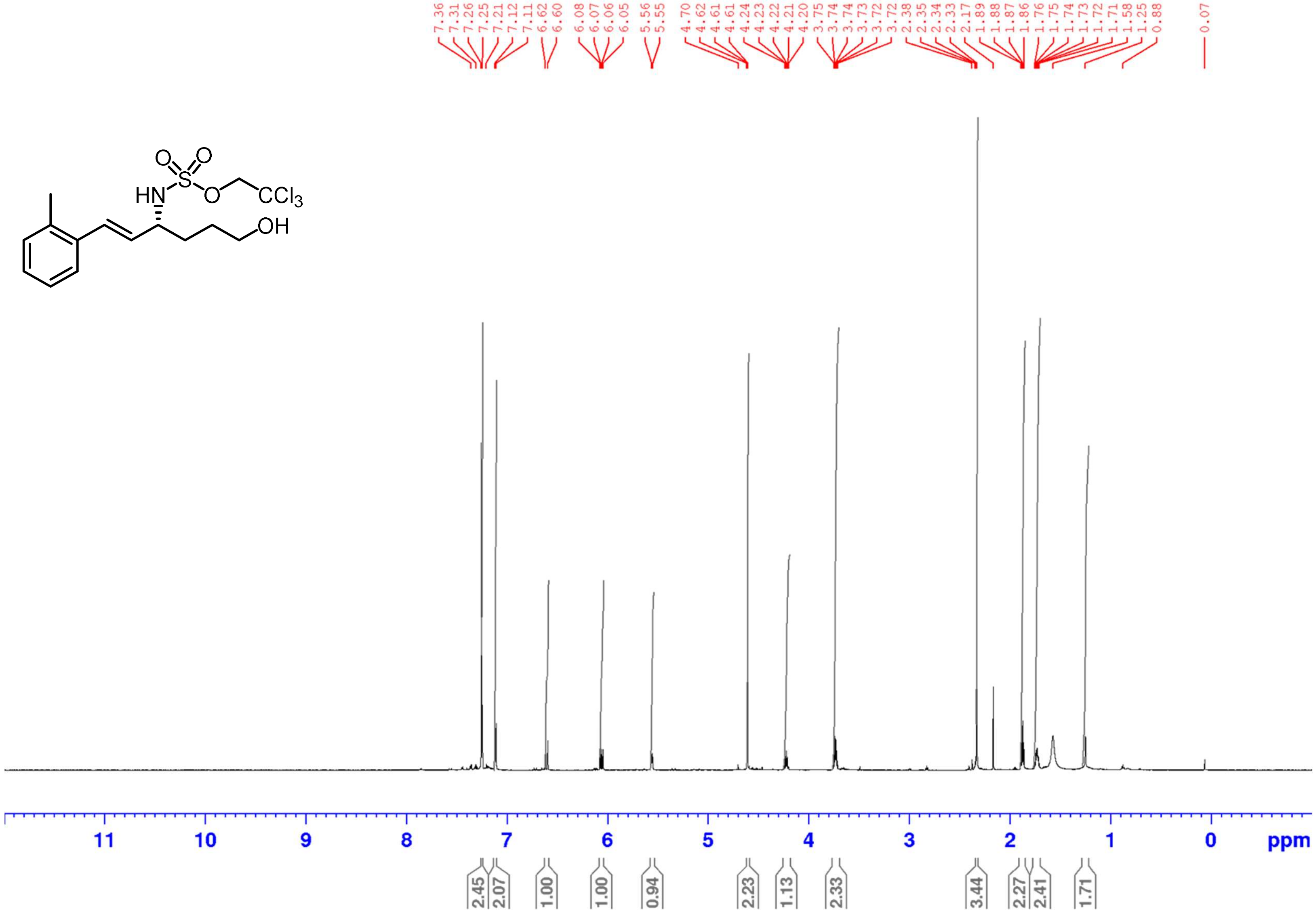

^{13}C NMR (176 MHz, $CDCl_3$) for ***2,2,2-trichloroethyl (R,E)-(6-hydroxy-1-(o-tolyl)hex-1-en-3-yl)sulfamate (2l)***

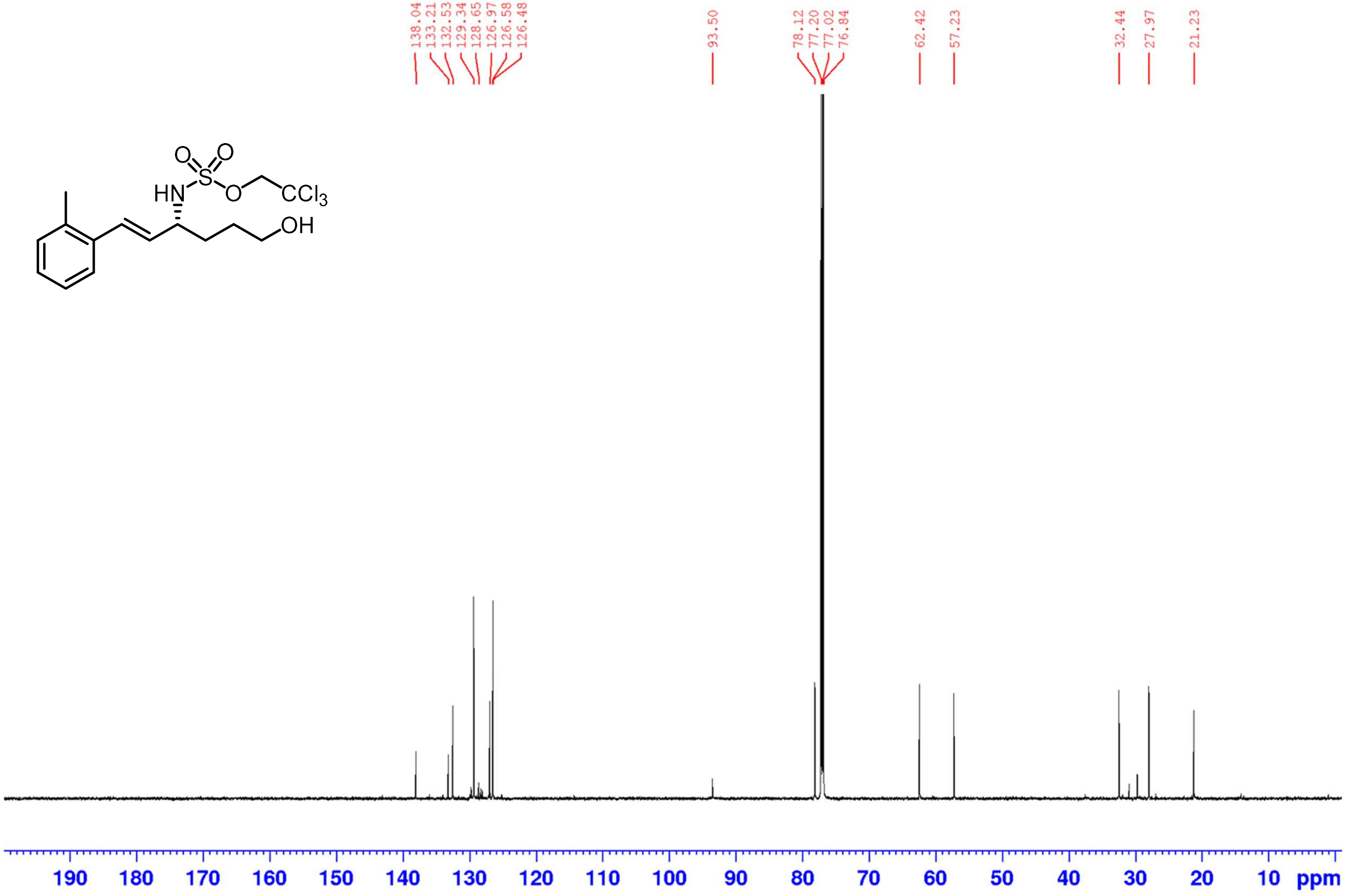

^{1}H NMR (700 MHz, $CDCl_3$) for ***2,2,2-trichloroethyl (R,E)-(1-(2-chlorophenyl)-6-hydroxyhex-1-en-3-yl)sulfamate (2m)***

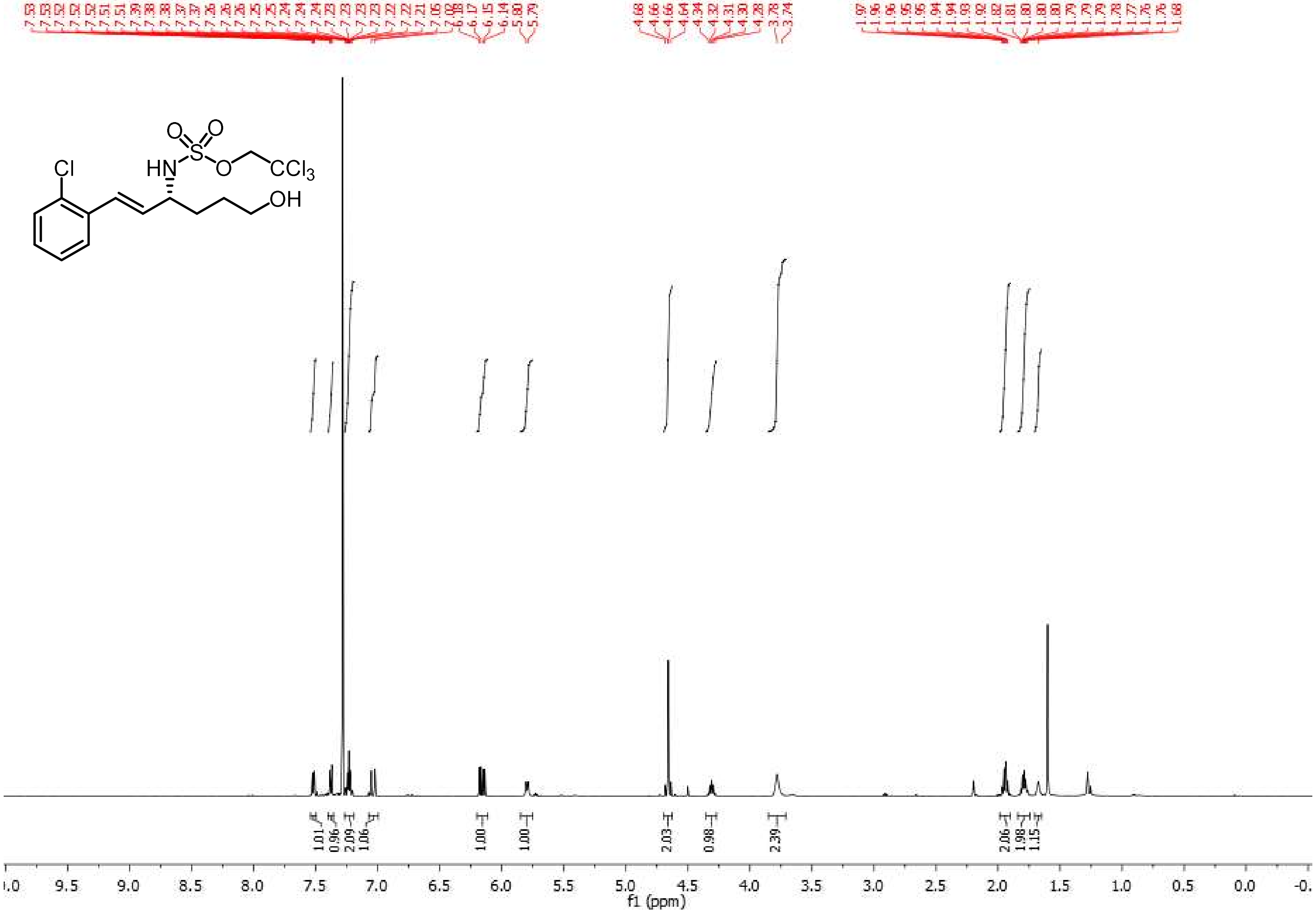

^{13}C NMR (176 MHz, $CDCl_3$) for ***2,2,2-trichloroethyl (R,E)-(1-(2-chlorophenyl)-6-hydroxyhex-1-en-3-yl)sulfamate (2m)***

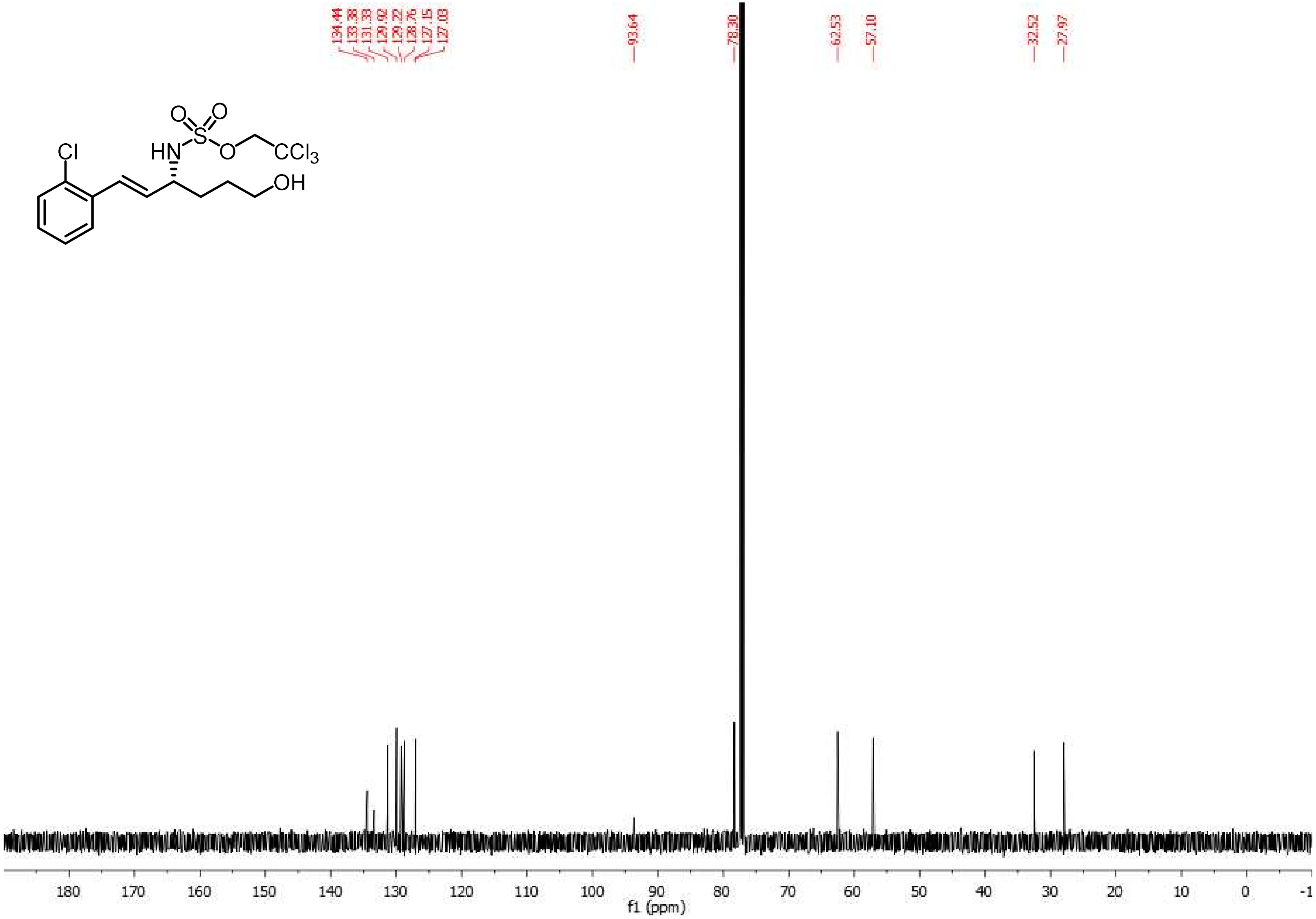

^{1}H NMR (700 MHz, $CDCl_3$) for ***2,2,2-trichloroethyl (R,E)-(1-(2-bromophenyl)-6-hydroxyhex-1-en-3-yl)sulfamate (2n)***

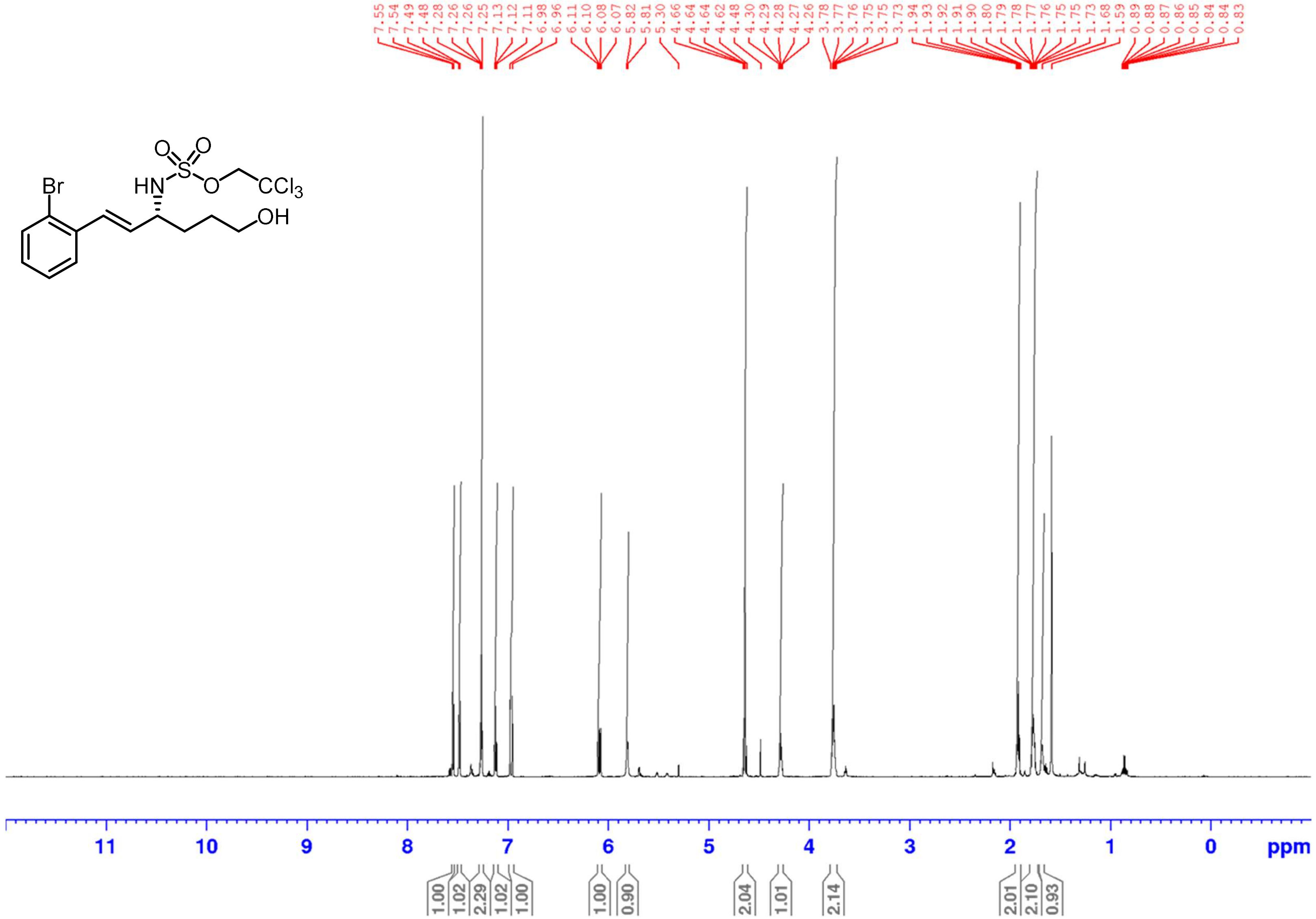

^{13}C NMR (176 MHz, $CDCl_3$) for ***2,2,2-trichloroethyl (R,E)-(1-(2-bromophenyl)-6-hydroxyhex-1-en-3-yl)sulfamate (2n)***

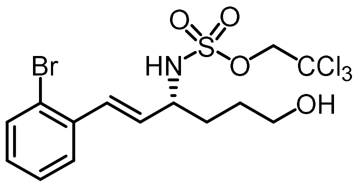

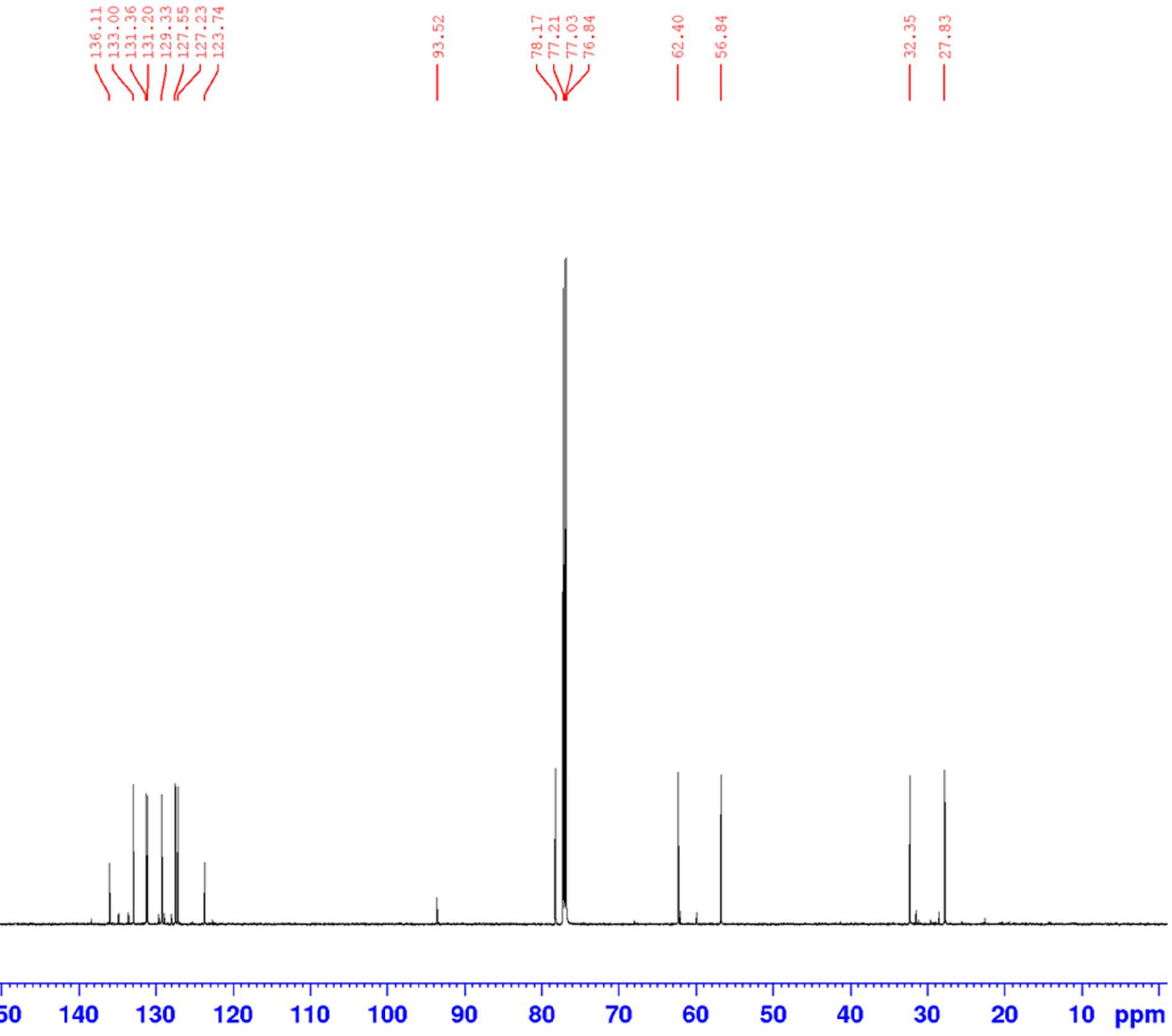

^{1}H NMR (700 MHz, $CDCl_3$) for ***2,2,2-trichloroethyl (R,E)-(1-(2-ethylphenyl)-6-hydroxyhex-1-en-3-yl)sulfamate (2o)***

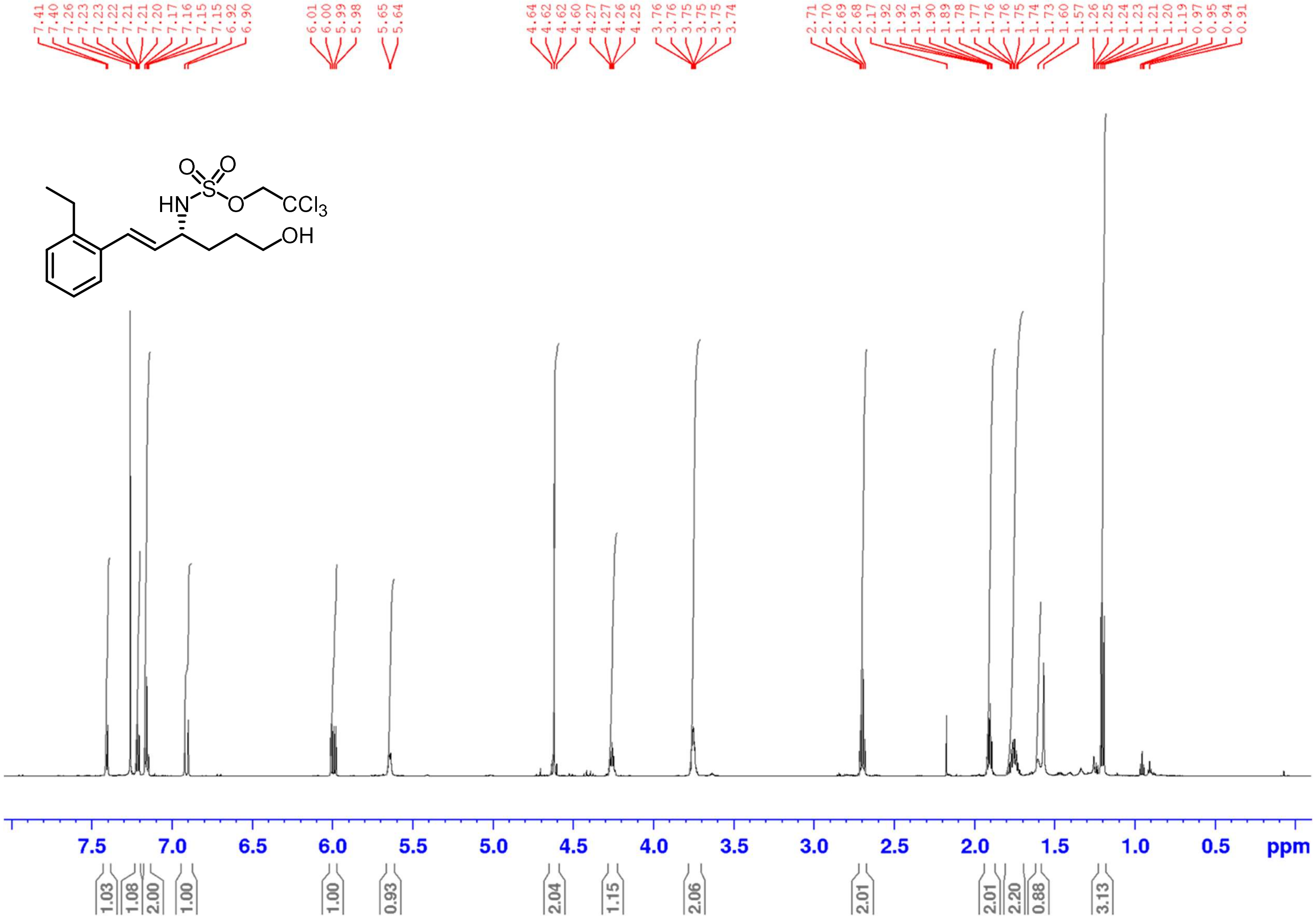

^{13}C NMR (176 MHz, $CDCl_3$) for ***2,2,2-trichloroethyl (R,E)-(1-(2-ethylphenyl)-6-hydroxyhex-1-en-3-yl)sulfamate (2o)***

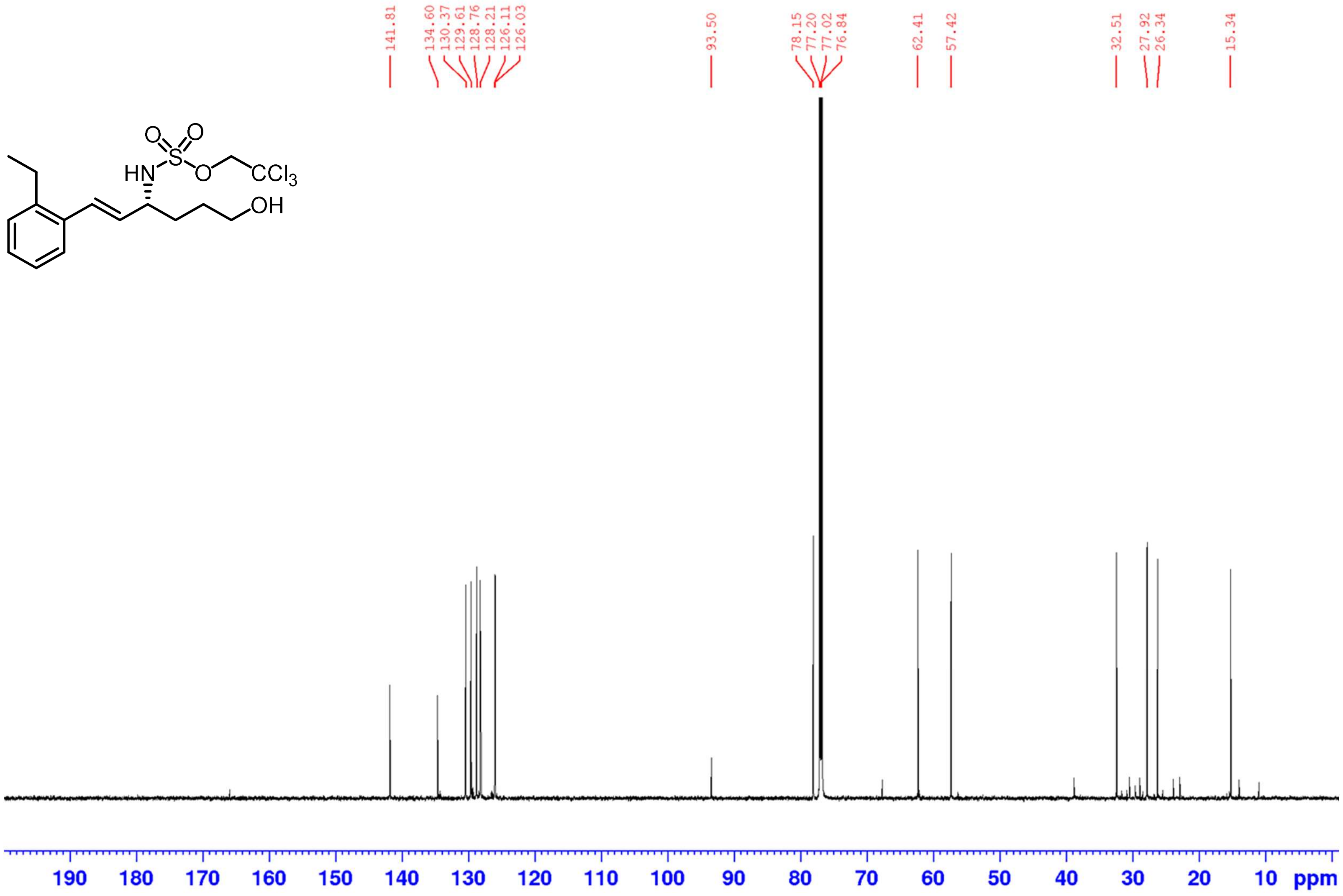

^{1}H NMR (700 MHz, $CDCl_3$) for ***2,2,2-trichloroethyl (R,E)-(6-hydroxy-1-mesitylhex-1-en-3-yl)sulfamate (2p)***

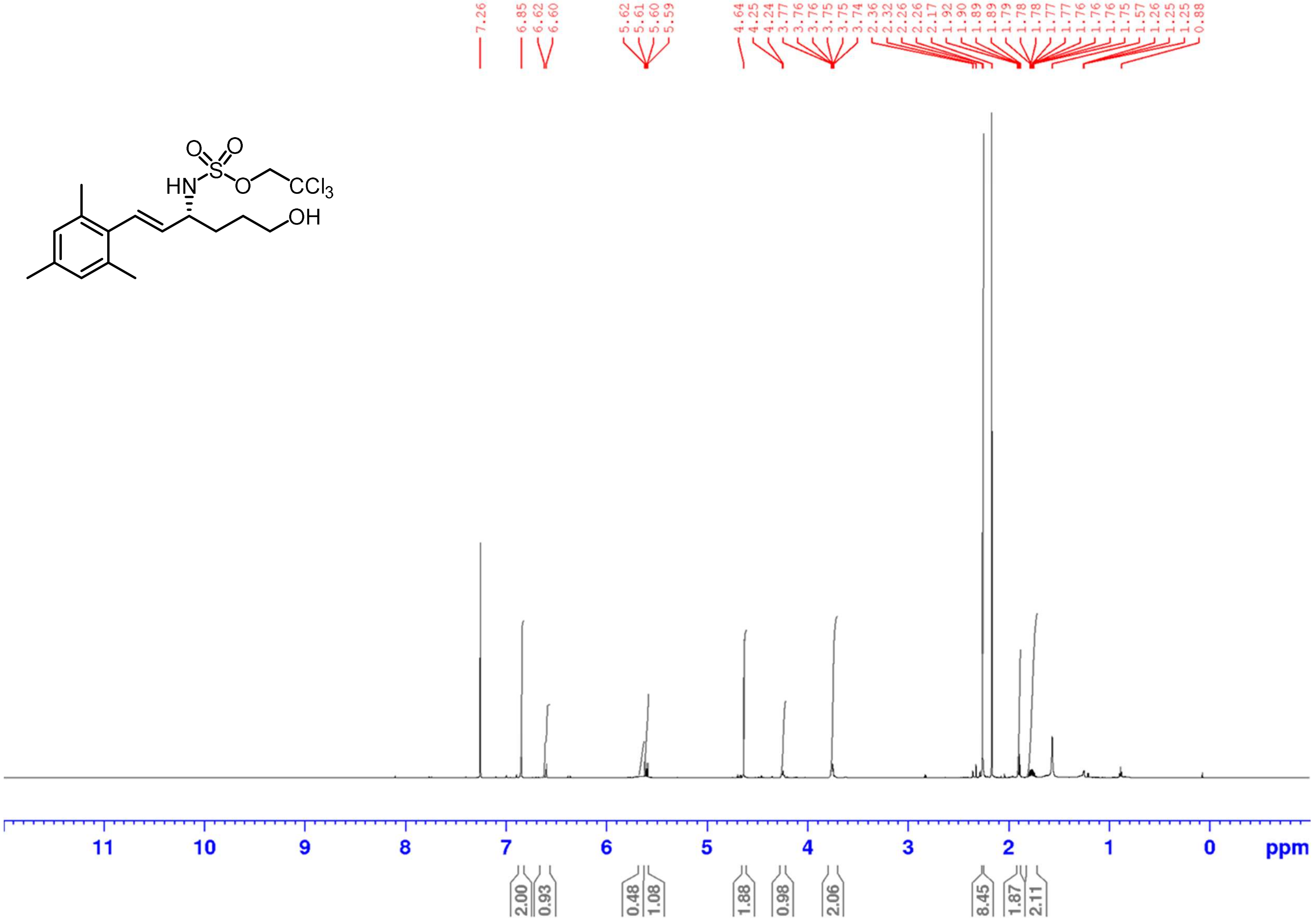

^{13}C NMR (176 MHz, $CDCl_3$) for ***2,2,2-trichloroethyl (R,E)-(6-hydroxy-1-mesitylhex-1-en-3-yl)sulfamate (2p)***

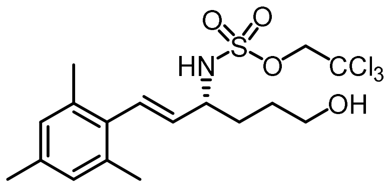

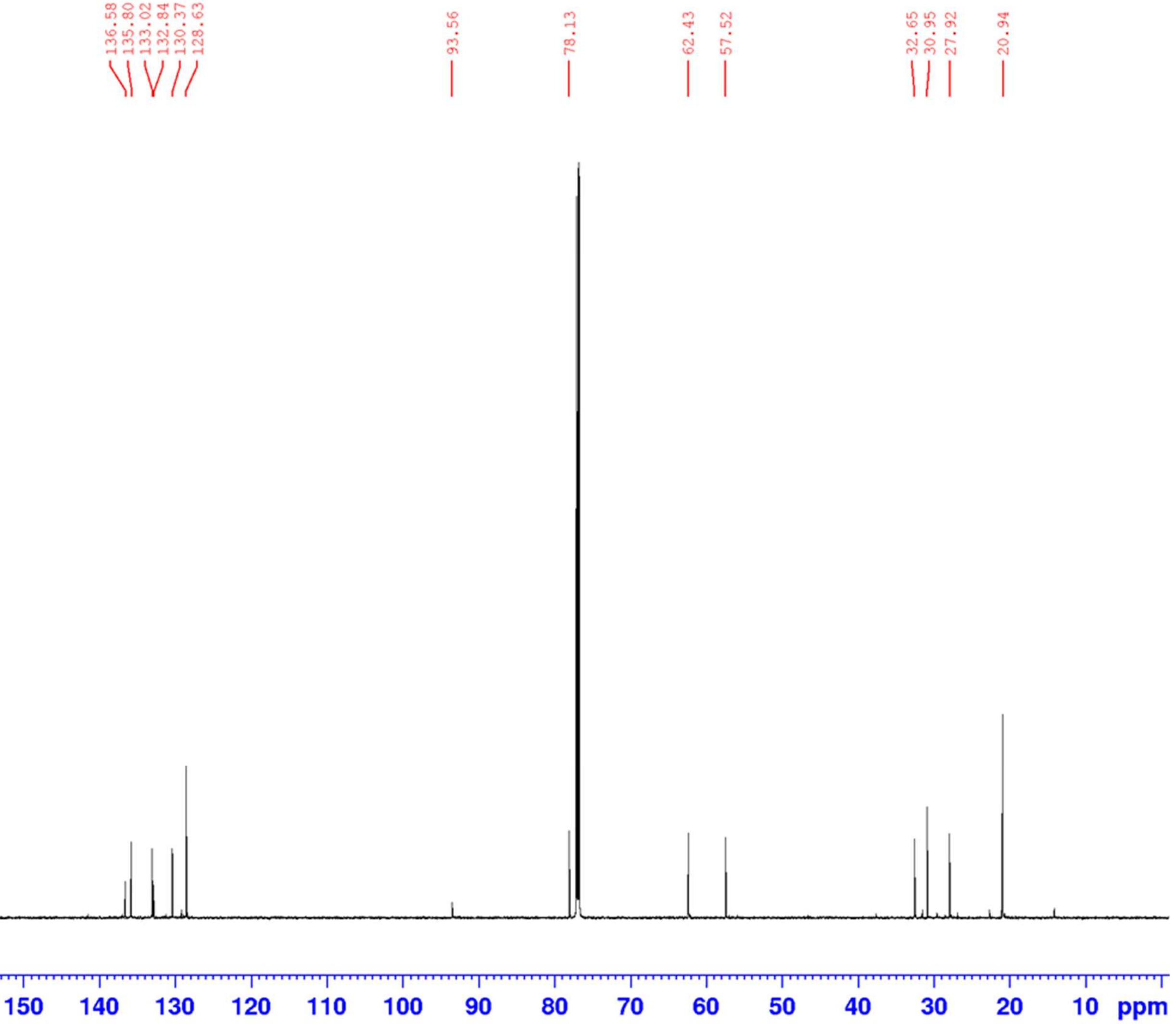

^{1}H NMR (700 MHz, $CDCl_3$) for ***2,2,2-trichloroethyl (R,E)-(6-hydroxy-6-methyl-1-phenylhept-1-en-3-yl)sulfamate (2q)***

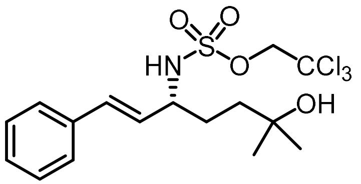

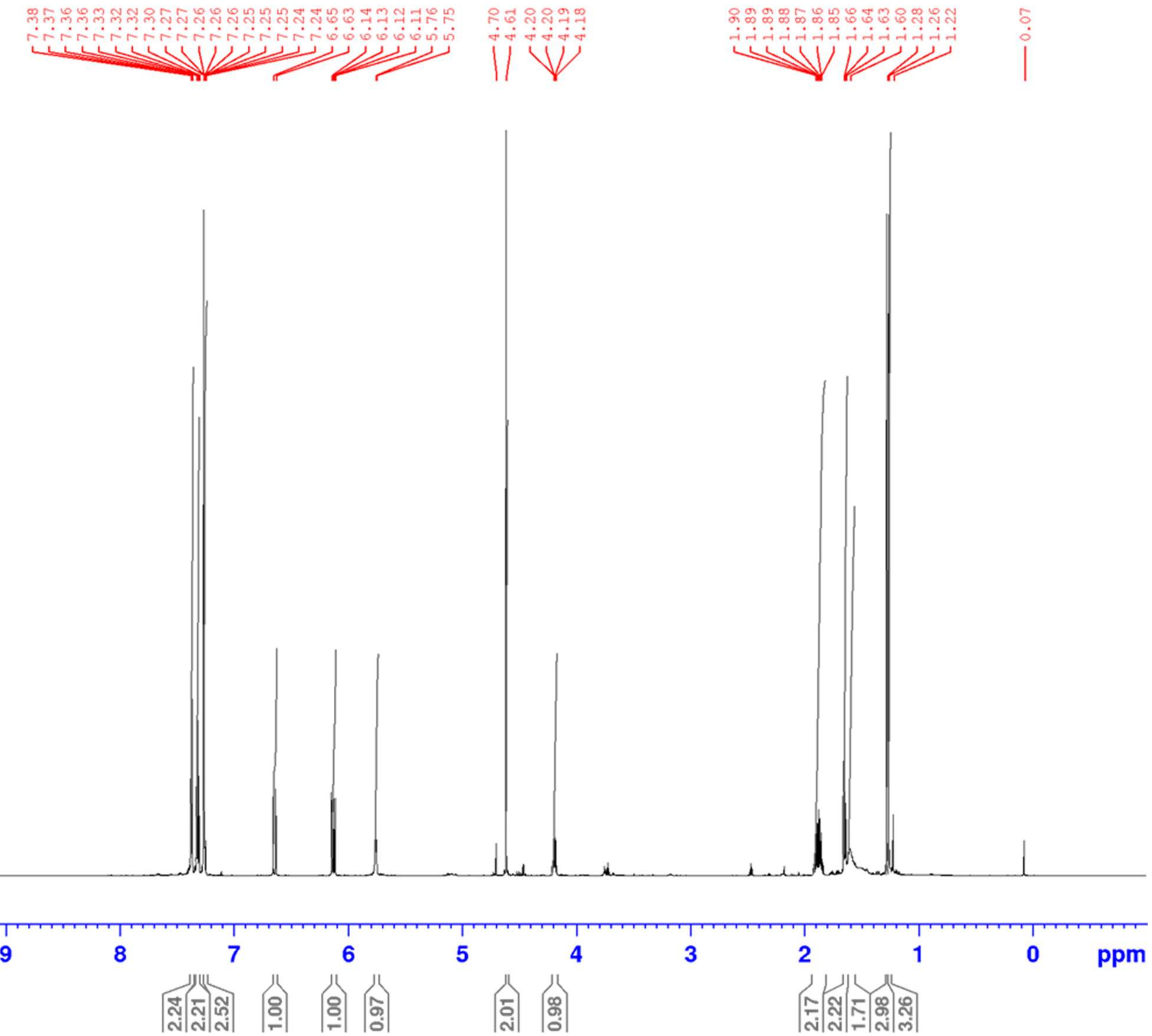

13C NMR (176 MHz, CDCl3) for ***2,2,2-trichloroethyl (R,E)-(6-hydroxy-6-methyl-1-phenylhept-1-en-3-yl)sulfamate (2q)***

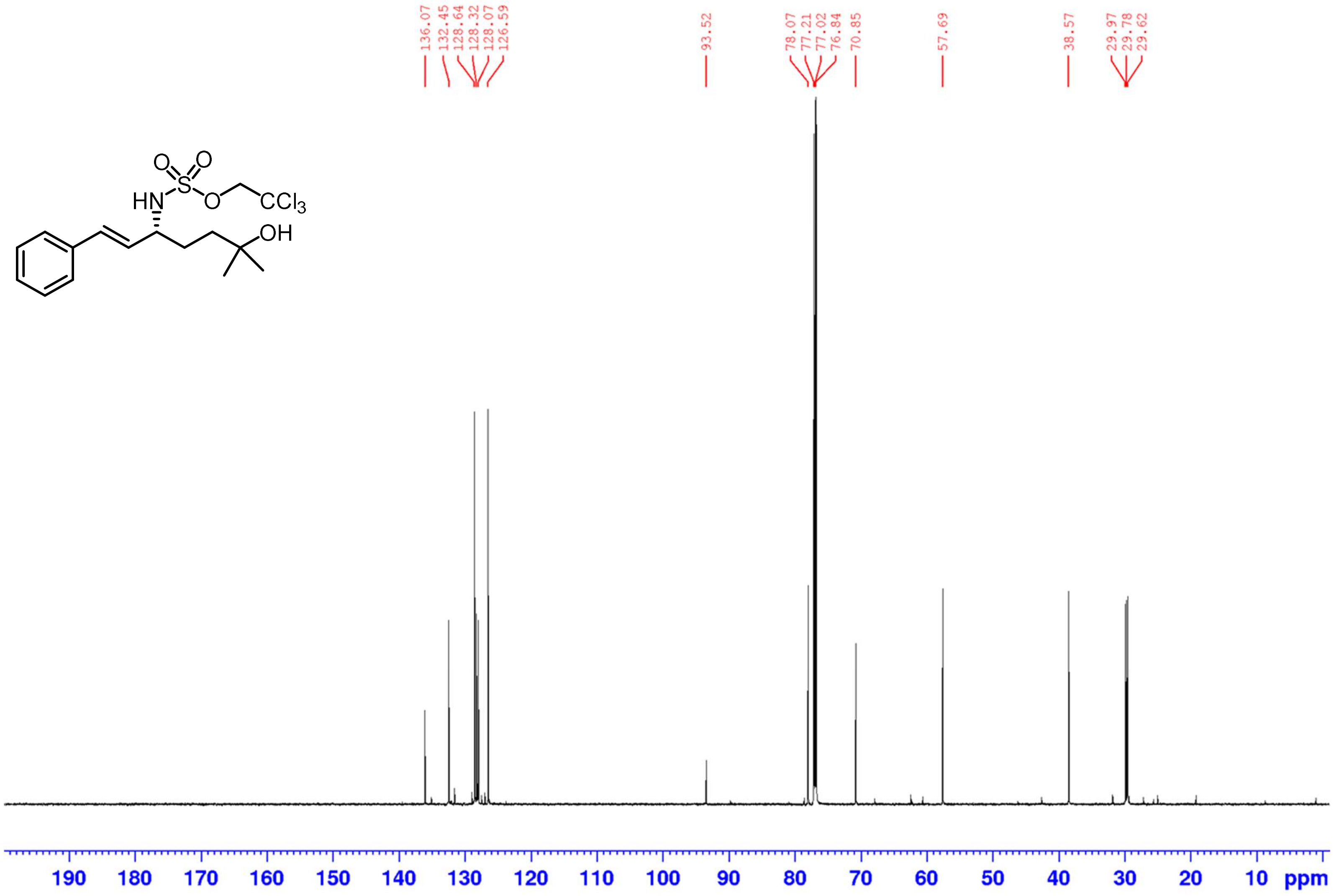

^{1}H NMR (400 MHz, $CDCl_3$) for ***2,2,2-trichloroethyl (R,E)-(1-hydroxydec-5-en-4-yl)sulfamate***

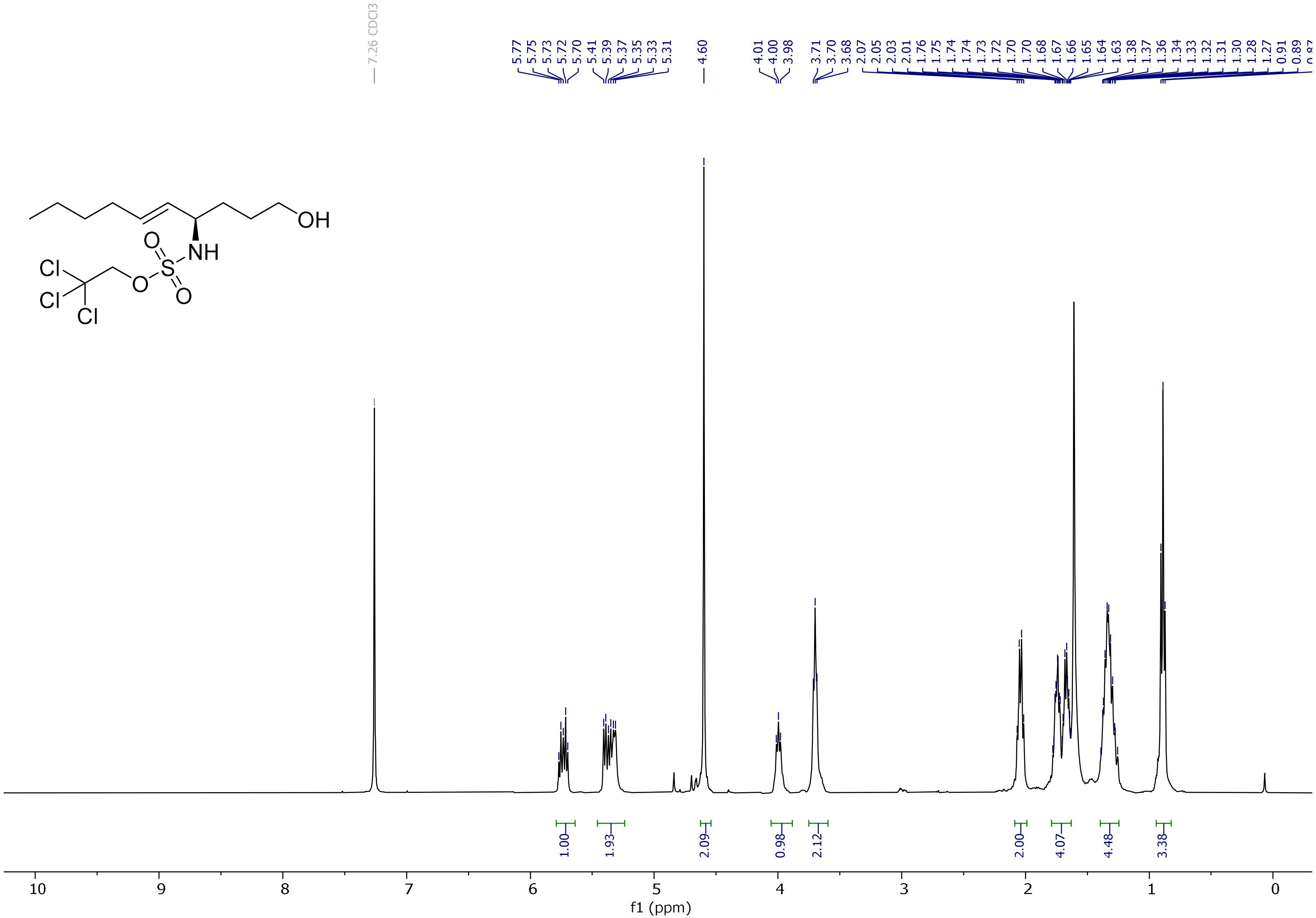

^{13}C NMR (101 MHz, $CDCl_3$) for ***2,2,2-trichloroethyl (R,E)-(1-hydroxydec-5-en-4-yl)sulfamate***

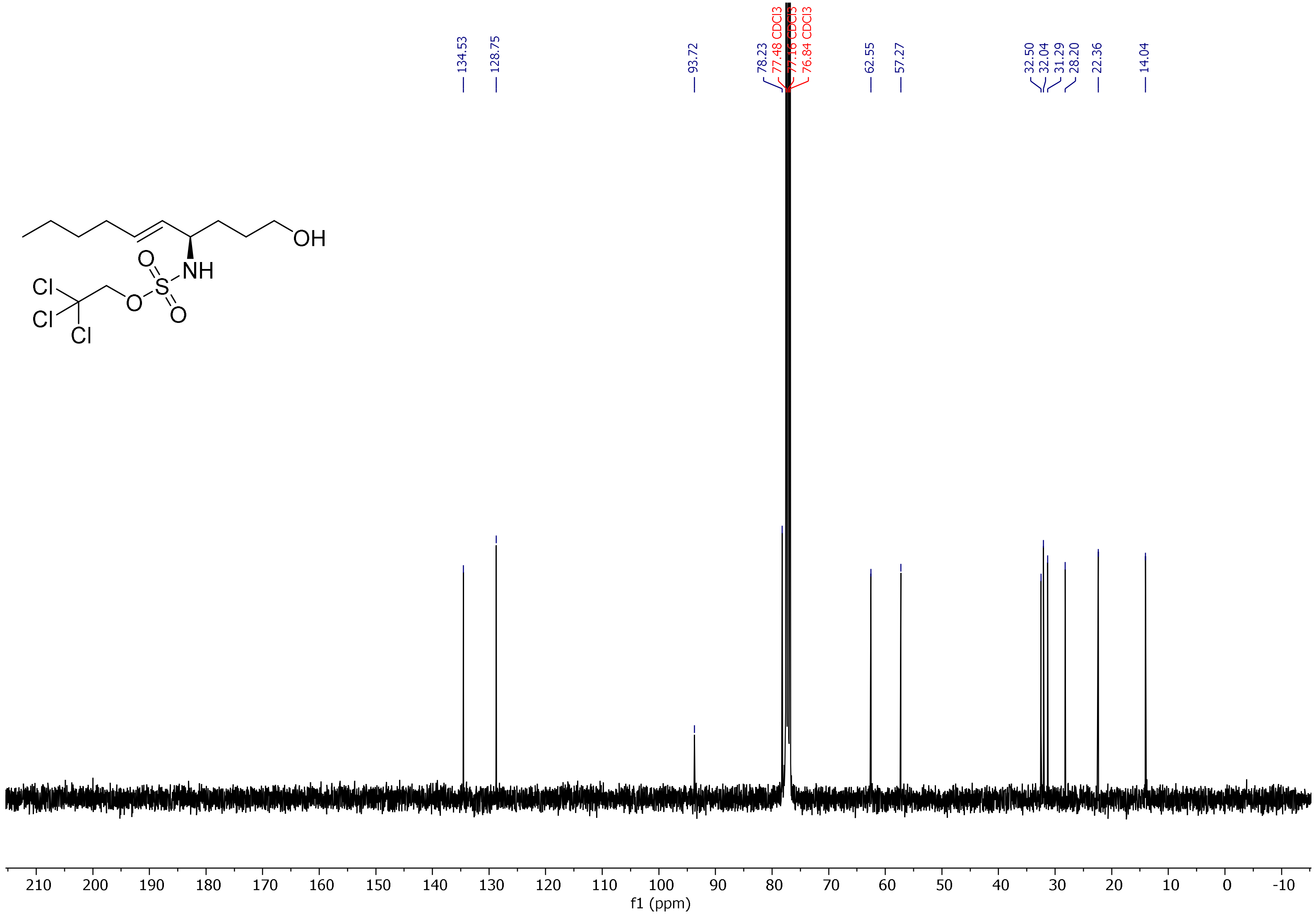

¹H NMR (700 MHz, $CDCl_3$) for ***2,2,2-trichloroethyl (R,E)-(6-methoxy-1-phenylhex-1-en-3-yl)sulfamate (10)***

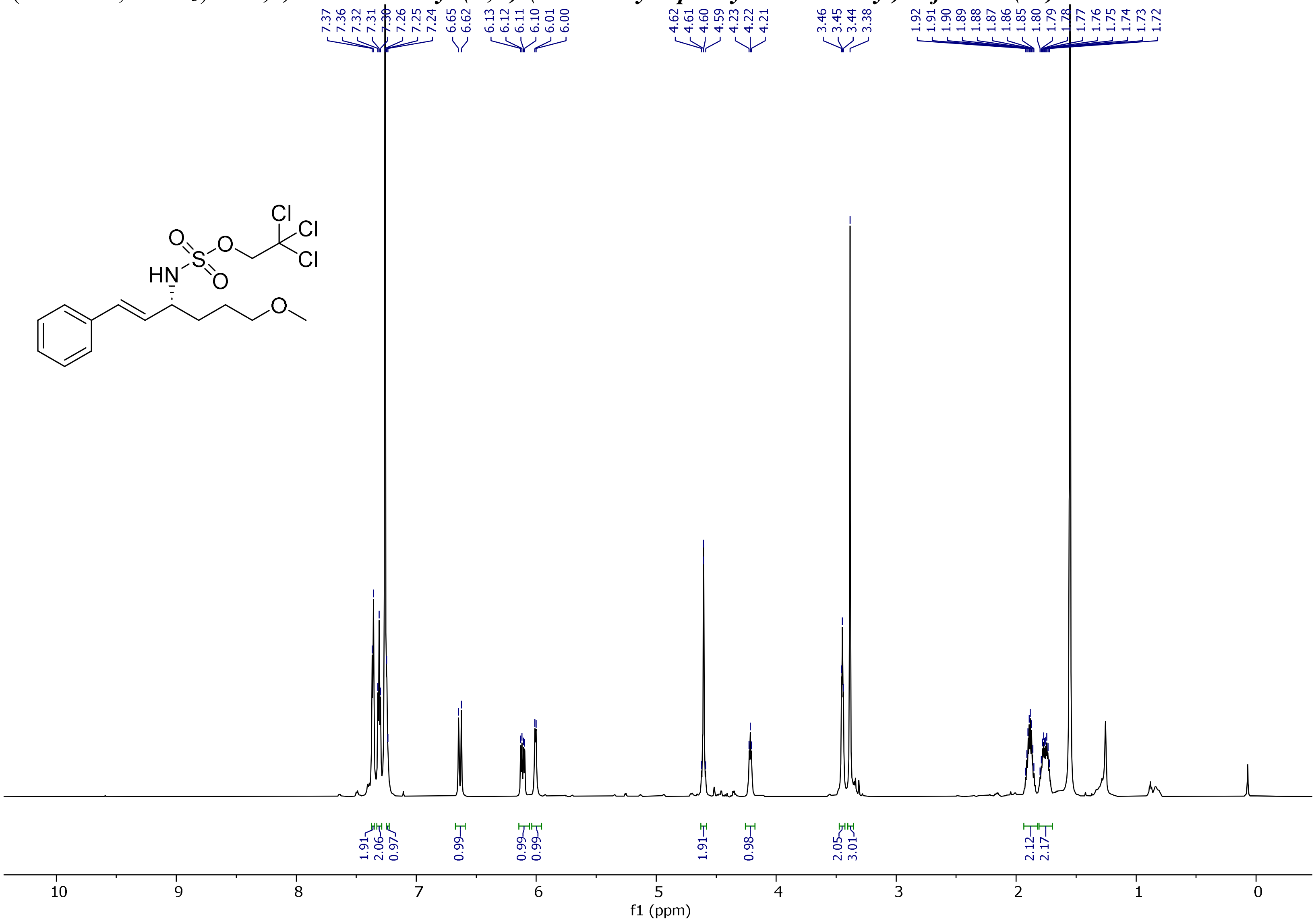

^{13}C NMR (176 MHz, $CDCl_3$) for ***2,2,2-trichloroethyl (R,E)-(6-methoxy-1-phenylhex-1-en-3-yl)sulfamate (10)***

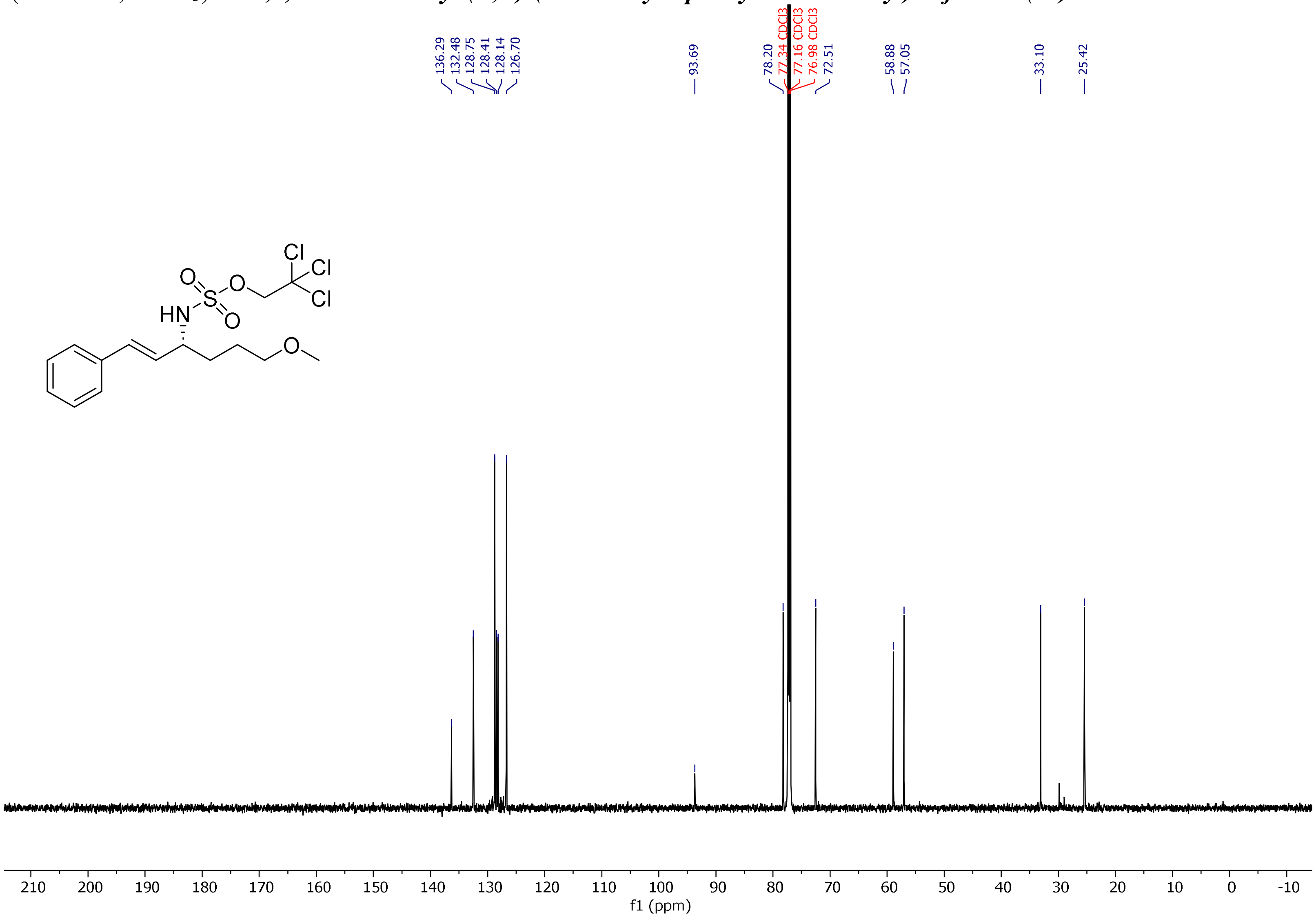

[1]**H NMR** (700 MHz, $CDCl_3$) for ***2,2,2-trichloroethyl (R,E)-(6-oxo-1-phenyl-6-(phenylamino)hex-1-en-3-yl)sulfamate (11)***

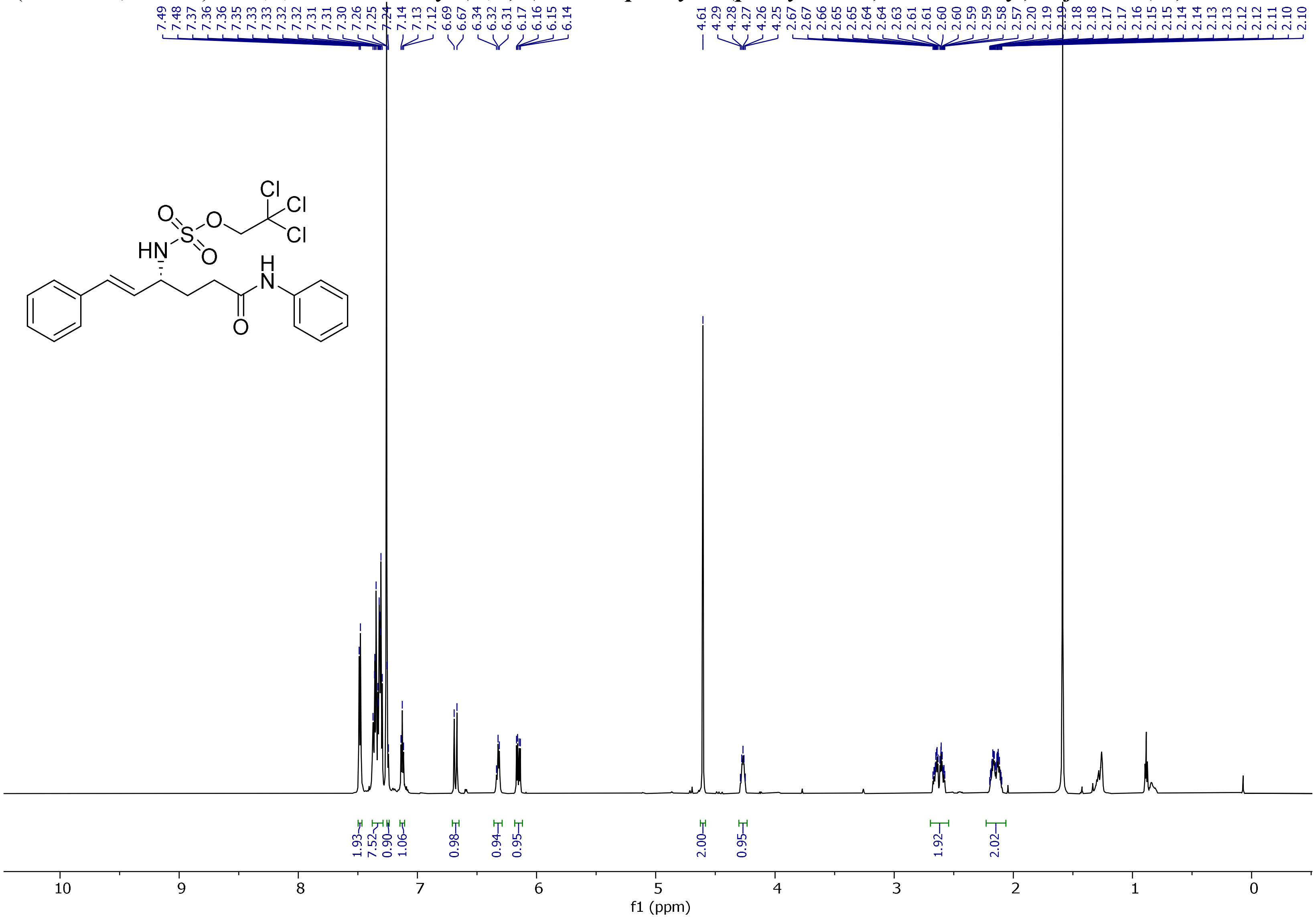

^{13}C NMR (176 MHz, $CDCl_3$) for ***2,2,2-trichloroethyl (R,E)-(6-oxo-1-phenyl-6-(phenylamino)hex-1-en-3-yl)sulfamate (11)***

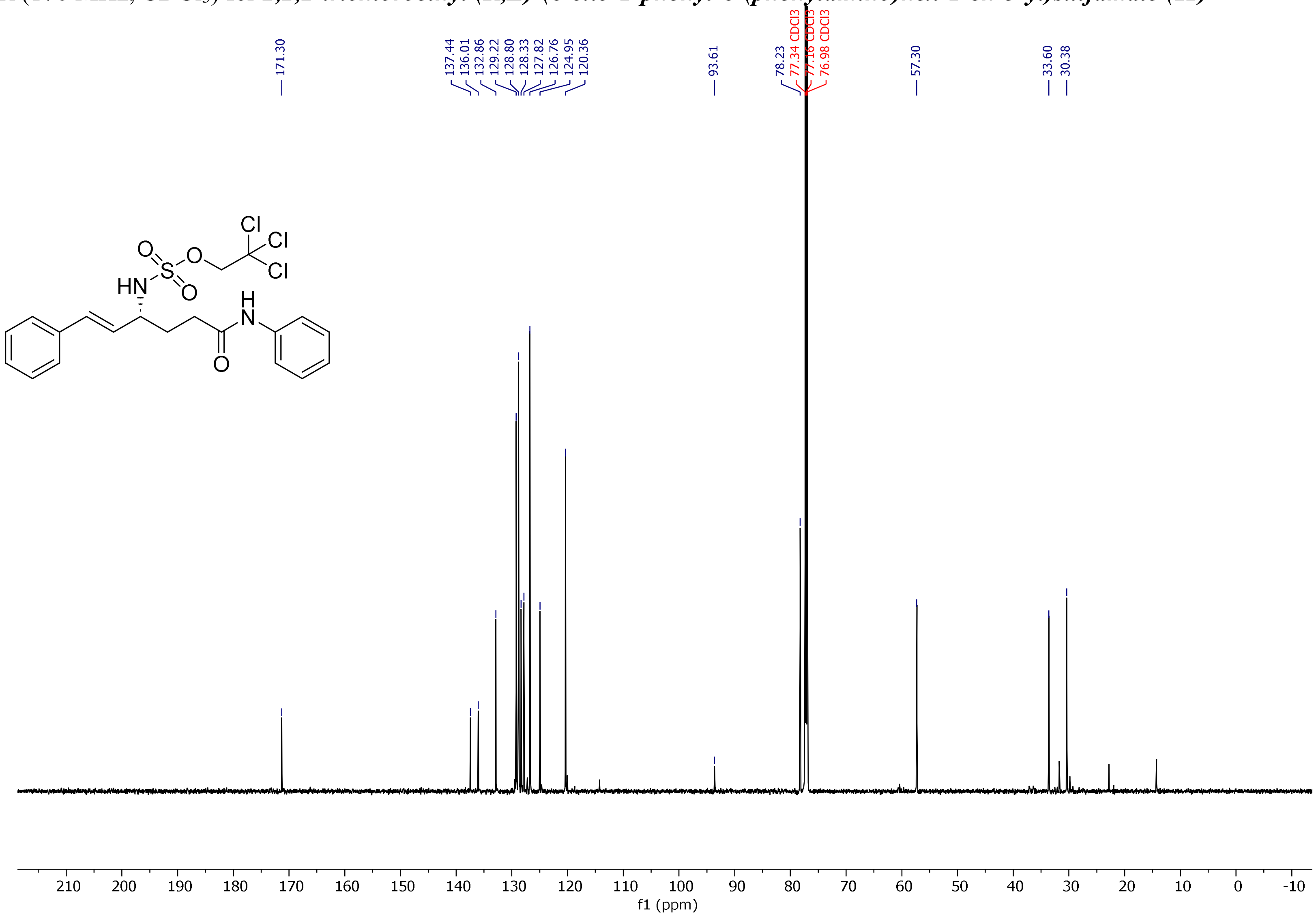

^{1}H NMR (700 MHz, $CDCl_3$) for ***2,2,2-trichloroethyl (R,E)-(5-hydroxy-1-phenylpent-1-en-3-yl)sulfamate (6a)***

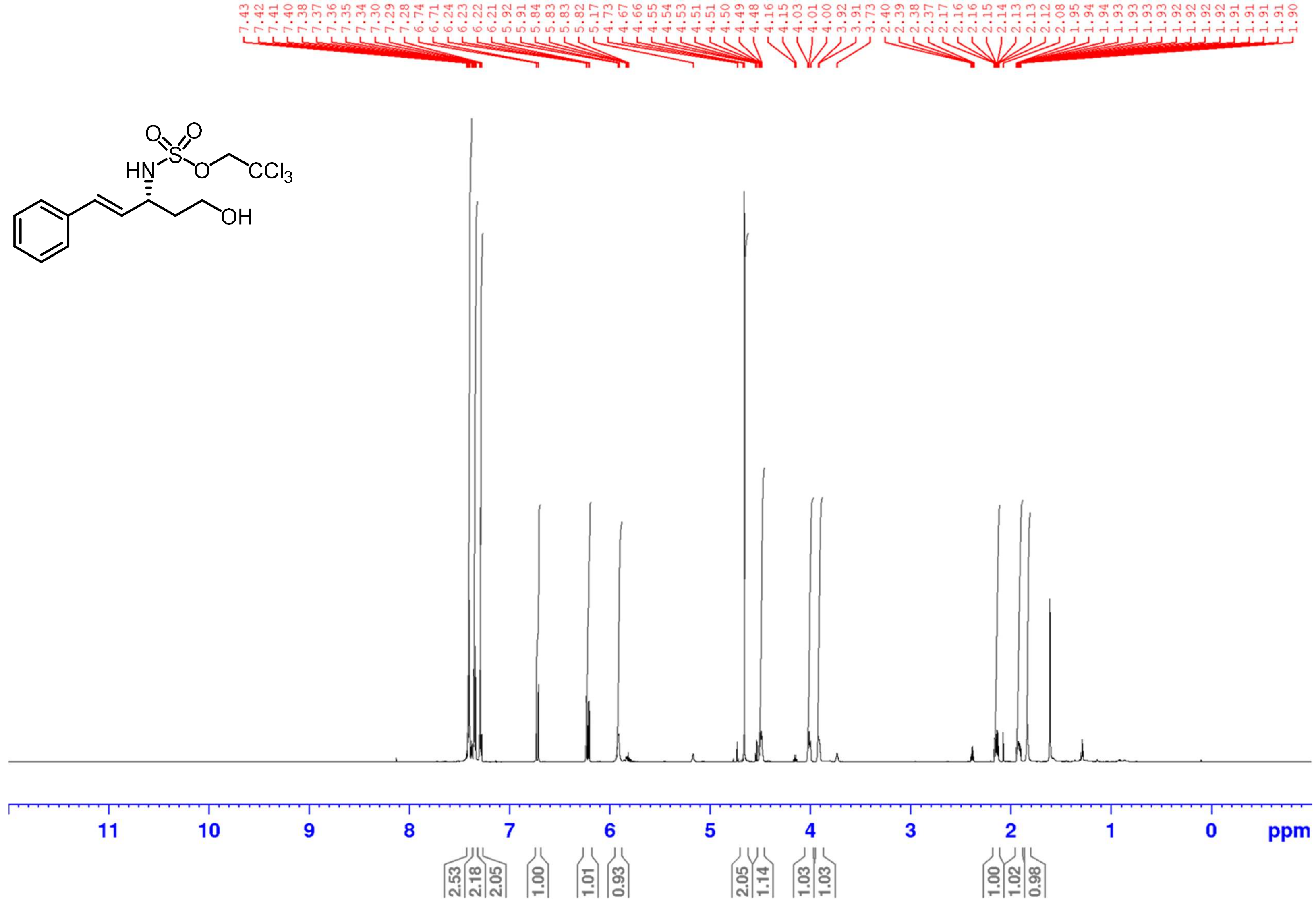

^{13}C NMR (176 MHz, CDCl3) for ***2,2,2-trichloroethyl (R,E)-(5-hydroxy-1-phenylpent-1-en-3-yl)sulfamate (6a)***

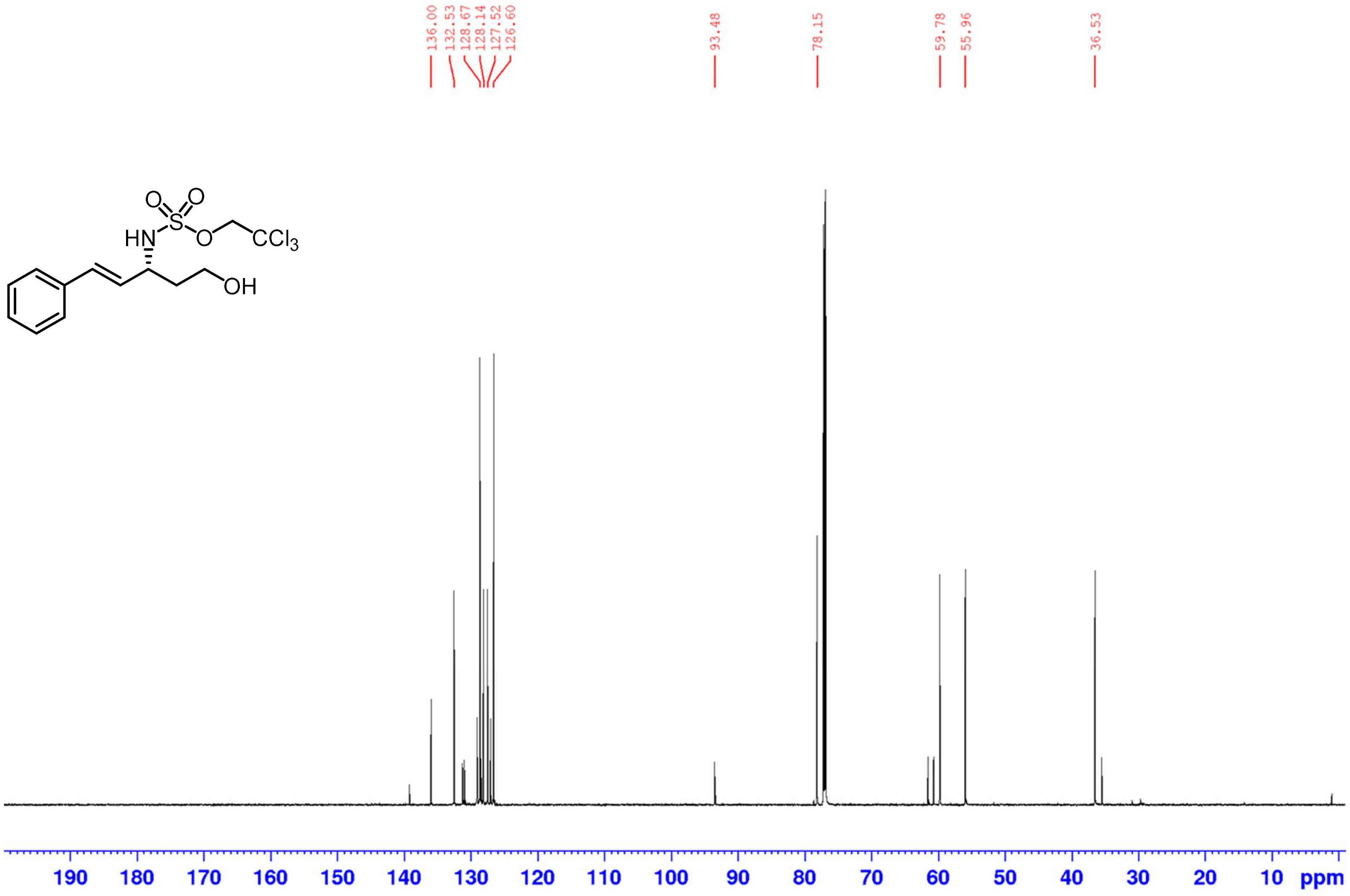

¹H NMR (700 MHz, $CDCl_3$) for ***2,2,2-trichloroethyl (R,E)-(5-hydroxy-1-(2-isopropylphenyl)pent-1-en-3-yl)sulfamate (6b)***

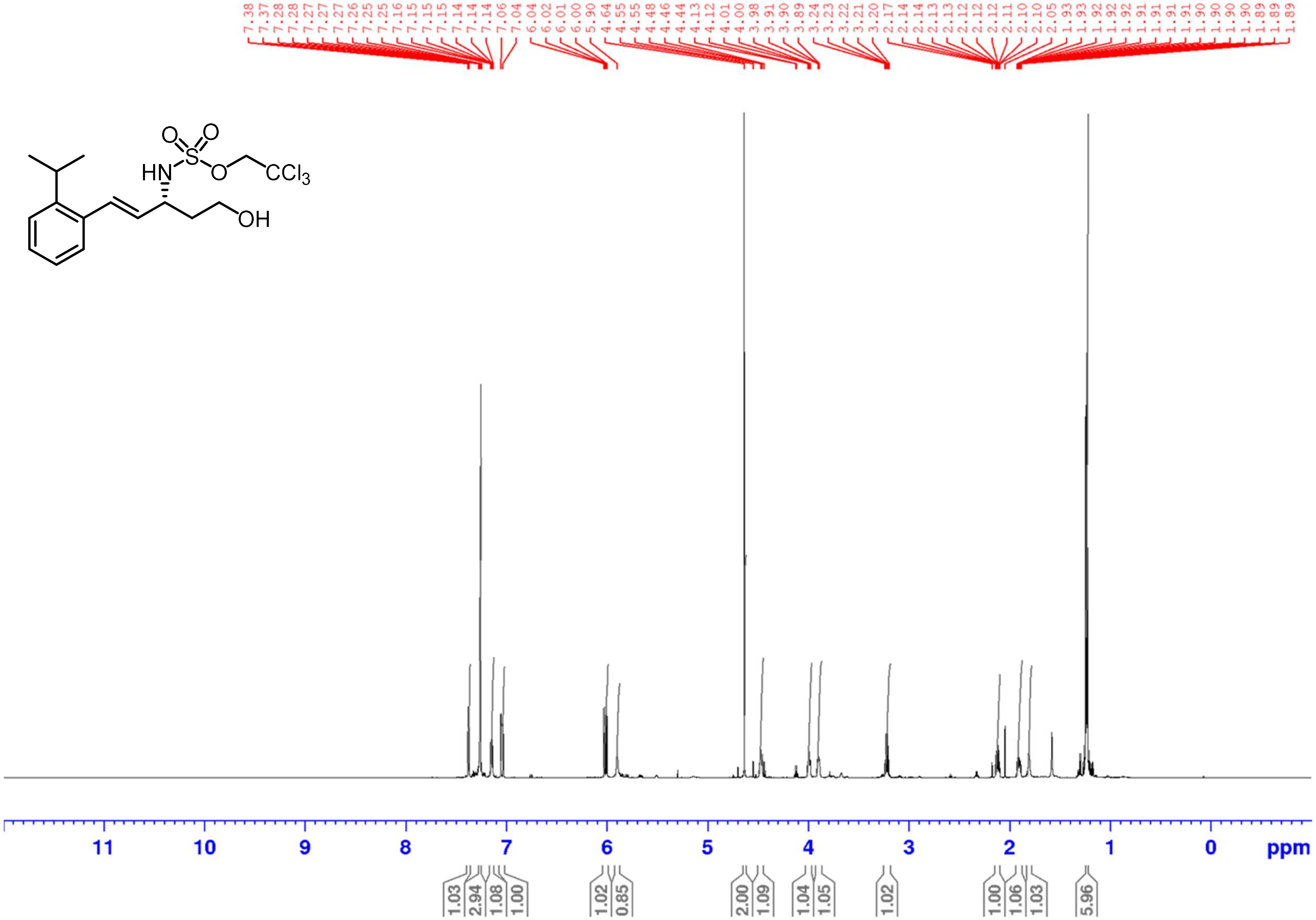

^{13}C NMR (176 MHz, $CDCl_3$) for ***2,2,2-trichloroethyl (R,E)-(5-hydroxy-1-(2-isopropylphenyl)pent-1-en-3-yl)sulfamate (6b)***

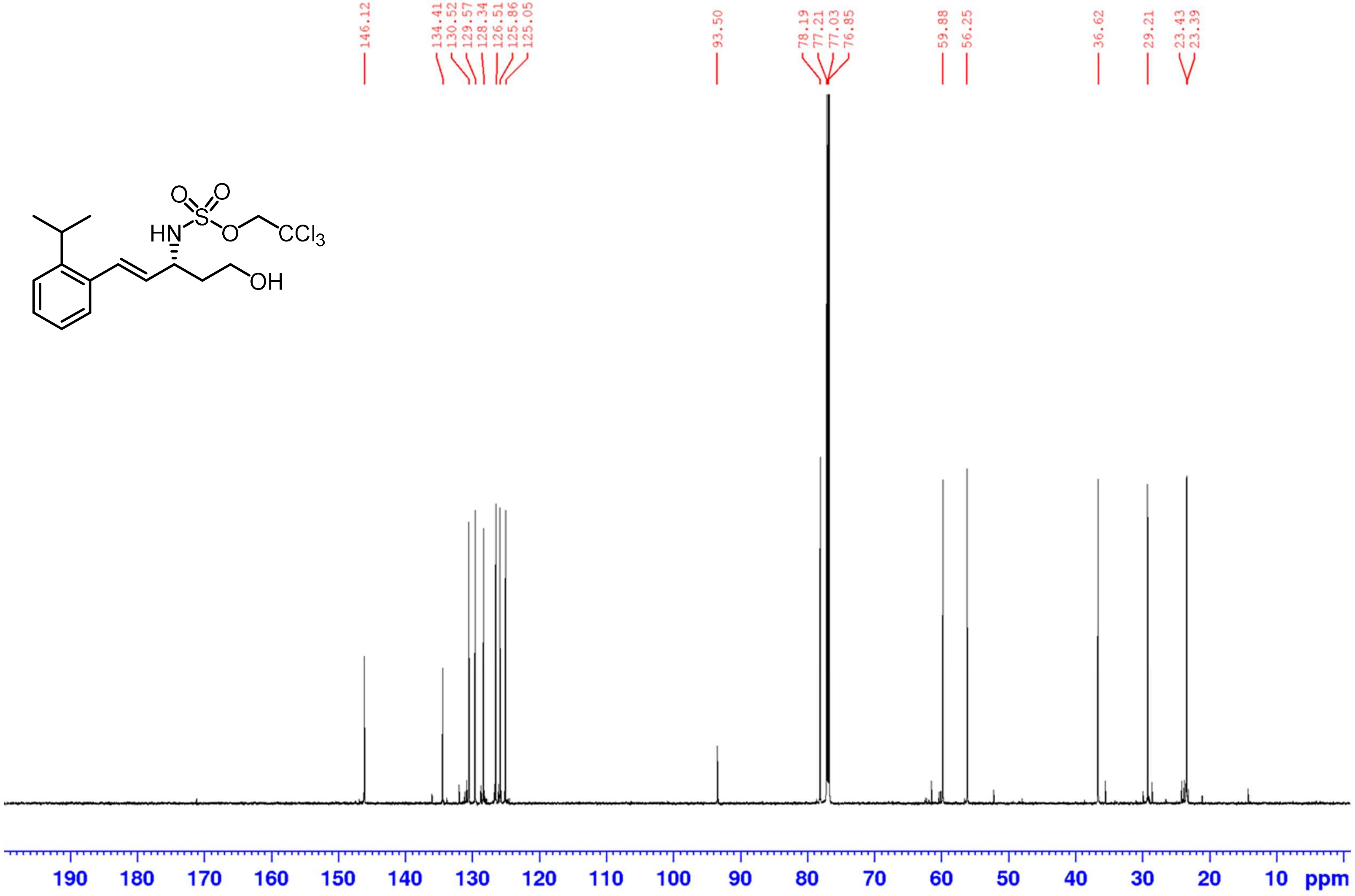

^{1}H NMR (700 MHz, $CDCl_3$) for ***2,2,2-trichloroethyl (R,E)-(5-hydroxy-1-(3-methoxyphenyl)pent-1-en-3-yl)sulfamate (6c)***

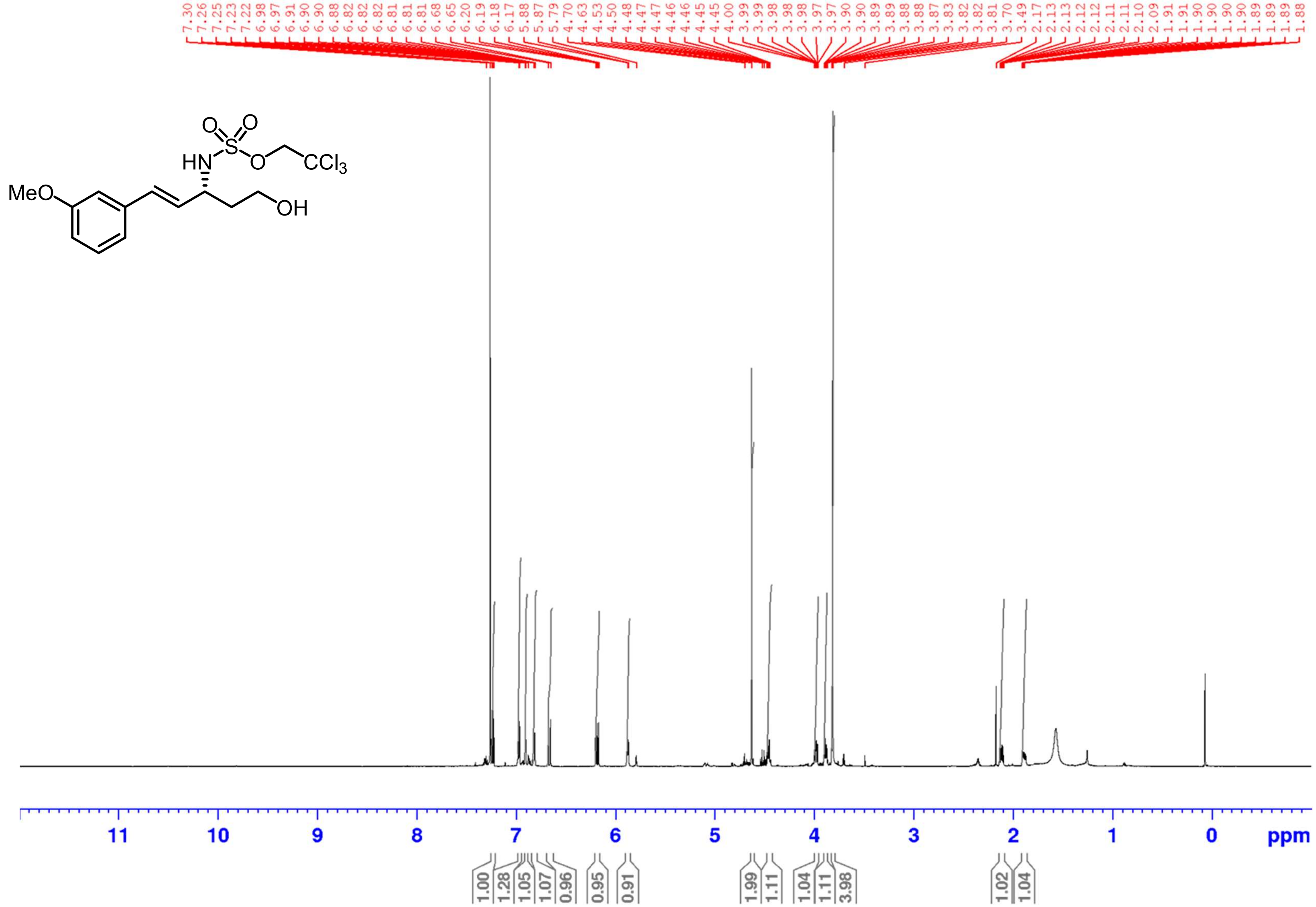

^{13}C NMR (176 MHz, $CDCl_3$) for ***2,2,2-trichloroethyl (R,E)-(5-hydroxy-1-(3-methoxyphenyl)pent-1-en-3-yl)sulfamate (6c)***

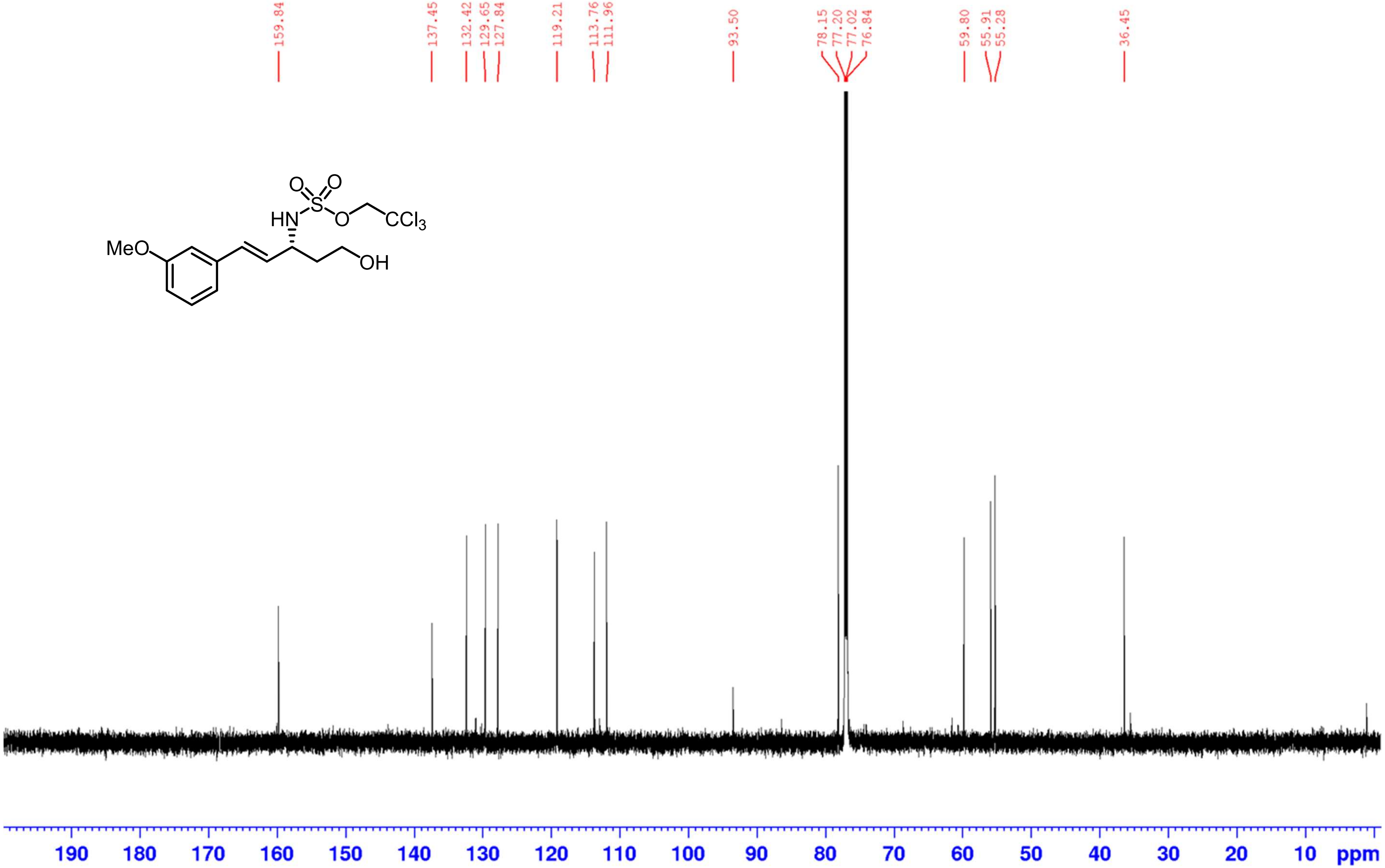

^{1}H NMR (700 MHz, $CDCl_3$) for ***2,2,2-trichloroethyl (R,E)-(1-(3,5-di-tert-butylphenyl)-5-hydroxypent-1-en-3-yl)sulfamate (6d)***

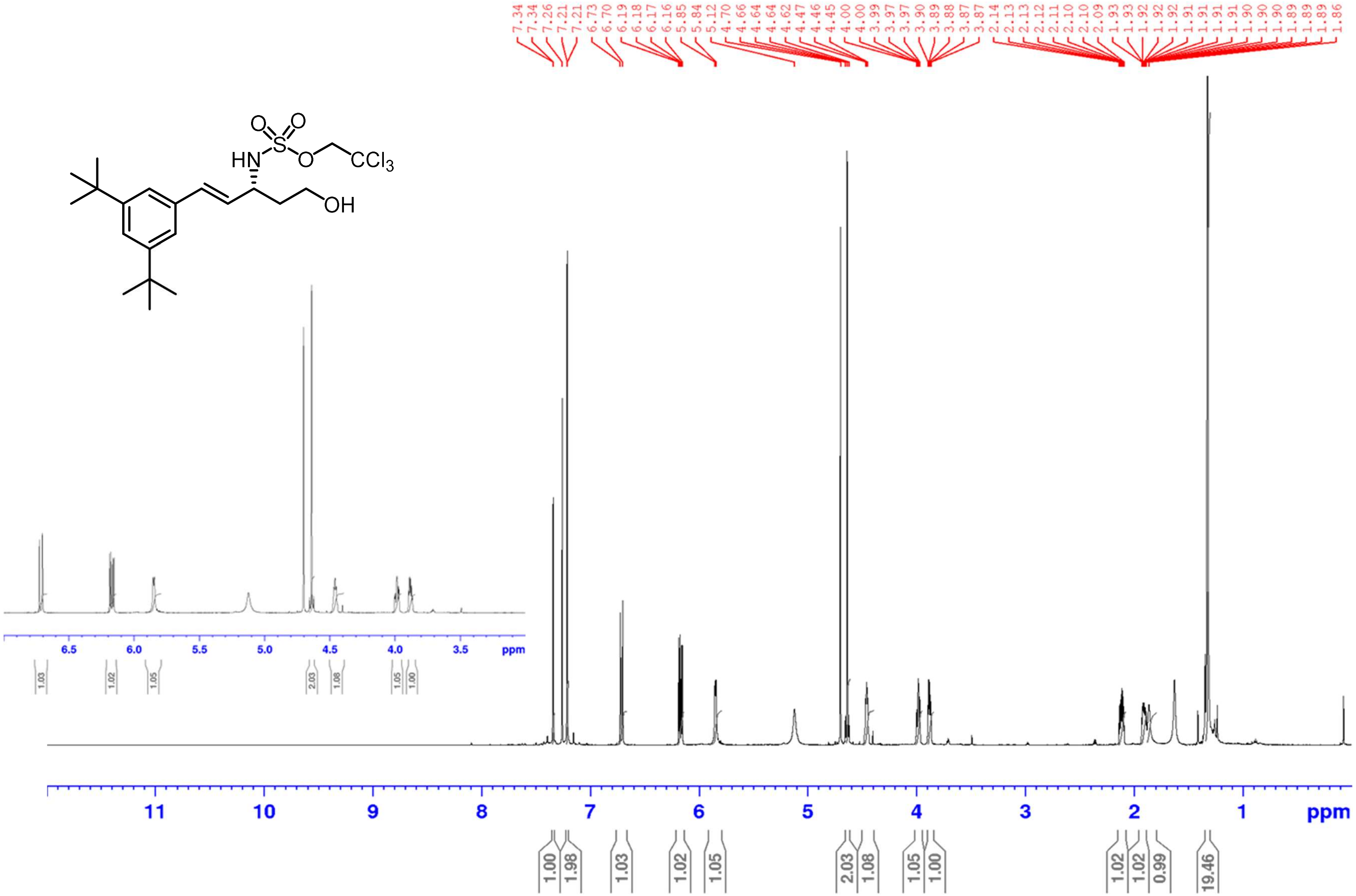

^{13}C NMR (176 MHz, $CDCl_3$) for ***2,2,2-trichloroethyl (R,E)-(1-(3,5-di-tert-butylphenyl)-5-hydroxypent-1-en-3-yl)sulfamate (6d)***

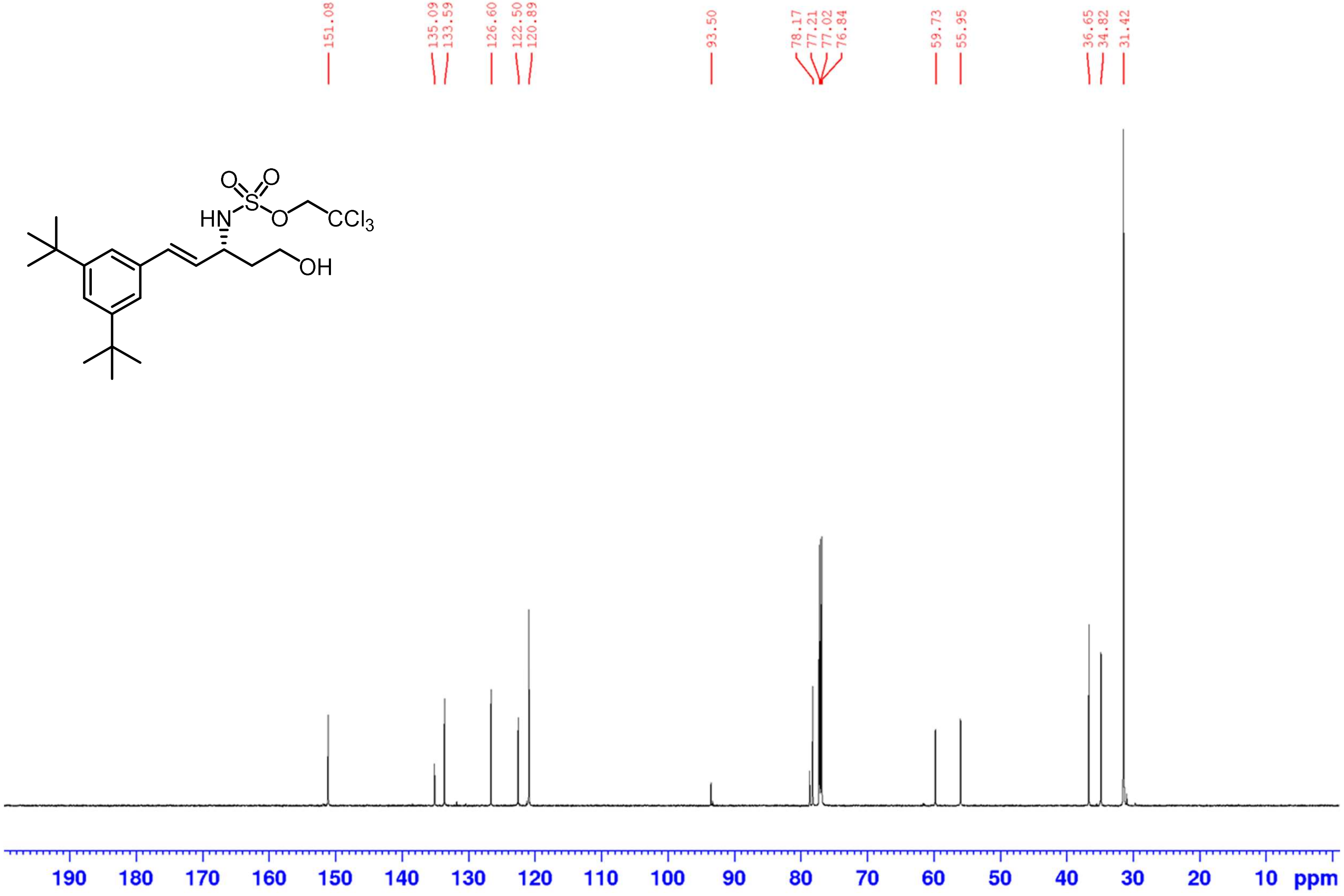

^{1}H NMR (700 MHz, $CDCl_3$) for ***Methyl (R,E)-2-(5-hydroxy-3-(((2,2,2-trichloroethoxy)sulfonyl)amino)pent-1-en-1-yl)benzoate (6e)***

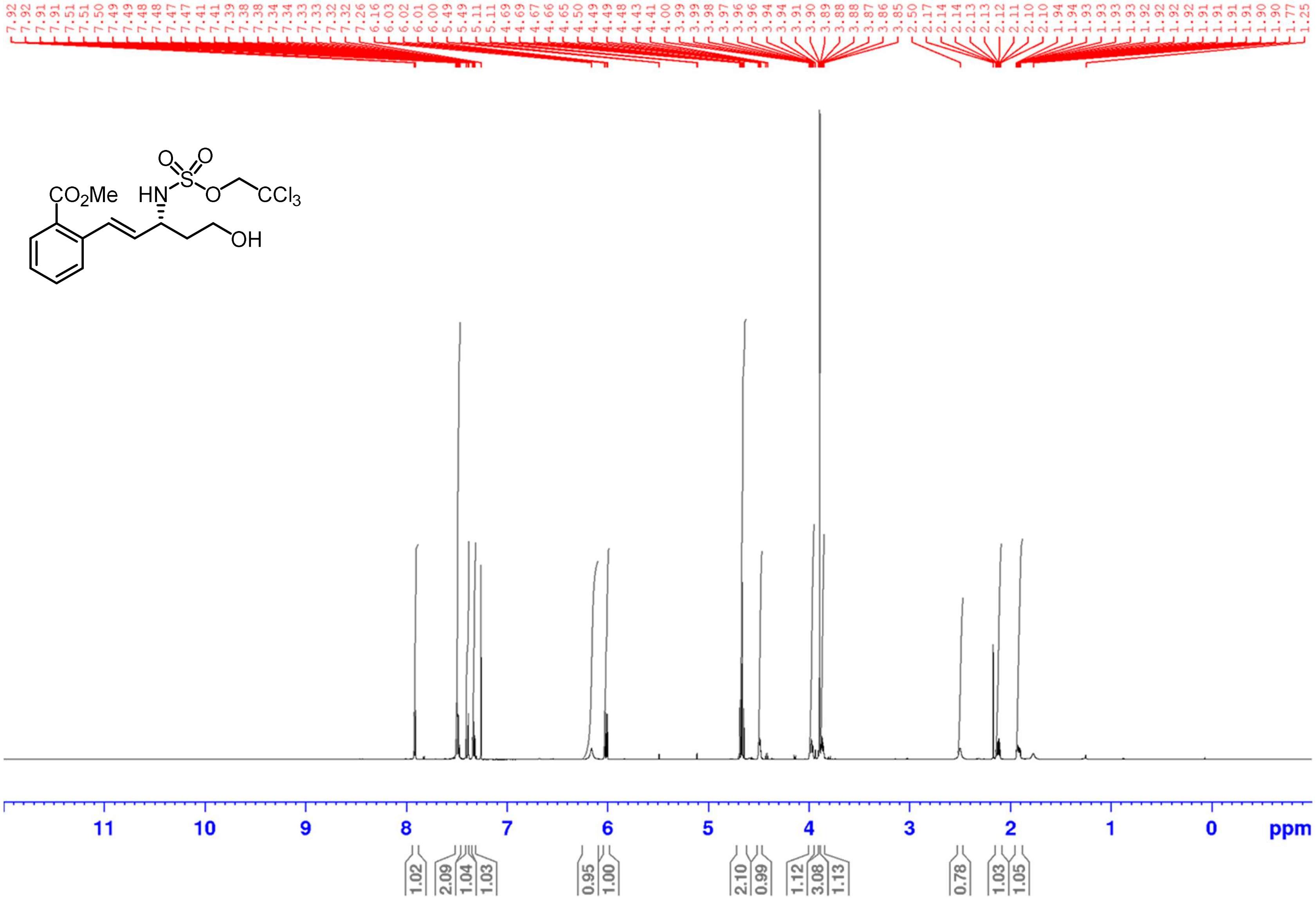

^{13}C NMR (176 MHz, $CDCl_3$) for ***Methyl (R,E)-2-(5-hydroxy-3-(((2,2,2-trichloroethoxy)sulfonyl)amino)pent-1-en-1-yl)benzoate (6e)***

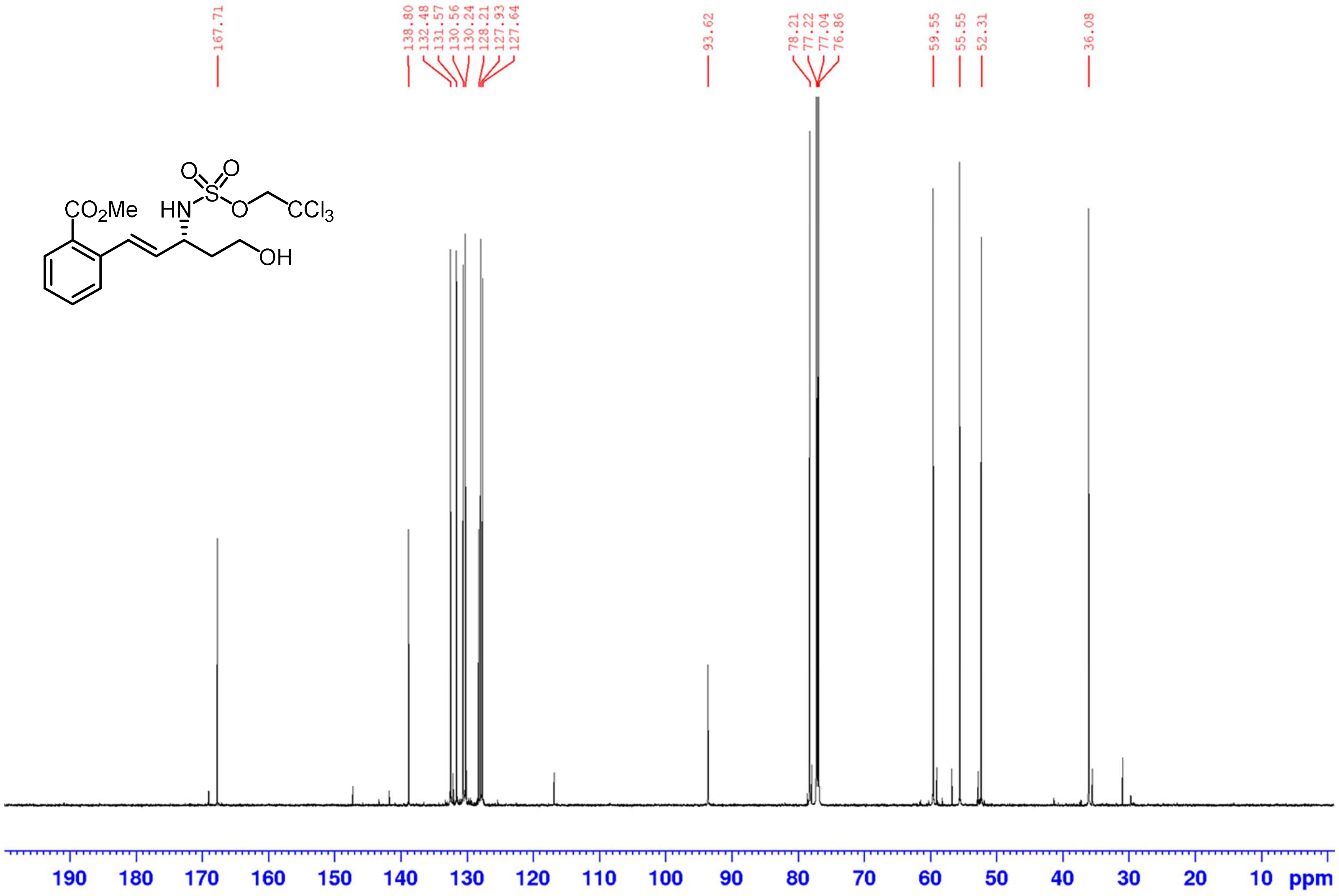

^{1}H NMR (700 MHz, $CDCl_3$) for ***2,2,2-trichloroethyl (R,E)-(5-hydroxy-5-methyl-1-phenylhex-1-en-3-yl)sulfamate (6f)***

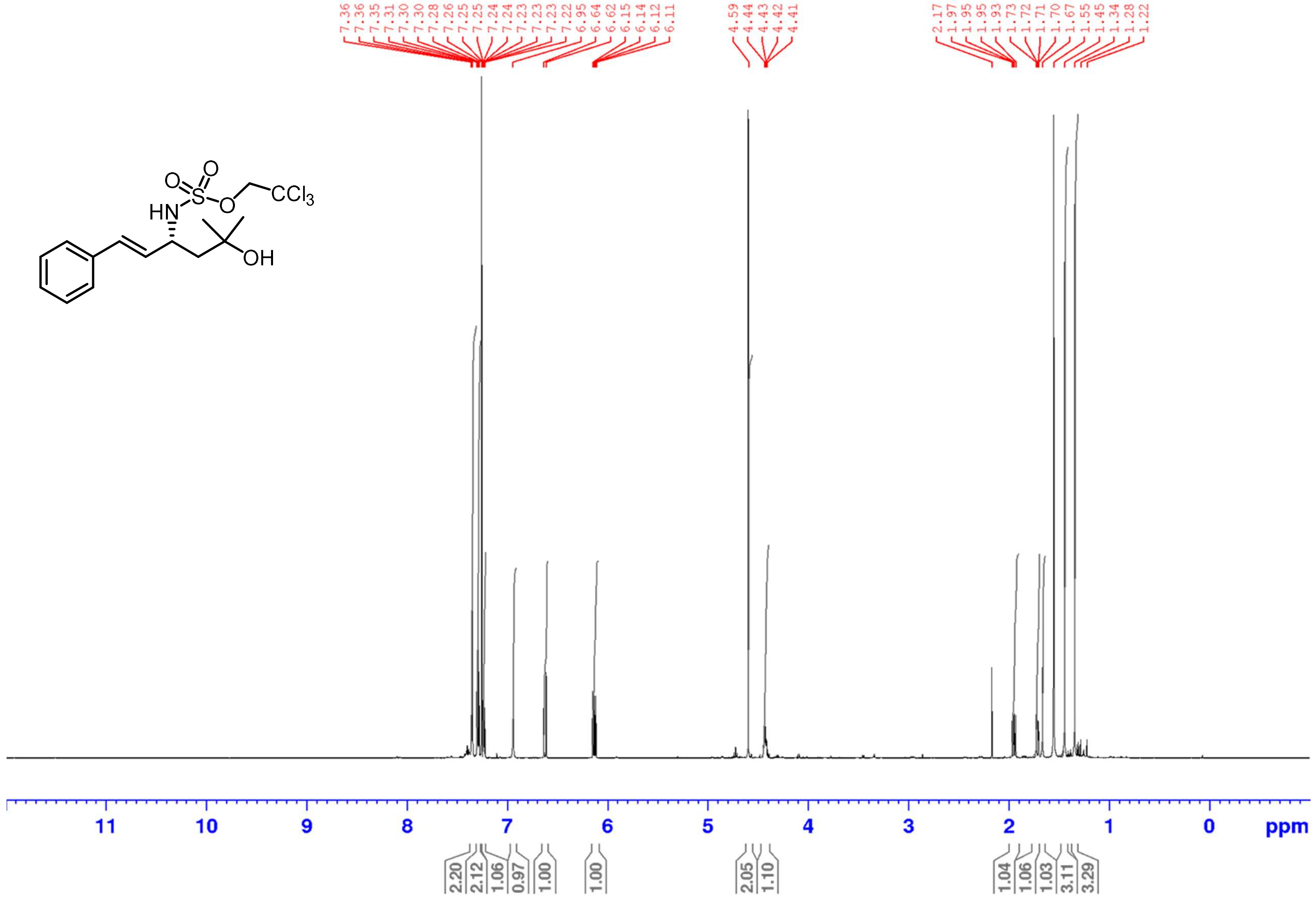

^{13}C **NMR** (176 MHz, CDCl3) for ***2,2,2-trichloroethyl (R,E)-(5-hydroxy-5-methyl-1-phenylhex-1-en-3-yl)sulfamate (6f)***

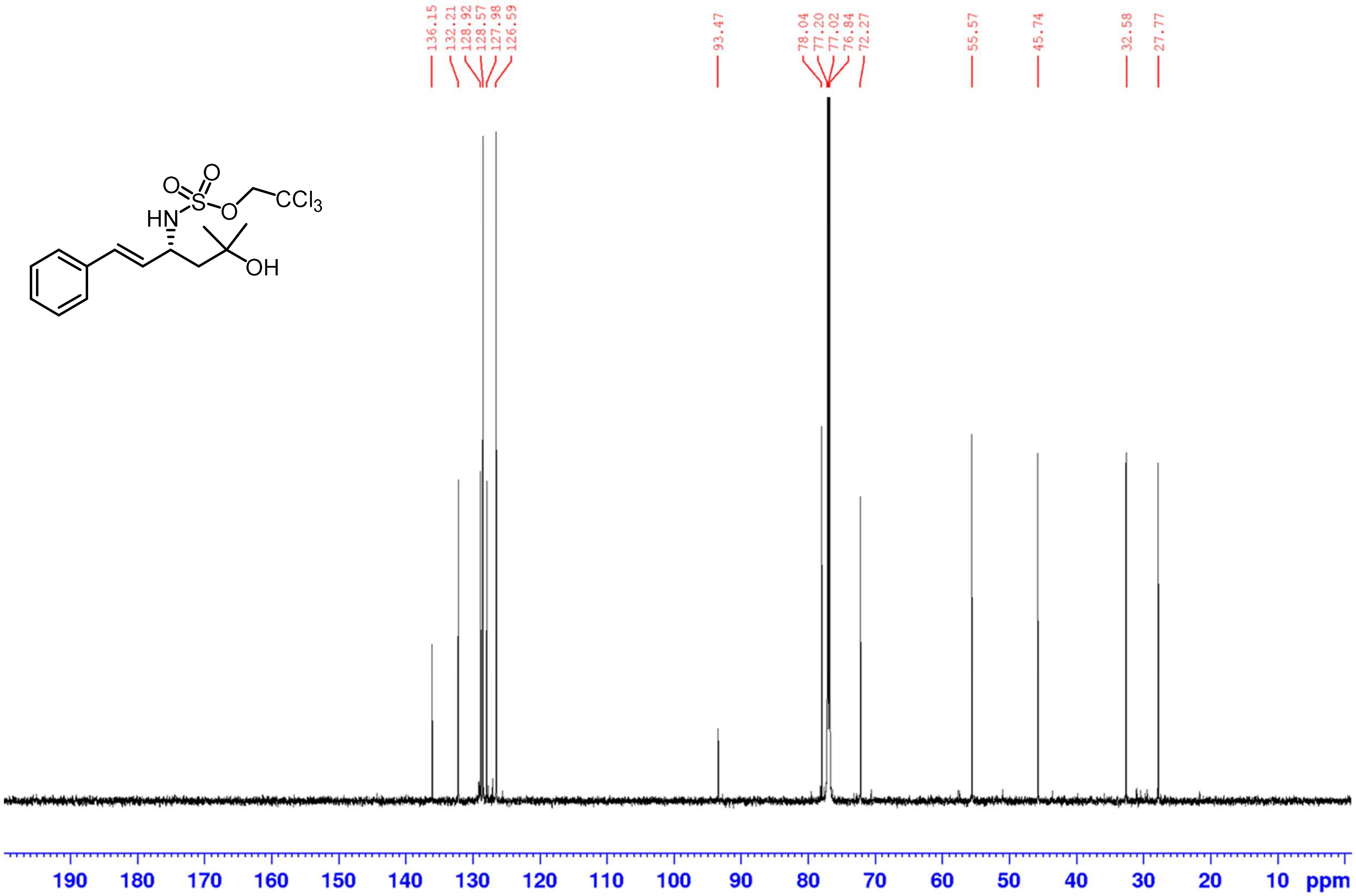

^{1}H NMR (700 MHz, $CDCl_3$) for ***tert-Butyl (R,E)-(6-hydroxy-1-phenylhex-1-en-3-yl)carbamate***

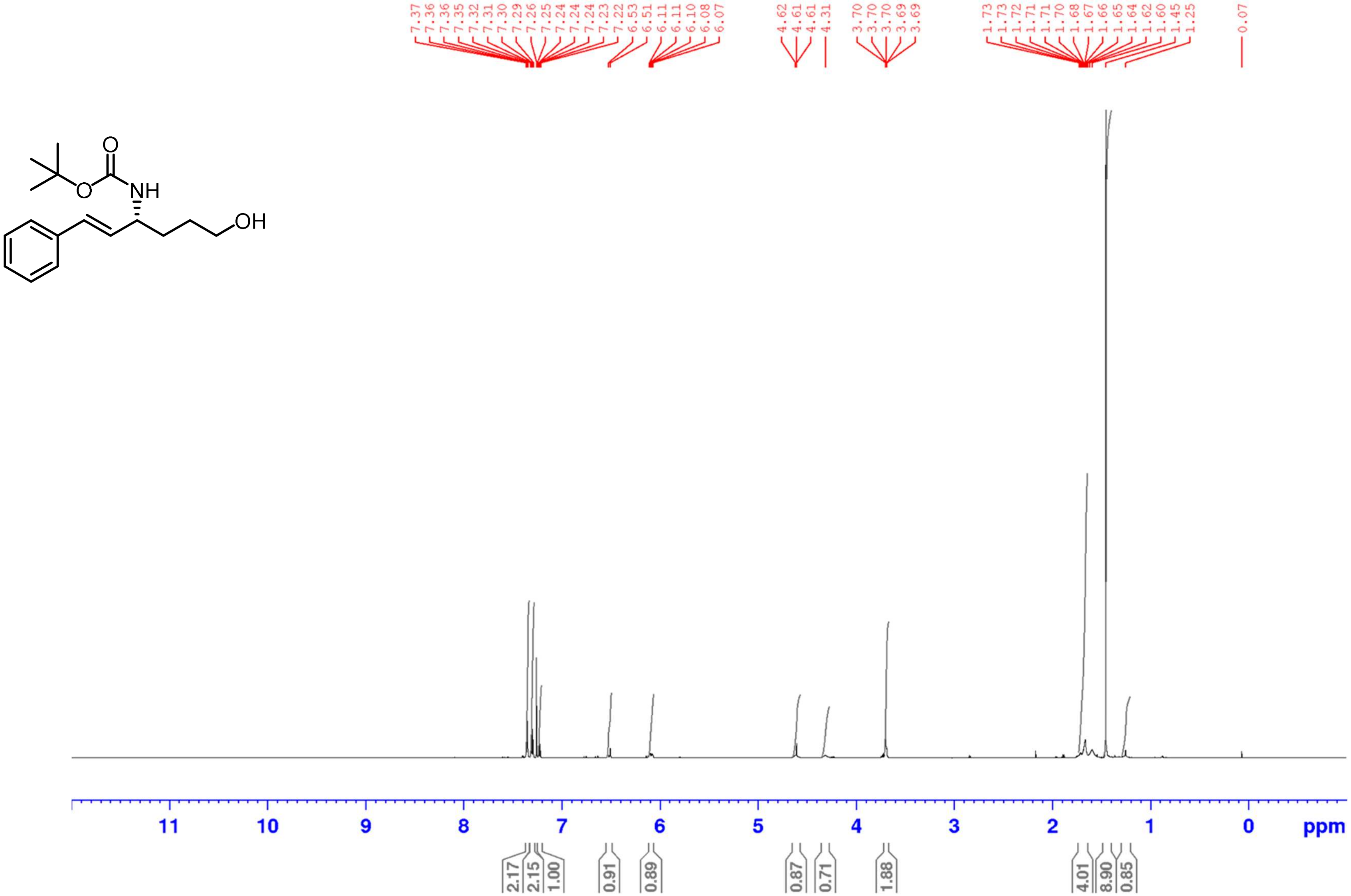

^{13}C NMR (176 MHz, $CDCl_3$) for ***tert-Butyl (R,E)-(6-hydroxy-1-phenylhex-1-en-3-yl)carbamate***

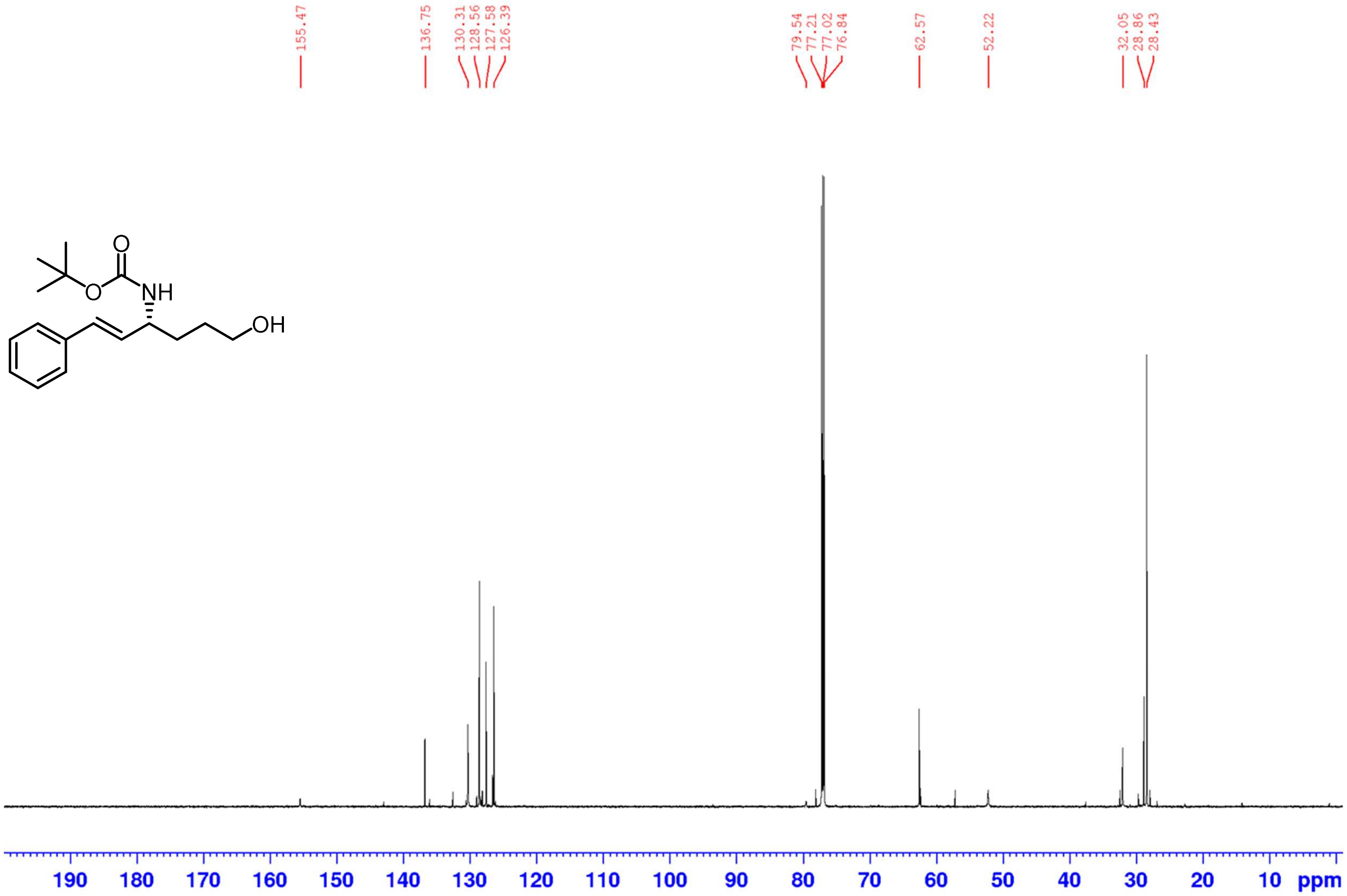

^{1}H NMR (700 MHz, $CDCl_3$) for ***Methyl (S,E)-3-(2-(1-((2,2,2-trichloroethoxy)sulfonyl)pyrrolidin-2-yl)vinyl)benzoate*** **(7)**

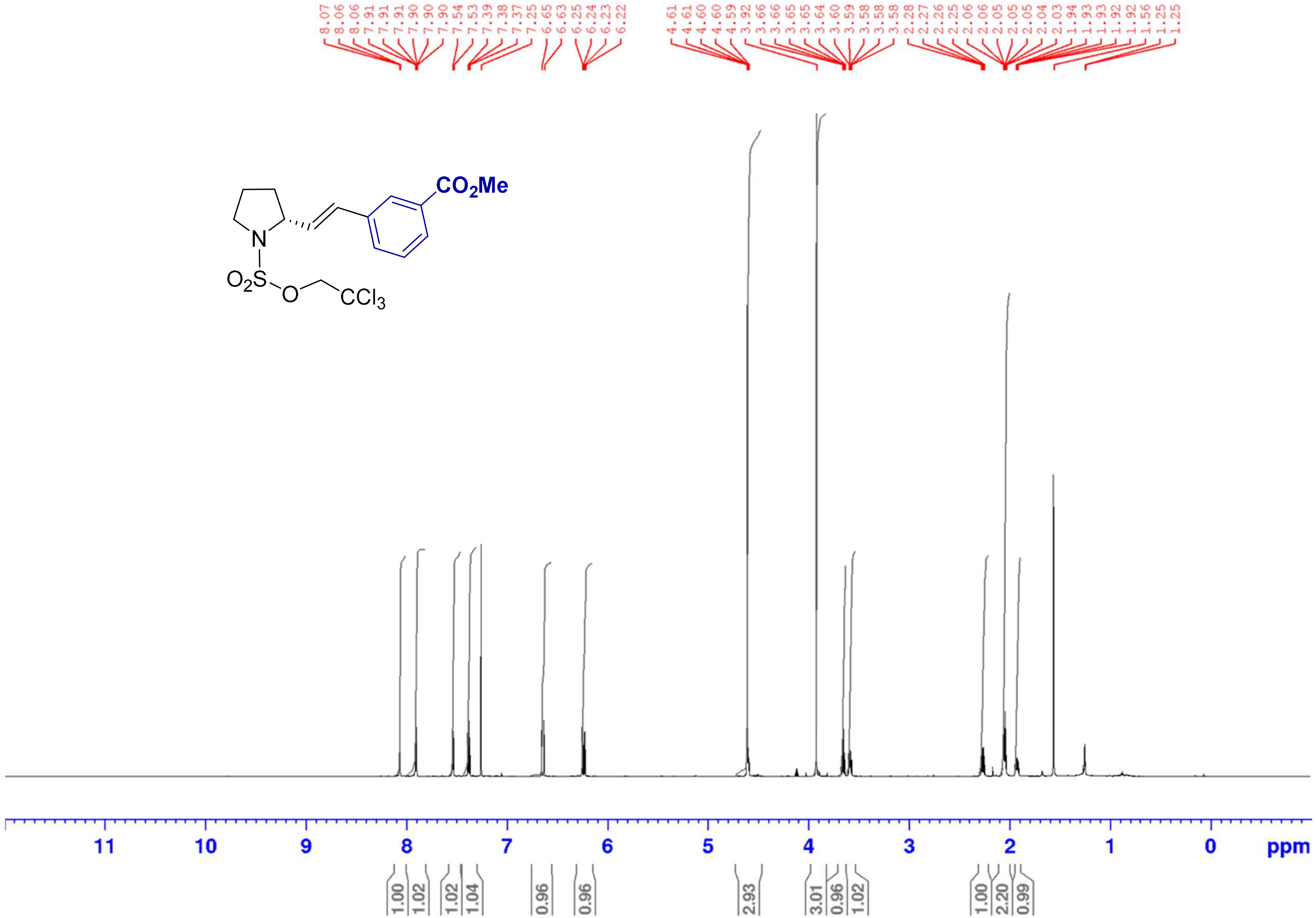

^{13}C NMR (176 MHz, $CDCl_3$) for ***Methyl (S,E)-3-(2-(1-((2,2,2-trichloroethoxy)sulfonyl)pyrrolidin-2-yl)vinyl)benzoate***

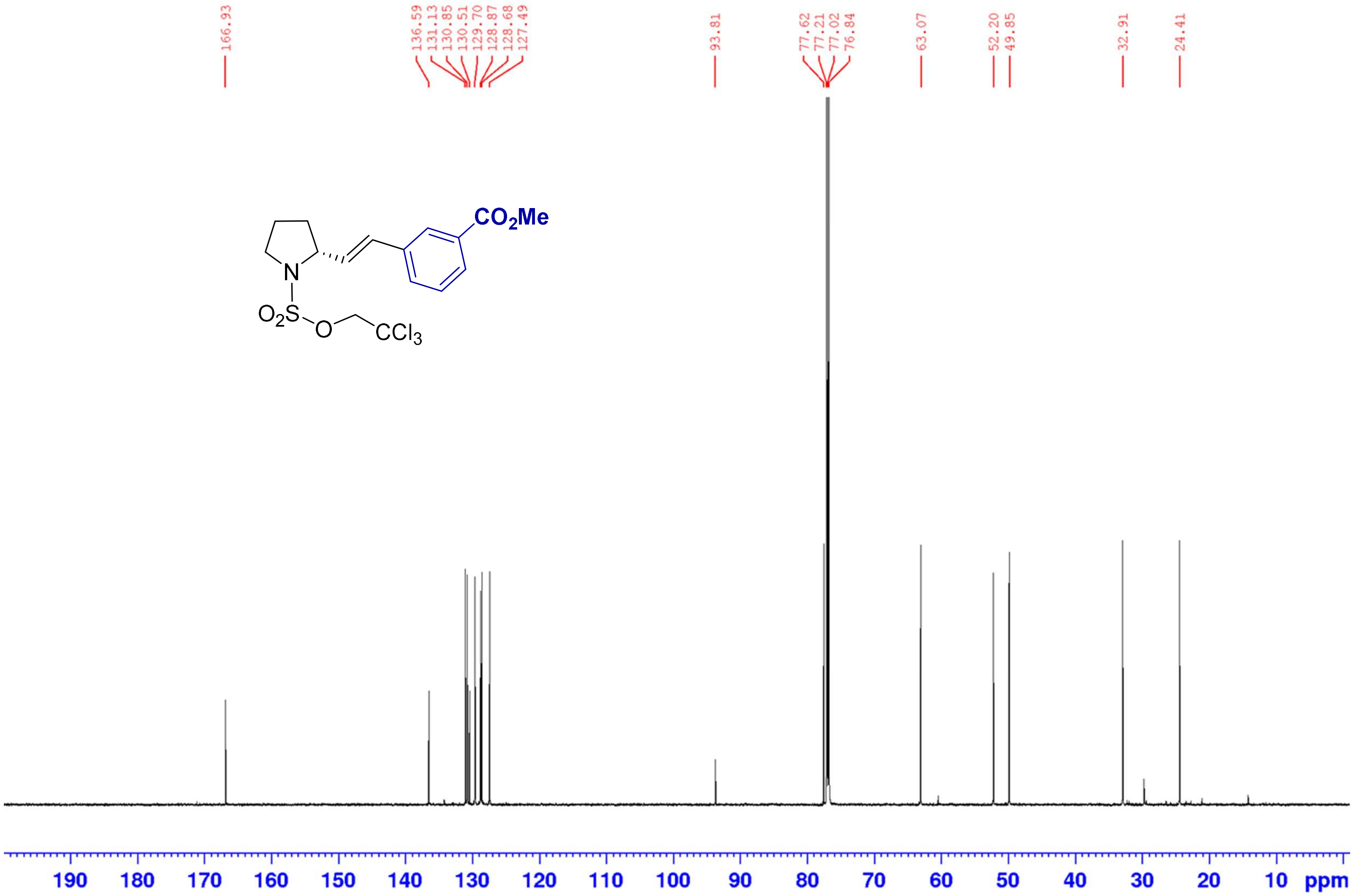

12. References

1. Gottlieb, H. E.; Kotlyar, V.; Nudelman, A., NMR Chemical Shifts of Common Laboratory Solvents as Trace Impurities. *The Journal of Organic Chemistry* **1997,** *62*, 7512-7515.
2. Piotto, M.; Bourdonneau, M.; Elbayed, K.; Wieruszeski, J.-M.; Lippens, G., New DEFT sequences for the acquisition of one-dimensional carbon NMR spectra of small unlabelled molecules. *Magnetic Resonance in Chemistry* **2006,** *44*, 943-947.
3. Sen, B.; Beraa, M.; Singh, M. S.; Hajra, S., Asymmetric total synthesis of (+)-propolisbenzofuran B. *Chem. Comm.* **2023,** *59*, 8254-8257.
4. Bera, S.; Fan, C.; Hu, X.; Bera, S.; Fan, C.; Hu, X., Enantio- and diastereoselective construction of vicinal C(sp3) centres via nickel-catalysed hydroalkylation of alkenes. *Nat. Catal.* **2022,** *5*, 1180-1187.
5. Musacchio, A. J.; Nguyen, L. Q.; Beard, G. H.; Knowles, R. R., Catalytic Olefin Hydroamination with Aminium Radical Cations: A Photoredox Method for Direct C–N Bond Formation. *J. Am. Chem. Soc.* **2014,** *136*.
6. Lu, S.-M.; Xiao, W.-J.; Huang, W.-F.; Wu, T.-J., Studies on the Wittig Reaction: Stereoselective Synthesis and Bioactivity of 1-Aryl-6-(1,2,4-Triazol-1-Yl)-1-Hexenes. *Phosphorus, Sulfur Relat. Elem.* **2000,** *167*, 195-203.
7. Wessig, P.; Badetko, D.; Czarnecki, M.; Wichterich, L.; Schmidt, P.; Brudy, C.; Sperlich, E.; Kelling, A., Studies toward the Total Synthesis of Arylnaphthalene Lignans via a Photo-Dehydro-Diels–Alder (PDDA) Reaction. *J. Org. Chem.* **2022,** *87*, 5904-5915.
8. Takami, K.; Mikami, S.; Yorimitsu, H.; Shinokubo, H.; Oshima, K., Triethylborane-Mediated Hydrogallation and Hydroindation: Novel Access to Organogalliums and Organoindiums. *J Org Chem* **2003,** *68*, 6627-6631.
9. Costello, J. P.; Ferreira, E. M., Regioselectivity Influences in Platinum-Catalyzed Intramolecular Alkyne O–H and N–H Additions. *Org. Lett.* **2019,** *21*, 9934-9939.
10. Gu, L.; Renault, K.; Romieu, A.; Richard, J.-A.; Srinivasan, R., Synthesis and spectral properties of 6′-triazolyl-dihydroxanthene-hemicyanine fused near-infrared dyes. *New. J. Chem.* **2020,** *44*, 12208-12215.
11. Nguyen, B. H.; Perkins, R. J.; Smith, J. A.; Moeller, K. D., Solvolysis, Electrochemistry, and Development of Synthetic Building Blocks from Sawdust. *J. Org. Chem.* **2015,** *80*, 11953-11962.
12. Ma, X.; Hazelden, I. R.; Langer, T.; Munday, R. H.; Bower, J. F., Enantioselective Aza-Heck Cyclizations of N-(Tosyloxy)carbamates: Synthesis of Pyrrolidines and Piperidines. *Journal of the American Chemical Society* **2019,** *141*, 3356-3360.
13. Dulayymi, J. a. R. A.; Baird, M. S.; Roberts, E.; Minnikin, D. E., The synthesis of single enantiomers of meromycolic acids from mycobacterial wax esters. *Tetrahedron* **2006,** *62*, 11867-11880.
14. Varseev, G. N.; Maier, M. E., Total Synthesis of (±)-Symbioimine. *Angew. Chem. Int. Ed.* **2006,** *45*, 4767-4771.
15. Farndon, J. J.; Young, T. A.; Bower, J. F., Stereospecific Alkene Aziridination Using a Bifunctional Amino-Reagent: An Aza-Prilezhaev Reaction. *J. Am. Chem. Soc.* **2018,** *140*, 17846-17850.
16. Logan, A. W. J.; Parker, J. S.; Hallside, M. S.; Burton, J. W., Manganese(III) Acetate Mediated Oxidative Radical Cyclizations. Toward Vicinal All-Carbon Quaternary Stereocenters. *Org. Lett.* **2012,** *14*, 2940-2943.

17. Ward, A. F.; Wolfe, J. P., Highly Diastereoselective Pd-Catalyzed Carboetherification Reactions of Acyclic Internal Alkenes. Stereoselective Synthesis of Polysubstituted Tetrahydrofurans. *Org. Lett.* **2010,** *12*, 1268-1271.
18. Fanourakis, A.; Williams, B. D.; Paterson, K. J.; Phipps, R. J., Enantioselective Intermolecular C–H Amination Directed by a Chiral Cation. *Journal of the American Chemical Society* **2021,** *143*, 10070-10076.
19. Fanourakis, A.; Hodson, N. J.; Lit, A. R.; Phipps, R. J., Substrate-Directed Enantioselective Aziridination of Alkenyl Alcohols Controlled by a Chiral Cation. *Journal of the American Chemical Society* **2023,** *145*, 7516-7527.
20. Hodson, N. J.; Takano, S.; Fanourakis, A.; Phipps, R. J., Enantioselective Nitrene Transfer to Hydrocinnamyl Alcohols and Allylic Alcohols Enabled by Systematic Exploration of the Structure of Ion-Paired Rhodium Catalysts. *Journal of the American Chemical Society* **2024,** *146*, 22629-22641.
21. Wu, Y.; Hu, L.; Li, Z.; Deng, L.; Wu, Y.; Hu, L.; Li, Z.; Deng, L., Catalytic asymmetric umpolung reactions of imines. *Nature* **2015,** *523*, 445-450.
